# Thin and thick primary cutaneous melanomas reveal distinct patterns of somatic copy number alterations

**DOI:** 10.18632/oncotarget.8758

**Published:** 2016-04-15

**Authors:** Valentina Montagnani, Matteo Benelli, Alessandro Apollo, Chiara Pescucci, Danilo Licastro, Carmelo Urso, Gianni Gerlini, Lorenzo Borgognoni, Lucio Luzzatto, Barbara Stecca

**Affiliations:** ^1^ Core Research Laboratory, Istituto Toscano Tumori, Florence, Italy; ^2^ Diagnostic Genetics Unit, Careggi University Hospital, Florence, Italy; ^3^ CBM – Genomics, Area Science Park, Basovizza, Trieste, Italy; ^4^ Anatomic Pathology Unit, Dermatopathology Section, S.M. Annunziata Hospital, Florence, Italy; ^5^ Plastic Surgery Unit, S.M. Annunziata Hospital, Regional Melanoma Referral Center, Istituto Toscano Tumori, Florence, Italy; ^6^ Department of Oncology, Careggi University Hospital, Florence, Italy; ^7^ Center for Integrative Biology, University of Trento, Trento, Italy

**Keywords:** melanoma, somatic copy number alterations, exome sequencing

## Abstract

Cutaneous melanoma is one of the most aggressive type of skin tumor. Early stage melanoma can be often cured by surgery; therefore current management guidelines dictate a different approach for thin (<1mm) *versus* thick (>4mm) melanomas. We have carried out whole-exome sequencing in 5 thin and 5 thick fresh-frozen primary cutaneous melanomas. Unsupervised hierarchical clustering analysis of somatic copy number alterations (SCNAs) identified two groups corresponding to thin and thick melanomas. The most striking difference between them was the much greater abundance of SCNAs in thick melanomas, whereas mutation frequency did not significantly change between the two groups. We found novel mutations and focal SCNAs in genes that are embryonic regulators of axon guidance, predominantly in thick melanomas. Analysis of publicly available microarray datasets provided further support for a potential role of Ephrin receptors in melanoma progression. In addition, we have identified a set of SCNAs, including amplification of *BRAF* and ofthe epigenetic modifier *EZH2*, that are specific for the group of thick melanomas that developed metastasis during the follow-up. Our data suggest that mutations occur early during melanoma development, whereas SCNAs might be involved in melanoma progression.

## INTRODUCTION

Cutaneous melanoma is a malignant tumor with a number of rather unique characteristics. In terms of etiology, there is strong evidence that exposure to sunlight/UV light is a causative factor [1]. Concerning the biology, although melanoma is a skin tumor, it originates from the neural crest and contains stem cells with distinctive features [2]. With respect to clinical course, whereas early stage melanoma can be cured by surgery, metastatic melanoma is a highly lethal condition because, although it may respond to MAPK pathway inhibitors and/or immunotherapy, to date it is rarely cured [3].

Driver mutations in *BRAF* and *NRAS* are the most prevalent oncogenic alterations in melanoma [4]. However, these mutations cannot fully explain melanoma oncogenesis, as they are found at similar rates also in benign nevi [5]. These skin lesions infrequently undergo malignant transformation into melanoma, but remain in their growth-arrested state and undergo senescence. This implies that additional genomic changes must be involved in transformation to melanoma. In recent years, genomic sequencing studies of melanoma have uncovered mutations in multiple genes [6–10]. However, the majority of these studies have investigated advanced primary or metastatic melanomas, but to our knowledge there are no data on early stages.

At the moment there is no way to predict whether surgical excision of a melanoma will be a definitive cure or not. We reasoned that comparative analysis of thin (< 1mm) and thick (> 4mm) melanomas with their different clinico-pathological features could be informative. First, thick melanomas have high risk of recurrence, whereas thin melanomas are at low risk [11, 12]. Second, a thick melanoma that after excision has produced metastasis could help to tell us which one(s) among many mutations drive metastasis. Third, a rare thin melanoma with poor prognosis would be complementary to the above and might give a clear pointer to metastasis-driving alterations. Accordingly, we have carried out whole-exome sequencing of 5 thin and 5 thick primary melanomas.

## RESULTS

### Genetic alterations in thin and thick primary melanomas

**Figure 1:**
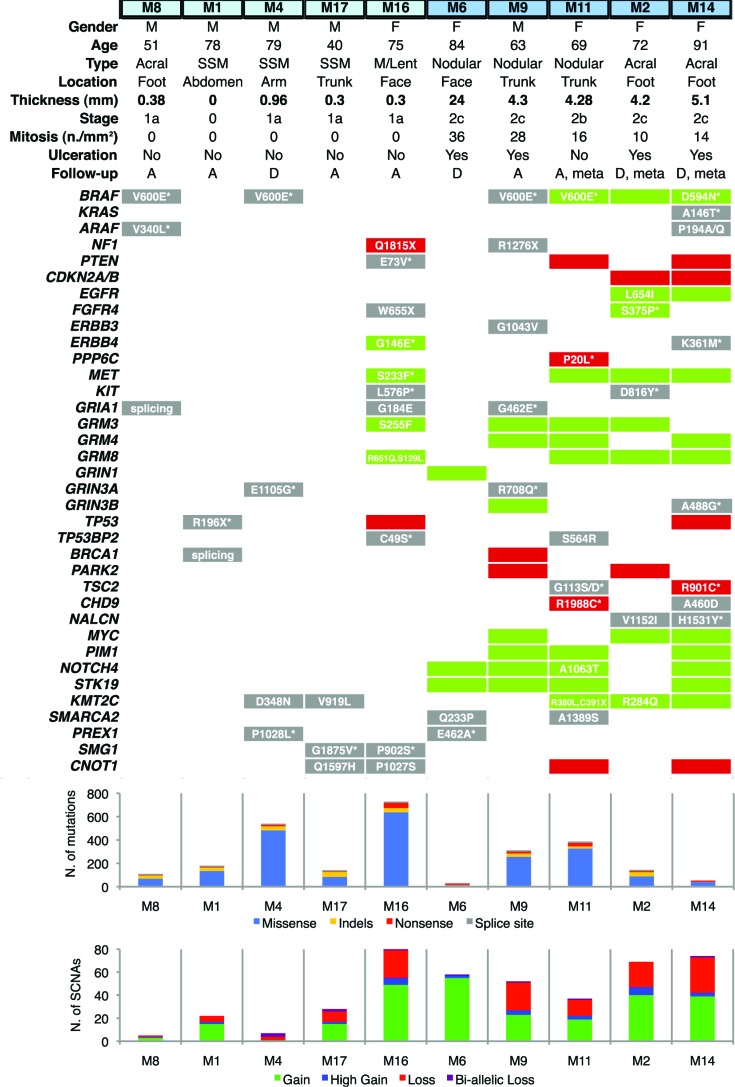
Distribution of genetic alterations in melanoma-related genes in thin and thick primary melanomas Thin melanomas are labeled in light blue and thick in deep blue (top). Gene copy gains are shown by green bars and copy losses by red bars. Point mutations are shown in a grey background; when associated with concomitant copy gain/loss of the gene, mutations are shown in a green/red background. Histograms show total number of mutations (upper) and of SCNAs (bottom). Copy gains are classified as duplications (gain) or as amplification of more than 2 times (high gain). Copy losses are classified as heterozygous deletions (loss) or homozygous deletions (bi-allelic loss). M: male; F: female; SSM: Superficial Spreading Melanoma; M/Lent: Microinvasive melanoma on Lentigo; A: alive; D: deceased; meta: melanomas that produced metastasis during follow-up; SCNA: Somatic Copy Number Alteration. Asterix denotes that the amino acid substitution is potentially damaging. All reported genes are expressed in melanocytes and/or malignant melanoma.

In total, we identified 3815 mutations: 1192 were synonymous and 2623 non-synonymous. The latter included 2150 missense, 138 non-sense, 58 splice-site variants and 277 small insertions/deletions (Supplementary Table). Thin melanomas had higher mutation frequency (340±124.4, mean±SEM) compared to thick melanomas (184.6±70.9, mean±SEM), although this difference was not statistically different (*p* = 0.3) due the limited number of samples and the high variability among them. Analysis of SCNAs revealed a total of 289 gains and 143 losses across all samples; SCNAs were more numerous in thick (58±6.5) than in thin (28.4±13.6) melanomas (Figure 1). Four out of ten melanomas had the *BRAF* V600E mutation and one a non-canonical *BRAF* D594N mutation. The latter presented also a missense mutation of *KRAS* (A146T) and one of *ARAF* (P194A/Q). Alterations in the tumor suppressor *NF1*, a negative effector of Ras [13], were present in a thin melanoma (M16, non-sense mutation and copy loss) harboring wt *BRAF* and in a thick melanoma (M9, non-sense mutation) with *BRAF* V600E. *KIT* missense mutations were present in M16 (L576P) and M2 (D816Y), respectively a thin and a thick melanoma harboring wt *BRAF*. Several melanoma-related genes were found altered (mutated and/or affected by SCNAs), including protein tyrosine kinases (*EGFR, FGFR4, ERBB3, ERBB4, MET*), members of DNA damage response pathway (*TP53, TP53BP2, BRCA1*), ionotropic and metabotropic glutamate receptors (*GRIA1, GRIN3A, GRIN3B, GRM3, GRM4, GRM8*), the methyltransferase *KMT2C (MLL3),* the negative regulator of mTOR pathway *TSC2*, the RAC exchange factor *PREX1*, *NOTCH4* and the protein phosphatase *PPP6C* (Figure 1). Most of these mutations were predicted to be damaging by Sorting Intolerant from Tolerant (SIFT) or by PolyPhen2 algorithms.

The thin microinvasive melanoma M16 (thickness 0.3mm) had the highest number of mutations and of SCNAs. The thin melanoma M4 (thickness 0.96mm) was the second most mutated tumor but it presented a very low number of SCNAs (Figure 1). This patient died 2 years after diagnosis from complications of influenza vaccine in presence of severe cardiac disease. Among patients with thick melanomas, M6 died of pneumonia. Patients M11, M2 and M14 developed metastases and two of them (M2 and M14) died during follow-up.

### Analysis of somatic copy number alterations distinguishes between thin and thick melanomas

**Figure 2:**
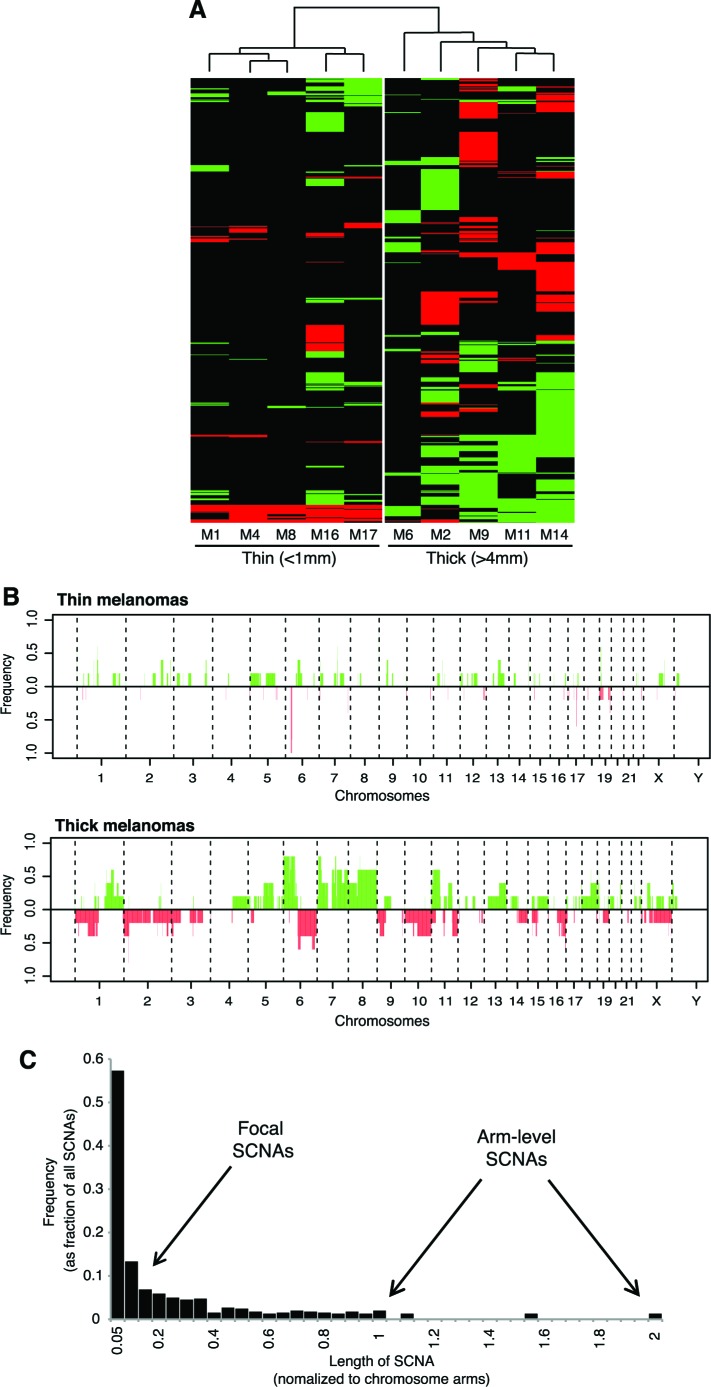
Different pattern of SCNAs in thin *versus* thick melanomas **A.** Unsupervised clustering analysis of SCNAs in thin and thick primary melanomas. Copy gains are indicated in green and copy losses in red. **B.** Frequency plot of copy gains (green) and copy losses (red). **C.** Length distribution of SCNAs across the 10 melanomas. Note that the majority (79%) of SCNAs are focal (less than 20% of the chromosome arm).

Unsupervised hierarchical clustering analysis of SCNAs identified two distinct clusters corresponding to thin and thick melanomas, suggesting distinct genetic differences between those two groups (Figure 2A). Consistently, frequency plot showed high frequency of copy losses and copy gains in thick melanomas, whereas thin melanomas presented only few (Figure 2B). The most prevalent SCNAs were very short (focal SCNAs, 79%) (Figure 2C). Only few SCNAs involved more than 45% of the chromosome arm. These aberrations were detected only in four thick melanomas, three of which gave rise to metastasis during follow-up. We detected arm-level SCNAs, including complete gain of 8q (M2, M14) and 8p (M9), complete loss of 19q (M9), of 10p (M14), and entire gain of chromosome 18 (M14). Common aberrations involved gain of chromosome arms 6p, 7p, 8p, 11p and 18q, and losses of 6q and 9p (Supplementary Figure 1).

**Figure 3:**
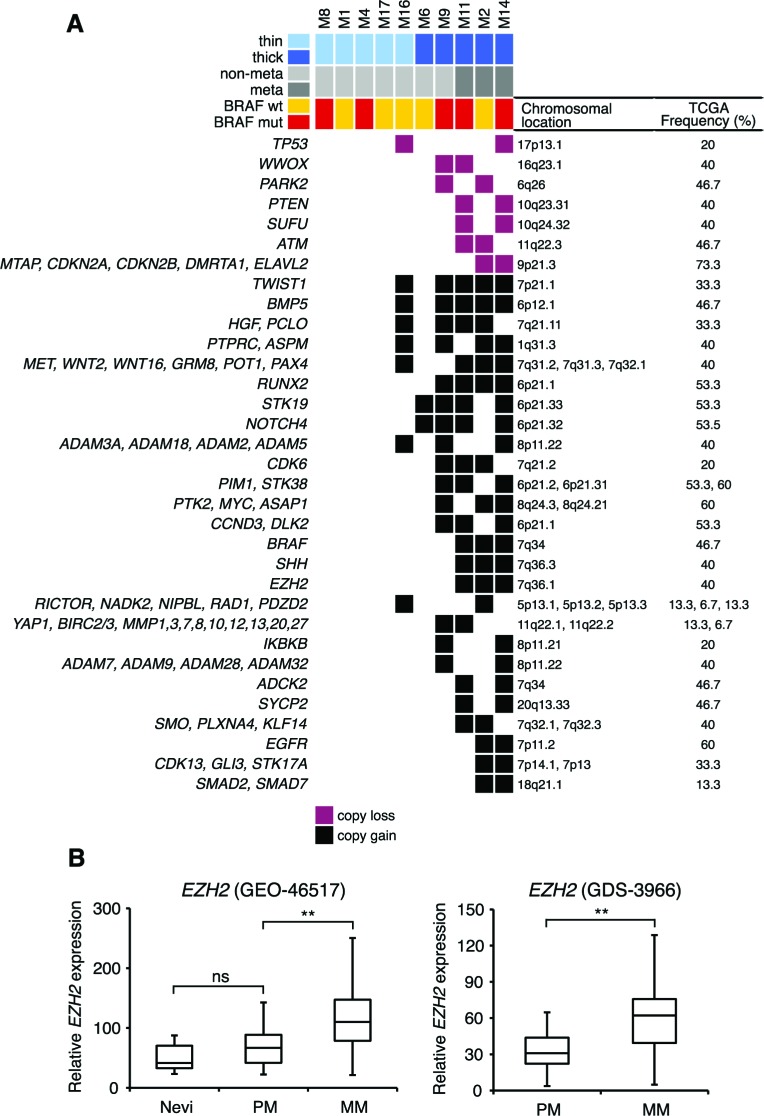
Recurrent focal SCNAs in primary melanomas **A.** Only recurrent (present in at least 20% of melanomas) SCNAs are shown. Focal SCNAs were observed mainly in thick melanomas. Known and putative tumor suppressor genes are present in regions with copy losses; several oncogenes are present in regions of copy gains. Frequencies of SCNAs were validated in primary melanomas in the TCGA database. **B.** Expression of *EZH2* mRNA in two different microarray data sets (GEO-46517 and GDS-3966). In GEO-46517 nevi (*n* = 9), primary (*n* = 31) and metastatic (*n* = 73) melanomas. In GDS-3966 primary melanomas (*n* = 31) and metastatic melanomas (*n* = 52). PM, primary melanomas; MM, metastatic melanomas. **, *p* < 0.001.

Mapping of focal SCNAs has a great power to pinpoint important genes targeted by loss or gain events [14]. Therefore, we analyzed regions with recurrent (present in at least 2 out of 10 samples) focal SCNAs. The majority of focal SCNAs were found in thick melanomas (Supplementary Figure 2A). Among regions with copy losses we identified several known and putative tumor suppressors, including *CDKN2A, CDKN2B, p53*, *WWOX, PARK2, PTEN, SUFU* and *ATM,* consistent with a previous report [6] (Figure 3A). Recurrent focal amplifications of 6p12.1 and 7p21.1, that contain the TGFβ member *BMP5* and *TWIST1,* respectively, occurred in 50% of the samples. Other recurrent amplifications were found in 40% of samples at 7q21.11 (*HGF*), 7q31-32 (*MET, WNT2, WNT16, GRM8, POT1* and *PAX4*), 1q31.3 (*PTPRC* and *ASPM*) and 6q21 (*RUNX2*, *NOTCH4* and *STK19)*. Two other amplifications (involving *c-MYC* and *PIM1*) were found, respectively, on chromosomes 8p24.21 and 6p21.2 in 30% of the samples. The presence of the *BRAF* V600E mutation was associated with a lower number of focal SCNAs (15.8±6.2, mean±SEM) compared to tumors not carrying this mutation (46.5±7.8, mean±SEM), although not statistically significant (*p* = 0.08) (Supplementary Figure 2B) due the limited number of samples.

### Alterations found in primary melanomas that developed metastasis

We reasoned that SCNAs and mutated genes present in those three thick melanomas that gave rise to metastasis during follow-up (M11, M2, M14) and not in the other seven samples must be regarded as candidates for being involved in metastasis. All three melanomas that developed metastasis displayed amplification of BRAF at 7q34 (amplicon size, 6 genes), which is a common event in melanoma [6, 15] and it is associated with worse clinical outcome [16]. Those three melanomas presented also amplification of 7q36.1 (amplicon size, 78 genes), which includes the epigenetic modifier *EZH2.* Amplification of *EZH2* locus in melanomas that developed metastasis is consistent with the higher *EZH2* mRNA expression in metastatic compared with primary melanomas (*p* < 0.001) in public microarrays (Figure 3B). This finding suggests that EZH2 might be a candidate gene involved in melanoma metastasis, in line with a recent report [17]. Other “metastasis-associated” recurrent amplifications occurred at 7q36.3 (*SHH*), at 7p11.2 (*EGFR*), at 7q32 (*SMO, PLXNA4, KLF14*) and at 7q34 (*ADCK2*) (Figure 3A). Among genes mutated in at least two of those three melanomas, we identified *TSC2* (tuberous sclerosis 2), *CHD9* (chromodomain-helicase-DNA-binding protein 9) and *NALCN* (sodium leak channel, non-selective) (Figure 1).

### Alterations in axon guidance genes

We identified mutations and SCNAs in axon guidance genes, particularly Slit/Robo signaling, Ephrins and Semaphorins/Plexins. These are important regulators of normal neural migration and positioning during embryonic development. Recently, they have been implicated in cancer cell growth, survival, invasion and angiogenesis [18]; however, aberrations of this class of genes have never been reported in melanoma.

**Figure 4:**
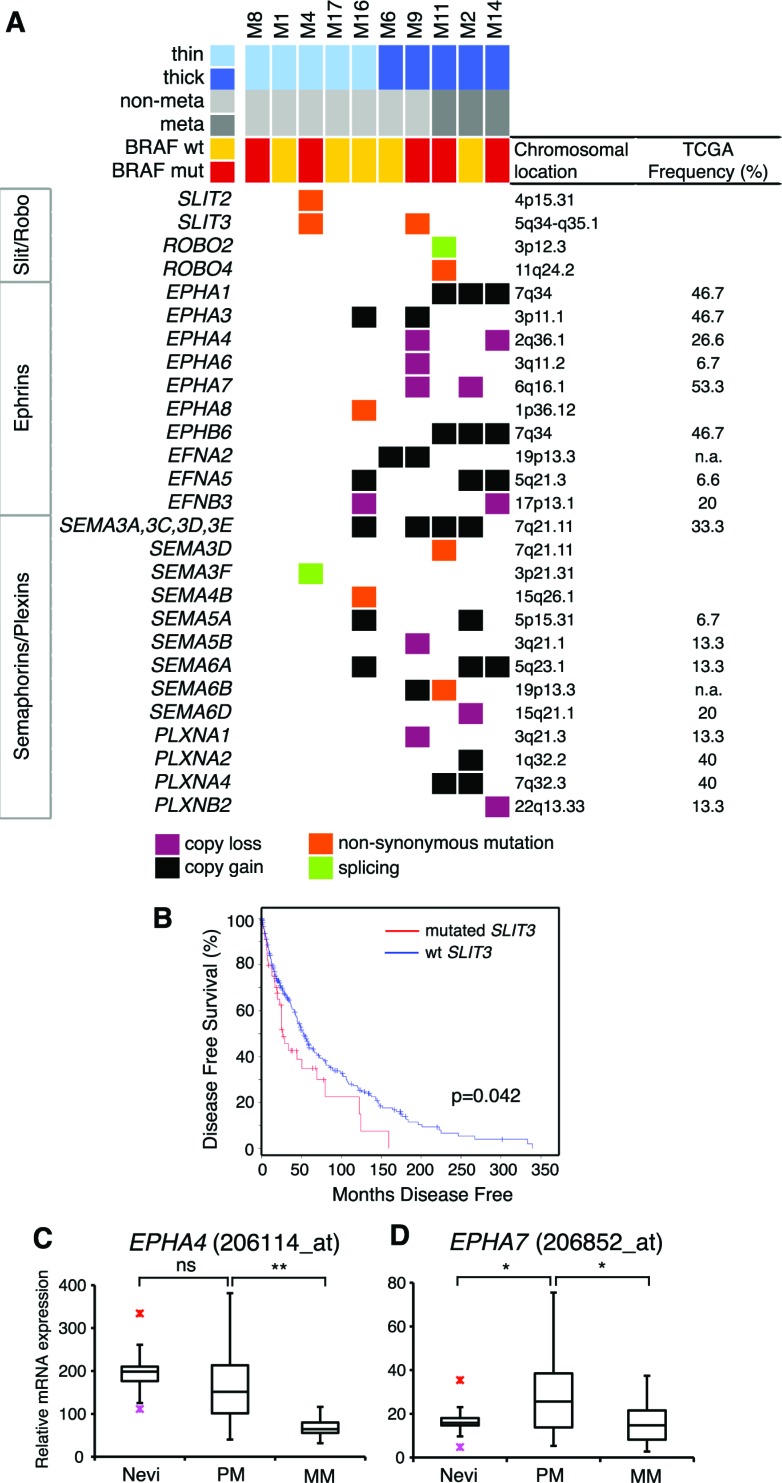
Mutations and SCNAs in axon guidance genes **A.** Axon guidance genes with mutations and/or SCNAs. Frequencies of SCNAs were validated in primary melanomas in the TCGA database (on the right). **B.** Kaplan-Meier disease free survival in patients with wild type *SLIT3* (*n* = 254, median months 52) compared with those with mutated *SLIT3* (*n* = 45, median months 26.3) (*p* = 0.042, log-rank test). Disease free survival curve was obtained from cBioportal database. **C**.-**D**. Expression of *EPHA4* (C) and *EPHA7* (D) mRNA in human nevi (*n* = 9), primary (*n* = 31) and metastatic (*n* = 73) melanomas, as determined by publicly available microarray data set (GEO-46517). PM, primary melanomas; MM, metastatic melanomas. Red crosses indicate outliner values. *, *p* < 0.05; **, *p* < 0.001.

Slit glycoproteins (SLIT1-3) signal through their Roundabout receptors (ROBO1-4) to elicit their effects inside the cell [19]. Missense mutations in *SLIT3* were found in M4 (S268L) and in M9 (G575R). The latter is located in the leucine-rich repeat domain, which is involved in protein-protein interaction. Melanoma M4 had also a concomitant missense mutations in *SLIT2* (L224F) (Figure 4A). All these mutations were predicted to be damaging or probably damaging by SIFT or PolyPhen2. Consistently, *SLIT3* was reported mutated in 15% of human melanomas in cBioPortal database (http://www.cbioportal.org/). Down-regulation of *ROBO1* is part of a molecular signature that predicts the metastatic risk associated with cutaneous melanoma [20], therefore we examined the survival of patients with *SLIT3* mutations. We found that patients with wild type *SLIT3* had a prolonged disease free survival (median months 52) compared with those harboring mutated *SLIT3* (median months 26.3) (*p* = 0.042, log-rank test, Figure 4B). These results altogether suggest that aberrant Slit/Robo signaling might be a potential feature of human melanoma and that mutations in *SLIT3* might associate with poor clinical outcome.

Several classes of Ephrin receptors and Ephrin ligands exhibited SCNAs, particularly in thick melanomas (Figure 4A). *EPHA1, EPHA3* and *EPHB6* were contained in regions of copy gain, suggesting their oncogenic role during melanoma progression. Interestingly, we found both *EPHA4* and *EPHA7* in regions of copy loss in 20% of the samples (Figure 4A). Deletion of *EPHA4* and *EPHA7* loci in thick melanomas is consistent with decreased expression of *EPHA4* and *EPHA7* mRNA in metastatic compared to primary melanomas (Figure 4C-4D, Supplementary Figure 3A). The Ephrin ligand *EFNB3* was contained in a region of copy loss and *EFNB3* mRNA expression was significantly decreased in metastatic compared to primary melanomas (Supplementary Figure 3B). These findings suggest that EPHA4, EPHA7 and EFNB3 might act as tumor suppressors during melanoma progression.

Class 3 semaphorins exhibited SCNAs and mutations (Figure 4A). In particular, amplification of *SEMA5A* and *SEMA6A* loci (found in 20% and 30% of the melanoma, respectively) were consistent with their increased mRNA expression in melanomas compared to nevi (Supplementary Figure 4). These results are in line with the role of SEMA5A in promoting invasion of gastric cancer cells [21] and of SEMA6A in controlling cell growth of BRAF^V600E^ mutant melanomas [22]. Semaphorins signal through Plexin and Neuropilin receptors to elicit their effects inside the cells [23]. Plexins A and B were also found altered in our cohort, although at lesser extent than Semaphorins. *PLXNA1* and *PLXNB2* were in a region of copy loss in 10% of the samples and *PLXNA4* was in a region of copy gain in two of the three melanomas that produced metastasis (Figure 4A), suggesting that human melanomas harbor alterations of Plexins, in particular *PLXNA4* amplification, as previously reported [24].

### Pathway analysis

**Figure 5:**
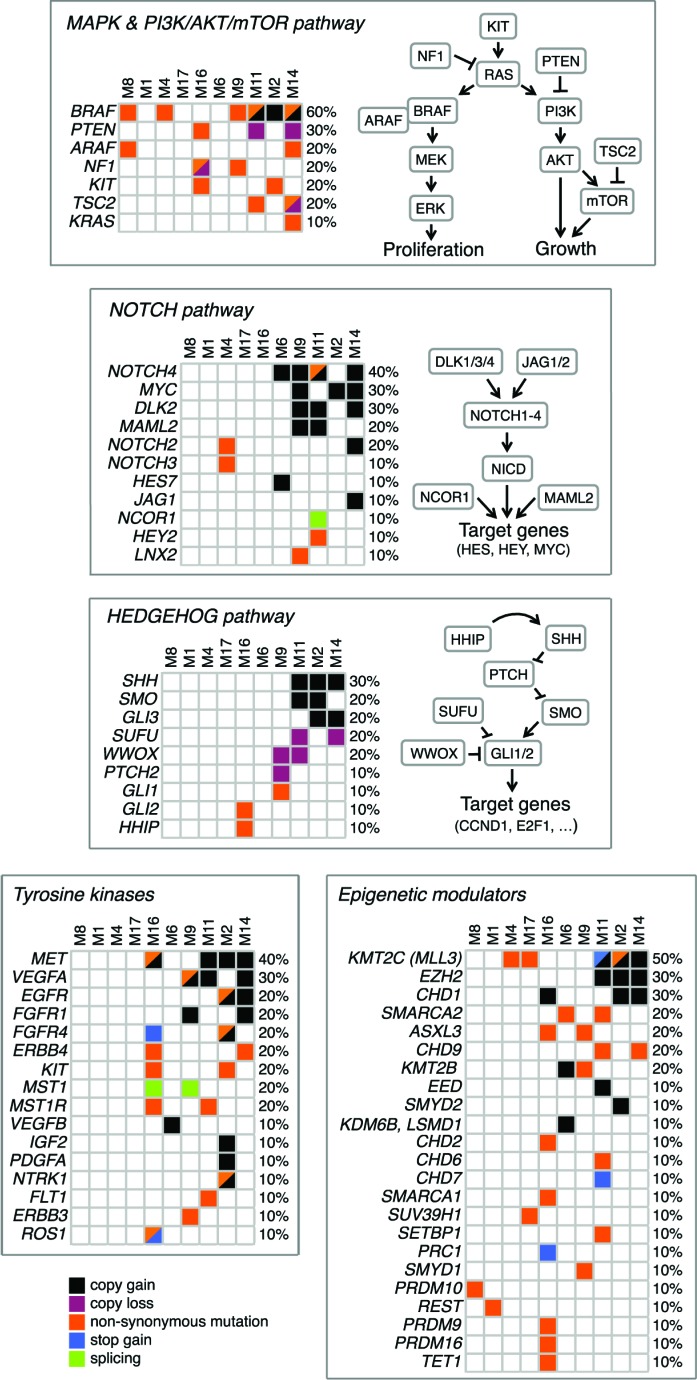
Altered pathways in primary melanomas Schematics of pathways with mutations and SCNAs occurring in at least one patient. All reported genes are expressed in melanocytes and/or malignant melanoma.

In addition to the MAPK pathway, we found alterations in multiple additional pathways, including NOTCH and HEDGEHOG signaling, several tyrosine kinases and epigenetic regulators (Figure 5). Specifically, the HEDGEHOG pathway exhibited alterations indicative of activation, such as amplification of the transmembrane receptor *SMOOTHENED (SMO)* and of the ligand Sonic Hedgehog (*SHH*), and missense mutations in the highly conserved region of the transactivator domain of *GLI1*, the downstream effector of the HH signaling. In addition, we observed copy losses of the negative regulators *SUFU*, as well as of the tumor suppressor *WWOX,* a newly identified negative modulator of GLI1 in breast cancer [25]. Similarly, aberrant activation of the NOTCH pathway is suggested by the alterations in several NOTCH pathway components, such as copy gains of the ligand *JAG1*, the receptors *NOTCH4* and *NOTCH2,* the co-activator *MAML2*, and the targets *HES7* and *MYC*. Among tyrosine kinases, we identified a region of amplification (7q31.2) in 40% of melanomas that contained the MET oncogene, which is involved in development and progression of melanoma [26]. Missense mutations were found in *ERBB4, ERBB3, KIT, FGFR4* and several other kinases. Multiple epigenetic regulators were found mutated or involved in regions of copy gains. For instance, the histone-lysine N-methyltransferase *MLL3 (KMT2C)* was altered in 50% of the samples. Other epigenetic modulators found frequently mutated are the member of the SWI/SNF family *SMARCA2*, and *ASXL3* and *CHD9*.

Many of these altered pathways represent therapeutic targets that are actionable in preclinical models and in the clinic [27–29]. For example, the NOTCH pathway can be targeted by the MK-0752, an inhibitor of γ-secretase in phase I clinical trial for patients with advanced solid tumors, including melanoma [30]. Activation of cMYC and Hedgehog pathway can be targeted by BET-bromodomain inhibition with the small molecule JQ1 [31, 32]. Similarly, the small molecule Glabrescione B has been shown to interfere with the Hedgehog pathway by inhibiting GLI1/DNA interaction in preclinical models of Hedgehog-dependent tumors [33]. The epigenetic modifier EZH2 can be targeted with the specific inhibitor GSK126 in melanoma [34]. Importantly, a number of these alterations could be targeted in combination with the BRAFV600E inhibitor vemurafenib.

## DISCUSSION

For some 20 years it has been known that thickness of cutaneous melanoma is a major prognostic factor. Thin melanoma has a much better prognosis than thick melanoma: so much so that thickness is an established criterion in dictating management. The dividing line between thin and thick has been set at 1 mm [11, 12]. It is reasonable to think that most of the thick melanomas have been thin before they became thick; a less obvious question is whether in some cases (or perhaps in the majority) thin melanoma is not just an early stage, but from the outset a different type of tumor.

In the aim to answer this question we undertook whole-exome sequencing of both thin and thick melanomas. Since our study was started, a wealth of data have accumulated on the mutational landscape of thick melanoma [6–10, 15]; but hardly any have been published on thin melanomas. In part this may be due simply to the fact that with a thin melanoma little or no material is left once histopathological examination has been properly carried out. We have been able to obtain material from a small number (*n* = 5) of thin melanomas. This has enabled us to demonstrate - for the first time to the best of our knowledge - that, although with considerable variation among samples, the number of point mutations in thin and thick melanomas is similar (Figure 1). This finding suggests that point mutations occur early during tumor development, as previously reported [8]. On the other hand, the number of SCNAs greatly increases from thin to thick melanomas (Figure 2), indicating that SCNAs might play a role in melanoma progression. A possible explanation is that thin and thick melanomas might have the same origin and tumor progression occurs through accumulation of SCNAs. However, since the number of point mutations does not increase from thin to thick melanomas, it is conceivable to hypothesize that from the outset there are two types of melanomas that differ in their tendency to undergo SCNAs: only those prone to make SCNAs are likely to progress. This is not surprising given the high number of evolving clones described in normal human skin [35].

At the moment is it not known whether the high number of SCNAs in thick melanomas might simply reflect the accumulation of new alterations during tumor progression, or whether they actually drive melanoma progression. What is clear is that an increased tendency to generate SCNAs is an important difference between thin melanomas and those that were analyzed when they were already thick. The mechanisms underlying the formation of focal SCNAs have not been fully elucidated. It can be speculated that activated oncogenes might induce breakage of DNA replication forks, leading to DNA replication stress and DNA double strand breaks [36]. A recent report suggests that break-induced replication repair of damaged forks promotes segmental duplications in the genome of cancer cells [37].

An outstanding challenge is to predict whether a thin melanoma will become thick and/or eventually develop metastasis. Indeed, there might be two types of thin melanomas: some are simply on their way to become thick, others remain thin for many years and may be intrinsically different from the former. For instance the thin melanoma M16 harbors the highest number of SCNAs and point mutations. In principle, the thickness of 0.3 mm was predictive of good prognosis; however, the set of alterations resembled that of a thick melanoma (Figure 1). Since the primary lesion was surgically removed, we cannot say how it might have otherwise evolved. In the event, this patient has no evidence of disease recurrence after a follow-up of 6 years: in spite of the fact that point mutations in several key melanoma genes (*NF1, PTEN, MET* and *KIT*) and the high number of SCNAs made us speculate that this tumor, though thin, might have become a fast-growing melanoma. This observation underscores the importance of periodical skin examination and early diagnosis.

Another interesting finding of our study is the involvement of axon guidance genes in melanoma. Axon guidance is an important component of organogenesis, regeneration, wound healing and other basic cellular processes. The numerous point mutations and SCNAs observed in axon guidance genes suggest that they might play a role in melanoma. In this respect, there are precedents in other tumors [18], including pancreatic cancers [38]. Slits have been shown to impair migration of neural crest cells [39], from which melanocytes originate. In most of the cancers, SLIT/ROBO signaling acts as a tumor suppressor by inhibiting cell invasion and migration [19]. Alterations in several components of the axon guidance signaling have been reported in brain metastases from melanomas [40]. The functional role of *SLIT3* mutations identified in this study is currently unknown. Nevertheless, the association between *SLIT3* mutations and reduced disease free survival suggests that SLIT3 might play an important role in melanoma. In agreement with a previous study showing the inhibitory role of SLIT3 in melanoma cell migration [41], we can hypothesis that SLIT3 acts as tumor suppressor in melanoma. Both oncogenic and tumor suppressor roles have been described for specific Ephrin receptors and their ligands [42]. Deletion of *EPHA4* and *EPHA7* loci, that we found in two out of five thick melanomas, correlates with their reduced expression in metastatic compared to primary melanomas, suggesting that EPHA4 and EPHA7 might act as tumor suppressor during melanoma progression. This is also supported by recent studies that showed inhibition of invasion and tumor growth by EPHA7 in follicular lymphoma and by EPHA4 in lung adenocarcinoma [43, 44].

A major signaling mode frequently altered in melanoma is the MAPK pathway, which regulates cell proliferation and survival [45, 46]. Seven out of ten melanomas have alterations in the MAPK pathway, including point mutations/SCNAs in *BRAF* and *NF1*, and mutations in *KRAS* and *ARAF*. Melanoma with *BRAF* D594N substitution had also a mutation in *KRAS* (A146T) and one in *ARAF* (P194A/Q). *BRAF* D594N is a kinase-dead protein; nevertheless, considering results in mice [47] co-occurrence of *BRAF* D594N and *KRAS* mutations might contribute to tumor progression. Some of these mutations are likely to have important implications for therapy. For instance, *KRAS* A146T and other exon 4 *KRAS* mutations are expected to respond to MEK inhibitors and to be resistant to EGFR inhibitors [48]. Likewise, *KIT* L576P, the most common *KIT* mutation in melanoma, has been shown to induce structural changes in KIT that reduce the affinity for imatinib but not for dasatinib [49]. Similarly, loss of NF1 function is associated with RAS activation, responsiveness to MEK inhibitors and, in the presence of concurrent BRAF mutations, vemurafenib resistance [50]. At any rate, our study confirms the predominance of alterations in the MAPK pathway [6, 15]. At the same time, unlike previous next-generation sequencing studies that have analyzed thick melanomas [6–8], we did not find a significant difference in mutation load between BRAF wt and BRAF V600E melanomas [8], although BRAF V600E melanomas tend to have a lower number of focal SCNAs.

In conclusion, we report for the first time an analysis of the mutational landscape of a small set of thin melanomas: they have numerous point mutations but very few SCNAs. In addition, we have identified a set of SCNAs, including amplification of *BRAF* and *EZH2,* in thick melanomas that subsequently developed metastasis. Although this finding needs to be confirmed on a larger cohort of samples, it suggests that these SCNAs might become useful prognostic markers in melanoma. In addition, thick melanomas often have alterations in multiple pathways, including MAPK, SLIT/ROBO, NOTCH and HEDGEHOG signaling, Ephrin receptors and tyrosine kinases (Figure 5). Therefore, it is not altogether surprising that targeting a single pathway results in therapeutic failure: it may be imperative to target at least two signaling pathways at one time.

## MATERIALS AND METHODS

### Melanoma samples

Fresh-frozen tissues from ten untreated primary cutaneous melanomas were collected from the Plastic Surgery Unit of the S.M. Annunziata Hospital (Florence, Italy). Samples were selected according to thickness: five melanomas were thin (< 1mm) and five thick (> 4mm). Matched patient blood was also collected to distinguish somatic from germline mutations. All patients gave informed consent and the protocol was approved by the local Ethic Committee. All patient studies were conducted in accordance with the declaration of Helsinki. Histological variables such as Breslow thickness (mm), melanoma subtypes, stage, presence of ulceration, mitotic rate (n./mm^2^) and follow-up were assessed. Only M1 was an *in situ* melanoma. Melanoma subtypes were: superficial spreading melanoma (SSM) (*n* = 3) (M1, M4, M17), nodular melanoma (*n* = 3) (M6, M9, M11), acral melanoma (*n* = 3) (M8, M2, M14) and microinvasive melanoma developed on Hutchinson lentigo (*n* = 1) (M16) (Figure 1). Samples M11, M2 and M14 developed metastases during follow-up; in patient M11 metastases occurred in the lungs, M2 had intra-abdominal and peritoneal metastases, M14 liver and lung metastases.

### Whole exome sequencing

Genomic DNA was extracted from the tumor and matched peripheral blood using the QIAamp DNA Minikit (Qiagen, Milan, Italy). One microgram of genomic DNA was sheared by sonication. Exome enrichment was conducted using the Illumina TruSeq Exome Enrichment kit (Illumina, San Diego, CA). Sequencing was carried out on an illumina HiScan SQ instrument. Samples were loaded in an indexed pool of 4 samples per lane, and an average coverage of 84x and 46x was achieved for tumor and matched normal samples, respectively (Supplementary Table), that is adequate for detecting a Single Nucleotide Variant (SNVs) [10].

### Sequencing alignment and variant calling

Reads were aligned against human reference genome (hg19) with BWA MEM [51]. GATK version 2.5.2 [52] was used to recalibrate base qualities and realign mapped reads around indels. PicardTools’ MarkDuplicates (version 1.98) was used to remove optical and PCR duplicates. In order to identify somatic SNVs and small Insertions/Deletions (InDels), matched normal/tumor samples were analysed by MuTect version 1.14 [53] and IndelGenotyperV2 of GATK, respectively. Functional annotation of somatic variants was carried out by ANNOVAR [54]. SIFT [55] and Polyphen [56] scores were used to determine the potential impact of point mutations. Somatic Copy Number Alterations (SCNAs) were detected by EXCAVATOR version 2.2 [57] (with cellularity = 0.7). Ward's hierarchical clustering of SCNAs was performed by using Pearson correlation coefficient to cluster tumor samples (columns) and the euclidean distance to cluster genomic events (rows). Frequency analysis of SCNAs was performed by bedtools [58] and R custom scripts as follow: for each 1Mbp genome window, alteration frequency (AF) for both gains and losses was calculated as the fraction of samples having at least 1 SCNAs in that genomic window. TCGA data were downloaded from The Cancer Genome Atlas (TCGA) Data Portal (https://tcga-data.nci.nih.gov/tcga/).

### Analysis of mutations and SCNAs

The BRAF mutation status was validated using Sanger sequencing. Fifteen primary melanomas (thickness ≥ 1.92mm) from the TCGA Data Portal were interrogated to confirm frequencies of SCNAs found in our samples. Two publicly available microarray data sets (GEO-46517 [59] and GDS3966), which included nevi, primary and metastatic melanoma samples profiled on Affymetrix U133 platforms, were used to assess the expression of genes involved in regions of copy loss and gains.

### Length and amplitude thresholds of SCNAs

Length of each SCNA was converted into chromosome-arm unit by calculating the fraction of each chromosome arm covered by SCNA [14]; for the SCNAs that cross the centromere, the length is expressed as the sum of the fractions of each chromosome arm covered by the SCNA. This normalization results in value ranging between 0 to 2. SCNA with lengths < 0.2 (< 20% of the chromosome arm) were considered as focal SCNA, while SCNA covering more than 45% of the chromosome arm (lengths > 0.45) were considered as genomic aberrations.

### Statistical analysis

Survival curves were plotted according to the Kaplan-Meier method, using cBioportal database [60, 61] (http://www.cbioportal.org/). Nonparametric Spearman correlation was used to evaluate the association between the number of mutations or SCNAs and continuous variables (age and mitotic rate). The Mann-Whitney U test was used to assess the association between the number of mutations or SCNAs and dichotomous variables (gender, BRAFV600E, BRAF mutational status and metastasis evolution). Kruskal-Wallis test was used to investigate the association between the number of mutations or SCNAs and variables with three or more categories (location and tumor type). mRNA levels were investigated for class-specific expression using *t*-test and visualized by boxplots.

## SUPPLEMENTARY MATERIAL FIGURES AND TABLES




