# Supplementary material for: Thin and thick primary cutaneous melanomas reveal distinct patterns of somatic copy number alterations
Source: Oncotarget. 2016 Apr 15;7(21):30365–78. doi: 10.18632/oncotarget.8758 (PMC5058686; doi:10.18632/oncotarget.8758)
Supplement: Supplementary file 1 [file oncotarget-07-30365-s001.pdf]

Thin and thick primary cutaneous melanomas reveal distinct patterns of somatic copy number alterations

Supplementary Material

SCNA > 0.45 Chromosome Arm

M2

Chr	% Alteration/Arm	Gain/Loss
6p	47%	Gain
6q	86%	Loss
7p	53%	Gain
9p	69%	Loss
11p	87%	Gain
18q	55%	Gain
**8q**	**complete (98%)**	Gain

M11

Chr	% Alteration/Arm	Gain/Loss
6p	47%	High Gain
11p	63%	Gain

M9

Chr	% Alteration/Arm	Gain/Loss
2q	68%	Loss
3q	52%	Loss
6p	47%	High Gain
6q	86%	Loss
8q	80%	Gain
**8p**	**complete (96%)**	Gain
**19q**	**complete (95%)**	Loss

M14

Chr	% Alteration/Arm	Gain/Loss
4q	55%	Gain
6p	70%	High Gain
7p	61%	Gain
8p	48%	Gain
**8p+q**	p half (50%)	Gain
	**q complete (99%)**	Gain
9p	49%	Loss
10q	75%	Loss
**10p**	**p complete (99%)**	Loss
11p	76%	Gain
13q	q complete (96%)	Gain
14q	45%	Loss
**18p+q**	**p complete (99%)**	Gain
	**q complete (96%)**	Gain
20p	70%	Gain

Common aberrations

Chr	% Alteration/Arm	Gain/Loss	Samples
6p	47%	Gain	M2, M11 M9, M14
6q	at least 85%	Loss	M2, M9
7p	at least 53%	Gain	M2, M14
8p	at least 48%	Gain	M9, M11
9p	at least 50%	Loss	M2, M14
11p	at least 76%	Gain	M2, M11, M14
18q	at lest 50%	Gain	M2, M11

Supplementary Figure 1. Chromosome aberrations in four thick melanomas.

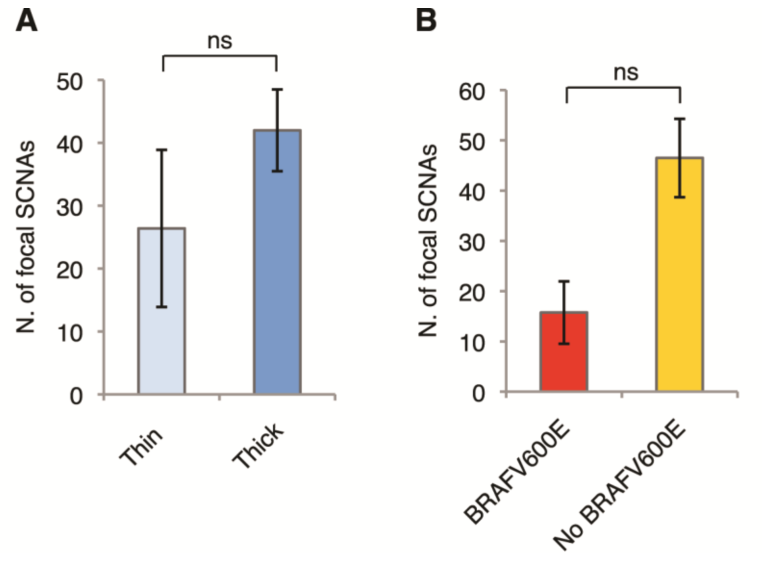

Supplementary Figure 2. (**A**) Number of focal SCNAs in thin (n=5) and thick melanomas (n=5) (p=0.27, Mann-Whitney U test). (**B**) Number of focal SCNAs in melanomas with BRAFV600E (n=4) and with no BRAFV600E mutation (n=6) (p=0.08, Mann-Whitney U test).

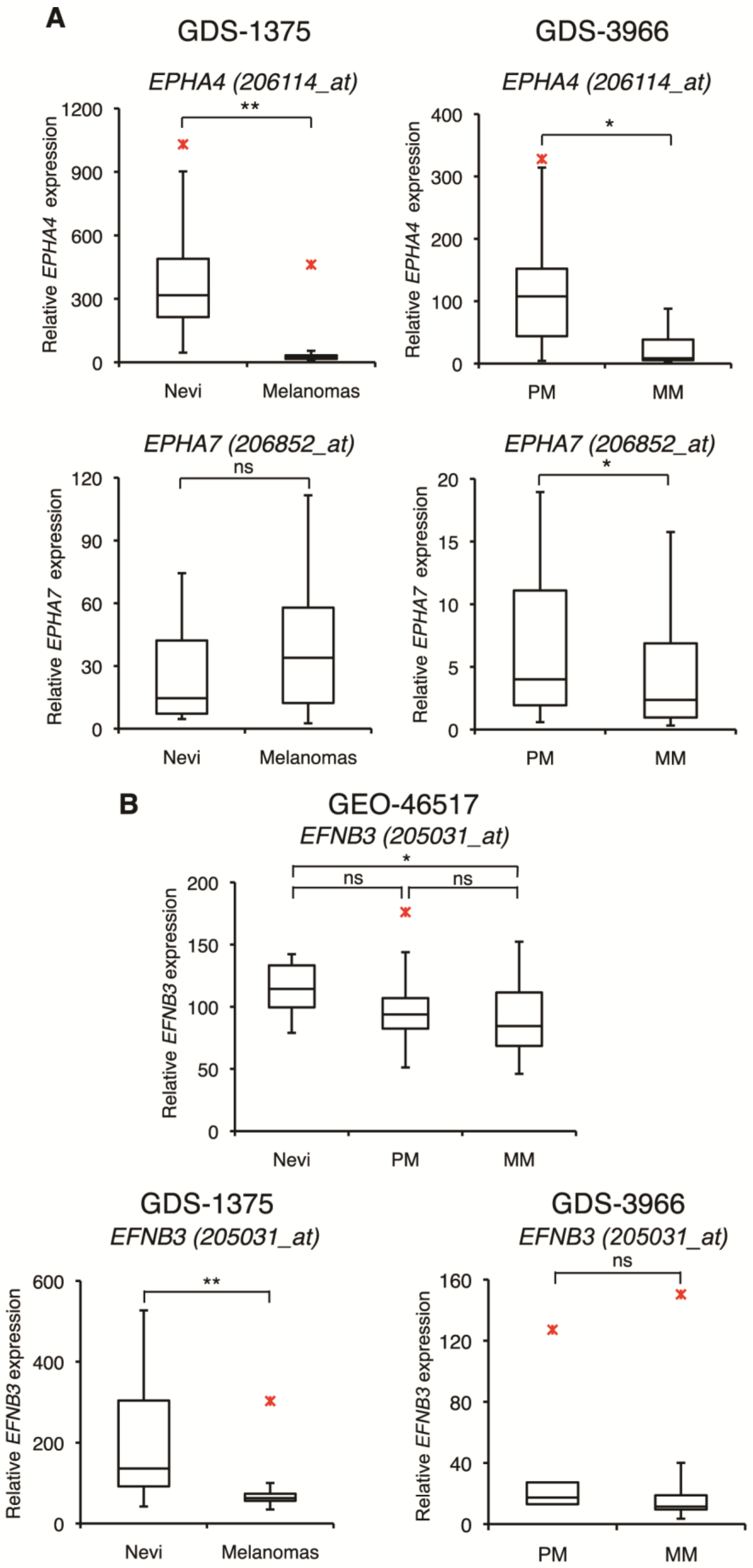
A
GDS-1375
EPHA4 (206114_at)
Relative EPHA4 expression
1200
900
600
300
0
**
Nevi
Melanomas
GDS-3966
EPHA4 (206114_at)
Relative EPHA4 expression
400
300
200
100
0
*
PM
MM
EPHA7 (206852_at)
Relative EPHA7 expression
120
90
60
30
0
ns
Nevi
Melanomas
EPHA7 (206852_at)
Relative EPHA7 expression
20
15
10
5
0
*
PM
MM
B
GEO-46517
EFNB3 (205031_at)
Relative EFNB3 expression
200
150
100
50
0
*
ns
ns
Nevi
PM
MM
GDS-1375
EFNB3 (205031_at)
Relative EFNB3 expression
600
400
200
0
**
Nevi
Melanomas
GDS-3966
EFNB3 (205031_at)
Relative EFNB3 expression
160
120
80
40
0
ns
PM
MM

Supplementary Figure 3. (**A**) Expression of *EPHA4* and *EPHA7* mRNA determined by different publicly available microarray data sets (GDS-1375 and GDS-3966). (**B**) Expression of *EFNB3* mRNA determined by different publicly available microarray data sets (GEO-46517, GDS-1375 and GDS-3966). In GEO-46517 nevi (n=9), primary (n=31) and metastatic (n=73) melanomas. In GDS-1375 nevi (n=17) and melanomas (n=45). In GDS-3966 primary melanomas (n=31) and metastatic melanomas (n=52). PM, primary melanomas; MM, metastatic melanomas. Red crosses indicate outliner values. *, p<0.05; **, p<0.001.

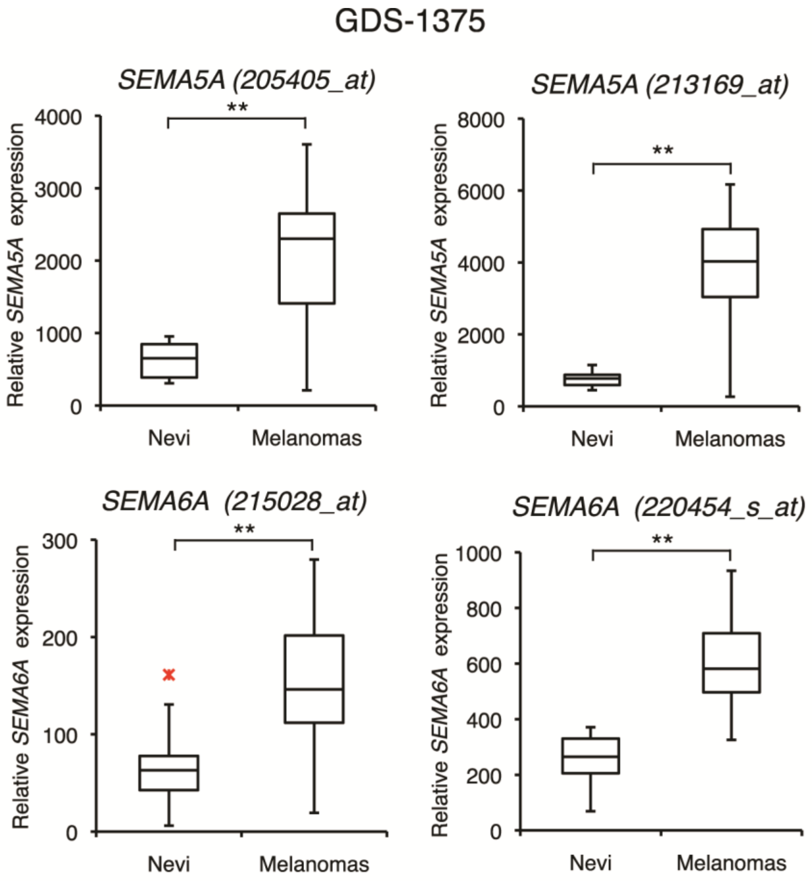

Supplementary Figure 4. Expression of *SEMA5A* and *SEMA6A* mRNA in nevi (n=17) and melanomas (n=45) determined by a publicly available microarray data set (GDS-1375). Red crosses indicate outliner values. **, p<0.001.

**Table S1:**	Summary of genomic data
**Table S2:**	Summary of somatic mutations
**Table S3:**	Non-synonymous and stopgain SNVs
**Table S4:**	Small indels
**Table S5:**	Splice variants
**Table S6:**	SCNA gain
**Table S7:**	SCNA loss
**Table S8:**	Mutations found in TCGA and Cosmic databases
