# Supplementary material for: Thin and thick primary cutaneous melanomas reveal distinct patterns of somatic copy number alterations
Source: Oncotarget. 2016 Apr 15;7(21):30365–78. doi: 10.18632/oncotarget.8758 (PMC5058686; doi:10.18632/oncotarget.8758)
Supplement: Supplementary file 2 [file oncotarget-07-30365-s002.pdf]

**Somatic SNVs, splice site variants and InDels in 10 primary melanomas**		
**Table S1:**	Summary of genomic data	
**Table S2:**	Summary of somatic mutations	
**Table S3:**	Non-synonymous and stopgain SNVs	
**Table S4:**	Small indels	
**Table S5:**	Splice variants	
**Table S6:**	SCNA gain	
**Table S7:**	SCNA loss	
**Table S8:**	Mutations found in TCGA and Cosmic databases	
Nomenclature	SNV	Single Nucleotide Variant
	Non-Syn	non synonymous
	InDel	Small Insertions and Deletions
	SIFT	Sorting Intolerant from Tolerant-http://sift.bii.a-star.edu.sg/
	PolyPhen2	Polymorphism Phenotyping v2-http://genetics.bwh.harvard.edu/pph2/
	GERP++	Genomic Evolutionary Rate Profiling

**Supplementary Table S1**										
**Summary of genomic data**										
**Sample**	**M1**	**M2**	**M4**	**M6**	**M8**	**M9**	**M11**	**M14**	**M16**	**M17**
**Tumor Bases Sequenced**	4503469345	6195271195	5232754210	6261716882	5583555317	5351785545	5341091813	4560712004	4467582677	4569279857
**Normal Bases Sequenced**	2367399291	1921479243	1666103631	4748181895	2517910195	3227203795	3203787467	4749850637	2054593095	2363325389
**Tumor Depth (Mean)**	72.54	99.79	84.28	100.86	89.93	86.2	86.03	73.46	71.96	73.6
**Normal Depth (Mean)**	38.13	30.95	26.84	76.48	40.56	51.98	51.6	76.51	33.09	38.07
**Tumor Depth (Median)**	59	77	68	91	70	66	40	66	57	59
**Normal Depth (Median)**	30	24	22	71	32	40	66	69	26	30
**Tumor Bases Above 10X (%)**	92.7	94.3	93.7	94.1	94.3	93.7	93.8	92.2	92.7	93.2
**Normal Bases Above 10X (%)**	87.7	83.8	80.3	92.9	88.7	91.4	91.5	93.1	85.2	87.5

**Supplementary Table S2**		
**Summary of somatic mutations**		
**Category**	**Class**	**Total**
Base Substitutions & Indels	Total mutations	3,815
	Synonymous	*1,192*
	Missense	*2,150*
	Nonsense	*138*
	Splice Site	*58*
	In-Frame Indel	*72*
	Frame-shift indel	*205*
	Total Indels	277
	Insertions	*87*
	Deletions	*190*

Supplementary Table S3															
**Non Synonymous and Stop Gain SNVs**															
**Chr**	**Start**	**End**	**Gene symbol**	**Gene dscription**	**Accession**	**Reference Allele**	**Variant Allele**	**Variant classification**	**cDNA change**	**Protein change**	**Sample**	**SIFT**	**Polyphen2**	**GERP++**
1	111861974	111861974	CHIA	chitinase, acidic	NM_201653.3	T	C	nonsynonymous SNV	T1061C	F354S	M1	D	D	3.8
1	120057246	120057246	HSD3B1	hydroxy-delta-5-steroid dehyd	NM_000862	C	A	nonsynonymous SNV	C1100A	T367N	M1	T	B	-0.043
1	12368514	12368514	VPS13D	vacuolar protein sorting 13 ho	NM_015378.3	G	T	nonsynonymous SNV	G6466T	V2156F	M1	D	B	4.47
1	152188109	152188109	HRNR	hornerin	NM_001009931	T	A	nonsynonymous SNV	A5996T	Q1999L	M1	T	B	3.05
1	157668390	157668390	FCRL3	Fc receptor-like 3	NM_052939	T	C	nonsynonymous SNV	A82G	N28D	M1	T	B	-10.7
1	158298748	158298748	CD1B	CD1b molecule	NM_001764	G	A	nonsynonymous SNV	C943T	L315F	M1	T	B	-3.14
1	19413261	19413261	UBR4	ubiquitin protein ligase E3 co	NM_020765	T	A	nonsynonymous SNV	A14599T	M4867L	M1	T	B	0.893
1	205272648	205272648	NUAK2	myogenin (myogenic factor 4	NM_030952	G	A	nonsynonymous SNV	C1817T	S606F	M1	D	D	4.94
1	231471745	231471745	EXOC8	exocyst complex component	NM_175876	G	T	nonsynonymous SNV	C1747A	P583T	M1	T	D	5.54
1	247835908	247835908	OR13G1	olfactory receptor, family 13,	NM_001005487	T	A	nonsynonymous SNV	A436T	M146L	M1	D	B	-1.12
1	248059712	248059712	OR2W3	olfactory receptor, family 2, s	NM_001001957	T	C	nonsynonymous SNV	T824C	M275T	M1	T	B	5.29
1	26385076	26385076	TRIM63	tripartite motif containing 63,	NM_032588	C	A	nonsynonymous SNV	G636T	K212N	M1	D	B	4.58
1	33361178	33361178	TMEM54	transmembrane protein 54	NM_033504	G	A	nonsynonymous SNV	C437T	P146L	M1	D	D	5.32
1	89652071	89652071	GBP4	guanylate binding protein 4	NM_052941	T	C	nonsynonymous SNV	A1652G	E551G	M1	T	B	-6.34
10	3208567	3208567	PITRM1	pitrilysin metallopeptidase 1	NM_001242307.1	0	GCACGCTAGG	stopgain	271_272insCATTCCT	Q91_F92delinsPFLSI	M1			
10	48414365	48414365	GDF2	growth differentiation factor 2	NM_016204	C	T	nonsynonymous SNV	G503A	G168E	M1	T	D	5.73
11	18735639	18735639	IGSF22	immunoglobulin superfamily,	NM_173588	C	A	nonsynonymous SNV	G1855T	V619L	M1	T	D	0.836
11	499914	499914	RNH1	ribonuclease/angiogenin inhil	NM_002939.3	G	A	nonsynonymous SNV	C358T	L120F	M1	D	D	3.03
11	55322235	55322235	OR4C15	olfactory receptor, family 4, s	NM_001001920	G	A	nonsynonymous SNV	G453A	M151I	M1	T	B	5.12
11	55703068	55703068	OR5I1	olfactory receptor, family 5, s	NM_006637	G	A	nonsynonymous SNV	C809T	P270L	M1	T	B	2.24
11	56185689	56185689	OR5R1	olfactory receptor, family 5, s	NM_001004744	A	G	nonsynonymous SNV	T20C	I7T	M1	T	B	5.7
11	56230240	56230240	OR5M9	olfactory receptor, family 5, s	NM_001004743	A	T	nonsynonymous SNV	T638A	L213H	M1	D	D	4.39
11	56409051	56409051	OR5AP2	olfactory receptor, family 5, s	NM_001002925	G	A	nonsynonymous SNV	C865T	P289S	M1	D	P	4.02
11	64137784	64137784	RPS6KA4	ribosomal protein S6 kinase,	NM_003942.2	C	T	nonsynonymous SNV	C1885T	R629C	M1	T	P	4.3
11	70331722	70331722	SHANK2	SH3 and multiple ankyrin rep	NM_133266	C	A	nonsynonymous SNV	G2912T	S971I	M1	D	P	4.48
11	75511479	75511479	DGAT2	diacylglycerol O-acyltransfera	NM_032564.4	G	A	nonsynonymous SNV	G1093A	A365T	M1	T	P	3.47
11	828916	828916	CRACR2B	calcium release activated cha	NM_001286606.1	G	A	nonsynonymous SNV	G230A	R77Q	M1	T	B	2.2
12	109923849	109923849	UBE3B	ubiquitin protein ligase E3B	NM_130466.3	C	A	nonsynonymous SNV	C315A	S105R	M1	T	B	5.03
12	120941627	120941627	COQ5	coenzyme Q5, methyltransfer	NM_032314	G	T	nonsynonymous SNV	C944A	T315K	M1	D	D	5.76
12	15650259	15650259	PTPRO	protein tyrosine phosphatase	NM_030667.2	C	T	nonsynonymous SNV	C430T	P144S	M1	T	D	2.497
12	52818504	52818504	KRT75	keratin 75, type II	NM_004693	T	G	nonsynonymous SNV	A1453C	S485R	M1	T	B	-3.45
12	52842736	52842736	KRT6B	keratin 6B, type II	NM_005555	T	C	nonsynonymous SNV	A1093G	I365V	M1	T	B	1.15
12	53586319	53586319	ITGB7	integrin, beta 7	NM_000889	G	T	nonsynonymous SNV	C1950A	D650E	M1		P	3.39
12	6838439	6838439	COPS7A	COP9 signalosome subunit 7	NM_001164093.1	G	T	nonsynonymous SNV	G354T	E118D	M1	T	B	1.17
12	91502687	91502687	LUM	lumican	NM_002345	C	T	nonsynonymous SNV	G70A	D24N	M1	T	B	4.75
13	25671292	25671292	PABPC3	poly(A) binding protein, cytop	NM_030979	C	T	nonsynonymous SNV	C956T	T319I	M1	D	P	0.875
13	35735999	35735999	NBEA	neurobeachin	NM_015678	G	A	nonsynonymous SNV	G3974A	R1325Q	M1	T	D	5.04
14	103803160	103803160	EIF5	eukaryotic translation initiatio	NM_001969.4	G	T	stopgain	G301T	E101X	M1	T		4.7
14	39777676	39777676	CTAGE5	CTAGE family, member 5	NM_001247989.1	G	C	nonsynonymous SNV	G1093C	E365Q	M1		B	4.5
14	65239539	65239539	SPTB	spectrin, beta, erythrocytic	NM_001024858.2	C	T	stopgain	G5312A	W1771X	M1	T		5.21
15	42166500	42166500	SPTBN5	spectrin, beta, non-erythrocyt	NM_016642	C	T	nonsynonymous SNV	G4679A	R1560H	M1	T	B	-5.21
15	43820498	43820498	MAP1A	microtubule-associated prote	NM_002373	C	T	nonsynonymous SNV	C6827T	S2276F	M1	T	B	4.23
16	11272426	11272426	CLEC16A	C-type lectin domain family 1	NM_015226	A	C	nonsynonymous SNV	A3041C	D1014A	M1	T	D	4.87
16	28607232	28607232	SULT1A2	sulfotransferase family, cytos	NM_001054.3	A	G	nonsynonymous SNV	T20C	I7T	M1	T	B	-4.51
16	48201432	48201432	ABCC11	ATP-binding cassette, sub-fa	NM_032583.3	T	C	nonsynonymous SNV	A4031G	H1344R	M1	T	B	2.03
16	53653071	53653071	RPGRIP1L	RPGRIP1-like	NM_015272.3	G	T	nonsynonymous SNV	C3482A	S1161Y	M1		P	5.11
16	848784	848784	GNG13	guanine nucleotide binding p	NM_016541	A	T	nonsynonymous SNV	T139A	F47I	M1	D	D	5.07
17	26955355	26955355	KIAA0100	KIAA0100	NM_014680	T	C	nonsynonymous SNV	A4522G	T1508A	M1	T	B	-1.17
17	33288604	33288604	ZNF830	zinc finger protein 830	NM_052857	G	T	nonsynonymous SNV	G19T	A7S	M1	T	B	-5.03
17	48677002	48677002	CACNA1G	calcium channel, voltage-dep	NM_018896.4	C	T	nonsynonymous SNV	C3472T	H1158Y	M1	T	B	1.63
17	71386453	71386453	SDK2	sidekick cell adhesion molecu	NM_001144952	C	A	nonsynonymous SNV	G4165T	A1389S	M1	T	D	5.2
17	7366032	7366032	ZBTB4	zinc finger and BTB domain c	NM_020899.3	C	A	nonsynonymous SNV	G2269T	A757S	M1	T	B	-1.34
17	74765833	74765833	MFSD11	major facilitator superfamily d	NM_001242532.1	G	A	nonsynonymous SNV	G754A	E252K	M1	D	P	4.71
17	7578263	7578263	TP53	tumor protein p53	NM_000546.5	G	A	stopgain	C586T	R196X	M1	T		4.44
19	12541795	12541795	ZNF443	zinc finger protein 443	NM_005815	C	A	nonsynonymous SNV	G1191T	Q397H	M1	D	B	-2.74
19	12542653	12542653	ZNF443	zinc finger protein 443	NM_005815	T	G	nonsynonymous SNV	A333C	K111N	M1	T	B	-0.006
19	12542654	12542654	ZNF443	zinc finger protein 443	NM_005815	T	A	nonsynonymous SNV	A332T	K111I	M1	T	B	-1.34
19	13211811	13211811	LYL1	lymphoblastic leukemia assoc	NM_005583	C	A	nonsynonymous SNV	G175T	G59C	M1	D	P	3.53
19	15918802	15918802	OR10H1	olfactory receptor, family 10,	NM_013940	C	G	nonsynonymous SNV	G46C	G16R	M1	D	D	2.28
19	17040006	17040006	CPAMD8	C3 and PZP-like, alpha-2-ma	NM_015692	C	A	nonsynonymous SNV	G3031T	V1011L	M1	T	B	2.35
19	22846697	22846697	ZNF492	zinc finger protein 492	NM_020855	T	C	nonsynonymous SNV	T226C	C76R	M1	D	B	-0.852
19	42596244	42596244	POU2F2	POU class 2 homeobox 2	NM_001207025.2	C	A	nonsynonymous SNV	G1377T	L459F	M1	D	D	3.62
19	45716461	45716461	EXOC3L2	exocyst complex component	NM_138568	G	C	nonsynonymous SNV	C1096G	G247R	M1	T	B	2.12

19	50463982	50463982	SIGLEC11	sialic acid binding Ig-like lecti	NM_052884.2	T	G	nonsynonymous SNV	A287C	E96A	M1	T	B	-1.5
19	54745508	54745508	LILRA6,LILRB3	leukocyte immunoglobulin-like	NM_001081450.2	G	A	nonsynonymous SNV	C602T	T201I	M1	D	P	-4.75
19	56515242	56515242	NLRP5	NLR family, pyrin domain con	NM_153447	G	A	nonsynonymous SNV	G223A	E75K	M1	T	D	2.12
19	57089691	57089691	ZNF470	zinc finger protein 470	NM_001001668	C	T	nonsynonymous SNV	C1894T	H632Y	M1	T	B	3.92
19	9028373	9028373	MUC16	mucin 16, cell surface associ	NM_024690	G	A	nonsynonymous SNV	C36419T	T12140M	M1	D	D	2.63
2	113404557	113404557	SLC20A1	solute carrier family 20 (phos	NM_005415	C	A	nonsynonymous SNV	C152A	A51D	M1	D	D	5.28
2	113414759	113414759	SLC20A1	solute carrier family 20 (phos	NM_005415	T	A	nonsynonymous SNV	T719A	V240D	M1	T	P	3.52
2	132290275	132290275	CCDC74A	coiled-coil domain containing	NM_138770.2	T	A	nonsynonymous SNV	T797A	L266Q	M1	D	D	2.66
2	138738831	138738831	HNMT	histamine N-methyltransferas	NM_001024075	G	T	nonsynonymous SNV	G236T	C79F	M1	T	B	1.15
2	141283848	141283848	LRP1B	low density lipoprotein recept	NM_018557	G	A	stopgain	C7834T	Q2612X	M1	T		5.08
2	141526900	141526900	LRP1B	low density lipoprotein recept	NM_018557	A	T	nonsynonymous SNV	T5640A	F1880L	M1	T	D	1.93
2	233199157	233199157	DIS3L2	DIS3 like 3'-5' exoribonucleas	NM_152383	C	T	nonsynonymous SNV	C2237T	S746F	M1	D	D	3.93
20	3624876	3624876	ATRN	attractin	NM_139321	C	T	nonsynonymous SNV	C4126T	P1376S	M1	D	P	5.26
20	41076951	41076951	PTPRT	protein tyrosine phosphatase	NM_133170.3	A	G	nonsynonymous SNV	T1469C	L490P	M1	T	P	5.88
20	744308	744308	SLC52A3	solute carrier family 52 (ribofl	NM_033409	T	C	nonsynonymous SNV	A907G	I303V	M1	D	D	4.95
22	24560375	24560375	CABIN1	calcineurin binding protein 1	NM_001199281.1	G	A	stopgain	G4754A	W1585X	M1	T		4.65
22	37692041	37692041	CYTH4	cytohesin 4	NM_013385	C	T	nonsynonymous SNV	C169T	R57W	M1	D	D	4.01
22	39078990	39078990	TOMM22	translocase of outer mitochor	NM_020243	C	A	nonsynonymous SNV	C343A	Q115K	M1	T	P	5.36
22	39497404	39497404	APOBEC3H	apolipoprotein B mRNA editir	NM_001166003.2	G	C	nonsynonymous SNV	G313C	G105R	M1	T	B	-4.63
3	111639187	111639187	PHLDB2	pleckstrin homology-like dom	NM_001134438.1	T	C	nonsynonymous SNV	T1922C	M641T	M1	T	B	5.13
3	125648356	125648356	ALG1L	ALG1, chitobiosyldiphosphod	NM_001195223.1	T	C	nonsynonymous SNV	A463G	N155D	M1	T	B	2.3
3	128623338	128623338	ACAD9	acyl-CoA dehydrogenase fam	NM_014049	C	A	nonsynonymous SNV	C1139A	A380D	M1	D	D	5.7
3	167742718	167742718	GOLIM4	golgi integral membrane prote	NM_014498	C	A	nonsynonymous SNV	G1789T	V597L	M1	T	P	5.02
3	187446196	187446196	BCL6	B-cell CLL/lymphoma 6	NM_001706.4	G	A	nonsynonymous SNV	C1492T	A204D	M1	T	B	4.1
3	195452018	195452018	MUC20	mucin 20, cell surface associ	NM_001282506	A	G	nonsynonymous SNV	A544G	S182G	M1	T	P	-1.67
3	46490500	46490500	LTF	lactotransferrin	NM_002343.4	C	T	nonsynonymous SNV	G1066A	E356K	M1	T	B	1.46
3	52823719	52823719	ITIH1	inter-alpha-trypsin inhibitor he	NM_002215.3	C	A	nonsynonymous SNV	C2170A	Q724K	M1	T	B	-1.36
4	39450229	39450229	KLB	Klotho beta	NM_175737	C	A	nonsynonymous SNV	C3058A	Q1020K	M1	T	B	2.81
4	55972974	55972974	KDR	Kinase insert domain recepto	NM_002253	T	A	nonsynonymous SNV	A1416T	Q472H	M1	T	P	-1.82
4	57797375	57797375	REST	RE1-silencing transcription fa	NM_005612	C	T	nonsynonymous SNV	C2351T	P784L	M1	T	B	0.413
4	677003	677003	MFSD7	Major facilitator superfamily d	NM_032219	G	T	nonsynonymous SNV	C1147A	L383M	M1	T	D	1.45
5	127647634	127647634	FBN2	Fibrillin 2	NM_001999	T	A	nonsynonymous SNV	A4891T	T1631S	M1	T	P	6.07
5	141244356	141244356	PCDH1	Protocadherin 1	NM_032420	C	T	nonsynonymous SNV	G1540A	A514T	M1	T	B	3.87
5	16680198	16680198	MYO10	Myosin X	NM_012334	G	T	nonsynonymous SNV	C4400A	T1467N	M1	D	P	5.46
5	75954287	75954287	IQGAP2	Q motif containing GTPase a	NM_006633	G	T	nonsynonymous SNV	G2324T	G775V	M1	T	B	3.9
5	76332468	76332468	AGGF1	Angiogenic factor with G patc	NM_018046	G	T	nonsynonymous SNV	G604T	V202L	M1		B	5.09
5	96098057	96098057	CAST	Calpastatin	NM_001042440	C	G	nonsynonymous SNV	C1790G	A597G	M1	T	B	0.31
6	100868779	100868779	SIM1	Single-minded family bHLH tr	NM_005068	G	T	nonsynonymous SNV	C1054A	P352T	M1	T	B	5.8
6	127898617	127898617	C6orf58	Chromosome 6 open reading	NM_001010905	G	T	nonsynonymous SNV	G287T	W96L	M1	D	D	5.24
6	152668349	152668349	SYNE1	Spectrin repeat containing, nu	NM_182961	C	A	nonsynonymous SNV	G11923T	G3975C	M1	T	P	3.11
6	158342607	158342607	SNX9	Sorting nexin 9	NM_016224	C	T	stopgain	C994T	Q332X	M1			
6	168351906	168351906	MLLT4	myeloid/lymphoid or mixed-lin	NM_001291964	C	A	nonsynonymous SNV	C3728A	A1243D	M1	T	B	1.48
6	32809352	32809352	PSMB8	Proteasome (prosome, macro	NM_004159	G	T	nonsynonymous SNV	C686A	A229D	M1	D	P	-0.706
6	3290037	3290037	SLC22A23	solute carrier family 22, mem	NM_015482	C	T	nonsynonymous SNV	G1274A	R425Q	M1	D	P	4.33
6	56482141	56482141	DST	Dystonin	NM_001723	C	A	nonsynonymous SNV	G6124T	A2042S	M1	T	B	-0.633
7	100680401	100680401	MUC17	Mucin 17, Cell Surface Assoc	NM_001040105	G	T	nonsynonymous SNV	G5704T	A1902S	M1	T	B	-2.38
7	107669572	107669572	LAMB4	Laminin, Beta 4	NM_007356	C	T	nonsynonymous SNV	G5062A	E1688K	M1	T	B	2.71
7	116869894	116869894	ST7	Suppression Of Tumorigenici	NM_018412	C	A	nonsynonymous SNV	C1648A	Q550K	M1	T	D	5.4
7	137150753	137150753	DGKI	Diacylglycerol Kinase, Iota	NM_004717	G	A	nonsynonymous SNV	C2537T	S846F	M1	D	P	5.82
7	142630534	142630534	TRPV5	Transient Receptor Potential	NM_019841	G	A	nonsynonymous SNV	C23T	A8V	M1	T	B	-1.91
7	79842048	79842048	GNAI1	Guanine nucleotide binding p	NM_002069	G	T	nonsynonymous SNV	G737T	S246I	M1		D	6.17
8	101054142	101054142	RGS22	regulator of G-protein signalir	NM_015668	G	A	nonsynonymous SNV	C1826T	S609L	M1			
8	106814615	106814615	ZFPM2	Zinc finger protein, FOG fami	NM_012082	C	T	nonsynonymous SNV	C2305T	P769S	M1	T	B	4.83
8	145998769	145998769	ZNF34	Zinc finger protein 34	NM_030580	C	A	nonsynonymous SNV	G1565T	R522L	M1	T	B	-1.62
8	146107670	146107670	ZNF250	zinc finger protein 250	NM_021061	G	A	nonsynonymous SNV	C913T	H305Y	M1	D	D	3.94
9	137620555	137620555	COL5A1	Collagen, type V, alpha 1	NM_000093	G	A	nonsynonymous SNV	G826A	E276K	M1	T	D	4.24
9	139302388	139302388	SDCCAG3	Serologically defined colon ca	NM_001039707	C	A	nonsynonymous SNV	G292T	D98Y	M1	D	D	5.34
9	79324872	79324872	PRUNE2	Prune homolog 2	NM_015225	C	A	nonsynonymous SNV	G2318T	R773L	M1	D	B	-3.06
X	102842229	102842229	TCEAL4	Transcription elongation facto	NM_001300901	T	C	nonsynonymous SNV	T1055C	I352T	M1	T	B	-0.255
X	151092653	151092653	MAGEA4	Melanoma antigen family A4	NM_001011550	G	A	nonsynonymous SNV	G517A	A173T	M1			-0.42
X	30326904	30326904	NR0B1	Nuclear receptor subfamily 0,	NM_000475	T	A	nonsynonymous SNV	A577T	T193S	M1	T	B	-9.23
X	3228309	3228309	MXRA5	Matrix-remodelling associated	NM_015419	G	T	nonsynonymous SNV	C7935A	N2645K	M1	T	P	-0.242
X	49355893	49355893	GAGE2A	G antigen 2A	NM_001127212	C	G	nonsynonymous SNV	C175G	Q59E	M1	T	B	-1.69
X	9707575	9707575	GPR143	G protein-coupled receptor 14	NM_000273	G	T	nonsynonymous SNV	C1070A	S357Y	M1	D	P	-8.94
1	248801602	248801602	OR2T35	Olfattory Receptor Family 2, S	NM_001001827	0	CA	stopgain	957_958insTG	I320_R321delinsX	M1			
13	46170839	46170839	ERICH6B	Glutamate-Rich 6B	NM_182542	G	T	nonsynonymous SNV	C302A	A101E	M1	D	B	-6.51
2	113588140	113588140	IL1B	Inteleukin 1, Beta	NM_000576	G	T	nonsynonymous SNV	C608A	P203H	M1	T	D	3.63

3	195452870	195452870	MUC20	Mucin20	NM_001282506	G	A	nonsynonymous SNV	G1396A	E466K	M1	T	B	2.47
2	179449692	179449692	TTN	Titin	NM_001267550	G	C	nonsynonymous SNV	C64676G	A21559G	M1	D	B	5.3
15	89398295	89398295	ACAN	aggrecan	NM_013227	A	T	stopgain	A2479T	K827X	M1	T		-3.62
16	848780	848780	GNG13	guanine nucleotide binding pr	NM_016541	A	T	nonsynonymous SNV	T143A	L48Q	M1	D	D	5.07
2	113588132	113588132	IL1B	Inteleukin 1, Beta	NM_000576	A	T	nonsynonymous SNV	T616A	Y206N	M1	T	D	2.95
3	195452872	195452872	MUC20	Mucin20	NM_001282506	A	C	nonsynonymous SNV	A1398C	E466D	M1	T	B	-3.24
3	195452873	195452873	MUC20	Mucin20	NM_001282506	G	A	nonsynonymous SNV	G1399A	A467T	M1	T	P	2.42
1	145296373	145296373	NBPF10	Neuroblastoma Breakpoint Fa	M_001039703	G	T	nonsynonymous SNV	G295T	V99F	M1	D		-158
1	1018290	1018290	C1orf159	chromosome 1 open reading	NM_017891	G	A	nonsynonymous SNV	C580T	R194C	M11	D	D	3.29
1	10239498	10239498	UBE4B	ubiquitination factor E4B	NM_001105562.2	C	A	nonsynonymous SNV	C3725A	T1242K	M11	T	P	5.78
1	111060917	111060917	KCNA10	potassium channel, voltage g	NM_005549	C	T	nonsynonymous SNV	G493A	E165K	M11	D	D	5.93
1	11893615	11893615	CLCN6	chloride channel, voltage-sen	NM_001286.3	C	T	nonsynonymous SNV	C1292T	P431L	M11	D	B	5.78
1	13183631	13183631	HNRNPCL2	heterogeneous nuclear ribon	NM_001136561	A	T	nonsynonymous SNV	T242A	V81D	M11			
1	145561870	145561870	ANKRD35	ankyrin repeat domain 35	NM_144698.4	G	A	nonsynonymous SNV	G1558A	G520R	M11	T	B	4.31
1	149901053	149901053	MTMR11	myotubularin related protein 1	NM_001145862.1	G	A	nonsynonymous SNV	C2098T	L700F	M11	T	B	0.6
1	150676620	150676620	HORMAD1	HORMA domain containing 1	NM_032132.4	G	A	nonsynonymous SNV	C922T	P308S	M11	T	B	3.66
1	152129137	152129137	RPTN	repetin	NM_001122965	A	T	nonsynonymous SNV	T438A	H146Q	M11	T	B	-3.44
1	152284648	152284648	FLG	filaggrin	NM_002016	C	T	nonsynonymous SNV	G2714A	G905D	M11	T	P	0.822
1	158990263	158990263	IFI16	interferon, gamma-inducible p	NM_001206567.1	C	T	nonsynonymous SNV	C937T	L313F	M11	D	D	-0.713
1	159410507	159410507	OR10J1	olfactory receptor, family 10, s	NM_012351	C	T	nonsynonymous SNV	C959T	S320F	M11	D	D	0.596
1	159558349	159558349	APCS	amyloid P component, serum	NM_001639	G	A	nonsynonymous SNV	G523A	D175N	M11	T	D	3.33
1	161137789	161137789	PPOX	protoporphyrinogen oxidase	NM_000309.3	C	A	nonsynonymous SNV	C343A	L115I	M11	T	B	4.63
1	17275337	17275337	CROCC	ciliary rootlet coiled-coil, rootl	NM_014675	C	T	nonsynonymous SNV	C2752T	R918W	M11	D	D	2.36
1	202861672	202861672	KLHL12	kelch-like family member 12	NM_021633	G	A	nonsynonymous SNV	C1696T	R566C	M11	D	P	5.27
1	215955491	215955491	USH2A	Usher syndrome 2A (autoson	NM_206933	G	A	nonsynonymous SNV	C10633T	R3545W	M11	D	P	3.62
1	223986173	223986173	TP53BP2	tumor protein p53 binding pro	NM_001031685	G	T	nonsynonymous SNV	C1692A	S564R	M11	T	B	-5.07
1	240371146	240371146	FMN2	formin 2	NM_020066	C	T	nonsynonymous SNV	C3034T	P1012S	M11		P	2.53
1	24385467	24385467	MYOM3	myomesin 3	NM_152372	G	A	nonsynonymous SNV	C3997T	R1333C	M11	D	D	4.64
1	24426228	24426228	MYOM3	myomesin 3	NM_152372	G	A	nonsynonymous SNV	C598T	R200C	M11	T	B	4.81
1	248487736	248487736	OR2M7	olfactory receptor, family 2, su	NM_001004691	C	T	nonsynonymous SNV	G135A	M45I	M11	T	B	1.55
1	248551026	248551026	OR2T6	olfactory receptor, family 2, su	NM_001005471	G	A	nonsynonymous SNV	G117A	M39I	M11	T	B	-1.34
1	27648704	27648704	TMEM222	transmembrane protein 222	NM_032125	G	A	nonsynonymous SNV	G16A	G6R	M11	D	B	3.63
1	36068927	36068927	PSMB2	proteasome (prosome, macro	NM_002794.4	T	G	nonsynonymous SNV	A547C	I183L	M11	D	B	5.82
1	47767944	47767944	STIL	SCL/TAL1 interrupting locus	NM_001048166.1	G	A	nonsynonymous SNV	C227T	S76L	M11	T	B	3.12
1	48701511	48701511	SLC5A9	solute carrier family 5 (sodium	NM_001135181.1	C	T	nonsynonymous SNV	C1327T	R443C	M11	D	D	5.73
1	84878115	84878115	DNASE2B	deoxyribonuclease II beta	NM_021233.2	C	T	stopgain	C631T	Q211X	M11	T		4.51
1	86965508	86965508	CLCA1	chloride channel accessory 1	NM_001285	T	C	nonsynonymous SNV	T2525C	I842T	M11	D	D	5.59
1	94543278	94543278	ABCA4	ATP-binding cassette, sub-fa	NM_000350	G	A	nonsynonymous SNV	C1522T	R508C	M11	T	P	4.29
1	97770838	97770838	DPYD	dihydropyrimidine dehydroge	NM_000110	C	T	nonsynonymous SNV	G2276A	R759Q	M11	D	B	4.41
10	11505566	11505566	USP6NL	USP6 N-terminal like	NM_001080491.3	G	A	nonsynonymous SNV	C1412T	P471L	M11	D	B	5.74
10	25273722	25273722	ENKUR	enkurin, TRPC channel intera	NM_145010.3	T	G	nonsynonymous SNV	A707C	Q236P	M11	D	D	4.98
10	28228936	28228936	ARMC4	armadillo repeat containing 4	NM_001290020.1	C	T	nonsynonymous SNV	G1987A	E663K	M11	T	B	5.53
10	29840139	29840139	SVIL	supervillin	NM_021738.2	G	A	stopgain	C214T	R72X	M11			4.45
10	52569793	52569793	A1CF	APOBEC1 complementation	NM_001198819.1	G	T	nonsynonymous SNV	C1518A	F506L	M11	T	P	4
10	6553150	6553150	PRKCQ	protein kinase C, theta	NM_006257.4	C	T	nonsynonymous SNV	G125A	G42E	M11	T	D	5.33
10	76857491	76857491	DUSP13	dual specificity phosphatase	NM_001007273	G	A	nonsynonymous SNV	C431T	P144L	M11	D	D	5.9
10	7745400	7745400	ITIH2	inter-alpha-trypsin inhibitor he	NM_002216	G	A	nonsynonymous SNV	G3A	M1I	M11	D	D	5.66
10	96985083	96985083	ACSM6	acyl-CoA synthetase medium	NM_207321	G	A	nonsynonymous SNV	G1237A	E413K	M11	T	B	
11	123909219	123909219	OR10G7	olfactory receptor, family 10, s	NM_001004463	G	A	nonsynonymous SNV	C490T	H164Y	M11	D	P	1.34
11	124764204	124764204	ROBO4	roundabout guidance recepto	NM_019055.5	G	A	nonsynonymous SNV	C1211T	T404I	M11	T	D	4.93
11	22399062	22399062	SLC17A6	solute carrier family 17 (vesic	NM_020346	G	A	nonsynonymous SNV	G1525A	E509K	M11	T	B	5.85
11	5443735	5443735	OR51Q1	olfactory receptor, family 51, s	NM_001004757	A	C	nonsynonymous SNV	A305C	Q102P	M11	D	D	5
11	5462717	5462717	OR51I1	olfactory receptor, family 51, s	NM_001005288	G	A	stopgain	C28T	Q10X	M11	T		5.75
11	57243942	57243942	RTN4RL2	reticulon 4 receptor-like 2	NM_178570	C	A	nonsynonymous SNV	C821A	A274D	M11	T	B	4.36
11	63175679	63175679	SLC22A9	solute carrier family 22 (organ	NM_080866	C	T	nonsynonymous SNV	C1384T	P462S	M11	D	D	2.46
11	64120647	64120647	CCDC88B	coiled-coil domain containing	NM_032251	C	T	stopgain	C3622T	Q1208X	M11	T		4.14
11	6943099	6943099	OR2D3	olfactory receptor, family 2, su	NM_001004684	G	A	nonsynonymous SNV	G867A	M289I	M11	T	B	4.02
11	7064674	7064674	NLRP14	NLR family, pyrin domain con	NM_176822	G	A	nonsynonymous SNV	G1417A	E473K	M11	T	B	2.47
11	72409141	72409141	ARAP1	ArfGAP with RhoGAP domain	NM_001040118.2	G	A	nonsynonymous SNV	C2552T	P851L	M11	D	D	5.44
11	72409142	72409142	ARAP1	ArfGAP with RhoGAP domain	NM_001040118.2	G	A	nonsynonymous SNV	C2551T	P851S	M11	D	D	4.53
11	75298775	75298775	MAP6	microtubule-associated prote	NM_033063	A	T	nonsynonymous SNV	T1771A	S591T	M11	T	B	-0.357
11	76915147	76915147	MYO7A	myosin VIIA	NM_000260.3	C	T	nonsynonymous SNV	C5353T	P1785S	M11	D	D	4.84
11	829467	829467	CRACR2B	calcium release activated cha	NM_001286606.1	G	T	nonsynonymous SNV	G385T	D129Y	M11	D	P	4.15
11	94862169	94862169	ENDOD1	endonuclease domain contai	NM_015036	G	A	nonsynonymous SNV	G929A	S310N	M11	T	B	1.83
12	102155397	102155397	GNPTAB	N-acetylglucosamine-1-phos	NM_024312	G	A	nonsynonymous SNV	C2860T	P954S	M11	D	D	5.86
12	104102233	104102233	STAB2	stabilin 2	NM_017564	C	T	nonsynonymous SNV	C4207T	H1403Y	M11	T	P	5.4
12	119588962	119588962	SRRM4	serine/arginine repetitive mat	NM_194286	G	A	nonsynonymous SNV	G1217A	R406Q	M11		B	2.63

12	122398578	122398578	WDR66	WD repeat domain 66	NM_144668.5	C	T	nonsynonymous SNV	C2221T	R741C	M11	T	P	3.87
12	124229490	124229490	ATP6V0A2	ATPase, H+ transporting, lyso	NM_012463	C	T	stopgain	C1576T	R526X	M11	T		3.86
12	125455916	125455916	DHX37	DEAH (Asp-Glu-Ala-His) box	NM_032656	G	A	nonsynonymous SNV	C1123T	H375Y	M11	D	D	5.22
12	18891674	18891674	CAPZA3	capping protein (actin filamen	NM_033328	C	T	nonsynonymous SNV	C472T	H158Y	M11	D	P	3.95
12	21918865	21918865	KCNJ8	potassium channel, inwardly	NM_004982	C	T	nonsynonymous SNV	G1067A	R356Q	M11	D	B	5.55
12	27152555	27152555	TM7SF3	transmembrane 7 superfamily	NM_016551	G	A	nonsynonymous SNV	C301T	L101F	M11	D	D	3.49
12	48368002	48368002	COL2A1	collagen, type II, alpha 1	NM_001844.4	C	T	nonsynonymous SNV	G4187A	S1396N	M11	D	D	5.06
12	4848488	4848488	GALNT8	polypeptide N-acetylgalactosa	NM_017417	C	A	nonsynonymous SNV	C669A	S223R	M11	D	D	1.53
12	51773216	51773216	GALNT6	polypeptide N-acetylgalactosa	NM_007210	C	T	nonsynonymous SNV	G350A	G117E	M11	D	D	4.52
12	58114649	58114649	OS9	osteosarcoma amplified 9, en	NM_006812.3	C	T	nonsynonymous SNV	C1961T	P654L	M11	T	B	-10.6
12	9980190	9980190	KLRF1	killer cell lectin-like receptor s	NM_016523.2	G	A	nonsynonymous SNV	G50A	R17K	M11		P	-0.447
13	103389530	103389530	CCDC168	coiled-coil domain containing	NM_001146197	C	T	nonsynonymous SNV	G13517A	G4506E	M11			
13	110804750	110804750	COL4A1	collagen, type IV, alpha 1	NM_001845	C	T	nonsynonymous SNV	G4859A	G1620E	M11	D	D	5.51
13	111143607	111143607	COL4A2	collagen, type IV, alpha 2	NM_001846	G	A	nonsynonymous SNV	G3374A	G1125E	M11	D	D	5.04
13	114758024	114758024	RASA3	RAS p21 protein activator 3	NM_007368	G	A	nonsynonymous SNV	C2182T	R728C	M11	0	D	5.05
13	28877356	28877356	FLT1	fms-related tyrosine kinase 1	NM_002019	G	C	nonsynonymous SNV	C3965G	S1322C	M11	D	P	4.21
13	32355867	32355867	RXFP2	relaxin/insulin-like family pept	NM_130806.3	C	T	nonsynonymous SNV	C845T	S282L	M11	T	B	3.83
13	32885862	32885862	ZAR1L	zygote arrest 1-like	NM_001136571	C	A	nonsynonymous SNV	G201T	Q67H	M11	D	B	2.17
13	36229748	36229748	NBEA	neurobeachin	NM_015678	G	A	nonsynonymous SNV	G8161A	D2721N	M11	D	D	6.06
13	46287470	46287470	SPERT	spermatid associated	NM_152719.2	G	A	nonsynonymous SNV	G310A	E104K	M11	T	D	5.1
13	49281381	49281381	CYSLTR2	cysteinyl leukotriene receptor	NM_020377	C	T	nonsynonymous SNV	C428T	P143L	M11	D	D	5.89
14	105419084	105419084	AHNAK2	AHNAK nucleoprotein 2	NM_138420	G	A	nonsynonymous SNV	C2704T	P902S	M11	T	P	-6.96
14	19553526	19553526	POTEG	POTE ankyrin domain family,	NM_001005356	G	A	nonsynonymous SNV	G110A	G37E	M11	T	B	0
14	20770112	20770112	TTC5	tetratricopeptide repeat doma	NM_138376	G	A	stopgain	C64T	Q22X	M11			4.48
14	24537864	24537864	LRRC16B	leucine rich repeat containing	NM_138360	G	A	nonsynonymous SNV	G3754A	E1252K	M11	T	P	4.17
14	50558457	50558457	C14orf183	long intergenic non-protein co	NM_001014830	C	A	nonsynonymous SNV	G111T	Q37H	M11	D	D	2.72
14	51080016	51080016	ATL1	atlastin GTPase 1	NM_015915.4	G	A	nonsynonymous SNV	G670A	E224K	M11	T	P	5.41
14	58690508	58690508	ACTR10	actin-related protein 10 homo	NM_018477	C	G	nonsynonymous SNV	C722G	S241C	M11	T	P	3.9
14	81046750	81046750	CEP128	centrosomal protein 128kDa	NM_152446	G	A	stopgain	C2824T	Q942X	M11	T		5.73
14	91739484	91739484	CCDC88C	coiled-coil domain containing	NM_001080414	G	A	nonsynonymous SNV	C5572T	R1858W	M11	D	D	3.86
14	92268705	92268705	TC2N	tandem C2 domains, nuclear	NM_152332.5	C	T	nonsynonymous SNV	G362A	G121E	M11	T	B	4.47
14	94754765	94754765	SERPINA10	serpin peptidase inhibitor, cla	NM_016186.2	G	A	nonsynonymous SNV	C850T	R284C	M11	T	P	3.44
14	97022743	97022743	PAPOLA	poly(A) polymerase alpha	NM_032632.4	C	A	nonsynonymous SNV	C1997A	T666K	M11	T	B	4.3
15	62944246	62944246	TLN2	talin 2	NM_015059	C	T	nonsynonymous SNV	C277T	R93W	M11	D	B	5.92
15	65489182	65489182	CILP	cartilage intermediate layer p	NM_003613	C	T	nonsynonymous SNV	G3442A	G1148R	M11	T	B	3.97
15	73023725	73023725	BBS4	Bardet-Biedl syndrome 4	NM_033028.4	G	T	nonsynonymous SNV	G791T	C264F	M11	D	B	1.87
15	73616228	73616228	HCN4	hyperpolarization activated cy	NM_005477	A	T	nonsynonymous SNV	T2206A	Y736N	M11	T	P	3.46
15	79067065	79067065	ADAMTS7	ADAM metallopeptidase with	NM_014272	G	A	nonsynonymous SNV	C1777T	P593S	M11	T	P	3.51
15	98984374	98984374	FAM169B	family with sequence similarit	NM_182562	C	T	nonsynonymous SNV	G385A	E129K	M11	D	B	-0.446
16	14988889	14988889	NOMO1	NODAL modulator 1	NM_014287	C	T	nonsynonymous SNV	C3479T	S1160F	M11	D	D	2.96
16	20999357	20999357	DNAH3	dynein, axonemal, heavy cha	NM_017539	G	A	nonsynonymous SNV	C6632T	S2211F	M11	T	D	5.09
16	2104297	2104297	TSC2	tuberous sclerosis 2	NM_000548.3	G	A	nonsynonymous SNV	G337A	G113S	M11		D	4.02
16	2104298	2104298	TSC2	tuberous sclerosis 2	NM_000548.3	G	A	nonsynonymous SNV	G338A	G113D	M11	T	D	4.01
16	21063054	21063054	DNAH3	dynein, axonemal, heavy cha	NM_017539	C	T	nonsynonymous SNV	G4175A	G1392D	M11	D	D	5.78
16	29883590	29883590	SEZ6L2	seizure related 6 homolog (m	NM_001243332.1	G	A	nonsynonymous SNV	C2660T	S887F	M11	D	D	5.43
16	30545601	30545601	ZNF747	zinc finger protein 747	NM_023931	G	A	nonsynonymous SNV	C400T	P134S	M11	T	P	0.042
16	4414299	4414299	CORO7,CORO7	CORO7-PAM16 readthrough	NM_001201479.1	G	A	nonsynonymous SNV	C1253T	P418L	M11	T	B	3.78
16	53337880	53337880	CHD9	chromodomain helicase DNA	NM_025134	C	T	nonsynonymous SNV	C5962T	R1988C	M11	D	D	6.16
16	56974084	56974084	HERPUD1	homocysteine-inducible, endo	NM_014685.3	C	T	nonsynonymous SNV	C832T	L278F	M11	T	D	5.74
16	57994779	57994779	CNGB1	cyclic nucleotide gated chann	NM_001297.4	C	T	nonsynonymous SNV	G499A	E167K	M11	D	B	3.67
16	66919622	66919622	PDP2	pyruvate dehyrogenase phos	NM_020786	A	T	nonsynonymous SNV	A1435T	I479F	M11	D	P	3.4
16	67270563	67270563	FHOD1	formin homology 2 domain co	NM_013241	T	G	nonsynonymous SNV	A1216C	S406R	M11	T	B	2.91
16	71007323	71007323	HYDIN	HYDIN, axonemal central pai	NM_001270974	C	T	nonsynonymous SNV	G5302A	E1768K	M11	T	P	4.35
16	71008524	71008524	HYDIN	HYDIN, axonemal central pai	NM_001270974	C	T	nonsynonymous SNV	G4822A	E1608K	M11	T	B	3.69
16	71423754	71423754	CALB2	calbindin 2	NM_001740	G	A	nonsynonymous SNV	G802A	E268K	M11	T	B	5.35
16	75146500	75146500	LDHD	lactate dehydrogenase D	NM_153486.3	C	T	nonsynonymous SNV	G1358A	R453K	M11		P	5.51
17	10304066	10304066	MYH8	myosin, heavy chain 8, skelet	NM_002472	C	T	nonsynonymous SNV	G3376A	E1126K	M11	D	D	4.41
17	10346712	10346712	MYH4	myosin, heavy chain 4, skelet	NM_017533	T	A	stopgain	A5800T	K1934X	M11	T		5.19
17	15522641	15522641	CDRT1	CMT1A duplicated region tran	NM_006382.3	C	A	nonsynonymous SNV	G186T	K62N	M11	T	B	-0.782
17	1747907	1747907	RPA1	replication protein A1, 70kDa	NM_002945	G	A	nonsynonymous SNV	G199A	E67K	M11	T	P	5.02
17	18671865	18671865	FBXW10	F-box and WD repeat domain	NM_001267585.1	G	A	nonsynonymous SNV	G1723A	G575R	M11	D	D	2.97
17	28545887	28545887	SLC6A4	solute carrier family 6 (neurot	NM_001045	C	T	nonsynonymous SNV	G406A	E136K	M11	D	D	5.71
17	33767745	33767745	SLFN13	schlafen family member 13	NM_144682	G	A	nonsynonymous SNV	C2563T	R855W	M11	D	D	3.26
17	3665235	3665235	ITGAE	integrin, alpha E (antigen CD	NM_002208	C	T	nonsynonymous SNV	G289A	V97I	M11	D	B	1.5
17	39659206	39659206	KRT13	keratin 13, type I	NM_153490.2	C	T	nonsynonymous SNV	G880A	E294K	M11	T	B	3.35
17	46607875	46607875	HOXB1	homeobox B1	NM_002144	C	T	nonsynonymous SNV	G392A	G131E	M11	T	B	0.693
17	54428267	54428267	ANKFN1	ankyrin-repeat and fibronectin	NM_153228	C	T	nonsynonymous SNV	C338T	S113F	M11	T	D	5.84

17	59482001	59482001	TBX2	T-box 2	NM_005994	G	A	nonsynonymous SNV	G922A	E308K	M11	T	P	4.75
17	63198176	63198176	RGS9	regulator of G-protein signalir	NM_003835.3	G	A	nonsynonymous SNV	G1042A	E348K	M11	T	D	5.81
17	65040837	65040837	CACNG1	calcium channel, voltage-dep	NM_000727	G	C	nonsynonymous SNV	G61C	V21L	M11	T	B	4.42
17	72367925	72367925	GPR142	G protein-coupled receptor 1	NM_181790	C	T	nonsynonymous SNV	C575T	S192F	M11	D	D	4.02
17	72954801	72954801	HID1	HID1 domain containing	NM_030630	G	A	nonsynonymous SNV	C1207T	L403F	M11	D	D	4.82
17	73236917	73236917	GGA3	golgi-associated, gamma ada	NM_138619.3	G	T	nonsynonymous SNV	C1168A	L390M	M11	D	D	4.56
17	7324322	7324322	SPEM1	spermatid maturation 1	NM_199339	C	T	nonsynonymous SNV	C328T	H110Y	M11	D	B	2.04
17	76488802	76488802	DNAH17	dynein, axonemal, heavy cha	NM_173628	G	A	nonsynonymous SNV	C6454T	P2152S	M11	T		3.68
17	78402442	78402442	ENDOV	endonuclease V	NM_173627.4	C	T	nonsynonymous SNV	C761T	P254L	M11	T	B	0.781
17	9757818	9757818	GLP2R	glucagon-like peptide 2 recep	NM_004246	C	T	nonsynonymous SNV	C511T	R171C	M11	T	B	1.83
18	12009885	12009885	IMPA2	inositol(myo)-1(or 4)-monoph	NM_014214	C	A	nonsynonymous SNV	C234A	R171C	M11	D	D	5.48
18	42530690	42530690	SETBP1	SET binding protein 1	NM_015559	C	A	nonsynonymous SNV	C1385A	P462H	M11	T	B	5.2
18	42532021	42532021	SETBP1	SET binding protein 1	NM_015559	C	T	nonsynonymous SNV	C2716T	P906S	M11	T	B	4.34
18	44146307	44146307	LOXHD1	lipoxygenase homology doma	NM_144612	G	A	nonsynonymous SNV	C2350T	P784S	M11	T	P	4.95
18	55222081	55222081	FECH	ferrochelatase	NM_001012515.2	G	A	nonsynonymous SNV	C926T	S309F	M11	D	D	5.77
18	57103301	57103301	CCBE1	collagen and calcium binding	NM_133459	C	T	nonsynonymous SNV	G1060A	E354K	M11	D	B	5.8
18	61323113	61323113	SERPINB3	serpin peptidase inhibitor, cla	NM_006919	C	T	nonsynonymous SNV	G951A	M317I	M11	T	B	1.21
18	61325785	61325785	SERPINB3	serpin peptidase inhibitor, cla	NM_006919	C	T	nonsynonymous SNV	G431A	R144Q	M11	D	B	0.091
18	77726676	77726676	HSBP1L1	heat shock factor binding pro	NM_001136180	G	A	nonsynonymous SNV	G116A	R39K	M11	T	B	3.8
19	15572085	15572085	RASAL3	RAS protein activator like 3	NM_022904	C	T	nonsynonymous SNV	G488A	G163E	M11	D	P	1.5
19	15572086	15572086	RASAL3	RAS protein activator like 3	NM_022904	C	T	nonsynonymous SNV	G487A	G163R	M11	D	P	3.88
19	15806747	15806747	CYP4F12	cytochrome P450, family 4, s	NM_023944	G	A	nonsynonymous SNV	G1117A	D373N	M11	D	P	2.41
19	15905670	15905670	OR10H5	olfactory receptor, family 10,	NM_001004466	G	A	nonsynonymous SNV	G812A	G271E	M11	T	P	2.81
19	22156481	22156481	ZNF208	zinc finger protein 208	NM_007153	G	A	nonsynonymous SNV	C1355T	P452L	M11	T	D	0.432
19	22363024	22363024	ZNF676	zinc finger protein 676	NM_001001411	G	A	nonsynonymous SNV	C1495T	H499Y	M11	D	D	0.81
19	22941345	22941345	ZNF99	zinc finger protein 99	NM_001080409	C	T	nonsynonymous SNV	G1366A	E456K	M11	T	B	-2.55
19	29699033	29699033	UQCRFS1	ubiquinol-cytochrome c reduc	NM_006003	T	C	nonsynonymous SNV	A247G	I83V	M11	T	B	-4.98
19	35800953	35800953	MAG	myelin associated glycoprote	NM_002361.3	C	T	nonsynonymous SNV	C1408T	L470F	M11	T	B	2.64
19	37643575	37643575	ZNF585A	zinc finger protein 585A	NM_001288800.1	G	A	nonsynonymous SNV	C1226T	S409L	M11	D	B	0.708
19	40903175	40903175	PRX	periaxin	NM_181882	A	T	nonsynonymous SNV	T1084A	F362I	M11	T	D	4.56
19	42818481	42818481	TMEM145	transmembrane protein 145	NM_173633	C	T	nonsynonymous SNV	C178T	R60C	M11	D	B	3.43
19	44117527	44117527	SRRM5	serine/arginine repetitive mat	NM_001145641	T	A	nonsynonymous SNV	T1254A	S418R	M11	T	P	-7.61
19	4552503	4552503	SEMA6B	sema domain, transmembran	NM_032108	G	A	nonsynonymous SNV	C920T	A307V	M11	D	D	4.24
19	4552599	4552599	SEMA6B	sema domain, transmembran	NM_032108	G	A	nonsynonymous SNV	C824T	P275L	M11	D	B	4.18
19	45976262	45976262	FOSB	FBJ murine osteosarcoma vi	NM_006732.2	G	A	nonsynonymous SNV	G1009A	A337T	M11	T	D	4.89
19	49671565	49671565	TRPM4	transient receptor potential ca	NM_017636.3	T	C	nonsynonymous SNV	T497C	V166A	M11	D	D	4.75
19	49693499	49693499	TRPM4	transient receptor potential ca	NM_017636.3	C	T	nonsynonymous SNV	C2054T	A685V	M11	T	B	2.71
19	50212088	50212088	CPT1C	carnitine palmitoyltransferase	NM_001199752.2	C	T	nonsynonymous SNV	C1558T	P520S	M11	T	D	4.42
19	51628322	51628322	SIGLEC9	sialic acid binding Ig-like lecti	NM_001198558.1	G	A	nonsynonymous SNV	G91A	V31M	M11	D	D	2.88
19	52824895	52824895	ZNF480	zinc finger protein 480	NM_144684.3	C	T	nonsynonymous SNV	C392T	S131F	M11	D	P	-3.71
19	54080055	54080055	ZNF331	zinc finger protein 331	NM_018555.5	C	T	nonsynonymous SNV	C241T	R81C	M11	D	B	-0.85
19	55284824	55284824	KIR2DL1	killer cell immunoglobulin-like	NM_014218	G	C	nonsynonymous SNV	G110C	R37P	M11	T	B	1.24
19	55331197	55331197	KIR3DL1	killer cell immunoglobulin-like	NM_013289	C	T	nonsynonymous SNV	C385T	H129Y	M11	D	P	1.33
19	55450851	55450851	NLRP7	NLR family, pyrin domain con	NM_001127255.1	G	A	nonsynonymous SNV	C1336T	R446C	M11	T	B	-1.84
19	5867208	5867208	FUT5	fucosyltransferase 5 (alpha (	NM_002034	C	T	nonsynonymous SNV	G529A	D177N	M11	D	D	2.17
19	58806093	58806093	ZNF8	zinc finger protein 8	NM_021089	C	T	nonsynonymous SNV	C919T	H307Y	M11	D	D	4.82
19	8807909	8807909	ACTL9	actin-like 9	NM_178525	C	T	stopgain	G1143A	W381X	M11	T		4.51
19	9070708	9070708	MUC16	mucin 16, cell surface associ	NM_024690	G	A	nonsynonymous SNV	C16738T	L5580F	M11	D	B	-3.34
2	101648828	101648828	TBC1D8	TBC1 domain family, membe	NM_001102426	G	A	nonsynonymous SNV	C1793T	S598F	M11	D	D	5.02
2	120723203	120723203	PTPN4	protein tyrosine phosphatase	NM_002830	C	T	nonsynonymous SNV	C2540T	P847L	M11	D	D	5.62
2	128186322	128186322	PROC	protein C (inactivator of coag	NM_000312	G	A	nonsynonymous SNV	G1186A	D396N	M11	D	D	4.84
2	141816481	141816481	LRP1B	low density lipoprotein recept	NM_018557	C	T	nonsynonymous SNV	G1379A	R460Q	M11	T	B	4.57
2	160303331	160303331	BAZ2B	bromodomain adjacent to zin	NM_013450.3	G	A	stopgain	C658T	Q220X	M11	T		5.7
2	167128979	167128979	SCN9A	sodium channel, voltage gate	NM_002977	C	T	nonsynonymous SNV	G3248A	G1083E	M11	T	B	4.49
2	177034144	177034144	HOXD3	homeobox D3	NM_006898	G	A	nonsynonymous SNV	G302A	G101E	M11		B	4.59
2	179417157	179417157	TTN	titin	NM_001267550	C	T	nonsynonymous SNV	G90470A	G30157E	M11	D	D	5.76
2	179614277	179614277	TTN	titin	NM_133379	C	A	nonsynonymous SNV	G12850T	A4284S	M11	T	B	3.23
2	179616439	179616439	TTN	titin	NM_133379	G	A	nonsynonymous SNV	C10688T	S3563F	M11	T	P	4.87
2	179644864	179644864	TTN	titin	NM_001267550	G	A	stopgain	C3592T	Q1198X	M11	D		4.9
2	203653609	203653609	ICA1L	islet cell autoantigen 1,69kDa	NM_138468.5	C	T	nonsynonymous SNV	G1187A	R396Q	M11	T	B	-1.24
2	209025590	209025590	CRYGA	crystallin, gamma A	NM_014617	G	A	nonsynonymous SNV	C463T	H155Y	M11	T	B	3.73
2	21225126	21225126	APOB	apolipoprotein B	NM_000384	C	T	nonsynonymous SNV	G13168A	D4390N	M11	D	P	5.02
2	21230594	21230594	APOB	apolipoprotein B	NM_000384	G	A	nonsynonymous SNV	C9146T	R3638Q	M11	D	P	4.92
2	219449406	219449406	RQCD1	RCD1 required for cell differe	NM_001271634.1	C	T	nonsynonymous SNV	C392T	P131L	M11	D	D	5.22
2	230377642	230377642	DNER	delta/notch-like EGF repeat c	NM_139072	G	A	nonsynonymous SNV	C1004T	S335F	M11	T	B	5.54
2	27278681	27278681	AGBL5	ATP/GTP binding protein-like	NM_021831.5	C	T	nonsynonymous SNV	C1040T	S347F	M11	T	P	5.36
2	48047595	48047595	FBXO11	F-box protein 11	NM_025133.4	C	A	nonsynonymous SNV	G1451T	G484V	M11	D	P	5.35

2	49190891	49190891	FSHR	follicle stimulating hormone re	NM_000145.3	C	T	nonsynonymous SNV	G1069A	E357K	M11		D	5.13
2	65299677	65299677	CEP68	centrosomal protein 68kDa	NM_015147	C	T	nonsynonymous SNV	C1447T	L483F	M11	T	D	4.9
2	96781254	96781254	ADRA2B	adrenoceptor alpha 2B	NM_000682	C	A	nonsynonymous SNV	G635T	G212V	M11	D	B	0.85
20	210261	210261	DEFB129	defensin, beta 129	NM_080831	C	T	nonsynonymous SNV	C401T	T134I	M11	T	B	-7.35
20	36668956	36668956	RPRD1B	egulation of nuclear pre-mRN	NM_021215	C	T	nonsynonymous SNV	C271T	H91Y	M11	T	D	5.54
20	40033738	40033738	CHD6	chromodomain helicase DNA	NM_032221	G	A	nonsynonymous SNV	C7643T	S2548F	M11	D	B	4.6
20	43615812	43615812	STK4	serine/threonine kinase 4	NM_006282	C	T	nonsynonymous SNV	C400T	L134F	M11	D	D	5.39
20	44678350	44678350	SLC12A5	solute carrier family 12 (potas	NM_001134771.1	G	A	nonsynonymous SNV	G2171A	G724E	M11	D	D	4.43
20	8609000	8609000	PLCB1	phospholipase C, beta 1 (pho	NM_015192.3	G	A	nonsynonymous SNV	G306A	M102I	M11	T	D	6.17
21	10934114	10934114	TPTE	transmembrane phosphatase	NM_199261.3	C	T	nonsynonymous SNV	G863A	R288K	M11	T	B	1.17
21	27078356	27078356	JAM2	junctional adhesion molecule	NM_021219.3	G	A	nonsynonymous SNV	G763A	G255S	M11	T	D	5.02
21	27078357	27078357	JAM2	junctional adhesion molecule	NM_021219.3	G	A	nonsynonymous SNV	G764A	G255D	M11	T	D	5.02
21	43277341	43277341	PRDM15	PR domain containing 15	NM_022115.4	G	A	nonsynonymous SNV	C1327T	P443S	M11	D	D	5.58
21	45815369	45815369	TRPM2	transient receptor potential ca	NM_003307	C	T	nonsynonymous SNV	C1867T	R623C	M11	D	D	4.32
21	46021150	46021150	KRTAP10-7	keratin associated protein 10-	NM_198689	C	A	nonsynonymous SNV	C614A	T205K	M11	T	B	0.846
22	19511859	19511859	CLDN5	claudin 5	NM_001130861.1	C	T	nonsynonymous SNV	G175A	A59T	M11	D	B	1.19
22	19707925	19707925	SEPT5	septin 5	NM_002688.5	C	T	stopgain	C445T	Q149X	M11	T		1.6
22	39714546	39714546	RPL3	ribosomal protein L3	NM_000967.3	G	A	nonsynonymous SNV	C55T	R19W	M11	D	P	4.17
3	107435678	107435678	BBX	bobby sox homolog (Drosoph	NM_001142568.2	C	A	stopgain	C387A	Y129X	M11	T		0.585
3	114070014	114070014	ZBTB20	zinc finger and BTB domain c	NM_001164342.2	G	A	nonsynonymous SNV	C911T	T304I	M11	D	B	5.52
3	121126200	121126200	STXBP5L	syntaxin binding protein 5-like	NM_014980	G	A	nonsynonymous SNV	G2770A	E924K	M11	T	B	4.69
3	130699468	130699468	ATP2C1	ATPase, Ca++ transporting, t	NM_001199180.1	C	T	nonsynonymous SNV	C1886T	S629L	M11	D	D	5.73
3	138763305	138763305	PRR23C	proline rich 23C	NM_001134657	G	A	nonsynonymous SNV	C158T	A53V	M11	T	D	-0.863
3	160225913	160225913	KPNA4	karyopherin alpha 4 (importin	NM_002268	G	A	nonsynonymous SNV	C1354T	L452F	M11	T	B	5.22
3	16242269	16242269	GALNT15	polypeptide N-acetylgalactosa	NM_054110	G	T	nonsynonymous SNV	G850T	A284S	M11	T	B	1.82
3	164777039	164777039	SI	sucrase-isomaltase (alpha-gl	NM_001041	T	A	nonsynonymous SNV	A1195T	T399S	M11	D	D	5.59
3	167183073	167183073	SERPINI2	serpin peptidase inhibitor, cla	NM_001012303.2	C	T	nonsynonymous SNV	G817A	E273K	M11	D	P	5.74
3	189612028	189612028	TP63	tumor protein p63	NM_003722.4	C	T	stopgain	C1780T	R594X	M11			5.02
3	48604446	48604446	COL7A1	collagen, type VII, alpha 1	NM_000094	C	T	nonsynonymous SNV	G8120A	G2707E	M11	D	D	4.59
3	49940808	49940808	MST1R	macrophage stimulating 1 rec	NM_002447.2	G	T	nonsynonymous SNV	C235A	H79N	M11	D	B	2.12
3	50645386	50645386	CISH	cytokine inducible SH2-conta	NM_013324.5	G	T	nonsynonymous SNV	C480A	N160K	M11	T	P	2.86
3	52955840	52955840	SFMBT1	Scm-like with four mbt domai	NM_016329	G	A	nonsynonymous SNV	C1139T	S380F	M11	T	B	1.35
3	54798283	54798283	CACNA2D3	Calcium channel, voltage-dep	NM_018398	G	A	nonsynonymous SNV	G1285A	E429K	M11	T	D	5.95
3	64004626	64004626	PSMD6	Proteasome (prosome, macro	NM_001271779	T	A	nonsynonymous SNV	A744T	K248N	M11	T	D	2.4
3	75714941	75714941	FRG2C	FSHD region gene 2 family, n	NM_001124759	C	T	nonsynonymous SNV	C598T	H200Y	M11	T	P	-1.35
3	93733599	93733599	STX19	Syntaxin 19	NM_001001850	A	G	nonsynonymous SNV	T515C	V172A	M11	D	D	4.76
3	9726584	9726584	MTMR14	Myotubularin related protein 1	NM_001077525	C	G	nonsynonymous SNV	C1063G	H355D	M11	D	D	5.83
4	106613242	106613242	INTS12	Integrator complex subunit 12	NM_001142471	T	A	nonsynonymous SNV	A548T	H183L	M11	T	D	5.46
4	123663799	123663799	BBS12	Bardet-Biedl syndrome 12	NM_001178007	G	T	nonsynonymous SNV	G752T	G251V	M11	D	B	3.44
4	126240913	126240913	FAT4	FAT atypical cadherin 4	NM_001291285	G	A	nonsynonymous SNV	G3347A	G1116E	M11	D	D	4.36
4	166418723	166418723	CPE	Carboxypeptidase E	NM_001873	G	A	nonsynonymous SNV	G1392A	M464I	M11	D	P	6.08
4	176622799	176622799	GPM6A	Glycoprotein M6A	NM_005277	C	T	nonsynonymous SNV	G157A	G53R	M11	T	D	6.01
4	183652188	183652188	TENM3	Teneurin transmembrane pro	NM_001080477	G	A	nonsynonymous SNV	G2863A	D955N	M11	T	B	5.12
4	25664462	25664462	SLC34A2	Solute carrier family 34 (type	NM_006424	C	T	nonsynonymous SNV	C248T	S83L	M11	D	P	5.24
4	47667154	47667154	CORIN	Corin, serine peptidase	NM_006587	G	A	nonsynonymous SNV	C1484T	P495L	M11	T	D	5.25
4	71347144	71347144	MUC7	Mucin 7, secreted	NM_001145006	C	T	nonsynonymous SNV	C683T	S228F	M11		B	-1.32
4	71498383	71498383	ENAM	Enamelin	NM_031889	C	T	stopgain	C184T	Q62X	M11	T		6.07
4	73179476	73179476	ADAMTS3	ADAM metallopeptidase with	NM_014243	C	T	nonsynonymous SNV	G1663A	G555R	M11	D	D	6.02
4	88533290	88533290	DSPP	Dentin sialophosphoprotein	NM_014208	G	A	nonsynonymous SNV	G85A	E29K	M11	D	P	3.12
5	102515794	102515794	PPIP5K2	Diphosphoinositol pentakisph	NM_001276277	C	T	stopgain	C2827T	R943X	M11	T		4.02
5	121739511	121739511	SNCAIP	Synuclein, alpha interacting p	NM_001242935	G	A	nonsynonymous SNV	G35A	R12Q	M11	T	B	3.85
5	133295535	133295535	C5orf15	Chromosome 5 open reading	NM_020199	G	A	nonsynonymous SNV	C316T	P106S	M11	T	B	3.15
5	13692194	13692194	DNAH5	Dynein, axonemal, heavy cha	NM_001369	G	A	stopgain	C13774T	R4592X	M11	T		5.76
5	13811812	13811812	DNAH5	Dynein, axonemal, heavy cha	NM_001369	C	T	nonsynonymous SNV	G7351A	E2451K	M11	D	B	5.78
5	156816384	156816384	CYFIP2	Cytoplasmic FMR1 interacting	NM_001291721	C	T	nonsynonymous SNV	C3317T	A1106V	M11	T	D	4.93
5	161116139	161116139	GABRA6	Gamma-aminobutyric acid (G	NM_000811	G	A	nonsynonymous SNV	G410A	R137K	M11	D	D	5.59
5	170722951	170722951	RANBP17	RAN binding protein 17	NM_022897	C	T	nonsynonymous SNV	C3103T	L1035F	M11	T	B	5.85
5	192030	192030	LRRC14B	Leucine rich repeat containin	NM_001080478	G	A	nonsynonymous SNV	G377A	G126E	M11	T	B	4.06
5	32048714	32048714	PDZD2	PDZ domain containing 2	NM_178140	T	C	nonsynonymous SNV	T1589C	I530T	M11	T	B	-5.98
5	55081160	55081160	DDX4	DEAD (Asp-Glu-Ala-Asp) box	NM_024415	G	A	nonsynonymous SNV	G661A	G221S	M11	T	B	3.41
5	64483988	64483988	ADAMTS6	ADAM metallopeptidase with	NM_197941	C	T	nonsynonymous SNV	G2765A	R922K	M11	D	D	5.37
5	73136510	73136510	ARHGEF28	Rho guanine nucleotide exch	NM_001080479	C	T	nonsynonymous SNV	C1352T	S451L	M11	T	B	2.06
5	94764359	94764359	FAM81B	Family with sequence similar	NM_152548	G	A	nonsynonymous SNV	G709A	E237K	M11	T	D	5.87
6	102503243	102503243	GRIK2	Glutamate receptor, ionotropi	NM_021956	C	T	stopgain	C2350T	Q784X	M11	T		5.68
6	126080691	126080691	HEY2	Hes-related family bHLH tran	NM_012259	C	T	nonsynonymous SNV	C757T	L253F	M11	T	P	5.61
6	129637295	129637295	LAMA2	Laminin, alpha 2	NM_000426	G	A	nonsynonymous SNV	G4037A	G1346E	M11	T	D	5.65
6	130762270	130762270	TMEM200A	Transmembrane protein 200A	NM_052913	G	A	nonsynonymous SNV	G703A	E235K	M11	T	B	5.69

6	131915473	131915473	MED23	Mediator complex subunit 23	NM_004830	G	A	nonsynonymous SNV	C2998T	R1000C	M11	D	D	6.03
6	133072417	133072417	VNN2	Vanin 2	NM_004665	C	T	nonsynonymous SNV	G1067A	G356E	M11	T	P	4.75
6	134210728	134210728	TCF21	Transcription factor 21	NM_003206	A	T	stopgain	A193T	K65X	M11	T		4.31
6	135318774	135318774	HBS1L	HBS1-like translational GTPa	NM_006620	C	A	nonsynonymous SNV	G560T	G187V	M11	D	D	5.3
6	135763729	135763729	AHI1	Abelson helper integration sit	NM_001134830	G	A	nonsynonymous SNV	C1903T	P635S	M11	T	D	5.66
6	150342289	150342289	RAET1L	Retinoic acid early transcript	NM_130900	T	C	nonsynonymous SNV	A383G	E128G	M11	D	P	-3.81
6	153365149	153365149	RGS17	Regulator of G-protein signali	NM_012419	C	T	nonsynonymous SNV	G5A	R2Q	M11	D	P	5.15
6	160969590	160969590	LPA	Lipoprotein, Lp(a)	NM_005577	C	T	nonsynonymous SNV	G5075A	R1692Q	M11	T	P	-5.41
6	25923931	25923931	SLC17A2	Solute carrier family 17, mem	NM_001286123	C	T	nonsynonymous SNV	G232A	D78N	M11	T	B	-2.62
6	2675285	2675285	MYLK4	Myosin light chain kinase fam	NM_001012418	G	A	nonsynonymous SNV	C1115T	A372V	M11	T	B	5.91
6	28227768	28227768	NKAPL	NFKB activating protein-like	NM_001007531	A	G	nonsynonymous SNV	A619G	N207D	M11	T	B	-7.83
6	31619473	31619473	BAG6	BCL2-associated athanogene	NM_004639	G	A	nonsynonymous SNV	C68T	T23I	M11	D	D	4.76
6	32171591	32171591	NOTCH4	Notch 4	NM_004557	C	T	nonsynonymous SNV	G3187A	A1063T	M11	T	B	2.29
6	33256783	33256783	WDR46	WD Repeat Domain 46	NM_005452	C	A	nonsynonymous SNV	G77T	R26L	M11	T	B	4.25
6	41162548	41162548	TREML2	Triggering Receptor Express	NM_024807	G	A	nonsynonymous SNV	C400T	P134S	M11	T	D	3.06
6	42981065	42981065	MEA1	Male-Enhanced Antigen 1	NM_014623	G	C	nonsynonymous SNV	C91G	P31A	M11	D	B	5.06
6	46847599	46847599	GPR116	adhesion G protein-coupled r	NM_001098518	G	A	nonsynonymous SNV	C992T	S331L	M11	T	B	4.99
6	52871184	52871184	ICK	Intestinal cell (MAK-like) kina	NM_016513	G	A	nonsynonymous SNV	C1673T	S558F	M11	D	D	5.63
6	55925721	55925721	COL21A1	Collagen, Type XXI, Alpha 1	NM_030820	G	A	nonsynonymous SNV	C2320T	P774S	M11	T	B	2.06
6	74229644	74229644	EEF1A1	Eukaryotic Translation Elonga	NM_001402	T	G	nonsynonymous SNV	A106C	K36Q	M11	D	P	4.15
6	75797349	75797349	COL12A1	Collagen, type XII, alpha 1	NM_004370	C	T	nonsynonymous SNV	G9125A	G3042E	M11	D	D	5.75
7	123594010	123594010	SPAM1	sperm adhesion molecule 1 (	NM_003117	A	C	nonsynonymous SNV	A386C	K129T	M11		B	-2.14
7	137792253	137792253	AKR1D1	Aldo-keto reductase family 1,	NM_005989	G	A	nonsynonymous SNV	G782A	R261H	M11	D	D	5.55
7	143453704	143453704	CTAGE6	CTAGE Family, Member 6	NM_178561	C	T	nonsynonymous SNV	G1048A	E350K	M11			
7	143657860	143657860	OR2F1	Olfactory Receptor, Family 2,	NM_012369	C	T	nonsynonymous SNV	C797T	P266L	M11	T	B	4.41
7	143771325	143771325	OR2A25	Olfactory Receptor, Family 2,	NM_001004488	C	T	stopgain	C13T	Q5X	M11	T		0.713
7	150094663	150094663	ZNF775	Zinc Finger Protein 775	NM_173680	C	T	nonsynonymous SNV	C1094T	A365V	M11		B	3.14
7	151962134	151962134	KMT2C	Lysine (K)-Specific Methyltra	NM_170606	G	T	stopgain	C1173A	C391X	M11	T	D	
7	151962168	151962168	KMT2C	Lysine (K)-Specific Methyltra	NM_170606	C	A	nonsynonymous SNV	G1139T	R380L	M11	T	D	4.65
7	21521570	21521570	SP4	Sp4 transcription factor	NM_003112	C	T	stopgain	C1936T	Q646X	M11	T		5.47
7	40041528	40041528	CDK13	Cyclin-dependent kinase 13	NM_003718	C	T	stopgain	C2251T	R751X	M11			1.31
7	63680022	63680022	ZNF735	Zinc finger protein 735	NM_001159524	C	T	nonsynonymous SNV	C593T	S198L	M11			
7	82580270	82580270	PCLO	Piccolo presynaptic cytomatri	NM_033026	G	T	nonsynonymous SNV	C9634A	Q3212K	M11	D	P	5.33
7	84710865	84710865	SEMA3D	Sema domain, immunoglobul	NM_152754	C	T	nonsynonymous SNV	G343A	E115K	M11	D	P	5.47
7	98792945	98792945	KPNA7	Karyopherin alpha 7 (importin	NM_001145715	C	T	nonsynonymous SNV	G301A	E101K	M11	D	B	4.69
8	106814156	106814156	ZFPM2	Zinc finger protein, FOG fami	NM_012082	C	T	nonsynonymous SNV	C1846T	P616S	M11	T	B	3.9
8	108276562	108276562	ANGPT1	Angiopoietin 1	NM_001146	T	G	nonsynonymous SNV	A1223C	H408P	M11	D	D	5.97
8	110464379	110464379	PKHD1L1	Polycystic kidney and hepatic	NM_177531	C	T	nonsynonymous SNV	C6377T	T2126I	M11	T	P	1.97
8	139890514	139890514	COL22A1	Collagen, type XXII, alpha 1	NM_152888	G	A	nonsynonymous SNV	C137T	S46F	M11		D	5.19
8	145737644	145737644	RECQL4	RecQ protein-like 4	NM_004260	G	A	nonsynonymous SNV	C3119T	P1040L	M11			
8	17869206	17869206	PCM1	Pericentriolar material 1	NM_006197	C	T	nonsynonymous SNV	C5381T	T1794I	M11	D	D	3.87
8	27293809	27293809	PTK2B	Protein tyrosine kinase 2 beta	NM_173174	C	T	nonsynonymous SNV	C1285T	R429C	M11	D	D	3.67
8	57228854	57228854	SDR16C5	Short chain dehydrogenase/r	NM_138969	G	A	nonsynonymous SNV	C53T	S18L	M11	T	B	-0.292
8	61778412	61778412	CHD7	Chromodomain helicase DNA	NM_017780	G	T	stopgain	G8914T	E2972X	M11	D		4.47
8	87683280	87683280	CNGB3	Cyclic nucleotide gated chan	NM_019098	C	T	nonsynonymous SNV	G385A	D129N	M11	T	B	4.79
8	88885976	88885976	DCAF4L2	DDB1 and CUL4 associated	NM_152418	T	G	nonsynonymous SNV	A224C	N75T	M11	D	B	1.92
9	113308559	113308559	SVEP1	Sushi, von Willebrand factor	NM_153366	C	T	nonsynonymous SNV	G800A	G267E	M11	D	D	5.74
9	113445053	113445053	MUSK	muscle, skeletal, receptor tyr	NM_005592	C	T	nonsynonymous SNV	C179T	S60F	M11	T	P	5.57
9	126144643	126144643	DENND1A	DENN/MADD Domain Contai	NM_020946	G	A	nonsynonymous SNV	C2098T	P700S	M11	T	D	4.15
9	127951939	127951939	PPP6C	Protein phosphatase 6, catal	NM_001123355	G	A	nonsynonymous SNV	C59T	P20L	M11	D	P	4.81
9	129642756	129642756	ZBTB34	Zinc Finger And BTB Domain	NM_001099270	C	T	nonsynonymous SNV	C1066T	P356S	M11	T	B	5.88
9	13190207	13190207	MPDZ	Multiple PDZ domain protein	NM_003829	A	G	nonsynonymous SNV	T2060C	V687A	M11	T	N	2.08
9	139369268	139369268	SEC16A	SEC16 Homolog A	NM_014866	G	T	nonsynonymous SNV	C2800A	P934T	M11	T	D	3.61
9	2161869	2161869	SMARCA2	SWI/SNF related, matrix asso	NM_003070	G	T	nonsynonymous SNV	G4165T	A1389S	M11	T	B	5.1
9	33948481	33948481	UBAP2	Ubiquitin associated protein 2	NM_001282529	C	A	nonsynonymous SNV	G360T	Q120H	M11	T	D	2.1
9	35102161	35102161	STOML2	Stomatin (EPB72)-like 2	NM_001287032	G	A	nonsynonymous SNV	C61T	R21W	M11	D	D	5.56
9	35547189	35547189	RUSC2	RUN and SH3 domain contai	NM_001135999	C	T	nonsynonymous SNV	C671T	S224F	M11	D	P	5.09
9	40772993	40772993	ZNF658	Zinc finger protein 658	NM_033160	C	A	nonsynonymous SNV	G2282T	R761L	M11	T	B	-0.563
9	90585502	90585502	CDK20	Cyclin-dependent kinase 20	NM_001039803	C	T	nonsynonymous SNV	G544A	D182N	M11	D	B	4.73
9	93650819	93650819	SYK	Spleen tyrosine kinase	NM_003177	C	T	nonsynonymous SNV	C1745T	T582I	M11	T	B	5.22
9	96070639	96070639	WNK2	WNK lysine deficient protein	NM_001282394	C	T	nonsynonymous SNV	C6400T	P2134S	M11	T	P	5.45
X	102755366	102755366	RAB40A	RAB40A, member RAS onco	NM_080879	G	A	stopgain	C319T	R107X	M11	T		0.225
X	105189925	105189925	NRK	Nik related kinase	NM_198465	G	A	nonsynonymous SNV	G4121A	R1374Q	M11	D	B	3.47
X	11174649	11174649	ARHGAP6	Rho GTPase activating prote	NM_013427	G	A	nonsynonymous SNV	C1907T	S636L	M11		B	4.65
X	117712597	117712597	DOCK11	Dedicator of cytokinesis 11	NM_144658	C	T	nonsynonymous SNV	C1499T	S500F	M11	T	D	5.77
X	119249586	119249586	RHOXF1	Rhox homeobox family, mem	NM_139282	C	T	nonsynonymous SNV	G187A	D63N	M11	T	B	-2.24
X	12632951	12632951	FRMPD4	FERM and PDZ domain cont	NM_014728	G	A	nonsynonymous SNV	G373A	D125N	M11	T	D	5.55

X	129270094	129270094	AIFM1	Apoptosis-inducing factor, mi	NM_004208	C	T	nonsynonymous SNV	G1231A	G411S	M11	D	P	6
X	134186056	134186056	FAM127B	Family with sequence similari	NM_001078172	G	A	nonsynonymous SNV	C83T	P28L	M11	D	D	2.38
X	135323348	135323348	MAP7D3	MAP7 domain containing 3	NM_024597	G	A	nonsynonymous SNV	C506T	S169F	M11	D	D	1.13
X	135761728	135761728	ARHGEF6	Rac/Cdc42 guanine nucleotic	NM_004840	G	A	nonsynonymous SNV	C1796T	P599L	M11	D	D	4.7
X	138864823	138864823	ATP11C	ATPase, class VI, type 11C	NM_173694	T	G	nonsynonymous SNV	A1844C	N615T	M11	T	B	5.68
X	149013215	149013215	MAGEA8	Melanoma antigen family A8	NM_001166400	G	T	nonsynonymous SNV	G169T	G57W	M11	D	D	-0.342
X	149013233	149013233	MAGEA8	Melanoma antigen family A8	NM_001166400	C	T	stopgain	C187T	Q63X	M11	T		1.03
X	151304001	151304001	MAGEA10	Melanoma antigen family A1	NM_001011543	G	A	nonsynonymous SNV	C92T	P31L	M11	T	B	-0.808
X	153763423	153763423	G6PD	Glucose-6-phosphate dehydr	NM_000402	C	G	nonsynonymous SNV	G535C	A179P	M11	T	B	4.76
X	154507236	154507236	CLIC2	Chloride intracellular channel	NM_001289	C	T	nonsynonymous SNV	G700A	E234K	M11	D	D	4.86
X	30712565	30712565	GK	Glycerol kinase	NM_001205019	C	T	nonsynonymous SNV	C463T	R155C	M11	D	D	5.46
X	40542175	40542175	MED14	Mediator complex subunit 14	NM_004229	G	A	stopgain	C2131T	R711X	M11	T		5.7
X	51639881	51639881	MAGED1	Melanoma antigen family D1	NM_001005333	C	T	nonsynonymous SNV	C1298T	P433L	M11	T	B	2.95
X	70387352	70387352	NLGN3	Neuroligin 3	NM_018977	A	C	nonsynonymous SNV	A1345C	T449P	M11	D	D	4.9
X	72433623	72433623	NAP1L2	Nucleosome assembly protei	NM_021963	C	T	nonsynonymous SNV	G706A	D236N	M11	T	D	0.824
X	75003383	75003383	MAGEE2	Melanoma antigen family E2	NM_138703	C	A	nonsynonymous SNV	G1504T	A502S	M11	D	P	1.81
X	79937511	79937511	BRWD3	Bromodomain and WD repea	NM_153252	G	A	nonsynonymous SNV	C4480T	P1494S	M11		B	4.09
7	140453136	140453136	BRAF	v-raf murine sarcoma viral o	NM_004333	A	T	nonsynonymous SNV	T1799A	V600E	M11	D	D	5.65
19	49558211	49558211	CGB7	Chorionic Gonadotropin, Beta	NM_033142	T	G	nonsynonymous SNV	A70C	M24L	M11			946
7	100645960	100645960	MUC12	Mucin12	NM_001164462	A	T	nonsynonymous SNV	A12116T	D4039V	M11	T		-1.72
1	248801602	248801602	OR2T35	Olfattory Receptor Family 2,	NM_001001827	0	CA	stopgain	957_958insTG	I320_R321delinsX	M11			
11	77614590	77614590	INTS4	Integrator Complex Subunit 4	NM_033547	T	G	nonsynonymous SNV	A2093C	K698T	M11	T	B	4.41
7	100639761	100639761	MUC12	Mucin12	NM_001164462	G	C	nonsynonymous SNV	G5917C	D1973H	M11	T		-275
19	49558216	49558216	CGB7	Chorionic Gonadotropin, Beta	NM_033142	C	T	nonsynonymous SNV	G65A	R22K	M11	T		-353
5	43277335	43277335	NIM1K	NM1 Serine/treonin Kinase	NM_153361	G	C	nonsynonymous SNV	G469C	E157Q	M11	D	D	5.91
1	152129130	152129130	RPTN	repetin	NM_001122965	G	T	nonsynonymous SNV	C445A	P149T	M11	T	B	-10.4
1	214171121	214171121	PROX1	prospero homeobox 1	NM_001270616.1	G	A	nonsynonymous SNV	G1243A	V415I	M14	T	D	5.43
10	100453657	100453657	HPSE2	heparanase 2 (inactive)	NM_021828.4	T	C	nonsynonymous SNV	A1004G	H335R	M14		P	5.84
10	46254776	46254776	FAM21C	family with sequence similarit	NM_015262.2	A	C	nonsynonymous SNV	A1562C	Y521S	M14	T	B	3.26
10	49994817	49994817	WDFY4	WDFY family member 4	NM_020945	C	A	nonsynonymous SNV	C3710A	T1237K	M14		B	5.31
12	123065158	123065158	KNTC1	kinetochore associated 1	NM_014708	C	T	stopgain	C2923T	Q975X	M14	T		4.97
12	25378562	25378562	KRAS	Kirsten rat sarcoma viral onc	NM_004985.4	C	T	nonsynonymous SNV	G436A	A146T	M14	D	D	5.52
12	53668689	53668689	ESPL1	extra spindle pole bodies like	NM_012291	C	G	nonsynonymous SNV	C1595G	A532G	M14	T	B	2.91
13	101717769	101717769	NALCN	sodium leak channel, non sel	NM_052867	G	A	nonsynonymous SNV	C4591T	H1531Y	M14	D	D	5.71
14	102461124	102461124	DYNC1H1	dynein, cytoplasmic 1, heavy	NM_001376	G	A	nonsynonymous SNV	G3271A	G1091R	M14	T	B	5.73
15	48431333	48431333	SLC24A5	solute carrier family 24 (sodiu	NM_205850	G	A	nonsynonymous SNV	G1039A	A347T	M14	T	P	4.02
16	2126130	2126130	TSC2	tuberous sclerosis 2	NM_000548.3	C	T	nonsynonymous SNV	C2701T	R901C	M14	D	D	5.09
16	2546619	2546619	TBC1D24	TBC1 domain family, membe	NM_001199107.1	G	A	nonsynonymous SNV	G470A	R157H	M14	T	B	4.11
16	28507445	28507445	APOBR	apolipoprotein B receptor	NM_018690	G	C	nonsynonymous SNV	G1083C	E361D	M14	T	B	-2
16	28507452	28507452	APOBR	apolipoprotein B receptor	NM_018690	G	T	nonsynonymous SNV	G1090T	G364W	M14	D	B	-2.49
16	53191380	53191380	CHD9	chromodomain helicase DNA	NM_025134	C	A	nonsynonymous SNV	C1379A	A460D	M14	T	B	1.39
16	70563087	70563087	SF3B3	splicing factor 3b, subunit 3,	NM_012426	C	G	nonsynonymous SNV	C382G	R128G	M14	D	D	3.65
17	4996073	4996073	ZFP3	ZFP3 zinc finger protein	NM_153018	A	C	nonsynonymous SNV	A1274C	E425A	M14	D	B	2.79
18	21110511	21110511	C18orf8	chromosome 18 open readin	NM_013326.4	A	G	nonsynonymous SNV	A1829G	Y610C	M14	D	D	5.8
18	60646603	60646603	PHLPP1	PH domain and leucine rich r	NM_194449	C	T	nonsynonymous SNV	C5093T	P1698L	M14	D	B	-0.764
19	1004963	1004963	GRIN3B	glutamate receptor, ionotropi	NM_138690	C	G	nonsynonymous SNV	C1463G	A488G	M14	D	D	4.43
19	1879987	1879987	ABHD17A	abhydrolase domain containi	NM_031213.3	A	C	nonsynonymous SNV	T613G	S205A	M14	D	B	2.21
19	34824610	34824610	KIAA0355	KIAA0355	NM_014686	T	G	nonsynonymous SNV	T1265G	V422G	M14	D	D	5.63
19	58868676	58868676	ZNF497	zinc finger protein 497	NM_198458.2	C	T	nonsynonymous SNV	G326A	G109E	M14	T	B	0.032
2	113742550	113742550	IL36G	interleukin 36, gamma	NM_019618.3	G	C	nonsynonymous SNV	G434C	R145T	M14	T	B	-8.78
2	14775367	14775367	LOC653602	LOC653602	NM_001291410	C	A	nonsynonymous SNV	C41A	S14Y	M14			
2	212576817	212576817	ERBB4	erb-b2 receptor tyrosine kina	NM_005235.2	T	A	nonsynonymous SNV	A1082T	K361M	M14	D	D	5.05
2	214174893	214174893	SPAG16	sperm associated antigen 16	NM_024532.4	G	T	nonsynonymous SNV	G390T	Q130H	M14	D	D	1.02
2	74702163	74702163	CCDC142	coiled-coil domain containing	NM_032779	G	A	nonsynonymous SNV	C1855T	P619S	M14	T	B	1.75
2	84934797	84934797	DNAH6	dynein, axonemal, heavy cha	NM_001370	G	A	nonsynonymous SNV	G9005A	C3002Y	M14	T	P	5.58
22	50315971	50315971	CRELD2	cysteine-rich with EGF-like d	NM_001135101	C	G	nonsynonymous SNV	C619G	P207A	M14	T	B	-2.64
3	139094367	139094367	COPB2	coatomer protein complex, su	NM_004766	G	T	nonsynonymous SNV	C594A	D198E	M14	T	B	4.48
3	151474822	151474822	AADACL2	arylacetamide deacetylase-lik	NM_207365	G	A	nonsynonymous SNV	G646A	V216I	M14	T	B	4.05
3	3189176	3189176	TRNT1	tRNA nucleotidyl transferase,	NM_182916	G	C	nonsynonymous SNV	G845C	S282T	M14	T	B	3.83
3	48664065	48664065	SLC26A6	solute carrier family 26 (anior	NM_022911.2	G	C	nonsynonymous SNV	C2127G	H709Q	M14	T	B	-0.162
3	49770300	49770300	IP6K1	inositol hexakisphosphate kin	NM_153273.3	G	C	nonsynonymous SNV	C540G	H180Q	M14	T	B	0.6
4	46067589	46067589	GABRG1	Gamma-aminobutyric acid (G	NM_173536	C	G	nonsynonymous SNV	G334C	D112H	M14	D	D	5.08
5	179996160	179996160	CNOT6	CCR4-NOT transcription com	NM_015455	G	T	nonsynonymous SNV	G1078T	A360S	M14		D	5.86
6	128302261	128302261	PTPRK	Protein tyrosine phosphatase	NM_001291981	G	T	nonsynonymous SNV	C3777A	D1259E	M14		D	-8.16
6	31239378	31239378	HLA-C	Major histocompatibility comp	NM_002117	T	G	nonsynonymous SNV	A341C	D114A	M14	T	B	-1.89
7	140453155	140453155	BRAF	B-Raf Proto-Oncogene, Serir	NM_004333	C	T	nonsynonymous SNV	G1780A	D594N	M14	D	D	5.65
8	52387647	52387647	PXDNL	Peroxidasin-like	NM_144651	C	G	nonsynonymous SNV	G579C	E193D	M14	T	B	-5.07

9	119065180	119065180	PAPPA	Pregnancy-Associated Plasm	NM_002581	A	G	nonsynonymous SNV	A3098G	D1033G	M14	D	P	5.82
9	39099985	39099985	CNTNAP3	Contactin Associated Protein-	NM_033655	C	T	nonsynonymous SNV	G2918A	G973E	M14	D	D	3.68
X	115577943	115577943	SLC6A14	Solute carrier family 6 (aminc	NM_007231	C	A	nonsynonymous SNV	C826A	L276I	M14	D	P	-0.28
X	129518458	129518458	GPR119	G protein-coupled receptor 1	NM_178471	A	T	nonsynonymous SNV	T964A	S322T	M14	T	B	2.8
X	36122731	36122731	CHDC2	Cilia and flagella associated	NM_173695	C	T	nonsynonymous SNV	C968T	S323F	M14	T	B	2.64
X	41495910	41495910	CASK	Calcium/calmodulin-depende	NM_003688	C	T	nonsynonymous SNV	G836A	R279Q	M14	D	P	5.78
X	47426060	47426060	ARAF	A-Raf proto-oncogene, serine	NM_001654	C	G	nonsynonymous SNV	C580G	P194A	M14	T	B	4.46
X	47426061	47426061	ARAF	A-Raf proto-oncogene, serine	NM_001654	C	A	nonsynonymous SNV	C581A	P194Q	M14	T	B	1.02
1	10493999	10493999	APITD1,APITD1	APITD1-CORT readthrough	NM_198544.3	C	T	nonsynonymous SNV	C152T	S51L	M16	T	D	4.28
1	109839740	109839740	MYBPHL	myosin binding protein H-like	NM_001010985.2	G	A	nonsynonymous SNV	C502T	P168S	M16	T	P	2.85
1	1133192	1133192	TTLL10	tubulin tyrosine ligase-like far	NM_001130045	G	A	nonsynonymous SNV	G1987A	E663K	M16	T	B	1.42
1	117944839	117944839	MAN1A2	mannosidase, alpha, class 1/	NM_006699	C	T	stopgain	C334T	R112X	M16	T		4.73
1	119934859	119934859	HAO2	hydroxyacid oxidase 2	NM_016527.3	G	A	nonsynonymous SNV	G898A	G300R	M16		D	5.17
1	1275465	1275465	DVL1	dishevelled segment polarity	NM_004421	C	G	nonsynonymous SNV	G862C	A288P	M16	D	D	3.43
1	12939817	12939817	PRAMEF4	PRAME family member 4	NM_001009611	C	T	nonsynonymous SNV	G985A	D329N	M16	T	B	-2.05
1	12943170	12943170	PRAMEF4	PRAME family member 4	NM_001009611	C	T	nonsynonymous SNV	G46A	G16R	M16	T	B	-0.706
1	152058267	152058267	TCHHL1	trichohyalin-like 1	NM_001008536	C	A	stopgain	G1891T	G631X	M16	T		-1.64
1	152059763	152059763	TCHHL1	trichohyalin-like 1	NM_001008536	C	T	nonsynonymous SNV	G395A	R132K	M16	T	B	1.42
1	152284394	152284394	FLG	filaggrin	NM_002016	C	T	nonsynonymous SNV	G2968A	E990K	M16	T	B	1.42
1	152324797	152324797	FLG2	filaggrin family member 2	NM_001014342	C	T	nonsynonymous SNV	G5465A	R1822Q	M16	T	B	0.594
1	152484015	152484015	LCE5A	late cornified envelope 5A	NM_178438	C	T	nonsynonymous SNV	C5T	S2F	M16	D	D	5.28
1	152484159	152484159	LCE5A	late cornified envelope 5A	NM_178438	C	T	nonsynonymous SNV	C149T	S50F	M16	D	P	2.23
1	153029109	153029109	SPRR2A	small proline-rich protein 2A	NM_005988	G	A	nonsynonymous SNV	C103T	P35S	M16	D	D	2.79
1	153314103	153314103	PGLYRP4	peptidoglycan recognition prc	NM_020393	C	T	nonsynonymous SNV	G625A	A209T	M16		P	4.2
1	153789954	153789954	GATAD2B	GATA zinc finger domain con	NM_020699	A	G	nonsynonymous SNV	T794C	V265A	M16	T	B	6.17
1	156883829	156883829	PEAR1	platelet endothelial aggregati	NM_001080471	C	G	nonsynonymous SNV	C2899G	R967G	M16	T	B	1.39
1	157548560	157548560	FCRL4	Fc receptor-like 4	NM_031282	C	T	nonsynonymous SNV	G1360A	V454I	M16		B	3.92
1	157653007	157653007	FCRL3	Fc receptor-like 3	NM_052939	C	T	nonsynonymous SNV	G1838A	S613N	M16		B	4.41
1	158262056	158262056	CD1C	CD1c molecule	NM_001765	G	A	nonsynonymous SNV	G511A	E171K	M16	T	B	-1.07
1	158670295	158670295	OR6K2	olfactory receptor, family 6, su	NM_001005279	G	A	stopgain	C148T	Q50X	M16	T		3.73
1	158724778	158724778	OR6K6	olfactory receptor, family 6, su	NM_001005184	C	T	nonsynonymous SNV	C173T	P58L	M16	T	B	-0.06
1	162335286	162335286	NOS1AP	nitric oxide synthase 1 (neurc	NM_014697.2	C	A	nonsynonymous SNV	C1032A	D344E	M16	T	D	5.62
1	169439403	169439403	SLC19A2	solute carrier family 19 (thiam	NM_006996	G	A	nonsynonymous SNV	C829T	L277F	M16	T	B	3.97
1	172633498	172633498	FASLG	Fas ligand (TNF superfamily,	NM_000639	A	C	nonsynonymous SNV	A419C	K140T	M16	T	B	2.39
1	17316456	17316456	ATP13A2	ATPase type 13A2	NM_022089.3	G	A	stopgain	C2455T	R819X	M16	T		2.53
1	175332847	175332847	TNR	tenascin R	NM_003285	G	A	stopgain	C2704T	Q902X	M16	T		4.58
1	175332856	175332856	TNR	tenascin R	NM_003285	G	A	stopgain	C2695T	R899X	M16	T		5.5
1	176104155	176104155	RFWD2	ring finger and WD repeat do	NM_022457.6	G	A	nonsynonymous SNV	C959T	P320L	M16	T	D	4.48
1	177901896	177901896	SEC16B	SEC16 homolog B, endoplas	NM_033127	A	C	nonsynonymous SNV	T2869G	S957A	M16	T	D	4.6
1	181686370	181686370	CACNA1E	calcium channel, voltage-dep	NM_001205293.1	T	C	nonsynonymous SNV	T1457C	V486A	M16	D	D	5.32
1	181764064	181764064	CACNA1E	calcium channel, voltage-dep	NM_001205293.1	C	T	nonsynonymous SNV	C6092T	S2031F	M16	D	P	4.94
1	181767540	181767540	CACNA1E	calcium channel, voltage-dep	NM_001205293.1	C	T	nonsynonymous SNV	C6512T	S2171F	M16	D	D	5.55
1	182845605	182845605	DHX9	DEAH (Asp-Glu-Ala-His) box	NM_001357	C	T	nonsynonymous SNV	C2053T	P685S	M16	D	T	5.54
1	182847164	182847164	DHX9	DEAH (Asp-Glu-Ala-His) box	NM_001357	T	C	nonsynonymous SNV	T2207C	V736A	M16	D	P	5.92
1	196887357	196887357	CFHR4	complement factor H-related	NM_001201550.2	G	A	nonsynonymous SNV	G1558A	E520K	M16	T	P	1.03
1	196964973	196964973	CFHR5	complement factor H-related	NM_030787	G	A	nonsynonymous SNV	G734A	G245E	M16	T	D	3.49
1	198498237	198498237	ATP6V1G3	ATPase, H+ transporting, lysc	NM_133262	T	C	nonsynonymous SNV	A157G	K53E	M16	D	B	1.31
1	200969055	200969055	KIF21B	kinesin family member 21B	NM_001252100.1	G	A	stopgain	C1723T	Q575X	M16	T		4.88
1	200977926	200977926	KIF21B	kinesin family member 21B	NM_001252100.1	C	T	nonsynonymous SNV	G418A	E140K	M16	T	D	5.57
1	201178297	201178297	IGFN1	immunoglobulin-like and fibrc	NM_001164586	G	A	nonsynonymous SNV	G4276A	E1426K	M16			1.69
1	201179181	201179181	IGFN1	immunoglobulin-like and fibrc	NM_001164586	G	T	nonsynonymous SNV	G5160T	M1720I	M16			-0.645
1	201179738	201179738	IGFN1	immunoglobulin-like and fibrc	NM_001164586	C	A	nonsynonymous SNV	C5717A	A1906E	M16			-5.23
1	201179745	201179745	IGFN1	immunoglobulin-like and fibrc	NM_001164586	T	A	nonsynonymous SNV	T5724A	F1908L	M16			-3.29
1	203053777	203053777	MYOG	myogenin (myogenic factor 4	NM_002479	C	T	nonsynonymous SNV	G551A	G184E	M16	T	B	2.06
1	207680076	207680076	CR1	complement component (3b/4	NM_000651.4	C	T	nonsynonymous SNV	C319T	P107S	M16	T	D	2.94
1	207890877	207890877	CR1L	complement component (3b/4	NM_175710	C	T	nonsynonymous SNV	C1483T	P495S	M16	T	B	3.01
1	215960098	215960098	USH2A	Usher syndrome 2A (autoson	NM_206933	G	A	nonsynonymous SNV	C10301T	S3434F	M16	T	B	3.97
1	216850658	216850658	ESRRG	estrogen-related receptor gar	NM_001438.3	C	T	nonsynonymous SNV	G232A	G78R	M16	T	D	6.16
1	223568258	223568258	CCDC185	coiled-coil domain containing	NM_152610	G	A	nonsynonymous SNV	G1441A	E481K	M16	D	P	2.78
1	224008952	224008952	TP53BP2	tumor protein p53 binding prc	NM_001031685	A	T	nonsynonymous SNV	T145A	C49S	M16	D	D	5.75
1	224345354	224345354	FBXO28	F-box protein 28	NM_015176	C	T	nonsynonymous SNV	C1013T	S338F	M16	D	B	5.97
1	228506635	228506635	OBSCN	obscurin, cytoskeletal calmoc	NM_052843.3	C	T	nonsynonymous SNV	C14182T	P4728S	M16	T	D	3.81
1	22919855	22919855	EPHA8	EPH receptor A8	NM_020526	C	G	nonsynonymous SNV	C1352G	A451G	M16	D	B	4.27
1	22970626	22970626	C1QC	complement component 1, q	NM_001114101.1	G	A	nonsynonymous SNV	G110A	G37E	M16	D	D	5.26
1	231061316	231061316	TTC13	tetratricopeptide repeat doma	NM_024525.4	G	A	nonsynonymous SNV	C1535T	S512F	M16	D	P	5.75
1	233394089	233394089	PCNXL2	pecanex-like 2	NM_014801	C	T	nonsynonymous SNV	G1519A	G507R	M16	T	B	4.15
1	233515429	233515429	KIAA1804	mixed lineage kinase 4	NM_032435	A	T	nonsynonymous SNV	A2677T	T893S	M16	T	B	3.58

1	236729311	236729311	HEATR1	HEAT repeat containing 1	NM_018072	A	T	nonsynonymous SNV	T4343A	F1448Y	M16	T	B	4.7
1	243419531	243419531	SDCCAG8	serologically defined colon ca	NM_006642	G	A	nonsynonymous SNV	G56A	R19Q	M16	T	P	4.6
1	244780960	244780960	C1orf101	chromosome 1 open reading	NM_001130957.1	C	T	nonsynonymous SNV	C2620T	R874C	M16	T	D	2.68
1	245849638	245849638	KIF26B	kinesin family member 26B	NM_018012	C	T	nonsynonymous SNV	C3353T	S1118F	M16	D	P	3.74
1	245861540	245861540	KIF26B	kinesin family member 26B	NM_018012	C	T	nonsynonymous SNV	C5957T	S1118F	M16	D	P	5.68
1	24786981	24786981	NIPAL3	NIPA-like domain containing 3	NM_020448	T	A	nonsynonymous SNV	T873A	F291L	M16	T	D	-3.11
1	247875171	247875171	OR6F1	olfactory receptor, family 6, su	NM_001005286	T	A	nonsynonymous SNV	A887T	E296V	M16	D	B	3.49
1	247875270	247875270	OR6F1	olfactory receptor, family 6, su	NM_001005286	G	A	nonsynonymous SNV	C788T	S263F	M16	D	P	1.54
1	248084579	248084579	OR2T8	olfactory receptor, family 2, su	NM_001005522	G	A	nonsynonymous SNV	G260A	S87N	M16	T	B	-6.49
1	248112794	248112794	OR2L8	olfactory receptor, family 2, su	NM_001001963	G	C	nonsynonymous SNV	G635C	G212A	M16	T	B	-3.61
1	248344218	248344218	OR2M2	olfactory receptor, family 2, su	NM_001004688	G	A	nonsynonymous SNV	G931A	E311K	M16	T	B	-3.33
1	248436683	248436683	OR2T33	olfactory receptor, family 2, su	NM_001004695	G	A	nonsynonymous SNV	C434T	S145L	M16	D	B	-0.748
1	26164006	26164006	AUNIP	aurora kinase A and ninein in	NM_001287490.1	G	A	nonsynonymous SNV	C118T	L40F	M16	T	B	-2.79
1	3328068	3328068	PRDM16	PR domain containing 16	NM_022114	C	T	nonsynonymous SNV	C1307T	S436F	M16	D	D	5.26
1	34071511	34071511	CSMD2	CUB and Sushi multiple doma	NM_052896	G	A	nonsynonymous SNV	C6301T	P2101S	M16			
1	3647553	3647553	TP73	tumor protein p73	NM_005427.3	C	T	nonsynonymous SNV	C1408T	H470Y	M16	D	P	5.13
1	41012675	41012675	ZNF684	zinc finger protein 684	NM_152373	A	C	nonsynonymous SNV	A680C	K227T	M16	T	B	1.72
1	41979220	41979220	HIVEP3	human immunodeficiency viru	NM_024503.4	C	A	nonsynonymous SNV	G5672T	R1891L	M16	D	B	-6.49
1	44071282	44071282	PTPRF	protein tyrosine phosphatase	NM_002840.3	G	A	nonsynonymous SNV	G3472A	E1158K	M16	T	B	3.13
1	45250871	45250871	BEST4	bestrophin 4	NM_153274	C	T	nonsynonymous SNV	G821A	G274D	M16	T	B	1.57
1	46501047	46501047	MAST2	microtubule associated serine	NM_015112	G	A	nonsynonymous SNV	G4706A	G1569E	M16	T	B	-1.18
1	46725680	46725680	RAD54L	RAD54-like (S. cerevisiae)	NM_003579.3	C	T	nonsynonymous SNV	C316T	R106C	M16	T	D	5.29
1	47498981	47498981	CYP4X1	cytochrome P450, family 4, s	NM_178033	C	T	nonsynonymous SNV	C433T	H145Y	M16	D	D	2.91
1	50610812	50610812	ELAVL4	ELAV like neuron-specific RN	NM_021952.4	C	T	nonsynonymous SNV	C193T	L65F	M16	D	D	6.17
1	54060403	54060403	GLIS1	zinc finger, CCHC domain co	NM_147193	G	A	nonsynonymous SNV	C173T	P58L	M16	T	B	4.53
1	57169736	57169736	PRKAA2	protein kinase, AMP-activated	NM_006252	A	T	nonsynonymous SNV	A881T	K294I	M16	T	B	3.55
1	57209907	57209907	C1orf168	chromosome 1 open reading	NM_001004303	C	T	nonsynonymous SNV	G1420A	E474K	M16	T	D	2.37
1	57233561	57233561	C1orf168	chromosome 1 open reading	NM_001004303	G	A	nonsynonymous SNV	C1004T	S335L	M16	T	P	5.18
1	60312778	60312778	HOOK1	hook microtubule-tethering pr	NM_015888	G	A	nonsynonymous SNV	G850A	E284K	M16	T	D	6.05
1	70505181	70505181	LRRC7	leucine rich repeat containing	NM_020794	G	A	nonsynonymous SNV	G3560A	G1187E	M16	T	P	5.03
1	75702693	75702693	SLC44A5	solute carrier family 44, mem	NM_152697.4	C	T	nonsynonymous SNV	G719A	R240K	M16	T	B	0.193
1	78478831	78478831	DNAJB4	DnaJ (Hsp40) homolog, subfa	NM_007034	G	A	nonsynonymous SNV	G308A	G103E	M16	D	D	4.46
1	7887537	7887537	PER3	period circadian clock 3	NM_001289862.1	C	T	nonsynonymous SNV	C2548T	P850S	M16	T	D	0.942
1	79470830	79470830	ELTD1	adhesion G protein-coupled r	NM_022159	A	T	nonsynonymous SNV	T97A	C33S	M16		P	5.31
1	8420970	8420970	RERE	arginine-glutamic acid dipepti	NM_012102.3	G	A	nonsynonymous SNV	C2597T	P866L	M16	T	D	5.18
1	8420971	8420971	RERE	arginine-glutamic acid dipepti	NM_012102.3	G	A	nonsynonymous SNV	C2596T	P866S	M16	T	D	5.18
1	89834165	89834165	GBP6	guanylate binding protein fam	NM_198460	G	A	nonsynonymous SNV	G55A	E19K	M16	T	B	2.46
10	116225556	116225556	ABLIM1	actin binding LIM protein 1	NM_002313.5	G	A	nonsynonymous SNV	C1342T	H448Y	M16	T	B	5.41
10	120933972	120933972	PRDX3	peroxiredoxin 3	NM_006793.4	G	A	nonsynonymous SNV	C302T	P101L	M16	D	D	4.89
10	121551564	121551564	INPP5F	inositol polyphosphate-5-phos	NM_001243195	C	T	nonsynonymous SNV	C628T	R210C	M16		B	3.28
10	124909368	124909368	HMX2	H6 family homeobox 2	NM_005519	C	T	nonsynonymous SNV	C551T	S184F	M16	D	D	3.35
10	129350736	129350736	NPS	neuropeptide S	NM_001030013	T	C	nonsynonymous SNV	T103C	S35P	M16	T	B	-3.1
10	16996430	16996430	CUBN	cubilin (intrinsic factor-cobala	NM_001081	G	A	nonsynonymous SNV	C4813T	P1605S	M16	T	B	-7.75
10	17199746	17199746	TRDMT1	tRNA aspartic acid methyltran	NM_004412	G	A	nonsynonymous SNV	C581T	P194L	M16	T	P	5.41
10	18250624	18250624	SLC39A12	solute carrier family 39 (zinc t	NM_001145195.1	G	A	nonsynonymous SNV	G376A	E126K	M16	T	B	4.51
10	24762302	24762302	KIAA1217	KIAA1217	NM_019590.4	C	T	nonsynonymous SNV	C992T	P331L	M16	D	D	5.74
10	30315955	30315955	KIAA1462	KIAA1462	NM_020848	C	T	nonsynonymous SNV	G3122A	R1041Q	M16	T	B	1.85
10	31810176	31810176	ZEB1	zinc finger E-box binding hom	NM_001128128.2	C	T	nonsynonymous SNV	C1865T	S622L	M16	T	B	4.41
10	37506705	37506705	ANKRD30A	ANKRD30A	NM_052997	G	A	nonsynonymous SNV	G2998A	E1000K	M16	D	B	1.9
10	43089641	43089641	ZNF33B	zinc finger protein 33B	NM_006955	C	T	nonsynonymous SNV	G757A	G253R	M16	D	B	-3.62
10	44112386	44112386	ZNF485	zinc finger protein 485	NM_145312	C	T	stopgain	C895T	Q299X	M16	T		1.27
10	48387897	48387897	RBP3	retinol binding protein 3, inter	NM_002900	C	A	nonsynonymous SNV	G2981T	G994V	M16	D	P	4.66
10	48428871	48428871	GDF10	growth differentiation factor 1	NM_004962	C	T	nonsynonymous SNV	G1015A	D339N	M16	T	B	5.3
10	61833782	61833782	ANK3	ankyrin 3, node of Ranvier (a	NM_020987	G	A	nonsynonymous SNV	C6857T	P2286L	M16	D	D	5.92
10	61834035	61834035	ANK3	ankyrin 3, node of Ranvier (a	NM_020987	C	T	nonsynonymous SNV	G6604A	E2202K	M16	D	B	6.05
10	61834036	61834036	ANK3	ankyrin 3, node of Ranvier (a	NM_020987	C	T	nonsynonymous SNV	G6603A	M2201I	M16	T	B	4.16
10	61967957	61967957	ANK3	ankyrin 3, node of Ranvier (a	NM_020987	C	T	nonsynonymous SNV	G1031A	G344E	M16	D	D	4.78
10	68526075	68526075	CTNNA3	catenin (cadherin-associated	NM_013266.3	C	T	nonsynonymous SNV	G1228A	E410K	M16	T	D	6.17
10	70405014	70405014	TET1	tet methylcytosine dioxygena	NM_030625	T	A	nonsynonymous SNV	T2528A	I843K	M16	D	B	-0.647
10	73121791	73121791	SLC29A3	solute carrier family 29 (equil	NM_018344	C	T	nonsynonymous SNV	C854T	S285L	M16	T	B	-3.01
10	78778807	78778807	KCNMA1	potassium channel, calcium a	NM_001014797.2	C	T	nonsynonymous SNV	G1991A	G664E	M16	D	D	5.49
10	81929029	81929029	ANXA11	annexin A11	NM_001157.2	G	A	nonsynonymous SNV	C257T	P86L	M16	T	B	3.05
10	81929030	81929030	ANXA11	annexin A11	NM_001157.2	G	A	nonsynonymous SNV	C256T	P86S	M16	T	B	4.9
10	82298121	82298121	SH2D4B	SH2 domain containing 4B	NM_207372	G	T	nonsynonymous SNV	G34T	D12Y	M16	D	D	5.53
10	89690811	89690811	PTEN	phosphatase and tensin hom	NM_000314	A	T	nonsynonymous SNV	A218T	E73V	M16		D	5.62
10	90524299	90524299	LIPN	lipase, family member N	NM_001102469	G	A	nonsynonymous SNV	G359A	R120Q	M16	D	D	4.52
10	96580331	96580331	CYP2C19	cytochrome P450, family 2, s	NM_000769	G	A	nonsynonymous SNV	G898A	E300K	M16	D	D	3.95

10	96708988	96708988	CYP2C9	cytochrome P450, family 2, s	NM_000771	G	A	nonsynonymous SNV	G766A	D256N	M16	D	B	2.38
11	1016406	1016406	MUC6	mucin 6, oligomeric mucus/ge	NM_005961	G	A	nonsynonymous SNV	C6395T	S2132F	M16	D	B	0.796
11	123909690	123909690	OR10G7	olfactory receptor, family 10,	NM_001004463	G	C	nonsynonymous SNV	C19G	L7V	M16			
11	134244947	134244947	GLB1L2	galactosidase, beta 1-like 2	NM_138342	A	G	nonsynonymous SNV	A1906G	K636E	M16	T	B	-8.68
11	134244948	134244948	GLB1L2	galactosidase, beta 1-like 2	NM_138342	A	G	nonsynonymous SNV	A1907G	K636R	M16	D	B	4.17
11	1908086	1908086	LSP1	lymphocyte-specific protein 1	NM_001242932.1	C	T	nonsynonymous SNV	C1226T	P409L	M16	D	P	3.3
11	19259595	19259595	E2F8	E2F transcription factor 8	NM_024680.3	C	T	nonsynonymous SNV	G100A	E34K	M16	D	B	5.05
11	22257764	22257764	ANO5	anoctamin 5	NM_213599.2	T	C	nonsynonymous SNV	T704C	F235S	M16	T	B	5.77
11	30033267	30033267	KCNA4	potassium channel, voltage g	NM_002233	G	A	nonsynonymous SNV	C959T	S320F	M16	D	D	5.16
11	36614527	36614527	RAG2	recombination activating gene	NM_000536.3	C	T	nonsynonymous SNV	G1192A	E398K	M16	D	P	4.53
11	44253940	44253940	EXT2	exostosin glycosyltransferase	NM_000401.3	C	T	nonsynonymous SNV	C1799T	P600L	M16	T	D	5.16
11	4608285	4608285	OR52I2	olfactory receptor, family 52,	NM_001005170	G	A	nonsynonymous SNV	G243A	M81I	M16	T	B	3.15
11	46404254	46404254	MDK	midkine (neurite growth-prom	NM_001012334.2	G	T	nonsynonymous SNV	G362T	R121L	M16	T	b	3.84
11	46748083	46748083	F2	coagulation factor II (thrombin	NM_000506	G	A	nonsynonymous SNV	G910A	D304N	M16	D	B	2
11	46896462	46896462	LRP4	low density lipoprotein recept	NM_002334	T	C	nonsynonymous SNV	A4118G	D1373G	M16	D	P	5.67
11	4790462	4790462	OR51F1	olfactory receptor, family 51,	NM_001004752	C	T	nonsynonymous SNV	G686A	R229K	M16	T	B	-2.13
11	48177364	48177364	PTPRJ	protein tyrosine phosphatase	NM_002843	C	T	nonsynonymous SNV	C3241T	R1081C	M16	D	D	5.65
11	4945302	4945302	OR51G1	olfactory receptor, family 51,	NM_001005237	C	T	nonsynonymous SNV	G268A	D90N	M16	T	B	3.26
11	50003617	50003617	OR4C12	olfactory receptor, family 4, su	NM_001005270	G	A	nonsynonymous SNV	C421T	L141F	M16	T	B	2.98
11	55111078	55111078	OR4A16	olfactory receptor, family 4, su	NM_001005274	G	A	nonsynonymous SNV	G402A	M134I	M16	D	B	2.69
11	55339891	55339891	OR4C16	olfactory receptor, family 4, su	NM_001004701	G	A	nonsynonymous SNV	G288A	M96I	M16	T	B	-7.33
11	55418495	55418495	OR4S2	olfactory receptor, family 4, su	NM_001004059	G	A	nonsynonymous SNV	G116A	G39E	M16	D	D	5.36
11	55681275	55681275	OR5W2	olfactory receptor, family 5, su	NM_001001960	G	A	nonsynonymous SNV	C784T	P262S	M16	D	P	5.01
11	57995447	57995447	OR10Q1	olfactory receptor, family 10,	NM_001004471	C	T	nonsynonymous SNV	G901A	D301N	M16	D	D	5.07
11	62189842	62189842	SCGB1A1	secretoglobin, family 1A, mer	NM_003357	C	T	nonsynonymous SNV	C205T	L69F	M16	D	P	1.53
11	64811819	64811819	SAC3D1	SAC3 domain containing 1	NM_013299	C	T	nonsynonymous SNV	C697T	R233C	M16	D	D	4.4
11	66192028	66192028	NPAS4	neuronal PAS domain protein	NM_178864	A	G	nonsynonymous SNV	A1667G	N556S	M16	T	B	1.09
11	68475844	68475844	MTL5	metallothionein-like 5, testis-s	NM_004923	C	T	nonsynonymous SNV	G1459A	E487K	M16	D	D	5.32
11	6964819	6964819	ZNF215	zinc finger protein 215	NM_013250	G	T	nonsynonymous SNV	G659T	S220I	M16	T	B	-2.36
11	74914466	74914466	SLCO2B1	solute carrier organic anion tr	NM_007256.4	G	A	nonsynonymous SNV	G1943A	R648Q	M16	D	D	5.24
11	78467965	78467965	TENM4	teneurin transmembrane prot	NM_001098816	C	T	nonsynonymous SNV	G2641A	E881K	M16		D	4.22
11	7960352	7960352	OR10A3	olfactory receptor, family 10,	NM_001003745	G	A	nonsynonymous SNV	C716T	S239F	M16	D	D	4.74
11	94758846	94758846	KDM4E	lysine (K)-specific demethylas	NM_001161630	A	G	nonsynonymous SNV	A125G	Q42R	M16	T	B	0.198
12	10772847	10772847	STYK1	serine/threonine/tyrosine kina	NM_018423	C	T	nonsynonymous SNV	G1165A	D389N	M16	T	D	4.96
12	10954619	10954619	TAS2R7	taste receptor, type 2, membe	NM_023919	A	T	stopgain	T551A	L184X	M16	T		3.02
12	11420912	11420912	PRB3	proline-rich protein BstNI sub	NM_006249	C	T	nonsynonymous SNV	G271A	G91S	M16	T	P	-0.531
12	11461786	11461786	PRB4	proline-rich protein BstNI sub	NM_002723.4	C	T	nonsynonymous SNV	G131A	G44E	M16	D	D	0.769
12	122691349	122691349	B3GNT4	UDP-GlcNAc:betaGal beta-1,	NM_030765	G	A	stopgain	G551A	W184X	M16	T		5.2
12	124350615	124350615	DNAH10	dynein, axonemal, heavy cha	NM_207437	A	T	nonsynonymous SNV	A6808T	N2270Y	M16	D	B	4.81
12	14834382	14834382	GUCY2C	guanylate cyclase 2C	NM_004963	G	A	nonsynonymous SNV	C641T	S214F	M16	D	P	5.78
12	15095599	15095599	ARHGDIB	Rho GDP dissociation inhibito	NM_001175	A	G	nonsynonymous SNV	T463C	F155L	M16	T	P	4.41
12	21069050	21069050	SLCO1B3	solute carrier organic anion tr	NM_019844.3	G	A	nonsynonymous SNV	G1978A	D660N	M16	T	B	1.62
12	22025564	22025564	ABCC9	ATP-binding cassette, sub-fa	NM_005691.3	C	T	stopgain	G2193A	W731X	M16	T		5.48
12	4383387	4383387	CCND2	cyclin D2	NM_001759	A	C	nonsynonymous SNV	A181C	T61P	M16	T	B	4.17
12	48866827	48866827	ANP32D	acidic (leucine-rich) nuclear p	NM_012404	A	G	nonsynonymous SNV	A380G	N127S	M16	T	B	0.115
12	5020986	5020986	KCNA1	potassium channel, voltage g	NM_000217	G	A	nonsynonymous SNV	G442A	E148K	M16	T	B	4.35
12	52714927	52714927	KRT83	keratin 83, type II	NM_002282	C	T	nonsynonymous SNV	G193A	G65R	M16	T	B	3.8
12	52910916	52910916	KRT5	keratin 5, type II	NM_000424	G	A	nonsynonymous SNV	C1193T	A398V	M16	D	B	4.8
12	55420987	55420987	NEUROD4	neuronal differentiation 4	NM_021191	C	T	nonsynonymous SNV	C764T	P255L	M16	D	D	5.85
12	55820189	55820189	OR6C76	olfactory receptor, family 6, su	NM_001005183	C	T	nonsynonymous SNV	C152T	S51F	M16	T	B	4.35
12	57648645	57648645	R3HDM2	R3H domain containing 2	NM_014925	G	A	nonsynonymous SNV	C2842T	R948C	M16	D	P	5.32
12	6105299	6105299	VWF	von Willebrand factor	NM_000552	G	A	stopgain	C5932T	Q1978X	M16	T		3.63
12	6435679	6435679	PLEKHG6	pleckstrin homology domain c	NM_018173.3	C	T	nonsynonymous SNV	C1610T	S537F	M16	D	P	0.662
12	66700185	66700185	HELB	helicase (DNA) B	NM_033647	T	C	nonsynonymous SNV	T668C	V223A	M16		B	3.53
12	75436128	75436128	KCNC2	potassium channel, voltage g	NM_139136	C	T	nonsynonymous SNV	G1807A	E118D	M16	T	B	5.87
12	76788484	76788484	OSBPL8	oxysterol binding protein-like	NM_020841.4	G	A	nonsynonymous SNV	C698T	E118D	M16	D	D	3.7
12	80982053	80982053	PTPRQ	protein tyrosine phosphatase	NM_001145026	G	A	stopgain	G3915A	W1305X	M16			5.35
12	81067024	81067024	PTPRQ	protein tyrosine phosphatase	NM_001145026	G	A	nonsynonymous SNV	G6163A	D2055N	M16	D	D	5.78
13	114434293	114434293	GRK1	G protein-coupled receptor ki	NM_002929	C	T	nonsynonymous SNV	C1171T	P391S	M16	D	P	3.64
13	19997242	19997242	TPTE2	transmembrane phosphoinos	NM_199254.2	G	A	nonsynonymous SNV	C1529T	P510L	M16	0	B	1.08
13	29608058	29608058	MTUS2	microtubule associated tumor	NM_001033602	C	A	nonsynonymous SNV	C2272A	P758T	M16		D	4.61
13	35632872	35632872	NBEA	neurobeachin	NM_015678	C	T	nonsynonymous SNV	C1111T	L371F	M16	D	D	5.43
13	41766983	41766983	KBTBD7	kelch repeat and BTB (POZ)	NM_032138	G	A	nonsynonymous SNV	C1411T	P471S	M16	T	D	5.5
13	46946138	46946138	KIAA0226L	KIAA0226-like	NM_025113.3	G	A	nonsynonymous SNV	C473T	P158L	M16	T	B	3.96
13	51935422	51935422	SERPINE3	serpin peptidase inhibitor, cla	NM_001101320	G	A	nonsynonymous SNV	G1009A	G337S	M16	D	B	2.9
13	60435628	60435628	DIAPH3	diaphanous-related formin 3	NM_001042517.1	G	A	nonsynonymous SNV	C2650T	H884Y	M16	D	D	5.38
14	20665925	20665925	OR11G2	olfactory receptor, family 11, s	NM_001005503	C	T	nonsynonymous SNV	C431T	S144F	M16	T	D	4.93

14	20692716	20692716	OR11H6	olfactory receptor, family 11, s	NM_001004480	G	A	nonsynonymous SNV	G848A	G283E	M16	T	B	3.14
14	21992054	21992054	SALL2	spalt-like transcription factor 2	NM_005407.2	C	T	nonsynonymous SNV	G1808A	G603E	M16	T	B	3.88
14	21993446	21993446	SALL2	spalt-like transcription factor 2	NM_005407.2	C	G	nonsynonymous SNV	G416C	G139A	M16	T	B	4.22
14	23855298	23855298	MYH6	myosin, heavy chain 6, cardia	NM_002471	C	T	nonsynonymous SNV	G5002A	D1668N	M16	D	D	4.05
14	35245502	35245502	BAZ1A	bromodomain adjacent to zin	NM_013448.2	G	A	nonsynonymous SNV	C2456T	S819F	M16	D	D	5.75
14	38060761	38060761	FOXA1	forkhead box A1	NM_004496	C	T	nonsynonymous SNV	G1228A	E410K	M16	D	D	4.29
14	52899209	52899209	TXNDC16	thioredoxin domain containing	NM_020784.2	A	G	nonsynonymous SNV	T2291C	V764A	M16	T	B	-7.87
14	61180403	61180403	SIX4	SIX homeobox 4	NM_017420	G	A	nonsynonymous SNV	C2068T	P690S	M16	T	B	3.78
14	63174495	63174495	KCNH5	potassium channel, voltage g	NM_139318	G	A	nonsynonymous SNV	C2698T	P900S	M16	T	B	5.44
14	65253298	65253298	SPTB	spectrin, beta, erythrocytic	NM_001024858.2	G	A	stopgain	C3385T	Q1129X	M16	T		3.02
14	70634091	70634091	SLC8A3	solute carrier family 8 (sodiur	NM_183002.2	C	T	nonsynonymous SNV	G1049A	S350N	M16	D	D	5.85
14	75514207	75514207	MLH3	mutL homolog 3	NM_001040108.1	G	A	nonsynonymous SNV	C2152T	P718S	M16	T	B	2.62
14	75991472	75991472	BATF	basic leucine zipper transcrip	NM_006399	C	T	nonsynonymous SNV	C109T	R37C	M16	D	D	5.71
14	92949171	92949171	SLC24A4	solute carrier family 24 (sodiu	NM_153646.3	C	T	nonsynonymous SNV	C1403T	S468F	M16	D	D	5.74
14	94526808	94526808	DDX24	DEAD (Asp-Glu-Ala-Asp) box	NM_020414	G	A	nonsynonymous SNV	C1549T	P517S	M16	T	B	5.36
14	94752521	94752521	SERPINA10	serpin peptidase inhibitor, cla	NM_016186.2	C	T	nonsynonymous SNV	G1067A	G356E	M16	D	D	4.45
14	94964692	94964692	SERPINA12	serpin peptidase inhibitor, cla	NM_173850	G	A	nonsynonymous SNV	C43T	L15F	M16	T	P	3.55
14	95670076	95670076	CLMN	calmin (calponin-like, transme	NM_024734	G	A	nonsynonymous SNV	C1610T	S537F	M16	D	P	4.33
15	101569214	101569214	LRRK1	leucine-rich repeat kinase 1	NM_024652	C	T	nonsynonymous SNV	C2740T	H914Y	M16	T	P	5.12
15	33925277	33925277	RYR3	ryanodine receptor 3	NM_001036.4	A	G	nonsynonymous SNV	A2995G	I999V	M16	T	D	3.06
15	40093784	40093784	GPR176	G protein-coupled receptor 17	NM_007223.2	C	T	nonsynonymous SNV	G1097A	G366D	M16	D	D	6.17
15	42373759	42373759	PLA2G4D	phospholipase A2, group IVD	NM_178034	G	A	nonsynonymous SNV	C875T	A292V	M16	T	P	3.67
15	43622033	43622033	LCMT2	leucine carboxyl methyltransf	NM_014793	C	T	nonsynonymous SNV	G655A	E219K	M16	D	D	5.39
15	45059486	45059486	TRIM69	tripartite motif containing 69	NM_182985.4	A	G	nonsynonymous SNV	A1019G	K340R	M16	D	B	5.5
15	48595048	48595048	SLC12A1	solute carrier family 12 (sodiu	NM_000338.2	G	A	nonsynonymous SNV	G3266A	G1089E	M16	D	D	5.86
15	49917531	49917531	DTWD1	DTW domain containing 1	NM_020234.5	C	T	nonsynonymous SNV	C167T	S56L	M16		B	5.09
15	57999064	57999064	POLR2M	polymerase (RNA) II (DNA di	NM_015532.4	C	A	nonsynonymous SNV	C24A	F8L	M16	T	B	4.67
15	60643445	60643445	ANXA2	annexin A2	NM_001002858.2	A	G	nonsynonymous SNV	T838C	C280R	M16	D	D	4.3
15	65234706	65234706	ANKDD1A	ankyrin repeat and death don	NM_182703	C	T	nonsynonymous SNV	C910T	P304S	M16	T	D	3.95
15	65234707	65234707	ANKDD1A	ankyrin repeat and death don	NM_182703	C	T	nonsynonymous SNV	C911T	P304L	M16	T	P	4.87
15	69560649	69560649	GLCE	glucuronic acid epimerase	NM_015554	C	T	nonsynonymous SNV	C920T	S307F	M16	D	P	5.6
15	75042710	75042710	CYP1A2	cytochrome P450, family 1, s	NM_000761	G	A	nonsynonymous SNV	G631A	E211K	M16	D	B	0.232
15	75660469	75660469	MAN2C1	mannosidase, alpha, class 2C	NM_006715.3	C	T	nonsynonymous SNV	G172A	E58K	M16	T	B	0.723
15	79051801	79051801	ADAMTS7	ADAM metallopeptidase with	NM_014272	C	A	nonsynonymous SNV	G5023T	A1675S	M16	T	B	0.947
15	79051846	79051846	ADAMTS7	ADAM metallopeptidase with	NM_014272	T	C	nonsynonymous SNV	A4978G	I1660V	M16	T	B	-0.818
15	79058951	79058951	ADAMTS7	ADAM metallopeptidase with	NM_014272	T	C	nonsynonymous SNV	A3302G	H1101R	M16	T	B	-2.73
15	80762683	80762683	ARNT2	aryl-hydrocarbon receptor nu	NM_014862	C	T	nonsynonymous SNV	C319T	R107C	M16	D	D	5
15	81631768	81631768	TMC3	transmembrane channel-like	NM_001080532	C	T	nonsynonymous SNV	G1922A	R641Q	M16	T	B	2.9
15	82512139	82512139	EFTUD1	elongation factor Tu GTP bin	NM_024580.5	T	A	nonsynonymous SNV	A1465T	S489C	M16	D	P	3.12
15	89400401	89400401	ACAN	aggrecan	NM_013227.3	C	T	nonsynonymous SNV	C4585T	P1529S	M16	T	D	2.38
15	89400818	89400818	ACAN	aggrecan	NM_013227.3	G	A	nonsynonymous SNV	G5002A	E1668K	M16	D	D	5.86
15	89416190	89416190	ACAN	aggrecan	NM_013227.3	G	A	nonsynonymous SNV	G7267A	E2423K	M16	D	D	5.26
15	90764695	90764695	SEMA4B	sema domain, immunoglobul	NM_020210.3	C	T	nonsynonymous SNV	C692T	S231F	M16	T	P	5.46
15	91292685	91292685	BLM	Bloom syndrome, RecQ helic	NM_000057.3	A	G	nonsynonymous SNV	A187G	K63E	M16	D	B	4.65
15	91527990	91527990	PRC1	protein regulator of cytokines	NM_003981	C	T	stopgain	G77A	W26X	M16	T		5.65
15	93485138	93485138	CHD2	chromodomain helicase DNA	NM_001271	G	A	nonsynonymous SNV	G779A	S260N	M16	T	B	5.37
16	18880555	18880555	SMG1	SMG1 phosphatidylinositol 3-	NM_015092	G	A	nonsynonymous SNV	C2704T	P902S	M16	T	D	5.22
16	2031176	2031176	NOXO1	NADPH oxidase organizer 1	NM_144603.3	G	T	nonsynonymous SNV	C5A	A2E	M16	T	B	1.47
16	20492161	20492161	ACSM2A	acyl-CoA synthetase medium	NM_001010845	C	T	nonsynonymous SNV	C1427T	S476L	M16	D	P	2.17
16	21049192	21049192	DNAH3	dynein, axonemal, heavy cha	NM_017539	C	T	nonsynonymous SNV	G4841A	G1614E	M16	D	D	5.76
16	22926708	22926708	HS3ST2	heparan sulfate (glucosamine	NM_006043	G	A	nonsynonymous SNV	G929A	G310E	M16	D	D	5.11
16	24895381	24895381	SLC5A11	solute carrier family 5 (sodiur	NM_052944.4	C	T	nonsynonymous SNV	C593T	A198V	M16	T	P	5.11
16	28944232	28944232	CD19	CD19 molecule	NM_001178098.1	G	A	nonsynonymous SNV	G356A	G119E	M16		B	3.71
16	420004	420004	MRPL28	mitochondrial ribosomal prote	NM_006428	G	A	nonsynonymous SNV	C215T	P72L	M16	T	D	3.89
16	46940844	46940844	GPT2	glutamic pyruvate transamina	NM_133443.3	C	T	nonsynonymous SNV	C533T	P178L	M16	T	B	4.8
16	55727927	55727927	SLC6A2	solute carrier family 6 (neurot	NM_001172504.1	G	A	stopgain	G924A	W308X	M16	T	O	4.57
16	55862775	55862775	CES1	carboxylesterase 1	NM_001025195.1	G	A	nonsynonymous SNV	C164T	P55L	M16	D	D	4.48
16	58585615	58585615	CNOT1	CCR4-NOT transcription com	NM_016284.4	G	A	nonsynonymous SNV	C3079T	P1027S	M16	T	B	5.41
16	67405015	67405015	LRRC36	leucine rich repeat containing	NM_018296.5	G	A	nonsynonymous SNV	G1364A	G455E	M16	T	P	2.67
16	88503168	88503168	ZNF469	zinc finger protein 469	NM_001127464	G	A	nonsynonymous SNV	G9206A	G3069E	M16	T	B	-3.57
17	10247210	10247210	MYH13	myosin, heavy chain 13, skel	NM_003802	C	T	nonsynonymous SNV	G1801A	D601N	M16	D	D	4.33
17	12666536	12666536	MYOCD	myocardin	NM_001146312.2	C	T	nonsynonymous SNV	C2536T	P846S	M16	T	B	6.08
17	18023016	18023016	MYO15A	myosin XVA	NM_016239	C	T	nonsynonymous SNV	C902T	P301L	M16	T	B	3.51
17	26092620	26092620	NOS2	nitric oxide synthase 2, induc	NM_000625	C	A	nonsynonymous SNV	G2369T	R790L	M16	D	P	5.19
17	26943509	26943509	KIAA0100	KIAA0100	NM_014680	G	A	nonsynonymous SNV	C6176T	P2059L	M16	T	B	6.11
17	27215999	27215999	FLOT2	flotillin 2	NM_004475	C	T	stopgain	G95A	W32X	M16	T		5.8
17	29654691	29654691	NF1	neurofibromin 1	NM_001042492	C	T	stopgain	C5443T	Q1815X	M16	T		5.99

17	33690337	33690337	SLFN11	schlafen family member 11	NM_001104587.1	C	T	nonsynonymous SNV	G490A	G164R	M16	T	B	-5.25
17	39525935	39525935	KRT33B	keratin 33B, type I	NM_002279	G	A	nonsynonymous SNV	C68T	P23L	M16	T	B	4.44
17	39643373	39643373	KRT36	keratin 36, type I	NM_003771	G	A	nonsynonymous SNV	C1037T	S346F	M16	D	D	4.96
17	39780517	39780517	KRT17	keratin 17, type I	NM_000422	C	T	nonsynonymous SNV	G245A	G82E	M16	D	P	4.25
17	40831669	40831669	CCR10	chemokine (C-C motif) recept	NM_016602	G	A	nonsynonymous SNV	C991T	P331S	M16	T	B	-4.01
17	42335497	42335497	SLC4A1	solute carrier family 4 (anion	NM_000342	C	T	nonsynonymous SNV	G1139A	G380E	M16	D	D	4.82
17	42426834	42426834	GRN	granulin	NM_002087	C	T	nonsynonymous SNV	C179T	P60L	M16	T	B	2.54
17	42828203	42828203	DBF4B	DBF4 zinc finger B	NM_145663	C	T	nonsynonymous SNV	C1430T	P477L	M16		B	-5
17	43313510	43313510	FMNL1	formin-like 1	NM_005892	C	A	nonsynonymous SNV	C622A	P208T	M16	T	B	2.94
17	44039785	44039785	MAPT	microtubule-associated prote	NM_001123066.3	G	A	nonsynonymous SNV	G82A	G28S	M16	D	B	2.8
17	46628262	46628262	HOXB3	homeobox B3	NM_002146	T	C	nonsynonymous SNV	A730G	K244E	M16	D	B	3.66
17	47122365	47122365	IGF2BP1	insulin-like growth factor 2 ml	NM_006546.3	G	A	nonsynonymous SNV	G1333A	E445K	M16	T	D	5.01
17	47247037	47247037	B4GALNT2	beta-1,4-N-acetyl-galactosam	NM_153446.2	C	T	stopgain	C1648T	Q550X	M16	T		2.41
17	48186067	48186067	PDK2	pyruvate dehydrogenase kina	NM_002611.4	C	T	nonsynonymous SNV	C953T	T318I	M16	D	B	3.94
17	48271805	48271805	COL1A1	collagen, type I, alpha 1	NM_000088	G	A	nonsynonymous SNV	C1519T	P507S	M16	T	B	2.61
17	48653174	48653174	CACNA1G	calcium channel, voltage-dep	NM_018896.4	C	T	nonsynonymous SNV	C1411T	L471F	M16	T	B	3.16
17	48918243	48918243	WFIKKN2	WAP, follistatin/kazal, immun	NM_175575	G	A	nonsynonymous SNV	G1594A	E532K	M16	T	B	5.12
17	5076229	5076229	USP6	ubiquitin specific peptidase 6	NM_004505	G	A	nonsynonymous SNV	G4177A	E1393K	M16	D	D	2.35
17	54969098	54969098	TRIM25	tripartite motif containing 25	NM_005082	G	A	nonsynonymous SNV	C1856T	S619F	M16	D	D	4.87
17	56383766	56383766	BZRAP1	benzodiazepine receptor (per	NM_004758.3	G	A	nonsynonymous SNV	C5161T	P1721S	M16	T	B	1.8
17	56386068	56386068	BZRAP1	benzodiazepine receptor (per	NM_004758.3	G	A	nonsynonymous SNV	C4565T	P1522L	M16	T	B	2.19
17	56386069	56386069	BZRAP1	benzodiazepine receptor (per	NM_004758.3	G	A	nonsynonymous SNV	C4564T	P1522S	M16	T	B	3.36
17	65052241	65052241	CACNG1	calcium channel, voltage-dep	NM_000727	G	A	nonsynonymous SNV	G523A	E175K	M16	T	B	0.276
17	65346383	65346383	PSMD12	proteasome (prosome, macro	NM_002816.3	G	A	stopgain	C367T	R123X	M16	T		3.87
17	66891080	66891080	ABCA8	ATP-binding cassette, sub-fa	NM_001288985.1	T	C	nonsynonymous SNV	A2839G	R123X	M16	T	D	3.73
17	73832722	73832722	UNC13D	unc-13 homolog D (C. elegar	NM_199242	A	G	nonsynonymous SNV	T1229C	I410T	M16	D	P	3.25
17	73843907	73843907	WBP2	WW domain binding protein 2	NM_012478	G	A	nonsynonymous SNV	C502T	P168S	M16	T	D	3.58
17	74394602	74394602	UBE2O	ubiquitin-conjugating enzyme	NM_022066	G	A	nonsynonymous SNV	C1847T	T616I	M16	T	D	5.42
17	76481017	76481017	DNAH17	dynein, axonemal, heavy cha	NM_173628	C	T	nonsynonymous SNV	G7582A	E2528K	M16	D		4.96
18	10763085	10763085	PIEZO2	piezo-type mechanosensitive	NM_022068	G	T	nonsynonymous SNV	C2883A	R171C	M16	T	B	5.23
18	24497022	24497022	CHST9	carbohydrate (N-acetylgalact	NM_031422	C	T	nonsynonymous SNV	G533A	R178Q	M16	D	D	6.16
18	31324974	31324974	ASXL3	additional sex combs like trar	NM_030632	C	A	nonsynonymous SNV	C5162A	S1721Y	M16	D	D	5.86
18	32833686	32833686	ZSCAN30	zinc finger and SCAN domair	NM_001166012.2	T	C	nonsynonymous SNV	A1213G	K405E	M16	D	P	4.19
18	43219847	43219847	SLC14A2	solute carrier family 14 (urea	NM_007163.3	G	A	nonsynonymous SNV	G980A	G327E	M16	D	B	4.37
18	70461449	70461449	NETO1	neuropilin (NRP) and tolloid (	NM_138966	C	T	nonsynonymous SNV	G542A	G181E	M16	D	D	5.29
19	10080357	10080357	COL5A3	collagen, type V, alpha 3	NM_015719	C	T	nonsynonymous SNV	G3992A	G1331E	M16	D	D	3.76
19	12186819	12186819	ZNF844	zinc finger protein 844	NM_001136501	C	T	nonsynonymous SNV	C884T	E652D	M16	T	B	-5
19	14992110	14992110	OR7A17	olfactory receptor, family 7, s	NM_030901	C	T	nonsynonymous SNV	G58A	E20K	M16	T	B	1.67
19	15198817	15198817	OR1I1	olfactory receptor, family 1, s	NM_001004713	G	A	nonsynonymous SNV	G941A	R314K	M16	T	B	0.032
19	20727663	20727663	ZNF737	zinc finger protein 737	NM_001159293	C	T	nonsynonymous SNV	G1346A	G449E	M16	T	B	0.801
19	20807247	20807247	ZNF626	zinc finger protein 626	NM_001076675	C	A	nonsynonymous SNV	G1436T	R479I	M16	D	B	-1.66
19	21366439	21366439	ZNF431	zinc finger protein 431	NM_133473	G	T	nonsynonymous SNV	G1333T	A445S	M16	T	B	-1.91
19	22942376	22942376	ZNF99	zinc finger protein 99	NM_001080409	C	T	nonsynonymous SNV	G335A	R112Q	M16	T	B	-1.87
19	22952066	22952066	ZNF99	zinc finger protein 99	NM_001080409	C	T	nonsynonymous SNV	G64A	D22N	M16	T	P	1.05
19	23927898	23927898	ZNF681	zinc finger protein 681	NM_138286	G	A	nonsynonymous SNV	C454T	H152Y	M16	T	B	-2.54
19	3008922	3008922	TLE2	transducin-like enhancer of s	NM_001300846.1	G	A	nonsynonymous SNV	C1198T	H400Y	M16	T	D	2.95
19	30934951	30934951	ZNF536	zinc finger protein 536	NM_014717	C	T	nonsynonymous SNV	C482T	P161L	M16	D	D	4.63
19	31769138	31769138	TSHZ3	teashirt zinc finger homeobox	NM_020856	C	T	nonsynonymous SNV	G1561A	D521N	M16	D	P	4.37
19	35624952	35624952	LGI4	Leucine-rich repeat LGI famil	NM_139284	G	A	nonsynonymous SNV	C227T	P76L	M16	D	D	3.6
19	36387308	36387308	NFKBID	nuclear factor of kappa light p	NM_139239	G	A	nonsynonymous SNV	C391T	P131S	M16	T	D	3.77
19	38230622	38230622	ZNF573	zinc finger protein 573	NM_152360.3	G	A	stopgain	C595T	Q199X	M16	T		1.18
19	38378670	38378670	WDR87	WD repeat domain 87	NM_001291088.1	C	T	nonsynonymous SNV	G5641A	E1881K	M16	T	B	1.32
19	38379186	38379186	WDR87	WD repeat domain 87	NM_001291088.1	C	T	nonsynonymous SNV	G5125A	E1709K	M16	T	B	0.025
19	38379719	38379719	WDR87	WD repeat domain 87	NM_001291088.1	T	C	nonsynonymous SNV	A4592G	E1531G	M16	D	P	1.27
19	3941088	3941088	NMRK2	nicotinamide riboside kinase	NM_001289117.1	C	T	nonsynonymous SNV	C430T	P144S	M16	D	P	4.03
19	4212663	4212663	ANKRD24	ankyrin repeat domain 24	NM_133475	G	A	nonsynonymous SNV	G1165A	E389K	M16	D	D	5.04
19	43093831	43093831	CEACAM8	carcinoembryonic antigen-rel	NM_001816	C	T	nonsynonymous SNV	G481A	D161N	M16	D	D	1.52
19	43514178	43514178	PSG11	pregnancy specific beta-1-gly	NM_002785.2	C	T	nonsynonymous SNV	G980A	G327E	M16	T	D	-2.23
19	43519288	43519288	PSG11	pregnancy specific beta-1-gly	NM_002785.2	G	A	nonsynonymous SNV	C944T	S315F	M16	T	P	-1.95
19	43762564	43762564	PSG9	pregnancy specific beta-1-gly	NM_002784.4	G	A	nonsynonymous SNV	C1033T	R345C	M16	T	D	1.31
19	43771969	43771969	PSG9	pregnancy specific beta-1-gly	NM_002784.4	C	T	nonsynonymous SNV	G397A	E133K	M16	T	P	-3.11
19	44680298	44680298	ZNF226	zinc finger protein 226	NM_001032372.1	C	T	nonsynonymous SNV	C883T	L295F	M16	D	D	3.2
19	4512515	4512515	PLIN4	perilipin 4	NM_001080400	C	T	nonsynonymous SNV	G1415A	G472D	M16	T	B	-10.5
19	4652341	4652341	TNFAIP8L1	tumor necrosis factor, alpha-i	NM_001167942.1	G	A	nonsynonymous SNV	G460A	D154N	M16	T	P	4.44
19	50368655	50368655	PNKP	polynucleotide kinase 3'-phos	NM_007254	G	A	nonsynonymous SNV	C227T	T76I	M16	T	B	1.08
19	51645918	51645918	SIGLEC7	sialic acid binding Ig-like lecti	NM_014385.3	C	T	nonsynonymous SNV	C292T	L98F	M16	T	P	-1.16
19	52619546	52619546	ZNF616	zinc finger protein 616	NM_178523	G	A	nonsynonymous SNV	C871T	H291Y	M16	D	D	0.954

19	52660383	52660383	ZNF836	zinc finger protein 836	NM_001102657	G	A	nonsynonymous SNV	C553T	P185S	M16	T	D	-2.59
19	54803106	54803106	LILRA3	leukocyte immunoglobulin-like	NM_006865.4	G	A	nonsynonymous SNV	C571T	R191C	M16	T	B	-0.191
19	55994548	55994548	ZNF628	zinc finger protein 628	NM_033113	C	T	nonsynonymous SNV	C1988T	P663L	M16	T	B	0.908
19	56241280	56241280	NLRP9	NLR family, pyrin domain con	NM_176820	C	T	nonsynonymous SNV	G1911A	M637I	M16	T	B	1.22
19	56423937	56423937	NLRP13	NLR family, pyrin domain con	NM_176810	C	T	nonsynonymous SNV	G1246A	E416K	M16	T	D	2.7
19	56466958	56466958	NLRP8	NLR family, pyrin domain con	NM_176811	G	A	nonsynonymous SNV	G1534A	E512K	M16	D	D	2
19	56569723	56569723	NLRP5	NLR family, pyrin domain con	NM_153447	G	A	nonsynonymous SNV	G3417A	M1139I	M16	T	B	-0.238
19	56895705	56895705	ZNF582	zinc finger protein 582	NM_144690	G	A	nonsynonymous SNV	C1081T	H361Y	M16	D	D	4.49
19	57327898	57327898	PEG3	paternally expressed 3	NM_006210.2	G	A	nonsynonymous SNV	C1912T	H638Y	M16	T	D	3.07
19	5790155	5790155	DUS3L	dihydrouridine synthase 3-like	NM_020175	A	G	nonsynonymous SNV	T290C	L97P	M16	T	B	-7.82
19	58639694	58639694	ZNF329	zinc finger protein 329	NM_024620	G	A	nonsynonymous SNV	C1177T	H393Y	M16	D	D	4.31
19	8212238	8212238	FBN3	fibrillin 3	NM_032447	C	T	nonsynonymous SNV	G127A	G43R	M16	T	P	3.41
19	9048512	9048512	MUC16	mucin 16, cell surface associ	NM_024690	C	T	nonsynonymous SNV	G33119A	G11040E	M16	D	B	-5.35
19	9066247	9066247	MUC16	mucin 16, cell surface associ	NM_024690	G	A	nonsynonymous SNV	C21199T	P7067S	M16	D	B	-1.44
19	9074082	9074082	MUC16	mucin 16, cell surface associ	NM_024690	G	A	nonsynonymous SNV	C13364T	S4455F	M16	D	D	2.15
19	9720910	9720910	ZNF561	zinc finger protein 561	NM_152289	G	A	nonsynonymous SNV	C1427T	P476L	M16	D	D	1.1
2	109964145	109964145	SH3RF3	SH3 domain containing ring f	NM_001099289	C	T	nonsynonymous SNV	C589T	P197S	M16	D	P	3.06
2	111399737	111399737	BUB1	BUB1 mitotic checkpoint seri	NM_004336.4	C	T	nonsynonymous SNV	G2422A	D808N	M16	T	B	4.32
2	112615888	112615888	ANAPC1	anaphase promoting complex	NM_022662	C	G	nonsynonymous SNV	G1353C	Q451H	M16	T	B	3.2
2	116485465	116485465	DPP10	dipeptidyl-peptidase 10	NM_001178034.1	G	A	nonsynonymous SNV	G662A	G221E	M16	D	D	5.46
2	120194981	120194981	TMEM37	transmembrane protein 37	NM_183240	C	T	nonsynonymous SNV	C538T	L180F	M16	T	D	4.84
2	1204906	1204906	SNTG2	syntrophin, gamma 2	NM_018968	G	A	nonsynonymous SNV	G709A	E237K	M16	T	B	3.14
2	121746527	121746527	GLI2	GLI family zinc finger 2	NM_005270	C	T	nonsynonymous SNV	C3037T	P1013S	M16	T	B	-2.6
2	121746528	121746528	GLI2	GLI family zinc finger 2	NM_005270	C	T	nonsynonymous SNV	C3038T	P1013L	M16	T	B	0.584
2	130939113	130939113	SMPD4	sphingomyelin phosphodieste	NM_017951.4	C	T	stopgain	G62A	W21X	M16	T		-1.5
2	149857253	149857253	KIF5C	kinesin family member 5C	NM_004522	G	A	nonsynonymous SNV	G2330A	R777K	M16		B	5.01
2	153616337	153616337	ARL6IP6	ADP-ribosylation factor-like 6	NM_152522	G	T	nonsynonymous SNV	G664T	A222S	M16	D	P	5.4
2	166848243	166848243	SCN1A	sodium channel, voltage gate	NM_001165963.1	G	A	stopgain	C5542T	Q1848X	M16	T		5.6
2	167108394	167108394	SCN9A	sodium channel, voltage gate	NM_002977	C	T	nonsynonymous SNV	G3320A	R1107K	M16	T	B	3.03
2	167262362	167262362	SCN7A	sodium channel, voltage gate	NM_002976	C	T	nonsynonymous SNV	G4777A	E1593K	M16	D	P	4.51
2	167297944	167297944	SCN7A	sodium channel, voltage gate	NM_002976	G	A	nonsynonymous SNV	C2119T	P707S	M16	T	B	-3.9
2	168099701	168099701	XIRP2	xin actin binding repeat conta	NM_152381.5	C	T	nonsynonymous SNV	C1799T	S600F	M16	D	P	3.53
2	173352338	173352338	ITGA6	integrin, alpha 6	NM_001079818.1	C	T	nonsynonymous SNV	C2224T	P742S	M16	D	D	5.61
2	179594516	179594516	TTN	titin	NM_001267550	G	A	nonsynonymous SNV	C18464T	S6155F	M16	D	P	6.17
2	1921109	1921109	MYT1L	myelin transcription factor 1-l	NM_015025	G	A	nonsynonymous SNV	C1480T	P494S	M16	T	B	5.73
2	197183279	197183279	HECW2	HECT, C2 and WW domain c	NM_020760	C	A	stopgain	G2335T	G779X	M16	T		4.91
2	198949979	198949979	PLCL1	phospholipase C-like 1	NM_006226	C	T	stopgain	C1738T	R580X	M16			1.91
2	207424736	207424736	ADAM23	ADAM metallopeptidase dom	NM_003812	G	A	nonsynonymous SNV	G1063A	E355K	M16	T	B	5.64
2	212652869	212652869	ERBB4	erb-b2 receptor tyrosine kina	NM_005235.2	C	T	nonsynonymous SNV	G437A	G146E	M16	D	D	5.32
2	227954610	227954610	COL4A4	collagen, type IV, alpha 4	NM_000092	C	T	nonsynonymous SNV	G1433A	G478E	M16	D	B	4.86
2	234590724	234590724	UGT1A7	UDP glucuronosyltransferase	NM_019077	A	T	nonsynonymous SNV	A141T	K47N	M16	D	P	-3.81
2	234638421	234638421	UGT1A3	UDP glucuronosyltransferase	NM_019093	C	T	nonsynonymous SNV	C649T	P217S	M16	T	P	3.2
2	27276296	27276296	AGBL5	ATP/GTP binding protein-like	NM_021831.5	G	A	nonsynonymous SNV	G242A	G81E	M16	D	D	5.67
2	27447242	27447242	CAD	carbamoyl-phosphate synthe	NM_004341	C	G	nonsynonymous SNV	C1138G	P380A	M16	T	B	3.08
2	27447243	27447243	CAD	carbamoyl-phosphate synthe	NM_004341	C	T	nonsynonymous SNV	C1139T	P380L	M16	T	B	3.21
2	27479365	27479365	SLC30A3	solute carrier family 30 (zinc t	NM_003459	C	T	nonsynonymous SNV	G907A	E303K	M16	T	B	5.24
2	27552354	27552354	GTF3C2	general transcription factor III	NM_001521.3	G	A	nonsynonymous SNV	C1769T	P590L	M16	T	B	4.78
2	36706651	36706651	CRIM1	cysteine rich transmembrane	NM_016441	C	T	nonsynonymous SNV	C1186T	P396S	M16	T	P	5.34
2	39178194	39178194	ARHGEF33	Rho guanine nucleotide exch	NM_001145451	G	A	nonsynonymous SNV	G985A	E329K	M16	T	P	4.74
2	80874881	80874881	CTNNA2	catenin (cadherin-associated	NM_001282597.2	C	T	nonsynonymous SNV	C2746T	L916F	M16		D	4.89
2	87042840	87042840	CD8B	CD8b molecule	NM_172213.3	G	A	nonsynonymous SNV	C622T	P208S	M16	D	B	1.5
2	9474874	9474874	ASAP2	ArfGAP with SH3 domain, an	NM_003887.2	C	T	stopgain	C694T	Q232X	M16	T		5.12
2	99013185	99013185	CNGA3	cyclic nucleotide gated chann	NM_001298.2	G	A	nonsynonymous SNV	G1552A	E518K	M16	T	D	4.87
20	14307771	14307771	FLRT3	fibronectin leucine rich transn	NM_198391.2	G	A	nonsynonymous SNV	C382T	P128S	M16	T	B	6.06
20	1459144	1459144	SIRPB2	signal-regulatory protein beta	NM_001122962.1	G	A	nonsynonymous SNV	C560T	P187L	M16	D	D	4.05
20	1616878	1616878	SIRPG	signal-regulatory protein gam	NM_018556.3	C	T	nonsynonymous SNV	G704A	G235E	M16	T	B	0.764
20	19665971	19665971	SLC24A3	solute carrier family 24 (sodiu	NM_020689	T	G	nonsynonymous SNV	T1290G	D430E	M16		B	-4.89
20	32345053	32345053	ZNF341	zinc finger protein 341	NM_001282933.1	C	T	nonsynonymous SNV	C841T	P281S	M16	T	D	5.87
20	32345054	32345054	ZNF341	zinc finger protein 341	NM_001282933.1	C	T	nonsynonymous SNV	C842T	P281L	M16	D	D	5.87
20	33503040	33503040	ACSS2	acyl-CoA synthetase short-ch	NM_001076552	C	T	nonsynonymous SNV	C854T	S285F	M16	T	B	4.71
20	3649620	3649620	ADAM33	ADAM metallopeptidase dom	NM_025220.3	C	A	nonsynonymous SNV	G2432T	C811F	M16	T	B	-1.84
20	37195804	37195804	RALGAPB	Ral GTPase activating protei	NM_020336.3	A	T	nonsynonymous SNV	A3883T	I1295F	M16	T	B	5.42
20	3891387	3891387	PANK2	pantothenate kinase 2	NM_153640.2	C	T	nonsynonymous SNV	C272T	P91L	M16	T	B	4.48
20	40827885	40827885	PTPRT	protein tyrosine phosphatase	NM_133170.3	C	T	nonsynonymous SNV	G2543A	G848E	M16	D	D	5.73
20	43739287	43739287	WFDC5	WAP four-disulfide core doma	NM_145652	G	A	nonsynonymous SNV	C215T	P72L	M16	T	D	-0.708
20	44678295	44678295	SLC12A5	solute carrier family 12 (potas	NM_001134771.1	G	A	nonsynonymous SNV	G2116A	D706N	M16	T	B	4.35
20	52790066	52790066	CYP24A1	cytochrome P450, family 24,	NM_000782.4	A	C	nonsynonymous SNV	T53G	L18R	M16	D	B	4.16

20	57564976	57564976	NELFCD	negative elongation factor co|	NM_198976	C	T	stopgain	C775T	Q259X	M16	T		5.74
20	57568570	57568570	NELFCD	negative elongation factor co|	NM_198976	G	A	nonsynonymous SNV	G1465A	E489K	M16	T	B	5.32
20	62168628	62168628	PTK6	protein tyrosine kinase 6	NM_005975.3	C	A	nonsynonymous SNV	G40T	V14L	M16	D	B	1.43
20	9402007	9402007	PLCB4	phospholipase C, beta 4	NM_001172646.1	G	A	nonsynonymous SNV	G2218A	G740R	M16	D	D	5.68
20	9546620	9546620	PAK7	p21 protein (Cdc42/Rac)-acti	NM_020341.3	C	T	nonsynonymous SNV	G1402A	E468K	M16		D	5.5
20	9560902	9560902	PAK7	p21 protein (Cdc42/Rac)-acti	NM_020341.3	C	T	nonsynonymous SNV	G880A	E294K	M16		P	5.6
21	10908835	10908835	TPTE	transmembrane phosphatase	NM_199261.3	C	T	nonsynonymous SNV	G1510A	E504K	M16	T	B	-4.36
21	41033288	41033288	B3GALT5	UDP-Gal:betaGlcNAc beta 1,	NM_006057.2	C	T	nonsynonymous SNV	C802T	P268S	M16	T	P	5.64
21	45815319	45815319	TRPM2	transient receptor potential ca	NM_003307	C	T	nonsynonymous SNV	C1817T	S606F	M16	T	D	4.32
21	47359951	47359951	PCBP3	poly(rC) binding protein 3	NM_020528.2	G	T	nonsynonymous SNV	G917T	G306V	M16	D	D	4.19
21	47965801	47965801	DIP2A	disco-interacting protein 2 ho	NM_015151.3	C	T	nonsynonymous SNV	C2321T	P774L	M16	T	B	4.84
22	18138598	18138598	BCL2L13	BCL2-like 13 (apoptosis facili	NM_015367.3	G	A	nonsynonymous SNV	G121A	G41R	M16	T	D	5.68
22	19969479	19969479	ARVCF	armadillo repeat gene deletec	NM_001670	T	A	nonsynonymous SNV	A346T	T116S	M16		D	3.45
22	21987504	21987504	CCDC116	coiled-coil domain containing	NM_152612	G	T	nonsynonymous SNV	G34T	A12S	M16	D	D	4.03
22	26165178	26165178	MYO18B	myosin XVIIIB	NM_032608	C	T	nonsynonymous SNV	C1295T	P432L	M16	D	B	2.59
22	29650193	29650193	EMID1	EMI domain containing 1	NM_133455.3	G	A	nonsynonymous SNV	G1121A	G374E	M16	D	B	4.93
22	29687573	29687573	EWSR1	EWS RNA-binding protein 1	NM_013986.3	T	C	nonsynonymous SNV	T1012C	F338L	M16	D	D	5.78
22	30770056	30770056	CCDC157	coiled-coil domain containing	NM_001017437	A	T	nonsynonymous SNV	A1648T	R550W	M16	D	B	-3.61
22	30890857	30890857	SEC14L4	SEC14-like lipid binding 4	NM_174977.3	T	A	nonsynonymous SNV	A515T	Q172L	M16	T	B	5.11
22	32756350	32756350	RFPL3	ret finger protein-like 3	NM_001098535.1	G	A	nonsynonymous SNV	G485A	R162K	M16	D	D	0.664
22	34046465	34046465	LARGE	like-glycosyltransferase	NM_004737.4	G	A	nonsynonymous SNV	C296T	S99F	M16	T	B	4.57
22	40661348	40661348	TNRC6B	trinucleotide repeat containin	NM_001162501.1	G	A	nonsynonymous SNV	G1114A	G372R	M16	D	P	5.07
22	45931198	45931198	FBLN1	fibulin 1	NM_006486.2	T	G	nonsynonymous SNV	T903G	D301E	M16	T	P	3.22
22	50297497	50297497	ALG12	ALG12, alpha-1,6-mannosyltı	NM_024105	G	A	nonsynonymous SNV	C1456T	R486W	M16	D	B	-10.6
3	111997609	111997609	SLC9C1	solute carrier family 9, subfar	NM_183061	C	T	nonsynonymous SNV	G285A	M95I	M16	T	B	3.31
3	112644023	112644023	CD200R1	CD200 receptor 1	NM_138806.3	A	C	nonsynonymous SNV	T787G	M95I	M16	T	B	-8.32
3	122404136	122404136	PARP14	poly (ADP-ribose) polymerase	NM_017554	C	T	nonsynonymous SNV	C292T	H98Y	M16	D	B	0.867
3	132047168	132047168	ACPP	acid phosphatase, prostate	NM_001099.4	G	A	nonsynonymous SNV	G178A	E60K	M16	D	D	5.72
3	134078254	134078254	AMOTL2	angiomotin like 2	NM_001278683.1	G	A	stopgain	C2149T	Q717X	M16	T		5.54
3	138724404	138724404	PRR23A	proline rich 23A	NM_001134659	G	A	nonsynonymous SNV	C707T	P236L	M16	D	D	3.23
3	148923998	148923998	CP	ceruloplasmin (ferroxidase)	NM_000096	C	T	nonsynonymous SNV	G1165A	G389S	M16	T	P	5.81
3	150416580	150416580	ERICH6	glutamate-rich 6	NM_152394	G	A	nonsynonymous SNV	C551T	S184L	M16	T	B	-7.46
3	155314127	155314127	PLCH1	phospholipase C, eta 1	NM_001130960.1	C	T	nonsynonymous SNV	G84A	M28I	M16	D	P	5.42
3	167512485	167512485	SERPINI1	serpin peptidase inhibitor, cla	NM_005025.4	A	G	nonsynonymous SNV	A754G	S252G	M16	D	P	4.53
3	169639114	169639114	SAMD7	sterile alpha motif domain co	NM_182610	C	T	nonsynonymous SNV	C199T	R67W	M16	D	P	3.53
3	170244494	170244494	SLC7A14	solute carrier family 7, memb	NM_020949	C	T	nonsynonymous SNV	G232A	V78M	M16	D	D	4.71
3	172224307	172224307	TNFSF10	tumor necrosis factor (ligand)	NM_003810	A	T	nonsynonymous SNV	T821A	F274Y	M16	T	B	-0.206
3	175345091	175345091	NAALADL2	N-acetylated alpha-linked aci	NM_207015	C	T	nonsynonymous SNV	C1813T	L605F	M16	T	D	5.35
3	186459696	186459696	KNG1	kininogen 1	NM_001102416.2	G	A	nonsynonymous SNV	G1511A	G504E	M16		B	-1.9
3	195516064	195516064	MUC4	mucin 4, cell surface associat	NM_018406	C	T	nonsynonymous SNV	G2387A	R796Q	M16	T	B	-3.42
3	33907878	33907878	PDCD6IP	programmed cell death 6 inte	NM_001162429.2	C	A	nonsynonymous SNV	C2483A	P828H	M16	D	D	5.37
3	38835477	38835477	SCN10A	sodium channel, voltage gate	NM_006514.3	C	T	nonsynonymous SNV	G25A	E9K	M16	D	B	-1.65
3	38936362	38936362	SCN11A	sodium channel, voltage gate	NM_014139.2	G	A	stopgain	C2497T	Q833X	M16	D		5.38
3	38946740	38946740	SCN11A	sodium channel, voltage gate	NM_014139.2	G	A	nonsynonymous SNV	C1546T	P516S	M16	D	B	1.78
3	42732364	42732364	KLHL40	kelch-like family member 40	NM_152393	G	A	nonsynonymous SNV	G1621A	E541K	M16	T	B	1.74
3	49699177	49699177	BSN	bassoon presynaptic cytomat	NM_003458	G	A	nonsynonymous SNV	G9899A	G3300E	M16	T	D	5.33
3	49936108	49936108	MST1R	macrophage stimulating 1 rec	NM_002447.2	G	A	nonsynonymous SNV	C1562T	P521L	M16	D	D	4.95
3	51393628	51393628	DOCK3	dedicator of cytokinesis 3	NM_004947	A	G	nonsynonymous SNV	A4358G	K1453R	M16	T	B	4.2
3	52417889	52417889	DNAH1	dynein, axonemal, heavy cha	NM_015512	G	A	nonsynonymous SNV	G8164A	V2722I	M16	T	B	3.32
3	52518642	52518642	NISCH	nischarin	NM_007184	C	T	nonsynonymous SNV	C1642T	P548S	M16	T	B	5.71
3	53381542	53381542	DCP1A	Decapping mRNA 1A	NM_018403	C	T	nonsynonymous SNV	G3A	M1I	M16			
3	74420367	74420367	CNTN3	Contactin 3 (plasmacytoma a	NM_020872	G	A	nonsynonymous SNV	C638T	P213L	M16	T	D	5.81
3	74420368	74420368	CNTN3	Contactin 3 (plasmacytoma a	NM_020872	G	A	nonsynonymous SNV	C637T	P213S	M16	T	B	5.81
3	97887693	97887693	OR5H15	Olfactory receptor, family 5, s	NM_001005515	G	A	stopgain	G150A	W50X	M16	T		1.58
3	98110187	98110187	OR5K3	Olfactory receptor, family 5, s	NM_001005516	G	A	nonsynonymous SNV	G678A	M226I	M16	T	B	3.22
4	113352162	113352162	ALPK1	Alpha-kinase 1	NM_025144	G	A	nonsynonymous SNV	G1459A	G487R	M16	D	B	4.64
4	121957787	121957787	NDNF	Neuron-derived neurotrophic	NM_024574	G	A	nonsynonymous SNV	C1339T	L447F	M16	T	D	4.08
4	126402868	126402868	FAT4	FAT atypical cadherin 4	NM_001291285	A	C	nonsynonymous SNV	A12797C	E4266A	M16	T	D	4.91
4	1345574	1345574	UVSSA	UV-stimulated scaffold protei	NM_020894	G	C	nonsynonymous SNV	G501C	L167F	M16	T	D	3.08
4	135121676	135121676	PABPC4L	Poly(A) binding protein, cytop	NM_001114734	C	T	nonsynonymous SNV	G673A	V225M	M16			
4	13603320	13603320	BOD1L1	Biorientation of chromosomes	NM_148894	C	T	nonsynonymous SNV	G5204A	R1735K	M16	T	B	4.81
4	145573860	145573860	HHIP	Hedgehog interacting protein	NM_022475	G	A	nonsynonymous SNV	G383A	R128K	M16	T	B	1.17
4	147830214	147830214	TTC29	Tetratricopeptide repeat doma	NM_001300761	G	A	nonsynonymous SNV	C442T	H148Y	M16	T	B	-0.287
4	154702631	154702631	SFRP2	Secreted frizzled-related prot	NM_003013	G	A	nonsynonymous SNV	C860T	S287F	M16	T	B	5.95
4	156632263	156632263	GUCY1A3	Guanylate cyclase 1, soluble,	NM_000856	G	A	nonsynonymous SNV	G946A	G316R	M16	T	B	5.76
4	15720535	15720535	BST1	Bone marrow stromal cell ant	NM_004334	C	T	nonsynonymous SNV	C710T	S237F	M16	D	D	4.75
4	165118773	165118773	ANP32C	Acidic (leucine-rich) nuclear p	NM_012403	C	T	nonsynonymous SNV	G91A	E31K	M16			

4	166916323	166916323	TLL1	Tolloid-like 1	NM_012464	C	T	nonsynonymous SNV	C625T	P209S	M16	T	D	5.83
4	166916324	166916324	TLL1	Tolloid-like 1	NM_012464	C	T	nonsynonymous SNV	C626T	P209L	M16	D	D	5.83
4	167921594	167921594	SPOCK3	Sparc/osteonectin, cwcv and	NM_016950	G	A	nonsynonymous SNV	C265T	P89S	M16	T	D	5.4
4	175636678	175636678	GLRA3	Glycine receptor, alpha 3	NM_006529	G	A	nonsynonymous SNV	C535T	P179S	M16	D	D	5.49
4	175899129	175899129	ADAM29	ADAM metallopeptidase dom	NM_001278125	C	T	nonsynonymous SNV	C2453T	T818M	M16		P	-0.888
4	186112121	186112121	CFAP97	Cilia and flagella associated p	NM_020827	G	A	nonsynonymous SNV	C230T	P77L	M16	T	B	-3.29
4	187130333	187130333	CYP4V2	Cytochrome P450, family 4, s	NM_207352	G	A	nonsynonymous SNV	G1312A	E438K	M16	T	B	5.39
4	187149402	187149402	KLKB1	Kallikrein B, plasma (Fletcher	NM_000892	C	T	nonsynonymous SNV	C53T	S18F	M16	D	B	4.39
4	188924697	188924697	ZFP42	Zinc finger protein 42	NM_174900	C	T	nonsynonymous SNV	C736T	R246W	M16	D	P	4.39
4	265061	265061	ZNF732	Zinc finger protein 732	NM_001137608	G	A	nonsynonymous SNV	C1582T	P528S	M16	D	B	0.977
4	265062	265062	ZNF732	Zinc finger protein 732	NM_001137608	T	A	nonsynonymous SNV	A1581T	K527N	M16	D	P	0.977
4	3225218	3225218	HTT	Huntingtin	NM_002111	C	T	nonsynonymous SNV	C7556T	A2519V	M16	D	P	4.88
4	40104742	40104742	N4BP2	NEDD4 binding protein 2	NM_018177	C	T	nonsynonymous SNV	C1277T	P426L	M16	D	B	5.24
4	4304146	4304146	ZBTB49	Zinc finger and BTB domain c	NM_145291	C	T	nonsynonymous SNV	C583T	P195S	M16	T	B	0.26
4	55593661	55593661	KIT	v-kit Hardy-Zuckerman 4 felir	M_000222	T	C	nonsynonymous SNV	T1727C	L576P	M16	D	B	6.06
4	675895	675895	MFSD7	Major facilitator superfamily d	NM_032219	C	A	nonsynonymous SNV	G1532T	C511F	M16	D	B	0.375
4	69533829	69533829	UGT2B15	UDP glucuronosyltransferase	NM_001076	G	A	nonsynonymous SNV	C802T	P268S	M16	D	D	1.57
4	70079799	70079799	UGT2B11	UDP glucuronosyltransferase	NM_001073	C	T	nonsynonymous SNV	G642A	M214I	M16	D	B	1.04
4	70504860	70504860	UGT2A1,UGT2A	UDP glucuronosyltransferase	NM_001252274; NM_00	G	A	nonsynonymous SNV	C1102T; C499T	L368F; L167F	M16	D	D	2.16
4	70504997	70504997	UGT2A1,UGT2A	UDP glucuronosyltransferase	NM_001252274; NM_00	C	T	nonsynonymous SNV	G965A; G362A	G322E; G121E	M16	T	B	5.01
4	70505036	70505036	UGT2A1,UGT2A	UDP glucuronosyltransferase	NM_001252274; NM_00	G	A	nonsynonymous SNV	C926T; C323T	P309L; P108L	M16	T	D	5.01
4	71255509	71255509	SMR3B	submaxillary gland androgen	NM_006685	C	T	nonsynonymous SNV	C184T	P62S	M16	D	B	-0.071
4	74277783	74277783	ALB	Albumin	NM_000477	C	T	nonsynonymous SNV	C784T	L262F	M16	D	D	3.39
4	79343109	79343109	FRAS1	Fraser extracellular matrix co	NM_025074	C	T	nonsynonymous SNV	C4633T	P1545S	M16	T	B	-11.4
4	8621228	8621228	CPZ	Carboxypeptidase Z	NM_003652	G	A	nonsynonymous SNV	G1810A	E604K	M16	T	B	0.719
5	1033496	1033496	NKD2	naked cuticle homolog 2	NM_033120	C	T	nonsynonymous SNV	C212T	P71L	M16	D	D	1.79
5	111504758	111504758	EPB41L4A	Erythrocyte membrane protei	NM_022140	C	T	nonsynonymous SNV	G1784A	R595Q	M16	T	D	5.86
5	122495210	122495210	PRDM6	PR domain containing 6	NM_001136239	C	T	nonsynonymous SNV	C1031T	S344L	M16	D	D	5.67
5	1334432	1334432	CLPTM1L	CLPTM1-like	NM_030782	G	A	nonsynonymous SNV	C863T	A288V	M16	T	D	4.48
5	135692463	135692463	TRPC7	Transient receptor potential c	NM_020389	C	T	nonsynonymous SNV	G613A	E205K	M16	T	D	5.26
5	13876871	13876871	DNAH5	Dynein, axonemal, heavy cha	NM_001369	A	T	stopgain	T3318A	Y1106X	M16			
5	140250898	140250898	PCDHA11	Protocadherin alpha 11	NM_018902	C	T	nonsynonymous SNV	C2210T	T737M	M16	T	B	-2.4
5	140474807	140474807	PCDHB2	Protocadherin beta 2	NM_018936	C	T	nonsynonymous SNV	C433T	P145S	M16	T	B	-2.2
5	140476678	140476678	PCDHB2	Protocadherin beta 2	NM_018936	C	G	nonsynonymous SNV	C2304G	F768L	M16	D	B	-1.54
5	140517327	140517327	PCDHB5	Protocadherin beta 5	NM_015669	C	T	nonsynonymous SNV	C2311T	P771S	M16	T	B	4.61
5	140558735	140558735	PCDHB8	Protocadherin beta 8	NM_019120	C	T	nonsynonymous SNV	C1120T	L374F	M16	D	B	4.25
5	140731433	140731433	PCDHGB1	Protocadherin gamma subfar	NM_018922	C	T	nonsynonymous SNV	C1606T	P536S	M16	D	D	4.61
5	140741666	140741666	PCDHGB2	Protocadherin gamma subfar	NM_018923	C	T	nonsynonymous SNV	C1964T	S655F	M16	D	P	4.95
5	150275261	150275261	ZNF300	Zinc finger protein 300	NM_001172831	T	G	nonsynonymous SNV	A1588C	I530L	M16	D	P	1.43
5	153029980	153029980	GRIA1	Glutamate receptor, ionotropi	NM_000827	G	A	nonsynonymous SNV	G551A	G184E	M16	T	B	5.33
5	153795390	153795390	GALNT10	Polypeptide N-acetylgalactos	NM_198321	G	T	nonsynonymous SNV	G1551T	Q517H	M16	T	P	4.72
5	153795391	153795391	GALNT10	Polypeptide N-acetylgalactos	NM_198321	C	A	nonsynonymous SNV	C1552A	H518N	M16	T	P	5.6
5	154330488	154330488	MRPL22	Mitochondrial ribosomal prote	NM_014180	G	C	nonsynonymous SNV	G185C	R62P	M16	T	B	4.03
5	161528284	161528284	GABRG2	Gamma-aminobutyric acid (G	NM_198903	C	T	nonsynonymous SNV	C592T	P198S	M16	D	D	5.45
5	162940850	162940850	MAT2B	Methionine adenosyltransfera	NM_013283	G	T	nonsynonymous SNV	G376T	A126S	M16	T	B	3.55
5	176523307	176523307	FGFR4	Fibroblast growth factor recep	NM_002011	G	A	stopgain	G1964A	W655X	M16	T		4.65
5	23526683	23526683	PRDM9	PR domain containing 9	NM_020227	G	A	nonsynonymous SNV	G1486A	E496K	M16	T	B	-0.417
5	33881348	33881348	ADAMTS12	ADAM metallopeptidase with	NM_030955	C	T	nonsynonymous SNV	G365A	R122K	M16	T	D	5.65
5	35691146	35691146	SPEF2	Sperm flagellar 2	NM_024867	T	C	nonsynonymous SNV	T1532C	V511A	M16	T	B	1.23
5	35800219	35800219	SPEF2	Sperm flagellar 2	NM_024867	A	C	nonsynonymous SNV	A4980C	Q1660H	M16	T	P	0.529
5	39135031	39135031	FYB	FYN binding protein	NM_001243093	C	T	nonsynonymous SNV	G1631A	G544E	M16	T	D	5.67
5	40945400	40945400	C7	Complement component 7	M_000587	C	T	nonsynonymous SNV	C668T	S223F	M16	T	B	-5.03
5	40947888	40947888	C7	Complement component 7	M_000587	C	T	nonsynonymous SNV	C923T	S308L	M16	D	D	5.9
5	40959587	40959587	C7	Complement component 7	M_000587	G	A	stopgain	G1526A	W509X	M16	T		5.51
5	41032878	41032878	MROH2B	Maestro heat-like repeat fami	NM_173489	G	A	nonsynonymous SNV	C2407T	R803W	M16	D	D	5.79
5	42718559	42718559	GHR	Growth hormone receptor	NM_001242400	G	A	nonsynonymous SNV	G950A	G317E	M16	D	D	6.02
5	42799501	42799501	CCDC152	Coiled-coil domain containing	NM_001134848	C	T	stopgain	C583T	Q195X	M16	D		5.23
5	49706770	49706770	EMB	Embigin	NM_198449	C	T	nonsynonymous SNV	G413A	G138E	M16	D	D	4.53
5	64521965	64521965	ADAMTS6	ADAM metallopeptidase with	NM_197941	C	T	nonsynonymous SNV	G2015A	G672E	M16	D	D	5.2
5	64907539	64907539	TRIM23	Tripartite motif containing 23	NM_001656	G	A	nonsynonymous SNV	C536T	S179F	M16	D	P	4.23
5	76700605	76700605	PDE8B	Phosphodiesterase 8B	NM_003719	C	T	nonsynonymous SNV	C1271T	S424L	M16	T	B	3.74
5	79616096	79616096	SPZ1	Spermatogenic leucine zippe	NM_032567	C	T	nonsynonymous SNV	C62T	P21L	M16	D	P	4.19
6	117665316	117665316	ROS1	ROS proto-oncogene 1 , rece	NM_002944	C	T	stopgain	G4431A	W1477X	M16	T		4.23
6	117709113	117709113	ROS1	ROS proto-oncogene 1 , rece	NM_002944	G	A	nonsynonymous SNV	C1844T	S615F	M16	D	B	2.42
6	145075868	145075868	UTRN	Utrophin	NM_007124	G	A	nonsynonymous SNV	G8368A	D2790N	M16	D	P	5.13
6	159029430	159029430	TMEM181	Transmembrane protein 181	NM_020823	C	T	stopgain	C1150T	Q384X	M16		T	5.21
6	159660971	159660971	FNDC1	Fibronectin type III domain cc	NM_032532	G	A	nonsynonymous SNV	G4603A	E1535K	M16	T	P	4.18

6	170160008	170160008	ERMARD	ER membrane-associated RN	NM_018341	T	C	nonsynonymous SNV	T680C	L227P	M16	T	P	-4.13
6	18236798	18236798	DEK	DEK proto-oncogene	NM_003472	G	A	nonsynonymous SNV	C932T	P311L	M16	T		4.88
6	26046032	26046032	HIST1H3C	Histone cluster 1, H3c	NM_003531	C	T	nonsynonymous SNV	C394T	R132C	M16			
6	27279063	27279063	POM121L2	POM121 transmembrane nuc	NM_033482	G	A	nonsynonymous SNV	C887T	S296F	M16	T	B	1.24
6	29079681	29079681	OR2J3	Olfactory receptor, family 2, s	NM_001005216	G	A	nonsynonymous SNV	G14A	G5E	M16	T	B	1.23
6	30917741	30917741	DPCR1	Diffuse panbronchiolitis critica	NM_080870	A	T	nonsynonymous SNV	A1500T	E500D	M16	T	B	-1.08
6	31797607	31797607	HSPA1B	Heat Shock 70kDa Protein 1E	NM_005346	C	T	nonsynonymous SNV	C1880T	P627L	M16	D		2.12
6	41197860	41197860	TREML4	Triggering Receptor Express	NM_198153	G	A	nonsynonymous SNV	G506A	R169K	M16	T	B	3.27
6	46657888	46657888	TDRD6	tudor domain containing 6	NM_001010870	G	A	nonsynonymous SNV	G2023A	E675K	M16	T	B	3.88
6	55659082	55659082	BMP5	Bone Morphogenetic Protein	NM_021073	C	T	nonsynonymous SNV	G827A	G276E	M16	T	B	5.02
6	55933863	55933863	COL21A1	Collagen, Type XXI, Alpha 1	NM_030820	G	A	nonsynonymous SNV	C2072T	P691L	M16	D	D	4.38
7	100639155	100639155	MUC12	Mucin 12, Cell Surface Assoc	NM_001164462	G	T	nonsynonymous SNV	G5311T	A1771S	M16	T		-0.333
7	100678530	100678530	MUC17	Mucin 17, Cell Surface Assoc	NM_001040105	C	T	nonsynonymous SNV	G5311T	A1771S	M16	T		-0.333
7	103138608	103138608	RELN	Reelin	NM_005045	A	G	nonsynonymous SNV	T8759C	I2920T	M16	T	B	5.89
7	11078450	11078450	PHF14	PHD Finger Protein 14	NM_014660	A	T	nonsynonymous SNV	A2044T	I682L	M16	T	B	4.64
7	116339836	116339836	MET	MET proto-oncogene, recept	NM_001127500	C	T	nonsynonymous SNV	C698T	S233F	M16	D	D	6.17
7	120385981	120385981	KCND2	Potassium Channel, Voltage	NM_012281	C	T	nonsynonymous SNV	C1615T	R539C	M16		B	6.06
7	123143417	123143417	IQUB	IQ motif and ubiquitin domain	NM_001282855	C	T	nonsynonymous SNV	G533A	G178E	M16	D	D	5.31
7	12379974	12379974	VWDE	Von Willebrand Factor D And	NM_001135924	C	T	nonsynonymous SNV	G4340A	G1447E	M16	D	D	4.73
7	126173484	126173484	GRM8	Glutamate receptor, metabot	NM_000845	C	T	nonsynonymous SNV	G1952A	R651Q	M16	T	D	5.75
7	126882873	126882873	GRM8	Glutamate receptor, metabot	NM_000845	G	A	nonsynonymous SNV	C386T	S129L	M16	D	B	3.75
7	128588858	128588858	IRF5	interferon regulatory factor 5	NM_001098630	G	T	nonsynonymous SNV	G1483T	A495S	M16	T	B	5.87
7	141840851	141840851	LOC93432		NM_001293626	G	A	nonsynonymous SNV	G1249A	E417K	M16	T	B	-4
7	141895967	141895967	LOC93432		NM_001293626	G	A	nonsynonymous SNV	G4360A	V1454M	M16	T		4.5
7	143054065	143054065	FAM131B	Family with sequence similari	NM_001278297	G	A	stopgain	C379T	Q127X	M16	D		5.46
7	143807150	143807150	OR2A2	Olfactory Receptor, Family 2,	NM_001005480	G	A	nonsynonymous SNV	G475A	E159K	M16	D	B	-7.21
7	148725416	148725416	PDIA4	Protein Disulfide Isomerase F	NM_004911	C	T	nonsynonymous SNV	G85A	E29K	M16	T	B	5.58
7	148951472	148951472	ZNF212	Zinc Finger Protein 212	NM_012256	C	T	nonsynonymous SNV	C1454T	A485V	M16	D	B	1.9
7	150164195	150164195	GIMAP8	GTPase, IMAP Family Memb	NM_175571	G	A	nonsynonymous SNV	G409A	D137N	M16	D	D	4.47
7	29152409	29152409	CPVL	Carboxypeptidase, vitellogen	NM_031311	G	A	nonsynonymous SNV	C199T	P67S	M16	T	B	0.771
7	35242110	35242110	TBX20	T-box 20	NM_001077653	G	A	stopgain	C1276T	Q426X	M16	T		5.66
7	39503902	39503902	POU6F2	POU class 6 homeobox 2	NM_007252	G	A	nonsynonymous SNV	G1693	D565N	M16	D	P	5.35
7	4185524	4185524	SDK1	Sidekick cell adhesion molec	NM_152744	A	T	nonsynonymous SNV	A4399T	T1467S	M16	T	B	4.96
7	47880086	47880086	PKD1L1	Polycystic kidney disease 1 li	NM_138295	C	T	nonsynonymous SNV	G5525A	R1842K	M16	T	B	3.66
7	5338638	5338638	SLC29A4	Solute carrier family 29 (equil	NM_001040661	T	A	nonsynonymous SNV	T902A	L301Q	M16	T	P	0.891
7	63726846	63726846	ZNF679	Zinc finger protein 679	NM_153363	C	A	nonsynonymous SNV	C835A	R279S	M16	T	B	0.81
7	63727156	63727156	ZNF679	Zinc finger protein 679	NM_153363	G	T	nonsynonymous SNV	G1145T	C382F	M16	D	P	0.819
7	6505875	6505875	KDELR2	KDEL (Lys-Asp-Glu-Leu) end	NM_006854	G	A	nonsynonymous SNV	C431T	A144V	M16	T	B	5.18
7	75131251	75131251	SPDYE5	Speedy/RINGO cell cycle reg	NM_001099435	G	A	nonsynonymous SNV	G1004A	R335H	M16			
7	8167593	8167593	ICA1	slet cell autoantigen 1, 69kDa	NM_001136020	C	T	nonsynonymous SNV	G1240A	E414K	M16	T	P	5.64
7	82579950	82579950	PCLO	Piccolo presynaptic cytomatri	NM_033026	A	T	stopgain	14406T	F4802F	M16			
7	82585599	82585599	PCLO	Piccolo presynaptic cytomatri	NM_033026	C	T	nonsynonymous SNV	G4670A	R1557Q	M16	D	D	5.26
7	86415872	86415872	GRM3	Glutamate receptor, metabot	NM_000840	C	T	nonsynonymous SNV	C764T	S255F	M16	D	P	6.07
7	87046717	87046717	ABCB4	ATP-binding cassette, sub-fa	NM_018849	G	A	nonsynonymous SNV	C2593T	P865S	M16	D	D	5.82
7	87183115	87183115	ABCB1	ATP-binding cassette, sub-fa	NM_000927	C	T	nonsynonymous SNV	G961A	V321I	M16	T	B	3.1
7	87537116	87537116	DBF4	DBF4 zinc finger	NM_006716	C	T	nonsynonymous SNV	C1663T	P555S	M16	T	B	2.63
7	878602	878602	SUN1	Sad1 and UNC84 domain co	NM_001130965	C	T	nonsynonymous SNV	C245T	S82F	M16	D	D	2.45
7	99956439	99956439	PILRB	Paired immunoglobin-like typ	NM_178238	T	C	nonsynonymous SNV	T191C	V64A	M16		B	-2.11
8	10466281	10466281	RP1L1	Retinitis pigmentosa 1-like 1	NM_178857	G	A	nonsynonymous SNV	C5327T	T1776I	M16	T	B	-0.077
8	10755826	10755826	XKR6	XK, Kell blood group complex	NM_173683	G	A	nonsynonymous SNV	C1562T	P521L	M16	T	D	5.37
8	108297076	108297076	ANGPT1	Angiopoietin 1	NM_001146	C	T	nonsynonymous SNV	G1039A	G347S	M16	D	D	5.73
8	113308134	113308134	CSMD3	CUB and Sushi multiple dom	NM_198123	G	A	stopgain	C8542T	R2848X	M16	T		4.42
8	121211740	121211740	COL14A1	Collagen, type XIV, alpha 1	NM_021110	C	T	nonsynonymous SNV	C812T	S271F	M16	D	D	5.54
8	128428248	128428248	POU5F1B	POU class 5 homeobox 1B	NM_001159542	G	A	nonsynonymous SNV	G137A	G46E	M16	T	P	-0.298
8	133044209	133044209	OC90	Otoconin 90	NM_001080399	G	A	nonsynonymous SNV	C950T	S317F	M16	D	P	4.98
8	139793190	139793190	COL22A1	Collagen, type XXII, alpha 1	NM_152888	C	T	nonsynonymous SNV	G1630A	D544N	M16	T	P	4.23
8	144942234	144942234	EPPK1	Epiplakin 1	NM_031308	C	T	nonsynonymous SNV	G5188A	D1730N	M16	T	D	4.84
8	145746561	145746561	LRRC14	Leucine rich repeat containin	NM_014665	G	A	nonsynonymous SNV	G1181A	S394N	M16	T	B	-0.716
8	15519748	15519748	TUSC3	Tumor suppressor candidate	NM_006765	G	T	nonsynonymous SNV	G651T	L217F	M16	T	D	-0.3
8	27462453	27462453	CLU	Clusterin	NM_001831	C	T	nonsynonymous SNV	G817A	E273K	M16	T	B	3.34
8	2820066	2820066	CSMD1	CUB and Sushi multiple dom	NM_033225	G	A	nonsynonymous SNV	C9550T	P3184S	M16	T	D	5.6
8	39442835	39442835	ADAM18	ADAM metallopeptidase dom	NM_014237	G	T	nonsynonymous SNV	G96T	p.K32N	M16	D	D	1.92
8	39468085	39468085	ADAM18	ADAM metallopeptidase dom	NM_014237	G	A	nonsynonymous SNV	G382A	E128K	M16	T	D	4.37
8	52359645	52359645	PXDNL	Peroxidasin-like	NM_144651	C	T	nonsynonymous SNV	G1444A	D482N	M16	D	P	0.972
8	55540830	55540830	RP1	Retinitis pigmentosa 1	NM_006269	C	T	nonsynonymous SNV	C4388T	S1463L	M16	D	B	5.48
8	55542620	55542620	RP1	Retinitis pigmentosa 1	NM_006269	G	A	nonsynonymous SNV	G6178A	E2060K	M16	D	B	5.48
8	68213506	68213506	ARFGEF1	ADP-ribosylation factor guani	NM_006421	G	A	stopgain	C265T	Q89X	M16	T		5.28

8	73850151	73850151	KCNB2	Potassium channel, voltage g	NM_004770	C	T	nonsynonymous SNV	C2561T	S854F	M16	D	P	5.46
8	73979618	73979618	SBSPON	Somatomedin B and thrombo	NM_153225	G	T	nonsynonymous SNV	C753A	D251E	M16	T	B	-5.2
8	76471138	76471138	HNF4G	Hepatocyte nuclear factor 4,	NM_004133	G	A	nonsynonymous SNV	G959A	G320E	M16	D	D	4.73
8	7718170	7718170	SPAG11A	Sperm associated antigen 11	NM_001081552	G	A	nonsynonymous SNV	G217A	D73N	M16	T	P	2.28
8	81577195	81577195	ZNF704	Zinc finger protein 704	NM_001033723	G	A	nonsynonymous SNV	C782T	S261F	M16	D	B	6.07
9	103108569	103108569	TEX10	testis expressed 10	NM_017746	C	T	nonsynonymous SNV	G922A	D308N	M16	T	P	5.15
9	113549986	113549986	MUSK	muscle, skeletal, receptor tyr	NM_005592	C	T	nonsynonymous SNV	C1795T	P599S	M16	T	P	5.18
9	115961176	115961176	FKBP15	FK506 binding protein 15, 13	NM_015258	T	A	stopgain	A676T	K226X	M16	T		5.47
9	124536639	124536639	DAB2IP	DAB2 interacting protein	NM_138709	C	T	nonsynonymous SNV	C2696T	P899L	M16	T	B	4.55
9	125004267	125004267	RBM18	RNA Binding Motif Protein 18	NM_033117	G	A	nonsynonymous SNV	C469T	P157S	M16	T	P	5.52
9	125273445	125273445	OR1J2	Olfactory Receptor, Family 1,	NM_054107	G	A	nonsynonymous SNV	G365A	R122Q	M16	D	D	3.11
9	125512491	125512491	OR1L6	Olfactory Receptor, Family 1,	NM_001004453	A	C	nonsynonymous SNV	A365C	D122A	M16	D	D	4.62
9	131513474	131513474	ZER1	Zyg-11 Related, Cell Cycle R	NM_006336	G	A	nonsynonymous SNV	C1112T	T371I	M16	T	B	5.01
9	131721120	131721120	NUP188	Nucleoporin 188kDa	NM_015354	C	T	nonsynonymous SNV	C412T	R138C	M16	D	D	5.41
9	136656942	136656942	VAV2	Vav 2 guanine nucleotide exc	NM_001134398	C	T	nonsynonymous SNV	G1151A	R384K	M16	T	B	2.86
9	139996098	139996098	MAN1B1	Mannosidase, Alpha, Class 1	NM_016219	C	T	nonsynonymous SNV	C1228T	R410C	M16		D	3.91
9	140099559	140099559	TMEM203	Transmembrane Protein 203	NM_053045	T	C	nonsynonymous SNV	A308G	Q103R	M16	T	B	3.16
9	141012486	141012486	CACNA1B	Calcium channel, voltage-dep	NM_000718	G	A	nonsynonymous SNV	G5866A	E1956K	M16	T	B	4.26
9	32630987	32630987	TAF1L	TAF1 RNA Polymerase II, TA	NM_153809	C	T	nonsynonymous SNV	G4591A	D1531N	M16	T	P	0.489
9	34990697	34990697	DNAJB5	DnaJ (Hsp40) homolog, subfa	NM_001135004	C	T	nonsynonymous SNV	C196T	R66W	M16	D	B	4.59
9	84607864	84607864	SPATA31D1	SPATA31 subfamily D, memb	NM_001001670	G	A	nonsynonymous SNV	G2479A	E827K	M16	T	P	-0.228
9	893957	893957	DMRT1	doublesex and mab-3 related	NM_021951	G	A	nonsynonymous SNV	G584A	G195E	M16	T	D	5.92
9	90296377	90296377	DAPK1	Death-associated protein kina	NM_004938	G	T	nonsynonymous SNV	G2060T	R687I	M16	D	D	3.34
9	90745700	90745700	SPATA31C2	SPATA31 subfamily C, memb	NM_001166137	G	A	nonsynonymous SNV	C2252T	S751L	M16			
9	90746796	90746796	SPATA31C2	SPATA31 subfamily C, memb	NM_001166137	C	T	nonsynonymous SNV	G1156A	V386M	M16			
9	96097714	96097714	C9orf129	Chromosome 9 open reading	NM_001098808	G	A	nonsynonymous SNV	C307T	P103S	M16	D		2.56
X	101969840	101969840	ARMCX5-GPRA	G protein-coupled receptor a	NM_001004051	G	A	nonsynonymous SNV	G43A	E15K	M16	T	B	3.57
X	107449786	107449786	COL4A6	Collagen, type IV, alpha 6	NM_001287758	G	A	nonsynonymous SNV	C571T	P191S	M16	T	B	2.98
X	110439150	110439150	PAK3	PAK3	NM_001128168	G	A	nonsynonymous SNV	G1299A	M433I	M16	T	B	5.46
X	11157531	11157531	ARHGAP6	Rho GTPase activating prote	NM_013427	C	T	nonsynonymous SNV	G2377A	D793N	M16	T	B	1.1
X	115590032	115590032	SLC6A14	Solute carrier family 6 (aminc	NM_007231	C	T	nonsynonymous SNV	C1840T	R614C	M16	D	D	5.68
X	118708878	118708878	UBE2A	Ubiquitin-conjugating enzyme	NM_003336	C	T	nonsynonymous SNV	C59T	P20L	M16	D	D	4.87
X	119694283	119694283	CUL4B	Cullin 4B	NM_001079872	G	T	nonsynonymous SNV	C211A	Q71K	M16		B	5.39
X	122532536	122532536	GRIA3	Glutamate receptor, ionotropi	NM_000828	T	C	nonsynonymous SNV	T962C	F321S	M16	T	D	5.93
X	128621065	128621065	SMARCA1	SWI/SNF related, matrix asso	NM_001282874	C	T	nonsynonymous SNV	G2147A	R716K	M16	T	B	3.96
X	129306163	129306163	RAB33A	RAB33A, member RAS onco	NM_004794	G	A	nonsynonymous SNV	G127A	G43R	M16	D	D	4.67
X	129306164	129306164	RAB33A	RAB33A, member RAS onco	NM_004794	G	A	nonsynonymous SNV	G128A	G43E	M16	D	D	4.67
X	132352251	132352251	TFDP3	Transcription factor Dp family	NM_016521	C	T	nonsynonymous SNV	G37A	E13K	M16	D	B	0.235
X	134185895	134185895	FAM127B	Family with sequence similar	NM_001078172	G	A	nonsynonymous SNV	C244T	P82S	M16		P	2.38
X	134989589	134989589	SAGE1	Sarcoma antigen 1	NM_018666	C	T	nonsynonymous SNV	C995T	P332L	M16	T	B	1.07
X	135581923	135581923	HTATSF1	HIV-1 Tat specific factor 1	NM_001163280	G	A	nonsynonymous SNV	G353A	R118K	M16	T	P	5.96
X	140995048	140995048	MAGEC1	Melanoma antigen family C1	NM_005462	G	A	nonsynonymous SNV	G1858A	E620K	M16	D	B	-1.75
X	150349133	150349133	GPR50	G protein-coupled receptor 5	NM_004224	G	A	nonsynonymous SNV	G1078A	E360K	M16	T	B	3.7
X	150868495	150868495	PRRG3	Proline rich Gla (G-carboxygl	NM_024082	C	T	nonsynonymous SNV	C35T	S12L	M16	T	B	3.95
X	151935268	151935268	MAGEA3	Melanoma antigen family A3	NM_005362	G	A	nonsynonymous SNV	C899T	S300F	M16	D	B	0.488
X	154348337	154348337	BRCC3	BRCA1/BRCA2-containing co	NM_024332	A	G	nonsynonymous SNV	A863G	E288G	M16	D	P	5.1
X	17819842	17819842	RAI2	Retinoic acid induced 2	NM_001172732	C	T	nonsynonymous SNV	G139A	E47K	M16	T	B	5.4
X	20062628	20062628	MAP7D2	MAP7 domain containing 2	NM_001168465	G	A	nonsynonymous SNV	C733T	P245S	M16	T	P	5.1
X	20134868	20134868	MAP7D2	MAP7 domain containing 2	NM_001168465	C	T	nonsynonymous SNV	G130A	G44S	M16	P	P	3.22
X	24024728	24024728	KLHL15	Kelch-like family member 15	NM_030624	C	T	nonsynonymous SNV	G83A	G28E	M16	D	B	4.57
X	26157335	26157335	MAGEB18	Melanoma antigen family B1	NM_173699	C	T	nonsynonymous SNV	C233T	S78L	M16	T	D	3.8
X	30872664	30872664	TAB3	TGF-beta activated kinase 1/	NM_152787:	G	A	nonsynonymous SNV	C1118T	S373F	M16	D	B	4.66
X	3229130	3229130	MXRA5	Matrix-remodelling associate	NM_015419	C	T	nonsynonymous SNV	G7114A	G2372R	M16	D	D	4.29
X	3239147	3239147	MXRA5	Matrix-remodelling associate	NM_015419	C	T	nonsynonymous SNV	G4579A	E1527K	M16	T	B	2.3
X	3241018	3241018	MXRA5	Matrix-remodelling associate	NM_015419	C	T	nonsynonymous SNV	G2708A	G903E	M16	T	B	-3.78
X	36366352	36366352	CXorf30	Cilia and flagella associated	NM_001098843	G	A	nonsynonymous SNV	G890A	G297E	M16	T	D	1.8
X	38267995	38267995	OTC	Ornithine carbamoyltransfera	NM_000531	G	A	nonsynonymous SNV	G664A	G222S	M16		P	5.58
X	38267996	38267996	OTC	Ornithine carbamoyltransfera	NM_000531	G	A	nonsynonymous SNV	G665A	G222D	M16	D	D	5.88
X	39911436	39911436	BCOR	BCL6 corepressor	NM_001123384	C	T	nonsynonymous SNV	G5038A	E1680K	M16	D	P	5.63
X	46360621	46360621	ZNF674	Zinc finger protein 674	NM_001039891	C	T	nonsynonymous SNV	G403A	D135N	M16	T	B	-5.43E-04
X	49113469	49113469	FOXP3	Forkhead box P3	NM_014009	C	T	nonsynonymous SNV	G455A	G152E	M16	T	D	4.14
X	53579280	53579280	HUWE1	HECT, UBA and WWE domai	NM_031407	G	A	nonsynonymous SNV	C8873T	P2958L	M16	T	D	5.95
X	55783759	55783759	RRAGB	Ras-related GTP binding B	M_006064	C	T	nonsynonymous SNV	C938T	S313F	M16	D	P	4.39
X	63411366	63411366	AMER1	APC membrane recruitment	NM_152424	G	A	stopgain	C1801T	R601X	M16	T		-0.532
X	69646824	69646824	GDPD2	Glycerophosphodiester phos	NM_001171192	C	T	nonsynonymous SNV	C665T	P222L	M16		D	2.6
X	73812165	73812165	RLIM	Ring finger protein, LIM doma	NM_016120	G	A	nonsynonymous SNV	C985T	R329C	M16	D	D	5.73
X	77528774	77528774	CYSLTR1	Cysteinyl leukotriene recepto	NM_001282186	G	A	nonsynonymous SNV	C470T	P157L	M16	D	D	4.44

X	79698499	79698499	FAM46D	Family with sequence similar	NM_001170574	G	A	nonsynonymous SNV	G461A	G154E	M16	D	D	4.8
X	79980475	79980475	BRWD3	Bromodomain and WD repea	NM_153252	C	T	stopgain	G1478A	W493X	M16			5.35
X	83126562	83126562	CYLC1	Cylicin, basic protein of sperr	NM_021118	C	T	nonsynonymous SNV	C161T	S54L	M16	T	B	-2.28
X	86890718	86890718	KLHL4	Kelch-like family member 4	NM_057162	G	A	nonsynonymous SNV	G1868A	G623E	M16	D	D	4.23
X	88008527	88008527	CPXCR1	CPX chromosome region, ca	NM_033048	C	T	nonsynonymous SNV	C112T	P38S	M16		B	-3.1
X	88009148	88009148	CPXCR1	CPX chromosome region, ca	NM_033048	G	A	nonsynonymous SNV	G733A	E245K	M16		B	0.551
X	99551325	99551325	PCDH19	Protocadherin 19	NM_001184880	G	A	nonsynonymous SNV	C3397T	R1133C	M16	D	P	4.65
12	109017374	109017374	SELPLG	Selectin P Ligand	NM_001206609	T	G	nonsynonymous SNV	A758C	E253A	M16	T	B	-5.23
13	46170839	46170839	ERICH6B	Glutamate-Rich 6B	NM_182542	G	T	nonsynonymous SNV	C302A	A101E	M16	D	B	-6.51
13	28498644	28498644	PDX1	Pancreatic And Duodenal Ho	NM_000209	G	C	nonsynonymous SNV	G658C	V220L	M16	T	B	3.12
2	179449692	179449692	TTN	Titin	NM_001267550	G	C	nonsynonymous SNV	C64676G	A21559G	M16	D	B	5.3
15	89398295	89398295	ACAN	aggrecan	NM_013227	A	T	stopgain	A2479T	K827X	M16	T		-3.62
11	44098842	44098842	ACCS	1-Aminocyclopropane-1-Carb	NM_001127219	C	G	nonsynonymous SNV	C570G	I190M	M16	D	P	3.87
7	44146353	44146353	AEBP1	AE binding protein 1	NM_001129	A	T	nonsynonymous SNV	A462T	K154N	M16	D	D	-0.51
2	27324371	27324371	CGREF1	Cell Growth Regulator With E	NM_006569	C	T	nonsynonymous SNV	G728A	G243E	M16	T	B	1.69
19	1440068	1440068	RPS15	ribosomal protein S15	NM_001018	G	C	nonsynonymous SNV	G140C	R47P	M16	T	B	3.21
1	153275079	153275079	PGLYRP3	peptidoglycan recognition pro	NM_052891	G	T	stopgain	C534A	C178X	M17	T		1.47
1	154897670	154897670	PMVK	phosphomevalonate kinase	NM_006556	C	A	stopgain	G514T	G172X	M17			
1	169510246	169510246	F5	coagulation factor V (proacce	NM_000130	G	A	nonsynonymous SNV	C4082T	P1361L	M17	T	B	-0.403
1	173878973	173878973	SERPINC1	serpin peptidase inhibitor, cla	NM_000488	G	T	nonsynonymous SNV	C870A	F290L	M17	T	D	3.57
10	14976709	14976709	DCLRE1C	DNA cross-link repair 1C	NM_001033855.2	G	T	nonsynonymous SNV	C530A	P177Q	M17	D	D	3.95
10	5009127	5009127	AKR1C1	aldo-keto reductase family 1,	NM_001353	C	A	stopgain	C261A	C87X	M17	T		-5
11	117050160	117050160	SIDT2	SID1 transmembrane family,	NM_001040455	A	T	nonsynonymous SNV	A181T	R61W	M17	T	B	1.63
11	65350589	65350589	EHBP1L1	EH domain binding protein 1-	NM_001099409	A	T	nonsynonymous SNV	A2446T	T816S	M17	T	B	-5.02
11	6648292	6648292	DCHS1	dachsous cadherin-related 1	NM_003737	G	T	nonsynonymous SNV	C5978A	A1993D	M17	T	B	5.18
12	122691869	122691869	B3GNT4	UDP-GlcNAc:betaGal beta-1,	NM_030765	G	T	nonsynonymous SNV	G1071T	W357C	M17	T	D	4.91
12	52699033	52699033	KRT86	keratin 86, type II	NM_002284	G	A	nonsynonymous SNV	G745A	V249I	M17	T	B	0.639
14	23828947	23828947	EFS	embryonal Fyn-associated su	NM_005864.3	C	G	nonsynonymous SNV	G740C	G247A	M17	T	B	2.6
14	50582893	50582893	VCPKMT	valosin containing protein lys	NM_024558.2	C	A	nonsynonymous SNV	G271T	D91Y	M17	D	B	0.76
14	77872423	77872423	NOXRED1	NADP-dependent oxidoreduc	NM_001113475	G	T	nonsynonymous SNV	C738A	N246K	M17	D	D	1.1
15	101188555	101188555	ASB7	ankyrin repeat and SOCS bo	NM_198243	G	T	nonsynonymous SNV	G845T	C282F	M17	D	D	5.51
15	66048683	66048683	DENND4A	DENN/MADD domain contair	NM_001144823.1	G	T	nonsynonymous SNV	C106A	H36N	M17	T	P	5.6
15	84651232	84651232	ADAMTSL3	ADAMTS-like 3	NM_207517.2	G	T	nonsynonymous SNV	G2852T	R951L	M17	T	B	-3.93
16	18859355	18859355	SMG1	SMG1 phosphatidylinositol 3-	NM_015092	C	A	nonsynonymous SNV	G5624T	G1875V	M17	T	D	5.83
16	28497683	28497683	CLN3	ceroid-lipofuscinosis, neurona	NM_001042432.1	G	T	nonsynonymous SNV	C662A	A221D	M17	T	P	0.596
16	58575414	58575414	CNOT1	CCR4-NOT transcription com	NM_016284.4	C	A	nonsynonymous SNV	G4791T	Q1597H	M17	T	P	3.73
16	66503736	66503736	BEAN1	brain expressed, associated	NM_001178020	G	T	nonsynonymous SNV	G257T	R86L	M17	D		1.69
17	40706526	40706526	HSD17B1	hydroxysteroid (17-beta) deh	NM_000413	C	A	nonsynonymous SNV	C643A	R215S	M17	T	B	3.19
17	650972	650972	GEMIN4	gem (nuclear organelle) asso	NM_015721	A	T	nonsynonymous SNV	T311A	I104N	M17	D	D	5.66
19	21720666	21720666	ZNF429	zinc finger protein 429	NM_001001415	G	T	nonsynonymous SNV	G1811T	R604L	M17	T	B	-1.05
19	23544189	23544189	ZNF91	zinc finger protein 91	NM_003430.3	A	T	stopgain	T1592A	L531X	M17	T		-3.43
19	2933655	2933655	ZNF77	zinc finger protein 77	NM_021217	T	A	nonsynonymous SNV	A1470T	K490N	M17	D	P	1.11
19	48204714	48204714	GLTSCR1	glioma tumor suppressor can	NM_015711	T	A	nonsynonymous SNV	T3725A	L1242Q	M17	T	B	-0.247
19	51295421	51295421	ACPT	acid phosphatase, testicular	NM_033068	G	T	nonsynonymous SNV	G542T	G181V	M17	T	B	4.39
19	52569365	52569365	ZNF841	zinc finger protein 841	NM_001136499	C	A	nonsynonymous SNV	G1770T	M590I	M17	T	B	-3.79
19	58385047	58385047	ZNF814	zinc finger protein 814	NM_001144989	G	T	nonsynonymous SNV	C1711A	P571T	M17	T	B	-3.34
2	10192380	10192380	KLF11	Kruppel-like factor 11	NM_003597.4	G	T	nonsynonymous SNV	G1285T	D429Y	M17	D	D	6.08
2	135095939	135095939	MGAT5	mannosyl (alpha-1,6-)-glycop	NM_002410	C	A	nonsynonymous SNV	C755A	A252E	M17	D	D	5.76
2	179517223	179517223	TTN	titin	NM_001267550	G	T	nonsynonymous SNV	C39089A	A13030E	M17	D	B	-1.86
2	198669955	198669955	PLCL1	phospholipase C-like 1	NM_006226	C	A	nonsynonymous SNV	C132A	D44E	M17		B	3.14
2	219694800	219694800	PRKAG3	protein kinase, AMP-activate	NM_017431	T	A	nonsynonymous SNV	A534T	K178N	M17	T	P	-0.09
2	223806303	223806303	ACSL3	acyl-CoA synthetase long-cha	NM_004457.3	C	G	nonsynonymous SNV	C2094G	F698L	M17	T	B	6.03
2	239059531	239059531	KLHL30	kelch-like family member 30	NM_198582	G	T	nonsynonymous SNV	G1562T	R521L	M17	T	B	4.66
2	3504756	3504756	ADI1	acireductone dioxygenase 1	NM_018269	C	A	nonsynonymous SNV	G249T	M83I	M17	T	B	2.9
2	7035925	7035925	RSAD2	radical S-adenosyl methionin	NM_080657	G	T	nonsynonymous SNV	G938T	C313F	M17	D	D	5.57
20	10389309	10389309	MKKS	McKusick-Kaufman syndrome	NM_018848.3	G	T	stopgain	C1128A	C376X	M17	T		3.78
20	43851376	43851376	SEMG2	semenogelin II	NM_003008	G	T	nonsynonymous SNV	G1103T	G368V	M17	D	P	-2.11
20	44207868	44207868	WFDC8	WAP four-disulfide core doma	NM_181510.2	C	T	nonsynonymous SNV	G19A	E7K	M17	T	B	-4.02
22	23654017	23654017	BCR	breakpoint cluster region	NM_004327.3	G	A	nonsynonymous SNV	G3316A	D1106N	M17	T	P	4.49
22	45607850	45607850	KIAA0930	KIAA0930	NM_015264.1	G	T	nonsynonymous SNV	C218A	A73E	M17	T	B	-10.4
3	100020913	100020913	TBC1D23	TBC1 domain family, membe	NM_001199198.2	G	T	nonsynonymous SNV	G1095T	M365I	M17		P	4.97
3	132319393	132319393	ACKR4	atypical chemokine receptor	NM_178445.2	T	C	nonsynonymous SNV	T152C	V51A	M17	T	B	5.4
3	195508558	195508558	MUC4	mucin 4, cell surface associa	NM_018406	A	T	nonsynonymous SNV	T9893A	L3298H	M17		B	0
3	49662632	49662632	BSN	bassoon presynaptic cytomat	NM_003458	G	T	nonsynonymous SNV	G449T	S150I	M17	T	B	5.34
3	50679738	50679738	MAPKAPK3	mitogen-activated protein kin	NM_001243926.1	A	T	nonsynonymous SNV	A479T	H160L	M17	T	B	-2.44
4	39114653	39114653	KLHL5	Kelch-like family member 5	NM_001007075	G	T	nonsynonymous SNV	G1702T	G568C	M17	D	D	5.67
4	85678262	85678262	WDFY3	WD repeat and FYVE domain	NM_014991	G	T	nonsynonymous SNV	C5241A	N1747K	M17	T	B	4.3

6	155450429	155450429	TIAM2	T-cell lymphoma invasion and	NM_012454	G	T	nonsynonymous SNV	G72T	K24N	M17	D	D	2.38
6	168376881	168376881	HGC6.3		M_001129895	T	G	nonsynonymous SNV	A452C	Q151P	M17			
6	52141122	52141122	MCM3	Minichromosome maintenanc	NM_001270472	C	A	nonsynonymous SNV	G1348T	A450S	M17	D	D	5.13
7	100137015	100137015	AGFG2	ArfGAP With FG Repeats 2	NM_006076	A	T	nonsynonymous SNV	A46T	S16C	M17	T	B	-1.01
7	112127073	112127073	LSMEM1	Leucine-rich single-pass men	NM_001134468	C	A	nonsynonymous SNV	C223A	L75M	M17	D	D	1.99
7	151932916	151932916	KMT2C	Lysine (K)-Specific Methyltrar	NM_170606	C	G	nonsynonymous SNV	G2755C	V919L	M17	T	B	3.05
7	5541062	5541062	FBXL18	F-box and leucine-rich repeat	NM_024963	T	A	stopgain	A838T	K280X	M17	T		4.37
7	72951692	72951692	BCL7B	B-cell CLL/lymphoma 7B	NM_001707	G	C	nonsynonymous SNV	C545G	A182G	M17	D	D	5.13
9	113637948	113637948	LPAR1	Lysophosphatidic acid recept	NM_057159	C	A	nonsynonymous SNV	G848T	C283F	M17	D	D	6.06
X	152083364	152083364	ZNF185	Zinc finger protein 185	NM_001178106	G	T	stopgain	G145T	E49X	M17	D		4.96
X	48564755	48564755	SUV39H1	Suppressor of variegation 3-9	NM_001282166	G	T	nonsynonymous SNV	G961T	V321L	M17	D	P	4.56
X	48681607	48681607	HDAC6	Histone deacetylase 6	NM_006044	T	A	nonsynonymous SNV	T2798A	M933K	M17	T	B	-7.62
19	49558211	49558211	CGB7	Chorionic Gonadotropin, Beta	NM_033142	T	G	nonsynonymous SNV	A70C	M24L	M17			946
12	109017374	109017374	SELPLG	Selectin P Ligand	NM_001206609	T	G	nonsynonymous SNV	A758C	E253A	M17	T	B	-5.23
7	57529086	57529086	ZNF716	Zinc Finger Protein 716	NM_001159279	C	A	nonsynonymous SNV	C919A	R307S	M17	T	B	-218
17	7483366	7483366	CD68	CD68 Molecule	NM_001251	T	A	nonsynonymous SNV	T288A	N96K	M17	T	B	-5.96
11	618683	618683	CDHR5	Cadherin-Related Family Mer	NM_021924	T	A	nonsynonymous SNV	A1876T	T626S	M17	T	B	592
2	113588140	113588140	IL1B	Inteleukin 1, Beta	NM_000576	G	T	nonsynonymous SNV	C608A	P203H	M17	T	D	3.63
11	77614590	77614590	INTS4	Integrator Complex Subunit 4	NM_033547	T	G	nonsynonymous SNV	A2093C	K698T	M17	T	B	4.41
2	179449692	179449692	TTN	Titin	NM_001267550	G	C	nonsynonymous SNV	C64676G	A21559G	M17	D	B	5.3
11	618667	618667	CDHR5	Cadherin-Related Family Mer	NM_021924	C	A	nonsynonymous SNV	G1892T	G631V	M17	D	B	446
12	51740409	51740409	CELA1	chymotrypsin-like elastase fa	NM_001971	T	G	nonsynonymous SNV	A14C	Y5S	M17	T	B	1.29
12	51740410	51740410	CELA1	chymotrypsin-like elastase fa	NM_001971	A	G	nonsynonymous SNV	T13C	Y5H	M17	T	B	2.28
2	113588132	113588132	IL1B	Inteleukin 1, Beta	NM_000576	A	T	nonsynonymous SNV	T616A	Y206N	M17	T	D	2.95
11	48511193	48511193	OR4A47	olfactory receptor, family 4, su	NM_001005512	A	T	nonsynonymous SNV	A849T	L283F	M17	T	B	-451
1	152129130	152129130	RPTN	repetin	NM_001122965	G	T	nonsynonymous SNV	C445A	P149T	M17	T	B	-10.4
19	20229099	20229099	ZNF90	Zinc Finger Protein 90	NM_007138	G	T	nonsynonymous SNV	G736T	A246S	M17		B	-2.22
19	20045163	20045163	ZNF93	Zinc Finger Protein 93	NM_031218	T	A	nonsynonymous SNV	T1399A	S467T	M17	T	B	-1.7
19	53269382	53269382	ZNF600	Zinc Finger Protein 600	NM_198457	A	T	nonsynonymous SNV	T1627A	C543S	M17	T	D	-3.02
1	227842419	227842419	ZNF678	Zinc Finger Protein 678	NM_178549	C	A	nonsynonymous SNV	C633A	D211E	M17	T	B	-2.68
1	101490945	101490945	DPH5	diphthamide biosynthesis 5	NM_001077394.1	C	T	nonsynonymous SNV	G55A	G19S	M2	D	D	6.08
1	155582064	155582064	MSTO1	misato 1, mitochondrial distrit	NM_018116.3	G	C	nonsynonymous SNV	G770C	G257A	M2	T	D	3.17
1	156843445	156843445	NTRK1	neurotrophic tyrosine kinase,	NM_002529.3	C	T	nonsynonymous SNV	C871T	H291Y	M2	T	B	5.03
1	161641245	161641245	FCGR2B	Fc fragment of IgG, low affinit	NM_004001.4	C	G	nonsynonymous SNV	C197G	S66C	M2	D	P	-6.97
1	162551088	162551088	UAP1	UDP-N-acetylglucosamine py	NM_003115	G	T	nonsynonymous SNV	G673T	G225C	M2	D	D	5.14
1	173019932	173019932	TNFSF18	tumor necrosis factor (ligand)	NM_005092	C	A	nonsynonymous SNV	G171T	L57F	M2	T	B	-0.765
1	220273854	220273854	IARS2	isoleucyl-tRNA synthetase 2,	NM_018060	G	T	nonsynonymous SNV	G413T	R138L	M2	D	D	5.95
1	236727946	236727946	HEATR1	HEAT repeat containing 1	NM_018072	G	T	nonsynonymous SNV	C4451A	A1484E	M2	T	B	3.95
1	248224578	248224578	OR2L3	olfactory receptor, family 2, su	NM_001004687	T	C	nonsynonymous SNV	T595C	F199L	M2	T	P	0.915
1	27876669	27876669	AHDC1	AT hook, DNA binding motif,	NM_001029882	T	C	nonsynonymous SNV	A1958G	Q653R	M2	D	P	5.51
10	37488676	37488676	ANKRD30A	ANKRD30A	NM_052997	C	T	nonsynonymous SNV	C2570T	S857L	M2	T	B	-1.26
10	99211890	99211890	ZDHHC16	zinc finger, DHHC-type conta	NM_032327.3	C	A	nonsynonymous SNV	C287A	A96D	M2	T	P	4.64
11	1263899	1263899	MUC5B	mucin 5B, oligomeric mucus/	NM_002458	T	A	nonsynonymous SNV	T5789A	L1930Q	M2	D	B	-1.43
11	128839734	128839734	ARHGAP32	Rho GTPase activating prote	NM_001142685.1	G	A	nonsynonymous SNV	C5332T	P1778S	M2	D	P	4.95
11	13443375	13443375	BTBD10	BTB (POZ) domain containin	NM_001297742.1	G	A	nonsynonymous SNV	C136T	R46C	M2	D	B	5.11
11	55595510	55595510	OR5L2	olfactory receptor, family 5, su	NM_001004739	A	T	nonsynonymous SNV	A816T	K272N	M2	D	D	-5.35
11	73022316	73022316	ARHGEF17	Rho guanine nucleotide exch	NM_014786	C	T	nonsynonymous SNV	C2633T	A878V	M2	T	B	2.77
12	123812357	123812357	SBNO1	strawberry notch homolog 1 (	NM_001167856.1	C	A	nonsynonymous SNV	G1435T	A479S	M2	D	D	6.03
12	50499359	50499359	GPD1	glycerol-3-phosphate dehydro	NM_005276	C	A	nonsynonymous SNV	C248A	A83E	M2	D	D	4.47
12	52981518	52981518	KRT72	keratin 72, type II	NM_080747.2	G	C	nonsynonymous SNV	C1207G	L403V	M2	D	D	4.92
12	56090158	56090158	ITGA7	integrin, alpha 7	NM_001144996.1	G	C	nonsynonymous SNV	C1808G	T603S	M2	T	B	-0.461
12	6493830	6493830	LTBR	lymphotoxin beta receptor (TI	NM_002342.2	G	A	nonsynonymous SNV	G173A	C58Y	M2	D	D	5.06
13	101742049	101742049	NALCN	sodium leak channel, non sel	NM_052867	C	T	nonsynonymous SNV	G3454A	V1152I	M2	T	P	5.77
13	28498647	28498647	PDX1	pancreatic and duodenal hom	NM_000209	G	C	nonsynonymous SNV	G661C	A221P	M2	T	B	1.87
14	100377911	100377911	EML1	echinoderm microtubule asso	NM_001008707.1	G	T	stopgain	G1549T	E517X	M2	D		5.72
14	102483555	102483555	DYNC1H1	dynein, cytoplasmic 1, heavy	NM_001376	T	G	nonsynonymous SNV	T7979G	V2660G	M2	D	D	5.46
15	58467216	58467216	AQP9	aquaporin 9	NM_020980	C	T	nonsynonymous SNV	C476T	A159V	M2	T	B	-2.17
15	71125439	71125439	LARP6	La ribonucleoprotein domain	NM_018357	C	A	nonsynonymous SNV	G428T	R143L	M2	T	D	4.68
15	89400137	89400137	ACAN	aggrecan	NM_013227.3	A	T	nonsynonymous SNV	A4321T	T1441S	M2	T	B	-1.44
16	27460236	27460236	IL21R	interleukin 21 receptor	NM_181078.2	C	A	nonsynonymous SNV	C1249A	P417T	M2	T	B	2.13
17	10368822	10368822	MYH4	myosin, heavy chain 4, skelet	NM_017533	G	A	nonsynonymous SNV	C442T	R148C	M2	D	D	4.95
17	18397545	18397545	LGALS9C	lectin, galactoside-binding, so	NM_001040078	T	A	stopgain	T935A	L312X	M2	T		0.817
17	34072954	34072954	GAS2L2	growth arrest-specific 2 like 2	NM_139285	C	T	nonsynonymous SNV	G1562A	G521D	M2	D	B	3.73
17	39538608	39538608	KRT34	keratin 34, type I	NM_021013	G	C	nonsynonymous SNV	C17G	P6R	M2	D	B	4.85
17	41165636	41165636	IFI35	interferon-induced protein 35	NM_005533	G	T	nonsynonymous SNV	G525T	E175D	M2	T	P	-4.89
18	2688669	2688669	SMCHD1	structural maintenance of chr	NM_015295	T	G	nonsynonymous SNV	T797G	V266G	M2	D	P	5.07
18	44560214	44560214	TCEB3B	transcription elongation facto	NM_016427	G	C	stopgain	C1422G	Y474X	M2	T		1.74

18	50278534	50278534	DCC	DCC netrin 1 receptor	NM_005215	G	A	nonsynonymous SNV	G202A	G68R	M2	D	B	5.52
19	45656956	45656956	NKPD1	NTPase, KAP family P-loop c	NM_198478	C	G	nonsynonymous SNV	G739C	G247R	M2	T	P	5.09
2	160994116	160994116	ITGB6	integrin, beta 6	NM_000888.4	G	T	nonsynonymous SNV	C1489A	L497M	M2	T	B	1.03
2	239177587	239177587	PER2	period circadian clock 2	NM_022817	C	A	nonsynonymous SNV	G801T	E267D	M2	T	D	3.04
20	33028122	33028122	ITCH	itchy E3 ubiquitin protein ligas	NM_001257137.1	C	A	nonsynonymous SNV	C965A	P322H	M2	D	B	5.19
21	15599340	15599340	RBM11	RNA binding motif protein 11	NM_144770	A	G	nonsynonymous SNV	A572G	H191R	M2	T	B	0.568
3	100038002	100038002	TBC1D23	TBC1 domain family, membe	NM_001199198.2	C	A	nonsynonymous SNV	C1777A	H593N	M2		B	6.06
3	111312874	111312874	ZBED2	zinc finger, BED-type contain	NM_024508	C	A	stopgain	G175T	E59X	M2	T		1.77
3	113378424	113378424	KIAA2018	KIAA2018	NM_001009899	T	A	nonsynonymous SNV	A2105T	N702I	M2	D	P	4.2
3	183028799	183028799	MCF2L2	MCF.2 cell line derived transf	NM_015078	A	C	nonsynonymous SNV	T897G	D299E	M2	T	B	4.37
3	186961288	186961288	MASP1	mannan-binding lectin serine	NM_001879.5	C	A	nonsynonymous SNV	G1212T	M404I	M2	T	B	4.69
3	193132524	193132524	ATP13A4	ATPase type 13A4	NM_032279	G	T	nonsynonymous SNV	C2858A	A953D	M2	D	D	5.15
4	190873403	190873403	FRG1	FSHD region gene 1	NM_004477	G	A	nonsynonymous SNV	G220A	D74N	M2	T	B	3.47
4	55599320	55599320	KIT	v-kit Hardy-Zuckerman 4 felir	M_000222	G	T	nonsynonymous SNV	G2446T	D816Y	M2	D	D	5.62
5	176520204	176520204	FGFR4	Fibroblast growth factor recep	NM_002011	T	C	nonsynonymous SNV	T1123C	S375P	M2	D	B	4.98
5	180335752	180335752	BTNL8	Butyrophilin-like 8	NM_001040462	C	A	nonsynonymous SNV	C216A	D72E	M2	T	B	-1.34
5	64521977	64521977	ADAMTS6	ADAM metallopeptidase with	NM_197941	G	A	nonsynonymous SNV	C2003T	A668V	M2	T	D	5.2
6	29408000	29408000	OR10C1	Olfactory receptor, family 10,	NM_013941	A	T	nonsynonymous SNV	A208T	I70F	M2	D	D	2.39
6	43230895	43230895	TTBK1	Tau Tubulin Kinase 1	NM_032538	G	A	nonsynonymous SNV	G1793A	R598H	M2	D	D	5.29
7	100645959	100645959	MUC12	Mucin 12, Cell Surface Assoc	NM_001164462	G	T	nonsynonymous SNV	G12115T	D4039Y	M2	D		-1.72
7	100677245	100677245	MUC17	Mucin 17, Cell Surface Assoc	NM_001040105	G	T	nonsynonymous SNV	G2548T	A850S	M2	T	B	-2.29
7	151970951	151970951	KMT2C	Lysine (K)-Specific Methyltrar	NM_170606	C	T	nonsynonymous SNV	G851A	R284Q	M2	D	P	4.87
7	33044951	33044951	FKBP9	FK506 binding protein 9, 63 k	NM_001284341	C	G	nonsynonymous SNV	C1860G	H620Q	M2	D	D	-3.51
7	34192846	34192846	BMPER	BMP binding endothelial regu	NM_133468	G	T	nonsynonymous SNV	G2019T	K673N	M2	T	B	3.76
7	37890245	37890245	NME8	NME/NM23 family member 8	NM_016616	C	T	stopgain	C106T	Q36X	M2	D		5.01
7	45123066	45123066	NACAD	NAC alpha domain containing	NM_001146334	G	T	nonsynonymous SNV	C2713A	P905T	M2	T	B	-0.565
7	5105019	5105019	RBAK	RB-associated KRAB zinc fin	NM_021163	C	A	nonsynonymous SNV	C1932A	S644R	M2		D	3.68
7	55240716	55240716	EGFR	Epidermal growth factor	NM_005228	C	A	nonsynonymous SNV	C1960A	L654I	M2	T	B	4.78
7	73474285	73474285	ELN	elastin	NM_001278939	T	C	nonsynonymous SNV	T1571C	V524A	M2	T	B	-2.18
8	52732961	52732961	PCMTD1	Protein-L-isoaspartate (D-asp	NM_052937	G	A	nonsynonymous SNV	C1024T	P342S	M2	D	D	6.07
9	125486686	125486686	OR1L4	Olfactory Receptor, Family 1,	NM_001005235	T	C	nonsynonymous SNV	T418C	W140R	M2	T	B	-0.339
9	33385103	33385103	AQP7	Aquaporin 7	NM_001170	G	A	nonsynonymous SNV	C929T	T310M	M2	T	B	-7.4
X	49179739	49179739	GAGE12J	G antigen 12J	NM_001098406	A	G	nonsynonymous SNV	A67G	M23V	M2	T	B	-1.36
19	49558211	49558211	CGB7	Chorionic Gonadotropin, Beta	NM_033142	T	G	nonsynonymous SNV	A70C	M24L	M2			946
7	100645960	100645960	MUC12	Mucin12	NM_001164462	A	T	nonsynonymous SNV	A12116T	D4039V	M2	T		-1.72
7	57529086	57529086	ZNF716	Zinc Finger Protein 716	NM_001159279	C	A	nonsynonymous SNV	C919A	R307S	M2	T	B	-218
17	7483366	7483366	CD68	CD68 Molecule	NM_001251	T	A	nonsynonymous SNV	T288A	N96K	M2	T	B	-5.96
11	618683	618683	CDHR5	Cadherin-Related Family Mer	NM_021924	T	A	nonsynonymous SNV	A1876T	T626S	M2	T	B	592
11	77614590	77614590	INTS4	Integrator Complex Subunit 4	NM_033547	T	G	nonsynonymous SNV	A2093C	K698T	M2	T	B	4.41
7	100639761	100639761	MUC12	Mucin12	NM_001164462	G	C	nonsynonymous SNV	G5917C	D1973H	M2	T		-275
3	195452870	195452870	MUC20	Mucin20	NM_001282506	G	A	nonsynonymous SNV	G1396A	E466K	M2	T	B	2.47
13	28498644	28498644	PDX1	Pancreatic And Duodenal Ho	NM_000209	G	C	nonsynonymous SNV	G658C	V220L	M2	T	B	3.12
11	618667	618667	CDHR5	Cadherin-Related Family Mer	NM_021924	C	A	nonsynonymous SNV	G1892T	G631V	M2	D	B	446
19	49558216	49558216	CGB7	Chorionic Gonadotropin, Beta	NM_033142	C	T	nonsynonymous SNV	G65A	R22K	M2	T		-353
14	23828948	23828948	EFS	embryonal Fyn-associated su	NM_005864	C	G	nonsynonymous SNV	G739C	G247R	M2	T	B	2.64
8	2092844	2092844	MYOM2	myomesin 2	NM_003970	C	G	nonsynonymous SNV	C4337G	A1446G	M2	T	B	-1.53
1	145296373	145296373	NBPF10	Neuroblastoma Breakpoint Fa	M_001039703	G	T	nonsynonymous SNV	G295T	V99F	M2	D		-158
1	248801610	248801610	OR2T35	Olfattory Receptor Family 2, S	NM_001001827	G	A	nonsynonymous SNV	C950T	A317V	M2	T	B	-3.53
1	227842419	227842419	ZNF678	Zinc Finger Protein 678	NM_178549	C	A	nonsynonymous SNV	C633A	D211E	M2	T	B	-2.68
1	110766454	110766454	KCNC4	potassium channel, voltage g	NM_004978.4	G	A	nonsynonymous SNV	G1547A	R516Q	M4	T	P	4.89
1	111717566	111717566	CEPT1	choline/ethanolamine phosph	NM_006090.3	C	A	nonsynonymous SNV	C695A	P232Q	M4	T	P	5.98
1	11561164	11561164	PTCHD2	patched domain containing 2	NM_020780	G	A	nonsynonymous SNV	G115A	G39R	M4	T	B	4.85
1	11574454	11574454	PTCHD2	patched domain containing 2	NM_020780	C	T	stopgain	C1324T	Q442X	M4	T		5.03
1	120484365	120484365	NOTCH2	notch 2	NM_024408	T	C	nonsynonymous SNV	A2765G	N922S	M4	D	D	6.08
1	12202548	12202548	TNFRSF8	tumor necrosis factor recepto	NM_001243.4	G	A	nonsynonymous SNV	G1748A	G583E	M4	D	D	5.51
1	144879542	144879542	PDE4DIP	phosphodiesterase 4D intera	NM_014644.5	A	G	nonsynonymous SNV	T3908C	L1303P	M4	T	B	2.15
1	150958836	150958836	ANXA9	annexin A9	NM_003568	A	G	nonsynonymous SNV	A497G	D166G	M4	D	D	5.1
1	151372145	151372145	PSMB4	proteasome (prosome, macro	NM_002796	C	T	nonsynonymous SNV	C82T	P28S	M4	T	B	0.222
1	151497202	151497202	CGN	cingulin	NM_020770	G	A	nonsynonymous SNV	G1454A	R485Q	M4	D	B	3.95
1	152081921	152081921	TCHH	trichohyalin	NM_007113	G	C	nonsynonymous SNV	C3772G	L1258V	M4	T	B	3.14
1	152129087	152129087	RPTN	repetin	NM_001122965	T	C	nonsynonymous SNV	A488G	K163R	M4	T	B	-4.06
1	152186699	152186699	HRNR	hornerin	NM_001009931	T	A	nonsynonymous SNV	A7406T	Q2469L	M4	T	P	1.18
1	152186766	152186766	HRNR	hornerin	NM_001009931	G	A	nonsynonymous SNV	C7339T	R2447C	M4	T	B	-4.42
1	152187935	152187935	HRNR	hornerin	NM_001009931	C	T	nonsynonymous SNV	G6170A	R2057Q	M4	T	B	-2.34
1	152187945	152187945	HRNR	hornerin	NM_001009931	C	T	nonsynonymous SNV	G6160A	G2054S	M4	T	B	-8.59
1	152188176	152188176	HRNR	hornerin	NM_001009931	G	A	nonsynonymous SNV	C5929T	R1977C	M4	T	P	-1.91
1	152191709	152191709	HRNR	hornerin	NM_001009931	C	G	nonsynonymous SNV	G2396C	S799T	M4	T	B	2.17

1	152192114	152192114	HRNR	hornerin	NM_001009931	C	T	nonsynonymous SNV	G1991A	R664Q	M4	T	B	-3.67
1	152192631	152192631	HRNR	hornerin	NM_001009931	C	T	nonsynonymous SNV	G1474A	G492R	M4	T	B	-3.36
1	152193286	152193286	HRNR	hornerin	NM_001009931	G	T	nonsynonymous SNV	C819A	H273Q	M4	T	B	-8.81
1	152193851	152193851	HRNR	hornerin	NM_001009931	C	T	nonsynonymous SNV	G254A	R85H	M4	T	B	0.954
1	152281639	152281639	FLG	filaggrin	NM_002016	G	A	nonsynonymous SNV	C5723T	P1908L	M4	D	P	1.84
1	15392193	15392193	KAZN	kazrin, periplakin interacting p	NM_201628	C	T	nonsynonymous SNV	C1166T	S389F	M4	D	D	5.27
1	156642239	156642239	NES	nestin	NM_006617	C	T	nonsynonymous SNV	G1741A	E581K	M4	D	B	4.28
1	158368846	158368846	OR10T2	olfactory receptor, family 10, s	NM_001004475	T	C	nonsynonymous SNV	A411G	I137M	M4	T	B	2.64
1	158435686	158435686	OR10K1	olfactory receptor, family 10, s	NM_001004473	C	T	nonsynonymous SNV	C335T	S112F	M4	D	D	4.5
1	16534022	16534022	ARHGEF19	Rho guanine nucleotide exch	NM_153213	A	G	nonsynonymous SNV	T869C	L290P	M4	T	B	4.9
1	1685785	1685785	NADK	NAD kinase	NM_023018.4	G	A	nonsynonymous SNV	C881T	S294F	M4	D	D	5.66
1	169390783	169390783	CCDC181	coiled-coil domain containing	NM_001300968.1	G	A	nonsynonymous SNV	C886T	L296F	M4	D	B	-3.33
1	175092674	175092674	TNN	tenascin N	NM_022093	C	T	nonsynonymous SNV	C2789T	P930L	M4	T	P	3.1
1	17668609	17668609	PADI4	peptidyl arginine deiminase, t	NM_012387	C	T	nonsynonymous SNV	C824T	S275F	M4	D	P	4.63
1	177909791	177909791	SEC16B	SEC16 homolog B, endoplas	NM_033127	C	T	nonsynonymous SNV	G2081A	R694K	M4	T	B	-2.19
1	183616926	183616926	APOBEC4	apolipoprotein B mRNA editir	NM_203454	T	C	nonsynonymous SNV	A991G	K331E	M4	T	B	-0.467
1	18808056	18808056	KLHDC7A	kelch domain containing 7A	NM_152375	G	C	nonsynonymous SNV	G581C	R194P	M4	T	B	0.453
1	19465666	19465666	UBR4	ubiquitin protein ligase E3 co	NM_020765	G	A	nonsynonymous SNV	C8642T	T2881I	M4	D	D	5.96
1	198671531	198671531	PTPRC	protein tyrosine phosphatase	NM_002838	G	A	nonsynonymous SNV	G455A	R152K	M4	T	B	1.33
1	205890942	205890942	SLC26A9	solute carrier family 26 (anior	NM_052934.3	C	T	nonsynonymous SNV	G1807A	E603K	M4	T	B	4.23
1	209950760	209950760	TRAF3IP3	TRAF3 interacting protein 3	NM_025228.3	C	G	nonsynonymous SNV	C1117G	Q373E	M4	T	B	5.56
1	21024901	21024901	KIF17	kinesin family member 17	NM_020816.3	C	T	nonsynonymous SNV	G1204A	V402M	M4	T	B	2.87
1	216348764	216348764	USH2A	Usher syndrome 2A (autoson	NM_206933	C	T	nonsynonymous SNV	G4457A	R1486K	M4	T	B	-2.24
1	22456342	22456342	WNT4	wingless-type MMTV integrat	NM_030761	T	G	nonsynonymous SNV	A80C	Y27S	M4	T	B	5.22
1	225555602	225555602	DNAH14	dynein, axonemal, heavy cha	NM_001373	T	G	nonsynonymous SNV	T11907G	N3969K	M4	T	B	-10.1
1	227920103	227920103	JMJD4	jumonji domain containing 4	NM_023007.2	G	A	nonsynonymous SNV	C1382T	A461V	M4	T	B	-2.14
1	236718620	236718620	HEATR1	HEAT repeat containing 1	NM_018072	T	C	nonsynonymous SNV	A5899G	N1967D	M4	T	B	5.3
1	240370333	240370333	FMN2	formin 2	NM_020066	G	A	nonsynonymous SNV	G2221A	A741T	M4	D	B	5.06
1	245848996	245848996	KIF26B	kinesin family member 26B	NM_018012	G	A	nonsynonymous SNV	G2711A	R904Q	M4	T	B	4.56
1	248202344	248202344	OR2L2	olfactory receptor, family 2, su	NM_001004686	G	C	nonsynonymous SNV	G775C	V259L	M4	T	B	1.9
1	248512786	248512786	OR14C36	olfactory receptor, family 14, s	NM_001001918	C	T	nonsynonymous SNV	C710T	S237F	M4	D	D	3.91
1	249141516	249141516	ZNF672	zinc finger protein 672	NM_024836	T	G	nonsynonymous SNV	T43G	S15A	M4	T	B	-6.85
1	33402499	33402499	RNF19B	ring finger protein 19B	NM_153341.3	C	T	nonsynonymous SNV	G2107A	G703S	M4	T	B	5.14
1	34663411	34663411	C1orf94	chromosome 1 open reading	NM_001134734.1	C	G	nonsynonymous SNV	C906G	D302E	M4	T	B	1.31
1	39879517	39879517	KIAA0754	KIAA0754	NM_015038	G	A	nonsynonymous SNV	G3580A	E1194K	M4	T	B	0.067
1	39917945	39917945	MACF1	microtubule-actin crosslinking	NM_012090	G	A	nonsynonymous SNV	G14359A	E4787K	M4	D	D	5.98
1	40980559	40980559	EXO5	exonuclease 5	NM_022774	G	A	nonsynonymous SNV	G343A	D115N	M4	T	B	3.65
1	43212926	43212926	LEPRE1	prolyl 3-hydroxylase 1	NM_001243246.1	G	A	nonsynonymous SNV	C2072T	A691V	M4	T		0
1	43906238	43906238	SZT2	seizure threshold 2 homolog	NM_015284	G	C	nonsynonymous SNV	G7154C	R2385P	M4			
1	46500993	46500993	MAST2	microtubule associated serine	NM_015112	A	G	nonsynonymous SNV	A4652G	D1551G	M4	T	B	1.67
1	47607840	47607840	CYP4A22	cytochrome P450, family 4, s	NM_001010969	C	A	nonsynonymous SNV	C443A	P148Q	M4	D	D	1.7
1	47610065	47610065	CYP4A22	cytochrome P450, family 4, s	NM_001010969	A	C	nonsynonymous SNV	A827C	K276T	M4	T	P	1.51
1	6279370	6279370	RNF207	ring finger protein 207	NM_207396	G	C	nonsynonymous SNV	G1808C	G603A	M4	T	B	1.01
1	66384362	66384362	PDE4B	phosphodiesterase 4B, cAMF	NM_002600.3	G	A	nonsynonymous SNV	G125A	G42E	M4	T	B	5.6
1	75622666	75622666	LHX8	LIM homeobox 8	NM_001001933.1	C	T	nonsynonymous SNV	C899T	S300F	M4	D	P	5.12
1	7913430	7913430	UTS2	urotensin 2	NM_021995	G	A	nonsynonymous SNV	C62T	T21M	M4	T	B	-3.06
1	85666156	85666156	SYDE2	synapse defective 1, Rho GT	NM_032184	T	G	nonsynonymous SNV	A524C	Q175P	M4	T	B	-9.1
1	90180211	90180211	LRRC8C	leucine rich repeat containing	NM_032270	C	A	nonsynonymous SNV	C2082A	D694E	M4	T	B	4.02
1	92979484	92979484	EVI5	ecotropic viral integration site	NM_005665	G	A	nonsynonymous SNV	C2162T	P721L	M4	D	B	5.15
10	115409840	115409840	NRAP	nebulin-related anchoring pro	NM_198060.3	C	T	nonsynonymous SNV	G844A	A282T	M4	T	B	5.15
10	116032519	116032519	VWA2	von Willebrand factor A doma	NM_001272046	A	G	nonsynonymous SNV	A392G	E131G	M4	T	B	4.28
10	121429633	121429633	BAG3	BCL2-associated athanogene	NM_004281	T	C	nonsynonymous SNV	T451C	C151R	M4	T	B	0.064
10	15154802	15154802	NMT2	N-myristoyltransferase 2	NM_004808	G	T	nonsynonymous SNV	C1331A	A444D	M4	D	D	5.69
10	3202065	3202065	PITRM1	pitrilysin metallopeptidase 1	NM_001242307.1	T	C	nonsynonymous SNV	A982G	I328V	M4	T	B	0.303
10	38121131	38121131	ZNF248	zinc finger protein 248	NM_021045.2	T	A	nonsynonymous SNV	A1152T	E384D	M4	D	B	0.794
10	47665076	47665076	ANTXRL	anthrax toxin receptor-like	NM_001278688	G	A	nonsynonymous SNV	G332A	R111Q	M4			
10	49400730	49400730	FRMPD2	FERM and PDZ domain conta	NM_001018071	C	T	nonsynonymous SNV	G2162A	R721H	M4	T	B	0.934
10	61834170	61834170	ANK3	ankyrin 3, node of Ranvier (a	NM_020987	G	A	nonsynonymous SNV	C6469T	P2157S	M4	T	D	6.07
10	70748784	70748784	KIAA1279	KIF1 Binding Protein	NM_015634	G	A	nonsynonymous SNV	G196A	G66S	M4	T	B	-0.111
10	73121913	73121913	SLC29A3	solute carrier family 29 (equili	NM_018344	A	G	nonsynonymous SNV	A976G	I326V	M4	T	B	-9
10	75415677	75415677	SYNPO2L	synaptopodin 2-like	NM_001114133	C	T	nonsynonymous SNV	G4A	G2S	M4	D	P	4.53
10	96039597	96039597	PLCE1	phospholipase C, epsilon 1	NM_016341.3	G	C	nonsynonymous SNV	G4724C	R1575P	M4	T	B	4.64
10	97983721	97983721	BLNK	B-cell linker	NM_013314.3	G	C	nonsynonymous SNV	C386G	S129C	M4	T	P	5.1
10	99192229	99192229	PGAM1	phosphoglycerate mutase 1 (	NM_002629	C	T	nonsynonymous SNV	C713T	T238M	M4			
10	99332223	99332223	ANKRD2	ankyrin repeat domain 2 (stre	NM_001291218.1	C	G	nonsynonymous SNV	C17G	P6R	M4			
11	104869708	104869708	CASP5	caspase 5, apoptosis-related	NM_004347.3	G	C	nonsynonymous SNV	C1000G	L334V	M4	T	B	-4.68
11	109294657	109294657	C11orf87	chromosome 11 open reading	NM_207645	G	A	nonsynonymous SNV	G298A	E100K	M4	D	D	4.92

11	11374269	11374269	CSNK2A3	casein kinase 2, alpha 3 poly	NM_001256686	A	G	nonsynonymous SNV	T398C	I133T	M4			
11	116691675	116691675	APOA4	apolipoprotein A-IV	NM_000482	T	A	nonsynonymous SNV	A1099T	T367S	M4	T	B	0.012
11	117864063	117864063	IL10RA	interleukin 10 receptor, alpha	NM_001558	A	G	nonsynonymous SNV	A475G	S159G	M4	T	B	2.05
11	118221350	118221350	CD3G	CD3g molecule, gamma (CD:	NM_000073	G	T	nonsynonymous SNV	G391T	V131F	M4	T	P	1.63
11	119005132	119005132	HINFP	histone H4 transcription facto	NM_015517.4	C	T	nonsynonymous SNV	C1478T	A493V	M4	T	B	-0.697
11	123777497	123777497	OR8D4	olfactory receptor, family 8, su	NM_001005197	G	A	nonsynonymous SNV	G359A	C120Y	M4	T	B	4.66
11	123848121	123848121	OR10S1	olfactory receptor, family 10, :	NM_001004474	T	C	nonsynonymous SNV	A278G	K93R	M4	T	P	3.69
11	123848212	123848212	OR10S1	olfactory receptor, family 10, :	NM_001004474	C	T	nonsynonymous SNV	G187A	G63S	M4	T	B	1.9
11	123901192	123901192	OR10G8	olfactory receptor, family 10, :	NM_001004464	C	T	nonsynonymous SNV	C863T	T288I	M4	D	D	2.91
11	124095525	124095525	OR8G2	olfactory receptor, family 8, su	NM_001291438	T	C	nonsynonymous SNV	T128C	L43P	M4			
11	124095647	124095647	OR8G2	olfactory receptor, family 8, su	NM_001291438	A	C	nonsynonymous SNV	A250C	I84L	M4			
11	126162843	126162843	TIRAP	toll-interleukin 1 receptor (TIF	NM_148910.2	C	T	nonsynonymous SNV	C539T	S180L	M4		B	4.71
11	1264691	1264691	MUC5B	mucin 5B, oligomeric mucus/(	NM_002458	T	C	nonsynonymous SNV	T6581C	M2194T	M4	D	B	-4.47
11	1265375	1265375	MUC5B	mucin 5B, oligomeric mucus/(	NM_002458	C	T	nonsynonymous SNV	C7265T	S2422F	M4	D	B	1.28
11	1265987	1265987	MUC5B	mucin 5B, oligomeric mucus/(	NM_002458	C	G	nonsynonymous SNV	C7877G	T2626R	M4	D	P	0.116
11	18955941	18955941	MRGPRX1	MAS-related GPR, member X	NM_147199	G	T	nonsynonymous SNV	C391A	R131S	M4	T	D	2.28
11	4608542	4608542	OR52I2	olfactory receptor, family 52, :	NM_001005170	C	T	nonsynonymous SNV	C500T	T167M	M4	D	D	3.26
11	4928841	4928841	OR51A7	olfactory receptor, family 51, :	NM_001004749	T	C	nonsynonymous SNV	T242C	M81T	M4	T	B	3.86
11	4976291	4976291	OR51A2	olfactory receptor, family 51, :	NM_001004748	G	A	nonsynonymous SNV	C653T	S218F	M4	D	D	3.13
11	5173149	5173149	OR52A1	olfactory receptor, family 52, :	NM_012375	C	A	nonsynonymous SNV	G451T	V151L	M4	T	B	-4.13
11	5247947	5247947	HBB	hemoglobin, beta	NM_000518	G	A	nonsynonymous SNV	C175T	P59S	M4	D	B	-8.5
11	55035837	55035837	TRIM48	tripartite motif containing 48	NM_024114	C	A	nonsynonymous SNV	C567A	D189E	M4	T	B	0
11	56128474	56128474	OR8J1	olfactory receptor, family 8, su	NM_001005205	T	G	nonsynonymous SNV	T752G	F251C	M4	D	D	3.9
11	56185327	56185327	OR5R1	olfactory receptor, family 5, su	NM_001004744	T	C	nonsynonymous SNV	A382G	S128G	M4	D	B	4.66
11	5863013	5863013	OR52E6	olfactory receptor, family 52, :	NM_001005167	T	C	nonsynonymous SNV	A115G	I39V	M4	T	B	-3.21
11	61032665	61032665	VWCE	von Willebrand factor C and E	NM_152718	G	A	nonsynonymous SNV	C1985T	S662F	M4	T	D	4.88
11	62520035	62520035	ZBTB3	zinc finger and BTB domain c	NM_024784	C	T	nonsynonymous SNV	G1252A	D418N	M4	T	B	4.51
11	64116842	64116842	CCDC88B	coiled-coil domain containing	NM_032251	G	T	nonsynonymous SNV	G2656T	D886Y	M4	D	B	1.69
11	64453213	64453213	NRXN2	neurexin 2	NM_015080.3	C	T	nonsynonymous SNV	G1057A	G353S	M4	T	D	4
11	64981837	64981837	SLC22A20	solute carrier family 22, mem	NM_001004326	C	T	nonsynonymous SNV	C422T	A141V	M4			
11	65629934	65629934	MUS81	MUS81 structure-specific enc	NM_025128	G	C	nonsynonymous SNV	G539C	R180P	M4	T	B	-1.98
11	67288594	67288594	CABP2	calcium binding protein 2	NM_016366	C	T	nonsynonymous SNV	G281A	R94Q	M4	D	B	3.61
11	74413955	74413955	CHRDL2	chordin-like 2	NM_015424.5	G	A	nonsynonymous SNV	C1004T	P335L	M4	T	D	4.15
11	830120	830120	CRACR2B	calcium release activated cha	NM_001286606.1	A	G	nonsynonymous SNV	A593G	Q198R	M4	T	B	3.27
11	8751889	8751889	ST5	suppression of tumorigenicity	NM_005418.3	C	G	nonsynonymous SNV	G948C	K316N	M4	D	P	6.17
11	93122196	93122196	CCDC67	coiled-coil domain containing	NM_181645	T	G	nonsynonymous SNV	T946G	S316A	M4	T	P	5.95
12	102591388	102591388	PMCH	pro-melanin-concentrating hc	NM_002674	G	A	nonsynonymous SNV	C161T	A54V	M4	T	B	2.99
12	10275922	10275922	CLEC7A	C-type lectin domain family 7	NM_197947.2	G	A	nonsynonymous SNV	C533T	S178L	M4	T	D	4.34
12	109017898	109017898	SELPLG	selectin P ligand	NM_001206609.1	C	T	nonsynonymous SNV	G234A	M78I	M4	T	B	-1.84
12	114380167	114380167	RBM19	RNA binding motif protein 19	NM_001146699.1	G	A	nonsynonymous SNV	C1699T	L567F	M4	T	D	4.48
12	11545921	11545921	PRB2	proline-rich protein BstNI sub	NM_006248	C	T	nonsynonymous SNV	G1091A	G364E	M4	T	P	1.46
12	121175678	121175678	ACADS	acyl-CoA dehydrogenase, C-:	NM_000017	C	T	nonsynonymous SNV	C511T	R171W	M4	D	P	2.1
12	126003996	126003996	TMEM132B	transmembrane protein 132B	NM_052907	C	T	nonsynonymous SNV	C1103T	S368F	M4	T	D	4.94
12	128899781	128899781	TMEM132C	transmembrane protein 132C	NM_001136103	A	C	nonsynonymous SNV	A590C	E197A	M4	T	P	3.93
12	133252086	133252086	POLE	polymerase (DNA directed), e	NM_006231	C	T	nonsynonymous SNV	G1124A	R375Q	M4	D	D	5.91
12	133502439	133502439	ZNF605	zinc finger protein 605	NM_183238.3	G	T	stopgain	C1446A	C482X	M4	T		0.608
12	133780878	133780878	ZNF268	zinc finger protein 268	NM_003415.2	G	A	nonsynonymous SNV	G2606A	C482X	M4		B	3.4
12	22659723	22659723	C2CD5	C2 calcium-dependent domai	NM_001286176.1	G	A	nonsynonymous SNV	C1069T	P357S	M4	D	D	5.25
12	2794921	2794921	CACNA1C	calcium channel, voltage-dep	NM_199460.3	G	A	nonsynonymous SNV	G5842A	E1948K	M4	T	D	3.35
12	2794937	2794937	CACNA1C	calcium channel, voltage-dep	NM_199460.3	C	T	nonsynonymous SNV	C5858T	T1953M	M4	T	P	4.26
12	30877346	30877346	CAPRIN2	caprin family member 2	NM_001002259.2	G	A	nonsynonymous SNV	C1945T	P649S	M4	T	B	3.27
12	32134064	32134064	KIAA1551	KIAA1551	NM_018169	A	G	nonsynonymous SNV	A175G	I59V	M4		B	-2.22
12	39752071	39752071	KIF21A	kinesin family member 21A	NM_001173464.1	A	C	nonsynonymous SNV	T1124G	V375G	M4	D	D	4.33
12	48723595	48723595	H1FNT	H1 histone family, member N	NM_181788	G	A	nonsynonymous SNV	G521A	R174Q	M4	T	B	-1.27
12	48921837	48921837	OR8S1	olfactory receptor, family 8, su	NM_001005203	G	A	nonsynonymous SNV	G1031A	G344E	M4	D	D	0.158
12	49724412	49724412	TROAP	trophinin associated protein	NM_005480	T	G	nonsynonymous SNV	T1784G	I595S	M4	T	B	0.336
12	50188714	50188714	NCKAP5L	NCK-associated protein 5-like	NM_001037806	C	T	nonsynonymous SNV	G2929A	D977N	M4	T	D	5.23
12	50189475	50189475	NCKAP5L	NCK-associated protein 5-like	NM_001037806	C	G	nonsynonymous SNV	G2168C	G723A	M4	T	B	2.75
12	51069183	51069183	DIP2B	disco-interacting protein 2 ho	NM_173602	C	T	nonsynonymous SNV	C868T	P290S	M4	D	D	5.03
12	52710721	52710721	KRT83	keratin 83, type II	NM_002282	G	C	nonsynonymous SNV	C837G	I279M	M4	D	P	-0.295
12	52713088	52713088	KRT83	keratin 83, type II	NM_002282	G	A	nonsynonymous SNV	C445T	R149C	M4	D	B	4.39
12	53167395	53167395	KRT76	keratin 76, type II	NM_015848	C	T	nonsynonymous SNV	G847A	A283T	M4	D	D	4.48
12	54757512	54757512	GPR84	G protein-coupled receptor 84	NM_020370	G	T	nonsynonymous SNV	C124A	L42I	M4	T	B	3.93
12	55794703	55794703	OR6C65	olfactory receptor, family 6, su	NM_001005518	A	G	nonsynonymous SNV	A391G	T131A	M4	T	B	2.36
12	56846903	56846903	MIP	major intrinsic protein of lens	NM_012064	G	A	nonsynonymous SNV	C559T	R187C	M4	D	D	4.96
12	58190185	58190185	TSFM	Ts translation elongation facto	NM_001172696.1	T	A	nonsynonymous SNV	T860A	L287H	M4	T	P	5.42
12	6172202	6172202	VWF	von Willebrand factor	NM_000552	T	C	nonsynonymous SNV	A1451G	H484R	M4	T	B	1.25

12	7842932	7842932	GDF3	growth differentiation factor 3	NM_020634	C	T	nonsynonymous SNV	G637A	G213R	M4	T	B	1.64
12	863152	863152	WNK1	WNK lysine deficient protein	NM_018979.3	G	A	nonsynonymous SNV	G421A	A141T	M4	T	B	4.35
12	93100895	93100895	C12orf74	chromosome 12 open readin	NM_001178097.2	T	A	nonsynonymous SNV	T488A	L163Q	M4	T	B	-0.926
12	9317784	9317784	PZP	pregnancy-zone protein	NM_002864	A	G	nonsynonymous SNV	T2438C	V813A	M4	T	B	-0.929
13	46170870	46170870	ERICH6B	glutamate-rich 6B	NM_182542	G	T	nonsynonymous SNV	C271A	H91N	M4	D	B	-5.63
14	105408811	105408811	AHNAK2	AHNAK nucleoprotein 2	NM_138420	A	G	nonsynonymous SNV	T12977C	L4326P	M4	T	B	1.41
14	105408827	105408827	AHNAK2	AHNAK nucleoprotein 2	NM_138420	A	C	nonsynonymous SNV	T12961G	L4321V	M4	T	B	-6.62
14	105408955	105408955	AHNAK2	AHNAK nucleoprotein 2	NM_138420	A	G	nonsynonymous SNV	T12833C	V4278A	M4	T	B	-0.353
14	105410411	105410411	AHNAK2	AHNAK nucleoprotein 2	NM_138420	C	T	nonsynonymous SNV	G11377A	D3793N	M4	T	B	1.37
14	92126184	92126184	CATSPERB	catsper channel auxiliary sub	NM_024764	C	T	nonsynonymous SNV	G1429A	A477T	M4	T	D	4.76
14	96777468	96777468	ATG2B	autophagy related 2B	NM_018036	G	C	nonsynonymous SNV	C4147G	Q1383E	M4	T	B	2.55
15	22382897	22382897	OR4N4	olfactory receptor, family 4, s	NM_001005241	A	G	nonsynonymous SNV	A425G	Y142C	M4	T	B	3.37
15	45968435	45968435	SQRDL	sulfide quinone reductase-like	NM_021199.3	T	C	nonsynonymous SNV	T791C	I264T	M4	T	B	4.62
15	55477605	55477605	RSL24D1	ribosomal L24 domain contai	NM_016304	A	G	nonsynonymous SNV	T287C	V96A	M4	D	B	5.05
15	79189365	79189365	MORF4L1	mortality factor 4 like 1	NM_006791.3	G	A	nonsynonymous SNV	G928A	D310N	M4	T	B	4.77
15	89401397	89401397	ACAN	aggrecan	NM_013227.3	G	A	nonsynonymous SNV	G5581A	G1861R	M4	T	P	5.56
16	10769958	10769958	TEKT5	tektin 5	NM_144674	T	C	nonsynonymous SNV	A944G	Q315R	M4	T	B	2.67
16	15178499	15178499	RRN3	RRN3 homolog, RNA polyme	NM_018427.4	G	A	nonsynonymous SNV	C596T	S199L	M4		B	3.86
16	21209105	21209105	ZP2	zona pellucida glycoprotein 2	NM_003460.2	C	T	nonsynonymous SNV	G2077A	E693K	M4	D	B	-1.38
16	3165425	3165425	ZNF205	zinc finger protein 205	NM_003456.2	A	G	nonsynonymous SNV	A127G	T43A	M4	T	B	-9.4
16	31927613	31927613	ZNF267	zinc finger protein 267	NM_003414.5	A	T	nonsynonymous SNV	A2043T	R681S	M4	D	B	0.468
16	31927643	31927643	ZNF267	zinc finger protein 267	NM_003414.5	T	A	nonsynonymous SNV	T2073A	D691E	M4	T	B	-0.935
16	334543	334543	PDIA2	protein disulfide isomerase fa	NM_006849	C	G	nonsynonymous SNV	C356G	T119R	M4	D	D	3.17
16	420140	420140	MRPL28	mitochondrial ribosomal prote	NM_006428	G	A	nonsynonymous SNV	C79T	H27Y	M4	T	D	4.71
16	4933939	4933939	PPL	periplakin	NM_002705	G	C	nonsynonymous SNV	C4717G	Q1573E	M4	T	D	5.83
16	69727038	69727038	NFAT5	nuclear factor of activated T-c	NM_138714.3	C	T	stopgain	C3028T	A591S	M4	D		5.83
16	88808743	88808743	PIEZO1	piezo-type mechanosensitive	NM_001142864	A	G	nonsynonymous SNV	T248C	I83T	M4	T	B	4.37
17	3336703	3336703	OR1E2	olfactory receptor, family 1, s	NM_003554	T	C	nonsynonymous SNV	A433G	M145V	M4	D	D	4.39
17	38911327	38911327	KRT25	keratin 25, type I	NM_181534	C	G	nonsynonymous SNV	G197C	G66A	M4	T	B	4.74
17	40860071	40860071	EZH1	enhancer of zeste 1 polycom	NM_001991	T	A	nonsynonymous SNV	A1565T	Q522L	M4	T	D	5.49
17	7414585	7414585	POLR2A	polymerase (RNA) II (DNA di	NM_000937	G	A	nonsynonymous SNV	G3865A	E1289K	M4	D	D	4.89
18	59221453	59221453	CDH20	cadherin 20, type 2	NM_031891	G	T	nonsynonymous SNV	G1931T	R644L	M4	D	P	4.86
18	59221485	59221485	CDH20	cadherin 20, type 2	NM_031891	G	A	nonsynonymous SNV	G1963A	E655K	M4	T	P	5.39
18	77728135	77728135	HSBP1L1	heat shock factor binding pro	NM_001136180	G	T	nonsynonymous SNV	G165T	K55N	M4	T	B	-2.31
18	9886840	9886840	TXNDC2	thioredoxin domain containin	NM_032243.5	G	T	nonsynonymous SNV	G163T	A55S	M4		B	-5.1
19	11517474	11517474	RGL3	ral guanine nucleotide dissoc	NM_001161616.2	C	T	nonsynonymous SNV	G704A	G235E	M4	T	B	-2.79
19	11917964	11917964	ZNF491	zinc finger protein 491	NM_152356	G	T	nonsynonymous SNV	G1196T	R399I	M4	T	B	-0.814
19	12060786	12060786	ZNF700	zinc finger protein 700	NM_001271848.1	A	T	nonsynonymous SNV	A1956T	E652D	M4	T	P	-0.438
19	13041287	13041287	FARSA	phenylalanyl-tRNA synthetas	NM_004461	G	A	nonsynonymous SNV	C340T	R114W	M4	T	B	4.34
19	15281250	15281250	NOTCH3	notch 3	NM_000435	C	T	nonsynonymous SNV	G5006A	R1669H	M4	D	B	3.69
19	18285944	18285944	IFI30	interferon, gamma-inducible p	NM_006332	G	A	nonsynonymous SNV	G227A	R76Q	M4	T	P	4.22
19	20045247	20045247	ZNF93	zinc finger protein 93	NM_031218	T	A	nonsynonymous SNV	T1483A	S495T	M4	T	B	-1.7
19	21300624	21300624	ZNF714	zinc finger protein 714	NM_182515	T	A	nonsynonymous SNV	T1154A	I385K	M4	T	B	-2.03
19	21476980	21476980	ZNF708	zinc finger protein 708	NM_021269.2	C	A	nonsynonymous SNV	G788T	R263L	M4	T	B	-2.1
19	21719658	21719658	ZNF429	zinc finger protein 429	NM_001001415	G	T	nonsynonymous SNV	G803T	R268M	M4	T	B	-1.75
19	21720265	21720265	ZNF429	zinc finger protein 429	NM_001001415	C	A	nonsynonymous SNV	C1410A	S470R	M4	T	B	-1.62
19	22847946	22847946	ZNF492	zinc finger protein 492	NM_020855	T	C	nonsynonymous SNV	T1475C	L492P	M4	D	D	1.06
19	2396611	2396611	TMPRSS9	transmembrane protease, se	NM_182973	C	T	nonsynonymous SNV	C217T	R73W	M4	D	P	2.94
19	2422191	2422191	TMPRSS9	transmembrane protease, se	NM_182973	G	A	nonsynonymous SNV	G2392A	G798R	M4	T	B	0.432
19	37367862	37367862	ZNF345	zinc finger protein 345	NM_003419.4	G	C	nonsynonymous SNV	G130C	D44H	M4	T	P	2.81
19	37734303	37734303	ZNF383	zinc finger protein 383	NM_152604	G	T	nonsynonymous SNV	G1165T	A389S	M4	D	B	2.58
19	41869392	41869392	B9D2	B9 protein domain 2	NM_030578	T	C	nonsynonymous SNV	A33G	I11M	M4	T	B	-1.13
19	4210356	4210356	ANKRD24	ankyrin repeat domain 24	NM_133475	G	A	nonsynonymous SNV	G1046A	R349Q	M4	T	B	-1.19
19	43097942	43097942	CEACAM8	carcinoembryonic antigen-rel	NM_001816	G	A	nonsynonymous SNV	C175T	P59S	M4	T	B	0.808
19	51412668	51412668	KLK4	kallikrein-related peptidase 4	NM_004917	A	C	nonsynonymous SNV	T64G	S22A	M4	T	B	-7.01
19	52658643	52658643	ZNF836	zinc finger protein 836	NM_001102657	G	A	nonsynonymous SNV	C2293T	L765F	M4	D	D	0.961
19	53572625	53572625	ZNF160	zinc finger protein 160	NM_001102603.1	A	T	nonsynonymous SNV	T1162A	W388R	M4	T	B	-4.94
19	53856444	53856444	ZNF845	zinc finger protein 845	NM_138374	C	A	nonsynonymous SNV	C2516A	T839N	M4	D	B	-2.93
19	54802554	54802554	LILRA3	leukocyte immunoglobulin-lik	NM_006865.4	G	C	nonsynonymous SNV	C887G	T296R	M4	T	B	-0.638
19	57133707	57133707	ZNF71	zinc finger protein 71	NM_021216	T	A	nonsynonymous SNV	T1052A	V351E	M4	T	B	1.28
19	57838018	57838018	ZNF543	zinc finger protein 543	NM_213598	C	G	nonsynonymous SNV	C163G	P55A	M4	T	B	0.225
19	57868483	57868483	ZNF304	zinc finger protein 304	NM_001290318.1	G	A	nonsynonymous SNV	G1387A	A463T	M4	D	B	2.96
19	57910591	57910591	ZNF548	zinc finger protein 548	NM_001172773.1	G	T	nonsynonymous SNV	G972T	R324S	M4	T	B	-4.63
19	5866724	5866724	FUT5	fucosyltransferase 5 (alpha (1	NM_002034	G	A	nonsynonymous SNV	C1013T	T338M	M4	T	B	0.912
19	5867177	5867177	FUT5	fucosyltransferase 5 (alpha (1	NM_002034	G	A	nonsynonymous SNV	C560T	P187L	M4	T	P	1.25
19	7810697	7810697	CD209	CD209 molecule	NM_021155.3	C	A	nonsynonymous SNV	G455T	W152L	M4	T	B	-2.22
19	7810759	7810759	CD209	CD209 molecule	NM_021155.3	C	A	nonsynonymous SNV	G393T	K131N	M4	T	D	-0.018

19	7810776	7810776	CD209	CD209 molecule	NM_021155.3	C	A	stopgain	G376T	E126X	M4	T		-2.75
19	9070402	9070402	MUC16	mucin 16, cell surface associ	NM_024690	G	T	nonsynonymous SNV	C17044A	P5682T	M4	D	P	-2.48
2	101670689	101670689	TBC1D8	TBC1 domain family, membe	NM_001102426	G	A	nonsynonymous SNV	C467T	A156V	M4	T	P	5.78
2	109382873	109382873	RANBP2	RAN binding protein 2	NM_006267	C	T	stopgain	C5878T	Q1960X	M4	T		4.86
2	11597313	11597313	E2F6	E2F transcription factor 6	NM_198256.3	G	A	nonsynonymous SNV	C155T	S52F	M4	D	P	5.08
2	119739929	119739929	MARCO	macrophage receptor with co	NM_006770	C	T	nonsynonymous SNV	C1006T	P336S	M4	0	B	4.07
2	1241772	1241772	SNTG2	syntrophin, gamma 2	NM_018968	G	A	nonsynonymous SNV	G832A	E278K	M4	T	B	0.28
2	128379563	128379563	MYO7B	myosin VIIB	NM_001080527	C	G	nonsynonymous SNV	C3454G	L1152V	M4	T	P	3.03
2	130899915	130899915	CCDC74B	coiled-coil domain containing	NM_207310	G	A	nonsynonymous SNV	C335T	P112L	M4	T	B	0.912
2	135893372	135893372	RAB3GAP1	RAB3 GTPase activating pro	NM_001172435.1	A	G	nonsynonymous SNV	A1793G	N598S	M4	T	B	4.26
2	141812777	141812777	LRP1B	low density lipoprotein recept	NM_018557	G	A	nonsynonymous SNV	C1460T	S487L	M4	D	P	5.45
2	149227700	149227700	MBD5	methyl-CpG binding domain p	NM_018328	T	A	nonsynonymous SNV	T2188A	L730M	M4	T	P	4.96
2	155711314	155711314	KCNJ3	potassium channel, inwardly	NM_002239	C	T	nonsynonymous SNV	C995T	S332F	M4	T	B	5.7
2	168107798	168107798	XIRP2	xin actin binding repeat conta	NM_152381.5	T	C	nonsynonymous SNV	T9896C	V3299A	M4	T	D	5.45
2	170857815	170857815	UBR3	ubiquitin protein ligase E3 co	NM_172070	G	T	nonsynonymous SNV	G3920T	S1307I	M4	T	D	5.05
2	174081896	174081896	ZAK	sterile alpha motif and leucine	NM_016653.2	G	A	nonsynonymous SNV	G905A	S302N	M4	T	D	5.59
2	17696870	17696870	RAD51AP2	RAD51 associated protein 2	NM_001099218	C	T	nonsynonymous SNV	G2813A	G938E	M4	T	P	2.46
2	179425090	179425090	TTN	titin	NM_001267550	C	T	nonsynonymous SNV	G85769A	R28590Q	M4	D	D	5.88
2	179429821	179429821	TTN	titin	NM_001267550	C	T	nonsynonymous SNV	G81038A	R27013Q	M4	D	B	5
2	179611941	179611941	TTN	titin	NM_133379	G	T	stopgain	C15186A	Y5062X	M4	T		-8
2	197541385	197541385	CCDC150	coiled-coil domain containing	NM_001080539	G	A	nonsynonymous SNV	G1370A	R457Q	M4	T	B	-0.936
2	207824384	207824384	CPO	carboxypeptidase O	NM_173077	T	G	nonsynonymous SNV	T402G	S134R	M4	T	B	1.12
2	21228827	21228827	APOB	apolipoprotein B	NM_000384	C	T	nonsynonymous SNV	G10913A	R3638Q	M4	T	B	3.24
2	21235019	21235019	APOB	apolipoprotein B	NM_000384	G	T	nonsynonymous SNV	C4721A	T1574N	M4	D	B	4.03
2	216190020	216190020	ATIC	5-aminoimidazole-4-carboxar	NM_004044	C	G	nonsynonymous SNV	C347G	T116S	M4	T	B	5.87
2	220039825	220039825	CNPPD1	cyclin Pas1/PHO80 domain c	NM_015680	G	T	nonsynonymous SNV	C266A	A89E	M4	D	D	4.43
2	220284876	220284876	DES	desmin	NM_001927	C	T	nonsynonymous SNV	C638T	A213V	M4	D	B	3.67
2	228243905	228243905	TM4SF20	transmembrane 4 L six family	NM_024795	G	A	nonsynonymous SNV	C80T	A27V	M4	T	B	1.78
2	242573211	242573211	THAP4	THAP domain containing 4	NM_015963	T	C	nonsynonymous SNV	A361G	S121G	M4	T	B	-1.86
2	242674803	242674803	D2HGDH	D-2-hydroxyglutarate dehydro	NM_152783	G	A	nonsynonymous SNV	G164A	R55Q	M4	T	B	-6.41
2	24431184	24431184	ITSN2	intersectin 2	NM_006277.2	C	T	nonsynonymous SNV	G4600A	A1534T	M4	T	D	4.86
2	25022598	25022598	CENPO	centromere protein O	NM_024322.2	A	G	nonsynonymous SNV	A101G	Q34R	M4	T	B	-3.63
2	25141538	25141538	ADCY3	adenylate cyclase 3	NM_004036	A	G	nonsynonymous SNV	T319C	S107P	M4	T	B	2.94
2	26700700	26700700	OTOF	otoferlin	NM_194322	G	A	nonsynonymous SNV	C62T	P21L	M4		B	-0.572
2	26725272	26725272	OTOF	otoferlin	NM_194248	C	T	nonsynonymous SNV	G606A	M202I	M4	T	B	5.52
2	27156126	27156126	DPYSL5	dihydropyrimidinase-like 5	NM_020134.3	A	G	nonsynonymous SNV	A715G	T239A	M4		B	5.66
2	54856669	54856669	SPTBN1	spectrin, beta, non-erythrocyt	NM_003128.2	C	T	nonsynonymous SNV	C2398T	L800F	M4	D	P	3.38
2	65245365	65245365	SLC1A4	solute carrier family 1 (glutam	NM_003038.4	G	A	nonsynonymous SNV	G1195A	V399I	M4		B	0.22
2	68364478	68364478	WDR92	WD repeat domain 92	NM_138458.3	T	C	nonsynonymous SNV	A721G	M241V	M4	T	B	4.81
2	71360282	71360282	MPHOSPH10	M-phase phosphoprotein 10 (	NM_005791	G	A	nonsynonymous SNV	G344A	R115H	M4	T	B	2.81
2	77746870	77746870	LRRTM4	eucine rich repeat transmemb	NM_001134745.1	C	T	nonsynonymous SNV	G125A	G42D	M4	T	D	5.72
2	85893741	85893741	SFTPB	surfactant protein B	NM_000542.3	G	A	nonsynonymous SNV	C428T	T143I	M4	T	B	3.33
2	9621490	9621490	IAH1	isoamyl acetate-hydrolyzing e	NM_001039613	C	T	nonsynonymous SNV	C359T	S120F	M4	D	D	5.75
2	9630645	9630645	ADAM17	ADAM metallopeptidase dom	NM_003183	G	T	nonsynonymous SNV	C2136A	N712K	M4	T	D	-8.4
2	96795608	96795608	ASTL	astacin-like metallo-endopept	NM_001002036	T	G	nonsynonymous SNV	A829C	K277Q	M4	T	B	-2.33
2	97873681	97873681	ANKRD36	ankyrin repeat domain 36	NM_001164315	C	A	nonsynonymous SNV	C3203A	A1068D	M4	T	B	-1.93
20	1600524	1600524	SIRPB1	signal-regulatory protein beta	NM_006065.3	T	C	nonsynonymous SNV	A67G	R23G	M4	T	B	0.15
20	23375794	23375794	NAPB	N-ethylmaleimide-sensitive fa	NM_001283018.1	G	C	nonsynonymous SNV	C336G	I112M	M4	D	P	5.61
20	47266060	47266060	PREX1	phosphatidylinositol-3,4,5-tris	NM_020820	G	A	nonsynonymous SNV	C3083T	P1028L	M4	D	D	4.6
20	60913300	60913300	LAMA5	laminin, alpha 5	NM_005560	G	A	nonsynonymous SNV	C1744T	P582S	M4	D	P	5.38
20	60986019	60986019	RBBP8NL	RBBP8 N-terminal like	NM_080833	A	C	nonsynonymous SNV	T1910G	L637R	M4	T	P	1.32
21	31802768	31802768	KRTAP13-4	keratin associated protein 13	NM_181600	G	A	nonsynonymous SNV	G175A	A59T	M4	T	B	-2.5
21	31869286	31869286	KRTAP19-4	keratin associated protein 19	NM_181610	T	C	nonsynonymous SNV	A143G	Y48C	M4	D	B	-4.2
21	32201835	32201835	KRTAP7-1	keratin associated protein 7-1	NM_181606	A	C	nonsynonymous SNV	T182G	I61S	M4			
21	32201866	32201866	KRTAP7-1	keratin associated protein 7-1	NM_181606	A	G	nonsynonymous SNV	T151C	S51P	M4			
21	37605228	37605228	DOPEY2	dopey family member 2	NM_005128	C	T	nonsynonymous SNV	C2477T	S826F	M4	D	D	5.98
21	47754634	47754634	PCNT	pericentrin	NM_006031	C	G	nonsynonymous SNV	C591G	I197M	M4	T	B	-0.47
22	26997976	26997976	CRYBB1	crystallin, beta B1	NM_001887	C	T	nonsynonymous SNV	G442A	E148K	M4	T	B	4.7
22	37690758	37690758	CYTH4	cytohesin 4	NM_013385	G	A	nonsynonymous SNV	G160A	E54K	M4	T	B	2.53
22	38308466	38308466	MICALL1	MICAL-like 1	NM_033386	C	T	stopgain	C304T	Q102X	M4	T		4.81
22	50757291	50757291	DENND6B	DENN/MADD domain contair	NM_001001794	G	A	nonsynonymous SNV	C244T	P82S	M4	D	D	4.86
22	50876662	50876662	PPP6R2	protein phosphatase 6, regula	NM_001242898.1	T	G	nonsynonymous SNV	T1899G	D633E	M4	T	B	-7.96
3	100103375	100103375	TOMM70A	translocase of outer mitochor	NM_014820	G	A	nonsynonymous SNV	C683T	A228V	M4	T	D	5.95
3	108074154	108074154	HHLA2	HERV-H LTR-associating 2	NM_007072.3	C	T	nonsynonymous SNV	C611T	S204L	M4		P	3.89
3	121712730	121712730	ILDR1	immunoglobulin-like domain c	NM_001199799.1	A	C	nonsynonymous SNV	T866G	L289W	M4	T	D	5.14
3	122003757	122003757	CASR	calcium-sensing receptor	NM_001178065.1	G	T	nonsynonymous SNV	G2986T	A996S	M4	T	B	4.92
3	130125116	130125116	COL6A5	collagen, type VI, alpha 5	NM_001278298.1	G	C	nonsynonymous SNV	G4430C	C1477S	M4	T	B	0.227

3	130134492	130134492	COL6A5	collagen, type VI, alpha 5	NM_001278298.1	T	C	nonsynonymous SNV	T4765C	S1589P	M4	T	B	-5.28
3	132209844	132209844	DNAJC13	DnaJ (Hsp40) homolog, subfa	NM_015268	T	C	nonsynonymous SNV	T3572C	I1191T	M4	T	B	3.98
3	134339627	134339627	KY	kyphoscoliosis peptidase	NM_178554	C	T	nonsynonymous SNV	G556A	V186I	M4	T	D	4.66
3	1424809	1424809	CNTN6	contactin 6	NM_014461.3	G	A	nonsynonymous SNV	G2350A	E784K	M4	T	B	4.28
3	151150566	151150566	MED12L	mediator complex subunit 12-	NM_053002	C	T	nonsynonymous SNV	C6412T	P2138S	M4	D	B	4.65
3	172365832	172365832	NCEH1	neutral cholesterol ester hydr	NM_001146276.1	T	G	nonsynonymous SNV	A307C	K103Q	M4	T	B	4.78
3	172607409	172607409	SPATA16	spermatogenesis associated	NM_031955	C	T	nonsynonymous SNV	G1661A	R554Q	M4	D	D	5.78
3	186445052	186445052	KNG1	kininogen 1	NM_001102416.2	T	G	nonsynonymous SNV	T591G	I197M	M4	D	P	-9.97
3	187089031	187089031	RTP4	receptor (chemosensory) tran	NM_022147	C	A	nonsynonymous SNV	C611A	A204D	M4	T	B	0.673
3	193080414	193080414	ATP13A5	ATPase type 13A5	NM_198505	C	G	nonsynonymous SNV	G397C	E133Q	M4	T	B	5.6
3	195513365	195513365	MUC4	mucin 4, cell surface associat	NM_018406	G	A	nonsynonymous SNV	C5086T	L3298H	M4		B	0
3	195516305	195516305	MUC4	mucin 4, cell surface associat	NM_018406	C	T	nonsynonymous SNV	G2146A	A716T	M4	T	B	-5.21
3	32995966	32995966	CCR4	chemokine (C-C motif) recept	NM_005508	C	T	nonsynonymous SNV	C1052T	T351I	M4	D	P	5.86
3	38357961	38357961	SLC22A14	solute carrier family 22, mem	NM_004803	C	G	nonsynonymous SNV	C1679G	P560R	M4	D	D	2.39
3	38595902	38595902	SCN5A	sodium channel, voltage gate	NM_198056.2	C	T	nonsynonymous SNV	G4681A	A1561T	M4	T	B	0.876
3	3887871	3887871	LRRN1	leucine rich repeat neuronal 1	NM_020873	G	A	nonsynonymous SNV	G1546A	V516I	M4	T	B	5.69
3	46729757	46729757	ALS2CL	ALS2 C-terminal like	NM_147129.3	C	G	nonsynonymous SNV	G133C	E45Q	M4	T	P	4.36
3	48669174	48669174	SLC26A6	solute carrier family 26 (anion	NM_022911.2	C	A	nonsynonymous SNV	G811T	A271S	M4	D	P	-10.7
3	52740182	52740182	SPCS1	signal peptidase complex sub	NM_014041	C	G	nonsynonymous SNV	C121G	P41A	M4	T	B	-4.39
3	52833805	52833805	ITIH3	Inter-alpha-trypsin inhibitor he	NM_002217	C	A	nonsynonymous SNV	C943A	Q315K	M4	T	B	4.15
3	57132275	57132275	IL17RD	Interleukin 17 receptor D	NM_017563	C	T	nonsynonymous SNV	G1456A	G486R	M4	T	P	5.54
3	69299233	69299233	FRMD4B	FERM domain containing 4B	NM_015123	C	G	nonsynonymous SNV	G519C	E173D	M4	T	B	4.33
3	71021812	71021812	FOXP1	Forkhead box P1	NM_032682	T	G	nonsynonymous SNV	A1546C	N516H	M4	D	D	5.81
3	75788382	75788382	ZNF717	zinc finger protein 717	NM_001290209	C	T	nonsynonymous SNV	G242A	G81E	M4	T	B	-3.99
3	75788394	75788394	ZNF717	zinc finger protein 717	NM_001290209	C	G	nonsynonymous SNV	G230C	R77T	M4	T	B	1.23
3	97664725	97664725	MINA	MYC induced nuclear antigen	NM_001042533	C	T	nonsynonymous SNV	G1156A	A386T	M4	T	B	1.6
3	97868588	97868588	OR5H14	Olfactory receptor, family 5, s	NM_001005514	A	G	nonsynonymous SNV	A359G	Y120C	M4	D	B	2.49
4	13615865	13615865	BOD1L1	Biorientation of chromosomes	NM_148894	C	T	nonsynonymous SNV	G1129A	E377K	M4	T	D	5.45
4	1388945	1388945	CRIPAK	Cysteine-rich PAK1 inhibitor	NM_175918	T	C	nonsynonymous SNV	T646C	W216R	M4	T	B	-2.82
4	152570668	152570668	FAM160A1	Family with sequence similari	NM_001109977	A	G	nonsynonymous SNV	A1475G	Y492C	M4	T	B	4
4	159782889	159782889	FNIP2	Folliculin interacting protein 2	NM_020840	G	T	nonsynonymous SNV	G1426T	A476S	M4		P	5.74
4	162306909	162306909	FSTL5	Follistatin-like 5	NM_020116	C	T	nonsynonymous SNV	G2534A	G845E	M4	D	D	5.84
4	183673041	183673041	TENM3	Teneurin transmembrane pro	NM_001080477	C	T	nonsynonymous SNV	C3718T	R1240C	M4	D	D	5.38
4	183694753	183694753	TENM3	Teneurin transmembrane pro	NM_001080477	T	G	nonsynonymous SNV	T5021G	I1674S	M4	T	P	5.26
4	186318414	186318414	ANKRD37	Ankyrin repeat domain 37	NM_181726	T	G	nonsynonymous SNV	T138G	F46L	M4	T	B	-4.25
4	20490500	20490500	SLIT2	slit homolog 2 (Drosophila)	NM_004787	C	T	nonsynonymous SNV	C670T	L224F	M4	D	D	3.72
4	3487359	3487359	DOK7	Docking protein 7	NM_173660	C	T	nonsynonymous SNV	C626T	P209L	M4	D	D	4.7
4	4304522	4304522	ZBTB49	Zinc finger and BTB domain c	NM_145291	A	C	nonsynonymous SNV	A959C	Y320S	M4	T	B	-2.97
4	71099827	71099827	FDCSP	Follicular dendritic cell secret	NM_152997	T	A	nonsynonymous SNV	T181A	F61I	M4	D	B	1.19
4	76489582	76489582	C4orf26	Chromosome 4 open reading	NM_001206981	G	A	nonsynonymous SNV	G370A	V124I	M4		B	-4.61
4	77003361	77003361	ART3	ADP-ribosyltransferase 3	NM_001130016	C	T	nonsynonymous SNV	C454T	P152S	M4		B	2.37
4	88106627	88106627	KLHL8	Kelch-like family member 8	NM_020803	G	A	stopgain	C541T	R181X	M4	T		4.82
4	88536818	88536818	DSPP	Dentin sialophosphoprotein	NM_014208	A	G	nonsynonymous SNV	A3004G	N1002D	M4	T		
5	107216812	107216812	FBXL17	F-box and leucine-rich repeat	NM_001163315	T	A	nonsynonymous SNV	A1891T	T631S	M4	T	D	5.95
5	126732427	126732427	MEGF10	Multiple EGF-like-domains 10	NM_032446	G	A	nonsynonymous SNV	G616A	V206I	M4	T	B	0.787
5	138857925	138857925	TMEM173	Transmembrane protein 173	NM_198282	C	G	nonsynonymous SNV	G689C	G230A	M4	T	B	0.964
5	140202256	140202256	PCDHA5	Protocadherin alpha 5	NM_018908	G	A	nonsynonymous SNV	G896A	G299D	M4	D	D	4.11
5	140216042	140216042	PCDHA7	Protocadherin alpha 7	NM_018910	G	A	nonsynonymous SNV	G2074A	E692K	M4	T	B	0.65
5	140237158	140237158	PCDHA10	Protocadherin alpha 10	NM_018901	G	A	nonsynonymous SNV	G1525A	V509M	M4	D	D	3.63
5	140559914	140559914	PCDHB8	Protocadherin beta 8	NM_019120	T	C	nonsynonymous SNV	T2299C	F767L	M4	D	B	4.53
5	140574042	140574042	PCDHB10	Protocadherin beta 10	NM_018930	G	T	nonsynonymous SNV	G1917T	R639S	M4	T	P	1.18
5	140682757	140682757	SLC25A2	Solute carrier family 25 (mito	NM_031947	C	T	nonsynonymous SNV	G676A	V226I	M4	T	B	1.89
5	140725828	140725828	PCDHGA3	Protocadherin gamma subfan	NM_018916	C	T	nonsynonymous SNV	C2228T	A743V	M4	T	B	4.14
5	140751128	140751128	PCDHGB3	Protocadherin gamma subfan	NM_018924	C	G	nonsynonymous SNV	C1167G	N389K	M4			
5	140772427	140772427	PCDHGA8	protocadherin gamma subfan	NM_014004	T	G	nonsynonymous SNV	T47G	L16R	M4	D	D	5.04
5	140793007	140793007	PCDHGA10	Protocadherin gamma subfan	NM_018913	A	G	nonsynonymous SNV	A265G	I89V	M4	T	B	2.76
5	157214920	157214920	CLINT1	clathrin interactor 1	NM_001195556	G	A	nonsynonymous SNV	C1612T	R538W	M4	D	D	5.97
5	168233583	168233583	SLIT3	slit homolog 3	NM_001271946	G	A	nonsynonymous SNV	C803T	S268L	M4	T	B	4.66
5	174868847	174868847	DRD1	Dopamine receptor D1	NM_000794	G	A	nonsynonymous SNV	C1256T	A419V	M4	T	B	4.54
5	176963612	176963612	FAM193B	family with sequence similarit	NM_001190946	G	A	nonsynonymous SNV	C823T	P275S	M4	T	B	3.44
5	179044053	179044053	HNRNPH1	Heterogeneous nuclear ribon	NM_001257293	G	T	stopgain	C1116A	Y372X	M4	T		5.77
5	19747219	19747219	CDH18	cadherin 18, type 2	NM_001291956	C	G	nonsynonymous SNV	G355C	E119Q	M4		D	5.23
5	21975364	21975364	CDH12	Cadherin 12, type 2	NM_004061	T	G	nonsynonymous SNV	A362C	E121A	M4	D	D	5.16
5	41149420	41149420	C6	Complement component 6	NM_001115131	C	T	nonsynonymous SNV	G2546A	R849K	M4	D	D	5.85
5	72419267	72419267	TMEM171	Transmembrane protein 171	NM_173490	T	C	nonsynonymous SNV	T67C	F23L	M4	d	b	5.09
5	74518111	74518111	ANKRD31	Ankyrin repeat domain 31	NM_001164443	T	C	nonsynonymous SNV	A251G	K84R	M4	T	B	-0.013
5	78915496	78915496	PAPD4	PAP associated domain conta	NM_001114394	C	G	nonsynonymous SNV	C25G	R9G	M4	D	D	4.55

5	79028472	79028472	CMYA5	Cardiomyopathy associated 5	NM_153610	C	T	nonsynonymous SNV	C3884T	A1295V	M4	T	B	-0.023
5	79095383	79095383	CMYA5	Cardiomyopathy associated 5	NM_153610	G	A	nonsynonymous SNV	G12154A	G4052R	M4	D	D	5.99
5	82833369	82833369	VCAN	Versican	NM_004385	A	G	nonsynonymous SNV	A4547G	K1516R	M4	T	B	2.79
5	82835724	82835724	VCAN	Versican	NM_004385	T	A	nonsynonymous SNV	T6902A	F2301Y	M4	T	B	3.61
5	82837631	82837631	VCAN	Versican	NM_004385	G	T	nonsynonymous SNV	G8809T	D2937Y	M4	D	P	0.819
5	96086334	96086334	CAST	Calpastatin	NM_001042440	G	C	nonsynonymous SNV	G1349C	C450S	M4	T	B	4.46
6	132945390	132945390	TAAR2	Trace amine associated rece	NM_001033080	C	T	nonsynonymous SNV	G25A	E9K	M4	T	B	-1.59
6	133015271	133015271	VNN1	Vanin 1	NM_004666	T	C	nonsynonymous SNV	A392G	N131S	M4	T	D	6.07
6	158910698	158910698	TULP4	Tubby like protein 4	NM_020245	G	A	nonsynonymous SNV	G1565A	S522N	M4	T	B	5.57
6	160952838	160952838	LPA	Lipoprotein, Lp(a)	NM_005577	G	A	nonsynonymous SNV	C6046T	R2016C	M4	T	D	3.02
6	160977181	160977181	LPA	Lipoprotein, Lp(a)	NM_005577	A	G	nonsynonymous SNV	T4849C	Y1617H	M4	D	D	2.77
6	16145325	16145325	MYLIP	Myosin regulatory light chain	NM_013262	A	G	nonsynonymous SNV	A1025G	N342S	M4	T	B	3.85
6	16290761	16290761	GMPR	Guanosine monophosphate r	NM_006877	T	A	nonsynonymous SNV	T766A	F256I	M4	T	B	5.22
6	166575973	166575973	T	T, brachyury homolog (mouse	NM_003181	G	A	nonsynonymous SNV	C866T	P289L	M4			
6	167592535	167592535	TCP10L2	T-complex 10-like 2	NM_001145121	G	C	nonsynonymous SNV	G694C	G232R	M4	T	B	0.56
6	170059575	170059575	WDR27	WD repeat domain 27	NM_182552	G	A	nonsynonymous SNV	C1169T	A390V	M4	D	D	4.7
6	170070723	170070723	WDR27	WD repeat domain 27	NM_182552	A	G	nonsynonymous SNV	T398C	L133P	M4	T	B	0.661
6	170176146	170176146	ERMARD	ER membrane-associated RN	NM_018341	G	A	nonsynonymous SNV	G1505A	R502H	M4	T	B	-10.5
6	29080152	29080152	OR2J3	Olfactory receptor, family 2, s	NM_001005216	A	C	nonsynonymous SNV	A485C	H162P	M4	D	P	1.52
6	29759923	29759923	LOC554223		NM_001207043	T	C	nonsynonymous SNV	T139C	S47P	M4			
6	30673163	30673163	MDC1	Mediator of DNA-damage che	NM_014641	T	G	nonsynonymous SNV	A3797C	Y1266S	M4	T	B	1.26
6	31378956	31378956	MICA	MHC class I polypeptide-relat	NM_001289152	C	G	nonsynonymous SNV	C142G	L48V	M4	D	D	1.94
6	31540784	31540784	LTA	lymphotoxin alpha	NM_001159740	C	A	nonsynonymous SNV	C179A	T60N	M4	T	B	3.15
6	31605278	31605278	PRRC2A	Proline-rich coiled-coil 2A	NM_004638	C	T	nonsynonymous SNV	C6389T	P2130L	M4	D	P	5.84
6	31938412	31938412	DXO	Decapping Exoribonuclease	NM_005510	G	T	nonsynonymous SNV	C783A	H261Q	M4	T	B	3.59
6	34824636	34824636	UHRF1BP1	UHRF1 Binding Protein 1	NM_017754	A	G	nonsynonymous SNV	A1361G	Q454R	M4	T	B	5.01
6	4133855	4133855	ECI2	Enoyl-CoA delta isomerase 2	NM_206836	C	T	nonsynonymous SNV	G141A	M47I	M4	T	B	4.4
6	42074872	42074872	C6orf132	Chromosome 6 Open Readin	NM_001164446	A	C	nonsynonymous SNV	T778G	S260A	M4	T		3.62
6	42712957	42712957	TBCC	Tubulin Folding Cofactor C	NM_003192	A	C	nonsynonymous SNV	T855G	S285R	M4	D	B	3.26
6	42891022	42891022	PTCRA	pre T-cell antigen receptor al	NM_001243168	G	A	nonsynonymous SNV	G316A	V106I	M4	T	P	4.82
6	46555780	46555780	CYP39A1	cytochrome P450, family 39,	NM_001278739:	C	A	nonsynonymous SNV	G636T	L212F	M4	D	P	-1.57
6	46555781	46555781	CYP39A1	cytochrome P450, family 39,	NM_001278739:	A	T	stopgain	T635A	L212X	M4			
6	49753961	49753961	PGK2	Phosphoglycerate Kinase 2	NM_138733	A	C	nonsynonymous SNV	T940G	L314V	M4	D	D	3.03
6	49928150	49928150	DEFB114	Defensin, Beta 114	NM_001037499	G	A	nonsynonymous SNV	C65T	T22I	M4		B	0.415
6	56420538	56420538	DST	Dystonin	NM_001144769	C	T	nonsynonymous SNV	G8384A	R2795H	M4	T	B	5.67
6	65300143	65300143	EYS	Eyes shut homolog (Drosoph	NM_001292009	G	C	nonsynonymous SNV	C5617G	L1873V	M4	T	B	1.96
6	76023011	76023011	FILIP1	filamin A interacting protein 1	NM_001289987	G	A	nonsynonymous SNV	C2546T	P849L	M4	D	D	4.77
6	99374696	99374696	FBXL4	F-box and leucine-rich repeat	NM_001278716	C	T	nonsynonymous SNV	G169A	E57K	M4	D	B	4.65
7	100014711	100014711	ZCWPW1	zinc finger, CW type with PW	NM_017984	T	C	nonsynonymous SNV	A457G	T153A	M4		B	0.203
7	100683249	100683249	MUC17	Mucin 17, Cell Surface Assoc	NM_001040105	C	T	nonsynonymous SNV	C8552T	P2851L	M4	T	P	0.267
7	117199582	117199582	CFTR	Cystic Fibrosis Transmembra	NM_000492	G	A	nonsynonymous SNV	G1457A	G486E	M4	D	D	5.48
7	12376811	12376811	VWDE	Von Willebrand Factor D And	NM_001135924	A	C	nonsynonymous SNV	T4454G	F1485C	M4	T	B	4.86
7	12610594	12610594	SCIN	Scinderin	NM_001112706	A	G	nonsynonymous SNV	A182G	H61R	M4	T	B	4.9
7	134346341	134346341	BPGM	2,3-bisphosphoglycerate mut	NM_199186	C	T	stopgain	C82T	Q28X	M4	D	D	6.04
7	135291649	135291649	NUP205	Nucleoporin 205kDa	NM_01513	A	G	nonsynonymous SNV	A3056G	N1019S	M4	T	B	5.18
7	139026152	139026152	C7orf55,C7orf55	chromosome 7 open reading	C7orf55-LUC7L2:NM_0	T	G	nonsynonymous SNV	C7orf55-LUC7L2:T22G	C7orf55-LUC7L2:S8A	M4	T	B	2.58
7	141414151	141414151	WEE2	WEE1 Homolog 2 (S. Pombe	NM_001105558	C	T	nonsynonymous SNV	C485T	S162F	M4	D	P	4.92
7	142655008	142655008	KEL	Kell Blood Group, Metallo-En	NM_000420	G	A	nonsynonymous SNV	C578T	T193M	M4	D	B	3.96
7	142655503	142655503	KEL	Kell Blood Group, Metallo-En	NM_000420	G	A	nonsynonymous SNV	C413T	S138F	M4	T	B	3.78
7	143085968	143085968	ZYX	Zyxin	NM_001010972	C	T	nonsynonymous SNV	C1423T	R475C	M4	T	B	2.66
7	143747879	143747879	OR2A5	Olfactory Receptor, Family 2,	NM_012365	C	T	nonsynonymous SNV	C385T	P129S	M4	D	D	4.36
7	1482025	1482025	MICALL2	MICAL-Like 2	NM_182924	G	A	nonsynonymous SNV	C1514T	P505L	M4	T	B	2.5
7	150761314	150761314	SLC4A2	Solute carrier family 4 (anion	NM_001199692	G	A	nonsynonymous SNV	G77A	G26E	M4	T	B	-0.421
7	151962265	151962265	KMT2C	Lysine (K)-Specific Methyltrar	NM_170606	C	T	nonsynonymous SNV	G1042A	D348N	M4	T	D	4.65
7	2418877	2418877	EIF3B	eukaryotic translation initiatio	NM_001037283	G	A	nonsynonymous SNV	G2341A	G781R	M4	D	P	5.82
7	30795331	30795331	INMT	indolethylamine N-methyltran	NM_006774	A	G	nonsynonymous SNV	A656G	E219G	M4	T	P	3.67
7	36552907	36552907	AOAH	acyloxyacyl hydrolase (neutr	NM_001637	G	A	nonsynonymous SNV	C1679T	P560L	M4	D	D	4.67
7	44715648	44715648	OGDH	oxoglutarate (alpha-ketogluta	NM_002541	C	T	nonsynonymous SNV	C1106T	S369F	M4	D	D	5.14
7	44805882	44805882	ZMIZ2	zinc finger, MIZ-type containi	NM_031449	C	T	nonsynonymous SNV	C2362T	P788S	M4	T	P	4.83
7	53103959	53103959	POM121L12	POM121 transmembrane nuc	NM_182595	G	A	nonsynonymous SNV	G595A	E199K	M4	D	B	0.346
7	57528813	57528813	ZNF716	Zinc finger protein 716	NM_001159279	G	A	nonsynonymous SNV	G646A	E216K	M4	T	B	-0.39
7	64439678	64439678	ZNF117	Zinc finger protein 117	NM_015852	G	A	nonsynonymous SNV	C271T	H91Y	M4	T	B	-2.97
7	6456474	6456474	DAGLB	diacylglycerol lipase, beta	NM_139179	G	A	nonsynonymous SNV	C1297T	P433S	M4	T	P	4.85
7	82455968	82455968	PCLO	Piccolo presynaptic cytomatri	NM_033026	C	G	nonsynonymous SNV	G14352C	K4784N	M4	D	D	4.6
7	883199	883199	SUN1	Sad1 and UNC84 domain co	NM_001171945	C	T	nonsynonymous SNV	C763T	P255S	M4		B	0.169
7	92028039	92028039	ANKIB1	Ankyrin repeat and IBR doma	NM_019004	C	A	nonsynonymous SNV	C3046A	L1016M	M4	D	B	3.36
7	99817585	99817585	PVRIG	Poliovirus receptor related im	NM_024070	G	T	stopgain	G52T	E18X	M4	D		-3.09

7	99917237	99917237	SPDYE3	Speedy/RINGO cell cycle reg	NM_001004351	A	T	nonsynonymous SNV	A1396T	I466F	M4	D		-3.09
8	11994957	11994957	USP17L2	Ubiquitin specific peptidase 1	NM_201402	T	C	nonsynonymous SNV	A1313G	K438R	M4	T	B	-0.523
8	11995785	11995785	USP17L2	Ubiquitin specific peptidase 1	NM_201402	T	A	nonsynonymous SNV	A485T	H162L	M4	D	D	0.745
8	12042924	12042924	FAM86B1	Family with sequence similar	NM_001083537	G	T	nonsynonymous SNV	C751A	R251S	M4	D	D	0.249
8	120940812	120940812	DEPTOR	DEP domain containing MTO	NM_022783	C	T	nonsynonymous SNV	C295T	H99Y	M4	D	D	5.95
8	13357175	13357175	DLC1	DLC1 Rho GTPase activating	NM_182643	A	G	nonsynonymous SNV	T406C	S136P	M4	T	B	-3.63
8	143413136	143413136	TSNARE1	t-SNARE domain containing	NM_145003	C	T	nonsynonymous SNV	G802A	V268I	M4	T	B	2.41
8	144945792	144945792	EPPK1	Epiplakin 1	NM_031308	T	C	nonsynonymous SNV	A1630G	K544E	M4	T	B	0.205
8	144946818	144946818	EPPK1	Epiplakin 1	NM_031308	C	T	nonsynonymous SNV	G604A	.D202N	M4	T	P	4.14
8	26221299	26221299	PPP2R2A	protein phosphatase 2, regula	NM_001177591	T	A	nonsynonymous SNV	T895A	S299T	M4	D	P	6.03
8	40438757	40438757	ZMAT4	Zinc finger, matrin-type 4	NM_024645	T	C	nonsynonymous SNV	A601G	T201A	M4	T	B	4.49
8	7215694	7215694	ZNF705G	Zinc finger protein 705G	NM_001164457	A	G	nonsynonymous SNV	T707C	V236A	M4	T		0.049
8	7217837	7217837	ZNF705G	Zinc finger protein 705G	NM_001164457	A	C	nonsynonymous SNV	T157G	S53A	M4	T		-0.55
8	73850273	73850273	KCNB2	Potassium channel, voltage c	NM_004770	C	T	nonsynonymous SNV	C2683T	H895Y	M4	D	B	5.34
9	102590773	102590773	NR4A3	nuclear receptor subfamily 4,	NM_173200	C	G	nonsynonymous SNV	C482G	P161R	M4	T	B	2.27
9	104335490	104335490	GRIN3A	Glutamate receptor, ionotropi	NM_133445	T	C	nonsynonymous SNV	A3314G	E1105G	M4	D	B	5.55
9	113212454	113212454	SVEP1	Sushi, von Willebrand factor t	NM_153366	A	T	nonsynonymous SNV	T3988A	L1330M	M4	T	B	0.456
9	116359106	116359106	RGS3	regulator of G-protein signalin	NM_144488	G	T	nonsynonymous SNV	G3470T	R1157L	M4	T	D	3.53
9	116856481	116856481	KIF12	Kinesin family member 12	NM_138424	C	A	nonsynonymous SNV	G879T	M293I	M4	T	B	5.56
9	116931445	116931445	COL27A1	Collagen, type XXVII, alpha 1	NM_032888	T	C	nonsynonymous SNV	T1610C	I537T	M4	T	B	4.51
9	117124731	117124731	AKNA	AT-Hook Transcription Factor	NM_030767	G	A	nonsynonymous SNV	C1871T	P624L	M4	T	B	-0.371
9	125315557	125315557	OR1N2	Olfactory Receptor, Family 1,	NM_001004457	T	C	nonsynonymous SNV	T109C	W37R	M4	T	B	-2.62
9	125316350	125316350	OR1N2	Olfactory Receptor, Family 1,	NM_001004457	C	T	nonsynonymous SNV	C902T	T301M	M4	T	B	4.56
9	125330125	125330125	OR1L8	Olfactory Receptor, Family 1,	NM_001004454	C	G	nonsynonymous SNV	G632C	R211P	M4	T	B	3.6
9	125391369	125391369	OR1B1	Olfactory Receptor, Family 1,	NM_001004450	A	G	nonsynonymous SNV	T446C	L149S	M4	T	B	1.96
9	129851270	129851270	ANGPTL2	Angiopoietin-Like 2	NM_012098	G	A	nonsynonymous SNV	C1430T	S477L	M4	D	D	4.92
9	133491777	133491777	FUBP3	Far Upstream Element (FUSE	NM_003934	G	A	nonsynonymous SNV	G440A	R147H	M4	D	D	5.21
9	133962930	133962930	LAMC3	Laminin, Gamma 3	NM_006059	C	T	nonsynonymous SNV	C4298T	T1433M	M4	T	B	2.92
9	134334588	134334588	PRRC2B	Proline-Rich Coiled-Coil 2B	NM_013318	A	G	nonsynonymous SNV	A1249G	M417V	M4	T	B	-11.2
9	139378914	139378914	C9orf163	Chromosome 9 Open Readin	NM_152571	T	C	nonsynonymous SNV	T14C	.L5P	M4	D	D	3
9	140100317	140100317	NDOR1	NADPH dependent diflavin o	NM_001144026	C	G	nonsynonymous SNV	C88G	R30G	M4	T	B	3.93
9	140147273	140147273	C9orf173	chromosome 9 open reading	NM_001256699	C	T	nonsynonymous SNV	C652T	L218F	M4	T	P	1.63
9	33264270	33264270	BAG1	BCL2-associated athanogene	NM_004323	T	C	nonsynonymous SNV	A403G	T135A	M4	T	B	-1.04
9	34622389	34622389	ARID3C	AT Rich Interactive Domain 3	NM_001017363	A	C	nonsynonymous SNV	T1003G	C335G	M4	T	B	0.293
9	34724241	34724241	FAM205A	Family With Sequence Simila	NM_001141917	C	A	nonsynonymous SNV	G2996T	S999I	M4	T	B	1.05
9	34724796	34724796	FAM205A	Family With Sequence Simila	NM_001141917	T	A	nonsynonymous SNV	A2441T	E814V	M4	T	B	2.24
9	90500405	90500405	SPATA31E1	SPATA31 subfamily E, memb	NM_178828	A	C	nonsynonymous SNV	A1003C	T335P	M4	T	B	0.534
9	96097662	96097662	C9orf129	Chromosome 9 open reading	NM_001098808	G	A	nonsynonymous SNV	C359T	A120V	M4	D	B	2.56
X	135427333	135427333	GPR112	Adhesion G protein-coupled r	NM_153834	G	A	nonsynonymous SNV	G1468A	A490T	M4	T	B	-1.22
X	151532964	151532964	GABRA3	Gamma-aminobutyric acid (G	NM_000808	T	G	nonsynonymous SNV	A79C	T27P	M4	D	B	0.116
X	151869731	151869731	MAGEA6	Melanoma antigen family A6	NM_175868	G	A	nonsynonymous SNV	G421A	G141R	M4	T	B	-1.21
X	152159263	152159263	PNMA5	Paraneoplastic Ma antigen fa	NM_001103150	C	T	nonsynonymous SNV	G880A	A294T	M4	T	B	-2.14
X	152482824	152482824	MAGEA1	Melanoma antigen family A1	NM_004988	C	T	nonsynonymous SNV	G187A	A63T	M4	T	B	-1.87
X	34962641	34962641	FAM47B	Family with sequence similar	NM_152631	G	A	nonsynonymous SNV	G1693A	E565K	M4	D	P	0.843
X	47307746	47307746	ZNF41	Zinc finger protein 41	NM_007130	A	G	nonsynonymous SNV	T1423C	S475P	M4	D	P	-0.452
X	6995419	6995419	HDHD1	Haloacid dehalogenase-like h	NM_012080	C	T	nonsynonymous SNV	G352A	A118T	M4	T	B	-0.796
X	7811336	7811336	VCX	Variable charge, X-linked	NM_013452	C	A	nonsynonymous SNV	C92A	P31Q	M4	D	D	0.167
Y	6738049	6738049	AMELY	Amelogenin, Y-linked	NM_001143	C	T	nonsynonymous SNV	G100A	E34K	M4		D	1.57
7	140453136	140453136	BRAF	v-raf murine sarcoma viral or	NM_004333	A	T	nonsynonymous SNV	T1799A	V600E	M4	D	D	5.65
19	49558211	49558211	CGB7	Chorionic Gonadotropin, Beta	NM_033142	T	G	nonsynonymous SNV	A70C	M24L	M4			946
7	100645960	100645960	MUC12	Mucin12	NM_001164462	A	T	nonsynonymous SNV	A12116T	D4039V	M4	T		-1.72
12	109017374	109017374	SELPLG	Selectin P Ligand	NM_001206609	T	G	nonsynonymous SNV	A758C	E253A	M4	T	B	-5.23
7	57529086	57529086	ZNF716	Zinc Finger Protein 716	NM_001159279	C	A	nonsynonymous SNV	C919A	R307S	M4	T	B	-218
13	46170839	46170839	ERICH6B	Glutamate-Rich 6B	NM_182542	G	T	nonsynonymous SNV	C302A	A101E	M4	D	B	-6.51
3	195452870	195452870	MUC20	Mucin20	NM_001282506	G	A	nonsynonymous SNV	G1396A	E466K	M4	T	B	2.47
2	27324371	27324371	CGREF1	Cell Growth Regulator With E	NM_006569	C	T	nonsynonymous SNV	G728A	G243E	M4	T	B	1.69
1	94001896	94001896	FNBP1L	Formin Binding Protein 1-Like	NM_001164473	C	G	nonsynonymous SNV	C998G	S333C	M4	D	B	5.16
15	22743461	22743461	GOLGA6L1	golgin A6 family-like 1	NM_001001413	T	A	nonsynonymous SNV	T1846A	W616R	M4	T		
15	22743461	22743461	GOLGA6L22	golgin A6 family-like 22	NM_001271664	T	A	nonsynonymous SNV	T1810A	W604R	M4	T		
3	195452872	195452872	MUC20	Mucin20	NM_001282506	A	C	nonsynonymous SNV	A1398C	E466D	M4	T	B	-3.24
3	195452873	195452873	MUC20	Mucin20	NM_001282506	G	A	nonsynonymous SNV	G1399A	A467T	M4	T	P	2.42
8	2092844	2092844	MYOM2	myomesin 2	NM_003970	C	G	nonsynonymous SNV	C4337G	A1446G	M4	T	B	-1.53
5	43277335	43277335	NIM1K	NM1 Serine/treonin Kinase	NM_153361	G	C	nonsynonymous SNV	G469C	E157Q	M4	D	D	5.91
1	248801610	248801610	OR2T35	Olfattory Receptor Family 2, S	NM_001001827	G	A	nonsynonymous SNV	C950T	A317V	M4	T	B	-3.53
19	2917807	2917807	ZNF57	Zinc Finger Protein 57	NM_173480	A	C	nonsynonymous SNV	A1188C	Q396H	M4	T	B	-4.5
19	20229099	20229099	ZNF90	Zinc Finger Protein 90	NM_007138	G	T	nonsynonymous SNV	G736T	A246S	M4		B	-2.22
19	20045163	20045163	ZNF93	Zinc Finger Protein 93	NM_031218	T	A	nonsynonymous SNV	T1399A	S467T	M4	T	B	-1.7

19	53269382	53269382	ZNF600	Zinc Finger Protein 600	NM_198457	A	T	nonsynonymous SNV	T1627A	C543S	M4	T	D	-3.02
1	153665659	153665659	NPR1	natriuretic peptide receptor 1	NM_000906	G	A	nonsynonymous SNV	G3109A	D1037N	M6	T	B	4.42
11	3740679	3740679	NUP98	nucleoporin 98kDa	NM_016320.4	C	T	nonsynonymous SNV	G2362A	D788N	M6	T	D	5.37
12	104376700	104376700	TDG	thymine DNA glycosylase	NM_003211	A	C	nonsynonymous SNV	A602C	K201T	M6	T	P	5.4
12	8757515	8757515	AICDA	activation-induced cytidine de	NM_020661	T	A	nonsynonymous SNV	A431T	Y144F	M6	T	B	5.44
13	103385259	103385259	CCDC168	coiled-coil domain containing	NM_001146197	G	A	stopgain	C17788T	Q5930X	M6	T		2.01
15	58001174	58001174	GCOM1,POLR2	GRINL1A complex locus 1	NM_001285900	A	C	nonsynonymous SNV	A1567C	T523P	M6	D	B	-0.124
16	16177275	16177275	ABCC1	ATP-binding cassette, sub-fa	NM_004996	G	A	nonsynonymous SNV	G2168A	R723Q	M6	T	B	-2.86
16	50707676	50707676	SNX20	sorting nexin 20	NM_182854	C	A	nonsynonymous SNV	G592T	A198S	M6	T	P	5.63
16	66426186	66426186	CDH5	cadherin 5, type 2 (vascular e	NM_001795	C	T	stopgain	C1117T	Q373X	M6	T		2.53
19	14261908	14261908	LPHN1	adhesion G protein-coupled r	NM_001008701	C	G	nonsynonymous SNV	G4202C	S1401T	M6	T	D	3.93
19	18377653	18377653	KIAA1683	KIAA1683	NM_001145304.1	G	A	nonsynonymous SNV	C697T	R76Q	M6	D	B	0.004
2	208988958	208988958	CRYGD	crystallin, gamma D	NM_006891	T	C	nonsynonymous SNV	A130G	M44V	M6	T	B	4.35
2	219603798	219603798	TTLL4	tubulin tyrosine ligase-like far	NM_014640	A	C	nonsynonymous SNV	A1399C	T467P	M6	D	B	3.49
2	236403349	236403349	AGAP1	ArfGAP with GTPase domain	NM_001037131.2	C	A	nonsynonymous SNV	C19A	L7M	M6	D	B	2.87
20	47297823	47297823	PREX1	phosphatidylinositol-3,4,5-tris	NM_020820	T	G	nonsynonymous SNV	A1385C	E462A	M6	D	B	4.41
20	5904705	5904705	CHGB	chromogranin B	NM_001819	G	A	nonsynonymous SNV	G1915A	E639K	M6	D	P	5.79
22	21401723	21401723	LRRC74B	leucine rich repeat containing	NM_001291006	C	T	nonsynonymous SNV	C218T	S73F	M6	D		1.39
3	157188277	157188277	VEPH1	ventricular zone expressed P	NM_024621.2	G	C	nonsynonymous SNV	C180G	I60M	M6	D	P	2.85
3	195506741	195506741	MUC4	mucin 4, cell surface associat	NM_018406	G	A	nonsynonymous SNV	C11710T	P3904S	M6		B	0
5	73931128	73931128	ENC1	Ectodermal-neural cortex 1	NM_001256574	C	T	nonsynonymous SNV	G1183A	G395R	M6	D	D	5.89
6	74498287	74498287	CD109	CD109 molecule	NM_133493	C	T	nonsynonymous SNV	C2653T	P885S	M6	D	D	5.22
9	134183339	134183339	PPAPDC3	Phosphatidic Acid Phosphata	NM_032728	G	A	nonsynonymous SNV	G481A	G161S	M6	D	D	3.78
9	134183340	134183340	PPAPDC3	Phosphatidic Acid Phosphata	NM_032728	G	A	nonsynonymous SNV	G482A	G161D	M6	D	D	4.68
9	2039808	2039808	SMARCA2	SWI/SNF related, matrix asso	NM_003070	A	C	nonsynonymous SNV	A698C	Q233P	M6	D	D	5.89
9	35869919	35869919	OR13J1	Olfactory Receptor, Family 13	NM_001004487	C	T	nonsynonymous SNV	G480A	M160I	M6	D	B	-9.36
9	732392	732392	KANK1	KN motif and ankyrin repeat d	NM_001256877	C	G	stopgain	C3020G	S1007X	M6			5.64
1	152732951	152732951	KPRP	keratinocyte proline-rich prote	NM_001025231	C	G	nonsynonymous SNV	C887G	T296S	M8	D	D	4.89
1	21806621	21806621	NBPF3	neuroblastoma breakpoint far	NM_032264.4	T	G	nonsynonymous SNV	T1286G	F429C	M8	D	P	-0.545
1	28800114	28800114	PHACTR4	phosphatase and actin regula	NM_001048183.1	C	G	nonsynonymous SNV	C872G	S291C	M8	T	D	5.38
1	43649485	43649485	CFAP57	cilia and flagella associated p	NM_001195831.2	G	T	nonsynonymous SNV	G698T	S233I	M8	T	B	-3.64
1	52991465	52991465	ZCCHC11	zinc finger, CCHC domain co	NM_001009881.2	C	A	nonsynonymous SNV	G488T	G163V	M8	T	B	0.678
1	55273580	55273580	C1orf177	chromosome 1 open reading	NM_001110533.1	G	T	nonsynonymous SNV	G376T	G126C	M8	D	D	4.45
1	89652072	89652072	GBP4	guanylate binding protein 4	NM_052941	C	T	nonsynonymous SNV	G1651A	E551K	M8	T	B	-4.45
1	89652102	89652102	GBP4	guanylate binding protein 4	NM_052941	A	T	nonsynonymous SNV	T1621A	Y541N	M8	T	B	0.417
10	101563815	101563815	ABCC2	ATP-binding cassette, sub-fa	NM_000392	G	A	nonsynonymous SNV	G1249A	V417I	M8	T	B	-10.3
10	27688076	27688076	PTCHD3	patched domain containing 3	NM_001034842	G	T	stopgain	C1451A	S484X	M8	T		-0.472
11	111631593	111631593	PPP2R1B	protein phosphatase 2, regula	NM_002716.4	G	T	nonsynonymous SNV	C489A	F163L	M8	D	B	5.05
11	129780436	129780436	PRDM10	PR domain containing 10	NM_020228	T	G	nonsynonymous SNV	A3110C	Q1037P	M8	T	B	1.21
11	1643049	1643049	KRTAP5-4	keratin associated protein 5-4	NM_001012709	A	C	nonsynonymous SNV	T275G	V92G	M8	T	B	1.02
12	123200158	123200158	HCAR3	hydroxycarboxylic acid recep	NM_006018	G	C	nonsynonymous SNV	C1127G	A376G	M8	T	B	0.929
12	64810521	64810521	XPOT	exportin, tRNA	NM_007235	C	T	stopgain	C187T	Q63X	M8			5.2
13	21955683	21955683	ZDHHC20	zinc finger, DHHC-type conta	NM_153251.3	C	A	nonsynonymous SNV	G947T	G316V	M8	T	B	4.79
14	53525238	53525238	DDHD1	DDHD domain containing 1	NM_030637.2	A	T	nonsynonymous SNV	T1949A	I650N	M8	T	D	6.14
15	31332525	31332525	TRPM1	transient receptor potential ca	NM_001252020.1	C	A	nonsynonymous SNV	G2163T	E721D	M8	D	B	4.06
15	42744062	42744062	ZNF106	zinc finger protein 106	NM_022473	G	C	nonsynonymous SNV	C339G	D113E	M8	T	B	3.58
15	54305755	54305755	UNC13C	unc-13 homolog C (C. elegan	NM_001080534	C	G	nonsynonymous SNV	C655G	R219G	M8	D	D	4.97
15	89386652	89386652	ACAN	aggrecan	NM_013227.3	G	A	nonsynonymous SNV	G824A	R275Q	M8	T	D	5.56
16	15179544	15179544	RRN3	RRN3 homolog, RNA polyme	NM_018427.4	T	C	nonsynonymous SNV	A484G	I162V	M8	T	B	3.15
16	3586230	3586230	CLUAP1	clusterin associated protein 1	NM_015041.2	C	T	nonsynonymous SNV	C1201T	R401W	M8	D	P	2.59
17	1649055	1649055	SERPINF2	serpin peptidase inhibitor, cla	NM_000934.3	C	G	nonsynonymous SNV	C219G	D73E	M8	T	B	-1.8
17	45438788	45438788	EFCAB13	EF-hand calcium binding dom	NM_152347	C	T	stopgain	C706T	R236X	M8	T		1.68
17	4802111	4802111	CHRNE	cholinergic receptor, nicotinic	NM_000080	C	G	nonsynonymous SNV	G1402C	V468L	M8	T	B	0.885
18	6950809	6950809	LAMA1	laminin, alpha 1	NM_005559	G	T	nonsynonymous SNV	C8369A	A2790E	M8	T	P	5.5
19	8616735	8616735	MYO1F	myosin IF	NM_012335	C	A	nonsynonymous SNV	G660T	E220D	M8	T	B	3.79
19	9058907	9058907	MUC16	mucin 16, cell surface associa	NM_024690	G	C	nonsynonymous SNV	C28539G	H9513Q	M8	D	B	-4.44
19	9075346	9075346	MUC16	mucin 16, cell surface associa	NM_024690	T	A	nonsynonymous SNV	A12100T	I4034F	M8	D	P	-1.05
2	114257354	114257354	FOXD4L1	forkhead box D4-like 1	NM_012184	A	G	nonsynonymous SNV	A521G	H174R	M8	T	B	2.57
2	170033094	170033094	LRP2	low density lipoprotein recept	NM_004525	G	T	nonsynonymous SNV	C10398A	S3466R	M8	T	B	1.36
2	196825402	196825402	DNAH7	dynein, axonemal, heavy cha	NM_018897	T	C	nonsynonymous SNV	A2473G	K825E	M8	T	B	5.74
2	207041786	207041786	GPR1	G protein-coupled receptor 1	NM_005279.3	G	T	nonsynonymous SNV	C186A	F62L	M8	T	B	4.97
20	61444540	61444540	OGFR	opioid growth factor receptor	NM_007346	A	C	nonsynonymous SNV	A1573C	S525R	M8	T	P	-1.64
22	29735038	29735038	AP1B1	adaptor-related protein comp	NM_001127.3	G	A	nonsynonymous SNV	C2104T	L702F	M8	T	D	5.84
22	42422822	42422822	WBP2NL	WBP2 N-terminal like	NM_152613	C	A	stopgain	C567A	Y189X	M8	T		-4.17
22	42523514	42523514	CYP2D6	cytochrome P450, family 2, s	NM_000106.5	C	T	nonsynonymous SNV	G1108A	V370I	M8	T	B	-6.63
3	65415704	65415704	MAGI1	Membrane associated guany	NM_001033057	C	A	nonsynonymous SNV	G1658T	C553F	M8	D	D	5.92
5	140222641	140222641	PCDHA8	Protocadherin alpha 8	NM_018911	A	G	nonsynonymous SNV	A1735G	K579E	M8	T	B	0.381

5	179069883	179069883	C5orf60	Chromosome 5 open reading	NM_001142306	T	A	nonsynonymous SNV	A670T	T224S	M8	T	B	-0.839
5	892761	892761	BRD9	Bromodomain containing 9	NM_023924	C	A	nonsynonymous SNV	G12T	K4N	M8	D	B	2.62
6	168445537	168445537	KIF25	kinesin family member 25	NM_030615	G	T	nonsynonymous SNV	G1016T	C339F	M8	D	P	4.12
6	86259444	86259444	SNX14	Sorting nexin 14	NM_153816	C	A	nonsynonymous SNV	G788T	C263F	M8	D	D	5.17
7	44047061	44047061	SPDYE1	Speedy/RINGO cell cycle reg	NM_175064	G	T	nonsynonymous SNV	G827T	R276L	M8	T	P	
7	63727127	63727127	ZNF679	Zinc finger protein 679	NM_153363	G	T	nonsynonymous SNV	G1116T	R372S	M8	T	D	-0.427
8	71071744	71071744	NCOA2	Nuclear receptor coactivator :	NM_006540	G	T	nonsynonymous SNV	C1120A	H374N	M8	T	B	6.17
9	135946015	135946015	CEL	Carboxyl Ester Lipase	NM_001807	T	C	nonsynonymous SNV	T1463C	I488T	M8	D	D	4.57
9	35957669	35957669	OR2S2	Olfactory Receptor, Family 2,	NM_019897	T	C	nonsynonymous SNV	A427G	M143V	M8	T	B	2.09
X	106396402	106396402	NUP62CL	Nucleoporin 62kDa C-termina	NM_017681	A	G	nonsynonymous SNV	T530C	I177T	M8		B	-4.01
X	114426385	114426385	RBMXL3	RNA binding motif protein, X-	NM_001145346	G	A	nonsynonymous SNV	G2381A	R794H	M8	D	P	0.92
X	140993642	140993642	MAGEC1	Melanoma antigen family C1	NM_005462	C	T	nonsynonymous SNV	C452T	T151I	M8	T	B	
X	151909156	151909156	CSAG1	Chondrosarcoma associated	NM_153478	A	G	nonsynonymous SNV	A185G	K62R	M8	T	B	0.837
X	34148841	34148841	FAM47A	Family with sequence similari	NM_203408	A	G	nonsynonymous SNV	T1555C	S519P	M8	T	B	-0.44
X	35821055	35821055	MAGEB16	Melanoma antigen family B16	NM_001099921	A	G	nonsynonymous SNV	A742G	M248V	M8	D	B	-6.12
X	35821056	35821056	MAGEB16	Melanoma antigen family B16	NM_001099921	T	A	nonsynonymous SNV	T743A	M248K	M8	T	B	3.06
X	47426773	47426773	ARAF	A-Raf proto-oncogene, serine	NM_001654	G	T	nonsynonymous SNV	G1018T	V340L	M8	D	P	5.61
X	73641569	73641569	SLC16A2	Solute carrier family 16, mem	NM_006517	T	C	nonsynonymous SNV	T97C	S33P	M8	T	B	
7	140453136	140453136	BRAF	v-raf murine sarcoma viral or	NM_004333	A	T	nonsynonymous SNV	T1799A	V600E	M8	D	D	5.65
1	248801602	248801602	OR2T35	Olfattory Receptor Family 2, S	NM_001001827	0	CA	stopgain	957_958insTG	I320_R321delinsX	M8			
2	113588140	113588140	IL1B	Inteleukin 1, Beta	NM_000576	G	T	nonsynonymous SNV	C608A	P203H	M8	T	D	3.63
13	28498644	28498644	PDX1	Pancreatic And Duodenal Ho	NM_000209	G	C	nonsynonymous SNV	G658C	V220L	M8	T	B	3.12
11	44098842	44098842	ACCS	1-Aminocyclopropane-1-Carb	NM_001127219	C	G	nonsynonymous SNV	C570G	I190M	M8	D	P	3.87
12	51740409	51740409	CELA1	chymotrypsin-like elastase fa	NM_001971	T	G	nonsynonymous SNV	A14C	Y5S	M8	T	B	1.29
12	51740410	51740410	CELA1	chymotrypsin-like elastase fa	NM_001971	A	G	nonsynonymous SNV	T13C	Y5H	M8	T	B	2.28
14	23828948	23828948	EFS	embryonal Fyn-associated su	NM_005864	C	G	nonsynonymous SNV	G739C	G247R	M8	T	B	2.64
16	848780	848780	GNG13	guanine nucleotide binding pr	NM_016541	A	T	nonsynonymous SNV	T143A	L48Q	M8	D	D	5.07
19	2917807	2917807	ZNF57	Zinc Finger Protein 57	NM_173480	A	C	nonsynonymous SNV	A1188C	Q396H	M8	T	B	-4.5
1	11709274	11709274	FBXO2	F-box protein 2	NM_012168	C	A	nonsynonymous SNV	G626T	G209V	M9	T	D	3.29
1	120930062	120930062	FCGR1B	Fc fragment of IgG, high affin	NM_001017986.3	C	T	nonsynonymous SNV	G539A	G180E	M9	T	D	1.17
1	152127402	152127402	RPTN	repetin	NM_001122965	G	A	nonsynonymous SNV	C2173T	P725S	M9	T	B	1.7
1	153275022	153275022	PGLYRP3	peptidoglycan recognition pro	NM_052891	C	T	nonsynonymous SNV	G591A	M197I	M9	T	P	4.49
1	153932977	153932977	SLC39A1	solute carrier family 39 (zinc t	NM_014437.4	G	A	nonsynonymous SNV	C572T	S191F	M9	D	D	4.74
1	154295461	154295461	AQP10	aquaporin 10	NM_080429	C	A	nonsynonymous SNV	C236A	A79D	M9	D	D	5.04
1	167095836	167095836	DUSP27	dual specificity phosphatase :	NM_001080426	G	A	nonsynonymous SNV	G1468A	E490K	M9	D	P	5.36
1	168014350	168014350	DCAF6	DDB1 and CUL4 associated f	NM_001198956.1	C	T	nonsynonymous SNV	C2143T	P715S	M9	T	B	1.35
1	178442308	178442308	RASAL2	RAS protein activator like 2	NM_004841	C	G	nonsynonymous SNV	C3354G	I1118M	M9	T	P	4.78
1	180151396	180151396	QSOX1	quiescin Q6 sulfhydryl oxidas	NM_002826.4	A	G	nonsynonymous SNV	A694G	T232A	M9	T	B	2.9
1	183212376	183212376	LAMC2	laminin, gamma 2	NM_005562	G	A	nonsynonymous SNV	G3423A	M1141I	M9	T	B	3.12
1	193051735	193051735	TROVE2	TROVE domain family, memb	NM_001042369.2	C	T	nonsynonymous SNV	C1361T	A454V	M9	T	D	5.49
1	197404718	197404718	CRB1	crumbs family member 1, pho	NM_201253.2	G	A	nonsynonymous SNV	G3725A	G1242E	M9	T	B	3.51
1	214814895	214814895	CENPF	centromere protein F, 350/40	NM_016343	G	T	stopgain	G3214T	E1072X	M9	D		2.97
1	22174196	22174196	HSPG2	heparan sulfate proteoglycan	NM_001291860.1	G	A	nonsynonymous SNV	C8014T	P2672S	M9	T	D	5.13
1	235973162	235973162	LYST	lysosomal trafficking regulato	NM_000081.3	G	A	nonsynonymous SNV	C956T	P319L	M9	D	D	4.58
1	36205122	36205122	CLSPN	claspin	NM_022111.3	C	A	nonsynonymous SNV	G3152T	R1051M	M9	D	P	4.48
1	36785945	36785945	SH3D21	SH3 domain containing 21	NM_001162530.1	C	T	nonsynonymous SNV	C1681T	P561S	M9	T	B	-0.322
1	55598227	55598227	USP24	ubiquitin specific peptidase 24	NM_015306	G	T	stopgain	C3528A	Y1176X	M9	T		4.33
1	74507484	74507484	LRRIQ3	leucine-rich repeats and IQ m	NM_001105659	A	T	nonsynonymous SNV	T1131A	F377L	M9	T	B	0.396
1	82372743	82372743	LPHN2	adhesion G protein-coupled r	NM_012302.3	G	T	stopgain	G115T	E39X	M9	D		5.22
10	129908647	129908647	MKI67	marker of proliferation Ki-67	NM_002417.4	C	A	nonsynonymous SNV	G2411T	S804I	M9	T	B	-9.39
10	26446341	26446341	MYO3A	myosin IIIA	NM_017433	G	A	nonsynonymous SNV	G2896A	E966K	M9	T	P	5.31
10	48389122	48389122	RBP3	retinol binding protein 3, inter	NM_002900	C	T	nonsynonymous SNV	G1756A	E586K	M9	T	B	-2.05
10	50374978	50374978	C10orf128	chromosome 10 open reading	NM_001010863.2	C	T	nonsynonymous SNV	G174A	M58I	M9	D	B	2.27
10	50857639	50857639	CHAT	choline O-acetyltransferase	NM_020984.3	G	A	nonsynonymous SNV	G1114A	E372K	M9	T	B	5.04
10	54530495	54530495	MBL2	mannose-binding lectin (prote	NM_000242	C	T	nonsynonymous SNV	G239A	G80E	M9	D	D	4.98
10	64219504	64219504	ZNF365	zinc finger protein 365	NM_199451.2	G	A	stopgain	G929A	W310X	M9	T		1.16
10	96701634	96701634	CYP2C9	cytochrome P450, family 2, s	NM_000771	C	T	nonsynonymous SNV	C188T	P63L	M9	D	D	3.56
11	102573737	102573737	MMP27	matrix metallopeptidase 27	NM_022122	C	T	nonsynonymous SNV	G454A	G152R	M9	D	P	4.09
11	108380938	108380938	EXPH5	exophilin 5	NM_015065	C	T	nonsynonymous SNV	G5296A	V1766I	M9	T	B	-7.63
11	111956142	111956142	TIMM8B	translocase of inner mitochor	NM_012459	C	A	nonsynonymous SNV	G174T	K58N	M9	T	D	1.63
11	4127388	4127388	RRM1	ribonucleotide reductase M1	NM_001033	A	G	nonsynonymous SNV	A221G	Y74C	M9	D	D	5.77
11	47303131	47303131	MADD	MAP-kinase activating death	NM_003682.3	G	T	nonsynonymous SNV	G1296T	L432F	M9	T	D	-11.6
11	47510403	47510403	CELF1	CUGBP, Elav-like family mem	NM_006560.3	G	A	nonsynonymous SNV	C164T	P55L	M9	T	D	5.69
11	5536979	5536979	UBQLNL	ubiquilin-like	NM_145053	G	T	nonsynonymous SNV	C693A	N231K	M9	D	B	-1.8
11	56949505	56949505	LRRC55	leucine rich repeat containing	NM_001005210	C	A	nonsynonymous SNV	C138A	D46E	M9	T	B	3.07
11	57175311	57175311	SLC43A3	solute carrier family 43, mem	NM_014096.3	T	G	nonsynonymous SNV	A1430C	Y477S	M9	T	B	3.25
11	63532033	63532033	C11orf95	chromosome 11 open reading	NM_001144936	G	T	nonsynonymous SNV	C1062A	S354R	M9			

11	64375114	64375114	NRXN2	neurexin 2	NM_015080.3	T	A	nonsynonymous SNV	A4693T	M1565L	M9	D	P	2.2
11	65418056	65418056	SIPA1	signal-induced proliferation-a	NM_153253.29	C	T	nonsynonymous SNV	C2999T	G353S	M9	T	B	4.53
11	70028755	70028755	ANO1	anoctamin 1, calcium activate	NM_018043	G	A	nonsynonymous SNV	G2551A	D851N	M9		B	4.71
11	76910854	76910854	MYO7A	myosin VIIA	NM_000260.3	C	T	nonsynonymous SNV	C4843T	P1615S	M9	T	B	5.06
11	771348	771348	PDDC1	Parkinson disease 7 domain	NM_182612	C	T	nonsynonymous SNV	G529A	G177S	M9	D	D	4.44
11	78277192	78277192	NARS2	asparaginyl-tRNA synthetase	NM_024678	G	A	nonsynonymous SNV	C499T	H167Y	M9	D	D	5.13
11	84865626	84865626	DLG2	discs, large homolog 2 (Dros	NM_001142699	C	T	nonsynonymous SNV	G256A	E86K	M9	0	B	5
11	89133456	89133456	NOX4	NADPH oxidase 4	NM_016931.4	G	A	nonsynonymous SNV	C938T	S313L	M9	0	B	5.39
11	9829596	9829596	SBF2	SET binding factor 2	NM_030962	C	T	nonsynonymous SNV	G4394A	G1465E	M9	D	P	5.91
12	107371376	107371376	MTERF2	mitochondrial transcription te	NM_001033050.2	G	T	nonsynonymous SNV	C1117A	P373T	M9	D	D	5.95
12	11036756	11036756	PRH1	proline-rich protein HaeIII sub	NM_001291314	C	T	nonsynonymous SNV	G61A	E21K	M9	D		-2.49
12	112491427	112491427	NAA25	N(alpha)-acetyltransferase 25	NM_024953	G	A	stopgain	C1663T	Q555X	M9	T		5.99
12	11420548	11420548	PRB3	proline-rich protein BstNI sub	NM_006249	C	T	nonsynonymous SNV	G635A	G212E	M9	T	P	0.287
12	11420611	11420611	PRB3	proline-rich protein BstNI sub	NM_006249	C	T	nonsynonymous SNV	G572A	G191E	M9	D	B	-1.36
12	11420800	11420800	PRB3	proline-rich protein BstNI sub	NM_006249	C	T	nonsynonymous SNV	G383A	G128E	M9	D	P	0.707
12	116421171	116421171	MED13L	mediator complex subunit 13-	NM_015335	C	A	nonsynonymous SNV	G4706T	S1569I	M9	T	B	3.99
12	121471534	121471534	OASL	2'-5'-oligoadenylate synthetas	NM_003733.3	C	T	nonsynonymous SNV	G211A	G71R	M9	D	D	3.63
12	1250795	1250795	ERC1	ELKS/RAB6-interacting/CAST	NM_178040.3	C	T	nonsynonymous SNV	C1579T	L527F	M9	T	D	5.73
12	18854659	18854659	PLCZ1	phospholipase C, zeta 1	NM_033123	C	T	nonsynonymous SNV	G916A	E306K	M9	T	P	3.5
12	39711887	39711887	KIF21A	kinesin family member 21A	NM_001173464.1	A	G	nonsynonymous SNV	T3896C	V1299A	M9	T	P	4.69
12	44917186	44917186	NELL2	neural EGFL like 2	NM_001145107.1	C	T	nonsynonymous SNV	G2036A	R679Q	M9	T	D	4.93
12	5154412	5154412	KCNA5	potassium channel, voltage g	NM_002234	C	T	nonsynonymous SNV	C1099T	P367S	M9	D	D	4.87
12	52761138	52761138	KRT85	keratin 85, type II	NM_002283	A	G	nonsynonymous SNV	T52C	F18L	M9	D	B	4.63
12	53444023	53444023	TENC1	tensin 2	NM_170754	C	A	nonsynonymous SNV	C54A	D18E	M9	T	B	4.46
12	56493812	56493812	ERBB3	erb-b2 receptor tyrosine kinas	NM_001982	G	T	nonsynonymous SNV	G3128T	G1043V	M9	T	P	4.76
12	57865656	57865656	GLI1	GLI family zinc finger 1	NM_005269.2	A	G	nonsynonymous SNV	A3133G	T1045A	M9	T	B	5.04
12	71533592	71533592	TSPAN8	tetraspanin 8	NM_004616	C	T	nonsynonymous SNV	G160A	V54I	M9	T	B	-1.94
12	81101738	81101738	MYF6	myogenic factor 6 (herculin)	NM_002469	G	A	stopgain	G240A	W80X	M9	T		4.71
12	98909845	98909845	TMPO	thymopoietin	NM_003276.2	G	A	nonsynonymous SNV	G200A	S67N	M9	D	D	4.51
13	28141800	28141800	LNX2	ligand of numb-protein X 2	NM_153371	G	A	nonsynonymous SNV	C832T	L278F	M9	T	D	5.62
13	77713338	77713338	MYCBP2	MYC binding protein 2, E3 ub	NM_015057	G	A	nonsynonymous SNV	C7652T	P2551L	M9	D	P	5.48
14	105398097	105398097	PLD4	phospholipase D family, mem	NM_138790	G	A	nonsynonymous SNV	G931A	A311T	M9	T	B	-0.434
14	24526379	24526379	LRRC16B	leucine rich repeat containing	NM_138360	C	T	nonsynonymous SNV	C1066T	L356F	M9	D	D	5.12
14	31348664	31348664	COCH	cochlin	NM_001135058.1	G	T	nonsynonymous SNV	G409T	A137S	M9	T	B	3.92
14	64587770	64587770	SYNE2	spectrin repeat containing, nu	NM_182914.2	G	T	nonsynonymous SNV	G13149T	Q4383H	M9	T	D	-2.29
14	70634428	70634428	SLC8A3	solute carrier family 8 (sodiun	NM_183002.2	G	A	nonsynonymous SNV	C712T	L238F	M9	T	D	5.71
14	88945802	88945802	PTPN21	protein tyrosine phosphatase	NM_007039	G	A	nonsynonymous SNV	C1973T	S658F	M9	D	P	5.04
14	89085083	89085083	EML5	echinoderm microtubule asso	NM_183387	C	T	nonsynonymous SNV	G5449A	G1817S	M9	D	D	5.74
14	90752729	90752729	NRDE2	NRDE-2, necessary for RNA	NM_017970	G	A	nonsynonymous SNV	C3266T	P1089L	M9	T	P	5.59
14	94394604	94394604	FAM181A	family with sequence similarit	NM_138344	G	A	stopgain	G159A	W53X	M9	T		4.63
15	39884982	39884982	THBS1	thrombospondin 1	NM_003246	C	T	nonsynonymous SNV	C2746T	P916S	M9	D	D	5.3
15	42058627	42058627	MGA	MGA, MAX dimerization prote	NM_001164273.1	C	T	nonsynonymous SNV	C8347T	L2783F	M9	T	P	3.51
15	64701993	64701993	TRIP4	thyroid hormone receptor inte	NM_016213	G	A	nonsynonymous SNV	G1009A	E337K	M9	T	B	4.9
15	65391965	65391965	UBAP1L	ubiquitin associated protein 1	NM_001163692	C	G	nonsynonymous SNV	G787C	D263H	M9	D	D	5.4
15	68612707	68612707	ITGA11	integrin, alpha 11	NM_001004439	C	T	nonsynonymous SNV	G2432A	R811K	M9	T	B	4.7
15	75557697	75557697	GOLGA6C	golgin A6 family, member C	NM_001164404	G	A	nonsynonymous SNV	G691A	E231K	M9	T	B	-0.334
15	89010418	89010418	MRPL46	mitochondrial ribosomal prote	NM_022163	G	T	nonsynonymous SNV	C191A	T64N	M9	T	B	0.789
16	1129868	1129868	SSTR5	somatostatin receptor 5	NM_001053.3	G	A	nonsynonymous SNV	G1000A	E334K	M9	T	B	4.49
16	1545618	1545618	TELO2	telomere maintenance 2	NM_016111	C	T	nonsynonymous SNV	C607T	L203F	M9	T	D	5.33
16	24950860	24950860	ARHGAP17	Rho GTPase activating prote	NM_001006634	T	C	nonsynonymous SNV	A1549G	K517E	M9	T	D	5
16	27549603	27549603	GTF3C1	general transcription factor III	NM_001520.3	G	A	nonsynonymous SNV	C506T	P169L	M9	T	P	5.5
16	2905806	2905806	PRSS22	protease, serine, 22	NM_022119	G	C	nonsynonymous SNV	C328G	Q110E	M9	T	B	2.21
16	30736315	30736315	SRCAP	Snf2-related CREBBP activat	NM_006662	C	T	nonsynonymous SNV	C5570T	P1857L	M9	T	D	5.91
16	630868	630868	PIGQ	phosphatidylinositol glycan ar	NM_148920.2	T	A	nonsynonymous SNV	T1427A	L476Q	M9	D	D	5.22
16	67854818	67854818	TSNAXIP1	translin-associated factor X ir	NM_001288990.1	G	A	nonsynonymous SNV	G224A	R75Q	M9	T	B	3.03
16	68941449	68941449	TANGO6	transport and golgi organizati	NM_024562	G	T	nonsynonymous SNV	G1771T	A591S	M9	T	D	5.56
16	71713321	71713321	PHLPP2	PH domain and leucine rich r	NM_015020.3	G	T	nonsynonymous SNV	C1008A	D336E	M9	T	B	2.93
16	848768	848768	GNG13	guanine nucleotide binding pr	NM_016541	A	T	nonsynonymous SNV	T155A	L52Q	M9	T	D	5.07
17	10429959	10429959	MYH2	myosin, heavy chain 2, skelet	NM_017534.5	C	T	nonsynonymous SNV	G4144A	E1382K	M9	D	D	5.2
17	26887110	26887110	PIGS	phosphatidylinositol glycan ar	NM_033198	A	G	nonsynonymous SNV	T776C	L259P	M9	D	D	5.84
17	29562746	29562746	NF1	neurofibromin 1	NM_001042492	C	T	stopgain	C3826T	R1276X	M9	T		5.15
17	39316677	39316677	KRTAP4-4	keratin associated protein 4-4	NM_032524	T	G	nonsynonymous SNV	A267C	Q89H	M9	D	B	-2.2
17	39525831	39525831	KRT33B	keratin 33B, type I	NM_002279	C	T	nonsynonymous SNV	G172A	E58K	M9	D	D	4.44
17	39977230	39977230	FKBP10	FK506 binding protein 10, 65	NM_021939	C	T	nonsynonymous SNV	C1288T	L430F	M9	D	D	3.22
17	41143029	41143029	RUNDC1	RUN domain containing 1	NM_173079	C	G	nonsynonymous SNV	C1138G	P380A	M9	T	B	4.28
17	41477288	41477288	ARL4D	ADP-ribosylation factor-like 4	NM_001661	C	T	nonsynonymous SNV	C188T	P63L	M9	D	B	3.82
17	44095984	44095984	MAPT	microtubule-associated prote	NM_001123066.3	G	A	nonsynonymous SNV	G2003A	G668E	M9		D	5.02

17	73491064	73491064	KIAA0195	KIAA0195	NM_014738	C	A	nonsynonymous SNV	C2677A	P893T	M9	T	B	5.79
17	79517454	79517454	C17orf70	Fanconi anemia core comple	NM_025161	C	T	nonsynonymous SNV	G1066A	G356S	M9	T	B	-1.86
17	8007492	8007492	ALOXE3	arachidonate lipoxygenase 3	NM_001165960.1	A	G	nonsynonymous SNV	T2122C	F708L	M9	D	P	4.57
18	31324974	31324974	ASXL3	additional sex combs like trar	NM_030632	C	T	nonsynonymous SNV	C5162T	S1721F	M9	D	D	5.86
19	12125781	12125781	ZNF433	zinc finger protein 433	NM_001080411	C	A	nonsynonymous SNV	G1901T	E652D	M9	T	B	0.343
19	14499537	14499537	CD97	adhesion G protein-coupled r	NM_078481	A	G	nonsynonymous SNV	A97G	N33D	M9	T	B	0.967
19	21992253	21992253	ZNF43	zinc finger protein 43	NM_001256648.1	G	A	nonsynonymous SNV	C568T	H190Y	M9	T	B	0.283
19	36216411	36216411	KMT2B	lysine (K)-specific methyltran	NM_014727	T	C	nonsynonymous SNV	T3674C	F1225S	M9	D	D	5.54
19	41355828	41355828	CYP2A6	cytochrome P450, family 2, s	NM_000762	C	T	nonsynonymous SNV	G238A	V80M	M9	T	P	2.72
19	43372986	43372986	PSG1	pregnancy specific beta-1-gly	NM_001297773.1	C	T	nonsynonymous SNV	G910A	E304K	M9	D	D	0.535
19	49671906	49671906	TRPM4	transient receptor potential ca	NM_017636.3	G	A	nonsynonymous SNV	G709A	D237N	M9	T	P	4.49
19	49913070	49913070	CCDC155	coiled-coil domain containing	NM_144688	G	A	nonsynonymous SNV	G1207A	E403K	M9	T	P	3.9
19	50193376	50193376	ADM5	adrenomedullin 5 (putative)	NM_001101340	C	A	nonsynonymous SNV	C88A	Q30K	M9	D	P	1.45
19	55607508	55607508	PPP1R12C	protein phosphatase 1, regula	NM_017607.3	G	A	nonsynonymous SNV	C1064T	S355F	M9	T	D	4.59
19	5687908	5687908	HSD11B1L	hydroxysteroid (11-beta) dehy	NM_001267870	G	A	nonsynonymous SNV	G478A	G160S	M9			
19	7830731	7830731	CLEC4M	C-type lectin domain family 4	NM_014257.4	G	T	nonsynonymous SNV	G422T	R141L	M9	T	B	-1.81
19	9058414	9058414	MUC16	mucin 16, cell surface associ	NM_024690	G	A	nonsynonymous SNV	C29032T	P9678S	M9	D	B	-6.24
19	9075243	9075243	MUC16	mucin 16, cell surface associ	NM_024690	G	A	nonsynonymous SNV	C12203T	S4068L	M9	D	B	0.103
19	9086161	9086161	MUC16	mucin 16, cell surface associ	NM_024690	G	A	nonsynonymous SNV	C5654T	S1885F	M9	D	D	0.225
2	113416608	113416608	SLC20A1	solute carrier family 20 (phos	NM_005415	G	A	nonsynonymous SNV	G985A	E329K	M9	T	B	5.9
2	128262665	128262665	IWS1	IWS1 homolog (S. cerevisiae	NM_017969	G	A	stopgain	C814T	R272X	M9	T		4.84
2	139322555	139322555	SPOPL	speckle-type POZ protein-like	NM_001001664	G	A	stopgain	G1026A	W342X	M9			3.93
2	166231294	166231294	SCN2A	sodium channel, voltage gate	NM_021007.2	C	T	nonsynonymous SNV	C4072T	L1358F	M9	D	D	4.41
2	17697627	17697627	RAD51AP2	RAD51 associated protein 2	NM_001099218	C	T	nonsynonymous SNV	G2056A	E686K	M9	D	B	-0.446
2	176987569	176987569	HOXD9	homeobox D9	NM_014213	C	A	nonsynonymous SNV	C73A	L25I	M9	T	B	4.3
2	179456753	179456753	TTN	titin	NM_001267550	C	A	nonsynonymous SNV	G59878T	G19960C	M9	D	D	6.03
2	179613473	179613473	TTN	titin	NM_133379	C	T	nonsynonymous SNV	G13654A	E4552K	M9	T	B	0.829
2	207173658	207173658	ZDBF2	zinc finger, DBF-type containi	NM_020923.2	C	T	nonsynonymous SNV	C4406T	P1469L	M9	D	P	2.84
2	219000551	219000551	CXCR2	chemokine (C-X-C motif) rece	NM_001557.3	G	A	nonsynonymous SNV	G1027A	D343N	M9		B	4.46
2	228846549	228846549	SPHKAP	SPHK1 interactor, AKAP dom	NM_001142644.1	G	A	nonsynonymous SNV	C4987T	R1663W	M9	D	P	5.85
2	24413336	24413336	FAM228A	family with sequence similarit	NM_001040710	G	A	nonsynonymous SNV	G457A	A153T	M9	T	B	-6.61
2	85924668	85924668	GNLY	granulysin	NM_006433	G	A	nonsynonymous SNV	G295A	G99R	M9	T	B	-4.21
2	88409904	88409904	SMYD1	SET and MYND domain cont	NM_198274	G	A	nonsynonymous SNV	G1346A	R449H	M9	T	P	5.41
20	44596264	44596264	ZNF335	zinc finger protein 335	NM_022095	G	A	nonsynonymous SNV	C824T	A275V	M9	T	B	4.78
20	62338017	62338017	ARFRP1	ADP-ribosylation factor relate	NM_003224.5	G	A	nonsynonymous SNV	C167T	T56I	M9	D	D	4.92
20	62614539	62614539	PRPF6	pre-mRNA processing factor	NM_012469	G	A	nonsynonymous SNV	G211A	E71K	M9	T	P	5.22
21	19666683	19666683	TMPRSS15	transmembrane protease, se	NM_002772	G	A	nonsynonymous SNV	C2390T	P797L	M9		D	5.79
21	41459131	41459131	DSCAM	Down syndrome cell adhesio	NM_001389.3	C	T	nonsynonymous SNV	G3934A	D1312N	M9	T	D	4.71
21	47754641	47754641	PCNT	pericentrin	NM_006031	A	T	nonsynonymous SNV	A598T	I200F	M9	T	B	-0.47
22	23915574	23915574	IGLL1	immunoglobulin lambda-like p	NM_020070	G	A	nonsynonymous SNV	C521T	A174V	M9	T	B	-4.9
22	24379386	24379386	GSTT1	glutathione S-transferase the	NM_000853	C	T	nonsynonymous SNV	G326A	S109N	M9	T	B	-1.94
22	26164985	26164985	MYO18B	myosin XVIIIB	NM_032608	G	A	nonsynonymous SNV	G1102A	D368N	M9	T	B	0.318
22	26423366	26423366	MYO18B	myosin XVIIIB	NM_032608	G	A	nonsynonymous SNV	G7426A	E2476K	M9	D	B	2.7
22	33733727	33733727	LARGE	like-glycosyltransferase	NM_004737.4	G	A	nonsynonymous SNV	C1192T	R398C	M9	D	D	4.21
22	38130829	38130829	TRIOBP	TRIO and F-actin binding pro	NM_001039141	G	A	nonsynonymous SNV	G4486A	E1496K	M9	D	B	4.25
22	39085040	39085040	JOSD1	Josephin domain containing 1	NM_014876	T	A	nonsynonymous SNV	A409T	R137W	M9	D	D	5.63
3	121647356	121647356	SLC15A2	solute carrier family 15 (oligo	NM_021082.3	G	A	nonsynonymous SNV	G1295A	G432E	M9	T	D	2.79
3	127295837	127295837	TPRA1	transmembrane protein, adip	NM_001136053.2	T	A	nonsynonymous SNV	A341T	D114V	M9	D	D	4.33
3	132345585	132345585	ACAD11	acyl-CoA dehydrogenase fam	NM_032169	G	A	nonsynonymous SNV	C1147T	L383F	M9	T	D	5.31
3	138413734	138413734	PIK3CB	phosphatidylinositol-4,5-bisph	NM_006219.2	G	T	nonsynonymous SNV	C1786A	Q596K	M9	T	B	5.04
3	164735772	164735772	SI	sucrase-isomaltase (alpha-gl	NM_001041	T	C	nonsynonymous SNV	A3506G	N1169S	M9	D	D	5.17
3	179051969	179051969	ZNF639	zinc finger protein 639	NM_016331	A	G	nonsynonymous SNV	A1217G	D406G	M9	T	D	5.78
3	184953248	184953248	EHHADH	enoyl-CoA, hydratase/3-hydr	NM_001966	C	A	nonsynonymous SNV	G181T	A61S	M9	D	D	5.2
3	193042697	193042697	ATP13A5	ATPase type 13A5	NM_198505	G	C	nonsynonymous SNV	C1630G	Q544E	M9	T	D	5.34
3	37067242	37067242	MLH1	mutL homolog 1	NM_000249.3	C	T	nonsynonymous SNV	C1153T	R385C	M9	D	D	5.67
3	38647551	38647551	SCN5A	sodium channel, voltage gate	NM_198056.2	G	A	nonsynonymous SNV	C1229T	A410V	M9	D	D	5.54
3	40557940	40557940	ZNF620	zinc finger protein 620	NM_175888.3	C	A	stopgain	C855A	C285X	M9			-5.66
3	44308497	44308497	TOPAZ1	testis and ovary specific PAZ	NM_001145030	C	A	nonsynonymous SNV	C3029A	S1010Y	M9	D	P	2.26
3	50009492	50009492	RBM6	RNA binding motif protein 6	NM_005777	G	T	nonsynonymous SNV	G1324T	D442Y	M9		D	5.69
3	51864605	51864605	IQCF3	IQ motif containing F3	NM_001085479.2	G	A	nonsynonymous SNV	G253A	V85I	M9	T	B	-9.45
3	51895692	51895692	IQCF2	IQ motif containing F2	NM_203424	C	T	stopgain	C10T	R4X	M9	T	0	-2.8
3	57488191	57488191	DNAH12	Dynein, axonemal, heavy cha	NM_198564	G	A	nonsynonymous SNV	C1102T	P368S	M9	T	B	2.24
3	74570229	74570229	CNTN3	Contactin 3 (plasmacytoma a	NM_020872	G	A	nonsynonymous SNV	C35T	S12L	M9	T	B	5.2
3	98002107	98002107	OR5H2	Olfactory receptor, family 5, s	NM_001005482	G	A	nonsynonymous SNV	G376A	D126N	M9	D	D	2.31
4	103578946	103578946	MANBA	Mannosidase, beta A, lysoso	NM_005908	A	T	nonsynonymous SNV	T1597A	F533I	M9	D	P	5.32
4	15937750	15937750	FGFBP1	Fibroblast growth factor bindi	NM_005130	G	A	nonsynonymous SNV	C506T	S169F	M9	D	B	0.561
4	166915602	166915602	TLL1	Tolloid-like 1	NM_012464	G	A	nonsynonymous SNV	G431A	R144Q	M9	D	D	5.51

4	166978441	166978441	TLL1	Tolloid-like 1	NM_012464	C	T	nonsynonymous SNV	C1826T	P609L	M9	T	D	5.13
4	183674657	183674657	TENM3	Teneurin transmembrane pro	NM_001080477	G	A	nonsynonymous SNV	G3917A	R1306K	M9	D	D	5.65
4	183674669	183674669	TENM3	Teneurin transmembrane pro	NM_001080477	A	C	nonsynonymous SNV	A3929C	Q1310P	M9	T	B	4.44
4	183714955	183714955	TENM3	Teneurin transmembrane pro	NM_001080477	A	G	nonsynonymous SNV	A7130G	K2377R	M9	T	B	3.69
4	189022345	189022345	TRIML2	Tripartite motif family-like 2	NM_173553	C	A	nonsynonymous SNV	G195T	L65F	M9	T	P	3.33
4	22414813	22414813	GPR125	adhesion G protein-coupled r	NM_145290	C	A	nonsynonymous SNV	G2224T	V742F	M9	D	D	5.49
4	77661967	77661967	SHROOM3	Shroom family member 3	NM_020859	C	T	nonsynonymous SNV	C2641T	P881S	M9	T	B	4.48
4	79308532	79308532	FRAS1	Fraser extracellular matrix co	NM_025074	C	T	nonsynonymous SNV	C3652T	P1218S	M9	D	D	5.6
4	79443916	79443916	FRAS1	Fraser extracellular matrix co	NM_025074	T	G	nonsynonymous SNV	T10762G	F3588V	M9	D	B	5.81
4	80328964	80328964	GK2	Glycerol kinase 2	NM_033214	G	A	nonsynonymous SNV	C391T	P131S	M9	T	B	3.76
5	110448846	110448846	WDR36	WD repeat domain 36	NM_139281	C	T	nonsynonymous SNV	C1958T	S653F	M9	D	D	5.76
5	11346487	11346487	CTNND2	Catenin (cadherin-associated	NM_001288717	G	A	nonsynonymous SNV	C326T	P109L	M9	D	P	5.71
5	11346488	11346488	CTNND2	Catenin (cadherin-associated	NM_001288717	G	A	nonsynonymous SNV	C325T	P109S	M9	D	B	5.71
5	121761218	121761218	SNCAIP	Synuclein, alpha interacting p	NM_001242935	G	T	nonsynonymous SNV	G1174T	A392S	M9	T	D	5.67
5	13716648	13716648	DNAH5	Dynein, axonemal, heavy cha	NM_001369	C	T	nonsynonymous SNV	G12857A	G4286E	M9	D	P	5.53
5	149629841	149629841	CAMK2A	Calcium/calmodulin-depende	NM_171825	C	G	nonsynonymous SNV	G848C	R283T	M9	D	D	5.38
5	153078566	153078566	GRIA1	Glutamate receptor, ionotropi	NM_000827	G	A	nonsynonymous SNV	G1385A	G462E	M9	D	D	5.44
5	161116720	161116720	GABRA6	Gamma-aminobutyric acid (G	NM_000811	C	T	nonsynonymous SNV	C608T	S203L	M9	T	P	5.41
5	167891720	167891720	WWC1	WW and C2 domain containir	NM_001161661	C	G	nonsynonymous SNV	C2921G	S974C	M9	T	B	1.97
5	168180975	168180975	SLIT3	slit homolog 3	NM_001271946	C	T	nonsynonymous SNV	G1723A	G575R	M9	D	D	5.31
5	175813910	175813910	NOP16	NOP16 nucleolar protein	NM_001256539	C	T	nonsynonymous SNV	G217A	V73M	M9	D	D	5.42
5	61875707	61875707	LRRC70	Leucine rich repeat containin	NM_181506	C	T	nonsynonymous SNV	C442T	P148S	M9		B	-0.296
5	840644	840644	ZDHHC11	Zinc finger, DHHC-type conta	NM_024786	G	C	nonsynonymous SNV	C750G	H250Q	M9	T	B	-0.394
5	86685331	86685331	RASA1	RAS p21 protein activator	NM_002890	G	A	nonsynonymous SNV	G3047A	R1016H	M9	T	P	5.75
5	90050857	90050857	GPR98	Adhesion G protein-coupled r	NM_032119	T	C	nonsynonymous SNV	T11435C	I3812T	M9	D	B	5.73
6	109483990	109483990	CEP57L1	Centrosomal protein 57kDa-li	NM_001271852	G	T	nonsynonymous SNV	G1200T	M400I	M9	T	B	4.28
6	150710577	150710577	IYD	Iodotyrosine deiodinase	NM_001164694	T	C	nonsynonymous SNV	T268C	S90P	M9	D	D	2.93
6	43745314	43745314	VEGFA	vascular endothelial growth fa	NM_001025366	C	T	nonsynonymous SNV	C767T	S256F	M9	D	D	4.77
6	46979767	46979767	GPR110	Adhesion G Protein-Coupled	NM_153840	G	T	nonsynonymous SNV	C1092A	F364L	M9	T	B	3.17
7	121650421	121650421	PTPRZ1	Protein tyrosine phosphatase	NM_001206838	G	T	nonsynonymous SNV	G1321T	A441S	M9	T	B	-3.99
7	12617689	12617689	SCIN	Scinderin	NM_001112706	G	A	nonsynonymous SNV	G200A	G67E	M9	D	D	4.91
7	138437531	138437531	ATP6V0A4	ATPase, H+ transporting, lyso	NM_020632	C	A	nonsynonymous SNV	G868T	A290S	M9	T	B	2.78
7	140373452	140373452	ADCK2	AarF Domain Containing Kina	NM_052853	G	A	nonsynonymous SNV	G322A	V108M	M9	T	B	0.255
7	141752660	141752660	MGAM	Maltase-Glucoamylase	NM_004668	C	T	nonsynonymous SNV	C3035T	S1012F	M9	D	P	4.2
7	150499315	150499315	TMEM176A	Transmembrane Protein 176A	NM_018487	G	A	nonsynonymous SNV	G187A	V63M	M9	D	B	0.671
7	**150741103**	150741103	ABCB8	TP-binding cassette, sub-fam	NM_001282291	C	T	nonsynonymous SNV	C1862T	A621V	M9	D	D	4.89
7	155537826	155537826	RBM33	RNA Binding Motif Protein 33	NM_053043	C	G	nonsynonymous SNV	C2509G	P837A	M9	T	B	1.95
7	156752603	156752603	NOM1	Nucleolar Protein With MIF4G	NM_138400	G	A	nonsynonymous SNV	G1367A	G456E	M9	T	B	4.93
7	29994949	29994949	SCRN1	Secernin 1	NM_001145514	G	A	nonsynonymous SNV	C247T	P83S	M9	B	D	5.7
7	36895167	36895167	ELMO1	engulfment and cell motility 1	NM_001206480	C	A	nonsynonymous SNV	G2173T	D725Y	M9	D	P	4.77
7	50611657	50611657	DDC	Dopa decarboxylase (aromat	NM_001082971	G	A	nonsynonymous SNV	C127T	P43S	M9	D	D	5.92
7	72416199	72416199	POM121	POM121 transmembrane nuc	NM_001257190	G	A	nonsynonymous SNV	G2809A	G937R	M9	D	P	3.16
7	82582489	82582489	PCLO	Piccolo presynaptic cytomatri	NM_033026	C	T	nonsynonymous SNV	G7780A	D2594N	M9	D	D	4.8
8	108359286	108359286	ANGPT1	Angiopoietin 1	NM_001146	C	T	nonsynonymous SNV	G337A	A113T	M9	T	B	5.95
8	121522268	121522268	MTBP	MDM2 binding protein	NM_022045	G	A	nonsynonymous SNV	G1978A	E660K	M9	D	D	6.07
8	134063092	134063092	SLA	Src-like-adaptor	NM_006748	G	A	nonsynonymous SNV	C250T	R84C	M9	T	D	4.53
8	145731960	145731960	GPT	Glutamic-pyruvate transamina	NM_005309	T	C	nonsynonymous SNV	T1208C	I403T	M9	D	P	4.54
8	24242027	24242027	ADAMDEC1	ADAM-like, decysin 1	NM_014479	G	A	nonsynonymous SNV	G10A	G4R	M9	T	B	1.87
8	28635402	28635402	INTS9	Integrator complex subunit 9	NM_018250	G	A	nonsynonymous SNV	C1339T	P447S	M9	T	D	5.28
8	30703646	30703646	TEX15	Testis expressed 15	NM_031271	C	T	nonsynonymous SNV	G2888A	R963Q	M9	D	P	4.75
8	3443732	3443732	CSMD1	CUB and Sushi multiple doma	NM_033225	C	T	nonsynonymous SNV	G1148A	G383E	M9	D	D	5.77
8	37732756	37732756	RAB11FIP1	RAB11 family interacting prot	:NM_001002814	G	A	nonsynonymous SNV	C899T	S300F	M9	D	D	5.13
8	37985941	37985941	ASH2L	Ash2 (absent, small, or home	NM_004674	A	G	nonsynonymous SNV	A1298G	D433G	M9	T	B	4.62
8	41790698	41790698	KAT6A	K(lysine) acetyltransferase 6A	NM_001099412	T	A	nonsynonymous SNV	A5040T	Q1680H	M9	D	B	0.465
8	43152560	43152560	POTEA	POTE ankyrin domain family,	NM_001005365	A	T	nonsynonymous SNV	A546T	K182N	M9			
8	81889089	81889089	PAG1	Phosphoprotein membrane a	NM_018440	G	A	nonsynonymous SNV	C989T	S330L	M9	T	B	1.53
9	104432571	104432571	GRIN3A	Glutamate receptor, ionotropi	NM_133445	C	T	nonsynonymous SNV	G2123A	R708Q	M9	T	D	5.39
9	105767730	105767730	CYLC2	Cylicin, basic protein of sperr	NM_001340	G	A	nonsynonymous SNV	G817A	D273N	M9	T	P	3.69
9	115456415	115456415	INIP	INTS3 and NABP interacting	NM_021218	C	A	nonsynonymous SNV	G124T	A42S	M9	D	D	5.6
9	122075589	122075589	BRINP1	Bone Morphogenetic Protein/	NM_014618	C	T	stopgain	G45A	W15X	M9	T		5.39
9	123739159	123739159	C5	Complement Component 5	NM_001735	C	T	stopgain	G3683A	W1228X	M9	T		5.66
9	125315999	125315999	OR1N2	Olfactory Receptor, Family 1,	NM_001004457	C	T	nonsynonymous SNV	C551T	A184V	M9	D	B	0.204
9	130605443	130605443	ENG	Endoglin	NM_001114753	G	A	nonsynonymous SNV	C149T	S50L	M9	T	B	4.23
9	134019675	134019675	NUP214	Nucleoporin 214kDa	NM_005085	C	T	nonsynonymous SNV	C1303T	P435S	M9	T	B	4.67
9	137715314	137715314	COL5A1	Collagen, type V, alpha 1	NM_000093	C	A	nonsynonymous SNV	C4697A	P1566Q	M9	D	D	5.14
9	32633060	32633060	TAF1L	TAF1 RNA Polymerase II, TA	NM_153809	G	A	nonsynonymous SNV	C2518T	R840W	M9	D	D	1.19
9	35042957	35042957	C9orf131	Chromosome 9 open reading	NM_001040410	G	A	nonsynonymous SNV	G226A	E76K	M9	T	B	0.151

9	35650537	35650537	SIT1	Signaling Threshold Regulati	NM_014450	C	A	nonsynonymous SNV	G198T	W66C	M9	T	B	1.93
9	36105250	36105250	RECK	Reversion-Inducing-Cysteine	NM_021111	G	A	nonsynonymous SNV	G1546A	D516N	M9	T	B	5.34
9	74489983	74489983	ABHD17B	Abhydrolase domain containi	NM_001025780	G	A	nonsynonymous SNV	C14T	S5L	M9	D	P	5.93
9	90501587	90501587	SPATA31E1	SPATA31 subfamily E, memb	NM_178828	G	T	nonsynonymous SNV	G2185T	A729S	M9	T	B	-0.747
X	102004582	102004582	BHLHB9	Basic helix-loop-helix domain	NM_001142524	G	A	stopgain	G659A	W220X	M9			4.35
X	102004583	102004583	BHLHB9	Basic helix-loop-helix domain	NM_001142524	G	A	stopgain	G660A	W220X	M9			4.35
X	103268115	103268115	H2BFWT	2B histone family, member W	NM_001002916	C	T	nonsynonymous SNV	G118A	E40K	M9	T	B	1.56
X	114141258	114141258	HTR2C	5-hydroxytryptamine (seroton	NM_001256761	C	T	nonsynonymous SNV	C562T	L188F	M9	D	B	4.87
X	48319389	48319389	SLC38A5	Solute carrier family 38, mem	NM_033518	C	T	nonsynonymous SNV	G935A	G312E	M9	D	D	4.24
X	48762537	48762537	SLC35A2	Solute carrier family 35 (UDP	NM_001282651	C	T	nonsynonymous SNV	G733A	G245S	M9	T	D	5.33
X	53280139	53280139	IQSEC2	IQ motif and Sec7 domain 2	NM_015075	T	G	nonsynonymous SNV	A1004C	Q335P	M9	T	B	4.26
X	84362485	84362485	SATL1	Spermidine/spermine N1-ace	NM_001012980	T	A	nonsynonymous SNV	A1490T	N497I	M9	T	B	-2.54
X	99662155	99662155	PCDH19	Protocadherin 19	NM_001184880	C	T	nonsynonymous SNV	G1441A	D481N	M9	D	D	5.64
7	140453136	140453136	BRAF	v-raf murine sarcoma viral or	NM_004333	A	T	nonsynonymous SNV	T1799A	V600E	M9	D	D	5.65
7	100645960	100645960	MUC12	Mucin12	NM_001164462	A	T	nonsynonymous SNV	A12116T	D4039V	M9	T		-1.72
1	248801602	248801602	OR2T35	Olfattory Receptor Family 2, S	NM_001001827	0	CA	stopgain	957_958insTG	I320_R321delinsX	M9			
12	109017374	109017374	SELPLG	Selectin P Ligand	NM_001206609	T	G	nonsynonymous SNV	A758C	E253A	M9	T	B	-5.23
7	57529086	57529086	ZNF716	Zinc Finger Protein 716	NM_001159279	C	A	nonsynonymous SNV	C919A	R307S	M9	T	B	-218
17	7483366	7483366	CD68	CD68 Molecule	NM_001251	T	A	nonsynonymous SNV	T288A	N96K	M9	T	B	-5.96
11	618683	618683	CDHR5	Cadherin-Related Family Mei	NM_021924	T	A	nonsynonymous SNV	A1876T	T626S	M9	T	B	592
7	100639761	100639761	MUC12	Mucin12	NM_001164462	G	C	nonsynonymous SNV	G5917C	D1973H	M9	T		-275
7	44146353	44146353	AEBP1	AE binding protein 1	NM_001129	A	T	nonsynonymous SNV	A462T	K154N	M9	D	D	-0.51
1	94001896	94001896	FNBP1L	Formin Binding Protein 1-Like	NM_001164473	C	G	nonsynonymous SNV	C998G	S333C	M9	D	B	5.16
15	22743461	22743461	GOLGA6L1	golgin A6 family-like 1	NM_001001413	T	A	nonsynonymous SNV	T1846A	W616R	M9	T		
15	22743461	22743461	GOLGA6L22	golgin A6 family-like 22	NM_001271664	T	A	nonsynonymous SNV	T1810A	W604R	M9	T		
11	48511193	48511193	OR4A47	olfactory receptor, family 4, su	NM_001005512	A	T	nonsynonymous SNV	A849T	L283F	M9	T	B	-451
19	1440068	1440068	RPS15	ribosomal protein S15	NM_001018	G	C	nonsynonymous SNV	G140C	R47P	M9	T	B	3.21

Supplementary Table S4

Small InDels

Chr	Start	End	Gene symbol	Gene dscription	Accession	Reference A	Variant Allele	Variant classification	cDNA change	Protein change	Sample
20	126311	126312	DEFB126	Defensin, beta 126	NM_030931	CC	0	frameshift deletion	314_315del	T105fs	M1
5	74021847	74021852	GFM2	G elongation factor, mitochondrial 2	NM_032380	ACTCAA	0	nonframeshift deletion	1826_1831del	609_611del	M1
15	72455650	72455667	GRAMD2	GRAM domain containing 2	NM_001012642	GCTCCCCA	0	nonframeshift deletion	896_913del	299_305del	M1
1	175129925	175129933	KIAA0040	KIAA0040	NM_001162893	CTTCTTCT	0	nonframeshift deletion	217_225del	73_75del	M1
17	20370782	20370782	LGALS9B	Lectin, galactoside-binding, soluble, 9B	NM_001042685	0	TC	frameshift insertion	1_2insGA	M1fs	M1
1	26608820	26608843	UBXN11	UBX domain protein 11	NM_145345	GGGACTGC	0	nonframeshift deletion	1411_1434del	471_478del	M1
18	56204388	56204402	ALPK2	alpha-kinase 2	NM_052947	CAGTTGAT	0	nonframeshift deletion	3017_3031del	1006_1011del	M1
8	11666219	11666224	FDFT1	Farnesyl-Diphosphate Farnesyltransfera	NM_001287750	TCCCAC	0	nonframeshift deletion	193_198del	65_66del	M1
12	53069223	53069243	KRT1	keratin 1	NM_006121	ACCTCCGG	0	nonframeshift deletion	1669_1689del	557_563del	M1
11	95825375	95825383	MAML2	Mastermind-Like 2	NM_032427	TGCTGCTG	0	nonframeshift deletion	1812_1820del	604_607del	M1
1	209605637	209605648	MIR205HG	MIR205 host gene (non-protein coding)		AGCAGCAG	0	nonframeshift deletion	252_263del	84_88del	M1
11	48346661	48346669	OR4C3	olfactory receptor, family 4, subfamily C	NM_001004702	TTGCTGAT	0	nonframeshift deletion	169_177del	57_59del	M1
20	238436	238441	DEFB132	defensin, beta 132	NM_207469	TGGTCT	0	nonframeshift deletion	17_22del	6_8del	M1
16	71956512	71956517	IST1	increased sodium tolerance 1 homolog	NM_001270979	ATGCCC	0	nonframeshift deletion	244_249del	82_83del	M1
13	25671273	25671273	PABPC3	poly(A) binding protein, cytoplasmic 3	NM_030979	G	0	frameshift deletion	937delG	A313fs	M1
1	153233991	153233991	LOR	loricrin	NM_000427	0	CTCTGGCGGCGG	nonframeshift insertion	566_567insCTCTGGCGGCGG	Y189delinsYSGGG	M1
2	120194651	120194651	TMEM37	transmembrane protein 37	NM_183240	0	GTGTGC	nonframeshift insertion	208_209insGTGTGC	T70delinsSVP	M1
X	2835999	2836007	ARSD	arylsulfatase D	NM_001669	CCACGCCC	0	nonframeshift deletion	701_709del	234_237del	M1
13	25671311	25671315	PABPC3	poly(A) binding protein, cytoplasmic 3	NM_030979	TATGA	0	frameshift deletion	975_979del	V325fs	M1
7	131241029	131241029	PODXL	podocalyxin-like	NM_001018111	0	GGCGAC	nonframeshift insertion	89_90insGTCGCC	P30delinsPSP	M1
3	46414944	46414975	CCR5	chemokine (C-C motif) receptor 5 (gene	NM_000579	ACAGTCAG	0	frameshift deletion	551_582del	Y184fs	M1
17	39305775	39305775	KRTAP4-5	keratin associated protein 4-5	M_033188	0	GGCAGCAGCTGGGG	nonframeshift insertion	244_245insGCCCCAGCTGCTGCC	Q82delinsRPSCCQ	M1
11	7818383	7818383	OR5P2	olfactory receptor, family 5, subfamily P,	NM_153444	0	ATATGGTTACCAGGT	nonframeshift insertion	106_107insGCATCTACCTGGTAACCATAT	S36delinsCIYLVTIS	M1
5	141324955	141324955	PCDH12	protocadherin 12	NM_016580	0	CTGCTGCTG	nonframeshift insertion	3545_3546insCAGCAGCAG	R1182delinsSSSR	M1
17	17697094	17697096	RAI1	retinoic acid induced 1	NM_030665	CAG	0	nonframeshift deletion	832_834del	278_278del	M1
14	92537354	92537354	ATXN3	Ataxin 3	NM_004993	0	CTGCTGCTGCTGCT	nonframeshift insertion	915_916insCAGCAGCAGCAGCAGCAG	G306delinsQQQQQQQQG	M11
18	24126808	24126808	KCTD1	Potassium channel tetramerization dom	NM_001142730	C	0	frameshift deletion	1693delG	A565fs	M11
4	57180576	57180576	KIAA1211	KIAA1211	NM_020722:exon8	0	GGAGCGGAGGGAG	nonframeshift insertion	908_909insGGAGCGGAGGGAGCGGAG	A303delinsAERRERR	M11
9	12775861	12775861	LURAP1L	Leucine rich adaptor protein 1-like	NM_203403	0	GGCGGCGGC	nonframeshift insertion	147_148insGGCGGCGGC	G49delinsGGGG	M11
15	90320121	90320144	MESP2	Mesoderm posterior basic helix-loop-he	NM_001039958	AGGGGCAC	0	nonframeshift deletion	533_556del	178_186del	M11
1	209605637	209605648	MIR205HG	MIR205 host gene (non-protein coding)		AGCAGCAG	0	nonframeshift deletion	252_263del	84_88del	M11
20	238436	238441	DEFB132	defensin, beta 132	NM_207469	TGGTCT	0	nonframeshift deletion	17_22del	6_8del	M11
1	153233991	153233991	LOR	loricrin	NM_000427	0	CTCTGGCGGCGG	nonframeshift insertion	566_567insCTCTGGCGGCGG	Y189delinsYSGGG	M11
19	55790886	55790886	HSPBP1	HSPA (heat shock 70kDa) binding prote	NM_001297600	0	GCCGCCGCC	nonframeshift insertion	228_229insGGCGGCGGC	S77delinsGGGS	M11
1	85039999	85040007	CTBS	chitobiase, di-N-acetyl-	NM_004388	GCAGCGCC	0	nonframeshift deletion	92_100del	31_34del	M11
8	86126827	86126827	C8orf59	chromosome 8 open reading frame 59	NM_001293320	0	AACATT	nonframeshift insertion	147_148insAATGTT	D50delinsNVD	M11
11	6567895	6567895	DNHD1	dynein heavy chain domain 1	NM_144666	0	TGCCCTACTGCA	nonframeshift insertion	5726_5727insTGCCCTACTGCA	A1909delinsAALLH	M11
7	131241030	131241035	PODXL	podocalyxin-like	NM_001018111	GGCGAC	0	nonframeshift deletion	84_89del	28_30del	M11
5	141324955	141324955	PCDH12	protocadherin 12	NM_016580	0	CTGCTGCTG	nonframeshift insertion	3545_3546insCAGCAGCAG	R1182delinsSSSR	M11
8	8234868	8234868	SGK223	homolog of rat pragma of Rnd2	NM_001080826	0	GCCGCT	nonframeshift insertion	1050_1051insAGCGGC	A351delinsSGA	M11
15	79058941	79058943	ADAMTS7	ADAM metallopeptidase with thrombosp	NM_014272	CGC	0	nonframeshift deletion	3310_3312del	1104_1104del	M14
9	100616701	100616706	FOXE1	Forkhead box E1	:NM_004473	GCCGCC	0	nonframeshift deletion	505_510del	169_170del	M14
7	5352665	5352665	TNRC18	Trinucleotide repeat containing 18	NM_001080495	0	GAGGAG	nonframeshift insertion	7856_7857insCTCCTC	S2619delinsSSS	M14
1	26608843	26608843	UBXN11	UBX domain protein 11	NM_145345	0	GGGACA	nonframeshift insertion	1410_1411insTGTCCC	:G471delinsCPG	M14
16	28507398	28507424	APOBR	Apolipoprotein B receptor	NM_018690	GGGACAGC	0	nonframeshift deletion	1036_1062del	346_354del	M16
6	82461728	82461742	FAM46A	Family with sequence similarity 46, mer	NM_017633	CCGCCGAA	0	nonframeshift deletion	117_131del	39_44del	M16
11	1651586	1651615	KRTAP5-5	Keratin associated protein 5-5	NM_001001480	CTGCTGCC	0	nonframeshift deletion	516_545del	172_182del	M16
11	76895771	76895792	MYO7A	Myosin VIIA	NM_001127179	GGAGGCGC	0	frameshift deletion	3514_3535del	G1172fs	M16
16	855518	855523	PRR25	Proline rich 25	NM_001013638	GCAGCT	0	nonframeshift deletion	76_81del	26_27del	M16
8	87226635	87226642	SLC7A13	Solute carrier family 7 (anionic amino ac	NM_138817	CCGACATC	0	frameshift deletion	1413_1511del	*471fs	M16
2	231861033	231861059	SPATA3	Spermatogenesis associated 3	NM_139073	CAGCAGCC	0	nonframeshift deletion	85_111del	29_37del	M16
18	56204388	56204402	ALPK2	alpha-kinase 2	NM_052947	CAGTTGAT	0	nonframeshift deletion	3017_3031del	1006_1011del	M16
8	11666219	11666224	FDFT1	Farnesyl-Diphosphate Farnesyltransfera	NM_001287750	TCCCAC	0	nonframeshift deletion	193_198del	65_66del	M16
10	46999591	46999591	GPRIN2	G protein regulated inducer of neurite o	NM_014696	0	ATGAGGGAG	nonframeshift insertion	711_712insATGAGGGAG	G237delinsGMRE	M16
12	53069223	53069243	KRT1	keratin 1	NM_006121	ACCTCCGG	0	nonframeshift deletion	1669_1689del	557_563del	M16
11	95825375	95825383	MAML2	Mastermind-Like 2	NM_032427	TGCTGCTG	0	nonframeshift deletion	1812_1820del	604_607del	M16
11	48346661	48346669	OR4C3	olfactory receptor, family 4, subfamily C	NM_001004702	TTGCTGAT	0	nonframeshift deletion	169_177del	57_59del	M16
20	238436	238441	DEFB132	defensin, beta 132	NM_207469	TGGTCT	0	nonframeshift deletion	17_22del	6_8del	M16
1	153907279	153907287	DENND4B	DENN/MADD Domain Containning 4B	NM_014856	CTGCTGCT	0	nonframeshift deletion	2722_2730del	908_910del	M16
16	71956512	71956517	IST1	increased sodium tolerance 1 homolog	NM_001270979	ATGCCC	0	nonframeshift deletion	244_249del	82_83del	M16
13	25671273	25671273	PABPC3	poly(A) binding protein, cytoplasmic 3	NM_030979	G	0	frameshift deletion	937delG	A313fs	M16
3	197880131	197880139	FAM157A	family with sequence similarity 157, mer	NM_001145248	GCAGCAGC	0	nonframeshift deletion	210_218del	70_73del	M16
1	153233991	153233991	LOR	loricrin	NM_000427	0	CTCTGGCGGCGG	nonframeshift insertion	566_567insCTCTGGCGGCGG	Y189delinsYSGGG	M16

2	120194651	120194651	TMEM37	transmembrane protein 37	NM_183240	0	GTGTGC	nonframeshift insertion	208_209insGTGTGC	T70delinsSVP	M16
5	176026120	176026143	GPRIN1	G protein regulated inducer of neurite o	NM_052899	CTCAAAGA	0	nonframeshift deletion	693_716del	231_239del	M16
7	100612955	100612955	MUC12	Mucin12	NM_001164462	0	CTG	nonframeshift insertion	52_53insCTG	T18delinsTA	M16
13	25671311	25671315	PABPC3	poly(A) binding protein, cytoplasmic 3	NM_030979	TATGA	0	frameshift deletion	975_979del	V325fs	M16
13	25670801	25670801	PABPC3	poly(A) binding protein, cytoplasmic 3	NM_030979	0	G	frameshift insertion	465_466insG	I155fs	M16
13	25671146	25671157	PABPC3	poly(A) binding protein, cytoplasmic 3	NM_030979	GGAACGGC	0	nonframeshift deletion	810_821del	270_274del	M16
3	52027853	52027853	RPL29	ribosomal protein L29	NM_000992	0	CCTTGG	nonframeshift insertion	391_392insCCAAGG	D131delinsAKD	M16
8	8234868	8234868	SGK223	homolog of rat pragma of Rnd2	NM_001080826	0	GCCGCT	nonframeshift insertion	1050_1051insAGCGGC	A351delinsSGA	M16
21	34003928	34003928	SYNJ1	synaptojanin 1	NM_003895	0	AGTATT	nonframeshift insertion	4215_4216insAATACT	L1406delinsNTL	M16
14	23548783	23548783	ACIN1	apoptotic chromatin condensation induc	NM_014977	0	GAACGT	nonframeshift insertion	1934_1935insACGTTC	S645delinsSRS	M17
14	74060511	74060511	ACOT4	acyl-CoA thioesterase 4	NM_152331	0	TCAA	frameshift insertion	563_564insTCAA	L188fs	M17
14	74060514	74060517	ACOT4	acyl-CoA thioesterase 4	NM_152331	CTTA	0	frameshift deletion	566_569del	A189fs	M17
2	73613032	73613037	ALMS1	Alstrom syndrome 1	NM_015120	GGAGGA	0	nonframeshift deletion	36_41del	12_14del	M17
14	105055119	105055127	C14orf180	chromosome 14 open reading frame 18	NM_001008404	GACGGGC	0	frameshift deletion	482_819del	*161fs	M17
16	85743839	85743858	C16orf74	Chromosome 16 open reading frame 74	NM_206967	CGTCCAGC	0	frameshift deletion	84_103del	L28fs	M17
6	90577707	90577721	CASP8AP2	caspase 8 associated protein 2	NM_001137667	GACATCTT	0	nonframeshift deletion	4698_4712del	1566_1571del	M17
6	90577726	90577728	CASP8AP2	caspase 8 associated protein 2	NM_001137667	GGA	0	nonframeshift deletion	4717_4719del	1573_1573del	M17
22	50315936	50315973	CRELD2	Cysteine-rich with EGF-like domains 2	NM_002430	CCTCAGCA	0	frameshift deletion	1632_1637del	544_546del	M17
1	225707034	225707051	ENAH	enabled homolog (Drosophila)	NM_001008493	TCCAGGCC	0	nonframeshift deletion	651_668del	217_223del	M17
17	77710954	77710954	ENPP7	Ectonucleotide pyrophosphatase/phosp	NM_178543	C	0	frameshift deletion	1141delC	P381fs	M17
4	3076604	3076621	HTT	Huntingtin	NM_002111	CAGCAGCA	0	nonframeshift deletion	52_69del	18_23del	M17
15	100252710	100252715	MEF2A	myocyte enhancer factor 2A	NM_005587	CAGCAG	0	nonframeshift deletion	1234_1239del	412_413del	M17
22	28194895	28194900	MN1	Meningioma (disrupted in balanced tran	NM_002430	TGCTGT	0	nonframeshift deletion	1632_1637del	544_546del	M17
6	45390487	45390504	RUNX2	runt-related transcription factor 2	NM_001024630	GGCGGCGC	0	nonframeshift deletion	216_233del	72_78del	M17
10	21805466	21805466	SKIDA1	SKI/DACH domain containing 1	NM_207371	0	CCTCCT	nonframeshift insertion	1285_1286insAGGAGG	G429delinsEEG	M17
19	48305639	48305650	TPRX1	Tetra-peptide repeat homeobox 1	NM_198479	GGGCCTGC	0	nonframeshift deletion	618_629del	206_210del	M17
9	124855330	124855330	TTLL11	Tubulin tyrosine ligase-like family memb	NM_00113944	0	TGGCCT	nonframeshift insertion	367_368insAGGCCA	T123delinsKAT	M17
17	46882287	46882303	TTLL6	Tubulin tyrosine ligase-like family memb	NM_001130918	CTTTCCAA	0	frameshift deletion	154_170del:p	C52fs	M17
7	30325341	30325341	ZNRF2	Zinc and ring finger 2, E3 ubiquitin prote	NM_147128	0	CGG	nonframeshift insertion	368_369insCGG	D123delinsDG	M17
18	56204388	56204402	ALPK2	alpha-kinase 2	NM_052947	CAGTTGAT	0	nonframeshift deletion	3017_3031del	1006_1011del	M17
10	46999591	46999591	GPRIN2	G protein regulated inducer of neurite o	NM_014696	0	ATGAGGGAG	nonframeshift insertion	711_712insATGAGGGAG	G237delinsGMRE	M17
12	53069223	53069243	KRT1	keratin 1	NM_006121	ACCTCCGC	0	nonframeshift deletion	1669_1689del	557_563del	M17
1	209605637	209605648	MIR205HG	MIR205 host gene (non-protein coding)		AGCAGCAC	0	nonframeshift deletion	252_263del	84_88del	M17
11	48346661	48346669	OR4C3	olfactory receptor, family 4, subfamily C	NM_001004702	TTGCTGAT	0	nonframeshift deletion	169_177del	57_59del	M17
5	140574170	140574175	PCDHB10	protocadherin beta 10	NM_018930	AGGCCG	0	nonframeshift deletion	2045_2050del	682_684del	M17
20	238436	238441	DEFB132	defensin, beta 132	NM_207469	TGGTCT	0	nonframeshift deletion	17_22del	6_8del	M17
3	197880131	197880139	FAM157A	family with sequence similarity 157, me	NM_001145248	GCAGCAGC	0	nonframeshift deletion	210_218del	70_73del	M17
5	176026120	176026143	GPRIN1	G protein regulated inducer of neurite o	NM_052899	CTCAAAGA	0	nonframeshift deletion	693_716del	231_239del	M17
7	100612955	100612955	MUC12	Mucin12	NM_001164462	0	CTG	nonframeshift insertion	52_53insCTG	T18delinsTA	M17
12	122359397	122359397	WDR66	WD Repeat Domain 66	NM_144668	0	GAGGAGGAGGAGAA	nonframeshift insertion	186_187insGAGGAGGAGGAGAAA	G62delinsGEEEEK	M17
8	65494007	65494007	BHLHE22	basic helix-loop-helix family, member e	NM_152414	0	AGCGGC	nonframeshift insertion	660_661insAGCGGC	G220delinsGSG	M17
3	46414944	46414975	CCR5	chemokine (C-C motif) receptor 5 (gene	NM_000579	ACAGTCAG	0	frameshift deletion	551_582del	Y184fs	M17
9	12775861	12775861	LURAP1L	leucine rich adaptor protein 1-like	NM_203403	0	GGTGGC	nonframeshift insertion	147_148insGGCGGCGGC	G49delinsGGGG	M17
7	131241030	131241035	PODXL	podocalyxin-like	NM_001018111	GGCGAC	0	nonframeshift deletion	84_89del	28_30del	M17
3	52027853	52027853	RPL29	ribosomal protein L29	NM_000992	0	CCTTGG	nonframeshift insertion	391_392insCCAAGG	D131delinsAKD	M17
12	118506328	118506333	VSIG10	V-set and immunoglobulin domain cont	NM_019086	TCCTCC	0	nonframeshift deletion	1416_1421del	472_474del	M17
22	50921149	50921166	ADM2	adrenomedullin 2	NM_001253845	ACACTCGG	0	nonframeshift deletion	264_281del	88_94del	M2
11	129991653	129991658	APLP2	Amyloid beta (A4) precursor-like protein	NM_001642	GAAGAG	0	nonframeshift deletion	661_666del	221_222del	M2
19	13054656	13054658	CALR	Calreticulin	NM_004343	GAG	0	nonframeshift deletion	1183_1185del	395_395del	M2
17	72889649	72889649	FADS6	Fatty acid desaturase 6	NM_178128	0	GGTTCCATGGGCTC	nonframeshift insertion	44_45insTACGGAGCCCATGGAACC	P15delinsPTEPMEP	M2
2	187559029	187559029	FAM171B	Family with sequence similarity 171, me	NM_177454	0	CAGCAG	nonframeshift insertion	129_130insCAGCAG	Q43delinsQQQ	M2
17	39383323	39383334	KRTAP9-2	Kerain Associated Protein 9-2	NM_031961	CTGCTGCC	0	nonframeshift deletion	417_428del	139_143del	M2
11	47640434	47640434	MTCH2	Mitochondrial carrier 2	NM_014342	0	TGTC	frameshift insertion	862_863insGACA	K288fs	M2
1	111957502	111957525	OVGP1	Oviductal glycoprotein 1	NM_002557	TCACAGAC	0	nonframeshift deletion	1598_1621del	533_541del	M2
17	17697094	17697105	RAI1	retinoic acid induced 1	NM_030665	CAGCAGCA	0	nonframeshift deletion	832_843del	278_281del	M2
21	34948684	34948684	SON	SON DNA binding protein	NM_138927	0	A	frameshift insertion	7236dupA	G2412fs	M2
4	177106010	177106013	SPATA4	Spermatogenesis associated 4	NM_144644	TCTC	0	frameshift deletion	836_839del	R279fs	M2
14	58863049	58863054	TOMM20L	Translocase of outer mitochondrial men	NM_207377	AGGGCA	0	nonframeshift deletion	170_175del	57_59del	M2
1	26608812	26608835	UBXN11	UBX domain protein 11	NM_145345	CCAGGACA	0	nonframeshift deletion	1419_1442del	473_481del	M2
15	85186877	85186894	WDR73	WD repeat domain 73	NM_032856	CTTGGCTC	0	nonframeshift deletion	944_961del	315_321del	M2
18	56204388	56204402	ALPK2	alpha-kinase 2	NM_052947	CAGTTGAT	0	nonframeshift deletion	3017_3031del	1006_1011del	M2
8	11666219	11666224	FDFT1	Farnesyl-Diphosphate Farnesyltransfera	NM_001287750	TCCCAC	0	nonframeshift deletion	193_198del	65_66del	M2
10	46999591	46999591	GPRIN2	G protein regulated inducer of neurite o	NM_014696	0	ATGAGGGAG	nonframeshift insertion	711_712insATGAGGGAG	G237delinsGMRE	M2
12	53069223	53069243	KRT1	keratin 1	NM_006121	ACCTCCGC	0	nonframeshift deletion	1669_1689del	557_563del	M2
11	95825375	95825383	MAML2	Mastermind-Like 2	NM_032427	TGCTGCTG	0	nonframeshift deletion	1812_1820del	604_607del	M2
11	48346661	48346669	OR4C3	olfactory receptor, family 4, subfamily C	NM_001004702	TTGCTGAT	0	nonframeshift deletion	169_177del	57_59del	M2
5	140574170	140574175	PCDHB10	protocadherin beta 10	NM_018930	AGGCCG	0	nonframeshift deletion	2045_2050del	682_684del	M2

1	153907279	153907287	DENND4B	DENN/MADD Domain Containning 4B	NM_014856	CTGCTGCT	0	nonframeshift deletion	2722_2730del	908_910del	M2
16	71956512	71956517	IST1	increased sodium tolerance 1 homolog	NM_001270979	ATGCCC	0	nonframeshift deletion	244_249del	82_83del	M2
3	197880131	197880139	FAM157A	family with sequence similarity 157, mer	NM_001145248	GCAGCAGC	0	nonframeshift deletion	210_218del	70_73del	M2
3	10088407	10088410	FANCD2	Fanconi anemia, complementation grou	NM_033084	AGTA	0	frameshift deletion	1278_1278del	L426fs	M2
5	176026120	176026143	GPRIN1	G protein regulated inducer of neurite o	NM_052899	CTCAAAGA	0	nonframeshift deletion	693_716del	231_239del	M2
7	100612955	100612955	MUC12	Mucin12	NM_001164462	0	CTG	nonframeshift insertion	52_53insCTG	T18delinsTA	M2
12	122359397	122359397	WDR66	WD Repeat Domain 66	NM_144668	0	GAGGAGGAGGAGAA	nonframeshift insertion	186_187insGAGGAGGAGGAGAAA	G62delinsGEEEEK	M2
1	85039999	85040007	CTBS	chitobiase, di-N-acetyl-	NM_004388	GCAGCGCC	0	nonframeshift deletion	92_100del	31_34del	M2
8	86126827	86126827	C8orf59	chromosome 8 open reading frame 59	NM_001293320	0	AACATT	nonframeshift insertion	147_148insAATGTT	D50delinsNVD	M2
8	103573011	103573037	ODF1	outer dense fiber of sperm tails 1	NM_024410	TGCAACCC	0	nonframeshift deletion	652_678del	218_226del	M2
2	113300002	113300026	POLR1B	polymerase (RNA) I polypeptide B, 128	NM_001282772	TCCGGCGT	0	frameshift deletion	45_69del	L15fs	M2
12	118506328	118506333	VSIG10	V-set and immunoglobulin domain cont	NM_019086	TCCTCC	0	nonframeshift deletion	1416_1421del	472_474del	M2
19	47883158	47883163	DHX34	DEAH (Asp-Glu-Ala-His) box polypeptic	NM_014681	GGAGGA	0	nonframeshift deletion	2898_2903del	966_968del	M4
2	71801334	71801334	DYSF	Dysferlin	NM_001130977	0	AGGCGG	nonframeshift insertion	3139_3140insAGGCGG	Q1047delinsQAE	M4
15	40268998	40268998	EIF2AK4	Eukaryotic translation initiation factor 2	NM_001013703	0	GACGAC	nonframeshift insertion	2202_2203insGACGAC	E734delinsEDD	M4
13	46170720	46170737	ERICH6B	Glutamate-rich 6B	NM_182542	CCAGATAC	0	nonframeshift deletion	404_421del	135_141del	M4
7	150864247	150864261	GBX1	Gastrulation brain homeobox 1	NM_001098834	GCGGCGG	0	nonframeshift deletion	375_389del	125_130del	M4
1	9304979	9304986	H6PD	Hexose-6-phosphate dehydrogenase (g	NM_001282587	CCCAGGCA	0	nonframeshift deletion	24_26del	8_9del	M4
15	28518115	28518115	HERC2	HECT and RLD domain containing E3 u	NM_004667	C	0	frameshift deletion	836delG	G279fs	M4
17	46608184	46608184	HOXB1	Homeobox B1	NM_002144	0	GGGCGCTGT	nonframeshift insertion	82_83insACAGCGCCC	P28delinsHSAP	M4
X	78216054	78216054	P2RY10	purinergic receptor P2Y, G-protein coup	NM_014499	A	0	frameshift deletion	37delA	M13fs	M4
6	160560898	160560905	SLC22A1	Solute carrier family 22	NM_003057	TGGTAAGT	0	frameshift deletion	1275_1276del	P425fs	M4
X	118603706	118603706	SLC25A5	Solute carrier family 25 (mitochondrial c	NM_001152	0	G	frameshift insertion	195dupG	Q65fs	M4
17	76798549	76798554	USP36	Ubiquitin specific peptidase 36	NM_025090	TTTTTC	0	nonframeshift deletion	2874_2879del	958_960del	M4
18	56204388	56204402	ALPK2	alpha-kinase 2	NM_052947	CAGTTGAT	0	nonframeshift deletion	3017_3031del	1006_1011del	M4
1	209605637	209605648	MIR205HG	MIR205 host gene (non-protein coding)		AGCAGCAC	0	nonframeshift deletion	252_263del	84_88del	M4
5	140574170	140574175	PCDHB10	protocadherin beta 10	NM_018930	AGGCCG	0	nonframeshift deletion	2045_2050del	682_684del	M4
16	71956512	71956517	IST1	increased sodium tolerance 1 homolog	NM_001270979	ATGCCC	0	nonframeshift deletion	244_249del	82_83del	M4
13	25671273	25671273	PABPC3	poly(A) binding protein, cytoplasmic 3	NM_030979	G	0	frameshift deletion	937delG	A313fs	M4
1	153233991	153233991	LOR	loricrin	NM_000427	0	CTCTGGCGGCGG	nonframeshift insertion	566_567insCTCTGGCGGCGG	Y189delinsYSGGG	M4
2	120194651	120194651	TMEM37	transmembrane protein 37	NM_183240	0	GTGTGC	nonframeshift insertion	208_209insGTGTGC	T70delinsSVP	M4
X	2835999	2836007	ARSD	arylsulfatase D	NM_001669	CCACGCCC	0	nonframeshift deletion	701_709del	234_237del	M4
3	10088407	10088410	FANCD2	Fanconi anemia, complementation grou	NM_033084	AGTA	0	frameshift deletion	1278_1278del	L426fs	M4
19	55790886	55790886	HSPBP1	HSPA (heat shock 70kDa) binding prote	NM_001297600	0	GCCGCCGCC	nonframeshift insertion	228_229insGGCGGCGGC	S77delinsGGGS	M4
13	25671311	25671315	PABPC3	poly(A) binding protein, cytoplasmic 3	NM_030979	TATGA	0	frameshift deletion	975_979del	V325fs	M4
7	131241029	131241029	PODXL	podocalyxin-like	NM_001018111	0	GGCGAC	nonframeshift insertion	89_90insGTCGCC	P30delinsPSP	M4
8	103573011	103573037	ODF1	outer dense fiber of sperm tails 1	NM_024410	TGCAACCC	0	nonframeshift deletion	652_678del	218_226del	M4
13	25670801	25670801	PABPC3	poly(A) binding protein, cytoplasmic 3	NM_030979	0	G	frameshift insertion	465_466insG	I155fs	M4
13	25671146	25671157	PABPC3	poly(A) binding protein, cytoplasmic 3	NM_030979	GGAACGGC	0	nonframeshift deletion	810_821del	270_274del	M4
17	17697094	17697096	RAI1	retinoic acid induced 1	NM_030665	CAG	0	nonframeshift deletion	832_834del	278_278del	M4
19	17397498	17397501	ANKLE1	nkyrin repeat and LEM domain containi	NM_001278444	TGTT	0	frameshift deletion	1930_1933del	C644fs	M6
4	88536863	88536880	DSPP	Dentin sialophosphoprotein	NM_014208	AATAGTAGT	0	nonframeshift deletion	3049_3066del	1017_1022del	M6
22	38482353	38482394	BAIAP2L2	BAI1-associated protein 2-like 2	NM_025045	TGCGGGAC	0	nonframeshift deletion	1322_1363del	441_455del	M8
9	70900911	70900911	CBWD3	COBW domain containing 3	NM_201453	0	A	frameshift insertion	772dupA	Q257fs	M8
17	77807917	77807917	CBX4	chromobox homolog 4	NM_003655	0	GCCGCC	nonframeshift insertion	1523_1524insGGCGGC	A508delinsAAA	M8
1	240370914	240370946	FMN2	Formin 2	NM_020066	GCCCCCTC	0	nonframeshift deletion	2802_2834del	934_945del	M8
6	42179532	42179543	MRPS10	Mitochondrial ribosomal protein S10	NM_018141	TACCAAGT	0	nonframeshift deletion	299_310del	100_104del	M8
1	248722428	248722428	OR2T29	Olfactory receptor, family 2, subfamily T	NM_001004694	0	CG	frameshift insertion	364_365insCG	M122fs	M8
11	608503	608514	PHRF1	PHD and ring finger domains 1	NM_001286581	GGACGCG	0	nonframeshift deletion	3047_3058del	1016_1020del	M8
19	11558341	11558346	PRKCSH	Protein kinase C substrate 80K-H	NM_001001329	GAGGAG	0	nonframeshift deletion	937_942del	313_314del	M8
9	79318376	79318390	PRUNE2	Prune homolog 2 (Drosophila)	NM_015225	GTGACAGC	0	nonframeshift deletion	8139_8153del	2713_2718del	M8
X	118603962	118603962	SLC25A5	Solute carrier family 25 (mitochondrial c	NM_001152	T	0	frameshift deletion	450delT	A150fs	M8
19	7585338	7585364	ZNF358	Zinc finger protein 358	NM_018083	GCAGCTGC	0	nonframeshift deletion	1210_1236del	404_412del	M8
8	11666219	11666224	FDFT1	Farnesyl-Diphosphate Farnesyltransfera	NM_001287750	TCCCAC	0	nonframeshift deletion	193_198del	65_66del	M8
10	46999591	46999591	GPRIN2	G protein regulated inducer of neurite o	NM_014696	0	ATGAGGGAG	nonframeshift insertion	711_712insATGAGGGAG	G237delinsGMRE	M8
12	53069223	53069243	KRT1	keratin 1	NM_006121	ACCTCCGG	0	nonframeshift deletion	1669_1689del	557_563del	M8
11	95825375	95825383	MAML2	Mastermind-Like 2	NM_032427	TGCTGCTG	0	nonframeshift deletion	1812_1820del	604_607del	M8
11	48346661	48346669	OR4C3	olfactory receptor, family 4, subfamily C	NM_001004702	TTGCTGAT	0	nonframeshift deletion	169_177del	57_59del	M8
5	140574170	140574175	PCDHB10	protocadherin beta 10	NM_018930	AGGCCG	0	nonframeshift deletion	2045_2050del	682_684del	M8
1	153907279	153907287	DENND4B	DENN/MADD Domain Containning 4B	NM_014856	CTGCTGCT	0	nonframeshift deletion	2722_2730del	908_910del	M8
13	25671273	25671273	PABPC3	poly(A) binding protein, cytoplasmic 3	NM_030979	G	0	frameshift deletion	937delG	A313fs	M8
3	197880131	197880139	FAM157A	family with sequence similarity 157, mer	NM_001145248	GCAGCAGC	0	nonframeshift deletion	210_218del	70_73del	M8
2	120194651	120194651	TMEM37	transmembrane protein 37	NM_183240	0	GTGTGC	nonframeshift insertion	208_209insGTGTGC	T70delinsSVP	M8
X	2835999	2836007	ARSD	arylsulfatase D	NM_001669	CCACGCCC	0	nonframeshift deletion	701_709del	234_237del	M8
12	122359397	122359397	WDR66	WD Repeat Domain 66	NM_144668	0	GAGGAGGAGGAGAA	nonframeshift insertion	186_187insGAGGAGGAGGAGAAA	G62delinsGEEEEK	M8
8	65494007	65494007	BHLHE22	basic helix-loop-helix family, member e	NM_152414	0	AGCGGC	nonframeshift insertion	660_661insAGCGGC	G220delinsGSG	M8
2	233712210	233712230	GIGYF2	GRB10 interacting GYF protein 2	NM_001103147	CAGCAGCA	0	nonframeshift deletion	3676_3696del	1226_1232del	M8

17	39305775	39305775	KRTAP4-5	keratin associated protein 4-5	M_033188	0	GGCAGCAGCTGGGG	nonframeshift insertion	244_245insGCCCCAGCTGCTGCC	Q82delinsRPSCCQ	M8
9	12775861	12775861	LURAP1L	leucine rich adaptor protein 1-like	NM_203403	0	GGTGGC	nonframeshift insertion	147_148insGGCGGCGGC	G49delinsGGGG	M8
18	72223591	72223591	CNDP1	Carnosine dipeptidase 1	NM_032649	0	TGCTGC	nonframeshift insertion	43_44insTGCTGC	V15delinsVLL	M9
13	72440538	72440538	DACH1	dachshund family transcription factor 1	NM_080759	0	GCCGCC	nonframeshift insertion	369_370insGGCGGC	I124delinsGGI	M9
7	96635364	96635369	DLX6	Distal-less homeobox 6	NM_005222	GCAGCA	0	nonframeshift deletion	75_80del	25_27del	M9
10	100992131	100992131	HPSE2	heparanase 2	NM_021828	0	CCGGGC	nonframeshift insertion	421_422insGCCCGG	D141delinsGPD	M9
17	38975308	38975319	KRT10	Keratin 10, type I	NM_000421	GCCGCCGT	0	nonframeshift deletion	1468_1479del	490_493del	M9
1	52499071	52499097	KTI12	KTI12 homolog, chromatin associated (	NM_138417	GCCCGCCA	0	nonframeshift deletion	337_363del	113_121del	M9
7	20824941	20824943	SP8	Sp8 transcription factor	NM_182700	GCC	0	nonframeshift deletion	493_495del	165_165del	M9
22	26879947	26879967	SRRD	SRR1 domain containing	NM_001013694	GAGGCGG(	0	nonframeshift deletion	91_111del	31_37del	M9
13	113201853	113201867	TUBGCP3	Tubulin, gamma complex associated pr	NM_001286279	TGGGAAAG	0	nonframeshift deletion	1235_1249del	412_417del	M9
8	11666219	11666224	FDFT1	Farnesyl-Diphosphate Farnesyltransfera	NM_001287750	TCCCAC	0	nonframeshift deletion	193_198del	65_66del	M9
10	46999591	46999591	GPRIN2	G protein regulated inducer of neurite o	NM_014696	0	ATGAGGGAG	nonframeshift insertion	711_712insATGAGGGAG	G237delinsGMRE	M9
11	95825375	95825383	MAML2	Mastermind-Like 2	NM_032427	TGCTGCTG	0	nonframeshift deletion	1812_1820del	604_607del	M9
1	209605637	209605648	MIR205HG	MIR205 host gene (non-protein coding)		AGCAGCAG	0	nonframeshift deletion	252_263del	84_88del	M9
5	140574170	140574175	PCDHB10	protocadherin beta 10	NM_018930	AGGCCG	0	nonframeshift deletion	2045_2050del	682_684del	M9
1	153907279	153907287	DENND4B	DENN/MADD Domain Containning 4B	NM_014856	CTGCTGCT	0	nonframeshift deletion	2722_2730del	908_910del	M9
3	10088407	10088410	FANCD2	Fanconi anemia, complementation grou	NM_033084	AGTA	0	frameshift deletion	1278_1278del	L426fs	M9
19	55790886	55790886	HSPBP1	HSPA (heat shock 70kDa) binding prote	NM_001297600	0	GCCGCCGCC	nonframeshift insertion	228_229insGGCGGCGGC	S77delinsGGGS	M9
7	131241029	131241029	PODXL	podocalyxin-like	NM_001018111	0	GGCGAC	nonframeshift insertion	89_90insGTCGCC	P30delinsPSP	M9
11	6567895	6567895	DNHD1	dynein heavy chain domain 1	NM_144666	0	TGCCCTACTGCA	nonframeshift insertion	5726_5727insTGCCCTACTGCA	A1909delinsAALLH	M9
2	233712210	233712230	GIGYF2	GRB10 interacting GYF protein 2	NM_001103147	CAGCAGCA	0	nonframeshift deletion	3676_3696del	1226_1232del	M9
11	7818383	7818383	OR5P2	olfactory receptor, family 5, subfamily P,	NM_153444	0	ATATGGTTACCAGGT/	nonframeshift insertion	106_107insGCATCTACCTGGTAACCATAT	S36delinsCIYLVTIS	M9
2	113300002	113300026	POLR1B	polymerase (RNA) I polypeptide B, 128	NM_001282772	TCCGGCGT	0	frameshift deletion	45_69del	L15fs	M9
21	34003928	34003928	SYNJ1	synaptojanin 1	NM_003895	0	AGTATT	nonframeshift insertion	4215_4216insAATACT	L1406delinsNTL	M9

Supplementary Table S5

Splice variants

Chr	Start	End	Gene symbo	Gene dscription	Accession	Reference A	Variant Allel	Variant clas	cDNA change	Sample
17	41226539	41226539	BRCA1	breast cancer 1, early onset	NM_007300	C	A	splicing	4548-1G>T	M1
16	586144	586144	CAPN15	calpain 15	NM_005632	T	C	splicing		M1
6	70408934	70408934	LMBRD1	LMBR1 domain containing 1	NM_018368	C	T	splicing	1338+1G>A	M1
1	234556559	234556559	TARBP1	TAR (HIV-1) RNA binding protein 1	NM_005646	C	A	splicing	3445-1G>T	M1
12	43846499	43846499	ADAMTS20	ADAM metallopeptidase with thrombospondin type 1 motif, 20	NM_025003	C	T	splicing	1761-1G>A	M11
X	18616735	18616735	CDKL5	cyclin-dependent kinase-like 5	NM_001037343	T	G	splicing	977+2T>G	M11
4	103806372	103806372	CISD2	CDGSH iron sulfur domain 2	NM_001008388	G	T	splicing	104-1G>T	M11
19	15756528	15756528	CYP4F3	cytochrome P450, family 4, subfamily F, polypeptide 3	NM_000896	G	A	splicing	199-1G>A	M11
11	30253443	30253443	FSHB	follicle stimulating hormone, beta polypeptide	NM_000510	G	A	splicing		M11
17	39138716	39138716	KRT40	keratin 40	NM_182497	C	T	splicing	531-1G>A	M11
19	889637	889637	MED16	mediator complex subunit 16	NM_005481	C	T	splicing	447+1G>A	M11
17	16068476	16068476	NCOR1	nuclear receptor corepressor 1	NM_006311	C	T	splicing	436-1G>A	M11
7	151048509	151048509	NUB1	negative regulator of ubiquitin-like proteins 1	NM_001243351	G	A	splicing	358-1G>A	M11
8	43152603	43152603	POTEA	POTE ankyrin domain family, member A	NM_001005365	G	A	splicing	588+1G>A	M11
3	77651643	77651643	ROBO2	roundabout, axon guidance receptor, homolog 2 (Drosophila)	NM_001128929	G	A	splicing	3077+1G>A	M11
9	94808336	94808336	SPTLC1	serine palmitoyltransferase, long chain base subunit 1	NM_001281303	C	A	splicing	1082-1G	M11
8	108297077	108297077	ANGPT1	angiopoietin 1	NM_001146	C	T	splicing	1039-1G>A	M16
1	87029343	87029343	CLCA4	chloride channel accessory 4	NM_012128	G	A	splicing	449-1G>A	M16
5	1323985	1323985	CLPTM1L	CLPTM1-like	NM_030782	C	T	splicing	1198-1G>A	M16
17	74470811	74470811	RHBDF2	rhomboid 5 homolog 2 (Drosophila)	NM_024599	C	T	splicing	1389+1G>A	M16
15	51988071	51988071	SCG3	secretogranin III	NM_013243	G	A	splicing	869-1G>A	M16
6	108243115	108243115	SEC63	SEC63 homolog (S. cerevisiae)	NM_007214	0	GGGG	splicing	340-2->CCCC	M16
12	7080212	7080212	EMG1	EMG1 N1-specific pseudouridine methyltransferase	NM_006331	0	C	splicing	125+1->C	M16
3	49723167	49723167	MST1	macrophage stimulating 1 (hepatocyte growth factor-like)	NM_020998	T	G	splicing	1251-2A>C	M16
4	76836020	76836020	NAAA	N-acylethanolamine acid amidase	NM_014435	C	A	splicing	1116+1G>T	M17
2	27428217	27428217	SLC5A6	solute carrier family 5 (sodium/multivitamin and iodide cotranspor	NM_021095	C	A	splicing	734+1G>T	M17
17	73626918	73626918	RECQL5	RecQ protein-like 5	NM_004259	0	TG	splicing	1586-1->CA	M17
2	68274452	68274452	C1D	C1D nuclear receptor corepressor		C	A	splicing		M2
4	190874281	190874281	FRG1	FSHD region gene 1	NM_004477	G	T	splicing	317+1G>T	M2
15	44063302	44063302	PDIA3	protein disulfide isomerase family A, member 3	NM_005313	G	T	splicing	1405-1G>T	M2
10	29779774	29779774	SVIL	supervillin	NM_021738	A	T	splicing	4192+2T	M2
7	73254452	73254453	WBSCR27	Williams Beuren syndrome chromosome region 27	NM_152559	TG	0	splicing		M2
12	7080212	7080212	EMG1	EMG1 N1-specific pseudouridine methyltransferase	NM_006331	0	C	splicing	125+1->C	M2
17	73626918	73626918	RECQL5	RecQ protein-like 5	NM_004259	0	TG	splicing	1586-1->CA	M2
7	93116320	93116320	CALCR	calcitonin receptor	NM_001164737	C	T	splicing	29-1G>A	M4
1	19670851	19670851	CAPZB	capping protein (actin filament) muscle Z-line, beta	NM_001282162	C	T	splicing	818+1G>A	M4
8	76472578	76472578	HNF4G	hepatocyte nuclear factor 4, gamma	NM_004133	G	A	splicing	1094-1G>A	M4
11	68321726	68321726	PPP6R3	protein phosphatase 6, regulatory subunit 3	NM_001164160	G	A	splicing	731+1G>A	M4
3	50220998	50220998	SEMA3F	sema domain, immunoglobulin domain (Ig), short basic domain, s	NM_004186	G	A	splicing	1233+1G>A	M4
5	35740230	35740230	SPEF2	sperm flagellar 2	NM_024867	G	A	splicing	3192-1G>A	M4
12	7080212	7080212	EMG1	EMG1 N1-specific pseudouridine methyltransferase	NM_006331	0	C	splicing	125+1->C	M4
17	73626918	73626918	RECQL5	RecQ protein-like 5	NM_004259	0	TG	splicing	1586-1->CA	M4
5	153065890	153065890	GRIA1	glutamate receptor, ionotropic, AMPA 1	NM_000827	G	T	splicing	1134+1G	M8
12	7080212	7080212	EMG1	EMG1 N1-specific pseudouridine methyltransferase	NM_006331	0	C	splicing	125+1->C	M8
17	19645526	19645526	ALDH3A1	aldehyde dehydrogenase 3 family, member A1	NM_001135168	C	T	splicing	481-1G>A	M9
11	9191498	9191498	DENND5A	DENN/MADD domain containing 5A	NM_015213	T	C	splicing	2058-2A>G	M9
5	90059121	90059121	GPR98	G protein-coupled receptor 98	NM_032119	G	A	splicing	12121-1G>A	M9
8	142486249	142486249	MROH5	maestro heat-like repeat family member 5	NM_207414	T	G	splicing	1446-2A>C	M9
5	101774466	101774466	SLCO6A1	solute carrier organic anion transporter family, member 6A1	NM_001289002	C	T	splicing	1132-1G>A	M9
22	37465386	37465390	TMPRSS6	transmembrane protease, serine 6	NM_001289000	TGGGG	0	splicing		M9
3	49723167	49723167	MST1	macrophage stimulating 1 (hepatocyte growth factor-like)	NM_020998	T	G	splicing	1251-2A>C	M9

Supplementary Table S6				
**SCNA gain**				
**chrom**	**start**	**end**	**samples**	**genes**
chr1	14362	14595	M1,M16,M17,M9	DDX11L1, WASH7P
chr1	14595	17742	M1,M14,M16,M17,M9	WASH7P
chr1	17742	853100	M1,M14,M17	OR4F5, OR4F29, OR4F16, WASH7P, MIR1302-11, MIR1302-10, MIR1302-9, MIR1302-2, FAM138A, OR4G4P, OR4G11P, CICP27, RNU6-1100P, CICP7, WBP1LP7, MTND1P23, MTND2P28, MTATP8P1, MTATP6P1, WBP1LP6, CICP3, RNU6-1199P, FAM87B, LINC00115, LINC01128, FAM41C, TUBB8P11
chr1	853100	854817	M14,M17	
chr1	854817	861150	M14	SAMD11
chr1	33547048	33794470	M14	ADC, TRIM62, ZNF362, A3GALT2, PHC2
chr1	33794470	35220796	M14,M16	MIR3605, RN7SKP16, TLR12P, HSPD1P14, RNA5SP42, MIR552, PHC2, ZSCAN20, CSMD2, HMGB4, C1orf94, SMIM12, GJB5
chr1	35220796	35247976	M14	SMIM12, GJB5, GJB4, GJB3
chr1	45251822	45278875	M6	BEST4, PLK3, TCTEX1D4, BTBD19
chr1	55545210	61892228	M16	GYG1P3, MIR4422, GOT2P1, RN7SKP291, RNU6-830P, PIGQP1, RPL21P23, RPS20P5, DAB1-AS1, HNRNPA1P6, RPS26P15, RN7SL713P, LINC01135, PHBP3, MIR4711, RN7SL475P, PGBD4P8, USP24, PPAP2B, PRKAA2, C1orf168, C8A, C8B, DAB1, OMA1, TACSTD2, MYSM1, JUN, FGGY, HOOK1, CYP2J2, C1orf87, NFIA
chr1	86211123	86249941	M17	COL24A1
chr1	86249941	86836836	M1,M17	RNA5SP51, COL24A1, ODF2L
chr1	86836836	86852769	M17	ODF2L
chr1	100920960	102271631	M11	HNRNPA1P68, PPIAP7, RNU6-965P, CDC14A, GPR88, VCAM1, EXTL2, SLC30A7, DPH5, S1PR1, OLFM3
chr1	102271631	103347252	M11,M17	RNU6-352P, DNAJA1P5, OLFM3, COL11A1
chr1	103347252	103348754	M11,M16,M17	COL11A1
chr1	103348754	103354132	M11,M16,M17,M9	COL11A1
chr1	103354132	103480150	M1,M11,M16,M17,M9	COL11A1
chr1	103480150	103481298	M1,M11,M16,M17	COL11A1
chr1	103481298	107682821	M11,M16,M17	RN7SKP285, ACTG1P4, AMYP1, FTLP17, SEPT2P1, SOD2P1, COL11A1, RNPC3, AMY2B, AMY2A, AMY1A, AMY1B, AMY1C, PRMT6, NTNG1
chr1	107682821	109167370	M11,M17	VAV3-AS1, SLC25A24P1, NBPF5P, SLC25A24P2, ST13P21, NTNG1, VAV3, SLC25A24, NBPF4, NBPF6, FAM102B
chr1	109167370	109172279	M17	FAM102B
chr1	116194001	121312129	M14	HNRNPA1P43, ATP1A1OS, RNU6-817P, NAP1L4P1, MIR548AC, MIR320B1, GAPDHP64, NEFHP1, FTH1P22, RNA5SP55, MIR942, RPS15AP9, VPS25P1, RNA5SP56, PSMC1P12, WARS2-IT1, RBMX2P3, RPS3AP12, RPL6P2, RNU1-75P, HAO2-IT1, HSD3BP2, GAPDHP23, HSD3BP1, GAPDHP58, GAPDHP32, HSD3BP3, GAPDHP27, HSD3BP4, GAPDHP33, LINC00622, HSD3BP5, NBPF7, PFN1P9, NOTCH2P1, RNU6-465P, HIST2H3DP1, HIST2H2BA, SRGAP2C, EMBP1, VANGL1, CASQ2, NHLH2, SLC22A15, MAB21L3, ATP1A1, CD58, IGSF3, C1orf137, CD2, PTGFRN, CD101, TTF2, TRIM45, VTCN1, MAN1A2, FAM46C, GDAP2, WDR3, SPAG17, TBX15, WARS2, HAO2, HSD3B2, HSD3B1, ZNF697, PHGDH, HMGCS2, REG4, ADAM30, NOTCH2, FAM72B, FCGR1B
chr1	151846731	153390620	M14	KRT8P28, NBPF18P, SPTLC1P4, HDHD1P2, FLG-AS1, HMGN3P1, LINC00302, SPRR2C, RNU6-160P, THEM4, S100A10, S100A11, TCHHL1, TCHH, RPTN, HRNR, FLG, FLG2, CRNN, LCE5A, CRCT1, LCE3E, LCE3D, LCE3C, LCE3B, LCE3A, LCE2D, LCE2B, LCE2C, LCE2A, LCE4A, C1orf68, KPRP, LCE1F, LCE1E, LCE1D, LCE1C, LCE1B, LCE1A, LCE6A, SMCP, IVL, SPRR4, SPRR1A, SPRR3, SPRR1B, SPRR2D, SPRR2A, SPRR2B, SPRR2E, SPRR2F, SPRR2G, LELP1, PRR9, LOR, PGLYRP3, PGLYRP4, S100A9, S100A12, S100A8, S100A7A
chr1	154215692	155288544	M2	RNU6-239P, RNU6-121P, RNU7-57P, UBE2Q1-AS1, MIR4258, RNU7-150P, HMGN2P18, MIR92B, GBAP1, MTX1P1, RUSC1-AS1, UBAP2L, HAX1, AQP10, ATP8B2, IL6R, SHE, TDRD10, UBE2Q1, CHRNB2, ADAR, KCNN3, PMVK, PBXIP1, PYGO2, SHC1, CKS1B, FLAD1, LENEP, ZBTB7B, DCST2, DCST1, ADAM15, EFNA4, EFNA3, EFNA1, SLC50A1, DPM3, KRTCAP2, TRIM46, MUC1, THBS3, MTX1, GBA, FAM189B, SCAMP3, CLK2, HCN3, PKLR, FDPS
chr1	156618359	156670754	M2	BCAN, NES, CRABP2
chr1	156670754	156737652	M2,M6	CRABP2, ISG20L2, RRNAD1, MRPL24, HDGF, PRCC
chr1	156737652	161147263	M2	MIR765, RN7SL612P, KRT8P45, CYCSP52, VDAC1P9, MRPS21P2, KIRREL-IT1, ELL2P1, HMGN1P5, OR10T1P, EI24P2, OR10R3P, OR10R1P, OR6K1P, OR6K4P, OR6K5P, OR2AQ1P, OR10AA1P, RAD1P2, RNA5SP60, MPTX1, OR10J2P, OR10J7P, OR10J8P, OR10J9P, OR10AE1P, OR10J6P, CRPP1, LINC01133, SUMO1P3, RNU4-42P, SETP9, PRCC, SH2D2A, NTRK1, INSRR, PEAR1, LRRC71, ARHGEF11, ETV3L, ETV3, FCRL5, FCRL4, FCRL3, FCRL2, FCRL1, CD5L, KIRREL, CD1D, CD1A, CD1C, CD1B, CD1E, OR10T2, OR10K2, OR10K1, OR10R2, OR6Y1, OR6P1, OR10X1, OR10Z1, SPTA1, OR6K2, OR6K3, OR6K6, OR6N1, OR6N2, MNDA, PYHIN1, IFI16, AIM2, CADM3, DARC, FCER1A, OR10J3, OR10J4, OR10J1, OR10J5, APCS, CRP, DUSP23, FCRL6, SLAMF8, C1orf204, VSIG8, CCDC19, TAGLN2, IGSF9, SLAMF9, PIGM, KCNJ10, KCNJ9, IGSF8, ATP1A2, ATP1A4, CASQ1, PEA15, DCAF8, PEX19, COPA, NCSTN, NHLH1, VANGL2, SLAMF6, CD84, SLAMF1, CD48, SLAMF7, LY9, CD244, ITLN1, ITLN2, F11R, TSTD1, USF1, ARHGAP30, PVRL4, KLHDC9, PFDN2, NIT1, DEDD, UFC1, USP21, PPOX, B4GALT3
chr1	161147263	161200655	M2,M6	MIR5187, PPOX, B4GALT3, ADAMTS4, NDUFS2, FCER1G, APOA2, TOMM40L, NR1I3
chr1	161200655	161475273	M2	RRM2P2, RNU6-481P, NR1I3, PCP4L1, MPZ, SDHC, C1orf192, FCGR2A
chr1	161475273	179019500	M14,M2	RPS23P10, HSPA7, RPS23P9, RPL31P11, RN7SL466P, MIR4654, RNA5SP61, MIR556, SLAMF6P1, UQCRBP2, RN7SL861P, RNA5SP62, RNA5SP63, NMNAT1P2, RNU5F-6P, HMGB3P6, RNU6-171P, RNU6-755P, RNA5SP64, MIR921, FMO7P, FMO8P, FMO9P, FMO10P, RPL4P2, FMO11P, CNN2P10, DUTP6, RNA5SP65, AKR1D1P1, MIR1255B2, GCSHP5, RPL34P1, ANKRD36BP1, RNU6-1310P, MIR557, QRSL1P1, LINC00626, SUMO1P2, LINC00970, RPL29P7, RNA5SP66, RN7SL333P, RN7SL269P, MRPS10P1, SIGLEC30P, MIR3119-2, MIR3119-1, ISCUP1, LINC01142, HAUS4P1, MIR1295A, HMGB1P11, TOP1P1, GM2AP2, RNU6-290P, CYCSP53, RN7SL425P, RNU6-773P, PFN1P1, RPL4P3, DNM3-IT1, MIR214, DNM3OS, MIR199A2, RNU6-157P, RNU6-693P, SLC25A38P1, AIMP1P2, GOT2P2, RN7SKP160, GAS5-AS1, GAS5, SNORD78, RNA5SP67, RNA5SP68, RC3H1-IT1, RPL30P1, BANF1P4, NDUFAF4P4, RABGAP1L-IT1, RNU6-307P, SDCCAG3P2, TNR-IT1, SCARNA3, RNU2-12P, MORF4L1P7, PTP4A1P7, MIR488, RASAL2-AS1, LINC00083, RNA5SP69, MIR4424, PTPN2P1, FCGR2A, HSPA6, FCGR3A, FCGR2B, FCGR2C, FCGR3B, FCRLA, FCRLB, DUSP12, ATF6, OLFML2B, NOS1AP, C1orf226, C1orf111, SH2D1B, UHMK1, UAP1, DDR2, HSD17B7, C1orf110, RGS4, RGS5, NUF2, PBX1, LMX1A, RXRG, LRRC52, MGST3, ALDH9A1, TMCO1, UCK2, FAM78B, POGK, TADA1, ILDR2, MAEL, GPA33, DUSP27, POU2F1, CD247, CREG1, RCSD1, MPZL1, ADCY10, MPC2, DCAF6, GPR161, TIPRL, SFT2D2, TBX19, XCL2, XCL1, DPT, ATP1B1, NME7, BLZF1, CCDC181, SLC19A2, F5, SELP, C1orf112, SELL, SELE, METTL18, SCYL3, KIFAP3, METTL11B, GORAB, PRRX1, MROH9, FMO3, FMO6P, FMO2, FMO1, FMO4, PRRC2C, MYOC, VAMP4, METTL13, DNM3, PIGC, C1orf105, SUCO, FASLG, TNFSF18, TNFSF4, PRDX6, SLC9C2, ANKRD45, KLHL20, CENPL, DARS2, ZBTB37, SERPINC1, RC3H1, RABGAP1L, GPR52, CACYBP, MRPS14, TNN, KIAA0040, TNR, RFWD2, PAPPA2, ASTN1, BRINP2, SEC16B, RASAL2, TEX35, C1orf220, RALGPS2, ANGPTL1, FAM20B
chr1	179019500	180135625	M14	SETP10, EIF4A1P11, COX5BP8, RN7SL374P, MEF2AP1, HNRNPA1P54, RNU5F-2P, RN7SL230P, FAM20B, TOR3A, ABL2, SOAT1, AXDND1, NPHS2, TDRD5, FAM163A, TOR1AIP2, TOR1AIP1, CEP350, QSOX1
chr1	180135625	183909714	M14,M2	VDAC1P4, MIR3121, OVAAL, RNA5SP70, RN7SKP229, RNU6-152P, LINC00272, TEDDM1P1, HMGN1P4, RNA5SP71, KRT18P28, RNU6-41P, SMG7-AS1, QSOX1, LHX4, ACBD6, XPR1, KIAA1614, STX6, MR1, IER5, CACNA1E, ZNF648, GLUL, TEDDM1, RGSL1, RNASEL, RGS16, RGS8, NPL, DHX9, SHCBP1L, LAMC1, LAMC2, NMNAT2, SMG7, NCF2, ARPC5, RGL1, APOBEC4, COLGALT2
chr1	183909714	185270103	M14,M16,M2	RN7SL654P, RNU7-13P, RNA5SP72, FTH1P25, RPL5P5, COLGALT2, TSEN15, C1orf21, EDEM3, FAM129A, RNF2, TRMT1L, SWT1, IVNS1ABP
chr1	185270103	198713332	M14,M16,M2,M9	RNU7-183P, RNU6-1240P, LINC01036, LINC01037, FDPSP1, ERVMER61-1, RN7SKP156, RPS3AP9, LINC01035, CLPTM1LP1, RNA5SP73, HNRNPA1P46, MIR4426, RN7SKP126, LINC01032, ZNF101P2, MIR1278, LINC01031, RPL23AP22, EEF1A1P14, RNU6-983P, MIR4735, MRPS21P3, EEF1A1P32, FAM204BP, PEBP1P3, IVNS1ABP, HMCN1, PRG4, TPR, C1orf27, OCLM, PDC, PTGS2, PLA2G4A, BRINP3, RGS18, RGS21, RGS1, RGS13, RGS2, UCHL5, TROVE2, GLRX2, CDC73, B3GALT2, KCNT2, CFH, CFHR3, CFHR1, CFHR2, CFHR4, CFHR5, F13B, ASPM, ZBTB41, CRB1, DENND1B, C1orf53, LHX9, NEK7, ATP6V1G3, PTPRC
chr1	198713332	198717237	M14,M9	PTPRC
chr1	198717237	198719755	M14,M2,M9	PTPRC
chr1	198719755	198828056	M14,M2	MIR181A1HG, MIR181B1, PTPRC

chr1	198828056	207494816	M2	PPFIA4, SNORA77, KRT8P29, HSPE1P6, CBX1P3, LINC00303, RNA5SP74, RNA5SP75, SNRPGP10, LEMD1-AS1, MIR135B, RNU2-19P, RNU6-418P, SRGAP2-AS1, MYOG, C4BPAP1, ADORA1, C4BPAP2, MYBPH, CHI3L1, CHIT1, BTG2, FMOD, PRELP, OPTC, ATP2B4, LAX1, ZC3H11A, ZBED6, SNRPE, SOX13, ETNK2, REN, KISS1, GOLT1A, PLEKHA6, PPP1R15B, PIK3C2B, MDM4, MIR181A1HG, MIR181B1, MIR181A1, LRRN2, RNU6-778P, RNU6-716P, RNU6-609P, RNU6-570P, FAM58BP, LINC00862, RPL34P6, MROH3P, RNU6-704P, RPS10P7, IPO9-AS1, MIR5191, RNU6-501P, MIR1231, CRIP1P3, PTPRVP, CYCSP4, SLC25A39P1, PCAT6, HNRNPA1P59, NPM1P40, LINC01136, RNU6-487P, NFASC, CNTN2, TMEM81, RBBP5, DSTYK, TMCC2, NUAK2, KLHDC8A, LEMD1, CDK18, MFSD4, ELK4, SLC45A3, NUCKS1, RAB7L1, SLC41A1, PM20D1, SLC26A9, FAM72A, AVPR1B, C1orf186, CTSE, SRGAP2, IKBKE, C1orf147, RASSF5, EIF2D, DYRK3, MAPKAPK2, IL10, IL19, IL20, IL24, FAIM3, PIGR, FCAMR, C1orf116, YOD1, PFKFB2, C4BPB, C4BPA, NR5A2, ZNF281, KIF14, DDX59, CAMSAP2, GPR25, C1orf106, KIF21B, CACNA1S, ASCL5, TMEM9, IGFN1, PKP1, TNNT2, LAD1, TNNI1, PHLDA3, CSRP1, NAV1, IPO9, SHISA4, LMOD1, TIMM17A, RNPEP, ELF3, GPR37L1, ARL8A, PTPN7, LGR6, UBE2T, PPP1R12B, SYT2, KDM5B, RABIF, KLHL12, ADIPOR1, CYB5R1, TMEM183A
chr1	207494816	207933069	M16,M2	CD46P1, CD55, CR2, CR1, CR1L, CD46
chr1	207933069	214795421	M2	C1orf132, RPS26P13, ATP5G2P1, MIR205HG, MIR4260, SERTAD4-AS1, ST13P19, RNU5A-8P, IPO8P1, KCNH1-IT1, LINC00467, SNX25P1, ARPC3P2, FDPSP8, RN7SL344P, RPL21P28, MIR3122, RN7SKP98, SNORA16B, RN7SL512P, RNU6-423P, FLVCR1-AS1, RPL31P13, PROX1-AS1, KRT18P12, ABHD17AP3, CD46, CD34, PLXNA2, CAMK1G, LAMB3, G0S2, HSD11B1, TRAF3IP3, C1orf74, IRF6, DIEXF, SYT14, SERTAD4, HHAT, KCNH1, RCOR3, TRAF5, RD3, SLC30A1, NEK2, LPGAT1, INTS7, DTL, PPP2R5A, TMEM206, NENF, ATF3, FAM71A, BATF3, NSL1, TATDN3, C1orf227, FLVCR1, VASH2, ANGEL2, RPS6KC1, PROX1, SMYD2, PTPN14, CENPF
chr1	214795421	214826170	M16,M2	CENPF
chr1	214826170	220157599	M16,M2,M9	GAPDHP24, VDAC1P10, MRPS18BP1, SPATA17-AS1, UBBP2, LINC00210, RNU1-141P, RIMKLBP2, ZC3H11B, RNA5SP76, CENPF, KCNK2, KCTD3, USH2A, ESRRG, GPATCH2, SPATA17, RRP15, TGFB2, C1orf143, LYPLAL1, SLC30A10, EPRS
chr1	220157599	222794634	M2,M9	MIR215, MIR194-1, RPS15AP12, SNORA36B, MORF4L1P1, AURKAPS1, XRCC6P3, SLMO2P1, RN7SL464P, HDAC1P2, RNU6ATAC35P, HLA-AS1, RNU6-403P, QRSL1P2, CICP13, RNU6-791P, EPRS, BPNT1, IARS2, RAB3GAP2, MARK1, C1orf115, MARC2, MARC1, HLX, DUSP10, HHIPL2, TAF1A, MIA3
chr1	222794634	249213344	M2	OR9H1P, HSD17B7P1, OR6R1P, OR2X1P, OR2L9P, OR2L1P, OR2L6P, OR2T32P, OR2M1P, OR2AS2P, OR2AS1P, AHCYP8, MIR3124, RNU6-1205P, RN7SL276P, NDUFB1P2, RNU4-57P, SNRPEP10, RNU6-1248P, PHBP11, ACTBP11, CICP5, RNU6-1319P, RN7SKP49, MIR320B2, RNU6-1008P, MIR4742, AKR1B1P1, RNU6-1304P, NDUFA3P3, YBX1P9, RN7SKP165, CDKN2AIPNLP1, ITPKB-IT1, ITPKB-AS1, RPS27P5, BTF3P9, TUBB8P10, TUBB8P9, RNA5SP77, ZNF847P, SNAP47-AS1, MIR5008, CICP26, MIR3620, FAM96AP2, MIR4666A, HIST3H2BA, BTNL10, RNA5SP19, RNA5S1, RNA5S2, RNA5S3, RNA5S4, RNA5S5, RNA5S6, RNA5S7, RNA5S8, RNA5S9, RNA5S10, RNA5S11, RNA5S12, RNA5S13, RNA5S14, RNA5S15, RNA5S16, RNA5S17, RNA5SP18, DUSP5P1, FTH1P2, ISCA1P2, RNU6-180P, RN7SKP276, RNU4-21P, RNA5SP78, HMGN2P19, HMGB1P26, RN7SL467P, RNA5SP79, RN7SL837P, MIR1182, RNA5SP80, SNRPD2P2, LINC00582, RNU5A-5P, DISC1-IT1, RN7SL299P, RNU6-1211P, RNU1-74P, RPS7P3, RNU4-77P, MIR4427, RAC1P7, MIR4671, LINC00184, LINC01132, RNY4P16, RN7SL668P, SNORA14B, ARID4B-IT1, MIR4753, RPL23AP23, TBCE-AS1, RPS21P1, MTND6P14, MTND5P19, MTND4P10, MTND3P8, LDHAP2, LYST-AS1, MIR1537, RNU5E-2P, RNU6-968P, RNU2-70P, ENO1P1, LGALS8-AS1, RPL35P1, RN7SKP195, MIR4428, MTND6P15, MTND5P18, YWHAQP9, RNU6-725P, LINC01139, KRT18P32, MIPEPP2, CHRM3-AS2, CHRM3-AS1, RPS7P5, ADH5P3, RNU5F-8P, PRKRIRP8, RFKP1, HNRNPA1P42, MIR3123, BECN1P1, CFL1P4, RNU6-1139P, TUBB8P6, RNA5SP81, RN7SKP12, RSL24D1P4, RNU6-747P, FCF1P7, MIR4677, FABP7P1, AKT3 IT1, RN7SL148P, TGIF2P1, CYCSP5, HNRNPU-AS1, RN7SKP55, RNU6-947P, RNU6-1089P, RNU6-999P, RNU1-132P, DNAJC19P8, CHCHD4P5, RNU6-1283P, SMYD3-IT1, KIF28P, FGFR3P6, MIR3916, RNA5SP82, VN1R17P, VN1R5, OR2W5, GCSAML-AS1, RNU6-691P, OR14L1P, OR3D1P, MIA3, AIDA, BROX, FAM177B, DISP1, TLR5, SUSD4, C1orf65, CAPN8, CAPN2, TP53BP2, FBXO28, DEGS1, NVL, CNIH4, WDR26, CNIH3, DNAH14, LBR, ENAH, SRP9, EPHX1, TMEM63A, LEFTY1, PYCR2, LEFTY2, SDE2, H3F3A, ACBD3, MIXL1, LIN9, PARP1, C1orf95, ITPKB, PSEN2, ADCK3, CDC42BPA, ZNF678, SNAP47, JMJD4, PRSS38, WNT9A, WNT3A, ARF1, C1orf35, MRPL55, GUK1, GJC2, IBA57-AS1, IBA57, C1orf145, OBSCN, TRIM11, TRIM17, HIST3H3, HIST3H2A, HIST3H2BB, RNF187, RHOU, TMEM78, RAB4A, SPHAR, CCSAP, ACTA1, NUP133, ABCB10, TAF5L, URB2, GALNT2, PGBD5, COG2, AGT, CAPN9, C1orf198, TTC13, ARV1, FAM89A, TRIM67, C1orf131, GNPAT, EXOC8, SPRTN, EGLN1, TSNAX-DISC1, TSNAX, DISC1, SIPA1L2, MAP10, NTPCR, PCNXL2, KCNK1, SLC35F3, COA6, TARBP1, IRF2BP2, TOMM20, RBM34, ARID4B, GGPS1, TBCE, B3GALNT2, GNG4, LYST, NID1, GPR137B, ERO1LB, EDARADD, LGALS8, HEATR1, ACTN2, MTR, MT1HL1, RYR2, ZP4, MTRNR2L11, CHRM3, FMN2, GREM2, RGS7, FH, KMO, OPN3, CHML, WDR64, EXO1, MAP1LC3C, PLD5, CEP170, SDCCAG8, AKT3, ZBTB18, C1orf100, ADSS, C1orf101, DESI2, COX20, HNRNPU, EFCAB2, KIF26B, SMYD3, TFB2M, CNST, SCCPDH, AHCTF1, ZNF670, ZNF695, ZNF669, C1orf229, ZNF124, ZNF496, NLRP3, OR2B11, GCSAML, OR2C3, OR2G2, OR2G3, OR13G1, OR6F1, OR14A2, OR14K1, OR1C1, OR14A16, OR11L1, TRIM58, OR2W3, OR2T8, OR2AJ1, OR2L13, OR2L8, OR2AK2, OR2L5, OR2L2, OR2L3, OR2M5, OR2M2, OR2M3, OR2M4, OR2T33, OR2T12, OR2M7, OR14C36, OR2T4, OR2T6, OR2T1, OR2T7, OR2T2, OR2T3, OR2T5, OR2G6, OR2T29, OR2T34, OR2T10, OR2T11, OR2T35, OR2T27, OR14I1, LYPD8, SH3BP5L, ZNF672, ZNF692, PGBD2
chr10	104154379	104184477	M6	NFKB2, PSD, FBXL15, CUEDC2
chr11	126987	792179	M2	HRAS, LRRC56, C11orf35, RASSF7, PHRF1, IRF7, CDHR5, SCT, DRD4, DEAF1, EPS8L2, TMEM80, TALDO1, PDDC1, CEND1, SLC25A22, BET1L, SCGB1C1, ODF3, RIC8A, SIRT3, PSMD13, NLRP6, ATHL1, IFITM5, IFITM2, IFITM1, IFITM3, B4GALNT4, PKP3, SIGIRR, ANO9, PTDSS2, RNH1, LINC01001, RNU6-447P, RN7SL838P, MIR210HG, MIR210
chr11	792179	842471	M2,M6	SLC25A22, PIDD, RPLP2, PNPLA2, EFCAB4A, CD151, POLR2L, SNORA52
chr11	842471	1774442	M2	MUC5AC, MUC5B, TOLLIP, BRSK2, MOB2, DUSP8, KRTAP5-1, KRTAP5-2, KRTAP5-3, KRTAP5-4, KRTAP5-5, KRTAP5-6, IFITM10, CTSD, POLR2L, TSPAN4, CHID1, AP2A2, MUC6, MUC2, RNU6-1025P, TOLLIP-AS1, KRTAP5-AS1, FAM99A, FAM99B
chr11	1774442	1901385	M2,M6	CTSD, SYT8, TNNI2, LSP1, RPL36AP39, MIR4298
chr11	1901385	4080568	M2	CD81-AS1, RPL26P30, PHLDA2, NAP1L4, KCNQ1OT1, COX6CP18, KCNQ1-AS1, KCNQ1DN, SNORA54, CARS-AS1, RNU1-91P, MRGPRG-AS1, NDUFA5P8, NDUFA5P1, TSSC2, OR7E12P, FAM86GP, RPS3AP39, RPS24P14, OR7E117P, TRPC2, RNU6-1143P, RNU7-50P, MIR4687, HNRNPA1P76, RPS29P20, CARS, OSBPL5, MRGPRG, MRGPRE, ZNF195, ART5, ART1, CHRNA10, LSP1, NUP98, C11orf89, TNNT3, PGAP2, MRPL23, IGF2, INS-IGF2, INS, RHOG, STIM1, TH, ASCL2, C11orf21, TSPAN32, CD81, TSSC4, TRPM5, KCNQ1, CDKN1C, SLC22A18AS, SLC22A18, LINC01150, MRPL23-AS1, H19, MIR483, IGF2-AS, MIR4686, RNU6-878P
chr11	4080568	6625398	M14,M2	RRM1-AS1, OR55B1P, SSU72P5, SSU72P2, SSU72P6, SSU72P4, SSU72P3, SSU72P7, OR52B3P, OR51R1P, OR52P2P, OR52K3P, OR52M2P, OR51A9P, OR51C1P, OR51F5P, OR51C4P, KRT8P49, OR51F3P, OR51F4P, OR51N1P, OR52Y1P, OR51A8P, OR51H2P, OR51A6P, OR51A3P, OR51A5P, OR51P1P, OR52J2P, OR52E1, OR52S1P, OR52E3P, OR52J1P, OR52A4, OR51A1P, OR52Z1, HBBP1, OR51AB1P, OR51B3P, OR51B8P, OR51K1P, OR51A10P, OR52V1P, OR52H2P, OR52B5P, OR52T1P, HNRNPA1P53, OR52U1P, OR52P1P, OR56B2P, OR52N3P, OR52E7P, KRT18P58, RNA5SP329, OR56A7P, OR52L2P, OR52X1P, STIM1, RRM1, OR52B4, TRIM21, OR52K2, OR52K1, OR52M1, C11orf40, OR52I2, OR52I1, TRIM68, OR51D1, OR51E1, OR51E2, MMP26, OR51F1, OR52R1, OR51F2, OR51S1, OR51H1P, OR51T1, OR51A7, OR51G2, OR51G1, OR51A4, OR51A2, OR51L1, OR52J3, OR52E2, OR52A5, OR52A1, OR51V1, HBB, HBD, HBG1, HBG2, HBE1, OR51B4, OR51B2, OR51B5, OR51B6, OR51M1, OR51J1, OR51Q1, OR51I1, OR51I2, OR52D1, UBQLN3, UBQLNL, OR52H1, OR52B6, TRIM6, TRIM6-TRIM34, TRIM34, TRIM5, TRIM22, OR56B1, OR52N4, OR52N5, OR52N1, OR52N2, OR52E6, OR52E8, OR52E4, OR56A3, OR52L1, OR56A4, OR56A1, OR56B4, OR56B3P, OR52B1P, OR52B2, OR52W1, C11orf42, FAM160A2, CNGA4, CCKBR, PRKCDBP, SMPD1, APBB1, HPX, TRIM3, ARFIP2, TIMM10B, DNHD1, RRP8, ILK
chr11	6625398	6648458	M14,M2,M6	ILK, TAF10, TPP1, DCHS1
chr11	6648458	9759759	M14,M2	TMEM9B, NRIP3, SCUBE2, DENND5A, TMEM41B, IPO7, ZNF143, GVINP1, GVINP2, MIR302E, OR10AB1P, OR5P4P, OR5P1P, OR5E1P, RNU6-943P, COX6CP5, SNORA3, SNORA45, RNA5SP330, TMEM9B-AS1, MIR5691, KRT8P41, SNORA23, RN7SL56P, RN7SKP50, WEE1, SWAP70, DCHS1, MRPL17, OR2AG2, OR2AG1, OR6A2, OR10A5, OR10A2, OR10A4, OR2D2, OR2D3, ZNF215, ZNF214, NLRP14, RBMXL2, SYT9, OLFML1, PPFIBP2, CYB5R2, OVCH2, OR5P2, OR5P3, OR10A6, OR10A3, NLRP10, EIF3F, TUB, RIC3, LMO1, STK33, TRIM66, RPL27A, ST5, AKIP1, C11orf16, ASCL3
chr11	9759759	20906989	M11,M14,M2	SBF2-AS1, RNU7-28P, RNU6ATAC33P, MRVI1-AS1, SNORD97, SWAP70, SBF2, ZBED5-AS1, MTND5P21, MIR4299, LINC00958, RASSF10, RN7SKP151, HMGN2P36, FAR1-IT1, RPL39P26, RNA5SP331, SPON1, RNA5SP332, RNU7-49P, CALCP, RN7SL188P, RN7SKP90, RNU6-585P, OR7E14P, SNORD14A, SNORD14B, RNU6-593P, SDHCP4, HIGD1AP5, SAA3P, GLTPP1, SAA2-SAA4, RNA5SP333, ST13P5, RNA5SP334, MIR3159, ADM, SPTY2D1-AS1, AMPD3, PCNAP4, NAV2-IT1, RNA5SP335, NAV2-AS5, NAV2-AS4, MIR4486, MIR4694, NAV2-AS3, NAV2-AS2, NAV2-AS1, MTRNR2L8, RNF141, LYVE1, MRVI1, CTR9, EIF4G2, ZBED5, GALNT18, CSNK2A3, USP47, DKK3, MICAL2, MICALCL, PARVA, TEAD1, ARNTL, BTBD10, PTH, FAR1, RRAS2, COPB1, PSMA1, PDE3B, CYP2R1, CALCB, CALCA, INSC, SOX6, C11orf58, PLEKHA7, RPS13, PIK3C2A, NUCB2, NCR3LG1, KCNJ11, ABCC8, USH1C, OTOG, MYOD1, KCNC1, SERGEF, TPH1, SAAL1, MRGPRX3, MRGPRX4, SAA4, SAA2, SAA1, HPS5, GTF2H1, LDHA, LDHC, LDHAL6A, TSG101, UEVLD, SPTY2D1, TMEM86A, IGSF22, PTPN5, MRGPRX1, MRGPRX2, ZDHHC13, CSRP3, E2F8, NAV2, DBX1, HTATIP2, PRMT3, SLC6A5, NELL1
chr11	20906989	31828473	M11,M14,M16,M2	RNA5SP336, RNA5SP337, RNA5SP338, PRKRIRP4, RNU6-783P, RPL36AP40, BDNF-AS, RNA5SP339, LINC00678, CBX3P1, MIR610, RN7SKP158, OR2BH1P, HNRNPRP2, RN7SL240P, RPL7AP58, CYCSP25, NELL1, ANO5, SLC17A6, FANCF, GAS2, SVIP, CCDC179, LUZP2, ANO3, MUC15, SLC5A12, FIBIN, BBOX1, CCDC34, LGR4, LIN7C, BDNF, KIF18A, METTL15, KCNA4, FSHB, ARL14EP, MPPED2, DCDC1, DNAJC24, IMMP1L, ELP4, PAX6
chr11	31828473	43343686	M11,M14,M2	EIF4A2P5, WT1-AS, HNRNPA3P9, PIGCP1, CSTF3-AS1, RPL29P23, CIR1P3, MIR1343, KRT18P14, MIR3973, LINC00610, RPL7AP56, RNU6-99P, RNU6-365P, HNRNPKP3, PAX6, RCN1, WT1, EIF3M, CCDC73, PRRG4, QSER1, DEPDC7, TCP11L1, CSTF3, HIPK3, KIAA1549L, C11orf91, CD59, FBXO3, LMO2, CAPRIN1, NAT10, ABTB2, CAT, ELF5, EHF, APIP, PDHX, CD44, SLC1A2, PAMR1, FJX1, TRIM44, LDLRAD3, COMMD9, PRR5L, TRAF6, RAG1, RAG2, C11orf74, LRRC4C, API5

chr11	43343686	44331431	M14,M2	RN7SKP287, CTBP2P6, MIR670HG, MIR670, MIR129-2, SEC14L1P1, ALKBH3-AS1, API5, TTC17, HSD17B12, ALKBH3, C11orf96, ACCSL, ACCS, EXT2, ALX4
chr11	44331431	46564948	M2	RPL34P22, MIR4688, MIR3160-1, MIR3160-2, ALX4, CD82, TSPAN18, TP53I11, PRDM11, SYT13, CHST1, SLC35C1, CRY2, MAPK8IP1, C11orf94, PEX16, GYLTL1B, PHF21A, CREB3L1, DGKZ, MDK, CHRM4, AMBRA1
chr11	47189737	47204587	M6	ARFGAP2, PACSIN3
chr11	47644252	47660603	M17,M8	MTCH2
chr11	47660603	47688615	M17	MTCH2, AGBL2
chr11	55029657	55038594	M1	TRIM48
chr11	63953664	64107743	M6	STIP1, FERMT3, TRPT1, NUDT22, DNAJC4, VEGFB, FKBP2, PPP1R14B, PLCB3, BAD, GPR137, KCNK4, TEX40, ESRRA, TRMT112, PRDX5, CCDC88B
chr11	65149412	65688398	M6	KCNK7, MAP3K11, PCNXL3, SIPA1, RELA, KAT5, RNASEH2C, AP5B1, OVOL1, CFL1, SNX32, MUS81, EFEMP2, CTSW, FIBP, CCDC85B, FOSL1, C11orf68, DRAP1, NEAT1, MALAT1, SNRPGP19, SSSCA1-AS1, MIR4690, MIR4489, RN7SL309P, KRT8P26, OVOL1-AS1, SLC25A45, FRMD8, SCYL1, LTBP3, SSSCA1, FAM89B, EHBP1L1
chr11	67766642	67864597	M2	ALDH3B1, MIR4691, UNC93B1, NDUFS8, TCIRG1, CHKA
chr11	68685203	72695261	M2	MIR3164, IFITM9P, ANO1-AS2, RNU6-1175P, ANO1-AS1, H2AFZP4, MIR548K, SHANK2-AS1, SHANK2-AS2, SHANK2-AS3, MIR3664, KRTAP5-14P, OR7E87P, UNC93B6, OR7E4P, RPS3AP41, ENPP7P8, ALG1L9P, ZNF705E, RNA5SP342, OR7E128P, OR7E126P, RNU6-292P, MIR3165, RPEP6, MIR139, RNU7-105P, ARAP1-AS1, ARAP1-AS2, RPS12P20, MIR4692, RNU6-672P, IGHMBP2, MRGPRD, MRGPRF, TPCN2, MYEOV, CCND1, ORAOV1, FGF19, FGF4, FGF3, ANO1, FADD, PPFIA1, CTTN, SHANK2, DHCR7, NADSYN1, KRTAP5-7, KRTAP5-8, KRTAP5-9, KRTAP5-10, KRTAP5-11, FAM86C1, DEFB108B, RNF121, IL18BP, NUMA1, LRTOMT, LAMTOR1, ANAPC15, FOLR3, FOLR1, FOLR2, INPPL1, PHOX2A, CLPB, PDE2A, ARAP1, STARD10, ATG16L2, FCHSD2
chr11	72696106	79151695	M2	GUCY2EP, RNU7-59P, RSF1-IT2, FTH1P16, RSF1-IT1, RNU6-126P, KCTD21-AS1, RNU6-311P, COPS8P3, MIR708, MIR5579, OR8R1P, HMGN2P38, RN7SKP243, CCDC58P5, RNA5SP343, HNRNPA1P40, MIR548AL, CYCSP27, RANP3, RN7SKP297, MIR4696, RN7SL239P, RNU6-216P, OR2AT2P, NPM1P50, OR2AT1P, ZDHHC20P3, MIR326, SNORD15A, SNORD15B, RN7SL786P, PPP1R1AP1, RNA5SP344, FCHSD2, P2RY2, P2RY6, ARHGEF17, RELT, FAM168A, PLEKHB1, RAB6A, MRPL48, COA4, PAAF1, DNAJB13, UCP2, UCP3, C2CD3, PPME1, P4HA3, PGM2L1, KCNE3, LIPT2, POLD3, CHRDL2, RNF169, XRRA1, SPCS2, NEU3, OR2AT4, SLCO2B1, TPBGL, ARRB1, RPS3, KLHL35, GDPD5, SERPINH1, MAP6, MOGAT2, DGAT2, UVRAG, WNT11, PRKRIR, C11orf30, LRRC32, TSKU, ACER3, B3GNT6, CAPN5, OMP, MYO7A, GDPD4, PAK1, CLNS1A, AQP11, RSF1, AAMDC, INTS4, KCTD14, NDUFC2-KCTD14, THRSP, NDUFC2, ALG8, KCTD21, USP35, GAB2, NARS2, TENM4
chr11	85622323	87029178	M11	SLC25A1P1, RNU6-560P, SETP17, RN7SL225P, PTP4A1P6, OR7E13P, OR7E2P, HNRNPCP8, CCDC83, PICALM, EED, C11orf73, CCDC81, ME3, PRSS23, FZD4, TMEM135
chr11	87029178	92568250	M11,M9	PSMA2P1, RNU6-1135P, RNU6-1063P, MIR3166, GAPDHP70, GRM5-AS1, RNU6-16P, CBX3P7, FOLH1B, UBTFL2, TRIM64DP, TRIM53BP, TRIM51BP, MTND1P35, TRIM49D2P, TRIM64, TRIM51EP, TRIM53AP, TRIM64EP, UBTFL1, DISC1FP1, MIR4490, MIR1261, OSBPL9P2, OSBPL9P3, TUBB4BP4, RPL7AP57, NDUFB11P1, PGAM1P9, RPS3AP42, TMEM135, RAB38, CTSC, GRM5, TYR, NOX4, TRIM77, TRIM49, TRIM64B, TRIM49D1, TRIM49C, NAALAD2, CHORDC1, FAT3
chr11	92568250	92569731	M9	FAT3
chr11	92569731	105483161	M11,M9	CASP4, CASP5, CASP1, CARD16, CARD17, CARD18, GRIA4, SNRPGP16, RPL26P31, RN7SL223P, SCARNA9, SNORA25, SNORA32, SNORD6, SNORA1, SNORA8, SNORD5, SNORA18, MIR1304, SNORA40, RN7SL195P, HPRTP4, HPRTP3, MIR548L, LINC01171, ST13P11, CWC15, SRSF8, RNA5SP345, MIR1260B, JRKL-AS1, RNA5SP346, MED28P5, RNA5SP347, RN7SKP53, RPA2P3, RN7SL222P, RN7SKP115, MIR3920, RNU6-952P, WTAPP1, MMP12, RNU7-159P, MTND2P26, MTND1P36, MIR4693, RNA5SP348, CASP1P2, CASP1P1, OR2AL1P, FAT3, MTNR1B, SLC36A4, CCDC67, SMCO4, KIAA1731, TAF1D, C11orf54, MED17, VSTM5, HEPHL1, PANX1, FOLR4, GPR83, MRE11A, ANKRD49, PIWIL4, FUT4, AMOTL1, KDM4D, KDM4E, ENDOD1, SESN3, FAM76B, CEP57, MTMR2, MAML2, CCDC82, JRKL, CNTN5, ARHGAP42, TMEM133, PGR, TRPC6, ANGPTL5, KIAA1377, C11orf70, YAP1, BIRC3, BIRC2, TMEM123, MMP7, MMP20, MMP27, MMP8, MMP10, MMP1, MMP3, MMP13, DCUN1D5, DYNC2H1, PDGFD, DDI1, CASP12
chr11	105483161	107463949	M9	GRIA4, MSANTD4, KBTBD3, AASDHPPT, GUCY1A2, CWF19L2, ALKBH8, ELMOD1, RNU4-55P, RNU6-277P, SMARCE1P1
chr12	88256	149412	M16	DDX11L8, FAM138D
chr12	3390899	6219748	M16	RNU6-174P, HSPA8P5, CCND2-AS1, CCND2-AS2, RN7SL69P, TSPAN9, PRMT8, EFCAB4B, PARP11, CCND2, C12orf5, FGF23, FGF6, C12orf4, RAD51AP1, DYRK4, AKAP3, NDUFA9, GALNT8, KCNA6, KCNA1, KCNA5, NTF3, ANO2, VWF
chr12	6952186	7083551	M6	RPL13P5, DSTNP2, RNU7-1, EMG1, MIR200C, MIR141, SCARNA12, GNB3, CDCA3, USP5, TPI1, SPSB2, LRRC23, ENO2, ATN1, C12orf57, PTPN6, PHB2
chr12	7252496	7802292	M16	PEX5, ACSM4, CD163L1, CD163, APOBEC1, C1RL-AS1, RNU6-485P, GAPDHP31, NIFKP3, C1RL, RBP5, CLSTN3
chr12	9067315	12340053	M16	PHC1, M6PR, KLRG1, A2M, PZP, KLRB1, CLEC2D, CLECL1, CD69, KLRF1, CLEC2B, KLRF2, CLEC2A, CLEC12A, CLEC1B, CLEC12B, CLEC9A, CLEC1A, CLEC7A, OLR1, TMEM52B, GABARAPL1, KLRD1, KLRC4-KLRK1, KLRK1, KLRC4, KLRC3, KLRC2, KLRC1, MAGOHB, STYK1, YBX3, TAS2R7, TAS2R8, TAS2R9, PRR4, TAS2R10, PRH1, TAS2R13, PRH2, TAS2R14, TAS2R50, TAS2R20, TAS2R19, TAS2R31, TAS2R46, TAS2R43, TAS2R30, TAS2R42, PRB3, PRB4, PRB1, PRB2, ETV6, BCL2L14, LRP6, HNRNPA1P34, LINC00612, A2M-AS1, TPT1P12, RNU7-189P, A2MP1, PTMAP4, LINC00987, DDX12P, RNU6-700P, GOT2P3, RN7SKP161, HNRNPABP1, SLC25A39P2, KLRAP1, HSPE1P12, TAS2R15, TAS2R63P, TAS2R64P, TAS2R18, RNU6-676P, TAS2R67P, HIGD1AP8, RNU7-60P
chr12	38710556	41423021	M16,M9	RPL30P13, RNU6-713P, ALG10B, CPNE8, KIF21A, ABCD2, C12orf40, SLC2A13, LRRK2, MUC19, CNTN1
chr12	41423021	48468576	M9	RNA5SP360, MTND2P17, MTND1P24, RN7SL10P, RNU6-249P, MRPS36P5, EEF1A1P17, RNA5SP361, PLEKHA8P1, RNU6-671P, LINC00938, RN7SL246P, KNOP1P2, OR7A19P, MIR4698, PCED1B-AS1, MIR4494, CNTN1, PDZRN4, GXYLT1, YAF2, PPHLN1, ZCRB1, PRICKLE1, ADAMTS20, PUS7L, IRAK4, TWF1, TMEM117, NELL2, DBX2, ANO6, ARID2, SCAF11, SLC38A1, SLC38A2, SLC38A4, AMIGO2, PCED1B, RPAP3, ENDOU, RAPGEF3, SLC48A1, HDAC7, VDR, TMEM106C, COL2A1, SENP1
chr12	59267836	64173909	M16	RPS6P22, RNU6-279P, RNU6-871P, RNU4-20P, RPL21P104, KRT8P19, RPS3P6, KLF17P1, RNU6-595P, RNU6-399P, MIRLET7I, RPL32P26, GAPDHP44, RNU1-83P, RPL14P1, LDHAL6CP, RSL24D1P5, HNRNPA1P69, LRIG3, SLC16A7, FAM19A2, USP15, MON2, C12orf61, PPM1H, AVPR1A, DPY19L2, TMEM5
chr12	70989828	75602986	M16	PTPRB, FAHD2P1, MRS2P2, TRHDE-AS1, PTPRR, CHCHD3P2, RNU6-1012P, TSPAN8, LGR5, ZFC3H1, THAP2, TMEM19, RAB21, TBC1D15, TPH2, TRHDE, ATXN7L3B, KCNC2
chr12	75602986	88542281	M16,M9	GLIPR1L1, GLIPR1L2, GLIPR1, KRR1, CCDC59, PHLDA1, NAP1L1, METTL25, TMTC2, SLC6A15, TSPAN19, LRRIQ1, BBS10, ALX1, RASSF9, OSBPL8, NTS, MGAT4C, C12orf50, C12orf29, ZDHHC17, CEP290, TMTC3, CSRP2, E2F7, NAV3, SYT1, RN7SL734P, RNU6-1271P, RN7SKP172, YWHAQP7, RPL7P43, PAWR, PPP1R12A, MIR1252, RN7SL696P, RNA5SP363, SNRPGP20, RPL7P38, RNU7-106P, RN7SKP261, MIR617, MIR618, MIR4699, OTOGL, RNU6-977P, RPL6P25, RPL23AP68, CYCSP30, MKRN9P, PTPRQ, RPS4XP15, RNA5SP364, MYF6, MYF5, LIN7A, ACSS3, PPFIA2, KCNC2, CAPS2
chr12	88542281	88570096	M9	TMTC3
chr12	122219535	122248124	M6	TMEM120B, RHOF, SETD1B
chr13	19427325	34398053	M14	HMGA1P6, RNY3P4, LINC00362, RNU6-58P, TATDN2P3, SDAD1P4, RPLP1P13, SACS-AS1, LINC00327, LINC00352, MTCO3P2, C1QTNF9B-AS1, ANKRD20A19P, IPO7P2, MIR2276, SPATA13-AS1, C1QTNF9-AS1, NUS1P3, LINC00566, CYCSP33, TPTE2P6, PSPC1P2, RNY1P7, RPL26P34, IRX1P1, ANKRD20A10P, TPTE2P1, SLC25A15P3, RPL34P27, AMER2-AS1, LINC01053, LINC01076, RPL23AP69, TCEB2P1, RN7SL289P, RNU6-78P, RN7SL741P, RNY1P3, LINC00415, ATP8A2P3, PRUNEP1, PRKRIRP6, RPS3AP44, WASF3-AS1, FGFR1OP2P1, RPS21P8, RPS20P32, USP12-AS1, USP12-AS2, LINC00412, SNORD102, SNORA27, RNU6-70P, LINC01079, RNY1P1, RNU6-63P, NPM1P4, RNU6-73P, PDX1-AS1, LINC00543, RN7SL272P, KATNBL1P1, CHCHD2P8, PAN3-AS1, EEF1A1P3, RNU6-82P, EIF4A1P7, RNU6-53P, CYP51A1P2, POM121L13P, MTUS2-AS2, GAPDHP69, MTUS2-AS1, TIMM8BP1, LINC00297, LINC00572, LINC00544, LINC00365, LINC00384, LINC00385, RNU6-64P, PRDX2P1, LINC00427, LINC00426, LINC01058, UBE2L5P, RBM22P2, MFAP1P1, PTPN2P2, LINC00398, TEX26-AS1, LINC00545, LINC01066, WDR95P, ANKRD26P4, EEF1DP3, LINC01073, FRY-AS1, IFIT1P1, ATP8A2P2, RNY1P4, LINC00423, TOMM22P3, STARD13-IT1, STARD13-AS, TUBA3C, TPTE2, MPHOSPH8, PSPC1, ZMYM5, ZMYM2, GJA3, GJB2, GJB6, CRYL1, IFT88, IL17D, N6AMT2, XPO4, LATS2, SAP18, SKA3, MRP63, ZDHHC20, MICU2, FGF9, SGCG, SACS, TNFRSF19, MIPEP, C1QTNF9B, SPATA13, C1QTNF9, PARP4, ATP12A, RNF17, CENPJ, PABPC3, AMER2, MTMR6, NUPL1, ATP8A2, SHISA2, RNF6, CDK8, WASF3, GPR12, USP12, RPL21, RASL11A, GTF3A, MTIF3, LNX2, POLR1D, GSX1, PDX1, ATP5EP2, CDX2, URAD, FLT3, PAN3, FLT1, POMP, SLC46A3, MTUS2, SLC7A1, UBL3, KATNAL1, HMGB1, USPL1, ALOX5AP, MEDAG, TEX26, HSPH1, B3GALTL, RXFP2, FRY, ZAR1L, BRCA2, N4BP2L1, N4BP2L2, PDS5B, KL, STARD13, RFC3, ANKRD20A9P, RNU6-55P, RNU6-76P, SNX19P2, LINC00408, PHF2P2, LINC00442, USP24P1, GTF2IP3, RNA5SP24, CENPIP1, RNU6-52P, SMPD4P2, PSPC1P1, ANKRD26P3, MRPL3P1, LINC00421, PARP4P2, FAM58DP, SLC25A15P2, CYCSP32, MRPS31P2, ESRRAP1, CASC4P1, LINC00350, RN7SL166P, ST6GALNAC4P1, KRR1P1, LINC01072, PPIAP28, LINC00556, MIR4499, SLC35E1P1, RNU2-7P, RANP8, CNOT4P1, HNRNPA1P30, PPIAP27, RPSAP54, RNU6-51P, LATS2-AS1, RNU4-9P, RPS12P23, IPPKP1, RN7SL80P, LINC01046, ESRRAP2, MIPEPP3, GRK6P1, GAPDHP52, RNA5SP25, ZDHHC20-IT1, HIST1H2BPS3, FNTAP2, RNU6-59P, RPS7P10, RN7SL766P, LINC00424, NME1P1, MTND3P1, FTH1P7, DDX39AP1, RPL7AP73, IPMKP1, RFESDP1, LINC00621, BASP1P1, NUS1P2

chr13	34398053	40301685	M14,M16	RNU5A-4P, VDAC1P12, LINC00457, GAMTP2, SCAND3P1, PHBP13, LINC00445, RNU6-71P, HIST1H2APS6, GAPDHP34, TCEAL4P1, ARL2BPP3, NDE1P2, SMAD9-AS1, LAMTOR3P1, RPL29P28, EIF4A1P5, RN7SKP1, RPS12P24, LINC01048, RNA5SP26, HSPD1P9, LINC00571, LINC00437, LINC00366, PRDX3P3, RNU6-56P, FREM2-AS1, PLA2G12AP2, ANKRD26P2, NXT1P1, MIR4305, RFC3, NBEA, MAB21L1, DCLK1, SOHLH2, CCDC169-SOHLH2, CCDC169, SPG20, SPG20OS, CCNA1, SERTM1, RFXAP, SMAD9, ALG5, EXOSC8, SUPT20H, CSNK1A1L, POSTN, TRPC4, UFM1, FREM2, STOML3, PROSER1, NHLRC3, LHFP, COG6
chr13	40301685	60548485	M14	RNY4P14, CDKN2AIPNLP3, AZU1P1, LINC00332, RPL17P51, RNY3P9, RN7SKP2, LINC00598, RLIMP1, MIR320D1, MIR621, TPTE2P5, CYCSP34, SUGT1P3, RGS17P1, MIR3168, RN7SL597P, CALM2P3, MORF4L1P4, RAC1P3, RNU6-57P, TUBBP2, OR7E36P, OR7E155P, OR7E37P, MIR5006, RN7SL515P, RNU6-74P, KARSP1, RPS28P8, MAPK6PS3, CHCHD2P11, FHP1, FABP3P2, LINC01050, LINC00428, ZDHHC4P1, LINC00400, RPL36P19, ENOX1-AS2, ENOX1-AS1, LINC00284, DGKZP1, LINC00390, SMIM2-AS1, SMIM2-IT1, LINC01071, SMARCE1P5, LINC00407, LINC00330, RN7SL49P, RN7SKP3, RNU6-69P, SNORA31, TPT1-AS1, RCN1P2, SLC25A30-AS1, PPIAP25, RNA5SP27, TIMM9P3, COX4I1P2, LINC01055, AKR1B1P4, CPB2-AS1, RN7SL288P, RN7SKP5, FAM206BP, LINC00563, RNU2-6P, RNU6-68P, PPP1R2P4, OR7E101P, COX17P1, FKBP1AP3, HTR2A-AS1, GNG5P5, RN7SL700P, NAP1L4P3, RPL27AP8, LINC00444, LINC00562, SUCLA2-AS1, MED4-AS1, POLR2KP2, LINC00441, PPP1R26P1, PCNPP5, LINC01077, LINC00462, PSME2P2, RNU6-60P, RAD17P2, RNY3P2, COX7CP1, OGFOD1P1, SNRPGP14, RNY4P30, CTAGE10P, RNY4P9, MIR3613, DLEU2, MIR16-1, RPL18P10, ST13P4, RPL34P26, DLEU7-AS1, RNA5SP28, RNASEH2B-AS1, GUCY1B2, RNA5SP29, LINC00371, RPL5P31, SLMO2P2, MIR5693, INTS6-AS1, RPS4XP16, RN7SL320P, SNRPGP11, MIR4703, RNU6-65P, RNY1P6, RN7SL413P, ATP5F1P1, CTAGE3P, FABP5P2, MRPS31P5, TPTE2P2, RNY4P24, LINC00345, TPTE2P3, MRPS31P4, MIR759, PPIAP26, LINC01065, PCDH8P1, RN7SL618P, LINC00558, LINC00458, RPL13AP25, MIR5007, HNF4GP1, SPATA2P1, RN7SKP6, PRR20FP, MTCO2P3, SLC25A5P4, RPL31P53, RNA5SP30, LINC00374, RNY4P29, CTAGE16P, DNAJA1P1, HMGN2P39, POLR3KP1, RPP40P2, RNU7-88P, COG6, FOXO1, MRPS31, SLC25A15, ELF1, WBP4, KBTBD6, KBTBD7, MTRF1, NAA16, RGCC, VWA8, DGKH, AKAP11, TNFSF11, FAM216B, EPSTI1, DNAJC15, ENOX1, CCDC122, LACC1, SMIM2, SERP2, TSC22D1, NUFIP1, GPALPP1, GTF2F2, KCTD4, TPT1, SLC25A30, COG3, FAM194B, SPERT, SIAH3, ZC3H13, CPB2, LCP1, LRRC63, KIAA0226L, LRCH1, ESD, HTR2A, SUCLA2, NUDT15, MED4, ITM2B, RB1, LPAR6, RCBTB2, CYSLTR2, FNDC3A, MLNR, CDADC1, CAB39L, SETDB2, PHF11, RCBTB1, ARL11, EBPL, KPNA3, SPRYD7, TRIM13, KCNRG, DLEU1, DLEU7, RNASEH2B, FAM124A, SERPINE3, INTS6, WDFY2, DHRS12, CCDC70, ATP7B, ALG11, UTP14C, NEK5, NEK3, THSD1, VPS36, CKAP2, HNRNPA1L2, SUGT1, LECT1, PCDH8, OLFM4, PRR20A, PRR20B, PRR20C, PRR20D, PRR20E, PCDH17, DIAPH3
chr13	60548485	61068568	M14,M8	DIAPH3-AS1, RN7SL375P, DIAPH3-AS2, RNY4P28, LINC00434, TARDBPP2, DIAPH3, TDRD3
chr13	61068568	73318699	M14,M16,M8	RNA5SP31, EIF4A1P6, LINC00378, RNY3P5, RNY4P31, MIR3169, RAC1P8, LINC00358, LINC01075, LINC01074, LINC00459, RPL32P28, SQSTM1P1, LINC00448, LINC00376, LINC00395, OR7E156P, RNU6-81P, PPP1R2P10, OR7E104P, NFYAP1, LINC00355, LGMNP1, STARP1, HNRNPA3P5, LINC01052, MIR548X2, MIR4704, TRIM60P19, PCDH9-AS1, RNU7-87P, PCDH9-AS2, PCDH9-AS3, PCDH9-AS4, RPSAP53, LINC00364, BCRP9, NPM1P22, OR7E111P, OR7E33P, ELL2P3, HNRNPA1P18, RPL37P21, RPS3AP52, RPL12P34, RN7SL761P, ZDHHC20P4, SNRPFP3, LINC00383, LINC00401, SRSF1P1, RNY3P10, PSMC1P13, ATXN8OS, RNU6-54P, SOGA2P1, LINC00348, RABEPKP1, H3F3BP1, RPL21P109, RPL35AP31, RPS10P21, RNA5SP32, RPL18AP17, RPL21P110, RNU6-80P, TDRD3, PCDH20, PCDH9, KLHL1, DACH1, MZT1, BORA
chr13	73318699	77566058	M14,M8	RNU6-79P, PSMD10P3, FABP5P1, RNU6-66P, RNU4-10P, RNY1P8, MARK2P12, LINC00393, LINC00392, LINC00402, RNY1P5, RPL21P108, LINC00381, LINC00347, RIOK3P1, RNU6-38P, SSR1P2, CTAGE11P, LINC01078, TBC1D4-AS1, FAM204CP, LINC00561, LINC01034, RN7SL571P, BTF3P11, RPL7P44, DHX9P1, BORA, DIS3, PIBF1, KLF5, KLF12, TBC1D4, COMMD6, UCHL3, LMO7, C13orf45, KCTD12, IRG1, CLN5
chr13	77566058	92003532	M14,M8,M9	MYCBP2-AS1, MYCBP2-AS2, SCEL-AS1, RNY3P7, SPTLC1P5, MIR3665, EDNRB-AS1, RNF219-AS1, RN7SL810P, LINC01069, LINC00446, SRGNP1, RNY3P3, RPL31P54, TCEB1P23, RPL21P111, LINC00331, HSPD1P8, CCT5P2, NIPA2P5, BCAS2P3, RNA5SP33, RBM26-AS1, NDFIP2-AS1, LINC01068, LINC01038, LINC00382, RNU6-61P, HNRNPA1P31, PWWP2AP1, ARF4P4, DPPA3P3, LINC00564, RNU6-77P, HIGD1AP2, PTMAP5, GYG1P2, RNU6-67P, VENTXP2, UBE2D3P4, MTND4P1, MTND5P3, LINC00333, LINC00375, LINC00351, RNU6-72P, MOB1AP1, DDX6P2, TXNL1P1, LINC00430, UBBP5, LIN28AP2, MIR4500HG, MIR4500, LINC00397, TET1P1, RPL29P29, LINC00433, LINC00560, GRPEL2P1, LINC01047, TRIM60P13, LINC00440, SP3P, LINC01040, LINC00353, RPL7L1P1, PEX12P1, LINC00559, RNA5SP34, FAR1P1, KRT18P27, MIR622, LINC01049, RNU6-75P, LINC00410, BRK1P2, LINC00380, LINC00379, PPIAP23, MIR17HG, CLN5, FBXL3, MYCBP2, SCEL, SLAIN1, EDNRB, POU4F1, RNF219, RBM26, NDFIP2, SPRY2, SLITRK1, SLITRK6, SLITRK5
chr13	92003532	111953851	M14,M9	LINC00363, HNRNPA1P29, GPC6-AS2, RNA5SP35, GPC6-AS1, RNA5SP36, RN7SL585P, LINC00391, SOX21-AS1, BRD7P5, RPL21P112, LINC00557, RNU6-62P, RNY3P8, RNY4P27, MEMO1P5, CLDN10-AS1, DNAJC3-AS1, MTND5P2, MTND6P18, MTCYBP3, HMGN1P24, RN7SL164P, AMMECR1LP1, HSP90AB6P, LINC00359, TULP3P1, RN7SKP7, LINC00456, RNA5SP37, PSMA6P4, RPL7AP61, FTLP8, RN7SKP8, MIR3170, FARP1-AS1, STK24-AS1, NUS1P4, CYCSP35, CALM2P4, RN7SL60P, DOCK9-AS1, RPL7L1P12, RNU6-83P, DOCK9-AS2, RPS6P23, GAPDHP22, UBAC2-AS1, RN7SKP9, H2AFZP3, MIR623, HMGB3P4, CCR12P, LINC00449, RNY3P6, LINC01039, CFL1P8, MIR4306, CLYBL-AS2, CLYBL-AS1, LINC00554, NDUFA12P1, ASNSP3, RPL15P18, PCCA-AS1, RPS26P47, COX5BP6, NALCN-AS1, ARF4P3, LINC00411, RNU1-24P, HMGB3P7, MIR2681, LIPT1P1, RNY1P2, MIR4705, RPL39P29, FGF14-IT1, FGF14-AS1, FGF14-AS2, LINC00555, LINC00283, RNY5P8, METTL21EP, ATP6V1G1P7, RPL7P45, DAOA-AS1, LINC00343, RNA5SP38, LINC00460, RPL35P9, LINC00551, LINC00443, ATP5G1P5, PPIAP24, MIR1267, FAM155A-IT1, RNA5SP39, HCFC2P1, MYO16-AS2, MYO16-AS1, LINC00370, LINC01067, LINC00399, LINC00676, RN7SKP10, LINC00396, RN7SL783P, COL4A2-AS2, COL4A2-AS1, RPL21P107, LINC00567, ANKRD10-IT1, PARP1P1, LINC00431, LINC00368, ARHGEF7-AS2, ARHGEF7-IT1, ARHGEF7-AS1, MIR17HG, RNU4ATAC3P, FABP5P4, GPC5-AS2, GPC5-IT1, MIR548AS, GPC5-AS1, GPC5, GPC6, DCT, TGDS, GPR180, SOX21, ABCC4, CLDN10, DZIP1, DNAJC3, UGGT2, HS6ST3, OXGR1, MBNL2, RAP2A, IPO5, FARP1, RNF113B, STK24, SLC15A1, DOCK9, UBAC2, GPR18, GPR183, TM9SF2, CLYBL, ZIC5, ZIC2, PCCA, GGACT, TMTC4, NALCN, ITGBL1, FGF14, TPP2, METTL21C, CCDC168, TEX30, KDELC1, BIVM, BIVM-ERCC5, ERCC5, SLC10A2, DAOA, EFNB2, ARGLU1, FAM155A, LIG4, ABHD13, TNFSF13B, MYO16, IRS2, COL4A1, COL4A2, RAB20, CARKD, CARS2, ING1, LINC00346, ANKRD10, ARHGEF7
chr13	111953851	115092802	M9	LINC00354, LINC00404, LINC00403, LINC01070, LINC01043, LINC01044, ATP11A-AS1, MCF2L-AS1, F10-AS1, KARSP2, LDHBP1, GRTP1-AS1, DCUN1D2-AS, RNU1-16P, LINC00552, GAS6-AS1, GAS6-AS2, LINC00454, LINC00452, LINC00565, RASA3-IT1, MIR548AR, MIR4502, CLCP2, ARHGEF7, TEX29, SOX1, SPACA7, TUBGCP3, C13orf35, ATP11A, MCF2L, F7, F10, PROZ, PCID2, CUL4A, LAMP1, GRTP1, ADPRHL1, DCUN1D2, TMCO3, TFDP1, ATP4B, GRK1, TMEM255B, GAS6, RASA3, CDC16, UPF3A, CHAMP1
chr14	19378082	20793754	M14	OR11H12, POTEG, POTEM, OR4N2, OR4Q3, OR4M1, OR4K2, OR4K5, OR4K1, OR4K15, OR4Q2, OR4K14, OR4K13, OR4L1, OR4K17, OR4N5, OR11G2, OR11H6, OR11H7, OR11H4, TTC5, CCNB1IP1, ARHGAP42P5, NF1P4, MED15P1, RNU6-1239P, GRAMD4P3, BMS1P17, DUXAP10, OR11H13P, BMS1P18, GRAMD4P4, RNU6-1268P, MED15P6, ARHGAP42P4, OR11H2, OR11K2P, OR4H12P, OR4N1P, OR4K6P, OR4K3, OR4K4P, OR4K16P, OR4U1P, RNA5SP380, OR4T1P, RNA5SP381, PSMB7P1, OR11G1P, OR11H5P
chr14	24836282	25103311	M14	NFATC4, NYNRIN, CBLN3, KHNYN, SDR39U1, CMA1, CTSG, GZMH, GZMB
chr14	25103311	31103243	M14,M16	SCFD1, HMGN2P6, OR7K1P, CYB5AP5, NOVA1-AS1, MIR4307, LINC00645, BNIP3P1, RPL26P3, EIF4A1P12, BTF3P2, RNU6-864P, RNU11-5P, RNU6-1234P, SYF2P1, GZMB, STXBP6, NOVA1, FOXG1, C14orf23, PRKD1, G2E3
chr14	31103243	31856325	M14	SCFD1, COCH, STRN3, AP4S1, HECTD1, HEATR5A, UBE2CP1, HIGD1AP17, MIR624, RPL21P5, RNU6-541P
chr14	31856325	34420284	M14,M16	HEATR5A, DTD2, GPR33, NUBPL, ARHGAP5, AKAP6, NPAS3, EGLN3, RNU6-602P, RNU6-455P, ARHGAP5-AS1, RNU6-7, RNU6-8, RN7SL660P
chr14	34420284	54989241	M14	RNU6ATAC30P, RNU6-539P, RN7SL3, RN7SL2, RNU6-189P, PDLIM1P1, RN7SKP193, MIR4504, SNRPGP1, RN7SL452P, ZFP64P1, LINC00519, LINC00640, SETP2, FRMD6-AS2, RNU6-1291P, RNU6-1033P, RNA5SP385, RNU6-301P, OR7E105P, OR7E106P, OR7E159P, COX5AP2, RN7SL588P, RPS3AP46, MIR5580, ATP5C1P1, EGLN3, SPTSSA, EAPP, SNX6, CFL2, BAZ1A, SRP54, FAM177A1, PPP2R3C, KIAA0391, PSMA6, NFKBIA, INSM2, RALGAPA1, EGLN3-AS1, RNU1-27P, RNU1-28P, RPS19P3, RNU6-1261P, RNU7-41P, BRMS1L, MBIP, IGBP1P1, RPL7AP3, RPL9P3, DNAJC8P1, RPS3AP4, KRT18P6, QRSL1P3, NUTF2P2, ILF2P2, LINC00609, PTCSC3, SFTA3, RN7SKP21, DPPA3P2, RNU7-93P, NKX2-1-AS1, PHKBP2, RPL29P3, RN7SKP257, MIR4503, RNU6-273P, RNU6-886P, NKX2-1, RNU6-1277P, LINC00517, KRT8P1, NKX2-8, PAX9, LINC00639, SLC25A21, RPL7AP2, SLC25A21-AS1, MIPOL1, HNRNPUP1, KRT8P2, SNORD127, RNU6-552P, DNAJC19P9, LINC00871, RPA2P1, RPL13AP2, RPS15AP3, MIR548Y, LINC00648, RNU6-297P, RNA5SP384, RPL32P29, RN7SL1, RHOQP1, FOXA1, TTC6, SSTR1, CLEC14A, SEC23A, GEMIN2, TRAPPC6B, PNN, MIA2, CTAGE5, FBXO33, LRFN5, FSCB, C14orf28, KLHL28, FAM179B, PRPF39, FKBP3, FANCM, MIS18BP1, RPL10L, MDGA2, RPS29, LRR1, RPL36AL, MGAT2, DNAAF2, POLE2, KLHDC1, KLHDC2, NEMF, ARF6, C14orf182, C14orf183, VCPKMT, SOS2, L2HGDH, ATP5S, CDKL1, MAP4K5, ATL1, SAV1, NIN, PYGL, ABHD12B, TRIM9, TMX1, FRMD6, GNG2, C14orf166, NID2, PTGDR, PTGER2, TXNDC16, GPR137C, ERO1L, PSMC6, STYX, GNPNAT1, FERMT2, DDHD1, BMP4, CDKN3, CNIH1, GMFB, CGRRF1
chr14	59655442	60212812	M14	MIR5586, DAAM1, GPR135, L3HYPDH, JKAMP, CCDC175, RTN1
chr14	101365293	101391157	M17	MEG8, MIR370
chr14	101391157	101451184	M17,M8	MEG8, SNORD113-1, SNORD113-2, SNORD113-3, SNORD113-4, SNORD113-5, SNORD113-6, SNORD113-7, SNORD113-8, SNORD113-9, SNORD114-1, SNORD114-2, SNORD114-3, SNORD114-4, SNORD114-5, SNORD114-6, SNORD114-7, SNORD114-9, SNORD114-10, SNORD114-11, SNORD114-12, SNORD114-13, SNORD114-14, SNORD114-15, SNORD114-16, SNORD114-17, SNORD114-18, SNORD114-19, SNORD114-20, SNORD114-21, SNORD114-22, SNORD114-23, SNORD114-24
chr14	101451184	101489757	M17	SNORD114-24, SNORD114-25, SNORD114-26, SNORD114-27, SNORD114-28, SNORD114-29, SNORD114-30, SNORD114-31, MIR379, MIR411
chr14	103570333	103806057	M6	LINC00677, RPL21P12, GCSHP2, LINC00605, RPL21P13, RAP2CP1, SNORA28, EXOC3L4, TNFAIP2, EIF5

chr15	20739496	22840340	M16,M2	GOLGA6L6, POTEB2, IGHD5OR15-5B, IGHD4OR15-4B, IGHD3OR15-3B, IGHD2OR15-2B, IGHD1OR15-1B, POTEB, OR4M2, OR4N4, IGHV1OR15-1, IGHV4OR15-8, GOLGA6L1, TUBGCP5, GOLGA8CP, RN7SL759P, SPATA31E2P, IGHV1OR15-6, NBEAP1, RNU6-498P, GRAMD4P5, MIR3118-4, MIR3118-6, RNU6-749P, NF1P1, MIR5701-1, MIR5701-2, CT60, OR11J2P, OR11J5P, KIAA0125P2, RN7SL400P, BMS1P16, RNU6-1235P, CXADRP2, GRAMD4P6, RNU6-631P, NF1P2, OR11J1P, OR11H3P, OR11K1P, OR4Q1P, OR4H6P, OR4N3P, IGHV1OR15-3, IGHV1OR15-4, MIR1268A, MIR4509-1, MIR4509-3, MIR4509-2, ABCB10P1, GOLGA8DP, RN7SL545P, ELMO2P1
chr15	22840340	28391382	M2	TUBGCP5, CYFIP1, NIPA2, NIPA1, GOLGA8I, GOLGA8S, GOLGA6L2, MKRN3, MAGEL2, NDN, NPAP1, SNRPN, SNURF, UBE3A, ATP10A, GABRB3, GABRA5, GABRG3, OCA2, HERC2, WHAMMP3, RN7SL495P, HERC2P2, HERC2P7, GOLGA8EP, RN7SL106P, SPATA31E3P, RN7SL536P, MIR4508, RNU6-741P, PWRN4, PWRN2, PWRN3, PWRN1, RPL5P1, SNHG14, SNORD64, SNORD108, PWAR6, SNORD109A, SNORD116-1, SNORD116-2, SNORD116-3, SNORD116-5, SNORD116-6, SNORD116-7, SNORD116-8, SNORD116-9, SNORD116-10, SNORD116-11, SNORD116-12, SNORD116-13, SNORD116-14, SNORD116-15, SNORD116-16, SNORD116-17, SNORD116-18, SNORD116-19, SNORD116-21, SNORD116-20, SNORD116-23, SNORD116-24, SNORD116-25, SNORD116-26, SNORD116-27, SNORD116-29, SNORD116-30, TMEM261P1, SNORD115-1, SNORD115-2, SNORD115-3, SNORD115-4, SNORD115-5, SNORD115-6, SNORD115-8, SNORD115-9, SNORD115-10, SNORD115-11, SNORD115-12, SNORD115-14, SNORD115-15, SNORD115-16, SNORD115-17, SNORD115-18, SNORD115-19, SNORD115-20, SNORD115-21, SNORD115-22, SNORD115-23, SNORD115-24, SNORD115-25, SNORD115-27, SNORD115-28, SNORD115-29, SNORD115-30, SNORD115-31, SNORD115-32, SNORD115-33, SNORD115-34, SNORD115-35, SNORD115-36, SNORD115-37, SNORD115-38, SNORD115-39, SNORD115-40, SNORD115-41, SNORD115-42, SNORD115-43, SNORD115-44, SNORD115-45, SNORD115-47, SNORD115-48, SNORD109B, RNA5SP390, MIR4715, LINC00929, TVP23BP1, RNA5SP391, RPL5P32
chr15	28391382	28996820	M17,M2	HERC2, GOLGA8F, GOLGA8G, GOLGA8M, RPL41P2, RN7SL238P, ABCB10P3, MIR4509-1, MIR4509-3, MIR4509-2, ABCB10P4, RN7SL829P, HERC2P9, RN7SL719P, WHAMMP2
chr15	28996820	33261678	M2	APBA2, FAM189A1, NDNL2, TJP1, GOLGA8J, GOLGA8T, CHRFAM7A, GOLGA8R, GOLGA8Q, GOLGA8H, ARHGAP11B, FAN1, MTMR10, TRPM1, KLF13, OTUD7A, CHRNA7, GOLGA8K, GOLGA8O, GOLGA8N, ARHGAP11A, SCG5, GREM1, FMN1, WHAMMP2, GOLGA6L7P, TUBBP8, HMGN2P5, RN7SL673P, DNM1P28, ULK4P3, RN7SL469P, DNM1P30, RNU6-17P, RN7SL196P, RN7SL796P, DNM1P50, ULK4P2, RN7SL628P, GOLGA8UP, RN7SL82P, HERC2P10, RNU6-466P, MIR211, UBE2CP4, RNU6-18P, DNM1P31, RN7SL185P, ULK4P1, DNM1P32, RN7SL539P, RN7SL286P
chr15	40586543	40683724	M6	PLCB2-AS1, LINC00984, RNA5SP392, LINC00594, PLCB2, C15orf52, PHGR1, DISP2, KNSTRN
chr15	48010877	48426454	M14	SEMA6D, SLC24A5
chr15	48426454	50264882	M14,M16	SECISBP2L, COPS2, GALK2, FAM227B, FGF7, DTWD1, ATP8B4, KRT8P24, RN7SL577P, NDUFAF4P1, MIR4716, RN7SL307P, SLC24A5, MYEF2, CTXN2, SLC12A1, DUT, FBN1, CEP152, SHC4, EID1
chr15	50264882	50303114	M16	ATP8B4, RNA5SP394
chr15	52903771	64050568	M2	TPM1, LACTB, RPS27L, RAB8B, APH1B, CA12, USP3, FBXL22, RPSAP55, EEF1A1P22, RNU2-53P, HERC1, RNU6-449P, HNRNPA1P74, DYX1C1-CCPG1, MIR628, CNOT6LP1, RN7SL568P, CD24P2, RNU6-1287P, HNRNPA3P11, LINC00926, RNU6-844P, RN7SKP95, ZNF444P1, MIR2116, RNU4-80P, RNU6-212P, RNA5SP396, PIGHP1, RPS3AP6, NMNAT1P5, MESTP2, CYCSP38, RNA5SP397, RN7SL613P, NPM1P47, HMGN1P26, MIR190A, USP3-AS1, FAM214A, ONECUT1, WDR72, UNC13C, RSL24D1, RAB27A, PIGB, CCPG1, C15orf65, DYX1C1, PYGO1, PRTG, NEDD4, RFX7, TEX9, MNS1, ZNF280D, TCF12, CGNL1, GCOM1, MYZAP, POLR2M, ALDH1A2, AQP9, LIPC, ADAM10, FAM63B, RNF111, SLTM, CCNB2, MYO1E, LDHAL6B, FAM81A, GCNT3, GTF2A2, BNIP2, FOXB1, ANXA2, NARG2, RORA, VPS13C, C2CD4A, C2CD4B, TLN2
chr15	65345674	102516807	M2	CRTC3, MIR1827, RPL18P11, RPL21P116, RNU6-415P, MIR184, HNRNPCP3, BLM, RNU6-667P, LINC00927, MIR5572, RNU6-380P, MIR549, MIR4514, ANP32BP3, RNU1-77P, ADAMTS7P1, FURIN, UBE2Q2P2, UBE2Q2P6, CSPG4P8, FES, RN7SL61P, GOLGA6L17P, DNM1P38, ADAMTS7P2, RN7SL256P, CSPG4P9, UBE2Q2P3, RPL9P8, CSPG4P10, MAN2A2, RN7SL410P, GOLGA6L21P, DNM1P42, SCARNA15, MIR4515, RNU6-401P, TUBA8P2, RNU6-1339P, EFTUD1P1, CSPG4P11, GOLGA2P7, RN7SL331P, UBE2Q2P8, RN7SL417P, DNM1P51, CSPG4P5, UBE2Q2P11, UBE2Q2P12, GOLGA6L5P, UBE2Q2P1, UNC45A, LINC00933, SCAND2P, EGLN1P1, RNU6-339P, RNU6-796P, NIFKP8, CSPG4P12, RN7SL428P, GOLGA6L3, ADAMTS7P4, RNU7-79P, RNU6-1280P, MIR1276, RNU6-231P, MIR548AP, RNA5SP400, AGBL1-AS1, HDDC3, RNU6-185P, LINC00052, MED28P6, NTRK3-AS1, RCCD1, MIR1179, MIR7-2, KRT18P47, RNU7-195P, HMGB1P8, PRC1, LINC00925, MIR9-3, LINC00928, RPL36AP43, RNU6-132P, MRPL15P1, MIR5094, MIR5009, RNU6-1111P, RNU7-111P, MIR3174, RN7SL755P, RN7SL346P, VPS33B, RPS12P26, RN7SL736P, NDUFA3P4, HSPE1P3, RNU6-686P, RN7SL363P, RNU5A-1, RNU5B-1, RNU6-19P, PRC1-AS1, MIR4511, HNRNPA1P44, RNU6-265P, MIR4311, SCARNA14, RPL9P25, ATP5J2P6, RPL35AP32, MIR4512, SNORD18C, SNORD18B, SNORD16, SNORD18A, DUXAP6, NPM1P5, ENO1P2, HMGN2P47, RPS24P16, IQCH-AS1, HNRNPA1P5, RNU6-1, LINC00930, HMGN1P38, ASB9P1, RN7SL599P, HMGN2P40, MIR3175, SV2B, MIR4312, LINC00277, RN7SL438P, SLCO3A1, ST8SIA2, LINC00924, RNU2-3P, NR2F2-AS1, MIR1469, PGAM1P12, C15orf32, RN7SKP254, FAM149B1P1, SPATA8-AS1, RN7SKP181, FAM174B, RN7SL677P, RNA5SP401, RNU6-134P, RNU6-1186P, CHD2, MIR4714, HSP90B2P, HNRNPA1P62, RPL7P5, DNM1P46, RN7SL484P, RNA5SP402, SPATA41, RNU6-322P, PRKXP1, RNU6-181P, RNU6-807P, DNM1P47, RGMA, RN7SL209P, OR4F14P, OR4F13P, OR4F28P, SLC51B, RNA5SP398, WBP1LP5, RASL12, OR4G2P, FAM138E, MIR1302-11, MIR1302-10, MIR1302-9, MIR1302-2, KBTBD13, UBAP1L, LINC00593, WASH3P, MIR629, RNU6-745P, RPL29P30, KRT8P9, RPL5P3, MCTP2, PDCD7, NR2E3, RNA5SP399, RNU2-65P, HEXA-AS1, MIR630, RN7SL485P, RN7SL853P, ADPGK-AS1, NPM1P42, FKBP1AP2, DDX11L9, NPM1P43, MRPS15P1, LOXL1-AS1, DNM1P33, RN7SL429P, UBL7-AS1, MIR4513, RPL36AP45, NIFKP4, CLPX, RN7SL489P, RN7SL327P, DNM1P34, ANP32BP1, MIR631, RPL13P4, DNM1P35, MIR4313, CILP, RN7SL319P, DNM1P49, RN7SL510P, TYRO3P, MIR3713, RN7SKP217, PARP16, RN7SL278P, KRT8P23, CSPG4P13, IGDCC3, IGDCC4, RN7SL214P, NR2F2, ADAMTS7P3, DPP8, SPATA8, LINC00923, ARRDC4, FAM169B, IGF1R, PGPEP1L, SYNM, PTPLAD1, TTC23, LRRC28, VWA9, MEF2A, SLC24A1, DENND4A, RAB11A, MEGF11, LYSMD4, ADAMTS17, CERS3, LINS, DIS3L, ASB7, ALDH1A3, TIPIN, MAP2K1, SNAPC5, RPL4, LRRK1, CHSY1, VIMP, SNRPA1, PCSK6, ZWILCH, LCTL, SMAD6, SMAD3, TM2D3, AAGAB, TARSL2, IQCH, OR4F6, OR4F15, OR4F4, C15orf61, MAP2K5, SKOR1, PIAS1, CALML4, CLN6, FEM1B, ITGA11, CORO2B, ANP32A, SPESP1, NOX5, GLCE, PAQR5, KIF23, RPLP1, TLE3, UACA, LARP6, LRRC49, THAP10, THSD4, CT62, MYO9A, SENP8, GRAMD2, PKM, PARP6, CELF6, HEXA, TMEM202, ARIH1, GOLGA6B, HIGD2B, BBS4, ADPGK, NEO1, HCN4, C15orf60, NPTN, CD276, C15orf59, TBC1D21, LOXL1, STOML1, PML, GOLGA6A, ISLR2, ISLR, STRA6, CCDC33, CYP11A1, SEMA7A, UBL7, ARID3B, CLK3, EDC3, CYP1A1, CYP1A2, CSK, LMAN1L, CPLX3, ULK3, SCAMP2, MPI, FAM219B, COX5A, RPP25, SCAMP5, PPCDC, C15orf39, GOLGA6C, GOLGA6D, COMMD4, NEIL1, MAN2C1, SIN3A, PTPN9, SNUPN, IMP3, SNX33, CSPG4, ODF3L1, UBE2Q2, FBXO22, NRG4, C15orf27, ETFA, ISL2, SCAPER, RCN2, PSTPIP1, TSPAN3, PEAK1, HMG20A, LINGO1, TBC1D2B, SH2D7, CIB2, IDH3A, ACSBG1, DNAJA4, WDR61, CRABP1, IREB2, HYKK, PSMA4, CHRNA5, CHRNA3, CHRNB4, ADAMTS7, MORF4L1, CTSH, RASGRF1, ANKRD34C, TMED3, KIAA1024, MTHFS, ST20-MTHFS, ST20, C15orf37, BCL2A1, ZFAND6, FAH, ARNT2, ABHD17C, KIAA1199, MESDC2, MESDC1, C15orf26, IL16, STARD5, TMC3, MEX3B, EFTUD1, FAM154B, GOLGA6L10, GOLGA6L9, RPS17, GOLGA6L18, GOLGA6L19, GOLGA6L20, RPS17L, CPEB1, AP3B2, FSD2, WHAMM, HOMER2, FAM103A1, C15orf40, BTBD1, TM6SF1, BNC1, SH3GL3, ADAMTSL3, UBE2Q2L, GOLGA6L4, ZSCAN2, WDR73, NMB, SEC11A, ZNF592, ALPK3, SLC28A1, PDE8A, AKAP13, KLHL25, AGBL1, NTRK3, MRPL46, MRPS11, DET1, AEN, ISG20, ACAN, HAPLN3, MFGE8, ABHD2, RLBP1, FANCI, POLG, RHCG, TICRR, KIF7, PLIN1, PEX11A, WDR93, MESP1, MESP2, ANPEP, AP3S2, C15orf38-AP3S2, C15orf38, ZNF710, IDH2, SEMA4B, CIB1, GDPGP1, TTLL13, NGRN, GABARAPL3, ZNF774, IQGAP1
chr16	1813720	2053672	M6	RN7SL367P, SNORA10, SNORA64, SNHG9, MAPK8IP3, NME3, MRPS34, EME2, SPSB3, NUBP2, IGFALS, HAGH, FAHD1, MEIOB, HS3ST6, MSRB1, RPL3L, NDUFB10, RPS2, RNF151, TBL3, NOXO1, GFER, SYNGR3, ZNF598
chr16	2201728	2283543	M6	SNORD60, RAB26, TRAF7, CASKIN1, MLST8, BRICD5, PGP, E4F1
chr16	20410420	20749278	M16	PDILT, ACSM5, ACSM2A, ACSM2B, ACSM3, ACSM1, THUMPD1
chr16	29789593	29828571	M6	ZG16, KIF22, MAZ, PRRT2, PAGR1
chr16	29979460	30409682	M6	TMEM219, TAOK2, HIRIP3, INO80E, DOC2A, C16orf92, FAM57B, ALDOA, SLX1A-SULT1A3, PPP4C, TBX6, YPEL3, GDPD3, MAPK3, CORO1A, BOLA2B, SLX1A, SULT1A3, CD2BP2, TBC1D10B, MYLPF, ZNF48, SEPT1
chr16	76311175	80838175	M16	RN7SKP233, MIR4719, VN2R10P, KRT8P22, LSM3P5, RPS3P7, RNA5SP431, CNTNAP4, MON1B, SYCE1L, ADAMTS18, NUDT7, VAT1L, CLEC3A, WWOX, MAF, DYNLRB2, CDYL2
chr17	1933477	2076052	M6	MIR132, MIR212, DPH1, OVCA2, HIC1, SMG6
chr17	2276310	2324111	M6	SGSM2, MNT, METTL16
chr17	4613913	4641743	M6	ARRB2, MED11, CXCL16
chr17	4796317	4889226	M6	MINK1, CHRNE, C17orf107, GP1BA, SLC25A11, RNF167, PFN1, ENO3, SPAG7, CAMTA2
chr17	6909261	6946320	M6	ALOX12, RNASEK, RNASEK-C17orf49, C17orf49, BCL6B, SLC16A13, SLC16A11, MIR497HG
chr17	7096349	7232493	M6	DLG4, ACADVL, DVL2, PHF23, GABARAP, CTDNEP1, ELP5, CLDN7, MIR324, SLC2A4, YBX2, EIF5A, GPS2, NEURL4
chr17	7460810	7496111	M6	SENP3-EIF4A1, SNORA67, SNORA48, SNORD10, TNFSF12, TNFSF12-TNFSF13, TNFSF13, SENP3, EIF4A1, CD68, MPDU1, SOX15, FXR2
chr17	7727949	7764148	M6	DNAH2, KDM6B, TMEM88, LSMD1, CYB5D1
chr17	8026396	8159175	M6	HES7, PER1, VAMP2, TMEM107, C17orf59, AURKB, CTC1, PFAS, SNORD118, LINC00324
chr17	10297565	10443383	M11	MYH8, MYH4, MYH1, MYH2
chr17	29496908	29687721	M16	AK4P1, NF1, OMG, EVI2B, EVI2A
chr17	38209591	38278901	M6	MED24, THRA, NR1D1, MSL1

chr17	40714164	40734757	M6	COASY, MLX, PSMC3IP, FAM134C
chr17	42232263	42298178	M6	ASB16-AS1, C17orf53, ASB16, TMUB2, ATXN7L3, UBTF
chr17	49337311	49731001	M14	RPL7P48, RN7SL699P, MBTD1, UTP18, CA10
chr17	49731001	53150437	M14,M16	RPS2P48, ISCA1P3, ARL2BPP8, RN7SKP14, CA10, C17orf112, KIF2B, TOM1L1, COX11, STXBP4
chr17	53150437	54939556	M14	RNU6-1249P, RPL39P33, STXBP4, HLF, MMD, TMEM100, PCTP, ANKFN1, NOG, C17orf67, DGKE
chr17	66548052	71202346	M14	ABCA5, MAP2K6, KCNJ16, KCNJ2, SOX9, SLC39A11, SSTR2, COG1, FAM20A, ABCA8, ABCA9-AS1, MIR4524B, MIR4524A, SNRPGP4, LINC01028, KCNJ2-AS1, CALM2P1, CASC17, RNU7-155P, RNU6-305P, LINC01152, SOX9-AS1, LINC00511, RPL32P33, RN7SKP180, POLR3KP2, ABCA9, ABCA6, ABCA10
chr17	78355346	79857117	M2	AATK, MIR4740, MIR3186, AZI1, ENTHD2, C17orf89, SLC38A10, TMEM105, ACTG1, FSCN2, C17orf70, NPLOC4, TSPAN10, PDE6G, OXLD1, CCDC137, ARL16, HGS, MRPL12, SLC25A10, GCGR, FAM195B, PPP1R27, P4HB, MIR4730, RPL32P31, RPL31P7, RPL12P37, BAIAP2-AS1, MIR657, MIR3065, MIR338, MIR1250, AATK-AS1, LINC00482, ARHGDIA, ALYREF, ANAPC11, RNF213, ENDOV, NPTX1, RPTOR, CHMP6, BAIAP2
chr17	79857117	80059742	M2,M9	MAFG-AS1, ANAPC11, NPB, PCYT2, SIRT7, MAFG, PYCR1, MYADML2, NOTUM, ASPSCR1, STRA13, LRRC45, RAC3, DCXR, RFNG, GPS1, DUS1L, FASN, CCDC57
chr17	80059742	81188573	M2	NARF-IT1, MIR4525, CCDC57, SLC16A3, CSNK1D, CD7, SECTM1, TEX19, UTS2R, OGFOD3, HEXDC, C17orf62, NARF, FOXK2, WDR45B, RAB40B, FN3KRP, FN3K, TBCD, ZNF750, B3GNTL1, METRNL
chr18	163380	44098841	M14	RN7SL247P, MIR320C2, RAC1P1, WBP2P1, RN7SL97P, RPS24P18, DHFRP1, NPM1P2, FAM60BP, RNU6-1289P, CIAPIN1P, AQP4-AS1, LDLRAD4, UBA52P9, RBM22P1, PA2G4P3, ARIH2P1, RNU6-408P, RNU6-857P, RNU6-167P, RNU6-1002P, RN7SKP44, LRRC37A7P, RNU6-1050P, PGDP1, RNA5SP453, HNRNPA1P7, WBP11P1, FAM210A, RNMT, ZNF271, NRBF2P1, MIR187, MIR3929, MIR4318, RN7SKP182, RNU6-706P, LINC00669, RPL7AP66, MIR5583-1, RNU6-1242P, RPL17P45, RNU7-145P, LINC00907, MC5R, MC2R, RNA5SP454, RNU6-443P, RNA5SP455, KRT8P5, MIR4319, ZNF519, RN7SKP26, RNU6-1278P, POTEC, ANKRD30B, ROCK1, GREB1L, ESCO1, SNRPD1, ABHD3, MIB1, GATA6, CTAGE1, RBBP8, CABLES1, TMEM241, RIOK3, C18orf8, NPC1, ANKRD29, LAMA3, TTC39C, CABYR, OSBPL1A, IMPACT, HRH4, ZNF521, SS18, PSMA8, TAF4B, KCTD1, AQP4, CHST9, CDH2, DSC3, DSC2, DSC1, DSG1, DSG4, DSG3, DSG2, TTR, B4GALT6, SLC25A52, TRAPPC8, RNF125, RNF138, GAREM, MEP1B, KLHL14, CCDC178, ASXL3, NOL4, DTNA, MAPRE2, ZNF397, ZSCAN30, ZNF24, ZNF396, INO80C, GALNT1, C18orf21, RPRD1A, SLC39A6, ELP2, MOCOS, FHOD3, USP14, TPGS2, THOC1, COLEC12, KIAA1328, CETN1, CLUL1, C18orf56, CELF4, TYMS, ENOSF1, PIK3C3, RIT2, SYT4, YES1, ADCYAP1, METTL4, SETBP1, SLC14A2, NDC80, SLC14A1, SMCHD1, SIGLEC15, EPG5, EMILIN2, LPIN2, PSTPIP2, MYOM1, ATP5A1, HAUS1, MYL12A, MYL12B, TGIF1, C18orf25, RNF165, LOXHD1, DLGAP1, C18orf42, ZBTB14, EPB41L3, TMEM200C, L3MBTL4, C18orf64, ARHGAP28, LAMA1, LRRC30, PTPRM, RAB12, SOGA2, NDUFV2, ANKRD12, TWSG1, RALBP1, PPP4R1, RAB31, RN7SKP146, RNU1-109P, TXNDC2, COX6CP3, LINC00470, RN7SKP72, VAPA, KATNBL1P3, RNU6-340P, CBX3P2, RNU7-25P, RPL31P59, IGLJCOR18, RPL21P127, RN7SL39P, DLGAP1-AS1, DLGAP1-AS2, DLGAP1-AS3, RNU6-831P, DLGAP1-AS4, GAPDHP66, DLGAP1-AS5, APCDD1, NAPG, PPIAP14, LINC00526, LINC00667, MIR3976, RNU5F-3P, RN7SL723P, RNU6-349P, RPL6P27, RN7SL282P, LINC00668, SCML2P1, RNU6-916P, RN7SL537P, SLC25A51P2, PIEZO2, RFWD2P1, RN7SL50P, THEMIS3P, AKR1B1P6, RPS4XP19, RNU2-27P, RNU6-903P, KRT18P8, RN7SL862P, RNA5SP449, PIGPP4, RNA5SP450, CCDC58P1, PMM2P1, GNAL, NPIPB1P, CHMP1B, RNU6-324P, MPPE1, CCDC58P3, RNU6-170P, RNU7-129P, LDLRAD4-AS1, MIR5190, MIR4526, RN7SL362P, NF1P5, ANKRD20A5P, RNU6-316P, RHOT1P1, CYP4F35P, TERF1P2, FEM1AP2, LONRF2P1, CXADRP3, GRAMD4P7, RNU6-1021P, OR4K7P, OR4K8P, SNX19P3, RNU6-1210P, MIR3156-2, FGF7P1, RN7SL662P, BNIP3P3, RNU6-721P, RNU6-120P, EXOGP1, MIR320C1, RN7SL233P, MIR133A1, MIR1-2, RNU6-1038P, RNA5SP451, RNU6ATAC20P, GATA6-AS1, RNU6-702P, RPS4XP18, RNU6-1032P, UBE2CP2, MIR4741, RN7SL745P, RPS10P27, RPL23AP77, RNU5A-6P, RNA5SP452, RNU6-435P, IMPA2, ANKRD62, CIDEA, TUBB6, AFG3L2, SLMO1, SPIRE1, PSMG2, CEP76, PTPN2, SEH1L, CEP192
chr18	44098841	47799259	M14,M2	ST8SIA5, PIAS2, KATNAL2, TCEB3CL2, TCEB3CL, RNU6-1131P, MIR4527, TCEB3C, TPMTP1, TCEB3B, RNU6-708P, RNU7-191P, RNA5SP456, MIR4743, HDHD2, MIR4744, MIR1539, SNORD58C, SNORD58A, SNORD58B, SRP72P1, SMUG1P1, IER3IP1, SCARNA17, RNA5SP457, ADAD1P2, RN7SL310P, SKOR2, SMAD2, ZBTB7C, CTIF, SMAD7, DYM, C18orf32, RPL17-C18orf32, RPL17, LIPG, ACAA2, MYO5B, CCDC11, MBD1, LOXHD1
chr18	47799259	47906557	M14,M2,M6	RNA5SP458, MBD1, CXXC1, SKA1
chr18	47906557	77960703	M14,M2	RPLP0P11, HNRNPA3P16, RPL17P46, RN7SL695P, SRSF10P1, RNU1-46P, SS18L2P2, RSL24D1P9, RPS8P3, RPL29P32, SNORA37, SNRPGP2, RPSAP57, MAP1LC3P, RNA5SP459, MIR4529, RPL21P126, WDR7-OT1, LINC-ROR, RNU6-737P, RNU6-742P, RSL24D1P11, HMGN1P30, MIR122, RPL9P31, MRPL37P1, RN7SL112P, RNU6-219P, RNU2-69P, OACYLP, RPS26P54, GLUD1P4, NFE2L3P1, RN7SL342P, SDCCAG3P1, FAM60CP, RNU6-567P, RPS3AP49, RNU4-17P, MRPS5P4, CTBP2P3, RNU6-116P, RPL30P14, RPIAP1, RPL17P44, ACTBP9, RN7SL705P, RNU6-142P, ATP5G1P6, RPL12P39, LINC00305, RNU7-146P, PRPF19P1, RNU6-1037P, MIR5011, RPL31P9, AKR1B10P2, RNU6-39P, SDHCP1, RPS2P6, RN7SL795P, HNRNPA1P11, RNA5SP460, MIR548AV, RN7SL401P, RN7SL551P, FAUP1, LINC00909, LINC00683, ARL2BPP1, RNU6-346P, RPL26P35, BDP1P, RNA5SP461, LINC01029, RNU6-655P, RBFADN, SLC25A6P4, SKA1, MAPK4, MRO, ME2, ELAC1, SMAD4, MEX3C, DCC, MBD2, POLI, STARD6, C18orf54, DYNAP, RAB27B, CCDC68, TCF4, TXNL1, WDR7, BOD1L2, ST8SIA3, ONECUT2, FECH, NARS, ATP8B1, NEDD4L, ALPK2, MALT1, ZNF532, SEC11C, GRP, RAX, CPLX4, LMAN1, CCBE1, PMAIP1, MC4R, CDH20, RNF152, PIGN, KIAA1468, TNFRSF11A, ZCCHC2, PHLPP1, BCL2, KDSR, VPS4B, SERPINB5, SERPINB12, SERPINB13, SERPINB4, SERPINB11, SERPINB3, SERPINB7, SERPINB2, SERPINB10, HMSD, SERPINB8, CDH7, CDH19, DSEL, TMX3, CCDC102B, DOK6, CD226, RTTN, SOCS6, GTSCR1, CBLN2, NETO1, FBXO15, TIMM21, CYB5A, C18orf63, FAM69C, CNDP2, CNDP1, ZNF407, ZADH2, TSHZ1, SMIM21, ZNF516, LINC00908, ZNF236, MBP, GALR1, SALL3, ATP9B, NFATC1, CTDP1, KCNG2, PQLC1, HSBP1L1, TXNL4A, RBFA, ADNP2, PARD6G
chr18	77960703	78005397	M2	PARD6G
chr19	110678	1031130	M9	OR4F8P, CICP19, LINC01002, RNU6-1076P, VN2R11P, RNA5SP462, RPS2P52, MIR4745, MIR3187, RNU6-9, RNU6-2, OR4F17, PPAP2C, MIER2, THEG, C2CD4C, SHC2, ODF3L2, MADCAM1, TPGS1, CDC34, GZMM, BSG, HCN2, POLRMT, FGF22, RNF126, FSTL3, PRSS57, PALM, MISP, PTBP1, AZU1, PRTN3, ELANE, CFD, MED16, R3HDM4, KISS1R, ARID3A, WDR18, GRIN3B, TMEM259, CNN2
chr19	1031130	1425917	M6,M9	HMGB2P1, CIRBP-AS1, RPS15P9, CNN2, ABCA7, HMHA1, POLR2E, GPX4, SBNO2, STK11, C19orf26, ATP5D, MIDN, CIRBP, C19orf24, MUM1, EFNA2, NDUFS7, GAMT, DAZAP1
chr19	1425917	5679381	M9	RN7SL477P, RNU6-1223P, MIR1909, CSNK1G2-AS1, RN7SL226P, MIR1227, MIR4321, SPPL2B, RNU6-993P, TCEB1P28, RN7SL866P, CACTIN-AS1, FTLP5, RN7SL202P, MIR637, SNORD37, RN7SL84P, MIR4746, RN7SL528P, RN7SL121P, MIR7-3HG, MIR7-3, UHRF1, MIR4747, RPL32P34, RN7SL626P, TINCR, SNRPEP4, DAZAP1, RPS15, APC2, C19orf25, PCSK4, REEP6, ADAMTSL5, PLK5, MEX3D, MBD3, UQCR11, TCF3, ONECUT3, ATP8B3, REXO1, KLF16, ABHD17A, SCAMP4, ADAT3, CSNK1G2, BTBD2, MKNK2, MOB3A, IZUMO4, AP3D1, DOT1L, PLEKHJ1, SF3A2, AMH, JSRP1, OAZ1, C19orf35, LINGO3, LSM7, TMPRSS9, TIMM13, LMNB2, GADD45B, GNG7, DIRAS1, SLC39A3, SGTA, THOP1, ZNF554, ZNF555, ZNF556, ZNF57, ZNF77, TLE6, TLE2, AES, GNA11, GNA15, S1PR4, NCLN, CELF5, NFIC, C19orf77, DOHH, FZR1, MFSD12, C19orf71, HMG20B, GIPC3, TBXA2R, CACTIN, PIP5K1C, TJP3, APBA3, MRPL54, RAX2, MATK, ZFR2, ATCAY, NMRK2, DAPK3, EEF2, PIAS4, ZBTB7A, MAP2K2, CREB3L3, SIRT6, ANKRD24, EBI3, CCDC94, SHD, TMIGD2, FSD1, STAP2, MPND, SH3GL1, CHAF1A, UBXN6, PLIN4, PLIN5, LRG1, SEMA6B, TNFAIP8L1, C19orf10, DPP9, FEM1A, TICAM1, PLIN3, ARRDC5, KDM4B, PTPRS, ZNRF4, SAFB2, SAFB, RPL36, C19orf70
chr19	5679381	5727316	M6,M9	RPL36, C19orf70, HSD11B1L, LONP1, CATSPERD
chr19	5727316	6731251	M9	MIR3940, RPL7P50, CATSPERD, PRR22, DUS3L, NRTN, FUT6, FUT3, FUT5, NDUFA11, VMAC, CAPS, RANBP3, RFX2, ACSBG2, MLLT1, ACER1, CLPP, ALKBH7, PSPN, GTF2F1, KHSRP, SLC25A41, SLC25A23, CRB3, DENND1C, TUBB4A, TNFSF9, CD70, TNFSF14, C3, GPR108
chr19	6731251	6744637	M6,M9	GPR108, TRIP10
chr19	6744637	7960554	M9	PCP2, STXBP2, RETN, C19orf59, TRAPPC5, FCER2, CLEC4G, CD209, CLEC4M, EVI5L, LRRC8E, EMR4P, RPL21P129, CLEC4GP1, EXOSC3P2, RNA5SP463, TRIP10, SH2D3A, VAV1, EMR1, MBD3L5, MBD3L4, MBD3L2, MBD3L3, ZNF557, INSR, ARHGEF18, PEX11G, C19orf45, ZNF358, MCOLN1, PNPLA6, CAMSAP3, XAB2, PET100
chr19	7960554	8117969	M6,M9	LRRC8E, MAP2K7, TGFBR3L, SNAPC2, CTXN1, TIMM44, ELAVL1, CCL25, RN7SL115P
chr19	8117969	8923850	M9	CCL25, FBN3, CERS4, CD320, NDUFA7, RPS28, KANK3, ANGPTL4, RAB11B, MARCH2, HNRNPM, PRAM1, ZNF414, MYO1F, ADAMTS10, ACTL9, OR2Z1, ZNF558, RAB11B-AS1, MIR4999, RPL23AP78
chr19	8923850	8976259	M14,M9	ZNF558, MBD3L1, MUC16
chr19	8976259	8993372	M1,M14,M9	MUC16
chr19	8993372	8996320	M1,M14,M16,M9	MUC16
chr19	8996320	9027281	M1,M14,M16,M17,M9	MUC16
chr19	9027281	9028396	M14,M16,M9	MUC16
chr19	9028396	9768747	M14,M9	MUC16, OR1M1, OR7G2, OR7G1, OR7G3, ZNF317, OR7D2, OR7D4, OR7E24, ZNF699, ZNF559, ZNF559-ZNF177, ZNF177, ZNF266, ZNF560, ZNF426, ZNF121, ZNF561, C19orf82, ZNF562, OR1M4P, TCEB1P29, OR7E16P, OR7E25P, OR7D1P, OR7E18P, OR7E19P, OR7H1P
chr19	9768747	10078022	M9	ZNF562, ZNF812, ZNF846, FBXL12, UBL5, PIN1, OLFM2, COL5A3, RPS4XP22, UBE2L4, RN7SL94P
chr19	10078022	10116907	M1,M9	COL5A3

chr19	10116907	10678093	M9	COL5A3, RDH8, C19orf66, ANGPTL6, PPAN-P2RY11, PPAN, P2RY11, EIF3G, DNMT1, S1PR2, MRPL4, ICAM1, ICAM4, ICAM5, ZGLP1, FDX1L, RAVER1, ICAM3, TYK2, CDC37, PDE4A, KEAP1, S1PR5, ATG4D, KRI1, CDKN2D, C3P1, MIR5589, SNORD105, SNORD105B, MIR4322, MIR1181, RNU7-140P, MIR1238
chr19	36227659	36269979	M6	KMT2B, IGFLR1, U2AF1L4, PSENEN, LIN37, HSPB6, C19orf55, ARHGAP33
chr19	50357949	50391032	M6	PTOV1, PNKP, AKT1S1, TBC1D17
chr19	54600299	54710236	M6	OSCAR, NDUFA3, TFPT, PRPF31, CNOT3, LENG1, TMC4, MBOAT7, TSEN34, RPS9
chr19	56109102	56203271	M6	FIZ1, ZNF524, ZNF865, ZNF784, ZNF580, ZNF581, CCDC106, U2AF2, EPN1
chr19	58874081	59061743	M6	RNA5SP473, MIR4754, RNU6-1337P, RN7SL693P, RN7SL525P, ZNF497, ZNF837, RPS5, ZNF584, ZNF132, ZNF324B, ZNF324, ZNF446, SLC27A5, ZBTB45, TRIM28
chr19	59061743	59094187	M16,M6	CENPBD1P1, TRIM28, CHMP2A, UBE2M, MZF1
chr19	59094187	59095763	M16	CENPBD1P1
chr2	45439	283175	M17	FAM110C, SH3YL1, ACP1, FAM150B
chr2	27579736	27663543	M6	GTF3C2, EIF2B4, SNX17, ZNF513, PPM1G, NRBP1, FTH1P3
chr2	95426674	95541487	M17	SNX18P14, ANKRD20A8P, RNU6-1320P, TEKT4
chr2	137852442	151344180	M16	YWHAEP5, RN7SKP286, YY1P2, AHCYP4, MRPS18BP2, RN7SL283P, MTND2P19, MTND1P27, RNU6-904P, MTND6P11, MTND5P24, MTND4P22, MTND3P9, ZEB2-AS1, TEX41, RPL6P5, RNU7-2P, RPL17P12, PABPC1P2, RNU6-692P, RNU6-715P, RNA5SP106, RNU6-1275P, RNU2-9P, UBBP3, TXNP5, FAM8A3P, RNU6-601P, RNA5SP105, THSD7B, HNMT, SPOPL, NXPH2, LRP1B, KYNU, ARHGAP15, GTDC1, ZEB2, ACVR2A, ORC4, MBD5, EPC2, KIF5C, LYPD6B, LYPD6, MMADHC, RND3
chr2	179392172	179400708	M17	TTN-AS1, TTN
chr2	179400708	179406008	M17,M2	TTN-AS1, TTN
chr2	179406008	180036960	M16,M17,M2	TTN-AS1, RNU7-104P, RPS6P2, CCDC141, SESTD1, TTN
chr2	180036960	182756737	M16,M17	MIR1258, FTH1P20, MIR4437, RNU6ATAC19P, SESTD1, ZNF385B, CWC22, UBE2E3, ITGA4, CERKL, NEUROD1, SSFA2
chr2	182756737	183731150	M14,M16,M17	KRT18P29, RNA5SP113, RN7SL267P, RPL31P15, SSFA2, PPP1R1C, PDE1A, DNAJC10, FRZB
chr2	183731150	187694453	M16,M17	DUSP19, NUP35, ZNF804A, FSIP2, RNU6-1122P, KRT8P10, LIN28AP1, CACYBPP2, MIR548AE1, RPL21P32, RPL23AP35, MED28P3, DPRXP1, ZC3H15, ITGAV, FAM171B, ZSWIM2, FRZB, NCKAP1
chr2	187694453	189248552	M11,M16,M17	RN7SKP42, RNU6-989P, GAPDHP59, LINC01090, ST13P2, RNA5SP114, MIR561, ZSWIM2, CALCRL, TFPI, GULP1
chr2	189248552	189851865	M11,M14,M16,M17	MIR1245A, GULP1, DIRC1, COL3A1
chr2	189851865	189852806	M11,M14,M16	COL3A1
chr2	189852806	189852860	M11,M14,M16,M17	COL3A1
chr2	189852860	189853315	M11,M14,M17	COL3A1
chr2	189853315	189975062	M11,M14,M16,M17	COL3A1, COL5A2
chr2	189975062	190044605	M11,M16,M17	MIR3129, COL5A2
chr2	190044605	190315694	M11,M16	KRT18P19, COL5A2, WDR75
chr2	190315694	190316621	M11	WDR75
chr2	209103794	215919388	M16	IDH1-AS1, RNA5SP117, HSPA8P6, CRYGFP, MEAF6P1, PKP4P1, RNA5SP118, SNAI1P1, RNA5SP119, MTND2P23, MIR548F2, PCED1CP, MIR4776-1, MIR4776-2, MIR4438, RPL5P8, VWC2L-IT1, ENSAP3, IDH1, PIKFYVE, PTH2R, MAP2, UNC80, RPE, KANSL1L, ACADL, MYL1, LANCL1, CPS1, ERBB4, IKZF2, SPAG16, VWC2L, BARD1, ABCA12
chr2	227872733	227886763	M17	COL4A4
chr2	227886763	228167826	M1,M17	COL4A4, COL4A3
chr2	228167826	228172635	M1	COL4A3
chr20	5585047	25062537	M14	SDAD1P2, FAT1P1, RPS11P1, PGAM3P, LINC00687, RN7SKP111, PA2G4P2, ISM1-AS1, GAPDHP2, RNU6-278P, RPS3P1, AIMP1P1, RN7SL864P, RNU6-228P, MACROD2-IT1, RPS10P2, MACROD2-AS1, RNU6-1159P, RNU6-115P, RNA5SP475, ENSAP1, PPIAP17, RPLP0P1, RNU6-27P, RNU1-131P, DYNLT3P1, RPS27AP2, RN7SKP69, RNU6-192P, SNORD17, PTMAP3, RPL15P1, RNU7-137P, RN7SL14P, RNU2-56P, RN7SKP74, RNY4P11, LINC00851, GCNT1P1, RNA5SP476, MIR3192, RPL21P3, RPS19P1, LINC00493, RN7SL638P, RNU6ATAC34P, DUXAP7, EEF1A1P34, LINC00652, RPL17P1, RN7SL690P, EIF4E2P1, RN7SL607P, MRPS11P1, LINC00237, RPL24P2, PLK1S1, RNA5SP477, RPS15AP1, ZNF877P, RN7SKP140, GSTM3P1, NKX2-2-AS1, SLC25A6P1, RPL41P1, LINC00261, KRT18P3, LINC00656, RNA5SP478, RNA5SP479, CST12P, CST13P, CST9LP1, CST2P1, CSTP1, POM121L3P, RNU1-23P, GAPDHP53, RNU6-1257P, GPCPD1, C20orf196, CHGB, TRMT6, MCM8, CRLS1, LRRN4, FERMT1, BMP2, HAO1, RNU1-55P, RN7SL498P, TARDBPP1, CASC20, RN7SL547P, TMX4, PHKBP1, PLCB1-IT1, RNU105B, SNAP25-AS1, HIGD1AP15, RPL23AP6, PLCB1, PLCB4, LAMP5, PAK7, ANKEF1, SNAP25, MKKS, SLX4IP, JAG1, C20orf187, BTBD3, SPTLC3, ISM1, TASP1, ESF1, NDUFAF5, SEL1L2, MACROD2, FLRT3, KIF16B, SNRPB2, OTOR, PCSK2, BFSP1, DSTN, RRBP1, BANF2, SNX5, OVOL2, MGME1, PET117, CSRP2BP, ZNF133, DZANK1, POLR3F, RBBP9, SEC23B, DTD1, C20orf78, SCP2D1, SLC24A3, RIN2, NAA20, CRNKL1, C20orf26, INSM1, RALGAPA2, XRN2, NKX2-4, NKX2-2, PAX1, FOXA2, SSTR4, THBD, CD93, NXT1, GZF1, NAPB, CSTL1, CST11, CST8, CST9L, CST9, CST3, CST4, CST1, CST2, CST5, GGTLC1, SYNDIG1, CST7, APMAP, ACSS1, VSX1
chr20	44566140	44642837	M6	FTLP1, PCIF1, ZNF335, MMP9
chr20	55748310	55949662	M14	BMP7, SPO11, RAE1, MTRNR2L3, MIR4325, MTND1P9
chr20	55949662	58644992	M11,M14	CTSZ, TUBB1, ATP5E, SLMO2, ZNF831, EDN3, PHACTR3, RAE1, RBM38, SYCP2, CTCFL, FAM217B, PPP1R3D, CDH26, C20orf197, PCK1, ZBP1, PMEPA1, C20orf85, ANKRD60, PPP4R1L, RAB22A, HMGB1P1, MIR4532, APCDD1L-AS1, PIEZO1P2, MIR296, MIR298, GNAS-AS1, MRPS16P2, VAPB, PIEZO1P1, RNU7-141P, APCDD1L, STX16, STX16-NPEPL1, NPEPL1, GNAS, NELFCD
chr20	58644992	60640866	M11	C20orf197, CDH4, TAF4, MIR646, MTCO2P1, MIR4533, MIR548AG2, MIR1257
chr22	16256331	16256504	M1,M2	POTEH
chr22	16256504	16449314	M1,M14,M2	POTEH, OR11H1, POTEH-AS1, RNU6-816P, MED15P7, NF1P6, NEK2P2, ARHGAP42P3
chr22	16449314	17128147	M1,M2	OR11H1, CCT8L2, YME1L1P1, BNIP3P2, ZNF72P, ABCD1P4, PABPC1P9, SLC9B1P4, ACTR3BP6, CHEK2P4, KCNMB3P1, FABP5P11, TPTEP1, SLC25A15P5
chr22	17128147	17626007	M2	MTND1P17, IGKV1OR22-5, IGKV2OR22-4, IGKV2OR22-3, IGKV3OR22-2, IGKV1OR22-1, CECR7, XKR3, GAB4, IL17RA, CECR6, CECR5, TPTEP1, PARP4P3, ANKRD62P1-PARP4P3, ANKRD62P1, VWFP1, HSFY1P1, GPM6BP3, ZNF402P
chr22	18644556	24342268	M2	IGLL1, C22orf43, RGL4, ZNF70, VPREB3, GGT3P, C22orf15, CHCHD10, BCRP7, MMP11, DGCR5, DGCR9, DGCR10, CA15P1, SMARCB1, DGCR11, TSSK1A, KRT18P62, DERL3, RN7SL168P, MIR4761, MIR185, MIR3618, MIR1306, LINC00896, MIR1286, SLC2A11, MIF, GSTT2B, DDTL, DDT, GSTT2, PI4KAP1, RN7SKP131, CA15P2, PPP1R26P2, PPP1R26P3, RNU6-225P, KLHL22-IT1, RN7SL812P, KRT18P5, SMPD4P1, SLC9A3P2, ABHD17AP4, POM121L4P, BCRP5, TMEM191A, RN7SL389P, THAP7-AS1, TUBA3FP, MIR649, P2RX6P, TUBA3GP, BCRP2, FAM230B, POM121L8P, BCRP6, FAM230C, PPP1R26P5, RN7SKP63, TMEM191C, PI4KAP2, RN7SKP221, MIR301B, MIR130B, RN7SL280P, RNA5SP493, PRAMENP, IGLVI-70, IGLVI-68, IGLV10-67, IGLVIV-66-1, IGLVV-66, IGLVIV-65, IGLVIV-64, IGLVI-63, IGLV1-62, ABHD17AP5, SOCS2P2, IGLVIV-59, IGLVV-58, BMP6P1, IGLVI-56, IGLVIV-53, TOP3BP1, BMS1P20, IGLVI-42, IGLVVII-41-1, IGLV1-41, ASH2LP1, IGLVI-38, IGLV7-35, IGLV2-34, IGLV3-31, IGLV3-30, BCRP4, POM121L1P, IGLV3-29, IGLV2-28, IGLV3-26, IGLVVI-25-1, IGLV3-24, IGLVVI-22-1, IGLVI-20, IGLV3-17, IGLV3-15, IGLV3-13, MIR650, IGLV3-7, IGLV3-6, IGLV2-5, IGLV3-4, IGLV3-2, IGLC4, IGLC5, IGLC6, BCRP8, RN7SL263P, FBXW4P1, CES5AP1, ZDHHC8P1, GUSBP11, ASLP1, RN7SL268P, GSTTP1, USP18, DGCR6, PRODH, DGCR2, DGCR14, TSSK2, GSC2, SLC25A1, CLTCL1, HIRA, C22orf39, MRPL40, UFD1L, CDC45, CLDN5, SEPT5, GP1BB, TBX1, GNB1L, C22orf29, TXNRD2, COMT, ARVCF, TANGO2, DGCR8, TRMT2A, RANBP1, ZDHHC8, RTN4R, DGCR6L, GGTLC3, RIMBP3, FAM230A, USP41, ZNF74, SCARF2, KLHL22, MED15, PI4KA, SERPIND1, SNAP29, CRKL, AIFM3, LZTR1, THAP7, P2RX6, SLC7A4, POM121L7, GGT2, RIMBP3B, HIC2, RIMBP3C, UBE2L3, YDJC, CCDC116, SDF2L1, PPIL2, YPEL1, MAPK1, PPM1F, TOP3B, IGLV4-69, IGLV8-61, IGLV4-60, IGLV6-57, IGLV11-55, IGLV10-54, VPREB1, IGLV5-52, IGLV1-51, IGLV1-50, IGLV9-49, IGLV5-48, IGLV1-47, IGLV7-46, IGLV5-45, IGLV1-44, IGLV7-43, IGLV1-40, IGLV5-37, IGLV1-36, ZNF280B, ZNF280A, PRAME, IGLV2-33, IGLV3-32, GGTLC2, IGLV3-27, IGLV3-25, IGLV2-23, IGLV3-22, IGLV3-21, IGLV3-19, IGLV2-18, IGLV3-16, IGLV2-14, IGLV3-12, IGLV2-11, IGLV3-10, IGLV3-9, IGLV2-8, IGLV4-3, IGLV3-1, IGLL5, IGLJ1, IGLC1, IGLJ2, IGLC2, IGLJ3, IGLC3, IGLJ4, IGLJ5, IGLJ6, IGLJ7, IGLC7, RTDR1, GNAZ, RAB36, BCR
chr22	24342268	24394849	M1,M2	GSTT1, GSTTP1, MTND1P13, EIF4EBP1P2, GSTTP2
chr22	24394849	25255807	M2	CABIN1, SUSD2, GGT5, SPECC1L, SPECC1L-ADORA2A, ADORA2A, UPB1, GUCD1, SNRPD3, GGT1, FAM211B, PIWIL3, SGSM1, GSTTP2, POM121L9P, BCRP1, ADORA2A-AS1, BCRP3, POM121L10P, ARL5AP4, CRIP1P4

chr22	27003852	29838443	M2	CRYBB1, CRYBA4, ISCA2P1, MN1, MIAT, PITPNB, RNU6-1066P, TTC28, TTC28-AS1, MIR3199-1, MIR3199-2, RN7SL757P, RN7SL162P, ZNRF3-IT1, ZNRF3-AS1, RNU6-810P, RNU6-1219P, SNORD125, RFPL1S, CHEK2, HSCB, CCDC117, XBP1, ZNRF3, C22orf31, KREMEN1, EMID1, RHBDD3, EWSR1, GAS2L1, RASL10A, AP1B1, RFPL1
chr22	40750251	41863618	M2	COX6B1P3, RPL4P6, GAPDHP37, JTBP1, MIR4766, RNU6-379P, MIR1281, RNU6-375P, LRRC37A14P, RNU6-495P, ADSL, SGSM3, MKL1, MCHR1, SLC25A17, ST13, XPNPEP3, DNAJB7, RBX1, EP300, L3MBTL2, CHADL, RANGAP1, ZC3H7B, TEF, TOB2, PHF5A
chr22	41969658	42377840	M2	RNU6-476P, HMGN2P10, RNU6ATAC22P, MIR33A, MIR378I, LINC00634, CSDC2, PMM1, DESI1, XRCC6, NHP2L1, C22orf46, MEI1, CCDC134, SREBF2, SHISA8, TNFRSF13C, CENPM, SEPT3
chr22	43019794	50436171	M2	RPL5P34, GOLGA2P4, EFCAB6-AS1, HMGN2P9, MRPS18CP6, KRT18P23, LINC00207, LINC00229, ANP32BP2, MIR1249, RNU6-1161P, MIR4762, LINC00899, MIR3619, MIRLET7A3, MIR4763, MIRLET7B, LINC00898, MIR3201, MIR4535, RPL35P8, MIR3667, RN7SKP252, RPL5P35, CYB5R3, ATP5L2, A4GALT, ARFGAP3, PACSIN2, TTLL1, BIK, MCAT, TSPO, TTLL12, SCUBE1, MPPED1, EFCAB6, SULT4A1, PNPLA5, PNPLA3, SAMM50, PARVB, PARVG, KIAA1644, LDOC1L, PRR5, PRR5-ARHGAP8, ARHGAP8, PHF21B, NUP50, KIAA0930, UPK3A, FAM118A, SMC1B, RIBC2, FBLN1, ATXN10, WNT7B, C22orf26, PPARA, CDPF1, PKDREJ, TTC38, GTSE1, TRMU, CELSR1, GRAMD4, CERK, TBC1D22A, FAM19A5, C22orf34, BRD1, ZBED4, ALG12, CRELD2, PIM3, IL17REL
chr22	50943071	50970468	M6	LMF2, NCAPH2, SCO2, TYMP, ODF3B
chr22	51064363	51238064	M2	RNU6-409P, RPL23AP82, ARSA, SHANK3, ACR, RABL2B
chr3	17208258	31665353	M16	PDCL3P3, RAD23BP1, RNU6-138P, MIR4791, HSPA8P18, RNU4-85P, MIR3135A, SGOL1-AS1, RNY4P22, RNU6-822P, RNU6-815P, VENTXP7, ZNF385D-AS1, ZNF385D-AS2, HMGB1P5, RANP7, SALL4P5, RPL24P7, UBE2E2-AS1, RNU6-922P, RNU6-788P, UBE2E1-AS1, LINC00691, NPM1P23, RPL31P20, THRB-IT1, THRB-AS1, MIR4792, EIF3KP2, RN7SL216P, RNA5SP125, CFL1P7, RNA5SP126, MIR4442, CRIP1P2, TAF9BP1, RPEP2, HMGB3P12, VENTXP4, MINOS1P3, RNU6-342P, RN7SL859P, RPS20P15, RNU1-96P, FAM96AP1, LINC00693, MESTP4, RBMS3-AS3, RPS12P5, RBMS3-AS2, RBMS3-AS1, CNN2P6, RNA5SP127, THRAP3P1, TBC1D5, SATB1, KCNH8, EFHB, RAB5A, PP2D1, KAT2B, SGOL1, ZNF385D, UBE2E2, UBE2E1, NKIRAS1, RPL15, NR1D2, THRB, RARB, TOP2B, NGLY1, OXSM, LINC00692, LRRC3B, NEK10, SLC4A7, EOMES, CMC1, AZI2, ZCWPW2, RBMS3, TGFBR2, GADL1, STT3B
chr3	48604339	48623679	M1	MIR711, COL7A1
chr3	75790760	89531282	M16	RNU6-386P, VDAC1P7, RNU6-217P, RN7SL647P, RN7SKP61, MRPS17P3, RN7SL751P, MIR3923, HMGB1P38, OSBPL9P1, HNRNPA3P8, RNU2-28P, SETP6, RPL7AP23, CYP51A1P1, SRRM1P2, LINC00971, MIR5688, CADM2-AS2, CADM2-AS1, PRKRIRP2, RNU6-1129P, RN7SKP284, PPATP1, MIR4795, KRT8P25, APOOP2, PSMC1P6, RNU6-873P, RNU6ATAC6P, CBX5P1, ABCF2P1, NDUFA5P5, NARG2P2, GAPDHP50, ZNF717, ROBO2, ROBO1, GBE1, CADM2, VGLL3, CHMP2B, POU1F1, HTR1F, CGGBP1, ZNF654, C3orf38, EPHA3
chr3	111261040	113442873	M14	WDR52, SPICE1, SIDT1, KIAA2018, NAA50, PLCXD2-AS1, RFKP2, MIR567, OR7E100P, WDR52-AS1, SIDT1-AS1, MIR4446, RN7SL767P, CD96, ZBED2, PLCXD2, PHLDB2, ABHD10, TAGLN3, TMPRSS7, C3orf52, GCSAM, SLC9C1, CD200, BTLA, ATG3, SLC35A5, CCDC80, CD200R1L, CD200R1, GTPBP8, C3orf17, BOC
chr3	160956517	164700030	M16	NMD3, SPTSSB, OTOL1, SI, EEF1GP4, PSMC1P7, TOMM22P6, CT64, MIR1263, MIR720
chr3	164700030	167090738	M16,M17	SI, SLITRK3, BCHE, ZBBX, MTND4P17, MTND3P7, RN7SKP298, PSAT1P4, CBX1P5
chr3	167090738	169550971	M16	EGFEM1P, RNU2-20P, MIR551B, RPL21P43, RNU6-637P, TERC, ZBBX, SERPINI2, WDR49, PDCD10, SERPINI1, GOLIM4, MECOM, ACTRT3, MYNN, LRRC34, LRRIQ4, HMGN1P8, MEMO1P3, HMGN2P26
chr4	79766081	83292737	M16	RN7SL127P, LINC01088, LINC00989, OR7E94P, PCAT4, RNU5A-2P, COX5BP1, NPM1P41, HNRNPA3P13, RNU6-499P, BIN2P1, BMP2K, PAQR3, NAA11, GK2, ANTXR2, PRDM8, FGF5, C4orf22, BMP3, PRKG2, RASGEF1B, HNRNPD
chr4	113825640	190948296	M14	RAB33B, SETD7, TMEM154, TIGD4, ADAD1, MIR3688-2, MIR3688-1, ARFIP1, POU4F2, RPS14P7, MTHFD2P4, TOMM22P4, FREM3, MGST2, RPL35AP12, RN7SKP105, ANP32C, RNU6-284P, RNU6-668P, TTC29, GRIA2, TRIM60P14, MAML3, FAM218BP, GYPE, NACA3P, FHDC1, SCOC, GYPB, RNU4-87P, GK3P, MIR578, HADHAP1, SCGB1D5P, TRIM2, FAM198B, IL2, EDNRA, MND1, IL21, RNA5SP170, TMEM144, CLGN, RNA5SP171, RN7SL776P, PHBP14, RN7SKP188, BTF3L4P4, BBS12, RNU6-1336P, RPL9P16, RNU6-853P, RNY4P17, RPL6P12, PTGES3P3, FGF2, APOBEC3AP1, HSP90AA6P, RNU6ATAC13P, TMEM184C, KIAA0922, RN7SL253P, MIR548T, NUDT6, RPL5P11, RNU6-1096P, ELMOD2, PRMT10, GYPA, SPATA5, HAND2-AS1, RXFP1, UCP1, TLR2, ARHGAP10, RNF175, TBC1D9, SFRP2, HHIP, DCHS2, RNF150, MORF4, RANP6, RN7SL184P, RNU1-138P, MIR4276, TSEN2P1, ADAM20P2, MIR577, CIR1P2, RN7SL808P, MRPS33P3, RPF2P2, PGAM4P2, KRT18P21, EIF3KP3, TTC39CP1, SPRY1, PLRG1, ANKRD50, TRMT112P1, CUL4AP1, ACTN4P1, ANAPC10, RNU6-119P, NR3C2, FAT4, ZNF330, MARK2P4, RPSAP35, NT5C3AP1, FKBP4P1, RN7SKP136, SNHG8, RNA5SP172, LINC01099, RNU1-45P, RNA5SP173, LINC00290, NDUFS5P5, CEP170P1, RN7SKP13, ABCE1, INTU, IL15, DCLK2, RNU6-1054P, CICP16, MIR1305, RNU2-34P, RN7SKP67, KLHL2P1, RNU4-33P, FAM92A1P2, RNU6-1217P, WWC2-AS2, RNU7-158P, WWC2-AS1, VTI1BP2, RNU6-479P, RNU6-335P, RNU6-1053P, RN7SL28P, FGB, MIR3945, LINC01093, MIR4455, C4orf46, ETFDH, SLC25A31, INPP4B, RNU4-64P, ORAOV1P1, F11-AS1, OTUD4, LTV1P1, PPID, HSPA4L, SLC25A5P6, RNU6-1055P, RNU6-550P, SETP12, MRPS36P2, RN7SKP137, RNU6-948P, TUBB4BP5, RN7SL335P, FNIP2, ADAM20P3, LRBA, RNU6-173P, NARG2P1, LINC01060, C4orf45, RNU7-192P, RAPGEF2, HSP90AA4P, IL21-AS1, CETN4P, RNU1-51P, LINC01091, MLLT10P2, TUBB7P, RPL21P50, RNA5SP174, RNA5SP175, DUX4L9, TECRP2, NUPL1P1, TMEM248P1, PLK4, FSTL5, RBM48P1, FGA, RNU6-583P, FOSL1P1, ZSWIM5P3, FGG, RNY5P4, EEF1GP8, SMAD1, PGBD4P4, GAPDHP56, RNU6-224P, RN7SL205P, RPL7AP28, ELL2P2, USP38, NAF1, NPY1R, NPY5R, TKTL2, TMA16, R3HDM2P1, GAB1, PES1P1, MAB21L2, MMAA, RNU6-569P, KRT18P54, TARS2P1, RPS3A, LINC00613, RNU1-89P, MARCH1, TERF1P3, SERF1AP1, STMN1P2, RPS23P2, MFSD8, C4orf51, LINC00616, SLC7A11-AS1, LINC00498, LINC00499, ZNF827, LRAT, LINC00500, RNU6-531P, RN7SL382P, PPP1R14BP3, RN7SL311P, RNU6-506P, RBM46, RNU6-1074P, RNU6-1214P, SH3D19, NPY2R, TRIM61, SMARCA5, FAM218A, TRIM60, MAP9, TMEM192, C4orf29, KLHL2, FTH1P24, RN7SKP237, H3F3AP6, RN7SKP253, LSM6, RN7SL152P, TNRC18P1, RPL5P13, LSM3P4, MIR3139, SMARCA5-AS1, GUSBP5, SLC10A7, GUCY1A3, MSMO1, PRSS48, LARP1B, FAM160A1, GUCY1B3, CPE, PET112, TLL1, ASIC5, TDO2, SPOCK3, CTSO, FBXW7, PDGFC, KRT18P51, HHIP-AS1, HSPD1P5, PGRMC2, RN7SKP235, RPS23P4, RTN3P1, NMNAT1P4, SMAD1-AS2, SMAD1-AS1, LINC01095, RNU1-44P, MIR548G, GTF2F2P1, RNA5SP165, MIR4799, RN7SL254P, RNA5SP166, ATP5LP4, RNU7-197P, RNA5SP167, RNU6-1230P, RNU7-194P, ZBTB8OSP1, RNA5SP168, AK4P6, SNORD73A, RNU6-1282P, RN7SKP35, RNA5SP169, GLRB, JADE1, RN7SL446P, MIR3140, MIR4453, RNU6-1196P, ANXA2P1, RN7SL419P, RNU6-1285P, RNU2-66P, RNU2-44P, ANXA10, YWHAEP4, MTND6P17, MTND5P9, MTND4P8, MTND3P3, SCLT1, MTATP6P9, MTND2P33, MTND1P22, FTH1P21, RPPH1-3P, RNU6-582P, DDX60, NUDT19P5, C4orf33, RNU4-79P, RNU6-128P, FABP5P12, DDX60L, PCDH10, PABPC4L, PCDH18, SLC7A11, PALLD, CCRN4L, ELF2, MGARP, CBR4, NDUFC1, SH3RF1, NAA15, NEK1, CLCN3, C4orf27, MFAP3L, AADAT, GALNTL6, GALNT7, HMGB2, SAP30, SCRG1, HAND2, FBXO8, CEP44, HPGD, GLRA3, ADAM29, GPM6A, WDR17, SPATA4, ASB5, SPCS3, VEGFC, NEIL3, AGA, LINC01098, TENM3, DCTD, WWC2, ANK2, CLDN22, CLDN24, CDKN2AIP, ING2, RWDD4, TRAPPC11, STOX2, ENPP6, IRF2, CASP3, PRIMPOL, CENPU, ACSL1, CAMK2D, HELT, SLC25A4, KIAA1430, SNX25, LRP2BP, ANKRD37, UFSP2, C4orf47, CCDC110, ARSJ, UGT8, NDST4, PDLIM3, MTRNR2L13, TRAM1L1, NDST3, SORBS2, PRSS12, METTL14, SEC24D, TLR3, SYNPO2, FAM149A, MYOZ2, USP53, CYP4V2, C4orf3, FABP2, PDE5A, KLKB1, F11, MTNR1A, FAT1, ZFP42, TRIML2, TRIML1, MAD2L1, FRG1, PRDM5, FRG2, NDNF, TNIP3, QRFPR, ANXA5, TMEM155, EXOSC9, CCNA2, BBS7, TRPC3, KIAA1109
chr5	140372	1572074	M2	SDHAP3, PLEKHG4B, LRRC14B, CCDC127, SDHA, PDCD6, AHRR, C5orf55, EXOC3, SLC9A3, CEP72, TPPP, ZDHHC11, ZDHHC11B, BRD9, TRIP13, NKD2, SLC12A7, SLC6A19, SLC6A18, TERT, CLPTM1L, SLC6A3, LPCAT1, MIR4456, SPCS2P3, MIR4635, MIR4457
chr5	1572074	1599942	M16,M2	SDHAP3
chr5	1599942	1632959	M16	
chr5	1632959	9630463	M16,M2	MIR4277, LINC01019, LINC01020, RN7SKP73, ALG3P1, HMGB3P3, LINC01018, RN7SKP79, RNA5SP176, RNU1-76P, MIR4458HG, MTND6P2, MIR4636, SNHG18, SNORD123, MRPL36, NDUFS6, IRX4, IRX2, C5orf38, IRX1, ADAMTS16, KIAA0947, MED10, UBE2QL1, NSUN2, SRD5A1, PAPD7, ADCY2, C5orf49, MTRR, FASTKD3, SEMA5A, TAS2R1
chr5	9630463	13721108	M16	CMBL, MARCH6, RNA5SP177, ROPN1L, ANKRD33B, DAP, CTNND2, DNAH5, ROPN1L-AS1, RPL30P7, ANKRD33B-AS1, RNU6-429P, RNU6-679P, CT49, RPS23P5, TAS2R1, FAM173B, CCT5
chr5	13721108	14769300	M16,M2	DNAH5, CCT6P2, EEF1A1P13, TRIO, FAM105A, FAM105B, ANKH
chr5	14769300	31429581	M16	BASP1, CDH18, CDH12, PRDM9, C5orf17, CDH10, CDH9, CDH6, DROSHA, RBBP4P1, MIR4637, UQCRBP3, HNRNPKP5, SEPHS2P1, MARK2P5, MIR887, RNA5SP178, NACAP6, RNA5SP179, RNU6-660P, RNA5SP180, DCAF13P2, RNU6-1003P, RN7SKP133, FTH1P10, RPL36AP21, RN7SL58P, UBE2V1P12, RPL32P14, HSPD1P15, GUSBP1, HSPD1P1, PMCHL1, GCNT1P2, RN7SL572P, RNU6-374P, MSNP1, RNU4-43P, RNU6-738P, LINC01021, RNU6-909P, LSP1P3, SUCLG2P4, UBL5P1, RN7SKP207, RPL19P11, ANKH, FBXL7, MARCH11, ZNF622, FAM134B, MYO10
chr5	31429581	37227175	M16,M2	RNU6-363P, RNU6-358P, RNU6-760P, RPL5P14, TPT1P5, MIR4279, RNU6-1079P, RNU6-378P, MIR579, RNU6-923P, RPL21P54, RNU7-130P, MIR580, RNU6-1305P, NADK2-AS1, RNA5SP181, KRT18P31, RPS4XP6, OFD1P17, ADAMTS12, SPEF2, RXFP3, SLC45A2, AMACR, C1QTNF3, IL7R, CAPSL, RAI14, UGT3A1, DROSHA, UGT3A2, LMBRD2, SKP2, TTC23L, NADK2, RAD1, C5orf22, BRIX1, RANBP3L, PDZD2, DNAJC21, SLC1A3, AGXT2, GOLPH3, NIPBL, MTMR12, PRLR, C5orf42, ZFR, SUB1, NPR3, TARS
chr5	37227175	37443340	M2	RN7SL37P, RNU7-75P, NUP155, WDR70, C5orf42
chr5	37443340	44310632	M16,M2	RNU6-1190P, RNU6-484P, GDNF-AS1, EGFLAM-AS4, EGFLAM-AS3, EGFLAM-AS2, EGFLAM-AS1, LIFR-AS1, MIR3650, AIG1P1, GOLGA5P1, CCDC11P1, INTS6P1, GCSHP1, LINC00603, KRT18P56, LINC00604, RNU1-150P, SNORD72, RNU7-161P, OXCT1-AS1, MTHFD2P6, SERBP1P6, EEF1A1P19, NNT-AS1, RPL29P12, RNU6-381P, WDR70, GDNF, EGFLAM, LIFR, OSMR, RICTOR, FYB, C9, DAB2, PTGER4, TTC33, PRKAA1, RPL37, CARD6, C7, MROH2B, C6, PLCXD3, OXCT1, C5orf51, FBXO4, GHR, CCDC152, SEPP1, ANXA2R, ZNF131, NIM1K, HMGCS1, CCL28, C5orf28, C5orf34, PAIP1, NNT, FGF10
chr5	44310632	45696220	M16	FGF10-AS1, RN7SL383P, FGF10, MRPS30, HCN1

chr5	49692032	54592177	M16,M2	RNU6-480P, RNU6-1296P, HMGB1P47, RNA5SP182, KATNBL1P4, RPS17P11, ASS1P9, RNU6-272P, MIR581, RN7SL801P, MIR4459, LINC01033, RPL37P25, GZMAP1, MIR449A, MIR449B, MIR449C, EMB, PARP8, ISL1, ITGA1, PELO, ITGA2, MOCS2, FST, NDUFS4, ARL15, HSPB3, SNX18, ESM1, GZMK, GZMA, CDC20B, GPX8, MCIDAS, CCNO, DHX29
chr5	54592177	56160761	M2	MIR5687, RNF138P1, AK4P2, RNA5SP183, HNRNPH1P3, RNU6-299P, RPL17P22, RNA5SP184, RNA5SP185, PSMC1P4, RNU6ATAC2P, HMGN1P17, DHX29, SKIV2L2, PPAP2A, SLC38A9, DDX4, IL31RA, IL6ST, ANKRD55, MAP3K1
chr5	63256278	64769779	M2	RN7SL169P, RNU6-294P, MRPL49P1, RPEP1, HTR1A, RNF180, RGS7BP, FAM159B, SREK1IP1, CWC27, ADAMTS6
chr5	65321675	67591152	M11	MAST4-IT1, MAST4-AS1, RNU6-1232P, EEF1B2P2, ERBB2IP, SREK1, MAST4, CD180, PIK3R1
chr5	77684660	80724452	M2	FAM151B, ANKRD34B, DHFR, MTRNR2L2, MSH3, SCAMP1, RASGRF2, HMGB1P21, RNY3P1, TRMT112P2, KRT18P45, RBMX2P5, HNRNPA1P12, RNU6-211P, RPL7P24, DBIP2, CKMT2-AS1, CKMT2, ZCCHC9, ACOT12, SSBP2, LHFPL2, ARSB, DMGDH, BHMT2, BHMT, JMY, HOMER1, PAPD4, CMYA5, MTX3, THBS4, SERINC5, SPZ1, ZFYVE16
chr5	80724452	81605975	M14,M2	SHFM1P1, ATG10-IT1, PPIAP11, ATG10-AS1, RN7SL378P, SSBP2, ATG10, RPS23, ATP6AP1L
chr5	81605975	100222304	M14,M16,M2	PTP4A1P4, NBPF22P, MIR3607, RNU6-804P, RN7SKP34, MIR4280, RN7SL629P, RNU6-606P, RNU6-727P, ERAP2, TMEM161B-AS1, RNA5SP187, RPS3AP22, LINC00461, MEF2C-AS1, ARRDC3, LNPEP, MIR3660, RNU4-90P, LUCAT1, RAB5CP2, LIX1, PCBP2P3, RIOK2, LDHBP3, CCT7P2, POLD2P1, NR2F1-AS1, NR2F1, RGMB, CHD1, MIR2277, NPM1P27, FAM172A, MTND5P12, MTND6P3, RPL7P18, RNU6-308P, HSPD1P11, POU5F2, KIAA0825, FABP5P5, MIR583, RNU6-524P, SETP22, RNU1-73P, YTHDF1P1, ANKRD32, KRT8P32, MRPS35P2, CTBP2P4, RGMB-AS1, CSNK1A1P3, RNU6-402P, GUSBP8, EEF1A1P20, RPL5P16, MTND5P10, FAM174A, MTATP6P2, RNU6-1119P, ST8SIA4, RN7SKP62, MIR548P, ST13P12, SCARNA18, COQ10BP2, FTH1P9, VCAN-AS1, RN7SKP295, RNU6-620P, RNU4-11P, RNU6-448P, MCTP1, RBBP4P6, RPL5P17, RPS2P25, FAM81B, TTC37, ARSK, GPR150, RFESD, ATP6AP1L, TMEM167A, SPATA9, XRCC4, RHOBTB3, VCAN, GLRX, C5orf27, ELL2, PCSK1, HAPLN1, EDIL3, COX7C, RASA1, CAST, CCNH, TMEM161B, MEF2C, CETN3, MBLAC2, POLR3G, LYSMD3, GPR98, ERAP1
chr5	100222304	100231357	M14,M16	ST8SIA4
chr5	100231357	104435486	M14,M16,M2	ST8SIA4, RN7SL802P, OR7H2P, RNA5SP188, RN7SKP68, LINC00492, LINC00491, EIF3KP1, SLCO4C1, SLCO6A1, PAM, RNU1-140P, RN7SL255P, RNU6-334P, RAB9BP1, GIN1, PPIP5K2, C5orf30, NUDT12
chr5	104435486	106714833	M16,M2	RAB9BP1, RNA5SP189, PSMC1P5, EFNA5
chr5	106714833	108717404	M14,M16,M2	RN7SL782P, RN7SKP122, LINC01023, RNU6-47P, GJA1P1, EFNA5, FBXL17, FER, PJA2
chr5	108717404	109159423	M14,M16	KRT18P42, RN7SKP230, PJA2, MAN2A1
chr5	109159423	109202987	M14,M16,M2	MAN2A1
chr5	109202987	109756134	M16,M2	PGAM5P1, MAN2A1, TMEM232
chr5	109756134	125696151	M14,M16,M2	SLC25A46, HMGB1P22, RN7SKP117, GRAMD3, RPSAP37, TSLP, WDR36, CAMK4, STARD4, NREP, EPB41L4A, EPB41L4A-AS2, APC, MIR548F3, STARD4-AS1, HMGN1P13, RN7SKP57, HMGN1P14, NREP-AS1, EPB41L4A-AS1, SRP19, SNORA13, HMGB3P16, CBX3P3, RNU6-482P, XBP1P1, RNU4ATAC13P, RN7SKP89, REEP5, TRIM36-IT1, HMGN1P15, AK3P4, CCT5P1, RNU2-49P, RNU6-644P, HMGN2P27, RPS17P2, RPL35AP15, LINC00992, ZRSR1, RPL7L1P4, RNU7-34P, DCP2, PTMAP2, RNU6-373P, MCC, LAMTOR3P2, RNU6-701P, MIR5706, RN7SL174P, RNA5SP190, FABP5P6, RNU6-718P, TSSK1B, YTHDC2, RNU4-69P, ARGFXP1, RN7SL689P, RN7SL711P, KRT8P33, HMGB3P17, KRT18P16, HMGB1P29, LINC01170, KCNN2, TRIM36, PGGT1B, CCDC112, FEM1C, TICAM2, TMED7-TICAM2, TMED7, CDO1, ATG12, AP3S1, TMEM232, ARL14EPL, COMMD10, SEMA6A, DTWD2, DMXL1, TNFAIP8, HSD17B4, FAM170A, PRR16, FTMT, SRFBP1, LOX, ZNF474, SNCAIP, SNX2, SNX24, PPIC, PRDM6, CEP120, CSNK1G3, ZNF608
chr5	125696151	125816399	M14,M2	GRAMD3
chr5	125816399	126386996	M14	GRAMD3, RNU6-963P, HSPE1P10, RNU6-752P, ALDH7A1, PHAX, C5orf48, LMNB1, MARCH3, C5orf63
chr5	126386996	126705601	M14,M2	MRPS5P3, C5orf63, MEGF10
chr5	126705601	128990126	M14,M16,M2	HNRNPKP1, KDELC1P1, MIR4633, MIR4460, ADAMTS19-AS1, MEGF10, PRRC1, CTXN3, SLC12A2, FBN2, SLC27A6, ISOC1, ADAMTS19
chr5	128990126	129074376	M14,M2	ADAMTS19
chr5	129074376	131978731	M14	RNU6ATAC10P, RNA5SP191, HSPA8P4, RNU7-53P, ARL2BPP4, PDLIM4, P4HA2-AS1, MIR3936, SLC22A4, SLC22A5, C5orf56, IRF1, IL5, RAD50, ADAMTS19, KIAA1024L, CHSY3, HINT1, LYRM7, CDC42SE2, RAPGEF6, FNIP1, ACSL6, IL3, CSF2, P4HA2
chr5	149980641	150418786	M2	ZNF300P1, SYNPO, MYOZ3, RBM22, DCTN4, SMIM3, IRGM, ZNF300, GPX3, TNIP1
chr5	156693090	162864810	M2	RNU6-390P, RNU6-260P, RNU2-48P, MARK2P11, RNU4ATAC2P, GAPDHP40, RN7SL295P, MIR146A, MIR3142, GLRXP3, RNU6-164P, ARL2BPP5, MRP63P6, CYFIP2, FNDC9, ADAM19, NIPAL4, SOX30, C5orf52, THG1L, LSM11, CLINT1, EBF1, RNF145, UBLCP1, IL12B, ADRA1B, TTC1, PWWP2A, FABP6, CCNJL, C1QTNF2, C5orf54, SLU7, PTTG1, ATP10B, GABRB2, GABRA6, GABRA1, GABRG2, CCNG1
chr5	171849419	176831305	M2	MIR5003, SNORA74B, CDC42P5, RPL7AP33, RNA5SP200, RNU6-500P, GAPDHP71, SUMO2P6, HIGD1AP3, MIR4634, NIFKP2, ARL2BPP6, RN7SKP148, RNU6-226P, OR1X1P, MIR1271, RN7SL684P, MIR4281, ZNF346-IT1, RN7SL562P, SH3PXD2B, NEURL1B, DUSP1, ERGIC1, RPL26L1, ATP6V0E1, CREBRF, BNIP1, NKX2-5, STC2, BOD1, CPEB4, C5orf47, MSX2, DRD1, SFXN1, HRH2, CPLX2, THOC3, FAM153B, SIMC1, KIAA1191, ARL10, NOP16, HIGD2A, CLTB, FAF2, RNF44, CDHR2, GPRIN1, SNCB, EIF4E1B, TSPAN17, UNC5A, HK3, UIMC1, ZNF346, FGFR4, NSD1, RAB24, MXD3, PRELID1, LMAN2, RGS14, SLC34A1, PFN3, F12, GRK6
chr5	176831305	176959077	M2,M6	PRR7-AS1, F12, GRK6, PRR7, DBN1, PDLIM7, DOK3, DDX41, FAM193B
chr5	176959077	178039546	M2	OR1X5P, SUDS3P1, GMCL1P1, RN7SL646P, FAM193B, TMED9, B4GALT7, FAM153A, PROP1, FAM153C, N4BP3, RMND5B, NHP2, HNRNPAB, PHYKPL, COL23A1, CLK4
chr6	292100	292330	M11,M2,M9	DUSP22
chr6	292330	28499672	M11,M14,M2,M9	ACOT13, KDM1B, TDGF1P4, RNA5SP202, PSMC1P11, RMRPP2, MIR3691, HNRNPA1P37, RN7SL221P, BTN2A2, PKMP5, C6orf62, MIR5683, LY86-AS1, SNAPC5P1, CNN3P1, BTF3P7, RN7SL554P, HNRNPLP1, PIP5K1P1, HULC, RNU6ATAC21P, TFAP2A-AS1, LINC00518, MIR5689, MRPL48P1, RNA5SP203, ELOVL2-AS1, RNU1-64P, PRKRIRP5, DEK, SUMO2P12, RN7SKP293, RPL15P3, RNU1-11P, GFOD1-AS1, RPS4XP7, RN7SKP204, RNU7-91P, MRPL35P1, RNU7-133P, LINC01108, RNU6-793P, RN7SL332P, JARID2-AS1, RNU6-522P, RNU6-645P, ARPC3P5, MIR4639, MRPL42P2, RNU6-1114P, RPL7P26, SUMO2P13, RNU6-190P, RNA5SP204, RNU6-263P, MIR548A1, RPL5P20, RNA5SP205, KRT18P38, TBC1D7, UQCRFS1P3, RNU6-801P, RN7SL128P, E2F3-IT1, RNU6-141P, RNU6-150P, CASC15, RN7SKP240, CASC14, RNU6-1060P, SPTLC1P2, HNRNPA1P58, RNU6-391P, KRT8P43, PPIAP29, ASS1P1, RN7SL334P, CMAHP, DSP, RNY5P5, KATNBL1P5, RNU6-987P, PRELID1P2, HIST1H2BPS1, HIST1H2APS1, HIST1H2APS2, HIST1H1PS2, LARP1P1, HIST1H1PS1, HIST1H2APS3, HIST1H2APS4, HIST1H3PS1, RNU6-1259P, BTN2A3P, RNU6-502P, HCG11, VN1R14P, GUSBP2, GMNN, LINC00240, TRNAI2, VN1R12P, VN1R13P, VN1R11P, RNU2-62P, MIR3143, VN1R10P, BTN3A1, ZNF204P, MCFD2P1, HNRNPA1P1, RNU6-471P, TRNAI6, RSL24D1P1, HIST1H4PS1, HIST1H2BPS2, RNU7-26P, OR2W6P, RPLP2P1, OR2W4P, IQCB2P, OR2W2P, OR2B7P, OR2B8P, OR1F12, ZSCAN12P1, ZSCAN16-AS1, ZNF192P1, ZNF192P2, TOB2P1, RNU2-45P, COX11P1, OR2E1P, RNF144B, C6orf229, ID4, FAM65B, MBOAT1, SNRNP48, BTN3A3, GFOD1, BMP6, TXNDC5, SIRT5, BLOC1S5-TXNDC5, BLOC1S5, E2F3, EEF1E1-BLOC1S5, EEF1E1, CDKAL1, SLC35B3, NOL7, LRRC16A, BTN2A1, BTN1A1, RANBP9, HMGN4, ABT1, SOX4, OFCC1, ZNF322, MCUR1, SCGN, PRL, HDGFL1, NRSN1, HIST1H2AA, HIST1H2BA, SLC17A4, HIST1H2BJ, RNF182, HIST1H2AG, TFAP2A, HIST1H2BK, HIST1H4I, HIST1H2AH, PRSS16, SLC17A1, DCDC2, CD83, JARID2, KAAG1, MRS2, GCNT2, SLC17A3, DTNBP1, GPLD1, POM121L2, ZNF391, ZNF184, HIST1H2BL, HIST1H2AI, GCNT6, HIST1H3H, HIST1H2AJ, HIST1H2BM, HIST1H4J, HIST1H4K, C6orf52, HIST1H2BN, HIST1H2AK, HIST1H2AL, HIST1H1B, HIST1H3I, HIST1H4L, SLC17A2, HIST1H3J, HIST1H2AM, HIST1H2BO, OR2B2, OR2B6, TRIM38, HIST1H1A, PAK1IP1, HIST1H3A, HIST1H4A, ZNF165, HIST1H4B, ZSCAN16, HIST1H3B, HIST1H2AB, HIST1H2BB, TMEM14C, HIST1H3C, HIST1H1C, MYLIP, GMPR, HFE, ALDH5A1, TMEM14B, ATXN1, ZKSCAN8, ZSCAN9, ZKSCAN4, SYCP2L, STMND1, KIAA0319, NKAPL, PGBD1, RBM24, ZSCAN31, CAP2, TDP2, ZKSCAN3, ZSCAN12, ZSCAN23, HIST1H4C, GPX6, HIST1H1T, HIST1H2BC, HIST1H2AC, GPX5, HIST1H1E, HIST1H2BD, HIST1H2BE, HIST1H4D, HIST1H3D, MAK, HIST1H2AD, RN7SL352P, GMDS-AS1, HMGN2P28, MIR4645, FAM8A1, HIST1H2BF, LINC01011, HIST1H4E, HTATSF1P2, FAM136BP, SERPINB8P1, RNA5SP201, TUBB2BP1, HIST1H2BG, GCM2, NUP153, ELOVL2, HIST1H2AE, HIST1H3E, HIST1H1D, SMIM13, HIST1H4F, ERVFRD-1, NEDD9, HIST1H4G, HIST1H3F, HIST1H2BH, HIST1H3G, HIST1H2BI, HIST1H4H, KIF13A, BTN3A2, TMEM170B, ADTRP, DUSP22, HIVEP1, EDN1, PHACTR1, IRF4, NHLRC1, TPMT, EXOC2, HUS1B, FOXQ1, FOXF2, FOXC1, GMDS, C6orf195, MYLK4, WRNIP1, SERPINB1, SERPINB9, SERPINB6, NQO2, RIPK1, BPHL, TUBB2A, TUBB2B, PSMG4, SLC22A23, PXDC1, FAM50B, PRPF4B, FAM217A, C6orf201, ECI2, CDYL, RPP40, PPP1R3G, LYRM4, FARS2, NRN1, F13A1, LY86, RREB1, SSR1, CAGE1, RIOK1
chr6	28499672	28547196	M11,M14,M9	GPX5, SCAND3
chr6	28547196	28555112	M11,M14	SCAND3
chr6	28555112	28870779	M14	LINC00533, RPSAP2, RPL13P, ZNF90P2, HCG14, SCAND3, TRIM27

chr6	28870779	31911957	M11,M14	LY6G5B, LY6G5C, ABHD16A, RNU6-930P, KRT18P1, RN7SL471P, HCG15, HCG16, OR2AD1P, OR2P1P, OR2N1P, OR2J4P, OR2H4P, OR2G1P, OR2U1P, OR2U2P, OR2B4P, DDX6P1, UBDP1, MAS1LP1, RPS17P1, LINC01015, GPR53P, OR2I1P, OR2H5P, TMEM183AP1, SNORD32B, RPL13AP, SUMO2P1, ZDHHC20P1, HCG4P11, HLA-F-AS1, RPL23AP1, MICE, HCG9P5, IFITM4P, HLA-V, HCG4, HLA-P, RPL7AP7, MICG, HCG4P8, MICF, HCG4P7, HLA-H, HLA-T, DDX39BP1, MCCD1P1, HCG4B, HLA-K, HLA-U, HCG4P5, HLA-W, MICD, HCG9, DDX39BP2, MCCD1P2, ZNRD1-AS1, HCG4P3, HLA-J, ETF1P1, TRIM31-AS1, PAIP1P1, HCG17, TRIM26BP, HLA-L, HCG18, HLA-N, HCG19P, UBQLN1P1, MICC, TMPOP1, SUCLA2P1, RANP1, MIR877, PTMAP1, RPL7P4, MDC1-AS1, RN7SL353P, HCG20, LINC00243, RN7SKP186, DDR1-AS1, MIR4640, RN7SL175P, HCG21, HCG22, RNU6-1133P, POLR2LP, PSORS1C3, USP8P1, RPL3P2, WASF5P, DHFRP2, RNU6-283P, FGFR3P1, ZDHHC20P2, HLA-S, HCP5, LINC01149, PPIAP9, RPL15P4, SNORD117, SNORD84, DDX39B-AS1, UQCRHP1, SNORA38, C6orf47-AS1, MIR4646, RNU6-850P, SAPCD1-AS1, SNORD48, SNORD52, EHMT2-AS1, LY6G6F, LY6G6E, LY6G6D, C6orf25, LY6G6C, DDAH2, CLIC1, MSH5, MSH5-SAPCD1, SAPCD1, VWA7, VARS, LSM2, HSPA1L, HSPA1A, HSPA1B, C6orf48, NEU1, TRIM27, C6orf100, ZNF311, OR2W1, OR2B3, OR2J1, OR2J3, SLC44A4, OR2J2, OR14J1, OR5V1, EHMT2, OR12D3, OR12D2, OR12D1, OR11A1, OR10C1, OR2H1, MAS1L, UBD, GABBR1, C2, OR2H2, MOG, ZBTB12, CFB, ZFP57, HLA-F, HLA-G, HLA-A, ZNRD1, PPP1R11, RNF39, TRIM31, TRIM40, TRIM10, TRIM15, TRIM26, TRIM39, TRIM39-RPP21, RPP21, HLA-E, GNL1, PRR3, ABCF1, PPP1R10, MRPS18B, ATAT1, C6orf136, DHX16, PPP1R18, NRM, MDC1, TUBB, FLOT1, IER3, DDR1, GTF2H4, VARS2, SFTA2, DPCR1, MUC21, MUC22, C6orf15, PSORS1C1, CDSN, PSORS1C2, CCHCR1, TCF19, POU5F1, HCG27, HLA-C, HLA-B, MICA, MICB, MCCD1, ATP6V1G2-DDX39B, DDX39B, ATP6V1G2, NFKBIL1, LTA, TNF, LTB, LST1, NCR3, AIF1, PRRC2A, BAG6, APOM, C6orf47, GPANK1, CSNK2B
chr6	31911957	31928972	M11,M14,M6	MIR1236, C2, CFB, NELFE, SKIV2L
chr6	31928972	32011844	M11,M14,M6,M9	C4A-AS1, CYP21A1P, TNXA, STK19P, C4B-AS1, SKIV2L, DXO, STK19, C4A, C4B, CYP21A2, TNXB
chr6	32011844	32131017	M11,M14,M9	RNA5SP206, TNXB, ATF6B, FKBPL, PRRT1, PPT2, PPT2-EGFL8
chr6	32131017	32160307	M11,M14,M6,M9	PPT2, PPT2-EGFL8, EGFL8, AGPAT1, RNF5, AGER, PBX2, GPSM3
chr6	32160307	32160817	M14,M6,M9	GPSM3
chr6	32160817	32163613	M11,M14,M6,M9	GPSM3, NOTCH4
chr6	32163613	33170121	M11,M14,M9	HNRNPA1P2, RNU6-603P, HCG23, HLA-DRB9, RNU1-61P, HLA-DRB6, HLA-DQB1-AS1, MTCO3P1, HLA-DQB3, MIR3135B, TAPSAR1, PPP1R2P1, HLA-Z, BRD2-IT1, RPL32P1, HLA-DPA2, COL11A2P1, HLA-DPB2, HLA-DPA3, HCG24, RNY4P10, NOTCH4, C6orf10, BTNL2, HLA-DRA, HLA-DRB5, HLA-DRB1, HLA-DQA1, HLA-DQB1, HLA-DQA2, HLA-DQB2, HLA-DOB, TAP2, PSMB8, PSMB9, TAP1, HLA-DMB, HLA-DMA, BRD2, HLA-DOA, HLA-DPA1, HLA-DPB1, COL11A2, RXRB, SLC39A7
chr6	33170121	33180498	M11,M14,M6,M9	HSD17B8, RING1, MIR219-1, SLC39A7
chr6	33180498	33262753	M14,M6,M9	RING1, VPS52, ZNF70P1, HCG25, RPS18, B3GALT4, WDR46, PFDN6, RGL2
chr6	33262753	33382143	M11,M14,M6,M9	MYL8P, LYPLA2P1, RPL35AP4, RPL12P1, PFDN6, RGL2, TAPBP, ZBTB22, DAXX, KIFC1, PHF1
chr6	33382143	33382253	M11,M14,M6	PHF1
chr6	33382253	33400522	M11,M14,M6,M9	PHF1, CUTA, SYNGAP1
chr6	33400522	37414066	M11,M14,M9	SNRPC, PNPLA1, C6orf222, UHRF1BP1, ETV7, TAF11, PXT1, KCTD20, ANKS1A, STK38, SRSF3, CDKN1A, TCP11, RAB44, CPNE5, PPIL1, MIR5004, RN7SL26P, LINC00336, MIR3934, LINC01016, MIR1275, KRT18P9, CYCSP55, RPL35P2, RPL7P25, RN7SL200P, HSPE1P11, MIR5690, DPRXP2, RN7SL502P, RN7SL748P, MIR3925, RNU1-88P, LAP3P2, RPL12P2, RN7SL273P, C6orf89, PI16, MTCH1, SCUBE3, ZNF76, FGD2, DEF6, COX6A1P2, PIM1, TMEM217, PPARD, FANCE, RPL10A, TBC1D22B, TEAD3, RNF8, TULP1, CMTR1, FKBP5, ARMC12, CLPSL2, CLPSL1, SYNGAP1, ZBTB9, BAK1, CLPS, LHFPL5, SRPK1, GGNBP1, ITPR3, SLC26A8, UQCC2, IP6K3, LEMD2, MAPK14, MLN, GRM4, MAPK13, HMGA1, C6orf1, NUDT3, RPS10-NUDT3, RPS10, PACSIN1, SPDEF, BRPF3, C6orf106
chr6	37414066	38783433	M11,M14,M2,M9	MIR4462, RN7SL285P, RNU1-87P, BTBD9-AS1, RN7SL465P, CMTR1, CCDC167, MDGA1, ZFAND3, BTBD9, GLO1, DNAH8
chr6	38783433	38790613	M14,M2,M9	DNAH8
chr6	38790613	39835619	M11,M14,M2,M9	KIF6, DAAM2, ANKRD18EP, E2F4P1, RNU1-54P, DNAH8, GLP1R, SAYSD1, KCNK5, KCNK17, KCNK16
chr6	39835619	39836598	M11,M14,M2	DAAM2
chr6	39836598	41753278	M11,M14,M2,M9	ADCY10P1, TREML3P, RNA5SP207, TREML5P, RNU6-643P, MIR4641, NPM1P51, DAAM2, MOCS1, TUBBP9, TDRG1, LINC00951, RNU6-250P, LRFN2, UNC5CL, OARD1, TSPO2, APOBEC2, NFYA, TREML1, TREM2, TREML2, TREML4, TREM1, NCR2, FOXP4, MDFI, TFEB, PGC, FRS3, PRICKLE4
chr6	41753278	41774749	M11,M14,M9	FRS3, PRICKLE4, TOMM6, USP49
chr6	41774749	41839301	M14,M9	USP49
chr6	41839301	42626472	M11,M14,M9	RNU6-761P, USP49, MED20, BYSL, CCND3, TAF8, C6orf132, GUCA1A, GUCA1B, MRPS10, TRERF1, UBR2
chr6	42626472	42627493	M11,M9	UBR2
chr6	42627493	43469344	M11,M14,M9	DNPH1, RNU6-890P, ATP6V0CP3, RPL24P4, RN7SL403P, RPS2P28, RNU6-1113P, TTBK1, SLC22A7, CRIP3, ZNF318, ABCC10, DLK2, TJAP1, UBR2, PRPH2, TBCC, GLTSCR1L, RPL7L1, C6orf226, PTCRA, CNPY3, GNMT, PEX6, PPP2R5D, MEA1, KLHDC3, RRP36, CUL7, KLC4, MRPL2, PTK7, SRF, CUL9
chr6	43469344	43492290	M11,M14,M6,M9	TJAP1, LRRC73, POLR1C, YIPF3, XPO5
chr6	43492290	44151441	M11,M14,M9	POLR1C, XPO5, POLH, GTPBP2, MAD2L1BP, RSPH9, MRPS18A, VEGFA, C6orf223, MRPL14, TMEM63B, CAPN11
chr6	44151441	44230347	M11,M14,M2,M9	MIR4647, HSP90AB1, SLC35B2, NFKBIE, CAPN11, SLC29A1
chr6	44230347	44233121	M11,M2,M9	NFKBIE
chr6	44233121	44344210	M11,M14,M2,M9	NFKBIE, TMEM151B, TCTE1, AARS2, SPATS1
chr6	44344210	44344771	M14,M2,M9	SPATS1
chr6	44344771	46108959	M11,M14,M2,M9	MIR4642, NUDT19P4, MIR586, RNU6-515P, RNU6-754P, SPATS1, CDC5L, SUPT3H, RUNX2, CLIC5, ENPP4
chr6	46108959	46111012	M11,M14,M2	ENPP4
chr6	46111012	53761626	M11,M14,M2,M9	DEFB133, DEFB114, DEFB113, DEFB110, DEFB112, ACTG1P9, TFAP2D, RN7SKP116, RNU1-105P, HNRNPA3P4, RBMXP1, RNU7-65P, EEF1A1P42, CYP2AC1P, RPS17P5, FTH1P5, RN7SL580P, MIR206, MIR133BHG, MIR133B, SLC25A20P1, TRAM2-AS1, GSTA7P, GSTA6P, RN7SK, RN7SL244P, RNU6-464P, SOD1P1, RNU1-136P, HMGB1P20, MIR5685, RPS16P5, RPL31P28, RPL31P33, RN7SKP256, NANOGP3, TFAP2B, PKHD1, IL17A, IL17F, MCM3, PAQR8, EFHC1, ENPP4, ENPP5, TRAM2, RCAN2, CYP39A1, SLC25A27, TMEM14A, GSTA2, GSTA1, TDRD6, PLA2G7, GSTA5, GSTA3, ANKRD66, MEP1A, GSTA4, GPR116, ICK, GPR110, FBXO9, TNFRSF21, CD2AP, GCM1, ELOVL5, GPR111, GPR115, OPN5, GCLC, KLHL31, PTCHD4, MUT, LRRC1, CENPQ, GLYATL3, C6orf141, RHAG, CRISP2, CRISP3, PGK2, CRISP1
chr6	53761626	55966296	M11,M14,M16,M2,M9	MLIP-IT1, ERHP2, MLIP-AS1, CLNS1AP1, KRASP1, RNU6-1023P, NPM1P36, LRRC1, MLIP, TINAG, FAM83B, HCRTR2, GFRAL, HMGCLL1, BMP5, COL21A1
chr6	55966296	55988886	M11,M16,M2,M9	COL21A1
chr6	55988886	58287724	M11,M14,M16,M2,M9	RCC2P7, RPL17P26, RNU6-626P, OSTCP6, FTH1P15, PRIM2, MIR548U, GAPDHP41, RBBP4P3, GUSBP4, POM121L14P, LINC00680, COL21A1, DST, BEND6, KIAA1586, ZNF451, BAG2, RAB23
chr6	58287724	62389864	M14	LINC00680, GAPDHP15, RBBP4P4, MTRNR2L9
chr6	62389864	62995931	M1,M14,M16,M2,M9	KHDRBS2
chr6	62995931	66115260	M1,M16,M2,M9	SPTLC1P3, EEF1B2P5, RPL7AP34, RPL9P18, GCNT1P4, HNRNPDP2, KHDRBS2, FKBP1C, LGSN, PTP4A1, PHF3, EYS
chr6	66115260	73023375	M1,M16,M9	SLC25A51P1, ADH5P4, NUFIP1P, RNU7-66P, RNA5SP208, RNU6-280P, NPM1P37, GAPDHP42, RPL37P15, RNU7-48P, SLC25A6P6, LYPLA1P3, RNU6-411P, LINC00472, MIR30C2, MIR30A, KRT19P1, RNU4-66P, EYS, BAI3, LMBRD1, COL19A1, COL9A1, FAM135A, C6orf57, SMAP1, B3GAT2, OGFRL1, RIMS1
chr6	73023375	73843364	M16,M9	KCNQ5-IT1, MIR4282, KNOP1P4, PGAM1P10, RIMS1, KCNQ5
chr6	73843364	73935175	M16	KCNQ5-AS1, KHDC1P1, KCNQ5, KHDC1L
chr6	74502348	74519706	M17	CD109
chr6	74519706	75875495	M16,M17	TXNP7, CD109, COL12A1
chr6	75875495	75902071	M16	COL12A1
chr6	76640669	84574047	M16	RNU6-248P, RNU6-261P, RPS6P7, HMGN3-AS1, DBIP1, GAPDHP63, RPL35AP18, AK4P5, RPL17P25, RNA5SP210, RNU6-130P, LAP3P1, IMPG1, HTR1B, MEI4, IRAK1BP1, PHIP, HMGN3, LCA5, SH3BGRL2, ELOVL4, TTK, BCKDHB, FAM46A, IBTK, TPBG, UBE3D, DOPEY1, PGM3, RWDD2A, ME1, PRSS35, SNAP91, RIPPLY2, CYB5R4

chr6	88852209	90024869	M14	ACTBP8, RNU2-61P, RN7SL336P, TUBB3P1, CNR1, RNGTT, PNRC1, SRSF12, PM20D2, GABRR1, GABRR2
chr6	144999541	147830523	M16	GRM1, RAB32, ADGB, STXBP5, RNU1-33P, FUNDC2P3, RNA5SP222, RNU6-734P, KATNBL1P6, STXBP5-AS1, YAP1P1, SAMD5, UTRN, EPM2A, FBXO30, SHPRH
chr7	192968	4862185	M2	CYP2W1, C7orf50, GPR146, GPER1, ZFAND2A, UNCX, MICALL2, INTS1, MAFK, TMEM184A, PSMG3, ELFN1, MAD1L1, FTSJ2, NUDT1, SNX8, EIF3B, CHST12, LFNG, BRAT1, IQCE, TTYH3, AMZ1, GNA12, CARD11, SDK1, FOXK1, AP5Z1, RADIL, FAM20C, PDGFA, PRKAR1B, HEATR2, SUN1, GET4, ADAP1, COX19, MIR339, PSMG3-AS1, TFAMP1, MIR4655, GRIFIN, MIR4648, RN7SKP130, CYP3A54P, MIR4656
chr7	6785718	6854394	M2	RSPH10B2, CCZ1B, PMS2CL
chr7	6854394	7222245	M16,M2	CCZ1B, C1GALT1, OR7E39P, UNC93B2, OR7E136P, OR7E59P, ALG1L5P, FAM86LP, MIR3683
chr7	7222245	7222348	M1,M11,M16,M2,M9	C1GALT1
chr7	7222348	7572543	M1,M11,M14,M16,M2,M9	C1GALT1, COL28A1
chr7	7572543	20445794	M11,M14,M16,M2,M9	MIR1302-6, RN7SKP266, NPM1P13, MIR3146, RPL21P75, MACC1-AS1, COL28A1, MIOS, RPA3, RPA3-AS1, GLCCI1, ICA1, NXPH1, NDUFA4, PHF14, THSD7A, TMEM106B, VWDE, SCIN, ARL4A, ETV1, DGKB, AGMO, MEOX2, ISPD, SOSTDC1, LRRC72, ANKMY2, BZW2, TSPAN13, AGR2, AGR3, AHR, SNX13, PRPS1L1, HDAC9, TWIST1, FERD3L, TWISTNB, TMEM196, MACC1, ITGB8, RPL23AP51, RNU6-534P, GAPDHP68, HSPA8P8, NPM1P11, TAS2R2P, RN7SKP228, RBMX2P4, RPL6P21, EEF1A1P26, RPL36AP26, RPL36AP29, ISPD-AS1, RAD17P1, RNMTL1P2
chr7	20445794	20739758	M14,M16,M2,M9	EEF1A1P27, ITGB8, ABCB5
chr7	20739758	23180563	M14,M2,M9	RPL23P8, LINC01162, RPS26P30, RN7SL542P, ASS1P11, RNU1-15P, MIR1183, RNA5SP227, EEF1A1P6, SNORD93, KLHL7-AS1, AK3P3, ABCB5, SP8, SP4, DNAH11, CDCA7L, RAPGEF5, STEAP1B, IL6, TOMM7, FAM126A, KLHL7
chr7	23180563	38807198	M14,M2	EEPD1, KIAA0895, ANLN, AOAH, ELMO1, RNU7-143P, RPS2P32, FCF1P1, PCMTD1P3, TPT1P7, RNA5SP228, RNU6-1103P, SUMO2P14, TSEN15P3, MIR148A, GPR141, EPDR1, NME8, RPL7AP38, HMGB3P20, TPM3P4, NHP2P2, HOTAIRM1, SFRP4, STARD3NL, HOXA-AS2, TRGC2, TRGJ2, TRGJP2, TRGC1, HOXA-AS3, TRGJ1, TRGJP, TRGJP1, TRGV11, HOXA10-AS, TRGV10, TRGV9, MIR196B, TRGV8, HOXA11-AS, TRGV5, TRGV4, TRGV3, TRGV2, TRGV1, AMPH, HOTTIP, EVX1-AS, RPL35P4, HNRNPA1P73, EIF4HP1, PSMC1P2, RN7SL365P, RNU6-979P, JAZF1-AS1, TRIL, NANOGP4, ZNRF2P2, TMSB4XP3, FAM183B, DPY19L2P3, MIR550A3, VPS41, RPS27P16, MIR550A1, SNX2P2, SLC25A5P5, DPY19L1P1, ZNRF2P1, MIR550A2, DPY19L1P2, RP9P, RNU6-388P, RN7SL505P, RNA5SP229, RNU6-438P, NPSR1-AS1, RPL7P31, NCAPD2P1, RN7SL132P, MIR548N, DPY19L2P1, S100A11P2, RNU6-1085P, SEPT7P3, MARK2P13, AOAH-IT1, NPM1P18, MIR1200, ELMO1-AS1, RPS17P13, RNU6-565P, RPS10P14, NECAP1P1, TRGVB, TRGVA, TRGV7, TRGV6, TRGV5P, RN7SL83P, KRT8P20, KLHL7, NUPL2, GPNMB, MALSU1, IGF2BP3, TRA2A, CCDC126, FAM221A, STK31, NPY, MPP6, DFNA5, OSBPL3, CYCS, C7orf31, NPVF, NFE2L3, HNRNPA2B1, CBX3, SNX10, KIAA0087, C7orf71, SKAP2, HOXA1, HOXA2, HOXA3, HOXA4, HOXA5, HOXA6, HOXA7, HOXA9, HOXA10, HOXA11, HOXA13, EVX1, HIBADH, TAX1BP1, JAZF1, CREB5, CPVL, CHN2, PRR15, WIPF3, SCRN1, FKBP14, PLEKHA8, MTURN, ZNRF2, NOD1, GGCT, GARS, CRHR2, INMT, INMT-FAM188B, FAM188B, AQP1, GHRHR, ADCYAP1R1, NEUROD6, CCDC129, PPP1R17, PDE1C, LSM5, AVL9, KBTBD2, FKBP9, NT5C3A, RP9, BBS9, BMPER, NPSR1, DPY19L1, TBX20, HERPUD2, SEPT7
chr7	38807198	38810797	M14	VPS41
chr7	38810797	43832362	M14,M2	VPS41, POU6F2, YAE1D1, RALA, CDK13, MPLKIP, SUGCT, INHBA, GLI3, C7orf25, PSMA2, MRPL32, HECW1, POU6F2-AS2, POU6F2-AS1, LINC00265, RNU6-719P, CICP22, STK17A, COA1, RWDD4P2, RN7SL496P, INHBA-AS1, HMGN2P30, TCP1P1, HECW1-IT1, MIR3943, RNU7-35P, RNU6-575P, BLVRA
chr7	43832362	45717564	M2	TUBG1P, POLR2J4, BLVRA, RASA4CP, URGCP-MRPS24, MRPS24, URGCP, UBE2D4, SPDYE1, LINC00957, MIR4649, RNA5SP230, RNU6-1097P, DBNL, MIR4657, SNHG15, SNORA5A, SNORA5C, SNORA5B, ELK1P1, PGAM2, POLM, AEBP1, POLD2, MYL7, GCK, YKT6, CAMK2B, NUDCD3, NPC1L1, DDX56, TMED4, OGDH, ZMIZ2, PPIA, H2AFV, PURB, MYO1G, CCM2, NACAD, TBRG4, RAMP3, ADCY1
chr7	45717564	55493034	M14,M2	SEPT7P2, RNU6-241P, CICP20, RNU6-326P, RNU7-76P, FTLP15, TTC4P1, HMGN1P19, EPS15P1, MRPL42P4, LINC00525, GDI2P1, RNU6-1091P, RPL7L1P2, CICP17, ROBO2P1, RN7SL292P, HAUS6P1, RNU1-14P, RN7SKP218, RNU2-29P, SLC25A5P3, RNU6-1125P, RPL31P35, EGFR-AS1, CALM1P2, ADCY1, IGFBP1, IGFBP3, TNS3, C7orf65, HUS1, PKD1L1, C7orf69, SUN3, C7orf57, UPP1, ABCA13, VWC2, ZPBP, C7orf72, IKZF1, FIGNL1, DDC, GRB10, COBL, POM121L12, VSTM2A, SEC61G, EGFR, LANCL2
chr7	55493034	55757153	M2	CDC42P2, TUBBP6, FKBP9L, RNU6-389P, LANCL2, VOPP1
chr7	76751933	76958649	M11	CCDC146, FGL2, GSAP
chr7	76958649	77530027	M11,M2	CCDC146, GSAP, PTPN12, RSBN1L, TMEM60, PHTF2, GCNT1P5, RSBN1L-AS1
chr7	77530027	77572016	M11,M2,M9	PHTF2
chr7	77572016	86595291	M11,M16,M2,M9	PHTF2, MAGI2, GNAI1, CD36, GNAT3, SEMA3C, HGF, RPL13AP17, MAGI2-IT1, MAGI2-AS1, MAGI2-AS2, RNU6-337P, RNU6-530P, MAGI2-AS3, RNA5SP234, RNU6-849P, RN7SL869P, RN7SL35P, SNRPBP1, MIR1255B1, MTHFD2P5, RNA5SP235, RAD23BP2, RPL7P30, HMGN2P11, HNRNPA1P8, HSPA8P16, LINC00972, SOCS5P1, CACNA2D1, PCLO, SEMA3E, SEMA3A, SEMA3D, GRM3, KIAA1324L
chr7	86595291	91843205	M11,M2	TP53TG1, HNRNPA1P9, EIF4A1P13, PQLC1P1, EEF1A1P28, RNU6-274P, STEAP2-AS1, DPY19L2P4, TVP23CP1, PTP4A1P3, NIPA2P1, MIR1285-1, KIAA1324L, DMTF1, TMEM243, CROT, ABCB4, ABCB1, RUNDC3B, SLC25A40, DBF4, ADAM22, SRI, STEAP4, ZNF804B, C7orf62, STEAP1, STEAP2, C7orf63, GTPBP10, CLDN12, CDK14, FZD1, MTERF, AKAP9, CYP51A1, LRRD1, KRIT1
chr7	91843205	92861652	M11,M2,M9	TMBIM7P, RNU6-10P, RN7SL7P, KRIT1, ANKIB1, GATAD1, ERVW-1, PEX1, RBM48, FAM133B, CDK6, SAMD9, SAMD9L, HEPACAM2
chr7	92861652	93065372	M11,M16,M2,M9	CCDC132, CALCR
chr7	93065372	93536047	M11,M14,M16,M2,M9	MIR653, MIR489, MIR4652, NDUFAF4P2, CALCR, GNGT1, TFPI2
chr7	93536047	94029507	M11,M14,M16,M17,M2,M9	GNGT1, GNG11, BET1, COL1A2
chr7	94029507	94055842	M1,M11,M14,M16,M17,M2,M9	COL1A2
chr7	94055842	94139529	M11,M14,M16,M17,M2,M9	RNU6-1328P, COL1A2, CASD1
chr7	94139529	94157562	M11,M14,M16,M2,M9	CASD1
chr7	94157562	97501308	M11,M14,M2,M9	RPS3AP25, RNU6-956P, ATP5F1P2, GRPEL2P3, RN7SKP129, ARF1P1, RNU4-16P, MIR591, RNU6-532P, RNU6-364P, RNU7-188P, MARK2P10, RN7SL252P, DLX6-AS1, DLX6-AS2, HMGB3P21, RN7SKP104, CASD1, SGCE, PEG10, PPP1R9A, PON1, PON3, PON2, ASB4, PDK4, DYNC1I1, SLC25A13, SHFM1, C7orf76, DLX6, DLX5, ACN9, TAC1, ASNS
chr7	97501308	97501854	M11,M14,M9	ASNS
chr7	97501854	97601542	M14,M9	OR7E7P, MIR5692A1, MIR5692A2, MIR5692C2, OR7E38P, RPS3AP29, ASNS
chr7	97601542	97616468	M9	OCM2
chr7	99673208	99755513	M6	MIR25, MIR93, MIR106B, RPL7P60, MIR4658, ZNF3, COPS6, MCM7, AP4M1, TAF6, CNPY4, MBLAC1, LAMTOR4, C7orf43
chr7	100468038	100491329	M6	TRIP6, SRRT, UFSP1, ACHE
chr7	102312090	102389398	M14	RASA4DP
chr7	102389398	104909477	M11,M14,M2	RNU6-1136P, RN7SKP198, NFE4, CRYZP1, RPL19P12, DPY19L2P2, S100A11P1, RN7SKP86, EIF4BP6, LHFPL3-AS1, RN7SL8P, LINC01004, KMT2E-AS1, FAM185A, FBXL13, LRRC17, ARMC10, NAPEPLD, PMPCB, DNAJC2, PSMC2, SLC26A5, RELN, ORC5, LHFPL3, KMT2E, SRPK2
chr7	104909477	105099706	M14,M2	RWDD4P1, RNU6-1322P, SRPK2, PUS7
chr7	105099706	105516823	M14	YBX1P2, PUS7, RINT1, EFCAB10, ATXN7L1
chr7	105516823	105603656	M11,M14	ATXN7L1, CDHR3
chr7	105603656	107571800	M11,M14,M2	DCAF13P1, RNU6-392P, LARP1BP2, RNA5SP236, WBP1LP2, BANF1P5, SLC26A4-AS1, PIGCP2, CDHR3, SYPL1, NAMPT, CCDC71L, PIK3CG, PRKAR2B, HBP1, COG5, GPR22, DUS4L, BCAP29, SLC26A4, CBLL1, SLC26A3, DLD, LAMB1
chr7	107571800	127569382	M11,M14,M16,M2	LAMB4, NRCAM, PNPLA8, THAP5, DNAJB9, C7orf66, IMMP2L, LRRN3, DOCK4, RPL7P32, EIF3IP1, RPL3P8, DOCK4-AS1, RNA5SP237, RN7SKP187, MTND5P8, NPM1P14, MIPEPP1, RNA5SP238, MIR3666, RAC1P6, RNA5SP239, ST7-AS1, TPM3P1, ST7-AS2, ZNF277, IFRD1, MTND4P6, RNU6-581P, RNA5SP240, HMGN1P18, RNA5SP241, RNU6-517P, CYCSP19, RN7SKP277, PNPT1P2, LSMEM1, RNU7-154P, FEZF1-AS1, TMEM168, RPS26P31, LYPLA1P1, RNU6-296P, RNU6-11P, HYALP1, C7orf60, GPR85, SSU72P8, LINC00998, PPP1R3A, RNU6-102P, POT1-AS1, EEF1GP1, FOXP2, MIR592, MDFIC, TFEC, TES, CAV2, CAV1, MET, CAPZA2, ST7, ST7-OT4, WNT2, ASZ1, CFTR, CTTNBP2, NAA38, ANKRD7, KCND2, TSPAN12, ING3, CPED1, WNT16, FAM3C, PTPRZ1, AASS, FEZF1, CADPS2, RNF133, RNF148, TAS2R16, SLC13A1, IQUB, NDUFA5, ASB15, LMOD2, WASL, HYAL4, SPAM1, TMEM229A, GPR37, POT1, GRM8, ZNF800, GCC1, ARF5, FSCN3, PAX4, SND1, DLD, LAMB1
chr7	127569382	127630999	M14,M2	SND1
chr7	127630999	127732351	M11,M14,M2	MIR593, SND1, LRRC4

chr7	127732351	137790175	M11,M2	AGBL3, C7orf49, TMEM140, WDR91, STRA8, CNOT4, NUP205, C7orf73, SLC13A4, FAM180A, MTPN, LUZP6, CHRM2, PTN, DGKI, CREB3L2, AKR1D1, MIR129-1, RNU7-27P, RNU7-54P, RNU6-177P, CICP14, IMP3P2, RNA5SP242, RNA5SP243, RN7SL81P, KCP, RN7SL306P, ODCP, TPI1P2, CYCSP20, RNU7-16P, SNRPGP3, RNU1-72P, RNA5SP244, MIR182, MIR96, MIR183, RNA5SP245, MIR335, RNA5SP246, MIR29B1, MIR29A, LINC-PINT, RNU6-1010P, MKLN1-AS, COX5BP3, TUBB3P2, SDHDP2, RNU6-1154P, RNU6-223P, PSMC1P3, MIR490, KRT8P51, RN7SKP223, SND1, LEP, RBM28, PRRT4, IMPDH1, HILPDA, METTL2B, FAM71F2, FAM71F1, CALU, OPN1SW, CCDC136, FLNC, ATP6V1F, IRF5, TNPO3, TSPAN33, SMO, AHCYL2, STRIP2, SMKR1, NRF1, UBE2H, ZC3HC1, KLHDC10, TMEM209, SSMEM1, CPA2, CPA4, CPA5, CPA1, CEP41, MEST, COPG2, TSGA13, KLF14, MKLN1, PODXL, PLXNA4, CHCHD3, EXOC4, LRGUK, SLC35B4, AKR1B1, AKR1B10, AKR1B15, BPGM, CALD1
chr7	137790175	138554395	M11	AKR1D1, TRIM24, SVOPL, ATP6V0A4, TMEM213, KIAA1549, RCC2P3, MIR4468, UQCRFS1P2
chr7	138554395	140507759	M11,M14	KDM7A, SLC37A3, RAB19, MKRN1, DENND2A, ADCK2, NDUFB2, BRAF, KIAA1549, ZC3HAV1L, ZC3HAV1, TTC26, UBN2, C7orf55, C7orf55-LUC7L2, LUC7L2, KLRG2, CLEC2L, HIPK2, TBXAS1, PARP12, RNU6-1272P, RNU6-206P, RNU6-911P, ERHP1, JHDM1D-AS1, RNU6-797P, RNU1-58P, PPP1R2P6, RNA5SP247, RNA5SP248, RN7SL771P, NDUFB2-AS1
chr7	140507759	141301080	M11,M14,M2	CCT4P1, RNU4-74P, BRAF, MRPS33, TMEM178B, AGK
chr7	141301080	141310994	M14,M2	AGK
chr7	141310994	142638500	M11,M14,M2	WEE2-AS1, RNU1-82P, TAS2R6, MTND2P5, MTND1P3, MYL6P4, OR9A3P, OR9A1P, OR9N1P, MOXD2P, PRSS3P3, TRBV1, TRBV7-5, TRBV12-2, TRBV11-2, TRBV12-1, TRBV8-2, TRBV5-2, TRBV8-1, TRBV21-1, TRBV22-1, TRBVA, TRBV26, TRBVB, PGBD4P1, PRSS3P1, PRSS3P2, WBP1LP1, AGK, KIAA1147, WEE2, SSBP1, TAS2R3, TAS2R4, TAS2R5, PRSS37, MGAM, OR9A4, CLEC5A, TAS2R38, PRSS58, TRBV2, TRBV3 1, TRBV4-1, TRBV5-1, TRBV6-1, TRBV7-1, TRBV4-2, TRBV7-8, TRBV6-9, TRBV5-7, TRBV7-7, TRBV6-8, TRBV5-6, TRBV7-6, TRBV6-7, TRBV5-5, TRBV6-6, TRBV5-4, TRBV7-4, TRBV6-5, TRBV10-2, TRBV11-1, TRBV10-1, TRBV9, TRBV5-3, TRBV7-3, TRBV6-4, TRBV19, TRBV20-1, TRBV23-1, TRBV24-1, MTRNR2L6, TRBV25-1, TRBV27, TRBV28, TRBV29-1, PRSS1, TRBJ2-1, TRBJ2-2, TRBJ2-2P, TRBJ2-3, TRBJ2-4, TRBJ2-5, TRBJ2-6, TRBJ2-7, TRBC2, TRBV30, EPHB6, TRPV6, TRPV5, C7orf34, KEL
chr7	142638500	142639520	M14,M2	KEL
chr7	142639520	148513870	M11,M14,M2	OR9A2, OR6V1, PIP, TAS2R39, TAS2R40, GSTK1, TMEM139, OR9P1P, OR6W1P, RN7SL535P, RN7SL481P, HINT1P1, CASP2, EPHA1-AS1, TAS2R62P, OR2R1P, PAICSP5, CLCN1, CTAGE15, RNU6-162P, FAM115B, RNU6-267P, PAICSP6, FAM115D, OR2Q1P, SLC16A1P1, OR2A41P, OR2A15P, OR2A13P, OR2A3P, FAM131B, OR2AO1P, OR2A20P, OR2A1-AS1, ZYX, ARHGEF34P, OR2A9P, RNU6ATAC40P, EEF1A1P10, RN7SKP174, EI24P4, RPL7P59, RN7SL207P, DUTP3, RANP2, MIR548F4, RNU6-1184P, RNA5SP249, RN7SL456P, RN7SL72P, EPHA1, TAS2R60, TAS2R41, OR10AC1P, FAM115C, CTAGE6, FAM115A, OR2F2, OR2F1, OR6B1, OR2A5, OR2A25, OR2A12, OR2A2, OR2A14, CTAGE4, ARHGEF35, OR2A42, OR2A7, CTAGE8, OR2A1, ARHGEF5, NOBOX, TPK1, CNTNAP2, C7orf33, CUL1, EZH2, KEL
chr7	148513870	148984655	M11,M14	RNU7-20P, RN7SL569P, RNY5, RNY4, RNY3, RNY1, RNU6-650P, COX6B1P1, RN7SL521P, EZH2, PDIA4, ZNF786, ZNF425, ZNF398, ZNF282, ZNF212, ZNF783
chr7	148984655	158935187	M11,M14,M2	LRRC61, ZBED6CL, RARRES2, REPIN1, ZNF775, GIMAP8, GIMAP7, GIMAP4, GIMAP6, GIMAP2, GIMAP1, GIMAP5, TMEM176B, TMEM176A, AOC1, KCNH2, NOS3, NPM1P12, ZNF767, SSPO, ATP6V0E2-AS1, ABCB8, LINC00996, STRADBP1, ALDH7A1P3, ATG9B, ASIC3, IQCA1P1, MIR671, WDR86-AS1, MIR3907, RN7SL76P, ETF1P2, PRKAG2-AS1, RNU6-604P, YBX1P4, SEPT7P6, FABP5P3, CCT8L1P, LINC01003, RNA5SP250, RN7SL811P, ATP5F1P3, RN7SL845P, PAXIP1-AS1, RN7SKP280, CDK5, LINC01006, RNU4-31P, MNX1-AS2, MNX1-AS1, RN7SL142P, MIR153-2, MIR595, LINC01022, MIR5707, THAP5P1, LINC00689, SLC4A2, FASTK, ZNF783, ZNF777, ZNF746, TMUB1, AGAP3, KRBA1, ZNF467, ZNF862, ATP6V0E2, GBX1, ASB10, ACTR3C, ABCF2, CHPF2, SMARCD3, NUB1, WDR86, CRYGN, RHEB, PRKAG2, GALNTL5, GALNT11, KMT2C, XRCC2, ACTR3B, DPP6, PAXIP1-AS2, PAXIP1, HTR5A-AS1, HTR5A, INSIG1, BLACE, EN2, CNPY1, RBM33, SHH, C7orf13, RNF32, LMBR1, NOM1, MNX1, UBE3C, DNAJB6, PTPRN2, NCAPG2, ESYT2, WDR60, VIPR2
chr7	158935187	158935237	M11,M2	VIPR2
chr8	116087	116555	M9	OR4F21
chr8	116555	21847886	M14,M9	PPP1R3B, TNKS, MSRA, PRSS55, FAM90A9P, FAM90A10P, RP1L1, C8orf74, HSPD1P2, DEFB108P1, FAM66E, SOX7, USP17L8, USP17L3, FAM90A11P, PINX1, FAM90A24P, XKR6, FAM90A12P, OR7E96P, MIR548I3, ENPP7P1, FAM85B, MTMR9, FAM86B3P, ALG1L13P, RN7SL178P, SLC35G5, RNU6-682P, C8orf12, MIR4660, RNU7-55P, FAM167A, BLK, RNU6-1151P, RNU6-526P, GATA4, MIR597, LINC00599, C8orf49, NEIL2, RNU6-729P, PRSS51, MIR4286, RNA5SP252, MIR598, LINC00529, RPL19P13, FDFT1, TDH, RN7SL293P, RNU6-1084P, LINC00208, SUB1P1, OR7E158P, OR7E161P, OR7E160P, RNA5SP253, DEFB108P3, FAM66D, USP17L7, DEFB109P3, FAM90A2P, ALG1L11P, FAM85A, ENPP7P12, RNA5SP254, DEFB108P4, ZNF705C, FAM66A, DEFB109P1, LINC00965, FAM90A25P, ALG1L12P, ENPP7P6, RPS3AP34, RPS3AP35, OR7E8P, OR7E15P, OR7E10P, MIR5692A1, MIR5692A2, MIR3926-1, MIR3926-2, LINC00681, RNU6-842P, MTND4P7, RNA5SP255, CTSB, RNU7-153P, RNU6-397P, MIR383, PPM1AP1, MRPL49P2, RN7SL474P, ADAM24P, MIR548V, RNA5SP256, NATP, DEFB136, DEFB135, RPL35P6, DEFB134, ZNF705D, USP17L2, RPL30P9, RNU6-892P, LZTS1-AS1, FAM86B1, TMEM97P2, OR6R2P, DEFB130, FAM86B2, LONRF1, KIAA1456, DLC1, C8orf48, SGCZ, TUSC3, MSR1, FGF20, MICU3, ZDHHC2, CNOT7, RPL23AP53, FAM87A, ERICH1-AS1, VPS37A, MIR596, KBTBD11-OT1, RNA5SP251, RN7SL872P, PAICSP4, MTMR7, RN7SL318P, RPL23AP54, RN7SKP159, MIR4659A, MIR4659B, SLC7A2, XKR5, DEFA8P, DEFA9P, DEFA10P, PDGFRL, DEFT1P, DEFT1P2, DEFA11P, DEFA7P, RPS3AP30, RPS3AP33, OR7E125P, FAM90A15P, MTUS1, FAM90A3P, FAM90A4P, FAM90A13P, FAM90A5P, FAM90A20P, FAM66B, DEFB109P1B, USP17L1P, USP17L4, DEFB108P2, HSPD1P3, FAM90A6P, FAM90A7P, FAM90A21P, FAM90A22P, FAM90A23P, OR7E157P, OR7E154P, FAM90A14P, FAM90A18P, FAM90A16P, FAM90A8P, FAM90A17P, FAM90A19P, FGL1, PCM1, ASAH1, NAT1, NAT2, PSD3, SH2D4A, CSGALNACT1, INTS10, LPL, SLC18A1, ATP6V1B2, LZTS1, GFRA2, DOK2, XPO7, OR4F21, ZNF596, FBXO25, TDRP, ERICH1, DLGAP2, CLN8, ARHGEF10, KBTBD11, MYOM2, CSMD1, MCPH1, ANGPT2, AGPAT5, DEFB1, DEFA6, DEFA4, DEFA1, DEFA1B, DEFA3, DEFA5, ZNF705G, DEFB4B, DEFB103B, SPAG11B, DEFB104B, DEFB106B, DEFB105B, DEFB107B, PRR23D1, PRR23D2, DEFB107A, DEFB105A, DEFB106A, DEFB104A, SPAG11A, DEFB103A, DEFB4A, ZNF705B, CLDN23, MFHAS1, ERI1
chr8	21847886	22103042	M9	MIR320A, XPO7, NPM2, FGF17, DMTN, FAM160B2, NUDT18, HR, REEP4, LGI3, SFTPC, BMP1, PHYHIP, POLR3D
chr8	22103042	39172973	M14,M9	PPAPDC1B, WHSC1L1, RNU6-1086P, MIR3622B, MIR3622A, RNU6-1276P, MIR4287, RPL5P22, RNU6-178P, MIR4288, RNA5SP259, EXTL3-AS1, HMBOX1-IT1, RNA5SP260, RN7SL781P, HMGB1P23, RPL17P33, LINC00589, FAM183CP, MIR3148, MAP2K1P1, RNU6-1218P, RPS15AP24, HSPA8P11, TUBBP1, RBPMS-AS1, RNU5A-3P, UBXN8, LETM2, SUMO2P16, RNA5SP261, NRG1-IT1, NRG1-IT2, RNA5SP262, RNA5SP263, NRG1-IT3, RNU6-663P, MTND1P6, MTND2P32, RANP9, RNU6-528P, SNORD13, RN7SL621P, RN7SL457P, VENTXP5, LSM12P1, FGFR1, RN7SKP201, MTND6P19, RNU6-533P, RPL23P10, RNA5SP264, TPT1P8, SMARCE1P4, RNU6-607P, RN7SL709P, RPS20P22, RNF5P1, RPL3P10, ADAM5, RNU6-336P, RN7SL303P, RNU4-71P, RNU1-148P, NEFL, RN7SL651P, RNA5SP258, SDAD1P1, COX6B1P4, MIR548H4, GULOP, C8orf86, TACC1, PLEKHA2, HTRA4, TM2D2, ADAM9, ADAM32, POLR3D, PIWIL2, SLC39A14, PPP3CC, SORBS3, PDLIM2, C8orf58, CCAR2, BIN3, EGR3, PEBP4, RHOBTB2, TNFRSF10B, TNFRSF10C, TNFRSF10D, TNFRSF10A, CHMP7, R3HCC1, LOXL2, ENTPD4, SLC25A37, NKX3-1, NKX2-6, STC1, ADAM28, ADAMDEC1, ADAM7, NEFM, DOCK5, GNRH1, KCTD9, CDCA2, EBF2, PPP2R2A, BNIP3L, PNMA2, DPYSL2, ADRA1A, STMN4, TRIM35, PTK2B, CHRNA2, EPHX2, CLU, SCARA3, CCDC25, ESCO2, PBK, SCARA5, NUGGC, ELP3, PNOC, ZNF395, FBXO16, FZD3, EXTL3, INTS9, HMBOX1, KIF13B, DUSP4, TMEM66, LEPROTL1, MBOAT4, DCTN6, RBPMS, GTF2E2, SMIM18, GSR, PPP2CB, TEX15, PURG, WRN, NRG1, FUT10, TTI2, MAK16, RNF122, DUSP26, UNC5D, KCNU1, ZNF703, ERLIN2, PROSC, GPR124, BRF2, RAB11FIP1, GOT1L1, ADRB3, EIF4EBP1, ASH2L, STAR, LSM1, BAG4, DDHD2
chr8	39172973	39682416	M14,M16,M9	ADAM5, ADAM3A, ADAM18, ADAM2
chr8	39682416	43218328	M14,M9	RNU6-356P, MIR548AO, RPS29P2, RNU6-895P, KRT18P37, MIR486, RN7SL149P, RPL5P23, RN7SL806P, MIR4469, RNU1-124P, VN1R46P, AFG3L2P1, POTEA, RNU6-104P, ADAM2, IDO1, IDO2, C8orf4, ZMAT4, SFRP1, GOLGA7, GINS4, AGPAT6, NKX6-3, ANK1, KAT6A, AP3M2, PLAT, IKBKB, POLB, DKK4, VDAC3, SLC20A2, SMIM19, CHRNB3, CHRNA6, THAP1, RNF170, HOOK3, FNTA, POMK, HGSNAT
chr8	43218328	48192449	M14	POTEA, RN7SKP41, SNX18P27, CYP4F44P, HSPA8P13, ASNSP1, ASNSP4, TRIM60P15, LINC00293, MTND1P7, RNU6-656P, MTND6P20, MAPK6PS4, RN7SKP32, RNU6-819P, RPL10AP2, NDUFA5P12, ATP6V1G1P2, IGLV8OR8-1, SPIDR
chr8	48192449	49642278	M14,M2,M9	RNU6-665P, RNU6-519P, RNU6-295P, RPL29P19, SPIDR, CEBPD, PRKDC, MCM4, UBE2V2, EFCAB1
chr8	49642278	54742093	M14,M16,M2,M9	RN7SKP294, RFPL4AP7, PSAT1P1, CYCSP22, RPL34P17, EFCAB1, SNAI2, C8orf22, SNTG1, PXDNL, PCMTD1, ST18, FAM150A, RB1CC1, NPBWR1, OPRK1, ATP6V1H
chr8	54742093	59493121	M14,M2,M9	RNU6-1331P, TDGF1P5, RNU6ATAC32P, RNU105C, RN7SL250P, SBF1P1, RNA5SP265, RN7SL798P, RN7SL323P, SNORD54, NPM1P21, SDR16C6P, SEPT10P1, LINC00968, RNU6-13P, RNA5SP266, LINC00588, RNU6-596P, RPL30P10, RNU7-174P, ATP6V1H, RGS20, TCEA1, LYPLA1, MRPL15, SOX17, RP1, XKR4, TMEM68, TGS1, LYN, RPS20, MOS, PLAG1, CHCHD7, SDR16C5, PENK, IMPAD1, FAM110B, UBXN2B, CYP7A1, SDCBP
chr8	59493121	59535804	M14,M2,M6,M9	SDCBP, NSMAF

chr8	59535804	128429439	M14,M2,M9	C8orf59, CA13, RN7SL777P, MIR4661, RN7SKP231, MRPS16P1, IRF5P1, LINC00535, RNA5SP274, ZNF317P1, MIR378D2, PSMA2P2, RPL34P18, RPS4XP10, RNU6-1209P, MIR3150B, MIR3150A, RNU6-690P, CA1, RNU6-1172P, RNU7-177P, SUMO2P18, RPS23P1, SNORA72, RNU6-703P, RNU6-914P, RNU6-748P, RPL19P14, MRP63P7, CA3, RN7SKP85, MIR599, MIR875, CA2, RN7SL350P, MIR1273A, MIR4471, GAPDHP62, RNU6-1092P, RNU6ATAC41P, RPS20P23, RNU4-83P, RPS26P6, RN7SL685P, RN7SKP249, RNU7-67P, NACAP1, DUXAP2, RN7SL563P, MIR5680, SUMO2P19, RNU6ATAC8P, HSPE1P14, RPS12P15, RNU6-1224P, POU5F1P2, REXO1L1P, REXO1L11P, REXO1L10P, ATP6V0D2, RPL5P24, NPM1P52, MTND1P5, MIR3151, PSKH2, RNU4-50P, RNU6-1011P, SLC7A13, RNA5SP267, MIR548A3, NDUFA5P2, TMCC1P1, SLC2A13P1, PDCL3P1, RNU6-1202P, WWP1, NPM1P6, KRT8P3, RN7SKP97, MIR4470, SLC16A14P1, TAGLN2P1, RNU7-84P, HMGB1P46, PGAM1P13, RNA5SP275, NRBF2P4, RPS17P14, RNU2-67P, EEF1A1P37, RNU4-37P, RPL30P16, MIR2053, LINC00536, XRCC6P4, YTHDF3-AS1, RN7SL135P, IFITM8P, RN7SKP135, COX6CP8, LINC00966, MIR124-2, RNA5SP276, RAD21-AS1, MIR3610, RN7SL228P, RN7SL826P, RPS10P16, RPS26P35, RNU6-12P, MAL2, RN7SKP153, RN7SL396P, RNA5SP277, RPL35AP19, HAS2-AS1, MRPS36P3, LINC01151, RNY4P5, HMGB1P19, FAM83A-AS1, MIR4663, UBA52P5, RNU6-628P, RNU6-875P, MIR548AA1, MIR548D1, DUTP2, IMPDH1P6, RN7SKP155, FER1L6-AS1, FER1L6-AS2, ARF1P3, RNU6-756P, RNF139-AS1, MIR4662A, MIR4662B, LINC00964, RMDN1, RPL31P41, LINC00967, RNU6-1324P, PTTG3P, SNHG6, SNORD87, RNA5SP268, NDUFS5P6, RPL31P40, RPS15AP25, RNA5SP269, RNU7-102P, RN7SKP29, RN7SL675P, RNA5SP270, SDCBPP2, SUMO2P20, RNU1-101P, BTF3P12, CPNE3, RN7SL19P, TRAPPC2P2, RPS20P20, KIAA0196-AS1, RN7SL329P, RN7SL590P, RNA5SP271, RNU6-442P, LINC00861, HAUS1P3, SOD1P3, KNOP1P5, RFPL4AP5, RNU6-869P, RNU11-4P, PCAT1, PCAT2, PRNCR1, CASC19, CCAT1, CASC8, RNU6-285P, STAU2-AS1, VENTXP6, GYG1P1, RPS20P21, RNU6-1300P, CNGB3, RPS3AP32, RNU6-1197P, MIR5681A, CASC9, HIGD1AP6, PKMP4, RNU2-54P, LINC01109, LINC01111, ZFHX4-AS1, MRPL9P1, HIGD1AP18, RNU6-1220P, PRKRIRP7, RPL3P9, RNU7-85P, RN7SL41P, MIR5708, RNU6-1213P, RPSAP47, RNU2-71P, SLC25A51P3, RN7SL107P, CKS1BP7, RNU11-6P, RN7SL308P, HMGB1P41, UBE2HP1, FTH1P11, IMPA1P, NIPA2P4, RPS26P34, SLC10A5P1, HNRNPA1P36, HNRNPA1P4, TPM3P3, RNU6-1040P, ACTBP6, IGJP1, REXO1L8P, REXO1L3P, REXO1L12P, REXO1L9P, REXO1L2P, NTAN1P2, SLC2A3P4, GOLGA2P1, UBE2Q2P10, SOX5P, RNA5SP272, KRT8P4, COX6B1P6, RNU6-925P, LINC00534, RNA5SP273, LINC01030, CNBD1, DCAF4L2, MMP16, RIPK2, OSGIN2, NBN, DECR1, CALB1, TMEM64, NECAB1, C8orf88, TMEM55A, OTUD6B, LRRC69, SLC26A7, RUNX1T1, TRIQK, C8orf87, FAM92A1, RBM12B, RBM12B-AS1, TMEM67, PDP1, CDH17, GEM, RAD54B, FSBP, KIAA1429, ESRP1, DPY19L4, INTS8, CCNE2, NDUFAF6, TP53INP1, PLEKHF2, C8orf37, GDF6, UQCRB, MTERFD1, PTDSS1, SDC2, CPQ, TSPYL5, MTDH, LAPTM4B, MATN2, RPL30, C8orf47, HRSP12, POP1, NIPAL2, STK3, KCNS2, OSR2, VPS13B, COX6C, RGS22, FBXO43, POLR2K, SPAG1, RNF19A, ANKRD46, SNX31, PABPC1, YWHAZ, ZNF706, GRHL2, NCALD, RRM2B, UBR5, ODF1, KLF10, AZIN1, ATP6V1C1, C8orf56, BAALC, FZD6, CTHRC1, SLC25A32, DCAF13, RIMS2, NSMAF, DPYS, DCSTAMP, LRP12, ZFPM2, TOX, CA8, RAB2A, CHD7, OXR1, CLVS1, ABRA, ANGPT1, ASPH, RSPO2, EIF3E, EMC2, TMEM74, TRHR, NUDCD1, ENY2, NKAIN3, PKHD1L1, GGH, TTPA, YTHDF3, BHLHE22, EBAG9, CYP7B1, ARMC1, MTFR1, SYBU, PDE7A, KCNV1, DNAJC5B, TRIM55, CSMD3, CRH, RRS1, ADHFE1, C8orf46, MYBL1, TRPS1, EIF3H, UTP23, VCPIP1, C8orf44-SGK3, C8orf44, RAD21, SGK3, MCMDC2, AARD, SLC30A8, TCF24, PPP1R42, MED30, EXT1, COPS5, SAMD12, TNFRSF11B, COLEC10, NOV, CSPP1, ENPP2, TAF2, DSCC1, ARFGEF1, DEPTOR, COL14A1, CPA6, PREX2, MRPL13, MTBP, SNTB1, C8orf34, HAS2, ZHX2, DERL1, TBC1D31, SULF1, FAM83A, C8orf76, SLCO5A1, ZHX1-C8ORF76, ZHX1, PRDM14, NCOA2, ATAD2, TRAM1, WDYHV1, LACTB2, XKR9, FBXO32, EYA1, KLHL38, ANXA13, FAM91A1, FER1L6, TMEM65, TRMT12, RNF139, TATDN1, MSC, TRPA1, KCNB2, TERF1, NDUFB9, SBSPON, MTSS1, RPL7, RDH10, ZNF572, SQLE, STAU2, KIAA0196, UBE2W, NSMCE2, TRIB1, FAM84B, POU5F1B, TCEB1, TMEM70, LY96, JPH1, GDAP1, PI15, CRISPLD1, HNF4G, ZFHX4, PEX2, PKIA, ZC2HC1A, IL7, STMN2, HEY1, MRPS28, TPD52, ZBTB10, ZNF704, PAG1, FABP5, PMP2, FABP9, FABP4, FABP12, IMPA1, SLC10A5, ZFAND1, CHMP4C, SNX16, RALYL, LRRCC1, E2F5
chr8	128429439	128491327	M14,M2	CASC8, POU5F1B
chr8	128491327	144240279	M14,M2,M9	AGO2, PTK2, DENND3, CASC8, CASC11, PVT1, MIR1205, RNU1-106P, RNU4-25P, MIR1207, MIR1208, RN7SKP226, SLC45A4, LINC00977, RN7SKP206, CCDC26, MIR3686, RNU7-181P, MIR5194, RNU6-1255P, HPYR1, WISP1-OT1, ST13P6, MTND2P7, ZFAT-AS1, GPR20, PTP4A3, MIR30B, MIR30D, MAPRE1P1, RNU1-35P, RNU6-144P, CASC7, MIR151A, RNA5SP278, MROH5, TSNARE1, HNRNPA1P38, MIR4472-1, LINC00051, MROH4P, JRK, ZNHIT1P1, CDC42P3, AK3P2, BAI1, ARC, PSCA, LY6K, THEM6, SLURP1, LYPD2, LYNX1, LY6D, GML, CYP11B1, CYP11B2, LY6E, C8orf31, LY6H, MYC, TMEM75, GSDMC, FAM49B, ASAP1, ADCY8, EFR3A, OC90, HHLA1, KCNQ3, LRRC6, TMEM71, PHF20L1, TG, SLA, WISP1, NDRG1, ST3GAL1, ZFAT, KHDRBS3, FAM135B, COL22A1, KCNK9, TRAPPC9, C8orf17, CHRAC1
chr8	144240279	144620831	M2,M9	RNU6-220P, RHPN1-AS1, LY6H, GPIHBP1, ZFP41, GLI4, ZNF696, TOP1MT, RHPN1, MAFA, ZC3H3
chr8	144620831	144698332	M2,M6,M9	ZC3H3, GSDMD, MROH6, NAPRT1, EEF1D, TIGD5, PYCRL, TSTA3
chr8	144698332	144895667	M2,M9	RNU7-109P, MIR4664, FAM83H-AS1, MIR937, TSTA3, ZNF623, ZNF707, CCDC166, MAPK15, FAM83H, SCRIB
chr8	144895667	145166609	M2,M6,M9	MIR661, OPLAH, SCRIB, PUF60, NRBP2, EPPK1, PLEC, PARP10, GRINA, SPATC1, EXOSC4, GPAA1, CYC1, SHARPIN, MAF1, KIAA1875
chr8	145166609	145634477	M2,M9	TSSK5P1, MIR939, MIR1234, KIAA1875, FAM203A, MROH1, SCXB, FAM203B, BOP1, SCXA, HSF1, DGAT1, SCRT1, TMEM249, SLC52A2, FBXL6, ADCK5, CPSF1
chr8	145634477	145752793	M2,M6,M9	CYHR1, KIFC2, FOXH1, PPP1R16A, GPT, MFSD3, RECQL4, LRRC14, LRRC24, C8orf82, CPSF1, SLC39A4, VPS28, TONSL
chr8	145752793	146281415	M2,M9	ZNF252P, TMED10P1, ZNF252P-AS1, C8orf82, ARHGAP39, ZNF251, ZNF34, RPL8, ZNF517, ZNF7, COMMD5, ZNF250, ZNF16, RN7SL395P, C8orf33
chr9	14510	14725	M1,M16,M17,M8	DDX11L5
chr9	14725	29670	M1,M16,M17,M6,M8	MIR1302-11, MIR1302-10, MIR1302-9, MIR1302-2
chr9	29670	29739	M1,M16,M17,M8	MIR1302-11, MIR1302-10, MIR1302-9, MIR1302-2
chr9	29739	175784	M1,M16,M17	FOXD4, CBWD1, MIR1302-11, MIR1302-10, MIR1302-9, MIR1302-2, FAM138C
chr9	175784	179075	M1,M17	CBWD1
chr9	179075	312166	M1	CBWD1, C9orf66, DOCK8
chr9	37904207	37915895	M14	SLC25A51
chr9	37915895	39072765	M14,M16	RNU7-124P, VN1R48P, FAM220BP, CYP4F33P, SNX18P3, FAM201A, RNU6-765P, VN2R3P, SHB, ALDH1B1, IGFBPL1, ANKRD18A, CNTNAP3
chr9	39072765	45728282	M1,M14,M16	RN7SL640P, FAM74A5, ATP5A1P9, RN7SL462P, FAM74A1, VN2R4P, VN2R5P, RN7SL422P, FAM74A3, ATP5A1P1, FAM74A2, FAM74A6, ZNF658B, ATP5A1P8, RBPJP5, RN7SL763P, MEP1AP1, RNU6-1269P, SNX18P8, FAM95B1, CNN2P2, IGKV1OR9-2, MTND1P12, BMS1P14, AQP7P3, CYP4F59P, SNX18P4, RNU6-538P, MEP1AP3, ATP5A1P5, RN7SL343P, CNN2P4, CYP4F60P, SNX18P5, ANKRD20A7P, RNU6-599P, MEP1AP4, RBPJP6, ATP5A1P6, FAM27E4, CDK2AP2P1, MYO5BP1, RN7SL565P, CNTNAP3, SPATA31A1, SPATA31A2, SPATA31A3, ZNF658, SPATA31A4, SPATA31A5, ANKRD20A2, CBWD7, FOXD4L2, ANKRD20A3, SPATA31A6, CNTNAP3B, FAM27C, FAM27A
chr9	45728282	66499628	M14	RN7SL722P, RN7SL544P, RBPJP7, ATP5A1P7, VN2R6P, FAM74A4, RBPJP2, ATP5A1P10, CNN2P5, CYP4F61P, SNX18P6, RNA5SP283, FAM27E2, FAM27E1, FAM27D1, SPATA31A7
chr9	66499628	71357573	M14,M16	PTGER4P2, CDK2AP2P2, MYO5BP2, RNU6-156P, BMS1P9, AQP7P4, AQP7P1, BMS1P10, RN7SL787P, RNU6-368P, MYO5BP3, CDK2AP2P3, PTGER4P3, RNA5SP284, LINC00537, MIR4477B, MIR4477A, PGM5P1, MIR1299, PGM5P2, RNU6-1193P, SNX18P9, CYP4F25P, CNN2P3, AQP7P2, BMS1P11, IGKV1OR-2, RNU6-1293P, BMS1P12, IGKV1OR9-1, BMS1P13, IGKV1OR-3, MEP1AP2, PGM5-AS1, FAM27E3, FAM27B, ANKRD20A1, FOXD4L6, CBWD6, ANKRD20A4, FOXD4L5, FOXD4L4, CBWD5, CBWD3, FOXD4L3, PGM5, TMEM252, PIP5K1B
chr9	71357573	71549883	M14	PIP5K1B, FAM122A
chr9	137642387	137716701	M1	COL5A1
chr9	140057376	140167012	M6	MIR3621, GRIN1, LRRC26, TMEM210, ANAPC2, SSNA1, TPRN, TMEM203, NDOR1, RNF208, C9orf169, RNF224, SLC34A3, TUBB4B, FAM166A, C9orf173, NELFB
chrX	30714733	30852218	M9	GK, TAB3, GK-AS1
chrX	30852218	44107656	M14,M9	TAB3, FTHL17, DMD, FAM47A, TMEM47, FAM47B, MAGEB16, CXorf22, CHDC2, CXorf30, FAM47C, PRRG1, LANCL3, XK, CYBB, DYNLT3, CXorf27, SYTL5, SRPX, RPGR, OTC, TSPAN7, MID1IP1, BCOR, ATP6AP2, CXorf38, MED14, USP9X, DDX3X, NYX, CASK, GPR34, GPR82, MAOA, MAOB, NDP, EFHC2, TAB3-AS1, TAB3-AS2, RNU6-894P, RNA5SP501, NPM1P8, MIR3915, MIR548F5, DMD-AS3, TBCAP1, FTH1P14, RNU6-1087P, SIAH1P1, RNU6-641P, HMGB1P16, RPS15AP40, FTH1P18, MOB1AP2, FTH1P19, FTH1P27, FAM47DP, RNU6-49P, MIR548AJ2, TDGF1P1, FTLP16, RNU7-7P, MID1IP1-AS1, RNU6-591P, MIR3937, RN7SL732P, GAPDHP1, MIR1587, RPS11P7, RNU7-164P, MPC1L, MED14-AS1, DPRXP6, MKRN4P, SDCBPP3, RPS2P55, RNA5SP502, RN7SL15P, CASK-AS1, RNU6-1321P, RN7SL406P, RN7SL144P, RNU6-202P, ATP5G2P4, RNU6-630P, RNU6-1124P, PPP1R2P9, IMPDH1P4, NANOGP10, NDP-AS1
chrX	44107656	46893106	M14	MIR222, MED28P4, KRT18P68, PCNAP3, ACTBP1, GAPDHP65, RNU6-50P, ZNF674-AS1, MKI67P1, PGAM1P7, YBX1P8, EFHC2, FUNDC1, DUSP21, KDM6A, CXorf36, KRBOX4, ZNF674, CHST7, SLC9A7, RP2, CXorf31, JADE3, TATDN2P1, RRM2P3, FDPSP5, CHTF8P1, RPL19P20, RN7SL291P, RNU6-523P, KRT8P14, MIR221
chrX	47046041	47099247	M6	INE1, RBM10, UBA1, CDK16, USP11
chrX	54841680	57937067	M9	MTND2P24, MTND1P30, RPL23AP83, UQCRBP1, PPP1R11P2, MTHFD1P1, MAGED2, TRO, PFKFB1, APEX2, ALAS2, PAGE2B, PAGE2, FAM104B, MTRNR2L10, PAGE5, PAGE3, MAGEH1, USP51, FOXR2, RRAGB, KLF8, UBQLN2, SPIN3, SPIN2B, SPIN2A, FAAH2, ZXDB, ZXDA
chrX	77359665	79277741	M9	FNDC3CP, HMGN1P34, UBE2V1P7, PPATP2, RPL7P54, KIF4CP, PGK1, TAF9B, CYSLTR1, ZCCHC5, LPAR4, P2RY10, GPR174, ITM2A, TBX22

chrX	79277741	100093353	M16,M9	MIR548I4, SETP4, UBE2DNL, MIR361, NDUFA5P7, TPMTP4, EEF1A1P29, GEMIN8P3, FCF1P9, RPSAP15, SRIP2, USP12PX, RNF19BPX, UBE2V1P9, RNU6-555P, PABPC5-AS1, SERBP1P4, RNU2-26P, VDAC1P3, EIF4A1P10, KRT18P11, SNX3P1X, ST13P18, RPL7P55, RNU6-332P, PAICSP7, CCNB1IP1P3, MIR548M, CALM1P1, RNA5SP510, HNRNPDLP3, HNRNPDLP1, RPS7P13, RN7SL379P, RPS29P28, RN7SKP194, RN7SL74P, DIAPH2-AS1, RPL6P29, EEF1A1P15, HMGB1P32, XRCC6P5, RPSAP8, CHMP1B2P, HNRNPH3P1, HK2P1, RNU6-493P, VDAC1P1, RNU6-995P, RNU6-974P, RPL22P22, ATG4AP1, TERF1P4, TBX22, FAM46D, BRWD3, HMGN5, SH3BGRL, POU3F4, CYLC1, RPS6KA6, HDX, APOOL, SATL1, ZNF711, POF1B, CHM, DACH2, KLHL4, CPXCR1, TGIF2LX, PABPC5, PCDH11X, NAP1L3, FAM133A, DIAPH2, RPA4, PCDH19, TNMD, TSPAN6, SRPX2, SYTL4, CSTF2
chrX	100093353	100095921	M9	CSTF2
chrX	103903625	107403692	M14	RPL18AP14, KCTD9P2, RNU6-207P, NAP1L4P2, MIR548AN, EEF1A1P40, MYCLP1, DNAJA1P3, KRT18P49, FRMPD3-AS1, IL1RAPL2, TEX13A, NRK, SERPINA7, MUM1L1, CXorf57, RNF128, TBC1D8B, MORC4, RIPPLY1, CLDN2, RBM41, NUP62CL, PIH1D3, FRMPD3, PRPS1, TSC22D3, NCBP2L, MID2, TEX13B, VSIG1, PSMD10, ATG4A, COL4A6
chrX	107403692	107431746	M1,M14	COL4A6
chrX	107431746	107929359	M1,M14,M17	COL4A6, COL4A5
chrX	107929359	108636284	M14,M17	RNU6-309P, COL4A5, IRS4, GUCY2F
chrX	108636284	114391197	M14	RPS5P7, MIR652, MIR3978, SNORD96B, AMMECR1-IT1, TDGF1P3, FCF1P4, GLUD1P9, RNU6-496P, HMGB1P12, LINC00890, RN7SL661P, EIF4BP7, RPL18AP15, RNA5SP512, ALG13-AS1, TRPC5OS, DPRXP7, HMGB3P30, MIR4329, RN7SL266P, RNU6-1015P, QTRT1P1, RNU1-57P, RN7SL93P, SSU72P1, RPL36AP53, SNORA35, MIR764, MIR1912, MIR1298, MIR1911, HSPA8P7, MIR448, RNU6-648P, YAP1P2, GUCY2F, NXT2, KCNE1L, ACSL4, TMEM164, AMMECR1, GNG5P2, RGAG1, CHRDL1, PAK3, CAPN6, DCX, ALG13, TRPC5, ZCCHC16, LHFPL1, AMOT, HTR2C, IL13RA2, LRCH2
chrX	139866073	150909319	M14	ELL2P4, CXorf51B, CXorf51A, MIR513C, MIR513B, MIR513A1, MIR513A2, MIR506, MIR507, MIR508, MIR514B, MIR509-2, MIR509-3, MIR509-1, MIR510, MIR514A1, MIR514A2, MIR514A3, RNU6-382P, FMR1-AS1, FMR1-IT1, FTH1P8, RPL7L1P11, AFF2-IT1, RN7SKP267, RNU7-98P, IDSP1, LINC00893, TMEM185AP1, MAGEA7P, DUTP4, MAGEA8-AS1, LINC00894, MIR2114, XRCC6P2, RNU6-383P, RPL19P21, MIR4330, GPR50-AS1, MIR320D2, RNU6-3P, RBMX2P2, SPANXA2-OT1, RNA5SP516, RN7SKP81, RN7SKP149, PGBD4P6, MTND1P33, HNRNPH1P2, RRM2P4, HNRNPCP10, RN7SKP189, CYCSP44, MIR890, MIR888, MIR892A, MIR892B, MIR891B, MIR891A, RNA5SP517, CDR1, SPANXB2, SPANXB1, LDOC1, SPANXC, SPANXA1, SPANXA2, SPANXD, MAGEC3, MAGEC1, MAGEC2, SPANXN4, SPANXN3, SLITRK4, SPANXN2, UBE2NL, SPANXN1, SLITRK2, TMEM257, FMR1, FMR1NB, AFF2, IDS, CXorf40A, MAGEA9B, HSFX2, TMEM185A, MAGEA11, HSFX1, MAGEA9, MAGEA8, CXorf40B, MAMLD1, MTM1, MTMR1, CD99L2, HMGB3, GPR50, VMA21, PASD1, PRRG3, FATE1, CNGA2
chrX	153776182	155239981	M14	ATF4P2, ATF4P1, IKBKGP1, OR3B1P, SNORA36A, SNORA56, HMGN1P37, MIR1184-1, EEF1A1P31, MIR1184-3, MIR1184-2, TMLHE-AS1, AMDP1, DPH3P2, TCEB1P24, TRPC6P, IKBKG, CTAG1A, CTAG1B, CTAG2, GAB3, DKC1, MPP1, SMIM9, F8, H2AFB1, F8A1, FUNDC2, MTCP1, CMC4, BRCC3, VBP1, RAB39B, CLIC2, H2AFB2, F8A2, F8A3, H2AFB3, TMLHE, SPRY3, VAMP7, IL9R
chrY	2654895	6117053	M9	RNASEH2CP1, TOMM22P2, HSFY3P, NAP1L1P2, ZFY-AS1, EEF1A1P41, LINC00278, AGPAT5P1, PRRC2CP1, USP12PY, RNF19BPY, UBE2V1P3, RNU6-303P, SERBP1P2, RNU2-57P, VDAC1P6, EIF4A1P2, KRT18P10, RPL26P37, DLGAP5P1, TTTY23B, SRY, RPS4Y1, ZFY, TGIF2LY, PCDH11Y, TSPY2
chrY	6117053	8657040	M9	FAM197Y9, TSPY11P, TSPY19P, RBMY2GP, LINC00280, TTTY1B, TTTY2B, TTTY21B, TTTY7, TTTY8B, TSPY17P, SRIP3, GOT2P5, ATP5JP1, GPR143P, PRKY, RN7SKP282, RNU6-941P, RNU6-521P, RBMY2HP, TSPY12P, TTTY16, RFTN1P1, TTTY12, ZNF92P1Y, ZNF736P8Y, BPY2DP, ZNF736P7Y, ZNF736P9Y, RBMY2JP, RBMY2KP, TSPY24P, ZNF736P6Y, MTND6P1, MTCYBP1, MTND1P1, MTND2P3, TRIM60P3Y, ZNF736P10Y, LINC00279, TTTY18, TTTY19, TTTY11, TSPY2, AMELY, TBL1Y
chrY	8657040	9325425	M9	TSPY6P, TTTY11, OFD1P3Y, CDY3P, USP9YP22, USP9YP4, TCEB1P4, RBMY1A3P, TTTY20, FAM197Y8, FAM197Y7, TSPY7P, FAM197Y6, TSPY4, TSPY8, TSPY3, TSPY1
chrY	9325425	9452762	M9	TSPY6P, TSPY10, FAM197Y1, FAM197Y3, FAM197Y2, TSPY15P, RBMY3AP
chrY	14774297	14799392	M9	TTTY15
chrY	14799392	26980276	M4,M9	AGKP1, NLGN4Y-AS1, MED13P1, CYCSP46, HDHD1P1, STSP1, RNU6-109P, RNU6-184P, SURF6P1, TCEB1P35, FAM41AY1, TUBB1P2, CDY1B, FAM224B, RNA5SP520, RNA5SP521, CLUHP1, BPY2B, TAF9P1, CDY5P, PRYP1, ACTG1P2, USP9YP23, USP9YP27, DAZ3, USP9Y, RNU1-128P, TRAPPC2P3, OFD1P1Y, TCEB1P6, CDY6P, USP9YP7, USP9YP6, CDY7P, USP9YP34, USP9YP32, CDY8P, DAZ4, TCEB1P12, OFD1P2Y, TRAPPC2P8, USP9YP15, DDX3Y, RNU1-95P, USP9YP16, ACTG1P11, PRYP2, TCEB1P26, CDY9P, TAF9P2, CLUHP2, FAM224A, RNA5SP522, RNA5SP523, TUBB1P1, FAM41AY2, TCEB1P13, OFD1P4Y, USP9YP14, RNU1-48P, USP9YP5, UTY, XKRYP1, PRYP5, TCEB1P7, USP9YP1, GPM6BP1, TTTY9B, OFD1P5Y, RAB9AP4, TRAPPC2P7, OFD1P6Y, GPM6BP2, USP9YP2, TCEB1P14, PRYP6, XKRYP2, USP9YP10, USP9YP28, RNU1-41P, OFD1P7Y, MTCYBP2, TTTY14, ZNF839P1, CD24P4, RNU6-255P, GAPDHP19, BCORP1, TXLNG2P, TMSB4Y, RCC2P2, ZNF886P, VCY, ZNF885P, VCY1B, TTTY10, TBL1YP1, HSFY4P, NLGN4Y, GAPDHP17, TMEM167AP1, TOMM22P1, NEFLP1, RBMY2EP, CDY2B, RBMY2TP, TSPY14P, CDY2A, RBMY1HP, HSFY1, HSFY2, TTTY13, TCEB1P5, CDY10P, USP9YP3, USP9YP8, CDY11P, KDM5D, RBMY2AP, OFD1P8Y, HSFY5P, USP9YP17, CDY12P, TCEB1P15, TTTY6B, TSPY23P, RBMY2UP, RBMY1KP, TTTY5, TSPY22P, RBMY2FP, TTTY25P, TSPY21P, TTTY6, TCEB1P8, CDY13P, USP9YP24, HSFY7P, OFD1P9Y, RBMY2BP, RBMY2WP, TTTY17A, ZNF736P11Y, TTTY4, EIF1AY, TRIM60P8Y, ZNF736P3Y, TRIM60P9Y, PPP1R12BP2, REREP1Y, OFD1P10Y, HSFY6P, USP9YP18, CDY14P, RPS4Y2, CYorf17, RBMY1B, TCEB1P16, PRYP3, XKRYP3, USP9YP25, USP9YP29, RBMY1A1, RNU1-97P, RAB9AP1, TRAPPC2P9, OFD1P11Y, TCEB1P9, CDY15P, XKRYP4, USP9YP13, USP9YP11, USP9YP12, RNU1-86P, RAB9AP5, TTTY15, CDY4P, TRAPPC2P10, OFD1P12Y, TCEB1P10, CDY17P, USP9YP35, USP9YP31, CDY18P, GOLGA6L11P, DNM1P24, CSPG4P2Y, CSPG4P3Y, RBMY1E, GOLGA2P2Y, RN7SL818P, UBE2Q2P4Y, LINC00265-2P, CICP2, LINC00266-2P, RBMY2XP, CASKP1, TTTY17B, TRIM60P5Y, ZNF736P12Y, SFPQP1, TTTY4B, TAB3P1, DPPA2P1, PSMA6P1, KALP, TRIM60P10Y, ZNF736P2Y, TRIM60P11Y, PNPLA4P1, RBMY1D, PRY2, RBMY1F, RBMY1J, PRY, BPY2, DAZ1, DAZ2

Supplementary Table S7				
SCNA loss				
chrom	start	end	samples	genes
chr1	69090	1290532	M9	OR4F5, OR4F29, OR4F16, SAMD11, NOC2L, KLHL17, PLEKHN1, C1orf170, HES4, ISG15, AGRN, RNF223, C1orf159, TTLL10, TNFRSF18, TNFRSF4, SDF4, B3GALT6, FAM132A, UBE2J2, SCNN1D, ACAP3, PUSL1, CPSF3L, GLTPD1, TAS1R3, DVL1, MXRA8, CICP27, RNU6-1100P, CICP7, WBP1LP7, MTND1P23, MTND2P28, MTATP8P1, MTATP6P1, WBP1LP6, CICP3, RNU6-1199P, FAM87B, LINC00115, LINC01128, FAM41C, TUBB8P11, MIR200B, MIR200A, MIR429, TTLL10-AS1
chr1	1290623	2116137	M9	MXRA8, AURKAIP1, CCNL2, MRPL20, ANKRD65, TMEM88B, VWA1, ATAD3C, ATAD3B, ATAD3A, TMEM240, SSU72, C1orf233, MIB2, MMP23B, CDK11B, SLC35E2B, CDK11A, SLC35E2, NADK, GNB1, CALML6, TMEM52, C1orf222, GABRD, PRKCZ, C1orf86, RN7SL657P, MMP23A
chr1	2116360	3670805	M9	PRKCZ, C1orf86, SKI, MORN1, RER1, PEX10, PLCH2, PANK4, HES5, TNFRSF14, FAM213B, MMEL1, TTC34, ACTRT2, PRDM16, ARHGEF16, MEGF6, TPRG1L, WRAP73, TP73, CCDC27, LINC00982, MIR551A, TP73-AS1
chr1	3672020	17961420	M9	HNRNPCP5, RNU6-771P, RNA5SP41, RNU6-1265P, TBCAP2, TMEM51-AS1, CHCHD2P6, RNU7-179P, TBC1D3P6, ARHGEF19-AS1, ANO7P1, MT1XP1, CROCCP3, RNU1-1, CROCCP2, MST1P2, RNU1-3, EIF1AXP1, ESPNP, RNU1-4, CROCCP4, MST1L, MIR3675, RNU1-2, MIR3972, PADI6, CCDC27, SMIM1, LRRC47, CEP104, DFFB, C1orf174, AJAP1, NPHP4, KCNAB2, CHD5, RPL22, RNF207, ICMT, HES3, GPR153, ACOT7, HES2, ESPN, TNFRSF25, PLEKHG5, NOL9, TAS1R1, ZBTB48, KLHL21, PHF13, THAP3, DNAJC11, CAMTA1, VAMP3, PER3, UTS2, TNFRSF9, PARK7, ERRFI1, SLC45A1, RERE, ENO1, CA6, SLC2A7, SLC2A5, GPR157, H6PD, SPSB1, SLC25A33, TMEM201, PIK3CD, C1orf200, CLSTN1, CTNNBIP1, LZIC, NMNAT1, RBP7, UBE4B, RN7SL574P, LINC01134, KIF1B, MIR4417, MIR4689, LINC00337, MIR4252, RNU6-731P, RNU1-8P, CAMTA1-IT1, RNU1-7P, RN7SL729P, RNU6-991P, RPL7P11, RPL7P7, ENO1-IT1, ENO1-AS1, RNU6-304P, HMGN2P17, RN7SL451P, MIR34A, RNA5SP40, RN7SKP269, MIR5697, PGAM1P11, RNU6-828P, MIR1273D, RNU6-37P, RN7SL731P, RN7SL721P, RN7SL614P, HSPE1P24, CFL1P6, MTOR-AS1, RNU6-537P, RNU6-291P, RPL39P6, UBE2V2P3, NPPA-AS1, RNU5E-1, RNU5E-4P, RN7SL649P, RNU6-777P, MIR4632, SNORA59A, RNU6ATAC18P, RNU6-1072P, PRAMEF23, PRAMEF25, PGD, APITD1, APITD1-CORT, CORT, DFFA, PEX14, CASZ1, C1orf127, TARDBP, MASP2, SRM, EXOSC10, MTOR, ANGPTL7, UBIAD1, PTCHD2, FBXO2, FBXO44, FBXO6, MAD2L2, DRAXIN, AGTRAP, C1orf167, MTHFR, CLCN6, NPPA, NPPB, KIAA2013, PLOD1, MFN2, MIIP, TNFRSF8, TNFRSF1B, VPS13D, DHRS3, AADACL4, AADACL3, C1orf158, PRAMEF12, PRAMEF1, PRAMEF11, HNRNPCL1, PRAMEF2, PRAMEF4, PRAMEF10, PRAMEF7, PRAMEF6, PRAMEF22, PRAMEF26, PRAMEF3, PRAMEF5, PRAMEF8, PRAMEF9, PRAMEF13, PRAMEF18, PRAMEF16, PRAMEF21, PRAMEF15, PRAMEF14, PRAMEF19, PRAMEF17, PRAMEF20, LRRC38, PDPN, PRDM2, KAZN, TMEM51, C1orf195, FHAD1, EFHD2, CTRC, CELA2A, CELA2B, CASP9, DNAJC16, AGMAT, DDI2, RSC1A1, PLEKHM2, SLC25A34, TMEM82, FBLIM1, SPEN, ZBTB17, C1orf64, HSPB7, CLCNKA, CLCNKB, FAM131C, EPHA2, ARHGEF19, C1orf134, RSG1, FBXO42, SZRD1, SPATA21, NECAP2, FAM231B, NBPF1, FAM231A, FAM231C, CROCC, MFAP2, ATP13A2, SDHB, PADI2, PADI1, PADI3, PADI4, RCC2, ARHGEF10L
chr1	17961420	19168284	M14,M9	ARHGEF10L, ACTL8, IGSF21, KLHDC7A, PAX7, TAS1R2
chr1	19168284	28599091	M9	USP48, MIR4695, MIR1290, RNU6-1099P, RN7SL85P, RN7SL277P, RNU4-28P, RPS14P3, RN7SL304P, UBXN10-AS1, LINC01141, RPS4XP4, PINK1-AS, RNU7-200P, MIR1256, RPS15AP6, HSPE1P27, PPP1R11P1, NBPF2P, HS6ST1P1, CROCCP5, PFN1P10, RN7SL421P, RN7SL386P, RNU6-1022P, RN7SL768P, RN7SL186P, RNU6-776P, LINC00339, CDC42-IT1, MIR4418, ZBTB40-IT1, MIR4684, MIR4253, MIR3115, MIR4419A, RNU6-514P, RNU6-135P, RN7SL532P, RN7SL24P, MIR378F, RN7SL857P, RNU6-1208P, MIR4425, SDHDP6, RNU6-1171P, SNRPFP2, MIR3917, RNU6-110P, RN7SL490P, DPPA2P2, MIR1976, RN7SL679P, RN7SL501P, RN7SL165P, OSTCP2, CHCHD3P3, NPM1P39, SNRPEP7, RNU6-48P, RNU6-949P, CHMP1AP1, RNU6-424P, RPEP3, RNU6-1245P, SCARNA1, RN7SL559P, SPCS2P4, RNU6-176P, RNU7-29P, LDLRAD2, HSPG2, CELA3B, CELA3A, CDC42, WNT4, ZBTB40, EPHA8, C1QA, C1QC, C1QB, EPHB2, LACTBL1, C1orf234, KDM1A, LUZP1, HTR1D, HNRNPR, ZNF436, C1orf213, TCEA3, ASAP3, E2F2, ID3, MDS2, RPL11, TCEB3, PITHD1, LYPLA2, GALE, HMGCL, FUCA1, CNR2, PNRC2, SRSF10, MYOM3, IL22RA1, IFNLR1, GRHL3, STPG1, NIPAL3, RCAN3, NCMAP, SRRM1, CLIC4, RUNX3, SYF2, C1orf63, RHD, TMEM50A, RHCE, TMEM57, LDLRAP1, MAN1C1, SEPN1, MTFR1L, AUNIP, PAQR7, STMN1, PAFAH2, EXTL1, SLC30A2, TRIM63, PDIK1L, FAM110D, ZNF593, CNKSR1, CATSPER4, CEP85, SH3BGRL3, UBXN11, CD52, AIM1L, ZNF683, LIN28A, DHDDS, HMGN2, RPS6KA1, ARID1A, PIGV, ZDHHC18, SFN, GPN2, GPATCH3, NUDC, NR0B2, C1orf172, TRNP1, FAM46B, SLC9A1, WDTC1, TMEM222, SYTL1, MAP3K6, FCN3, CD164L2, GPR3, WASF2, AHDC1, FGR, IFI6, FAM76A, STX12, PPP1R8, THEMIS2, RPA2, SMPDL3B, XKR8, EYA3, PTAFR, DNAJC8, ATPIF1, SESN2, TAS1R2, ALDH4A1, IFFO2, UBR4, EMC1, MRTO4, AKR7L, AKR7A3, AKR7A2, PQLC2, CAPZB, MINOS1, MINOS1-NBL1, NBL1, HTR6, TMCO4, RNF186, OTUD3, PLA2G2E, PLA2G2A, PLA2G5, PLA2G2D, PLA2G2F, PLA2G2C, UBXN10, VWA5B1, CAMK2N1, MUL1, FAM43B, CDA, PINK1, DDOST, KIF17, SH2D5, HP1BP3, EIF4G3, ECE1, NBPF3, ALPL, RAP1GAP
chr1	28599091	29528557	M16,M9	RNU6ATAC27P, SNHG3, SNORA73B, PRDX3P2, SNHG12, SNORD99, RNU11, SESN2, MED18, PHACTR4, RCC1, TRNAU1AP, TAF12, RAB42, GMEB1, YTHDF2, OPRD1, EPB41, TMEM200B, SRSF4, MECR
chr1	29528557	45111081	M9	CCDC24, C1orf50, CAP1, CCDC23, SLC6A9, ERMAP, ZNF691, SLC2A1, PPT1, FAM183A, KLF17, EBNA1BP2, DMAP1, RLF, TMCO2, WDR65, ZMPSTE24, COL9A2, TMEM125, ERI3, C1orf210, TIE1, MATN1-AS1, MIR4420, RN7SKP91, SNORD103A, SNORD103B, SNORD85, SEPW1P, RNU6-40P, MIR4254, MIR5585, MTMR9LP, MPL, GAPDHP20, LRRC37A12P, RNF220, RN7SL122P, MIR3605, RN7SKP16, TLR12P, HSPD1P14, SMAP2, CDC20, RNA5SP42, MIR552, EFCAB14P1, RN7SL136P, RN7SL503P, RPL5P4, ZMYM4-AS1, RNY5P1, RN7SL281P, RN7SL131P, UBE2V2P4, RNU4-27P, FTLP18, MIR4255, RNU6-636P, RNA5SP43, RPS29P6, ELOVL1, LINC01137, MIR5581, FTH1P1, ACTN4P2, RNU6-584P, RNU6-510P, MIR3659, RNU6-753P, RNU6-605P, RNU6-608P, RNA5SP44, HSPE1P8, OXCT2P1, ZFP69B, PPIEL, SNORA55, RNU7-121P, ZFP69, RNU6-1237P, GTF2F2P2, MED8, NFYC-AS1, MIR30E, MIR30C1, EXO5, RN7SL326P, TMEM53, UBE2V1P8, RNA5SP45, HNRNPFP1, TMSB4XP1, RNU6-536P, ZNF684, ATP6V1E1P1, SLC2A1-AS1, RNU6-880P, RNU6-870P, RNA5SP46, SZT2, SZT2-AS1, HYI-AS1, KDM4A-AS1, RNU6-1058P, SHMT1P1, RIMS3, KRT8P47, RN7SL479P, OOSP1P1, ERI3-IT1, RNU6-369P, MIR5584, NFYC, HYI, KCNQ4, PTPRF, CITED4, CTPS1, SLFNL1, SCMH1, KDM4A, FOXO6, EDN2, HIVEP3, GUCA2B, GUCA2A, FOXJ3, RIMKLA, ZMYND12, PPCS, ST3GAL3, CCDC30, PPIH, YBX1, CLDN19, LEPRE1, ARTN, IPO13, DPH2, ATP6V0B, B4GALT2, MECR, PTPRU, MATN1, LAPTM5, SDC3, PUM1, NKAIN1, SNRNP40, ZCCHC17, FABP3, SERINC2, TINAGL1, HCRTR1, PEF1, COL16A1, BAI2, SPOCD1, PTP4A2, KHDRBS1, TMEM39B, KPNA6, TXLNA, CCDC28B, IQCC, DCDC2B, TMEM234, EIF3I, FAM167B, LCK, HDAC1, MARCKSL1, TSSK3, FAM229A, BSDC1, ZBTB8B, ZBTB8A, ZBTB8OS, RBBP4, SYNC, KIAA1522, YARS, S100PBP, FNDC5, HPCA, TMEM54, RNF19B, AK2, ADC, TRIM62, ZNF362, A3GALT2, PHC2, ZSCAN20, CSMD2, HMGB4, C1orf94, SMIM12, GJB5, GJB4, GJB3, GJA4, DLGAP3, ZMYM6NB, ZMYM6, ZMYM1, SFPQ, ZMYM4, KIAA0319L, NCDN, TFAP2E, PSMB2, C1orf216, CLSPN, AGO4, AGO1, AGO3, TEKT2, ADPRHL2, COL8A2, TRAPPC3, MAP7D1, THRAP3, SH3D21, EVA1B, STK40, LSM10, OSCP1, MRPS15, CSF3R, GRIK3, ZC3H12A, MEAF6, SNIP1, DNALI1, GNL2, RSPO1, C1orf109, CDCA8, EPHA10, MANEAL, YRDC, C1orf122, MTF1, INPP5B, SF3A3, FHL3, UTP11L, POU3F1, RRAGC, MYCBP, GJA9, RHBDL2, AKIRIN1, NDUFS5, MACF1, KIAA0754, BMP8A, PABPC4, HEYL, NT5C1A, HPCAL4, PPIE, BMP8B, OXCT2, TRIT1, MYCL, MFSD2A
chr1	45111081	45268825	M1,M9	RNF220, TMEM53, C1orf228, RNU5F-1, RNU5D-1, SNORD55, SNORD46, SNORD38A, SNORD38B, RPS15AP11, KIF2C, RPS8, BEST4, PLK3
chr1	45268825	55352921	M9	RNU6-1026P, RNU6-1253P, CCDC163P, PHBP12, MRPS6P2, MIR4421, CFL1P2, RNU6-877P, RNU6-1281P, CALR4P, GAPDHP51, SLC25A6P3, MMACHC, MIR761, TSEN15P2, RNA5SP48, RN7SL290P, TXNDC12-AS1, RN7SL788P, PDCL3P6, DNAJC19P7, PRDX1, ANAPC10P1, PLA2G12AP1, EEF1GP7, NDUFS5P3, RN7SL62P, RNU2-30P, RNU6-969P, MIR1273F, MIR5095, MIR1273G, TUBBP10, HIGD1AP11, SLC25A3P1, RNU7-95P, AKR1A1, HNRNPA3P12, MIR4781, HNRNPA1P63, NASP, CCDC17, GPBP1L1, TMEM69, IPP, MAST2, PIK3R3, TSPAN1, POMGNT1, RNU5E-6P, RNA5SP47, CCNB1IP1P1, MRPS17P1, OSTCP5, HMGB1P48, RPS15AP10, TMA16P2, LINC00505, MKNK1-AS1, EFCAB14-AS1, CYP4Z2P, CYP4A26P, CYP4A43P, CYP4A27P, CYP4A44P, CYP4A22-AS1, MTND1P34, LINC00853, FOXD2-AS1, RPL21P24, ATP6V0E1P4, CYP46A4P, SKINTL, PPP1R8P1, RNU6-723P, RNU4-61P, AGBL4-IT1, ZNF859P, MTND2P29, HMGB1P45, FCF1P6, LURAP1, RAD54L, LRRC41, UQCRH, NSUN4, FAAH, DMBX1, PLK3, KNCN, MKNK1, TCTEX1D4, BTBD19, PTCH2, MOB3C, ATPAF1, EIF2B3, EFCAB14, TEX38, CYP4B1, HECTD3, CYP4A11, CYP4X1, CYP4Z1, CYP4A22, UROD, PDZK1IP1, TAL1, STIL, CMPK1, FOXE3, FOXD2, TRABD2B, SLC5A9, ZSWIM5, HPDL, SPATA6, AGBL4, BEND5, MUTYH, ELAVL4, DMRTA2, FAF1, CDKN2C, C1orf185, RNF11, TTC39A, TOE1, EPS15, TESK2, OSBPL9, NRD1, RAB3B, TXNDC12, KTI12, BTF3L4, ZFYVE9, CC2D1B, ORC1, PRPF38A, ZCCHC11, GPX7, FAM159A, COA7, ZYG11B, ZYG11A, ECHDC2, SCP2, PODN, SLC1A7, CPT2, C1orf123, MAGOH, LRP8, DMRTB1, GLIS1, NDC1, YIPF1, DIO1, HSPB11, LRRC42, LDLRAD1, TMEM59, TCEANC2, CDCP2, CYB5RL, MRPL37, SSBP3, SSBP3-AS1, ACOT11, FAM151A, MROH7, MROH7-TTC4, TTC4, PARS2, TTC22, C1orf177, DHCR24
chr1	55446464	66827386	M9	GYG1P3, MIR4422, GOT2P1, RN7SKP291, RNU6-830P, PIGQP1, RPL21P23, RPS20P5, DAB1-AS1, HNRNPA1P6, RPS26P15, RN7SL713P, LINC01135, PHBP3, MIR4711, RN7SL475P, PGBD4P8, RNU6-414P, RNU6-1177P, LAMTOR5P1, RN7SL180P, MIR3116-2, MIR3116-1, PIGPP2, RPS15AP7, RNU6-371P, RNA5SP49, LINC00466, RN7SL488P, RNU7-123P, RN7SL130P, CFL1P3, RNU6-809P, RNU7-62P, MIR4794, SLC2A3P2, RNU6-1176P, MIR3671, MIR101-1, MRPS21P1, COX6CP13, RNU2-15P, RN7SL854P, RNU4-88P, TMEM61, BSND, PCSK9, USP24, PPAP2B, PRKAA2, C1orf168, C8A, C8B, DAB1, OMA1, TACSTD2, MYSM1, JUN, FGGY, HOOK1, CYP2J2, C1orf87, NFIA, TM2D1, INADL, L1TD1, KANK4, USP1, DOCK7, ANGPTL3, ATG4C, FOXD3, ALG6, ITGB3BP, EFCAB7, DLEU2L, PGM1, ROR1, UBE2U, CACHD1, RAVER2, JAK1, AK4, DNAJC6, LEPR, LEPROT, PDE4B

chr1	66827386	103347321	M14,M9	MIR3117, RNU6-586P, RNU4ATAC4P, RNU6-387P, RNU6-1031P, RN7SL392P, HNRNPCP9, RNU7-80P, GNG12-AS1, ARL5AP3, CTBP2P8, MIR1262, RPS7P4, COX6B1P7, TCEB1P18, TXNP2, RN7SL538P, PIN1P1, RN7SL242P, CASP3P1, ZRANB2-AS1, MIR186, ZRANB2-AS2, NEGR1-IT1, GDI2P2, RPL31P12, RNU6-1246P, KRT8P21, RN7SKP19, RNA5SP50, RNU4ATAC8P, RNU6-622P, RNU6-503P, SNORD45C, SNORD45A, SNORD45B, TPI1P1, RNU6-161P, RNU7-8P, RNA5SP20, RN7SL370P, RNA5SP21, NSRP1P1, HSPE1P25, NEXN-AS1, RNU6-1102P, RNA5SP22, RNFT1P2, RNA5SP23, PSAT1P3, ADH5P2, HMGB1P18, HNRNPA1P64, MTND2P30, RPL7P10, RN7SKP247, HNRNPA3P14, MED28P8, ST13P20, TTLL7-IT1, UOX, SPATA1, MIR4423, RNA5SP51, CLCA3P, LINC01140, RNA5SP52, RN7SL583P, RPL36AP10, RNU6-125P, TCEB1P19, PTGES3P1, CAPNS1P1, GBP1P1, RN7SKP272, GEMIN8P4, RNU6-695P, PHKA1P1, RPL5P6, FEN1P1, HSP90B3P, RN7SL653P, RN7SL235P, LPCAT2BP, SETSIP, GAPDHP46, PRKAR1AP, ACTBP12, RN7SL824P, HMGB3P9, RNU4-59P, CCNJP2, SNORD21, SNORA66, RNU6-970P, RN7SL692P, RNU6-210P, RN7SKP123, RNA5SP53, MIR760, CHCHD2P5, MTND4P11, RN7SL440P, GAPDHP29, LINC01057, KATNBL1P2, PGBD4P7, MIR378G, RNU1-130P, UBE2WP1, EEF1A1P11, RN7SL831P, NDUFS5P2, RPL7P9, RN7SKP270, DPYD-AS1, DPYD-IT1, SEC63P1, RPL26P9, DPYD-AS2, MIR137HG, NFU1P2, HMGB3P10, RNU4-75P, RNU6-750P, RNU6-1318P, BRI3P1, MIR553, BCAS2P2, HNRNPA1P68, PPIAP7, RNU6-965P, RNU6-352P, DNAJA1P5, PDE4B, SGIP1, TCTEX1D1, INSL5, WDR78, MIER1, SLC35D1, C1orf141, IL23R, IL12RB2, SERBP1, GADD45A, GNG12, DIRAS3, WLS, RPE65, DEPDC1, LRRC7, LRRC40, SRSF11, ANKRD13C, HHLA3, CTH, PTGER3, ZRANB2, NEGR1, LRRIQ3, FPGT, FPGT-TNNI3K, TNNI3K, LRRC53, C1orf173, CRYZ, TYW3, LHX8, SLC44A5, ACADM, RABGGTB, MSH4, ASB17, ST6GALNAC3, ST6GALNAC5, PIGK, AK5, ZZZ3, USP33, FAM73A, NEXN, FUBP1, DNAJB4, GIPC2, PTGFR, IFI44L, IFI44, ELTD1, LPHN2, TTLL7, PRKACB, SAMD13, DNASE2B, RPF1, GNG5, CTBS, C1orf180, SSX2IP, LPAR3, MCOLN2, WDR63, MCOLN3, SYDE2, C1orf52, BCL10, DDAH1, CYR61, ZNHIT6, COL24A1, ODF2L, CLCA2, CLCA1, CLCA4, SH3GLB1, HS2ST1, LMO4, PKN2, GTF2B, CCBL2, RBMXL1, GBP3, GBP1, GBP2, GBP7, GBP4, GBP5, GBP6, LRRC8B, LRRC8C, LRRC8D, ZNF326, BARHL2, ZNF644, HFM1, CDC7, TGFBR3, BRDT, EPHX4, BTBD8, KIAA1107, C1orf146, GLMN, RPAP2, GFI1, EVI5, RPL5, FAM69A, MTF2, TMED5, CCDC18, DR1, FNBP1L, BCAR3, DNTTIP2, GCLM, ABCA4, ARHGAP29, ABCD3, F3, SLC44A3, CNN3, ALG14, TMEM56, TMEM56-RWDD3, RWDD3, PTBP2, DPYD, SNX7, PALMD, FRRS1, AGL, SLC35A3, HIAT1, SASS6, TRMT13, LRRC39, DBT, RTCA, CDC14A, GPR88, VCAM1, EXTL2, SLC30A7, DPH5, S1PR1, OLFM3, COL11A1
chr1	103347321	103354159	M14	COL11A1
chr1	103481223	107691075	M9	RN7SKP285, ACTG1P4, AMYP1, FTLP17, SEPT2P1, SOD2P1, COL11A1, RNPC3, AMY2B, AMY2A, AMY1A, AMY1B, AMY1C, PRMT6, NTNG1
chr1	107691075	115836309	M14,M9	VAV3-AS1, SLC25A24P1, NBPF5P, SLC25A24P2, ST13P21, SPATA42, RANP5, SCARNA2, RNU7-122P, RNU6V, MIR197, RPL7P8, NDUFA5P10, KCNC4-AS1, LAMTOR5-AS1, CYMP, NRBF2P3, OR11I1P, CCNT2P1, RNA5SP54, CHIAP1, CHIAP2, CHIAP3, HIGD1AP12, PGCP1, UBE2FP3, RNU6-792P, LINC01160, KRT18P57, RNU6-151P, FAM212B-AS1, KCND3-IT1, KCND3-AS1, TXNP3, MRPL53P1, RNU7-70P, AKR7A2P1, SLC16A1-AS1, RLIMP2, MTND5P20, AP4B1-AS1, EIF2S2P5, PKMP1, RN7SL432P, NR1H5P, NTNG1, VAV3, SLC25A24, NBPF4, NBPF6, FAM102B, HENMT1, PRPF38B, FNDC7, STXBP3, AKNAD1, GPSM2, CLCC1, WDR47, TAF13, TMEM167B, C1orf194, KIAA1324, SARS, CELSR2, PSRC1, MYBPHL, SORT1, PSMA5, SYPL2, ATXN7L2, CYB561D1, AMIGO1, GPR61, GNAI3, GNAT2, AMPD2, GSTM4, GSTM2, GSTM1, GSTM5, GSTM3, EPS8L3, CSF1, AHCYL1, STRIP1, ALX3, UBL4B, SLC6A17, KCNC4, RBM15, SLC16A4, LAMTOR5, PROK1, KCNA10, KCNA2, KCNA3, CD53, LRIF1, DRAM2, CEPT1, DENND2D, CHI3L2, CHIA, PIFO, OVGP1, WDR77, ATP5F1, C1orf162, ADORA3, RAP1A, FAM212B, DDX20, KCND3, CTTNBP2NL, WNT2B, ST7L, CAPZA1, MOV10, RHOC, PPM1J, FAM19A3, SLC16A1, LRIG2, MAGI3, PHTF1, RSBN1, PTPN22, BCL2L15, AP4B1, DCLRE1B, HIPK1, OLFML3, SYT6, TRIM33, BCAS2, DENND2C, AMPD1, NRAS, CSDE1, SIKE1, SYCP1, TSHB, TSPAN2, NGF
chr1	115836309	121313686	M9	TCEB1P20, CNOT7P2, RN7SL420P, HNRNPA1P43, ATP1A1OS, RNU6-817P, NAP1L4P1, MIR548AC, MIR320B1, GAPDHP64, NEFHP1, FTH1P22, RNA5SP55, MIR942, RPS15AP9, VPS25P1, RNA5SP56, PSMC1P12, WARS2-IT1, RBMX2P3, RPS3AP12, RPL6P2, RNU1-75P, HAO2-IT1, HSD3BP2, GAPDHP23, HSD3BP1, GAPDHP58, GAPDHP32, HSD3BP3, GAPDHP27, HSD3BP4, GAPDHP33, LINC00622, HSD3BP5, NBPF7, PFN1P9, NOTCH2P1, RNU6-465P, HIST2H3DP1, HIST2H2BA, SRGAP2C, EMBP1, NGF, VANGL1, CASQ2, NHLH2, SLC22A15, MAB21L3, ATP1A1, CD58, IGSF3, C1orf137, CD2, PTGFRN, CD101, TTF2, TRIM45, VTCN1, MAN1A2, FAM46C, GDAP2, WDR3, SPAG17, TBX15, WARS2, HAO2, HSD3B2, HSD3B1, ZNF697, PHGDH, HMGCS2, REG4, ADAM30, NOTCH2, FAM72B, FCGR1B
chr1	245775186	249212072	M14	OR9H1P, HSD17B7P1, OR6R1P, OR2X1P, OR2L9P, OR2L1P, OR2L6P, OR2T32P, OR2M1P, OR2AS2P, OR2AS1P, AHCYP8, MIR3124, RNU6-1205P, CHCHD4P5, RNU6-1283P, SMYD3-IT1, KIF28P, FGFR3P6, MIR3916, RNA5SP82, VN1R17P, VN1R5, OR2W5, GCSAML-AS1, RNU6-691P, OR14L1P, OR3D1P, KIF26B, SMYD3, TFB2M, CNST, SCCPDH, AHCTF1, ZNF670, ZNF695, ZNF669, C1orf229, ZNF124, ZNF496, NLRP3, OR2B11, GCSAML, OR2C3, OR2G2, OR2G3, OR13G1, OR6F1, OR14A2, OR14K1, OR1C1, OR14A16, OR11L1, TRIM58, OR2W3, OR2T8, OR2AJ1, OR2L13, OR2L8, OR2AK2, OR2L5, OR2L2, OR2L3, OR2M5, OR2M2, OR2M3, OR2M4, OR2T33, OR2T12, OR2M7, OR14C36, OR2T4, OR2T6, OR2T1, OR2T7, OR2T2, OR2T3, OR2T5, OR2G6, OR2T29, OR2T34, OR2T10, OR2T11, OR2T35, OR2T27, OR14I1, LYPD8, SH3BP5L, ZNF672, ZNF692, PGBD2
chr10	93440	42827314	M14	PTCHD3, RAB18, SFMBT2, DDX10P1, ZEB1-AS1, RNA5SP309, SPTLC1P1, HMGB1P7, ARMC3, RN7SL825P, RPS4XP11, RNU7-22P, PPIAP31, RPS24P13, RNU6-1244P, C1DP1, AK3P5, CCNY, RN7SL847P, RN7SL398P, LINC00838, RPL23P11, TCEB2P4, PARD3-AS1, SS18L2P1, RNU6-847P, RNU6-193P, PRDX2P2, MIR3611, ITIH5, RNU7-77P, ATP6V1G1P4, RNU6-794P, RNU6-1167P, MIR4683, RPL7P37, MTND5P17, MKX, GJD4, FZD8, MSRB2, ARMC4, MTND4P18, NAMPTL, MKNK2P1, ARL6IP1P2, RNU6-811P, ATP8A2P1, RN7SL314P, ANKRD30A, LINC00993, EPC1, PTF1A, C10orf115, C10orf67, TMEM161BP1, VN1R53P, TACC1P1, MTND1P18, ZNF33BP1, MTRNR2L7, ZNF248, ZNF33AP1, RNU6-795P, EIF3LP3, FXYD6P2, HSD17B7P2, OTUD1, MPP7, KIAA1217, SEPT7P9, ZNF25, RNU6-1118P, CICP9, ABCD1P2, PABPC1P12, ZNF33A, SLC9B1P3, CCDC7, ACTR3BP5, CHEK2P5, KSR1P1, IGKV1OR10-1, PABPC1P8, ZNF37A, WAC, LINC00999, ARHGAP21, ITGB1, PRTFDC1, ENKUR, BAMBI, C10orf126, LYZL1, THNSL1, GPR158, ITIH2, SVIL, MYO3A, NRP1, GAD2, KIN, APBB1IP, KIAA1462, PDSS1, ATP5C1, MTPAP, PARD3, ABI1, MAP3K8, LYZL2, ZNF438, ZEB1, ANKRD26, TAF3, GATA3, YME1L1, ARHGAP12, CELF2, KIF5B, IL9RP2, RNA5SP297, RNA5SP298, RN7SL754P, MIR5699, CUL2, IDI2-AS1, RNU7-163P, LINC00200, ADARB2-AS1, LINC00700, RNU6-889P, RNU6-576P, LINC00701, MASTL, PITRM1-AS1, LINC00702, LINC00703, RNU6-163P, LINC00704, LINC00705, AKR1C6P, ACBD5, CREM, USP6NL, AKR1C5P, AKR1C7P, CALML3-AS1, RN7SL445P, NRBF2P5, RN7SKP78, MIR3155A, SDCBPP1, PRKCQ-AS1, LINC00707, COX6CP17, RNU6-535P, GATA3-AS1, PRPF38AP1, LINC00708, KRT8P37, CHCHD3P1, RNA5SP299, ECHDC3, LINC00709, HSP90AB7P, TCEB1P3, CUX2P1, SFTA1P, PROSER2, LINC00710, CELF2-AS2, CELF2-AS1, PROSER2-AS1, RNU6-1095P, UPF2, MIR548AK, RN7SL232P, RN7SL198P, RNU6ATAC39P, MIR4480, MIR548Q, RNA5SP300, RPL5P25, SNRPGP5, BTBD7P1, RNU6-6P, RNA5SP301, MIR4293, MIR1265, RNA5SP302, OR7E110P, OR7E26P, OR7E115P, DCLRE1CP1, GAPDHP45, PPIAP30, FTLP19, RNU6-1075P, RNU2-18P, VIM-AS1, ST8SIA6-AS1, PRPF38AP2, MIR511-1, MIR511-2, SLC39A12-AS1, ARL5B-AS1, AIFM1P1, UBE2V2P1, HMGN1P20, RNA5SP303, MTND2P16, RNU6-1212P, AMD1P1, MIR4675, MTND1P21, EIF4BP2, NPM1P30, NEBL-AS1, LUZP4P1, RNU6-15P, RNMTL1P1, MIR1915, RNU6-306P, HNRNPRP1, RNU6-1141P, RN7SKP219, RN7SKP37, ADIPOR1P1, RNU6-413P, RNA5SP304, YWHAZP3, MIR1254-2, MIR603, RNA5SP305, RN7SKP241, GPR158-AS1, RN7SKP220, GPN3P1, HIRAP1, RNA5SP306, RNU6-632P, RNA5SP307, LINC00264, DHTKD1, LINC00202-2, HSPA8P3, RNU6-946P, LINC00202-1, LINC00614, RNU6-490P, RNU2-24P, RNU7-12P, LRRC37A6P, ARMC4P1, RNU6-666P, RNU6-452P, FAM210CP, TRIAP1P1, SEC61A2, TUBB8, RN7SKP132, RPL36AP55, ZNF101P1, RN7SKP39, WAC-AS1, RNU4ATAC6P, TPRKBP1, RNU6-1067P, RNU6-270P, LINC00837, RNA5SP308, PTCHD3P1, ZMYND11, MIR604, MIR938, RNU6-908P, RNU6-598P, EEF1A1P39, DNM1P17, GOLGA2P6, RN7SL241P, NIFKP1, HNRNPA1P32, RN7SL63P, SVILP1, NUDT5, DIP2C, PRR26, LARP4B, CDC123, GTPBP4, IDI2, IDI1, WDR37, ADARB2, CAMK1D, PFKP, CCDC3, PITRM1, OPTN, KLF6, AKR1E2, AKR1C1, MCM10, AKR1C2, AKR1C3, AKR1CL1, AKR1C4, UCMA, UCN3, PHYH, TUBAL3, NET1, CALML5, CALML3, ASB13, FAM208B, GDI2, ANKRD16, FBXO18, SEPHS1, IL15RA, BEND7, IL2RA, PRPF18, RBM17, PFKFB3, PRKCQ, FRMD4A, FAM107B, CDNF, HSPA14, SUV39H2, DCLRE1C, MEIG1, OLAH, ACBD7, C10orf111, RPP38, NMT2, FAM171A1, ITGA8, FAM188A, PTER, C1QL3, RSU1, CUBN, TRDMT1, VIM, ST8SIA6, PTPLA, STAM, TMEM236, MRC1, SLC39A12, CACNB2, NSUN6, ARL5B, MALRD1, PLXDC2, NEBL, C10orf113, CASC10, SKIDA1, MLLT10, DNAJC1, EBLN1, COMMD3, COMMD3-BMI1, BMI1, SPAG6, PIP4K2A
chr10	42827314	47756685	M11,M14	CCNYL2, LINC00839, ZNF37BP, FXYD6P1, EIF3LP2, RNU6-1170P, CUBNP1, RSU1P1, DUXAP3, RNU6-885P, MIR5100, ZNF33B, BMS1, RNU6ATAC11P, RNU7-193P, CAP1P2, ZNF32-AS3, ZNF32-AS1, ZNF32-AS2, UQCRHP3, TCEB1P30, HNRNPA3P1, LINC00619, LINC00840, LINC00841, RET, EIF2AP4, TMEM72-AS1, CEP164P1, DUXAP4, RSU1P2, CUBNP3, CSGALNACT2, ANKRD54P1, ANKRD30BP3, MIR3156-1, RNU6-1207P, CUBNP2, OR6D1P, CTGLF10P, FAM21FP, RASGEF1A, RNA5SP310, FRMPD2P2, FXYD4, HNRNPF, CTGLF8P, BMS1P1, RNA5SP311, GLUD1P7, DUSP8P3, CTSLP5, ZNF487, FAM35BP, RN7SL248P, RHEBP1, ZNF239, ZNF485, LINC00842, HNRNPA1P33, RNA5SP312, BMS1P2, GLUD1P8, ZNF32, DUSP8P4, CTSLP7, CXCL12, FAM35DP, RN7SL453P, RHEBP2, AHCYP1, ANTXRLP1, CTGLF11P, FAM25HP, TMEM72, RASSF4, C10orf10, C10orf25, ZNF22, OR13A1, ALOX5, MARCH8, ZFAND4, FAM21C, FAM25E, AGAP4, PTPN20A, SYT15, GPRIN2, NPY4R, ANXA8L1, FAM25B, AGAP10, ANTXRL, ANXA8L2
chr10	47756685	64572993	M11	GDF2, GDF10, PTPN20B, FAM25C, FRMPD2, MAPK8, ARHGAP22, WDFY4, SLC9A3P4, CTSLP2, GLUD1P6, DUSP8P2, BMS1P6, RNA5SP313, FRMPD2P1, BMS1P5, RNA5SP314, GLUD1P2, DUSP8P1, CTSLP3, FAM35EP, RN7SL527P, CTGLF12P, RNA5SP315, BMS1P7, PTPN20CP, ARHGAP22-IT1, MIR4294, FAM170B-AS1, C10orf71-AS1, FAM25D, LRRC18, VSTM4, RNA5SP316, FAM170B, C10orf128, C10orf71, PARGP1, LINC00843, RNA5SP317, SLC9A3P3, SLC9A3P1, DYNC1I2P1, RMRPP4, RNU7-107P, DRGX, ERCC6, NUTM2HP, CTSLP4, PGBD3, PGGT1BP1, CCDC58P2, ERCC6-PGBD3, MIR605, RSU1P3, PRKG1-AS1, PRKRIRP3, CHAT, SNRPEP8, RNA5SP318, RNU6-687P, NEFMP1, MIR548F1, GAPDHP21, MIR3924, MRPS35P3, TPT1P10, FAM133CP, RN7SKP196, LINC00844, TRAF6P1, MRPL50P4, LINC00948, SLC18A3, C10orf53, ARL4P, RNU2-72P, LINC00845, OGDHL, RN7SL591P, ALDH7A1P4, PARG, FAM21D, AGAP8, TIMM23B, AGAP7, MSMB, NCOA4, TIMM23, AGAP6, FAM21A, ASAH2, SGMS1, ASAH2B, A1CF, PRKG1, CSTF2T, DKK1, MBL2, PCDH15, ANXA8L2, FAM21B, MTRNR2L5, ZWINT, ASAH2C, IPMK, AGAP9, FAM25G, ANXA8, CISD1, UBE2D1, ZNF488, RBP3, TFAM, BICC1, PHYHIPL, FAM13C, SLC16A9, CCDC6, C10orf40, ANK3, CDK1, RHOBTB1, TMEM26, C10orf107, ARID5B, RTKN2, ZNF365, ADO, EGR2
chr10	64572993	69909868	M11,M14	RNU6-543P, TATDN1P1, MIR1296, PRELID1P3, JMJD1C-AS1, MRPL35P2, DBF4P1, RPL17P35, CYP2C61P, ANXA2P3, NEK4P3, MYL6P3, AKR1B10P1, RNU6-1250P, RN7SL394P, DNAJC19P1, RPL12P8, RN7SL220P, POU5F1P5, EGR2, NRBF2, JMJD1C, REEP3, CTNNA3, LRRTM3, DNAJC12, SIRT1, HERC4, MYPN
chr10	69909868	69918242	M14	RN7SKP202, MYPN
chr10	69918242	71265996	M11,M14	DDX21, KIAA1279, SRGN, VPS26A, SUPV3L1, HKDC1, HK1, TACR2, TSPAN15, KRT19P4, RNA5SP319, TMEM14D, COX20P1, SNORD98, MIR1254-1, RNU6-697P, RNU6-571P, RN7SL373P, MED28P1, ACTBP14, ATP5G1P7, MYPN, ATOH7, PBLD, HNRNPH3, RUFY2, DNA2, SLC25A16, TET1, CCAR1, STOX1, DDX50
chr10	71265996	71266584	M14	TSPAN15

chr10	71266584	103543170	M11,M14	BTAF1, EIF4A2P2, DNAJC9-AS1, RNU6-833P, ANKRD22, LZTS2, STAMBPL1, GRID1, RNU6-883P, WAPAL, PDZD7, RNA5SP320, BMS1P4, GLUD1P3, DUSP8P5, POLR3A, ACTA2, CPEB3, RMRPP1, ZSWIM8-AS1, MARCH5, TIMM9P1, RPSAP6, RAB5CP1, MRPL35P3, POLR3DP1, SFXN3, PPIAP13, RPL39P25, ZNF503-AS1, IDE, HMGA1P5, KAZALD1, SPA17P1, ZNF503-AS2, RPS24, TLX1NB, TLX1, PLAU, LBX1, BTRC, MIR606, RN7SL518P, RNU6-673P, ATP5G1P8, FAS, COX6CP15, RNA5SP321, RNU6-1266P, OPN4, DPCD, RN7SL284P, DLG5-AS1, H2AFZP5, C10orf55, LDB3, GNAI2P2, LINC00856, VCL, LINC00595, POLL, ZMIZ1, ZMIZ1-AS1, KIF11, TPRX1P1, RPS12P18, MBL3P, AP3M1, HHEX, ADK, EXOC6, SFTPA3P, PPIF, BMPR1A, BEND3P3, NPAP1P2, CTSLP6, CYP26C1, PGGT1BP2, MBL1P, CYP26A1, ZNRF2P3, C1DP3, ZCCHC24, C1DP2, C1DP4, TMEM254-AS1, RPL22P18, LINC00857, RPS12P2, EIF5AP4, EIF5AL1, SFTPA2, MMRN2, CH25H, RPS7P9, FBXW4, LIPA, SFTPA1, KAT6B, FARSBP1, WARS2P1, RPA2P2, MYOF, NRG3-AS1, RNU6-441P, RNU6-478P, MARK2P15, RNU6-129P, SNCG, DUPD1, ADIRF, DUSP13, FAM25A, RNU1-65P, HMGN2P8, GLUD1, IFIT2, LINC00858, RPL12P29, TNPO1P1, CACYBPP1, RPS3AP5, RNU6-325P, RN7SKP84, NUTM2B, IFIT3, FGF8, RN7SKP238, RNA5SP322, MIR346, RNU6-780P, NUTM2E, RPL7AP8, TSPAN15, IFIT1B, IFIT1, RNU1-19P, ADIRF-AS1, IFIT5, SFTPD, BMS1P3, NPM3, AGAP11, RNU6-529P, RN7SL733P, NUTM2A-AS1, TMEM254, SLC16A12, LINC00863, SAMD8, NEUROG3, C10orf35, PANK1, FAM35A, NPAP1P3, CTSLP1, LINC00864, CFL1P1, RN7SL78P, NUTM2A, COL13A1, MED6P1, RPL7P34, KRT8P38, PLAC9, VDAC2, RCBTB2P1, PTCD2P2, KIF20B, ACTA2-AS1, MIR4679-2, MIR4679-1, NUTM2D, CEP55, FFAR4, MINPP1, RBP4, IFIT6P, PDE6C, MIR107, LINC00865, ANXA11, SNRPD2P1, RN7SKP143, RNU6-740P, LINC00502, DDX18P6, RPS27P1, GAPDHP28, FAF2P1, PAPSS2, FRA10AC1, H2AFY2, TNKS2-AS1, SRP9P1, SDHCP2, EIF4A1P8, AIFM2, ATAD1, NHP2P1, MARK2P9, RN7SL644P, EIF2S2P3, NIP7P1, XRCC6P1, RNA5SP323, RAB11AP1, PIPSL, RNY4P26, TYSND1, HDAC1P1, RNU6-657P, PLCE1-AS1, COMTD1, CTBP2P2, MTND4P19, CYP2C58P, SAR1A, MTND4P20, CYP2C59P, CYP2C60P, KLLN, ZNF503, PTEN, PAWRP1, ENTPD1-AS1, PPA1, C10orf11, MIR3157, RNU6-271P, ZNF518A, LGI1, MAT1A, NPM1P25, NPM1P26, RNA5SP324, RNU6-1274P, RPL13AP5, MIR607, HMGN2P35, HTR7, NPFFR1, DYDC1, RNLS, DYDC2, SLC35G1, LIPJ, LINC00866, MIR1287, MIR4685, ARL5AP2, RPL7P36, RPP30, LIPF, PLCE1, EBAG9P1, LRRC20, KCNMA1, EIF4EBP2, NODAL, PALD1, NANOGP6, DNMBP-AS1, PRF1, FAM213A, LIPK, TPM4P1, SNORA12, PHBP9, RNU6-422P, LINC00263, ADAMTS14, MIR608, LBX1-AS1, ANKRD1, TBATA, LIPN, RNU2-43P, SGPL1, RNU2-59P, MIR3158-2, MIR3158-1, LIPM, TSPAN14, PCGF5, NOC3L, PCBD1, RNU6-1165P, UNC5B, HECTD2, SLC29A3, CDH23, SH2D4B, TBC1D12, NRG3, PPP1R3C, TNKS2, HELLS, GHITM, FGFBP3, C10orf99, CDHR1, DLG5, TMEM256P1, MTND2P15, MTND1P20, C10orf105, C10orf54, RPL5P26, CALM2P2, CEP57L1P1, YY1P1, UNC5B-AS1, CDH23-AS1, RNU7-38P, RNU6-805P, HMGN2P34, LRIT2, LRIT1, MIR4676, NPM1P24, RGR, RPL17P50, PSAP, CCSER2, CYP2C18, CYP2C19, CHST3, SPOCK2, CYP2C9, ASCC1, CYP2C8, ANAPC16, C10orf129, DDIT4, DNAJB12, PDLIM1, MICU1, SORBS1, MCU, OIT3, PLA2G12B, P4HA1, NUDT13, ECD, ALDH18A1, FAM149B1, TCTN3, DNAJC9, MRPS16, TTC18, ENTPD1, ANXA7, C10orf131, CC2D2B, CCNJ, BLNK, MSS51, PPP3CB, DNTT, OPALIN, USP54, TLL2, TM9SF3, MYOZ1, SYNPO2L, AGAP5, SEC24C, PIK3AP1, FUT11, CHCHD1, ZSWIM8, LCOR, C10orf12, ARHGAP19-SLIT1, NDST2, SLIT1, CAMK2G, ARHGAP19, FRAT1, FRAT2, RRP12, PGAM1, EXOSC1, ZDHHC16, MMS19, UBTD1, ANKRD2, HOGA1, PI4K2A, C10orf62, MORN4, AVPI1, MARVELD1, ZFYVE27, SFRP5, GOLGA7B, CRTAC1, R3HCC1L, LOXL4, PYROXD2, HPS1, HPSE2, CNNM1, GOT1, NKX2-3, SLC25A28, ENTPD7, CUTC, COX15, ABCC2, DNMBP, CPN1, ERLIN1, CHUK, CWF19L1, BLOC1S2, PKD2L1, SCD, WNT8B, SEC31B, NDUFB8, HIF1AN, PAX2, FAM178A, MRPL43, SEMA4G, C10orf2
chr10	103543170	103544199	M14	NPM3
chr10	103544199	105344982	M11,M14	MGEA5, KCNIP2, C10orf76, HPS6, LDB1, PPRC1, NOLC1, KCNIP2-AS1, MIR146B, ELOVL3, PITX3, GBF1, RN7SL21P, RNU6-43P, RNU6-1231P, CYP17A1-AS1, PFN1P11, PTGES3P4, RNU6-372P, MARCKSL1P1, ST13P13, RNU11-3P, MIR1307, NFKB2, PSD, FBXL15, CUEDC2, C10orf95, TMEM180, ACTR1A, SUFU, TRIM8, ARL3, SFXN2, WBP1L, CYP17A1, C10orf32, C10orf32-ASMT, AS3MT, CNNM2, NT5C2, RPEL1, INA, PCGF6, TAF5, USMG5, PDCD11, CALHM2, CALHM1, CALHM3, NEURL1
chr10	105344982	105349270	M14	NEURL1, SH3PXD2A
chr10	105349270	120355160	M11,M14	EMX2OS, CASC2, DCLRE1A, NHLRC2, LINC00867, SLC25A18P1, ADRB1, C10orf118, TDRD1, VWA2, AFAP1L2, ABLIM1, FAM160B1, TRUB1, ATRNL1, GFRA1, CCDC172, PNLIPRP3, PNLIP, PNLIPRP1, PNLIPRP2, C10orf82, HSPA12A, ENO4, KIAA1598, RN7SL524P, MIR936, VAX1, KCNK18, SLC18A2, MIR609, MIR4482-1, CCDC147-AS1, SORCS3-AS1, RNU6-463P, YWHAZP5, RNA5SP325, RNA5SP326, PTGES3P5, MAPKAPK5P1, RN7SKP278, RNU5B-6P, RNU6-839P, PHB2P1, BTF3P15, RN7SL450P, SNRPGP12, PDZD8, HMGB3P5, RPL7P35, RN7SL686P, RN7SKP288, RNA5SP327, PDCD4-AS1, MIR4680, EMX2, RPL13AP6, MIR548E, BTBD7P2, RAB11FIP2, GUCY2GP, FAM204A, PRLHR, MIR4295, RNU7-165P, RNU6-709P, UBE2V1P5, MIR2110, RN7SL384P, PPIAP19, TAF9BP2, RNU6-1121P, SNRPGP6, HMGB3P8, RNU6-1090P, PNLIPP1, RPL5P27, MIR3663, NEURL1, SH3PXD2A, OBFC1, SLK, COL17A1, SFR1, WDR96, GSTO1, GSTO2, ITPRIP, CCDC147, SORCS3, SORCS1, XPNPEP1, ADD3, MXI1, SMNDC1, DUSP5, SMC3, RBM20, PDCD4, BBIP1, SHOC2, ADRA2A, GPAM, TECTB, ACSL5, ZDHHC6, VTI1A, TCF7L2, HABP2, NRAP, CASP7, PLEKHS1
chr10	120355160	120440493	M14	TOMM22P5, PRLHR, CACUL1
chr10	120440493	120809312	M11,M14	LDHAP5, CACUL1, NANOS1, EIF3A
chr10	120809312	121184597	M11,M14,M16	SNORA19, GRK5-IT1, RN7SL749P, MIR4681, EIF3A, FAM45A, SFXN4, PRDX3, GRK5
chr10	121184597	135440183	M11,M14	FAM24B, FAM24A, TIAL1, NPS, FOXI2, CLRN3, C10orf88, PTPRE, PSTK, IKZF5, BAG3, INPP5F, ACADSB, HMX3, HMX2, BUB3, RAD1P1, TXNP1, RN7SL846P, PHACTR2P1, MIR4682, NACAP2, WDR11-AS1, LINC01153, RN7SKP167, RPS15AP5, ATE1-AS1, RNU6-728P, MIR3941, DMBT1P1, MKI67, MCMBP, YBX2P1, RPS10P18, FAM53B-AS1, NPM1P31, MIR4296, MRPS21P6, MGMT, RPS27P18, TEX36-AS1, EBF3, SEC23IP, GLRX3, GPR26, CPXM2, ALDOAP2, EDRF1-AS1, MIR4484, RNU2-42P, GNG10P1, FANK1-AS1, RNA5SP328, LINC00601, PPAPDC1A, C10orf85, WDR11, BUB1P1, TCERG1L, PPP2R2D, BNIP3, JAKMIP3, CHST15, OAT, MIR4297, LINC00959, CTAGE7P, PPIAP32, MIR378C, TCERG1L-AS1, DPYSL4, LINC01165, LINC01166, LINC01167, LINC01168, GPR123-AS1, RPL5P28, MIR202HG, BANF1P2, MIR3944, OR6L2P, STK32C, NKX1-2, LHPP, OR7M1P, OR6L1P, FAM53B, LRRC27, FGFR2, METTL10, PWWP2B, FAM175B, ZRANB1, C10orf91, INPP5A, CTBP2, NKX6-2, TTC40, TEX36, EDRF1, GPR123, KNDC1, ATE1, MMP21, UROS, UTF1, VENTX, ADAM8, NSMCE4A, BCCIP, TUBGCP2, DHX32, TACC2, ZNF511, FANK1, CALY, PRAP1, FUOM, ECHS1, PAOX, ADAM12, C10orf90, MTG1, DOCK1, SPRN, CYP2E1, FAM196A, SYCE1, BTBD16, FRG2B, PLEKHA1, ARMS2, HTRA1, DMBT1, GRK5, RGS10, C10orf120, CUZD1
chr10	135440183	135440299	M11	FRG2B
chr11	126987	22707335	M9	CD81-AS1, RPL26P30, TMEM9B, PHLDA2, MUC5AC, NRIP3, HRAS, NAP1L4, SCUBE2, KCNQ1OT1, COX6CP18, KCNQ1-AS1, KCNQ1DN, LRRC56, SNORA54, CARS-AS1, RNU1-91P, MRGPRG-AS1, NDUFA5P8, NDUFA5P1, TSSC2, OR7E12P, FAM86GP, RPS3AP39, RPS24P14, OR7E117P, TRPC2, MUC5B, RNU6-1143P, RNU7-50P, C11orf35, MIR4687, HNRNPA1P76, RPS29P20, RRM1-AS1, OR55B1P, SSU72P5, SSU72P2, SSU72P6, SSU72P4, SSU72P3, SSU72P7, OR52B3P, OR51R1P, OR52P2P, OR52K3P, OR52M2P, OR51A9P, OR51C1P, OR51F5P, OR51C4P, KRT8P49, OR51F3P, OR51F4P, OR51N1P, RASSF7, OR52Y1P, OR51A8P, OR51H2P, DENND5A, CARS, OR51A6P, OR51A3P, OR51A5P, OR51P1P, OR52J2P, OR52E1, OR52S1P, OR52E3P, PHRF1, OR52J1P, TOLLIP, IRF7, TMEM41B, OR52A4, OR51A1P, OR52Z1, HBBP1, OR51AB1P, IPO7, BRSK2, OR51B3P, OR51B8P, OR51K1P, OR51A10P, OR52V1P, OR52H2P, OR52B5P, OR52T1P, HNRNPA1P53, OR52U1P, OR52P1P, OR56B2P, OR52N3P, OR52E7P, KRT18P58, RNA5SP329, OR56A7P, OR52L2P, OR52X1P, ZNF143, CDHR5, GVINP1, GVINP2, MIR302E, OR10AB1P, OR5P4P, OR5P1P, OR5E1P, RNU6-943P, COX6CP5, SNORA3, SNORA45, RNA5SP330, TMEM9B-AS1, MIR5691, KRT8P41, SNORA23, OSBPL5, RN7SL56P, RN7SKP50, SBF2-AS1, RNU7-28P, RNU6ATAC33P, MRVI1-AS1, SNORD97, MOB2, WEE1, SWAP70, DUSP8, KRTAP5-1, KRTAP5-2, SBF2, SCT, KRTAP5-3, DRD4, KRTAP5-4, KRTAP5-5, ZBED5-AS1, KRTAP5-6, IFITM10, MTND5P21, MIR4299, DEAF1, LINC00958, RASSF10, RN7SKP151, HMGN2P36, FAR1-IT1, RPL39P26, RNA5SP331, CTSD, SPON1, RNA5SP332, RNU7-49P, CALCP, RN7SL188P, RN7SKP90, RNU6-585P, OR7E14P, SNORD14A, SNORD14B, RNU6-593P, SDHCP4, EPS8L2, MRGPRG, MRGPRE, HIGD1AP5, SAA3P, GLTPP1, SAA2-SAA4, RNA5SP333, ST13P5, ZNF195, RNA5SP334, MIR3159, ADM, SPTY2D1-AS1, SYT8, AMPD3, PCNAP4, NAV2-IT1, RNA5SP335, NAV2-AS5, NAV2-AS4, MIR4486, MIR4694, NAV2-AS3, NAV2-AS2, NAV2-AS1, RNA5SP336, TNNI2, RNA5SP337, ART5, ART1, MTRNR2L8, RNF141, CHRNA10, LSP1, TMEM80, LYVE1, TALDO1, MRVI1, NUP98, PDDC1, C11orf89, TNNT3, CTR9, EIF4G2, CEND1, SLC25A22, PGAP2, ZBED5, MRPL23, GALNT18, CSNK2A3, USP47, PIDD, IGF2, INS-IGF2, INS, RPLP2, DKK3, RHOG, STIM1, TH, MICAL2, RRM1, PNPLA2, EFCAB4A, MICALCL, OR52B4, PARVA, ASCL2, TRIM21, C11orf21, OR52K2, OR52K1, OR52M1, C11orf40, OR52I2, OR52I1, TRIM68, CD151, TSPAN32, OR51D1, OR51E1, OR51E2, MMP26, OR51F1, OR52R1, POLR2L, OR51F2, OR51S1, OR51H1P, OR51T1, OR51A7, OR51G2, OR51G1, TSPAN4, OR51A4, OR51A2, OR51L1, OR52J3, OR52E2, OR52A5, OR52A1, OR51V1, HBB, CD81, TEAD1, HBD, HBG1, ARNTL, HBG2, CHID1, HBE1, OR51B4, OR51B2, OR51B5, OR51B6, OR51M1, OR51J1, OR51Q1, OR51I1, OR51I2, OR52D1, UBQLN3, TSSC4, UBQLNL, OR52H1, OR52B6, BTBD10, TRIM6, TRPM5, PTH, FAR1, KCNQ1, TRIM6-TRIM34, TRIM34, RRAS2, AP2A2, TRIM5, CDKN1C, SLC22A18AS, SLC22A18, TRIM22, COPB1, MUC6, PSMA1, OR56B1, OR52N4, OR52N5, OR52N1, OR52N2, OR52E6, OR52E8, OR52E4, OR56A3, MUC2, OR52L1, OR56A4, OR56A1, OR56B4, OR56B3P, OR52B1P, OR52B2, OR52W1, C11orf42, FAM160A2, CNGA4, CCKBR, PRKCDBP, PDE3B, CYP2R1, CALCB, SMPD1, CALCA, INSC, APBB1, SOX6, C11orf58, HPX, PLEKHA7, TRIM3, RPS13, PIK3C2A, ARFIP2, NUCB2, TIMM10B, NCR3LG1, DNHD1, KCNJ11, ABCC8, RRP8, ILK, USH1C, TAF10, TPP1, OTOG, DCHS1, MYOD1, MRPL17, KCNC1, OR2AG2, OR2AG1, OR6A2, OR10A5, OR10A2, SERGEF, OR10A4, OR2D2, OR2D3, ZNF215, ZNF214, NLRP14, RBMXL2, SYT9, TPH1, OLFML1, SAAL1, PPFIBP2, MRGPRX3, MRGPRX4, SAA4, SAA2, SAA1, CYB5R2, HPS5, OVCH2, OR5P2, OR5P3, OR10A6, OR10A3, NLRP10, EIF3F, TUB, GTF2H1, RIC3, LDHA, LMO1, STK33, TRIM66, RPL27A, LDHC, LDHAL6A, TSG101, ST5, UEVLD, SPTY2D1, TMEM86A, IGSF22, AKIP1, PTPN5, C11orf16, ASCL3, MRGPRX1, MRGPRX2, ZDHHC13, CSRP3, E2F8, NAV2, DBX1, HTATIP2, PRMT3, SLC6A5, NELL1, ANO5, SLC17A6, FANCF, GAS2, BET1L, SCGB1C1, ODF3, RIC8A, SIRT3, PSMD13, NLRP6, ATHL1, IFITM5, IFITM2, IFITM1, IFITM3, B4GALNT4, PKP3, SIGIRR, ANO9, PTDSS2, RNH1, LINC01001, RNU6-447P, RN7SL838P, MIR210HG, MIR210, SNORA52, RNU6-1025P, TOLLIP-AS1, KRTAP5-AS1, FAM99A, FAM99B, RPL36AP39, MIR4298, LINC01150, MRPL23-AS1, H19, MIR483, IGF2-AS, MIR4686, RNU6-878P
chr11	47689122	47859175	M17	RNA5SP340, AGBL2, FNBP4, NUP160

chr11	59633864	63414006	M2	SLC22A25, SLC22A9, HRASLS5, LGALS12, RARRES3, HRASLS2, PLA2G16, ATL3, SRD5A3P1, LINC00301, RNU6-933P, RN7SL23P, MIR4488, RPLP0P2, MIR611, MIR1908, RNU6-1243P, NPM1P35, RCC2P6, SNORA57, RNU6-118P, RN7SL119P, RNU2-2P, SNHG1, RN7SL259P, RPL29P22, CCND2P1, TCN1, OOSP1, OOSP2, MS4A3, MS4A2, MS4A6A, MS4A4E, MS4A4A, MS4A6E, MS4A7, MS4A14, MS4A5, MS4A1, MS4A12, MS4A13, MS4A8, MS4A18, MS4A15, MS4A10, CCDC86, PTGDR2, ZP1, PRPF19, TMEM109, TMEM132A, SLC15A3, CD6, CD5, VPS37C, PGA3, PGA4, PGA5, VWCE, DDB1, DAK, CYB561A3, TMEM138, TMEM216, CPSF7, SDHAF2, PPP1R32, LRRC10B, SYT7, DAGLA, MYRF, TMEM258, FEN1, FADS2, FADS1, FADS3, RAB3IL1, BEST1, FTH1, INCENP, SCGB1D1, SCGB2A1, SCGB1D2, SCGB2A2, SCGB1D4, ASRGL1, SCGB1A1, AHNAK, EEF1G, MIR3654, TUT1, MTA2, EML3, ROM1, B3GAT3, GANAB, INTS5, C11orf48, METTL12, C11orf83, UBXN1, LRRN4CL, HNRNPUL2-BSCL2, BSCL2, GNG3, HNRNPUL2, TTC9C, ZBTB3, POLR2G, TAF6L, TMEM223, TMEM179B, NXF1, STX5, WDR74, SLC3A2, CHRM1, SLC22A6, SLC22A8, SLC22A24, SLC22A10
chr11	63414006	65838313	M14,M2	KCNK7, MAP3K11, PCNXL3, RASGRP2, SIPA1, RELA, PYGM, ATL3, KAT5, RTN3, RNASEH2C, AP5B1, OVOL1, CFL1, SF1, SNX32, MUS81, C11orf84, EFEMP2, MARK2, CTSW, FIBP, MAP4K2, CCDC85B, FOSL1, C11orf68, DRAP1, RCOR2, TSGA10IP, SART1, NAA40, EIF1AD, COX8A, OTUB1, BANF1, MEN1, CST6, CATSPER1, GAL3ST3, SF3B2, MACROD1, FLRT1, C11orf95, PACS1, RN7SL596P, RNU6-1306P, RNU6-45P, STIP1, CDC42BPG, EHD1, FERMT3, ATG2A, TRPT1, MIR1237, MIR192, MIR194-2, RN7SL114P, HIGD1AP10, RNU2-23P, PGAM1P8, SLC22A20, NEAT1, MALAT1, SNRPGP19, SSSCA1-AS1, MIR4690, MIR4489, RN7SL309P, KRT8P26, OVOL1-AS1, NUDT22, PPP2R5B, DNAJC4, GPHA2, C11orf85, VEGFB, BATF2, FKBP2, ARL2, PPP1R14B, SNX15, PLCB3, SAC3D1, NAALADL1, BAD, GPR137, KCNK4, CDCA5, TEX40, ZFPL1, ESRRA, TMEM262, TRMT112, VPS51, PRDX5, CCDC88B, TM7SF2, ZNHIT2, FAU, RPS6KA4, SYVN1, SLC22A11, MRPL49, SPDYC, SLC22A12, CAPN1, NRXN2, POLA2, CDC42EP2, DPF2, TIGD3, SLC25A45, FRMD8, SCYL1, LTBP3, SSSCA1, FAM89B, EHBP1L1
chr11	65838313	66639157	M14	PACS1, KLC2, RAB1B, CNIH2, YIF1A, TMEM151A, CD248, RIN1, RNU1-84P, BRMS1, RNU4-39P, RN7SL12P, FTLP6, B3GNT1, SLC29A2, NPAS4, MRPL11, PELI3, DPP3, BBS1, ZDHHC24, ACTN3, CTSF, CCDC87, CCS, RBM14, RBM4, RBM14-RBM4, RBM4B, SPTBN2, C11orf80, RCE1, PC, LRFN4
chr11	66639157	67088804	M14,M2	RNU7-23P, MIR3163, PC, C11orf86, SYT12, RHOD, KDM2A, ADRBK1, ANKRD13D, SSH3
chr11	67088804	70170558	M14	RN7SKP239, RNU6-1238P, RN7SL59P, DOC2GP, RPL37P2, UNC93B5, OR7E11P, ALG1L8P, FAM86C2P, RNU6-46P, RPS3AP40, OR7E1P, ALDH3B1, MIR4691, NDUFA3P2, MIR3164, IFITM9P, ANO1-AS2, RNU6-1175P, ANO1-AS1, H2AFZP4, MIR548K, POLD4, CLCF1, RAD9A, PPP1CA, TBC1D10C, CARNS1, RPS6KB2, PTPRCAP, CORO1B, GPR152, CABP4, TMEM134, AIP, PITPNM1, CDK2AP2, CABP2, GSTP1, C11orf72, NDUFV1, NUDT8, TBX10, ACY3, ALDH3B2, UNC93B1, NDUFS8, TCIRG1, CHKA, SUV420H1, C11orf24, LRP5, PPP6R3, GAL, MTL5, CPT1A, MRPL21, IGHMBP2, MRGPRD, MRGPRF, TPCN2, MYEOV, CCND1, ORAOV1, FGF19, FGF4, FGF3, ANO1, FADD, PPFIA1
chr11	73366847	73753263	M16	RN7SKP243, CCDC58P5, PLEKHB1, RAB6A, MRPL48, COA4, PAAF1, DNAJB13, UCP2, UCP3, C2CD3
chr11	105623706	107219662	M11	GRIA4, MSANTD4, KBTBD3, AASDHPPT, GUCY1A2, CWF19L2, RNU4-55P, RNU6-277P
chr11	107219662	134257834	M11,M2	RNU6-321P, STT3A-AS1, RNU6-1156P, RNU2-35P, NAP1L1P1, RNU4-86P, RN7SL351P, RPL35AP26, KIRREL3-AS1, KIRREL3-AS2, MIR3167, KIRREL3-AS3, RN7SKP121, RN7SKP279, SENCR, RNU6-876P, RNU6-874P, ZNF123P, RPS27P20, LINC00167, TCEB2P2, RPL34P21, DDX18P5, BAK1P2, PPP1R10P1, RN7SL167P, RNU6ATAC12P, NTM-IT, RNU6-1182P, OPCML-IT2, OPCML-IT1, MIR4697, PTP4A2P2, CWF19L2, ALKBH8, ELMOD1, SLN, SLC35F2, RAB39A, CUL5, ACAT1, NPAT, SMARCE1P1, ATM, RPS2P39, CYCSP29, RNU6-654P, RNA5SP349, TFAMP2, HNRNPA1P60, RNA5SP350, RPS17P15, MIR4491, RNU2-60P, MIR34B, MIR34C, RN7SKP273, ALG9-IT1, GNG5P3, RPL37AP8, RNA5SP351, PPIHP1, RNU6-893P, MRPS36P4, RPS12P21, RPS6P16, ST13P10, RNU6-44P, RPL23AP62, RNU7-187P, NCAM1-AS1, MIR4301, ATF4P4, LRRC37A13P, RNU6-1107P, NXPE2P1, LINC00900, C11orf65, APOA1-AS, SIK3-IT1, RNY4P6, TMPRSS4-AS1, HSPE1P18, RN7SL86P, RPL5P30, RNU6-1157P, RNU6-376P, SETP16, MIR4492, RN7SL688P, RN7SL529P, RPL23AP64, MIR3656, KDELC2, SLC37A4, EXPH5, DDX10, C11orf87, ZC3H12C, RDX, FDX1, ARHGAP20, VPS11, RNU6-262P, USP2-AS1, DUXAP5, KRT8P7, RNU6-1123P, TCEB1P22, HMGB1P42, RPS4XP12, BMPR1APS2, RNU6-256P, MIR125B1, MIRLET7A2, MIR100, RNU4ATAC10P, RNU4ATAC5P, RNU6-592P, GLULP3, RNU4-23P, ATP5F1P5, RPL34P23, RPS26P43, RPL31P47, SNORD14E, SNORD14D, SNORD14C, MIR4493, LINC01059, SF3A3P2, RNU1-21P, OR6M2P, OR6M3P, OR10G5P, C11orf53, COLCA1, COLCA2, POU2AF1, OR10D5P, OR10D4P, OR10N1P, OR10D1P, OR8F1P, OR8G3P, OR8G7P, SLC5A4P1, OR8B7P, OR8B6P, OR8B5P, OR8B1P, OR8C1P, BTG4, OR8X1P, OR8A2P, OR8B9P, OR8B10P, OR8A3P, OR8Q1P, RNA5SP352, KRT18P59, C11orf88, LAYN, SIK2, PPP2R1B, ALG9, FDXACB1, C11orf1, CRYAB, HSPB2-C11orf52, C11orf52, DIXDC1, DLAT, PIH1D2, C11orf57, TIMM8B, SDHD, IL18, TEX12, BCO2, PTS, PLET1, NCAM1, TTC12, ANKK1, DRD2, TMPRSS5, ZW10, CLDN25, USP28, HTR3B, HTR3A, ZBTB16, NNMT, C11orf71, RBM7, REXO2, NXPE1, NXPE4, NXPE2, CADM1, BUD13, ZNF259, APOA5, APOA4, APOC3, APOA1, SIK3, PAFAH1B2, SIDT2, TAGLN, PCSK7, RNF214, BACE1, CEP164, DSCAML1, FXYD2, FXYD6-FXYD2, FXYD6, TMPRSS13, IL10RA, TMPRSS4, SCN4B, SCN2B, AMICA1, MPZL3, MPZL2, CD3E, CD3D, CD3G, UBE4A, ATP5L, KMT2A, TTC36, TMEM25, IFT46, ARCN1, PHLDB1, TREH, DDX6, CXCR5, BCL9L, UPK2, FOXR1, CCDC84, RPS25, TRAPPC4, HYOU1, HMBS, H2AFX, DPAGT1, C2CD2L, HINFP, ABCG4, NLRX1, PDZD3, CCDC153, CBL, MCAM, RNF26, C1QTNF5, MFRP, USP2, THY1, PVRL1, TRIM29, OAF, POU2F3, TMEM136, ARHGEF12, GRIK4, TBCEL, TECTA, SC5D, SORL1, BLID, UBASH3B, CRTAM, C11orf63, BSX, HSPA8, CLMP, GRAMD1B, SCN3B, ZNF202, OR6X1, OR6M1, TMEM225, OR8D4, OR4D5, OR6T1, OR10S1, OR10G6, OR10G4, OR10G9, OR10G8, OR10G7, VWA5A, OR10D3, OR8G1, OR8G5, OR8D1, OR8D2, OR8B2, OR8B3, OR8B4, OR8B8, OR8B12, OR8A1, PANX3, TBRG1, SIAE, SPA17, NRGN, VSIG2, ESAM, MSANTD2, ROBO3, ROBO4, HEPACAM, HEPN1, CCDC15, SLC37A2, TMEM218, PKNOX2, FEZ1, EI24, STT3A, CHEK1, ACRV1, PATE1, PATE2, PATE3, PATE4, HYLS1, PUS3, DDX25, CDON, RPUSD4, FAM118B, SRPR, FOXRED1, TIRAP, DCPS, ST3GAL4, KIRREL3, ETS1, FLI1, KCNJ1, KCNJ5, C11orf45, TP53AIP1, ARHGAP32, BARX2, TMEM45B, NFRKB, PRDM10, APLP2, ST14, ZBTB44, ADAMTS8, ADAMTS15, C11orf44, SNX19, NTM, OPCML, SPATA19, IGSF9B, JAM3, NCAPD3, VPS26B, THYN1, ACAD8, GLB1L3, GLB1L2, B3GAT1
chr12	7656240	8281958	M2	CD163, APOBEC1, GDF3, DPPA3, CLEC4C, NANOGNB, NANOG, SLC2A14, SLC2A3, FOXJ2, C3AR1, NECAP1, CLEC4A, GAPDHP31, NIFKP3, NANOGP1
chr12	19282984	19675173	M17	RN7SL459P, RN7SL67P, RNU6-254P, EEF1A1P4, PLEKHA5, AEBP2
chr12	110230456	112308164	M2	BRAP, ACAD10, ANKRD13A, C12orf76, IFT81, ALDH2, ATP2A2, MAPKAPK5, ANAPC7, ARPC3, GPN3, FAM216A, VPS29, MIR4497, RN7SL441P, RN7SL769P, HMGA1P3, RPL31P49, RN7SL387P, RPL29P25, RAD9B, RNA5SP373, HSPA8P14, PCNPP1, MAPKAPK5-AS1, PPTC7, TCTN1, HVCN1, PPP1CC, CCDC63, MYL2, CUX2, FAM109A, SH2B3, ATXN2, TRPV4, GLTP, TCHP, GIT2
chr12	120597945	120615303	M16	GCN1L1
chr12	120615303	124267811	M14,M16	PXN-AS1, HNF1A, RPS20P31, NME2P1, RNU4-2, RNU4-1, RNU6-1088P, RPS27P25, RPL31P52, DYNLL1-AS1, RPL29P24, RPL11P5, MIR4700, ARF1P2, CLIC1P1, RPL12P33, HNF1A-AS1, C12orf43, OASL, RNU6-1004P, RNU7-170P, P2RX7, RPL21P1, MIR4304, RN7SL133P, RNA5SP375, MIR3908, RPL27P12, P2RX4, CAMKK2, ANAPC5, RNF34, KDM2B, GCN1L1, RPLP0, ORAI1, MORN3, TMEM120B, RHOF, SETD1B, HPD, PSMD9, WDR66, BCL7A, MLXIP, LRRC43, IL31, B3GNT4, DIABLO, VPS33A, CLIP1, PXN, ZCCHC8, SIRT4, PLA2G1B, RSRC2, MSI1, COX6A1, TRIAP1, GATC, SRSF9, DYNLL1, COQ5, RNF10, KNTC1, HCAR1, HCAR2, HCAR3, DENR, POP5, CCDC62, CABP1, HIP1R, MLEC, UNC119B, ACADS, SPPL3, VPS37B, ABCB9, OGFOD2, ARL6IP4, PITPNM2, MPHOSPH9, C12orf65, CDK2AP1, SBNO1, SETD8, RILPL2, SNRNP35, RILPL1, TMED2, DDX55, EIF2B1, GTF2H3, TCTN2, ATP6V0A2, DNAH10
chr12	124267811	126004077	M14	BRI3BP, AACS, TMEM132B, RNU6-927P, MIR5188, RPL22P19, DNAH10, CCDC92, DNAH10OS, ZNF664, FAM101A, NCOR2, SCARB1, UBC, DHX37
chr12	133219405	133252679	M2	POLE
chr12	133252679	133783695	M16,M2	RNA5SP379, RPS11P5, RNU6-327P, NANOGNBP2, RNU4ATAC12P, PTP4A1P2, RPL23AP67, POLE, PXMP2, PGAM5, ANKLE2, GOLGA3, CHFR, ZNF605, ZNF26, ZNF84, ZNF140, ZNF891, ZNF10, ZNF268
chr14	50044052	50808940	M2	RNU6ATAC30P, RNU6-539P, RN7SL3, RN7SL2, RNU6-189P, PDLIM1P1, RN7SKP193, MIR4504, RPL32P29, RN7SL1, RHOQP1, RPS29, LRR1, RPL36AL, MGAT2, DNAAF2, POLE2, KLHDC1, KLHDC2, NEMF, ARF6, C14orf182, C14orf183, VCPKMT, SOS2, L2HGDH, ATP5S, CDKL1
chr14	54996855	59113505	M14	RNU6ATAC9P, MIR4308, FDPSP3, CHMP4BP1, RPL21P6, KTN1-AS1, RPL7AP4, RPL13AP3, LINC00520, OTX2-AS1, RN7SL461P, RNU6-1204P, RPL3P3, RN7SKP99, UBA52P3, RN7SL598P, HMGB1P14, RNU6-341P, HSBP1P1, HNRNPCP1, RPL9P5, CGRRF1, SAMD4A, GCH1, WDHD1, SOCS4, MAPK1IP1L, LGALS3, DLGAP5, FBXO34, ATG14, TBPL2, KTN1, PELI2, TMEM260, OTX2, EXOC5, AP5M1, NAA30, C14orf105, SLC35F4, C14orf37, ACTR10, PSMA3, ARID4A, TOMM20L, TIMM9, KIAA0586, DACT1

chr14	60558882	101393713	M14	WDR89, SGPP1, FAM35CP, RNU6-835P, LINC01146, ESRRB, CLMN, RNU4-22P, RNU4-92P, MPPE1P1, CAP2P1, FOXN3-AS1, SYNE2, VASH1, FOXN3-AS2, RN7SKP107, RN7SKP255, CHORDC2P, RAB42P1, RPL21P11, LINC00642, RNU7-30P, SNORA11B, RN7SL506P, NANOGP7, RNU6-366P, SYNE3, ITPK1-AS1, ANGEL1, RNU6-1258P, FAM181A-AS1, MIR4506, LINC00521, SERPINA2P, ADIPOR1P2, GLRX5, SERPINA13P, RPSAP4, RPL15P2, TCL1B, MIR3173, DICER1-AS1, C14orf166B, TCL1A, LINC00341, IRF2BPL, CIPC, SNHG10, SCARNA13, C14orf132, TMEM63C, TCL6, BDKRB2, BDKRB1, LINC00617, ATG2B, ZDHHC22, NGB, POMT2, RNU2-33P, PEBP1P1, RPL23AP10, GSKIP, SCOCP1, AK7, PSMA3P, RPL17P2, RBM8B, GNRHR2P1, SALL4P7, RN7SKP108, LINC00618, RN7SL710P, RN7SL714P, RPL3P4, SRMP2, GSTZ1, RNU1-47P, VDAC3P1, RNU6-398P, MIR151B, MIR342, HIF1A-AS1, HIF1A-AS2, COX4I1P1, MIR345, PAPOLA, LINC00643, RN7SL523P, LINC00644, RN7SKP92, LINC00523, MEG3, GPHB5, RN7SL540P, RNU6-852P, HSPE1P2, RNU6-597P, RNU6-1162P, RNU7-116P, RPS28P1, MIR548H1, TEX21P, RPPH1-2P, MIR4706, TMED8, SAMD15, ESR2, NOXRED1, MIR2392, RNU2-14P, MIR770, MIR493, MIR337, MIR665, MIR431, MIR433, MIR127, MIR432, MIR136, MEG8, RPL21P7, PTBP1P, MIR4708, RPL21P8, MIR625, EIF1AXP2, YBX1P1, MIR370, SNORD113-1, VIPAS39, SNORD113-2, LINC00238, VRK1, MTHFD1, MIR5694, COX7A2P1, C14orf64, C14orf177, BCL11B, ZBTB25, RN7SL369P, RPL21P9, RN7SL213P, AKAP5, RN7SL706P, ZBTB1, RN7SL108P, PPIAP6, RNU6-921P, RN7SL224P, BLZF2P, HMGN1P3, BANF1P1, ACTN1-AS1, HSPA2, PPP1R36, DDX18P1, SETD3, RPL7AP6, ADAM21P1, SYNJ2BP-COX16, RNU2-51P, ADAM20P1, RNU6-659P, KRT18P7, RN7SL77P, PLEKHG3, PTTG4P, AHSA1, SNORD56B, RN7SL683P, SPTB, CCNK, CCDC85C, ISM2, RN7SL586P, RNU6-419P, C14orf169, NT5CP2, NT5CP1, HHIPL1, NDUFB8P1, RNU6-240P, MIR4505, SPTLC2, RN7SL530P, SUB1P2, RPS2P2, MIR4709, RAP1AP, ALKBH1, CYP46A1, SLIRP, RNU6-689P, HIF1AP1, RNU4ATAC14P, RNA5SP387, EML1, RN7SL747P, CYCSP1, RN7SKP17, CHURC1, FNTB, RN7SL356P, SNW1, CHURC1-FNTB, MIR1260A, RN7SL137P, RN7SL587P, COX6CP11, RPL21P10, ZMYND19P1, FRDAP, RNA5SP388, C14orf178, GPX2, RAB15, DIO2-AS1, RPL17P3, NMNAT1P1, SNORA79, DYNLL1P1, UNGP3, EIF3LP1, ENSAP2, RNU7-51P, RNU6ATAC28P, ADCK1, MAX, EVL, NRXN3, FUT8, RNU6-976P, RNU3P3, LINC00911, DIO2, CEP128, DEGS2, GPHN, YY1, SLC25A29, SLC25A47, TSHR, WARS, FAM71D, MPP5, GTF2A1, STON2, ATP6V1D, EIF2S1, SEL1L, PLEK2, TMEM229B, FLRT2, WDR25, PLEKHH1, GALC, BEGAIN, PIGH, DLK1, ARG2, VTI1B, GPR65, KCNK10, RDH11, SPATA7, RTL1, RDH12, ZFYVE26, PTPN21, ZC3H14, RAD51B, ZFP36L1, ACTN1, EML5, DCAF5, EXD2, GALNT16, ERH, SLC39A9, TTC8, PLEKHD1, CCDC177, KIAA0247, SRSF5, FOXN3, SLC10A1, SMOC1, EFCAB11, TDP1, SLC8A3, COX16, KCNK13, PSMC1, SYNJ2BP, ADAM21, ADAM20, MED6, TTC9, MAP3K9, NRDE2, PCNX, CALM1, SIPA1L1, TTC7B, RGS6, RPS6KA5, C14orf159, DPF3, DCAF4, ZFYVE1, GPR68, CCDC88C, RBM25, SMEK1, PSEN1, CATSPERB, TC2N, FBLN5, TRIP11, PAPLN, NUMB, HEATR4, ACOT1, ACOT2, ACOT4, ACOT6, DNAL1, PNMA1, ELMSAN1, ATXN3, PTGR2, ZNF410, FAM161B, COQ6, ENTPD5, CCDC176, ALDH6A1, LIN52, VSX2, ABCD4, VRTN, SYNDIG1L, NPC2, ISCA2, NDUFB1, LTBP2, CPSF2, SLC24A4, AREL1, RIN3, FCF1, YLPM1, LGMN, PROX2, GOLGA5, CHGA, DLST, RPS6KL1, ITPK1, MOAP1, TMEM251, PGF, C14orf142, UBR7, EIF2B2, BTBD7, MLH3, UNC79, ACYP1, ZC2HC1C, NEK9, COX8C, PRIMA1, FAM181A, ASB2, TMED10, FOS, OTUB2, DDX24, JDP2, BATF, FLVCR2, IFI27L1, IFI27, TTLL5, IFI27L2, PPP4R4, SERPINA10, SERPINA6, SERPINA1, C14orf1, IFT43, TGFB3, GPATCH2L, SERPINA11, SERPINA9, SERPINA12, SERPINA4, SERPINA5, PCNXL4, SERPINA3, GSC, DHRS7, DICER1, PPM1A, C14orf39, SIX6, SIX1, SIX4, MNAT1, TRMT5, SLC38A6, PRKCH, TMEM30B, HIF1A, SNAPC1, SYT16, KCNH5, RHOJ, PPP2R5E
chr14	101488435	106950849	M14	IGHD2-2, KIAA0125, IGHD1-1, IGHV6-1, IGHV1-2, IGHV1-3, IGHV4-4, IGHV2-5, IGHV3-7, IGHV1-8, IGHV3-9, IGHV3-11, IGHV3-13, IGHV3-15, IGHV3-16, IGHV1-18, IGHV3-20, IGHV3-21, IGHV3-23, IGHV1-24, IGHV2-26, IGHV4-28, IGHV3-30, IGHV4-31, IGHV3-33, IGHV4-34, IGHV3-35, IGHV3-38, IGHV4-39, IGHV3-43, MIR379, MIR411, MIR299, MIR380, MIR1197, MIR323A, MIR758, MIR329-1, MIR329-2, MIR494, MIR1193, MIR543, MIR495, MIR376C, MIR654, MIR376A1, MIR300, MIR1185-1, MIR1185-2, MIR381HG, MIR381, MIR487B, MIR539, MIR889, MIR544A, MIR655, MIR487A, MIR382, MIR134, MIR485, MIR323B, MIR154, MIR496, MIR377, MIR541, MIR409, MIR412, MIR369, MIR410, MIR656, MEG9, LINC00524, DIO3OS, RNU6-790P, RN7SL472P, RNU6-244P, MIR4309, RN7SL546P, RNU6-1316P, RPL13P6, LINC00677, RPL21P12, GCSHP2, LINC00605, RPL21P13, RAP2CP1, SNORA28, HMGB3P26, RNU7-160P, RNU4-68P, LINC00637, RN7SL634P, MIR203, RNU6-684P, CEND1P1, MIR4710, LINC00638, RPS26P49, RPS2P4, RPS20P33, ATP5G1P1, ELK2BP, IGHGP, ELK2AP, IGHEP1, IGHJ3P, IGHJ2P, IGHJ1P, IGHVII-1-1, ADAM6, IGHVIII-2-1, IGHVIII-5-1, IGHVIII-5-2, IGHV3-6, IGHV2-10, IGHVIII-11-1, IGHV1-12, IGHVIII-13-1, IGHV1-14, IGHVII-15-1, IGHVIII-16-1, IGHV1-17, SLC20A1P2, IGHV3-19, IGHVII-20-1, IGHV3-22, IGHVII-22-1, IGHVIII-22-2, HOMER2P2, LINC00226, IGHV3-25, IGHVIII-25-1, IGHVIII-26-1, IGHVII-26-2, IGHV7-27, IGHVII-28-1, IGHV3-29, IGHVII-30-1, IGHV3-30-2, IGHVII-31-1, IGHV3-32, IGHVII-33-1, IGHV3-33-2, IGHV7-34-1, IGHV3-36, IGHV3-37, IGHVIII-38-1, IGHV7-40, IGHVII-40-1, IGHV3-41, HOMER2P1, IGHV3-42, IGHVII-43-1, IGHVIII-44, LINC00221, IGHVIV-44-1, IGHVII-44-2, DIO3, PPP2R5C, DYNC1H1, HSP90AA1, WDR20, MOK, ZNF839, CINP, TECPR2, ANKRD9, RCOR1, TRAF3, AMN, CDC42BPB, EXOC3L4, TNFAIP2, EIF5, MARK3, CKB, TRMT61A, BAG5, KLC1, APOPT1, XRCC3, ZFYVE21, PPP1R13B, C14orf2, TDRD9, RD3L, ASPG, KIF26A, C14orf144, TMEM179, C14orf180, INF2, ADSSL1, SIVA1, AKT1, ZBTB42, CEP170B, PLD4, AHNAK2, C14orf79, CDCA4, GPR132, JAG2, NUDT14, BRF1, BTBD6, PACS2, TEX22, MTA1, CRIP2, CRIP1, C14orf80, TMEM121, IGHA2, IGHE, IGHG4, IGHG2, IGHA1, IGHG1, IGHG3, IGHD, IGHM, IGHJ6, IGHJ5, IGHJ4, IGHJ3, IGHJ2, IGHJ1, IGHD7-27, IGHD1-26, IGHD6-25, IGHD5-24, IGHD4-23, IGHD3-22, IGHD2-21, IGHD1-20, IGHD6-19, IGHD5-18, IGHD4-17, IGHD3-16, IGHD2-15, IGHD1-14, IGHD6-13, IGHD5-12, IGHD4-11, IGHD3-10, IGHD3-9, IGHD2-8, IGHD1-7, IGHD6-6, IGHD5-5, IGHD4-4, IGHD3-3
chr15	20740232	22833394	M14	GOLGA6L6, POTEB2, IGHD5OR15-5B, IGHD4OR15-4B, IGHD3OR15-3B, IGHD2OR15-2B, IGHD1OR15-1B, POTEB, OR4M2, OR4N4, IGHV1OR15-1, IGHV4OR15-8, GOLGA6L1, GOLGA8CP, RN7SL759P, SPATA31E2P, IGHV1OR15-6, NBEAP1, RNU6-498P, GRAMD4P5, MIR3118-4, MIR3118-6, RNU6-749P, NF1P1, MIR5701-1, MIR5701-2, CT60, OR11J2P, OR11J5P, KIAA0125P2, RN7SL400P, BMS1P16, RNU6-1235P, CXADRP2, GRAMD4P6, RNU6-631P, NF1P2, OR11J1P, OR11H3P, OR11K1P, OR4Q1P, OR4H6P, OR4N3P, IGHV1OR15-3, IGHV1OR15-4, MIR1268A, MIR4509-1, MIR4509-3, MIR4509-2, ABCB10P1, GOLGA8DP, RN7SL545P, ELMO2P1
chr15	22833394	22841038	M11,M14	TUBGCP5
chr15	22841038	22963874	M11,M14,M16,M17	TUBGCP5, CYFIP1
chr15	22963874	22969362	M14,M16,M17	CYFIP1
chr15	22969362	23012439	M14,M17	CYFIP1, NIPA2
chr15	23012439	40682036	M14	NIPA2, NIPA1, GOLGA8I, GOLGA8S, GOLGA6L2, MKRN3, MAGEL2, NDN, NPAP1, SNRPN, SNURF, UBE3A, ATP10A, GABRB3, GABRA5, GABRG3, OCA2, HERC2, GOLGA8F, GOLGA8G, GOLGA8M, APBA2, FAM189A1, NDNL2, TJP1, GOLGA8J, GOLGA8T, CHRFAM7A, GOLGA8R, GOLGA8Q, GOLGA8H, ARHGAP11B, FAN1, MTMR10, TRPM1, KLF13, OTUD7A, CHRNA7, GOLGA8K, GOLGA8O, GOLGA8N, ARHGAP11A, SCG5, GREM1, FMN1, RYR3, AVEN, CHRM5, EMC7, PGBD4, KATNBL1, EMC4, SLC12A6, NOP10, NUTM1, LPCAT4, GOLGA8A, WHAMMP3, RN7SL495P, HERC2P2, HERC2P7, GOLGA8EP, RN7SL106P, SPATA31E3P, RN7SL536P, MIR4508, GOLGA8B, RNU6-741P, PWRN4, PWRN2, PWRN3, PWRN1, GJD2, ACTC1, AQR, RPL5P1, SNHG14, ZNF770, DPH6, SNORD64, SNORD108, PWAR6, SNORD109A, SNORD116-1, SNORD116-2, SNORD116-3, SNORD116-5, SNORD116-6, SNORD116-7, SNORD116-8, SNORD116-9, SNORD116-10, SNORD116-11, SNORD116-12, SNORD116-13, SNORD116-14, SNORD116-15, SNORD116-16, SNORD116-17, SNORD116-18, SNORD116-19, C15orf41, SNORD116-21, SNORD116-20, SNORD116-23, SNORD116-24, SNORD116-25, SNORD116-26, SNORD116-27, SNORD116-29, SNORD116-30, TMEM261P1, SNORD115-1, SNORD115-2, SNORD115-3, SNORD115-4, SNORD115-5, SNORD115-6, SNORD115-8, SNORD115-9, SNORD115-10, SNORD115-11, SNORD115-12, SNORD115-14, SNORD115-15, SNORD115-16, SNORD115-17, SNORD115-18, SNORD115-19, SNORD115-20, SNORD115-21, SNORD115-22, SNORD115-23, SNORD115-24, SNORD115-25, SNORD115-27, SNORD115-28, SNORD115-29, SNORD115-30, SNORD115-31, SNORD115-32, SNORD115-33, SNORD115-34, SNORD115-35, SNORD115-36, SNORD115-37, SNORD115-38, SNORD115-39, SNORD115-40, SNORD115-41, SNORD115-42, SNORD115-43, SNORD115-44, SNORD115-45, SNORD115-47, SNORD115-48, SNORD109B, RNA5SP390, MIR4715, LINC00929, TVP23BP1, MEIS2, RNA5SP391, RPL5P32, RPL41P2, RN7SL238P, ABCB10P3, MIR4509-1, MIR4509-3, MIR4509-2, ABCB10P4, RN7SL829P, HERC2P9, RN7SL719P, WHAMMP2, GOLGA6L7P, TUBBP8, TMCO5A, HMGN2P5, RN7SL673P, DNM1P28, ULK4P3, RN7SL469P, DNM1P30, RNU6-17P, RN7SL196P, RN7SL796P, DNM1P50, ULK4P2, RN7SL628P, GOLGA8UP, RN7SL82P, HERC2P10, RNU6-466P, MIR211, UBE2CP4, RNU6-18P, DNM1P31, RN7SL185P, SPRED1, ULK4P1, FAM98B, DNM1P32, RN7SL539P, RN7SL286P, RASGRP1, HNRNPA1P71, TMCO5B, MIR1233-1, HNRNPLP2, MIR1233-2, NANOGP8, PRELID1P4, ANP32AP1, MIR3942, HNRNPA1P45, DPH6-AS1, MIR4510, COX6CP4, LARP4P, TPST2P1, CSNK1A1P1, C15orf53, C15orf54, THBS1, FSIP1, GPR176, EIF2AK4, H3F3AP1, SRP14-AS1, PLCB2-AS1, LINC00984, RNA5SP392, LINC00594, SRP14, BMF, BUB1B, PAK6, C15orf56, PLCB2, ANKRD63, C15orf52, PHGR1, DISP2, KNSTRN
chr15	40682036	41861220	M14,M2	RMDN3, GCHFR, DNAJC17, SUMO2P15, C15orf62, ISCA1P4, CYCSP2, FAM92A1P1, RN7SL497P, OIP5-AS1, ZFYVE19, RNU6-1169P, TCEB1P2, PPP1R14D, SPINT1, RHOV, VPS18, DLL4, CHAC1, INO80, EXD1, CHP1, OIP5, NUSAP1, NDUFAF1, RTF1, ITPKA, LTK, RPAP1, TYRO3, RNU6-516P, RN7SL376P, RAD51-AS1, KNSTRN, IVD, BAHD1, CHST14, C15orf57, RPUSD2, CASC5, RAD51
chr15	41861220	42446519	M14	MIR626, RNA5SP393, MIR4310, TYRO3, MGA, MAPKBP1, PLA2G4B, JMJD7, JMJD7-PLA2G4B, SPTBN5, EHD4, PLA2G4E, PLA2G4D, PLA2G4F
chr15	42446519	45983324	M14,M2	MIR627, RNU6-188P, EIF4EBP2P2, KRT8P50, FDPSP4, RPS3AP47, SPCS2P1, ATP5HP1, RN7SL487P, RNU6-554P, RNU6-610P, PDIA3P2, STRCP1, RNU6-353P, CATSPER2P1, RNU6-354P, MIR1282, PIN4P1, ACTBP7, GAPDHP55, RN7SL347P, HNRNPMP1, EIF3J-AS1, RNU1-119P, RNU6-1108P, RNU6-1332P, RNU6-966P, RNU1-78P, RNU6-953P, MIR147B, RNU7-5P, HMGN2P46, DPPA5P2, PLA2G4F, VPS39, TMEM87A, GANC, CAPN3, ZNF106, SNAP23, LRRC57, HAUS2, STARD9, CDAN1, TTBK2, UBR1, EPB42, TMEM62, CCNDBP1, TGM5, TGM7, LCMT2, ADAL, ZSCAN29, TUBGCP4, TP53BP1, MAP1A, PPIP5K1, CKMT1B, STRC, CATSPER2, CKMT1A, PDIA3, ELL3, SERF2, SERINC4, HYPK, MFAP1, WDR76, FRMD5, CASC4, CTDSPL2, EIF3J, SPG11, PATL2, B2M, TRIM69, C15orf43, SORD, DUOX2, DUOXA2, DUOXA1, DUOX1, SHF, SLC28A2, GATM, SPATA5L1, C15orf48, SLC30A4, BLOC1S6, SQRDL
chr15	45983324	48053417	M2	RNU6-1014P, RN7SKP101, RN7SKP139, SQRDL, SEMA6D
chr15	50411319	52903452	M2	ATP8B4, SLC27A2, HDC, GABPB1, RN7SL494P, GABPB1-AS1, MIR4712, AHCYP7, RNA5SP395, RN7SL354P, DCAF13P3, MIR4713, MIR1266, EEF1B2P1, USP8, USP50, TRPM7, SPPL2A, AP4E1, TNFAIP8L3, CYP19A1, GLDN, DMXL2, SCG3, LYSMD2, TMOD2, TMOD3, LEO1, MAPK6, BCL2L10, GNB5, MYO5C, MYO5A, ARPP19, FAM214A

chr16	97278	2327974	M14	POLR3K, SNRNP25, RHBDF1, MIR3177, RN7SL367P, SNORA10, SNORA64, SNHG9, RN7SL219P, MIR1225, MIR4516, MIR3180-5, SNORD60, MPG, NPRL3, MIR940, MIR3677, MIR4717, HBZ, HBM, HBA2, HBA1, HBQ1, LUC7L, ITFG3, RGS11, ARHGDIG, PDIA2, AXIN1, MRPL28, TMEM8A, NME4, DECR2, RAB11FIP3, CAPN15, C16orf11, PIGQ, NHLRC4, RAB40C, WFIKKN1, C16orf13, FAM195A, WDR90, RHOT2, RHBDL1, STUB1, JMJD8, WDR24, FBXL16, METRN, FAM173A, CCDC78, HAGHL, NARFL, MSLN, MSLNL, RPUSD1, CHTF18, GNG13, PRR25, LMF1, SOX8, SSTR5, C1QTNF8, CACNA1H, TPSG1, TPSB2, TPSAB1, TPSD1, UBE2I, BAIAP3, TSR3, GNPTG, UNKL, C16orf91, CCDC154, CLCN7, PTX4, TELO2, IFT140, TMEM204, CRAMP1L, HN1L, MAPK8IP3, NME3, MRPS34, EME2, SPSB3, NUBP2, IGFALS, HAGH, FAHD1, MEIOB, HS3ST6, MSRB1, RPL3L, NDUFB10, RPS2, RNF151, TBL3, NOXO1, GFER, SYNGR3, ZNF598, NPW, SLC9A3R2, NTHL1, TSC2, PKD1, RAB26, TRAF7, CASKIN1, MLST8, BRICD5, PGP, E4F1, DNASE1L2, ECI1, RNPS1, ABCA3, HBZP1, MIR5587, MIR3176, MIR662, SSTR5-AS1, PRSS29P, TPSP2, RPS20P2, RPS3AP2
chr16	46503248	56901054	M11	ANKRD26P1, RNU6-845P, RAB43P1, CKBP1, RPL23AP72, RNA5SP424, RNA5SP425, EIF4BP5, NDUFA5P11, UBA52P8, RN7SL54P, MOCS1P1, RPS2P44, KLF8P1, RNU6-257P, ADAM3B, MRPS21P7, MRPS21P8, RPL34P29, RNY4P3, RPL10P14, RNA5SP426, SOD1P2, UNGP1, HNRNPA1P48, RN7SKP142, LINC00919, CASC16, RNA5SP427, RNU6-1153P, FTO-IT1, CRNDE, RN7SL841P, RPL31P56, CES1P2, CES1P1, MIR3935, MT1L, MT1JP, MT1DP, MT1CP, DPPA2P4, MIR138-2, SHCBP1, VPS35, ORC6, MYLK3, C16orf87, GPT2, DNAJA2, NETO2, ITFG1, PHKB, ABCC12, ABCC11, LONP2, SIAH1, N4BP1, CBLN1, C16orf78, ZNF423, CNEP1R1, HEATR3, PAPD5, ADCY7, BRD7, NKD1, SNX20, NOD2, CYLD, SALL1, C16orf97, TOX3, CHD9, RBL2, AKTIP, RPGRIP1L, FTO, IRX3, IRX5, IRX6, MMP2, LPCAT2, CAPNS2, SLC6A2, CES1, CES5A, GNAO1, AMFR, NUDT21, OGFOD1, BBS2, MT4, MT3, MT2A, MT1E, MT1M, MT1A, MT1B, MT1F, MT1G, MT1H, MT1X, NUP93, SLC12A3
chr16	56901054	59788570	M11,M14	RPS24P17, HMGB3P32, RNU6-20P, RN7SL645P, RNU6-269P, RNU6-1110P, RNU6-103P, SNORA46, SNORA50, RNU6-1155P, RN7SL143P, GEMIN8P2, RPS27P27, RNU4-58P, APOOP5, SLC12A3, HERPUD1, CETP, NLRC5, CPNE2, FAM192A, RSPRY1, ARL2BP, PLLP, CCL22, CX3CL1, CCL17, CIAPIN1, COQ9, POLR2C, DOK4, CCDC102A, GPR114, GPR56, GPR97, CCDC135, KATNB1, KIFC3, CNGB1, TEPP, ZNF319, USB1, MMP15, C16orf80, CSNK2A2, CCDC113, PRSS54, GINS3, NDRG4, SETD6, CNOT1, SLC38A7, GOT2
chr16	59788570	66515616	M11	APOOP5, GNPATP, RN7SKP76, RNU6-21P, UBE2FP2, RPS15AP34, LINC00922, RNA5SP428, LINC00920, CDH8, CDH11, CDH5, BEAN1
chr16	66515616	66670484	M11,M14	BEAN1, TK2, CKLF, CKLF-CMTM1, CMTM1, CMTM2, CMTM3, CMTM4
chr16	66670484	68155889	M14	ACD, PARD6A, ENKD1, C16orf86, GFOD2, RANBP10, TSNAXIP1, CENPT, THAP11, NUTF2, EDC4, NRN1L, PSKH1, CTRL, PSMB10, LCAT, SLC12A4, DPEP3, DPEP2, DUS2, DDX28, NFATC3, RN7SL543P, MIR328, RN7SKP118, RNU1-123P, KARSP3, RNU6-359P, CMTM4, DYNC1LI2, CCDC79, NAE1, CA7, PDP2, CDH16, RRAD, FAM96B, CES2, CES3, CES4A, CBFB, C16orf70, B3GNT9, TRADD, FBXL8, HSF4, NOL3, KIAA0895L, EXOC3L1, E2F4, ELMO3, LRRC29, TMEM208, FHOD1, SLC9A5, PLEKHG4, KCTD19, LRRC36, TPPP3, ZDHHC1, HSD11B2, ATP6V0D1, AGRP, FAM65A, CTCF, RLTPR
chr16	68155889	70765463	M14,M9	HSPE1P5, RNA5SP429, FTLP14, RNU6-898P, RPS2P45, RNU6-22P, MIR1538, MIR140, PDXDC2P, MIR1972-2, RN7SL407P, RN7SL279P, RPS27P26, RNU6-23P, SNORD111B, SNORD111, NFATC3, ESRP2, PLA2G15, SLC7A6, SLC7A6OS, PRMT7, SMPD3, ZFP90, CDH3, CDH1, RPS12P27, RNU6-1262P, RNU4-30P, RPL35AP33, TANGO6, HAS3, RNU4-36P, CHTF8, CIRH1A, SNTB2, VPS4A, COG8, PDF, NIP7, TMED6, TERF2, CYB5B, NFAT5, NQO1, NOB1, WWP2, CLEC18A, PDPR, CLEC18C, EXOSC6, AARS, DDX19B, DDX19A, ST3GAL2, FUK, COG4, SF3B3, IL34, MTSS1L, VAC14
chr16	70765463	70883628	M9	VAC14-AS1, VAC14, HYDIN
chr16	70883628	71196633	M4,M9	RNU6ATAC25P, HYDIN
chr16	71196633	73127362	M9	RNU6-1061P, RNU6-208P, SNORA70D, SNORD71, PKD1L3, RPL39P31, ATP5A1P3, RNU6-385P, RNU7-90P, KRT18P18, RNU7-71P, HCCAT5, HYDIN, CMTR2, CALB2, ZNF23, ZNF19, CHST4, TAT, MARVELD3, PHLPP2, AP1G1, ATXN1L, IST1, ZNF821, DHODH, TXNL4B, HP, HPR, DHX38, PMFBP1, ZFHX3
chr16	73127362	88719761	M11,M9	HCCAT5, RPSAP56, RNU6-237P, HSPE1P7, RNU6-758P, RN7SL520P, ATP5F1P7, RNA5SP430, RPL18P13, RN7SKP233, MIR4719, VN2R10P, KRT8P22, LSM3P5, RPS3P7, RNA5SP431, RNU6-1191P, MIR4720, RN7SKP190, RN7SL134P, MIR3182, RNA5SP432, RNA5SP433, COX6CP16, LINC00311, MIR5093, RN7SL381P, MIR1910, RNU1-103P, LINC01082, LINC01081, LINC00917, FENDRR, MIR5189, C16orf47, PSMD7, NPIPB15, CLEC18B, GLG1, RFWD3, MLKL, FA2H, WDR59, ZNRF1, LDHD, ZFP1, CTRB2, CTRB1, BCAR1, CFDP1, TMEM170A, CHST6, CHST5, TMEM231, GABARAPL2, ADAT1, KARS, TERF2IP, CNTNAP4, MON1B, SYCE1L, ADAMTS18, NUDT7, VAT1L, CLEC3A, WWOX, MAF, DYNLRB2, CDYL2, CMC2, CENPN, ATMIN, C16orf46, GCSH, PKD1L2, BCMO1, GAN, CMIP, PLCG2, SDR42E1, HSD17B2, MPHOSPH6, CDH13, HSBP1, MLYCD, OSGIN1, NECAB2, SLC38A8, MBTPS1, HSDL1, DNAAF1, TAF1C, ADAD2, KCNG4, WFDC1, ATP2C2, TLDC1, COTL1, KLHL36, USP10, CRISPLD2, ZDHHC7, KIAA0513, FAM92B, GSE1, GINS2, C16orf74, EMC8, COX4I1, IRF8, FOXF1, MTHFSD, FOXC2, FOXL1, C16orf95, FBXO31, MAP1LC3B, ZCCHC14, JPH3, KLHDC4, SLC7A5, CA5A, BANP, ZNF469, ZFPM1, ZC3H18, IL17C, CYBA, MVD
chr16	88719761	90142293	M11,M14,M9	SNAI3-AS1, MIR4722, LINC00304, RNU6-430P, RNU7-117P, SNORD68, VPS9D1-AS1, AFG3L1P, URAHP, MVD, SNAI3, RNF166, CTU2, PIEZO1, CDT1, APRT, GALNS, TRAPPC2L, PABPN1L, CBFA2T3, ACSF3, CDH15, SLC22A31, ZNF778, ANKRD11, SPG7, RPL13, CPNE7, DPEP1, CHMP1A, SPATA33, CDK10, SPATA2L, VPS9D1, ZNF276, FANCA, SPIRE2, TCF25, MC1R, TUBB3, DEF8, CENPBD1, DBNDD1, GAS8, C16orf3, PRDM7
chr16	90142293	90142338	M11,M9	PRDM7
chr17	6010	6089	M16	DOC2B
chr17	6089	1802848	M14,M16	DOC2B, RPH3AL, C17orf97, FAM101B, VPS53, FAM57A, GEMIN4, GLOD4, RNMTL1, NXN, TIMM22, ABR, BHLHA9, TUSC5, YWHAE, CRK, MYO1C, INPP5K, RPS4XP17, DBIL5P, MIR3183, PITPNA-AS1, RN7SL105P, MIR22HG, PITPNA, SLC43A2, SCARF1, RILP, PRPF8, TLCD2, WDR81, SERPINF2, SERPINF1, SMYD4, RPA1
chr17	1802848	7163960	M14	CAMKK1, P2RX1, ATP2A3, ZZEF1, CYB5D2, ANKFY1, UBE2G1, SPNS3, SPNS2, MYBBP1A, GGT6, SMTNL2, ALOX15, PELP1, ARRB2, MED11, CXCL16, ZMYND15, TM4SF5, VMO1, GLTPD2, PSMB6, PLD2, MINK1, CHRNE, C17orf107, GP1BA, SLC25A11, RNF167, PFN1, ENO3, SPAG7, CAMTA2, INCA1, KIF1C, SLC52A1, ZFP3, ZNF232, USP6, ZNF594, SCIMP, RABEP1, NUP88, RPAIN, C1QBP, DHX33, DERL2, MIS12, NLRP1, WSCD1, AIPL1, FAM64A, PITPNM3, KIAA0753, TXNDC17, MED31, C17orf100, SLC13A5, XAF1, FBXO39, TEKT1, ALOX12, RNASEK, RNASEK-C17orf49, C17orf49, BCL6B, SLC16A13, SLC16A11, CLEC10A, ASGR2, ASGR1, DLG4, ACADVL, DVL2, MIR132, MIR212, RN7SL624P, HNRNPA1P16, SNORD91B, SNORD91A, RN7SL33P, EIF4A1P9, SAMD11P1, RN7SL608P, PHF23, MIR1253, RN7SL605P, OR1E3, OR1P1, OR1D4, OR1D3P, GABARAP, OR3A4P, OR3A5P, OR1AC1P, OR1R1P, CTDNEP1, RNA5SP434, RYKP1, RN7SL774P, TXNP4, RNU6-955P, ATP6V0CP1, RN7SL171P, RN7SL784P, ELP5, RNU7-31P, RNU6-1264P, BTF3P14, RNA5SP435, CLDN7, MIR4520B, MIR4520A, RPL23AP73, ALOX12P2, MIR497HG, RPL7AP64, MIR324, RPA1, RTN4RL1, DPH1, OVCA2, HIC1, SMG6, SRR, TSR1, SGSM2, MNT, METTL16, PAFAH1B1, CLUH, RAP1GAP2, OR1D5, OR1D2, OR1G1, OR1A2, OR1A1, OR3A2, OR3A1, OR1E1, OR3A3, OR1E2, SPATA22, ASPA, TRPV3, TRPV1, SHPK, CTNS, TAX1BP3, P2RX5-TAX1BP3, EMC6, P2RX5, ITGAE, GSG2, C17orf85
chr17	7163960	7722726	M14,M16	CLDN7, SLC2A4, SENP3-EIF4A1, SNORA67, SNORA48, SNORD10, RPL29P2, YBX2, EIF5A, GPS2, NEURL4, ACAP1, KCTD11, TMEM95, TNK1, TMEM256-PLSCR3, TMEM256, NLGN2, SPEM1, C17orf74, TMEM102, FGF11, CHRNB1, ZBTB4, SLC35G6, POLR2A, TNFSF12, TNFSF12-TNFSF13, TNFSF13, SENP3, EIF4A1, CD68, MPDU1, SOX15, FXR2, SHBG, SAT2, ATP1B2, TP53, WRAP53, EFNB3, DNAH2
chr17	7722726	8286519	M14	CHD3, KCNAB3, TRAPPC1, CNTROB, GUCY2D, ALOX15B, ALOX12B, ALOXE3, HES7, PER1, VAMP2, TMEM107, C17orf59, AURKB, CTC1, PFAS, SLC25A35, RANGRF, ARHGEF15, ODF4, KRBA2, RPL26, SCARNA21, MIR4314, SNORD118, LINC00324, DNAH2, KDM6B, TMEM88, LSMD1, CYB5D1
chr17	29701030	30274704	M16	RN7SL79P, RN7SL45P, MIR4724, MIR193A, RNU6ATAC7P, MIR4725, MIR365B, RNU6-1134P, NF1, RAB11FIP4, COPRS, UTP6, SUZ12
chr17	41154888	43345375	M9	SPATA32, RPL21P4, NBR2, LINC00854, LINC00910, RNU6-1137P, RNU6-470P, MIR2117, RNU6-406P, RNU6-971P, RNU6-131P, ASB16-AS1, SHC1P2, RN7SL507P, RNU6-453P, RPL7L1P5, RN7SL258P, RN7SL819P, RN7SL405P, MAP3K14-AS1, MAP3K14, RPL27, IFI35, VAT1, RND2, BRCA1, NBR1, TMEM106A, ARL4D, DHX8, ETV4, MEOX1, SOST, DUSP3, C17orf105, MPP3, CD300LG, MPP2, FAM215A, PPY, PYY, NAGS, TMEM101, LSM12, G6PC3, HDAC5, C17orf53, ASB16, TMUB2, ATXN7L3, UBTF, SLC4A1, RUNDC3A, SLC25A39, GRN, FAM171A2, ITGA2B, GPATCH8, FZD2, C17orf104, CCDC43, DBF4B, ADAM11, GJC1, HIGD1B, EFTUD2, CCDC103, FAM187A, GFAP, KIF18B, C1QL1, DCAKD, NMT1, PLCD3, ACBD4, HEXIM1, HEXIM2, FMNL1
chr17	43345375	43367855	M1,M9	MAP3K14-AS1, MAP3K14
chr17	43367855	43368131	M1,M4,M9	MAP3K14
chr17	43368131	43394325	M1,M4	MAP3K14
chr17	43394325	44657088	M1,M17,M4,M9	ARHGAP27, PLEKHM1, CRHR1, SPPL2C, MAPT, STH, KANSL1, ARL17B, LRRC37A, LRRC37A2, ARL17A, MAP3K14, RNA5SP443, RN7SL730P, LRRC37A4P, RN7SL739P, DND1P1, RPS26P8, CRHR1-IT1, MAPT-AS1, RNU7-101P, KANSL1-AS1, RN7SL656P, NSFP1, RN7SL199P, FAM215B
chr17	44657088	44832735	M4,M9	ARL17A, NSF, RPS7P11
chr17	44832735	45259003	M4	NSF, WNT3, WNT9B, GOSR2, RPRML, CDC27, RNU6ATAC3P, MIR5089, LRRC37A17P, RN7SL270P
chr18	20936557	21197791	M16	TMEM241, RIOK3, C18orf8, NPC1, ANKRD29
chr18	74721804	77037196	M9	BDP1P, RNA5SP461, LINC01029, RNU6-655P, MBP, GALR1, SALL3, ATP9B

chr19	110678	8987334	M16	PCP2, STXBP2, RETN, C19orf59, TRAPPC5, FCER2, CLEC4G, CD209, CLEC4M, EVI5L, LRRC8E, MAP2K7, TGFBR3L, SNAPC2, CTXN1, TIMM44, ELAVL1, CCL25, FBN3, CERS4, CD320, NDUFA7, RPS28, KANK3, ANGPTL4, RAB11B, MARCH2, HNRNPM, PRAM1, ZNF414, MYO1F, ADAMTS10, ACTL9, OR2Z1, ZNF558, MBD3L1, MUC16, OR4F8P, CICP19, LINC01002, RNU6-1076P, VN2R11P, RNA5SP462, RPS2P52, MIR4745, MIR3187, RNU6-9, RNU6-2, HMGB2P1, CIRBP-AS1, RPS15P9, RN7SL477P, RNU6-1223P, MIR1909, CSNK1G2-AS1, RN7SL226P, MIR1227, MIR4321, SPPL2B, RNU6-993P, TCEB1P28, RN7SL866P, CACTIN-AS1, FTLP5, RN7SL202P, MIR637, SNORD37, RN7SL84P, MIR4746, RN7SL528P, RN7SL121P, MIR7-3HG, MIR7-3, UHRF1, MIR4747, RPL32P34, RN7SL626P, TINCR, SNRPEP4, MIR3940, RPL7P50, EMR4P, RPL21P129, CLEC4GP1, EXOSC3P2, RNA5SP463, RN7SL115P, RAB11B-AS1, MIR4999, RPL23AP78, OR4F17, PPAP2C, MIER2, THEG, C2CD4C, SHC2, ODF3L2, MADCAM1, TPGS1, CDC34, GZMM, BSG, HCN2, POLRMT, FGF22, RNF126, FSTL3, PRSS57, PALM, MISP, PTBP1, AZU1, PRTN3, ELANE, CFD, MED16, R3HDM4, KISS1R, ARID3A, WDR18, GRIN3B, TMEM259, CNN2, ABCA7, HMHA1, POLR2E, GPX4, SBNO2, STK11, C19orf26, ATP5D, MIDN, CIRBP, C19orf24, MUM1, EFNA2, NDUFS7, GAMT, DAZAP1, RPS15, APC2, C19orf25, PCSK4, REEP6, ADAMTSL5, PLK5, MEX3D, MBD3, UQCR11, TCF3, ONECUT3, ATP8B3, REXO1, KLF16, ABHD17A, SCAMP4, ADAT3, CSNK1G2, BTBD2, MKNK2, MOB3A, IZUMO4, AP3D1, DOT1L, PLEKHJ1, SF3A2, AMH, JSRP1, OAZ1, C19orf35, LINGO3, LSM7, TMPRSS9, TIMM13, LMNB2, GADD45B, GNG7, DIRAS1, SLC39A3, SGTA, THOP1, ZNF554, ZNF555, ZNF556, ZNF57, ZNF77, TLE6, TLE2, AES, GNA11, GNA15, S1PR4, NCLN, CELF5, NFIC, C19orf77, DOHH, FZR1, MFSD12, C19orf71, HMG20B, GIPC3, TBXA2R, CACTIN, PIP5K1C, TJP3, APBA3, MRPL54, RAX2, MATK, ZFR2, ATCAY, NMRK2, DAPK3, EEF2, PIAS4, ZBTB7A, MAP2K2, CREB3L3, SIRT6, ANKRD24, EBI3, CCDC94, SHD, TMIGD2, FSD1, STAP2, MPND, SH3GL1, CHAF1A, UBXN6, PLIN4, PLIN5, LRG1, SEMA6B, TNFAIP8L1, C19orf10, DPP9, FEM1A, TICAM1, PLIN3, ARRDC5, KDM4B, PTPRS, ZNRF4, SAFB2, SAFB, RPL36, C19orf70, HSD11B1L, LONP1, CATSPERD, PRR22, DUS3L, NRTN, FUT6, FUT3, FUT5, NDUFA11, VMAC, CAPS, RANBP3, RFX2, ACSBG2, MLLT1, ACER1, CLPP, ALKBH7, PSPN, GTF2F1, KHSRP, SLC25A41, SLC25A23, CRB3, DENND1C, TUBB4A, TNFSF9, CD70, TNFSF14, C3, GPR108, TRIP10, SH2D3A, VAV1, EMR1, MBD3L5, MBD3L4, MBD3L2, MBD3L3, ZNF557, INSR, ARHGEF18, PEX11G, C19orf45, ZNF358, MCOLN1, PNPLA6, CAMSAP3, XAB2, PET100
chr19	10123924	11217301	M16	RDH8, C19orf66, ANGPTL6, PPAN-P2RY11, PPAN, P2RY11, EIF3G, DNMT1, S1PR2, MRPL4, ICAM1, ICAM4, ICAM5, ZGLP1, FDX1L, RAVER1, ICAM3, TYK2, CDC37, PDE4A, KEAP1, S1PR5, ATG4D, KRI1, CDKN2D, AP1M2, SLC44A2, ILF3, QTRT1, DNM2, TMED1, C19orf38, CARM1, YIPF2, C19orf52, SMARCA4, C3P1, MIR5589, SNORD105, SNORD105B, MIR4322, MIR1181, RNU7-140P, MIR1238, ILF3-AS1, MIR638, MIR4748, MIR199A1, RN7SL192P, LDLR
chr19	11217301	11665113	M14,M16	RN7SL298P, RN7SL669P, RN7SL833P, LDLR, SPC24, KANK2, DOCK6, C19orf80, TSPAN16, RAB3D, TMEM205, CCDC159, SWSAP1, EPOR, RGL3, CCDC151, PRKCSH, ELAVL3, ZNF653, ECSIT, CNN1, ELOF1
chr19	11665113	14772930	M16	MIR5695, RN7SL619P, MIR24-2, MIR27A, MIR23A, MIR181C, MIR181D, EEF1DP1, RN7SL231P, SNRPGP15, MIR639, RN7SL337P, RN7SL842P, ITGB1P1, ZNF833P, HNRNPA1P10, RNA5SP464, RNA5SP465, RNA5SP466, RSL24D1P8, RNA5SP467, PPIAP20, PGK1P2, SNORD41, MIR5684, ELOF1, ZNF627, ACP5, ZNF823, ZNF441, ZNF491, ZNF440, ZNF439, ZNF69, ZNF700, ZNF763, ZNF433, ZNF878, ZNF844, ZNF788, ZNF20, ZNF625-ZNF20, ZNF625, ZNF136, ZNF44, ZNF563, ZNF442, ZNF799, ZNF443, ZNF709, ZNF564, ZNF490, ZNF791, MAN2B1, WDR83, WDR83OS, DHPS, FBXW9, TNPO2, C19orf43, ASNA1, BEST2, HOOK2, JUNB, PRDX2, RNASEH2A, RTBDN, MAST1, DNASE2, KLF1, GCDH, SYCE2, FARSA, CALR, RAD23A, GADD45GIP1, DAND5, NFIX, LYL1, TRMT1, NACC1, STX10, IER2, CACNA1A, CCDC130, MRI1, C19orf53, ZSWIM4, NANOS3, C19orf57, CC2D1A, PODNL1, DCAF15, RFX1, RLN3, IL27RA, PALM3, C19orf67, SAMD1, PRKACA, ASF1B, LPHN1, CD97, DDX39A, PKN1, PTGER1, GIPC1, DNAJB1, TECR, NDUFB7, CLEC17A, EMR3
chr19	16199854	19843921	M16	JUND, LSM4, PGPEP1, GDF15, LRRC25, SSBP4, ISYNA1, ELL, FKBP8, RN7SL844P, RN7SL146P, RN7SL835P, RN7SL823P, KXD1, SNORA68, RNA5SP468, RPS18P13, MIR3188, RPL39P38, RN7SL513P, MIR3189, RN7SL155P, RN7SL70P, RNU6-1028P, MIR640, PHF5CP, UBA52, CRLF1, C19orf60, TMEM59L, KLHL26, CRTC1, COMP, UPF1, CERS1, GDF1, COPE, DDX49, HOMER3, SUGP2, ARMC6, SLC25A42, TMEM161A, MEF2BNB-MEF2B, MEF2B, MEF2BNB, RFXANK, NR2C2AP, NCAN, HAPLN4, TM6SF2, SUGP1, MAU2, GATAD2A, TSSK6, NDUFA13, YJEFN3, CILP2, PBX4, LPAR2, GMIP, ATP13A1, ZNF101, ZNF14, TPM4, RAB8A, HSH2D, CIB3, FAM32A, AP1M1, KLF2, EPS15L1, CALR3, C19orf44, CHERP, SLC35E1, MED26, SMIM7, TMEM38A, NWD1, SIN3B, F2RL3, CPAMD8, HAUS8, MYO9B, USE1, OCEL1, NR2F6, USHBP1, BABAM1, ANKLE1, ABHD8, MRPL34, DDA1, ANO8, GTPBP3, PLVAP, BST2, MVB12A, TMEM221, NXNL1, SLC27A1, PGLS, FAM129C, COLGALT1, UNC13A, MAP1S, FCHO1, B3GNT3, INSL3, JAK3, RPL18A, SLC5A5, CCDC124, KCNN1, ARRDC2, IL12RB1, MAST3, PIK3R2, IFI30, MPV17L2, RAB3A, PDE4C, KIAA1683
chr19	28283907	45211132	M9	CYP2A6, FBL, VN1R96P, MIR641, RNU6-945P, RN7SL758P, RNU6-195P, CYP2T2P, CYP2F2P, CYP2G1P, CYP2B7P, CYP2A7, CYP2B6, PAFAH1B3, FCGBP, PRR19, TMEM145, CYP2A7P1, CYP2G2P, CYP2T3P, RPL36P16, RN7SL718P, RN7SL34P, PLEKHA3P1, MEGF8, CEACAMP3, PSMC4, DNAJC19P2, DNAJC19P3, HNRNPA1P52, MIR4323, CYP2A13, LIPE-AS1, CYP2F1, CYP2S1, RNU4-60P, CEACAMP2, CEACAMP1, CEACAMP5, RPS10P28, AXL, CEACAMP6, PSG10P, CEACAMP7, CEACAMP8, CEACAMP9, CEACAMP10, CEACAMP11, CEACAMP4, CD177P1, ZNF546, CNFN, LIPE, ZNF780B, CXCL17, ZNF780A, CEACAM1, RN7SL368P, RNU6-902P, RN7SL53P, NDUFA3P1, ZNF285B, CEACAM20, HNRNPUL1, CEACAM22P, MAP3K10, TTC9B, CNTD2, AKT2, TGFB1, CCDC97, CEACAM8, PSG3, TMEM91, PSG8, PSG1, B9D2, BCKDHA, PSG6, EXOSC5, PSG7, C19orf47, PSG11, B3GNT8, ATP5SL, PLD3, PSG2, PSG5, LINC00662, SLC25A1P5, C19orf69, CEACAM21, CEACAM4, CEACAM7, CEACAM5, HIPK4, PRX, LINC00906, SERTAD1, RNA5SP470, RN7SL340P, RPL9P32, TAF9P3, PSG4, SERTAD3, RNA5SP471, RNU6-967P, BLVRB, RNA5SP472, SPTBN4, PSG9, RPS12P31, RN7SL789P, RN7SKP22, RPL31P60, CEBPA-AS1, RPS3AP50, CEACAM6, AKR1B1P7, RPL21P131, RN7SL150P, RPS4XP23, CD177, RPS4XP20, CEACAM3, RPS4XP21, CHCHD2P3, RPL29P33, RN7SL154P, RPS26P55, ZNF807, LYPD4, SCGB1B2P, SCGB2B3P, DMRTC2, RPS19, LINC00904, HPN-AS1, FAM187B2P, MIR5196, CD79A, EEF1A1P7, RN7SL491P, SHKBP1, TEX101, LYPD3, ARHGEF1, PHLDB3, RN7SL765P, UPK1A-AS1, KMT2B, RN7SL402P, RN7SL287P, LINC00665, CTBP2P7, ETHE1, ZNF575, XRCC1, RPL31P61, ZNF571-AS1, PINLYP, LTBP4, RN7SL663P, IRGQ, ZNF576, RNU6-140P, IFNL3P1, MSRB1P1, IFNL4P1, RN7SL566P, MIR4530, TDGF1P7, RABAC1, ATP1A3, SRRM5, ZNF428, CADM4, RPS29P24, RPS29P25, RPS29P26, PLAUR, IRGC, SMG9, KCNN4, GRIK5, LYPD5, ZNF283, ZNF574, ZNF404, ZNF45, NUMBL, ZNF221, ZNF155, POU2F2, ZNF230, ZNF222, ZNF223, ZNF284, ZNF224, ADCK4, ZNF225, ZNF234, ZNF226, ITPKC, C19orf54, DEDD2, ZNF227, ZNF526, GSK3A, ERF, ZNF235, SNRPA, ZNF233, ZNF112, MIA-RAB4B, MIA, CIC, RAB4B, ZNF285, ZNF229, ZNF180, RAB4B-EGLN2, EGLN2, IGSF23, PVR, CEACAM19, CEACAM16, UQCRFS1, VSTM2B, POP4, PLEKHF1, C19orf12, CCNE1, URI1, ZNF536, TSHZ3, ZNF507, DPY19L3, PDCD5, ANKRD27, RGS9BP, NUDT19, TDRD12, SLC7A9, CEP89, C19orf40, RHPN2, GPATCH1, WDR88, LRP3, SLC7A10, CEBPA, CEBPG, PEPD, CHST8, KCTD15, LSM14A, KIAA0355, GPI, PDCD2L, UBA2, WTIP, SCGB2B2, ZNF302, ZNF181, ZNF599, ZNF30, ZNF792, GRAMD1A, SCN1B, HPN, FXYD3, LGI4, FXYD1, FXYD7, FXYD5, FAM187B, LSR, USF2, HAMP, MAG, CD22, FFAR1, FFAR3, GPR42, FFAR2, KRTDAP, DMKN, SBSN, GAPDHS, TMEM147, ATP4A, HAUS5, RBM42, ETV2, COX6B1, UPK1A, ZBTB32, IGFLR1, U2AF1L4, PSENEN, LIN37, HSPB6, C19orf55, ARHGAP33, PRODH2, NPHS1, KIRREL2, APLP1, NFKBID, HCST, TYROBP, LRFN3, SDHAF1, SYNE4, ALKBH6, CLIP3, THAP8, WDR62, OVOL3, POLR2I, TBCB, CAPNS1, COX7A1, ZNF565, ZNF146, ZFP14, ZFP82, ZNF566, ZNF260, ZNF529, ZNF382, ZNF461, ZNF567, ZNF850, ZNF790, ZNF345, ZNF829, ZNF568, ZNF420, ZNF585A, ZNF585B, ZNF383, HKR1, ZNF527, ZNF569, ZNF570, ZNF793, ZNF540, ZNF571, ZFP30, ZNF781, ZNF607, ZNF573, WDR87, SIPA1L3, DPF1, SPINT2, PPP1R14A, YIF1B, C19orf33, KCNK6, CATSPERG, PSMD8, GGN, SPRED3, FAM98C, RASGRP4, RYR1, MAP4K1, EIF3K, ACTN4, CAPN12, LGALS7, LGALS7B, LGALS4, ECH1, HNRNPL, RINL, SIRT2, NFKBIB, CCER2, SARS2, MRPS12, FBXO17, FBXO27, PAK4, NCCRP1, SYCN, IFNL3, IFNL4, IFNL2, IFNL1, LRFN1, GMFG, SAMD4B, PAF1, MED29, ZFP36, PLEKHG2, RPS16, SUPT5H, TIMM50, DLL3, EID2B, EID2, LGALS13, LGALS16, LGALS14, CLC, LEUTX, DYRK1B
chr19	45211132	50312515	M16,M9	CCDC61, PGLYRP1, IGFL4, IGFL3, IGFL2, IGFL1, HIF3A, PPP5C, CCDC8, PNMAL1, PPP5D1, PNMAL2, APOC1P1, RNU6-611P, CALM3, EIF5AP3, RPS16P9, MIR330, RN7SL836P, MIR642A, MIR769, TGIF1P1, IGFL1P1, RPL12P41, IGFL1P2, RNU6-924P, PTGIR, GAPDHP38, DACT3-AS1, RN7SL364P, GNG8, DACT3, PRKD2, MIR320E, HNRNPMP2, RN7SL533P, MIR3191, MIR3190, NAPA-AS1, RN7SL322P, SNORD23, RPL23AP80, SEC1P, RN7SL345P, RNU6-317P, NUCB1-AS1, NTF6A, NTF6G, NTF6B, RN7SL708P, SLC6A21P, SUMO1P4, MIR4324, SNORD32A, SNORD33, SNORD34, SNORD35A, SNORD35B, MIR150, COX6CP7, RNU6-841P, STRN4, FKRP, SLC1A5, AP2S1, ARHGAP35, NPAS1, TMEM160, ZC3H4, SAE1, BBC3, CCDC9, PRR24, C5AR1, C5AR2, DHX34, MEIS3, SLC8A2, KPTN, NAPA, ZNF541, GLTSCR1, EHD2, GLTSCR2, SEPW1, TPRX1, CRX, TPRX2P, SULT2A1, BSPH1, ELSPBP1, CABP5, PLA2G4C, LIG1, C19orf68, ZNF114, CARD8, CEACAM16, BCL3, CBLC, BCAM, CCDC114, EMP3, PVRL2, TMEM143, TOMM40, SYNGR4, APOE, KDELR1, GRIN2D, APOC1, GRWD1, APOC4-APOC2, APOC4, APOC2, KCNJ14, CLPTM1, CYTH2, RELB, LMTK3, SULT2B1, FAM83E, CLASRP, SPACA4, RPL18, SPHK2, ZNF296, GEMIN7, MARK4, DBP, CA11, NTN5, FUT2, MAMSTR, PPP1R37, NKPD1, TRAPPC6A, BLOC1S3, EXOC3L2, CKM, RASIP1, IZUMO1, FUT1, FGF21, BCAT2, KLC3, ERCC2, HSD17B14, PLEKHA4, PPP1R13L, PPP1R15A, TULP2, CD3EAP, NUCB1, ERCC1, DHDH, BAX, FOSB, FTL, RTN2, GYS1, PPM1N, VASP, RUVBL2, OPA3, GPR4, EML2, LHB, CGB, CGB2, CGB1, CGB5, C19orf83, CGB8, CGB7, GIPR, NTF4, KCNA7, SNRNP70, SNRPD2, QPCTL, LIN7B, FBXO46, SIX5, C19orf73, PPFIA3, DMPK, HRC, TRPM4, DMWD, RSPH6A, SYMPK, SLC6A16, FOXA3, IRF2BP1, MYPOP, NANOS2, NOVA2, CD37, TEAD2, DKKL1, CCDC155, PTH2, GFY, SLC17A7, PIH1D1, ALDH16A1, FLT3LG, RPL13A, RPS11, FCGRT, RCN3, NOSIP, PRRG2, PRR12, RRAS, SCAF1, IRF3, BCL2L12, PRMT1, ADM5, CPT1C, TSKS, AP2A1, FUZ
chr19	50312515	53993628	M9	PTOV1-AS1, MIR4749, MIR4750, MIR4751, SIGLEC16, NAPSB, RN7SL324P, GPR32P1, SNORD88B, SNORD88A, SNORD88C, KLKP1, SIGLEC18P, SIGLEC17P, SIGLEC20P, SIGLEC21P, SIGLEC22P, NIFKP6, SIGLEC26P, SIGLEC27P, SIGLEC28P, SIGLEC29P, RPL9P33, RPL7P51, SPACA6P-AS, MIR99B, MIRLET7E, MIR125A, SPACA6P, HCCAT3, RPL37P23, MIR643, DPPA5P1, RPL39P34, ZNF137P, ZNF888, ZNF816-ZNF321P, ZNF702P, ZNF818P, NDUFV2P1, FAM90A27P, FAM90A28P, VN1R6P, RPL39P36, TPM3P9, ZNF761, TPM3P6, FUZ, MED25, PTOV1, PNKP, AKT1S1, TBC1D17, IL4I1, NUP62, ATF5, SIGLEC11, VRK3, ZNF473, IZUMO2, MYH14, KCNC3, NR1H2, NAPSA, POLD1, SPIB, MYBPC2, FAM71E1, EMC10, JOSD2, ASPDH, LRRC4B, SYT3, C19orf81, SHANK1, CLEC11A, GPR32, ACPT, C19orf48, KLK1, KLK15, KLK3, KLK2, KLK4, KLK5, KLK6, KLK7, KLK8, KLK9, KLK10, KLK11, KLK12, KLK13, KLK14, CTU1, SIGLEC9, SIGLEC7, CD33, SIGLECL1, IGLON5, VSIG10L, ETFB, CLDND2, NKG7, LIM2, C19orf84, SIGLEC10, SIGLEC8, CEACAM18, SIGLEC12, SIGLEC6, ZNF175, SIGLEC5, SIGLEC14, HAS1, FPR1, FPR2, FPR3, ZNF577, ZNF649, ZNF613, ZNF350, ZNF615, ZNF614, ZNF432, ZNF841, ZNF616, ZNF836, PPP2R1A, ZNF766, ZNF480, ZNF610, ZNF880, ZNF528, ZNF534, ZNF578, ZNF808, ZNF701, ZNF83, ZNF611, ZNF600, ZNF28, ZNF468, ZNF320, ZNF816, ZNF321P, ERVV-1, ERVV-2, ZNF160, ZNF415, ZNF347, ZNF665, ZNF677, VN1R2, VN1R4, BIRC8, ZNF845, ZNF525, ZNF765, ZNF813
chr19	53993628	54079950	M1,M9	ZNF813, ZNF331

chr19	54079950	54318242	M1,M16,M9	RN7SL317P, RNU6-698P, MIR512-1, MIR512-2, MIR1323, MIR498, MIR520E, MIR515-1, MIR519E, MIR520F, MIR515-2, MIR519C, MIR1283-1, MIR520A, MIR526B, MIR519B, MIR525, MIR523, MIR518F, MIR520B, MIR518B, MIR526A1, MIR520C, MIR518C, MIR524, MIR517A, MIR519D, MIR521-2, RNU6-803P, MIR520D, MIR517B, MIR520G, MIR516B2, MIR526A2, MIR518E, MIR518A1, RNU6-980P, MIR518D, MIR516B1, MIR518A2, MIR517C, MIR520H, RNU6-982P, MIR521-1, RNU6-751P, MIR522, MIR519A1, MIR527, MIR516A1, MIR1283-2, RNU6-1041P, MIR516A2, MIR519A2, RNU6-165P, HMGN1P32, SEPT7P8, MIR371B, MIR371A, MIR372, MIR373, ZNF331, DPRX, NLRP12
chr19	54318242	54647485	M16,M9	MIR935, NLRP12, MYADM, PRKCG, CACNG7, CACNG8, CACNG6, VSTM1, TARM1, OSCAR, NDUFA3, TFPT, PRPF31, CNOT3
chr19	54647485	55237518	M9	RNU6-1307P, MIR4752, VN1R104P, LENG8-AS1, VN1R105P, LILRP1, LILRP2, CNOT3, LENG1, TMC4, MBOAT7, TSEN34, RPS9, LILRB3, LILRA6, LILRB5, LILRB2, LILRA3, LILRA5, LILRA4, LAIR1, TTYH1, LENG8, LENG9, CDC42EP5, LAIR2, KIR3DX1, LILRA2, LILRB1, LILRA1, LILRB4, KIR3DL1, KIR3DL3, KIR2DL4
chr19	55237518	55239076	M4,M9	KIR3DL1, KIR3DL3, KIR2DL4
chr19	55239076	55365501	M17,M4,M9	KIR3DL2, KIR2DP1, KIR3DP1, KIR3DL1, KIR3DL3, KIR2DL4, KIR2DL3, KIR2DL1, KIR2DS4
chr19	55365501	55451834	M17,M9	KIR3DL2, FCAR, NCR1, NLRP7, RNU6-222P, KIR3DL1
chr19	55451834	59095763	M9	NLRP7, NLRP2, GP6, RDH13, EPS8L1, PPP1R12C, TNNT1, TNNI3, DNAAF3, SYT5, RN7SKP109, RFPL4AP1, VN2R17P, ZNF542, PTPRH, SLC25A36P1, ZNF582-AS1, ZNF667-AS1, TMEM86B, SIGLEC31P, PPP6R1, OR5AH1P, MIMT1, RPL7AP69, VN1R107P, VN2R19P, LETM1P2, HNRNPDLP4, RN7SL526P, RPS15AP36, ERVK3-1, HSPBP1, A1BG-AS1, RNA5SP473, MIR4754, RNU6-1337P, RN7SL693P, RN7SL525P, CENPBD1P1, BRSK1, TMEM150B, SUV420H2, COX6B2, FAM71E2, IL11, TMEM190, TMEM238, RPL28, UBE2S, SHISA7, ISOC2, ZNF628, NAT14, SSC5D, SBK2, SBK3, ZNF579, FIZ1, ZNF524, ZNF865, ZNF784, ZNF580, ZNF581, CCDC106, U2AF2, EPN1, NLRP9, RFPL4A, RFPL4AL1, NLRP11, NLRP4, NLRP13, NLRP8, NLRP5, ZNF787, ZNF444, GALP, ZSCAN5B, ZSCAN5C, ZSCAN5A, ZSCAN5D, ZNF582, ZNF583, ZNF667, ZNF471, ZFP28, ZNF470, ZNF71, SMIM17, ZNF835, ZIM2, PEG3, USP29, ZIM3, DUXA, ZNF264, AURKC, ZNF805, ZNF460, ZNF543, ZNF304, ZNF547, TRAPPC2P1, ZNF548, ZNF17, ZNF749, VN1R1, ZNF772, ZNF419, ZNF773, ZNF549, ZNF550, ZNF416, ZIK1, ZNF530, ZNF134, ZNF211, ZSCAN4, ZNF551, ZNF154, ZNF671, ZNF776, ZNF586, ZNF552, ZNF587B, ZNF814, ZNF587, ZNF417, ZNF418, ZNF256, C19orf18, ZNF606, ZSCAN1, ZNF135, ZSCAN18, ZNF329, ZNF274, ZNF544, ZNF8, ZSCAN22, A1BG, ZNF497, ZNF837, RPS5, ZNF584, ZNF132, ZNF324B, ZNF324, ZNF446, SLC27A5, ZBTB45, TRIM28, CHMP2A, UBE2M, MZF1
chr2	45439	45912	M9	FAM110C
chr2	45912	24080284	M14,M9	LINC00487, RNF144A-AS1, RHOB, HS1BP3, FAM110C, GDF7, SH3YL1, C2orf43, RN7SKP112, RNU6ATAC37P, APOB, LINC00298, LINC00299, SNRPEP5, HMGB1P25, RPL30P3, RNU4-73P, MIR4261, RN7SL66P, SNORA80B, RN7SL832P, ACP1, TDRD15, RNU7-138P, KLHL29, RNU7-176P, RNU6-1081P, LINC00570, RNA5SP84, MIR4429, RNU2-13P, RNA5SP85, RN7SL674P, MIR548S, MIR4262, ATAD2B, FAM150B, TMEM18, SNTG2, TPO, MIR3681, RNU6-843P, MIR3125, LINC00276, RNU6-1288P, RNU5E-7P, PXDN, MYCNUN, MYCNOS, RN7SL104P, RN7SKP168, ZFYVE9P2, PSMC1P10, RNU6-1215P, MIR4757, CISD1P1, LINC00954, RN7SL140P, RNU6-961P, RNU7-113P, SLC7A15P, RNA5SP86, HS1BP3-IT1, RN7SL117P, MYT1L, RNA5SP87, RN7SKP27, TSSC1, TRAPPC12, ADI1, RNASEH1, RPS7, COLEC11, ALLC, DCDC2C, SOX11, CMPK2, RSAD2, RNF144A, ID2, KIDINS220, MBOAT2, ASAP2, ITGB1BP1, CPSF3, IAH1, ADAM17, YWHAQ, TAF1B, GRHL1, KLF11, CYS1, RRM2, C2orf48, HPCAL1, ODC1, NOL10, ATP6V1C2, PDIA6, KCNF1, C2orf50, PQLC3, ROCK2, E2F6, GREB1, NTSR2, LPIN1, TRIB2, FAM84A, NBAS, DDX1, MYCN, FAM49A, RAD51AP2, VSNL1, SMC6, GEN1, MSGN1, KCNS3, RDH14, NT5C1B-RDH14, NT5C1B, OSR1, TTC32, WDR35, MATN3, LAPTM4A, SDC1, PUM2, LINC01115, MYT1L-AS1, TSSC1-IT1, TRAPPC12-AS1, RNASEH1-AS1, TMSB4XP2, GAPDHP48, RN7SL531P, NPM1P48, RNU6-649P, LINC01105
chr2	24080284	24087794	M14,M2,M9	ATAD2B
chr2	24087794	24090704	M14,M2	ATAD2B
chr2	24090704	24092532	M14,M2,M9	ATAD2B
chr2	24092532	24398043	M11,M14,M2,M9	ATAD2B, UBXN2A, MFSD2B, C2orf44, FKBP1B, FAM228B, TP53I3, PFN4, FAM228A, PGAM1P6, SDHCP3, RN7SL610P, RNU6-370P
chr2	24398043	24431188	M11,M14,M9	FAM228A, ITSN2
chr2	24431188	27591560	M14,M9	DNAJC5G, TRIM54, UCN, ASXL2, MPV17, KIF3C, RAB10, GTF3C2, GAREML, HADHA, HADHB, EIF2B4, GPR113, EPT1, DRC1, OTOF, ITSN2, C2orf70, HMGN2P20, RPL36AP13, RNA5SP88, RNU6-936P, DNAJC27-AS1, RN7SL856P, SUCLA2P3, MIR1301, TPM3P7, NCOA1, PTGES3P2, UQCRHP2, RNU6-942P, SMARCE1P6, PPIL1P1, CDKN2AIPNLP2, CIB4, KCNK3, AGBL5-AS1, AGBL5-IT1, SLC35F6, CENPA, DPYSL5, PTRHD1, CENPO, ADCY3, MAPRE3, TMEM214, DNAJC27, AGBL5, EFR3B, OST4, POMC, EMILIN1, KHK, DNMT3A, CGREF1, ABHD1, DTNB, PREB, C2orf53, TCF23, SLC5A6, ATRAID, CAD, SLC30A3
chr2	27591560	27873678	M9	EIF2B4, SNX17, ZNF513, PPM1G, NRBP1, KRTCAP3, IFT172, FTH1P3, RNU6-986P, FNDC4, GCKR, C2orf16, ZNF512, CCDC121, GPN1
chr2	27873711	74185272	M9	MIR558, MIR4765, LINC00486, RNA5SP91, RNA5SP92, ATP6V0E1P3, HNRNPA1P61, PPP1CB, RNU6-1145P, MYADML, SLC25A5P2, RPS24P7, RN7SL602P, MIR548AD, RNU6-1117P, MRPL50P1, PPM1B, FAM136A, MIR5192, RN7SL51P, RNU6-577P, RNU6-846P, RN7SL18P, PSAT1P2, RSL24D1P2, RNU6-939P, RNU6-1116P, SPDYA, LINC00211, RMDN2-AS1, CYP1B1-AS1, ETAA1, RNU6-951P, RPL7P12, GAPDHP25, C1D, RPLP0P6, TGFA, DBIL5P2, TTC39DP, ASS1P2, RPS4XP5, RNU6-851P, RN7SL96P, SOS1-IT1, HNRNPA1P66, RNU6-198P, HSPE1P13, RNU6-1185P, LINC00309, MIR4433, TRMT61B, WDR92, RNU6-100P, SLC8A1-AS1, RN7SL341P, RN7SL211P, RNU6-548P, HNRNPA1P57, LDHAP3, RNU4-63P, WDR43, ADD2, RNU6-137P, FTOP1, RNU6-242P, RNU6-958P, RN7SL635P, VTRNA2-2P, KRT18P33, MIR4778, MEIS1-AS3, MEIS1-AS2, FAM161A, PNO1, RN7SKP66, RNU6-1048P, RNU6-566P, SIX3-AS1, MIR3126, RNA5SP96, RNU6-1216P, LINC01121, RN7SL604P, SNORA36C, SLC3A1, RN7SL414P, RN7SL817P, PCBP1-AS1, PPP3R1, BCYRN1, RN7SKP119, MIR559, FAM179A, CNRIP1, RNU6-688P, RPL36AP15, PLEK, CCT4, RN7SKP224, RNU4-49P, RNU6-282P, TPT1P11, TCEB2P3, CTBP2P5, RNU6-439P, RPL7P13, KNOP1P3, CRYGGP, C2orf71, FBXO48, CLIP4, CYP26B1, ZNF863P, FTH1P6, MIR4431, APLF, RNU6-997P, MIR3682, HMGB1P31, RNU7-172P, RPL23AP32, RNU7-81P, RNU6-433P, EXOC6B, PRORSD1P, PROKR1, RN7SL470P, MIR1285-2, BRD7P6, TGFA-IT1, HMGN2P21, ARHGAP25, LINC01143, TCEB1P21, RN7SL160P, OR7E91P, OR7E46P, OR7E62P, BTF3P5, RNU6-105P, RPS20P10, RNU6-775P, RNU6-221P, RNU6-634P, RNU2-39P, RN7SKP208, MIR217, MIR216A, MIR216B, RNA5SP93, RNU6-111P, ALK, ALMS1-IT1, ALMS1P, EIF3FP3, DGUOK-AS1, LINC01122, SPR, RNU6-508P, RNA5SP94, EMX1, RNU1-32P, MIR4432, RN7SL361P, RNU6-612P, ATP1B3P1, RN7SL632P, RNU4-51P, NONOP2, FIGLA, RNA5SP95, CLEC4F, CD207, SNORA70B, COMMD1, B3GNT2, SFXN5, VAX2, ATP6V1B1, TMEM17, EHBP1, BMP10, GKN2, GKN1, PREPL, RAB11FIP5, YPEL5, LBH, LCLAT1, ANKRD53, NOTO, SMYD5, TEX261, ANTXR1, OTX1, CAPN13, PRADC1, WDPCP, NAGK, CCT7, GFPT1, CAMKMT, GALNT14, NFU1, MCEE, MPHOSPH10, MDH1, PAIP2B, ZNF638, CAPN14, UGP2, SIX3, AAK1, SIX2, SRBD1, MIR4263, SNRPGP7, RNA5SP89, FBXO41, SNORD92, SNORD53, RN7SL516P, EHD3, RNA5SP90, SRD5A2, XDH, KRT18P52, AK2P2, DDX50P1, RNU6-647P, ANXA4, EGR4, ALMS1, MEMO1, PRKCE, VPS54, BIRC6-AS1, NAT8, TPRKB, GMCL1, DUSP11, DPY30, SPAST, SLC30A6, PELI1, SNRNP27, LGALSL, MXD1, EPAS1, AFTPH, GPN1, ASPRV1, PCBP1, C2orf42, NLRC4, YIPF4, BIRC6, TMEM247, SERTAD2, DYSF, ATP6V1E2, RHOQ, SLC1A4, SUPT7L, TIA1, SLC4A1AP, TTC27, MRPL33, RBKS, CEP68, LTBP1, PCYOX1, RAB1A, SNRPG, BRE, PIGF, ACTR2, SPRED2, FOSL2, PLB1, MEIS1, CRIPT, RASGRP3, SOCS5, LINC01118, LINC01119, MCFD2, FAM98A, C2orf78, STAMBP, CRIM1, FEZ2, ACTG2, TTC7A, DGUOK, VIT, C2orf61, STRN, HEATR5B, GPATCH11, CALM2, EIF2AK2, SULT6B1, CEBPZ-AS1, CEBPZ, EPCAM, MSH2, NDUFAF7, PRKD3, QPCT, CDC42EP3, KCNK12, RMDN2, MSH6, CYP1B1, ATL2, HNRNPLL, GALM, FBXO11, SRSF7, GEMIN6, DHX57, MORN2, ARHGEF33, SOS1, CDKL4, FOXN2, PPP1R21, MAP4K3, TMEM178A, THUMPD2, SLC8A1, C2orf91, PKDCC, EML4, COX7A2L, KCNG3, MTA3, OXER1, HAAO, STON1, THADA, STON1-GTF2A1L, ZFP36L2, GTF2A1L, PLEKHH2, DYNC2LI1, LHCGR, ABCG5, ABCG8, FSHR, LRPPRC, NRXN1, GPR75-ASB3, CHAC2, ERLEC1, GPR75, PSME4, ACYP2, TSPYL6, C2orf73, SPTBN1, EML6, RTN4, CLHC1, RPS27A, MTIF2, CCDC88A, CCDC104, SMEK2, PNPT1, EFEMP1, CCDC85A, VRK2, FANCL, BCL11A, PAPOLG, REL, PUS10, PEX13, KIAA1841, C2orf74, AHSA2, USP34, XPO1
chr2	74185272	74454257	M17,M9	DGUOK-AS1, RNA5SP97, FNBP1P1, BOLA3-AS1, DGUOK, TET3, BOLA3, MOB1A, MTHFD2, SLC4A5
chr2	74454257	92130494	M9	RNU7-64P, RN7SL251P, RN7SL830P, RN7SL126P, RNU1-38P, RN7SKP83, SNORD94, MIR4779, RNU6-640P, ANAPC1P1, MIR4771-1, MIR4771-2, LINC00152, MIR4435-2, MIR4435-1, MTATP8P2, INO80B, RNU2-63P, MIR4780, RNY4P15, MRPL45P1, RNU6-568P, RNU6-1168P, RNU6-1007P, FOXI3, ANKRD36BP2, WBP1, MOGS, MRPL53, MIR4436A, IGKV7-3, PGBD4P5, IGKV2-4, IGKV2-10, IGKV1-13, IGKV2-14, IGKV2-18, IGKV2-19, IGKV1-22, IGKV2-23, IGKV3-25, IGKV2-26, IGKV2-29, IGKV3-31, IGKV1-32, IGKV3-34, IGKV1-35, IGKV2-36, IGKV2-38, IGKV2D-38, IGKV2D-36, IGKV1D-35, IGKV3D-34, IGKV1D-32, IGKV3D-31, IGKV1D-27, CCDC142, IGKV3D-25, IGKV2D-23, IGKV1D-22, KRT18P26, RNU6-542P, NECAP1P2, IGKV2D-19, IGKV2D-18, TAF13P2, IGKV2D-14, DCTN1-AS1, IGKV2D-10, HMGA1P8, IGKV1OR2-118, LBX2-AS1, TVP23BP2, MIR5000, GAPDHP57, RNA5SP100, DRD5P1, SUCLA2P2, RN7SKP203, RN7SKP164, RNA5SP98, GGT8P, IGKV1OR-1, ABCD1P5, PABPC1P6, CYCSP6, TTC31, SLC9B1P2, RNU6-827P, RNU6-812P, REG1P, ACTR3BP2, RNU6-561P, MIR4264, RNA5SP99, RN7SL201P, RNU6-685P, MTND4P25, MTND5P27, MTND6P7, RPL37P10, LBX2, PCGF1, RNU6-1312P, ST6GALNAC2P1, FUNDC2P2, DUXAP1, LDHAP7, RPL12P18, LSM3P3, TCF7L1-IT1, RNU6-674P, PEBP1P2, RN7SL113P, RNU7-162P, TLX2, DQX1, AUP1, HTRA2, LOXL3, DOK1, M1AP, SEMA4F, HK2, POLE4, TACR1, EVA1A, MRPL19, GCFC2, LRRTM4, REG3G, REG1B, REG1A, REG3A, CTNNA2, LRRTM1, SUCLG1, DNAH6, TRABD2A, TMSB10, KCMF1, TCF7L1, TGOLN2, RETSAT, ELMOD3, SLC4A5, CAPG, SH2D6, DCTN1, MAT2A, GGCX, VAMP8, VAMP5, RNF181, C2orf81, TMEM150A, WDR54, RTKN, USP39, C2orf68, SFTPB, GNLY, ATOH8, ST3GAL5, POLR1A, PTCD3, IMMT, MRPL35, REEP1, KDM3A, CHMP3, RNF103-CHMP3, RNF103, RMND5A, CD8A, CD8B, RGPD1, PLGLB1, IGKV3OR2-268, PLGLB2, RGPD2, KRCC1, SMYD1, FABP1, THNSL2, TEX37, EIF2AK3, RPIA, IGKC, IGKJ5, IGKJ4, IGKJ3, IGKJ2, IGKJ1, IGKV4-1, IGKV5-2, IGKV1-5, IGKV1-6, IGKV3-7, IGKV1-8, IGKV1-9, IGKV3-11, IGKV1-12, IGKV3-15, IGKV1-16, IGKV1-17, IGKV3-20, IGKV6-21, IGKV2-24, IGKV1-27, IGKV2-28, IGKV2-30, IGKV1-33, IGKV1-37, IGKV1-39, IGKV2-40, IGKV2D-40, IGKV1D-39, IGKV1D-37, IGKV1D-33, IGKV2D-30, IGKV2D-29, IGKV2D-28, IGKV2D-26, IGKV2D-24, IGKV6D-21, IGKV3D-20, IGKV6D-41, IGKV1D-17, IGKV1D-16, IGKV3D-15, IGKV1D-13, IGKV1D-12, IGKV3D-11, IGKV1D-42, IGKV1D-43, IGKV1D-8, IGKV3D-7

chr2	95426674	136669063	M9	IL1F10, ZNF514, RPL23AP7, EN1, RNU4-8P, HMGN1P36, RNU7-96P, ATP5BP1, YWHAQP5, MAP4K4, RNU4-84P, RNU7-46P, IL1RN, MARCO, RNU6-744P, MIR4782, LINC01104, SEPHS1P7, RNU2-41P, DPP10-AS3, DPP10-AS2, DPP10-AS1, RPSAP23, MTND1P28, MTND2P21, RNU7-190P, HTR5BP, ZNF2, RN7SL111P, RN7SL611P, HMGN2P22, RN7SL360P, RN7SL468P, NANOGNBP1, STEAP3-AS1, RN7SL548P, SNORD89, MIR5696, MERTK, MIR4772, C1QL2, STEAP3, MTND3P10, MTND4P26, MTND5P28, AHCYP3, LINC01102, LINC01103, RNU4ATAC, NIFK-AS1, C2orf76, LINC01114, RPL12P15, NPM1P32, LIMS1, PSD4, RN7SKP102, MTND5P22, DBI, RNA5SP102, RNU6-259P, PROM2, YWHAZP2, RNU6-675P, RNU7-182P, NIFKP9, HMGB3P11, LINC01158, LINC01159, RNU6-1147P, MIR4783, RNU4-48P, RNY4P7, RNU6-395P, RN7SL206P, PLGLA, DYNLT3P2, TMEM87B, CD8BP, RNA5SP103, ISCA1P6, EEF1A1P12, ST6GAL2-IT1, PPP1R2P5, GACAT1, RGPD4-AS1, RAB6C-AS1, RANBP2, TMEM37, FAR2P1, SCTR, ARHGAP42P2, KCNIP3, KLF2P1, CYP4F27P, SULT1C2P1, SH3RF3-AS1, MIR4265, SNRPGP9, MIR4266, BMS1P19, RPL22P11, GPAA1P1, ZBTB45P1, RNU6-1049P, MED15P9, LINC01123, RHOQP3, PAX8, MED15P5, MTND1P29, MTND2P22, MIR4267, MIR4436B2, MIR4436B1, MTATP6P7, MTND4P27, MTND5P29, MTND6P8, LINC00116, FAR2P2, KLF2P2, CYP4F62P, FAHD2A, RNU6-473P, RNU6-848P, CYP4F30P, FBLN7, FAR2P3, LINC01106, KLF2P3, ZBTB45P2, CYCSP8, NEK2P4, IL1R2, NF1P8, TRIM43, CCDC138, MED15P8, ANKRD36C, RNU6-127P, FAR2P4, ZC3H8, KLF2P4, ARHGAP42P1, RPL5P9, MTND6P10, MIR4435-1HG, MTND5P23, MTND4P21, MTATP6P4, MTND2P18, RPS14P4, MIR4435-2, MIR4435-1, MTND1P26, MED15P3, LINC01120, ZC3H6, MIR4771-1, MIR4771-2, RN7SL297P, TMEM177, GNAQP1, RHOQP2, MIR4784, IL1R1, HMGN2P23, RNU6-1180P, PAX8-AS1, PTPN4, MED15P4, POTEKP, FAM138B, IGKV1OR2-108, MIR1302-3, WASH2P, SNX18P14, CBWD2, EDAR, ANKRD20A8P, RNU6-617P, LINC01087, GRAMD4P8, RN7SL701P, RNU6-1132P, RNA5-8SP5, MIR663B, CDC27P1, RGPD8, ZNF806, FAM201B, RNU6-175P, YBX1P7, RN7SKP103, RNU6-579P, RN7SKP154, NCKAP5-IT1, RN7SKP93, MIR3679, RNA5SP104, DDX11L2, CCNT2-AS1, RNU6-1320P, MIR5590, RNU6-512P, MIR128-1, GPAT2, SH3RF3, RPS24P6, RN7SL575P, SEPT10, IL1RL2, IL1RL1, EPB41L5, ADRA2B, ASTL, DUSP2, FABP7P2, TTL, UBTFL5, STARD7, TRIM43B, IL18R1, TRIM64FP, OR7E102P, POLR1B, TRIM51JP, FOXD4L1, TMEM127, UBTFL3, RABL2A, LINC00342, RN7SL210P, FAHD2CP, STARD7-AS1, NEURL3, SOWAHC, IL18RAP, RGPD5, CIAO1, SLC9A4, FER1L5, SLC9A2, TMEM185B, RALB, INHBB, SNRNP200, LINC01101, MIR3127, RNA5SP101, GLI2, TRIM43CP, MFSD9, IGKV2OR2-1, IGKV2OR2-2, IGKV1OR2-3, TMEM182, RN7SL313P, IGKV1OR2-11, IGKV2OR2-10, IGKV2OR2-7, IGKV1OR2-6, CHCHD5, IGKV3OR2-5, IGKV2OR2-7D, IGKV2OR2-8, IGKV1OR2-9, SLC35F5, SLC20A1, LIMS3, TFCP2L1, MALL, POU3F3, MRPS9, NT5DC4, NPHP1, ITPRIPL1, CKAP2L, ANKRD36B, CLASP1, GPR45, TGFBRAP1, ACTR3, NCAPH, C2orf49, FHL2, IL1A, UBTFL6, LINC01125, ARID5A, IL1B, KANSL3, DPP10, LIMS3L, RGPD6, IL37, IL36G, IL36A, NCK2, IL36B, C2orf40, DDX18, NIFK, IL36RN, UXS1, BUB1, TSN, LMAN2L, CNTNAP5, GYPC, RGPD3, CCDC93, ST6GAL2, RGPD4, CNNM4, CNNM3, ACOXL, INSIG2, BIN1, ANKRD23, BCL2L11, SLC5A7, ANKRD39, SULT1C3, SULT1C2, SEMA4C, ANAPC1, SULT1C4, FAM178B, GCC2, FAHD2B, CYP27C1, ANKRD36, ERCC3, COX5B, MAP3K2, ACTR1B, PROC, ZAP70, TMEM131, IWS1, VWA3B, MYO7B, CNGA3, INPP4A, LIMS2, GPR17, WDR33, COA5, UNC50, MGAT4A, KIAA1211L, TSGA10, SFT2D3, POLR2D, AMMECR1L, SAP130, C2orf15, LIPT1, MITD1, UGGT1, MRPL30, LYG2, LYG1, TXNDC9, EIF5B, HS6ST1, RAB6C, POTEF, REV1, CCDC74B, AFF3, SMPD4, LONRF2, CHST10, NMS, PDCL3, NPAS2, MZT2B, TUBA3E, CCDC115, RPL31, IMP4, PTPN18, TBC1D8, CNOT11, POTEI, CFC1B, RNF149, CFC1, CREG2, RFX8, POTEJ, GPR148, AMER3, ARHGEF4, FAM168B, PLEKHB2, POTEE, TEKT4, MAL, CYP4F31P, MZT2A, MRPS5, TUBA3D, CCDC74A, C2orf27A, C2orf27B, ANKRD30BL, GPR39, LYPD1, NCKAP5, MGAT5, TMEM163, ACMSD, CCNT2, MAP3K19, RAB3GAP1, ZRANB3, R3HDM1, UBXN4, LCT, MCM6, DARS
chr2	149246954	172640949	M9	SP5, ERICH2, GAD1, RNU2-9P, UBBP3, TXNP5, FAM8A3P, RNU6-601P, FABP5P10, MIR4773-2, MIR4773-1, RN7SL124P, RPL30P2, UBQLN4P2, ATP5F1P4, DNAJA1P2, PHBP4, RNA5SP107, CBX3P6, RNU6-1001P, MTND2P20, ATP5A1P2, MTND4P28, MTND5P30, MTND6P9, RNU6-546P, RPLP0P7, RN7SKP281, FAM133DP, RNU6-436P, RNU6-932P, PTP4A1P1, UPP2-IT1, CCDC148-AS1, HNRNPDLP2, RN7SL393P, OR7E89P, OR7E28P, OR7E90P, RNU2-21P, BTF3L4P2, RNU6-580P, GSTM3P2, RMRPP3, MIR4785, RN7SL423P, AHCTF1P1, KRT18P46, RPEP5, TIMM8AP1, EIF3EP2, RNA5SP109, RPL7P61, RNU6-627P, PRPS1P1, CYP2C56P, SNORA70F, RNA5SP110, RNA5SP111, RN7SL455P, MAPRE1P3, TTC21B-AS1, RN7SKP152, XIRP2-AS1, RNU7-148P, CTAGE14P, RN7SL95P, RN7SL813P, MIR4774, RNU6-766P, CERS6-AS1, UBE2V1P6, PTCHD3P2, RNU6-1006P, HMGB1P4, EIF2S2P4, DAP3P2, RPL21P38, GORASP2, TLK1, ARL5A, METTL8, CACNB4, DCAF17, CYBRD1, DYNC1I2, STAM2, FMNL2, SLC25A12, PRPF40A, ARL6IP6, RPRM, GALNT13, KCNJ3, NR4A2, GPD2, GALNT5, ERMN, CYTIP, ACVR1C, ACVR1, UPP2, CCDC148, PKP4, DAPL1, TANC1, WDSUB1, BAZ2B, MARCH7, CD302, LY75, LY75-CD302, PLA2R1, ITGB6, RBMS1, TANK, PSMD14, TBR1, SLC4A10, DPP4, GCG, FAP, IFIH1, GCA, KCNH7, FIGN, GRB14, COBLL1, SLC38A11, SCN3A, SCN2A, CSRNP3, GALNT3, TTC21B, SCN1A, SCN9A, SCN7A, XIRP2, B3GALT1, STK39, CERS6, NOSTRIN, SPC25, G6PC2, ABCB11, DHRS9, LRP2, BBS5, KLHL41, FASTKD1, PPIG, CCDC173, PHOSPHO2, KLHL23, SSB, METTL5, UBR3, MYO3B, LINC01124, MBD5, EPC2, KIF5C, LYPD6B, LYPD6, MMADHC, RND3, RBM43, NMI, TNFAIP6, RIF1, NEB
chr2	172640949	172966281	M14,M9	RNU6-182P, SLC25A12, HAT1, METAP1D, DLX1, DLX2
chr2	172966281	179444956	M9	SCRN3, GPR155, RAPGEF4-AS1, ALDH7A1P2, MLK7-AS1, RPL5P7, RN7SL65P, HNRNPA1P39, RNU6-5P, H3F3AP4, RNU6-763P, RNU6-1290P, MIR933, EXTL2P1, HOXD-AS2, MIR10B, HOXD-AS1, LINC01116, LINC01117, RNU6ATAC14P, FUCA1P1, RNU6-187P, RNA5SP112, KRT8P40, MIR4444-2, MIR4444-1, DNAJC19P5, MIR3128, API5P2, RNU6-629P, CYCTP, RNU5E-9P, WIPF1, TTN-AS1, CHRNA1, CHN1, ATF2, ATP5G3, KIAA1715, EVX2, HOXD13, HOXD12, HOXD11, HOXD10, HOXD9, HOXD8, HOXD3, HOXD4, HOXD1, MTX2, HNRNPA3, NFE2L2, DLX2, AGPS, ITGA6, TTC30B, TTC30A, PDE11A, PDK1, RAPGEF4, RBM45, OSBPL6, PRKRA, DFNB59, FKBP7, PLEKHA3, CDCA7, SP3, OLA1, TTN, SP9, CIR1
chr2	180347998	189460650	M9	DUSP19, NUP35, ZNF804A, FSIP2, MIR1258, FTH1P20, MIR4437, RNU6ATAC19P, KRT18P29, RNA5SP113, RN7SL267P, RPL31P15, RNU6-1122P, KRT8P10, LIN28AP1, CACYBPP2, MIR548AE1, RPL21P32, RPL23AP35, MED28P3, DPRXP1, RN7SKP42, RNU6-989P, GAPDHP59, LINC01090, ST13P2, RNA5SP114, MIR561, ZC3H15, ITGAV, ZNF385B, FAM171B, ZSWIM2, CWC22, CALCRL, TFPI, GULP1, UBE2E3, ITGA4, CERKL, NEUROD1, SSFA2, PPP1R1C, PDE1A, DNAJC10, FRZB, NCKAP1
chr2	190323478	198256699	M9	CCDC150, GTF3C3, OSGEPL1-AS1, HNRNPCP2, RN7SKP179, C2orf66, RNU6-959P, HMGB1P27, RNU6-1045P, DNAJB1P1, PCGEM1, PGAP1, RPS17P8, GLULP6, HNRNPA1P47, RNU6-169P, AHCYP5, RNU6-915P, RN7SL820P, HNRNPA3P15, ANKRD44-IT1, ATP5G2P3, NPM1P46, ANKRD44, SF3B1, WDR75, SLC40A1, ASNSD1, ANKAR, OSGEPL1, ORMDL1, PMS1, C2orf88, MSTN, HIBCH, INPP1, MFSD6, TMEM194B, NAB1, GLS, STAT1, STAT4, MYO1B, NABP1, SDPR, TMEFF2, SLC39A10, DNAH7, STK17B, HECW2
chr2	198256699	198631327	M11,M9	RNU6-1029P, SF3B1, COQ10B, HSPD1, HSPE1, HSPE1-MOB4, MOB4, RFTN2, MARS2, BOLL
chr2	198631327	209116291	M9	PSMA2P3, DAZAP2P1, BZW1, SNORD70, SNORD11B, SNORD11, RN7SL753P, RN7SL40P, MTND4P30, KRT8P15, RPL7P14, RN7SL670P, MRPL50P2, KRT18P39, NPM1P33, RNU6-474P, DSTNP5, RN7SKP178, GCSHP3, SNORD51, SNORA41, RN7SKP200, HMGN1P6, RN7SKP260, HNRNPA1P51, MIR3130-2, MIR3130-1, MIR2355, KLF7-IT1, MIR1302-4, RNU6-664P, PPP1R14BP2, MIR4775, CLK1, RNU6-360P, RNA5SP116, CRYGEP, TPT1P2, CDK15, PPIL3, FZD7, NIF3L1, SUMO1, RNU7-147P, SATB2-AS1, SEPHS1P6, NOP58, RN7SL717P, AOX3P, AOX2P, RNU1-133P, RNU6-31P, RNA5SP115, ORC2, RNU6-312P, RNU6-762P, RN7SL694P, HNRNPA1P35, RNU6-1206P, IMPDH1P10, CFLAR-AS1, RNU7-45P, MTND5P25, MTND4P23, MTND4P29, MTND5P31, ENO1P4, RNU6-651P, UBE2V1P11, RNU6-440P, FAM126B, BMPR2, FAM117B, NDUFB3, CFLAR, ICA1L, WDR12, CASP10, CARF, CASP8, NBEAL1, BOLL, PLCL1, ALS2CR12, TRAK2, CYP20A1, SATB2, ABI2, STRADB, FTCDNL1, C2orf69, TYW5, ALS2CR11, C2orf47, RAPH1, TMEM237, SPATS2L, CD28, CTLA4, ICOS, PARD3B, MPP4, KCTD18, SGOL2, AOX1, ALS2, NRP2, INO80D, NDUFS1, EEF1B2, GPR1, ZDBF2, ADAM23, DYTN, MDH1B, FASTKD2, CPO, KLF7, CREB1, METTL21A, CCNYL1, FZD5, PLEKHM3, CRYGD, CRYGC, CRYGB, CRYGA, C2orf80, IDH1
chr2	209118609	215875194	M9	IDH1-AS1, RNA5SP117, HSPA8P6, CRYGFP, MEAF6P1, PKP4P1, RNA5SP118, SNAI1P1, RNA5SP119, MTND2P23, MIR548F2, PCED1CP, MIR4776-1, MIR4776-2, MIR4438, RPL5P8, VWC2L-IT1, ENSAP3, IDH1, PIKFYVE, PTH2R, MAP2, UNC80, RPE, KANSL1L, ACADL, MYL1, LANCL1, CPS1, ERBB4, IKZF2, SPAG16, VWC2L, BARD1, ABCA12
chr2	215876162	219855016	M9	LINC00607, POLHP1, PSMB3P2, RNA5SP120, FABP5P14, MREG, RN7SKP43, RPL7L1P9, CXCR2P1, HMGB1P9, MIR26B, RN7SKP38, RNU6-136P, RNU6-642P, KRT8P30, LINC00608, PECR, TMEM169, XRCC5, MARCH4, SMARCAL1, RPL37A, IGFBP2, IGFBP5, TNP1, DIRC3, TNS1, RUFY4, CXCR2, CXCR1, ARPC2, GPBAR1, AAMP, PNKD, TMBIM1, C2orf62, SLC11A1, CTDSP1, VIL1, USP37, RQCD1, PLCD4, ZNF142, BCS1L, RNF25, STK36, TTLL4, CYP27A1, PRKAG3, WNT6, WNT10A, CDK5R2, FEV, CRYBA2, ABCA12, ATIC, FN1
chr2	219855016	227598499	M14,M9	SERPINE2, FAM124B, CUL3, DOCK10, MIR375, MIR3131, RN7SL764P, TUBA4B, MIR153-1, MIR3132, RN7SKP213, RPL23P4, MIR4268, TMEM256P2, NYAP2, IRS1, HSPA9P1, NANOGP2, RNU6-619P, GAPDHP49, ATG12P2, RN7SL807P, HIGD1AP4, MIR4439, MIR5702, CRYBA2, CCDC108, IHH, NHEJ1, SLC23A3, CNPPD1, FAM134A, ZFAND2B, ABCB6, ATG9A, ANKZF1, GLB1L, STK16, TUBA4A, DNAJB2, PTPRN, RESP18, DNPEP, DES, SPEG, GMPPA, ASIC4, CHPF, TMEM198, OBSL1, INHA, STK11IP, SLC4A3, EPHA4, PAX3, CCDC140, SGPP2, FARSB, MOGAT1, ACSL3, KCNE4, SCG2, AP1S3, WDFY1, MRPL44
chr2	227598499	243102469	M9	IRS1, RHBDD1, COL4A4, COL4A3, MIR5703, RNA5SP121, SNRPGP8, TDGF1P2, RNU6-624P, RPL7L1P10, RN7SKP283, RNU7-9P, RNU6-613P, RNU6-964P, FBXO36-IT1, RNU6-1027P, RNY4P19, RN7SL834P, HMGB1P3, TPM3P8, RNU6-451P, BANF1P3, RNU6-268P, COX20P2, SPATA3-AS1, RNU1-93P, MIR4777, ZBTB8OSP2, SNORA75, SNORD20, SNORD82, RNU2-22P, LINC00471, RN7SL499P, MIR1471, MIR562, ECEL1P3, ECEL1P2, DIS3L2P1, MIR5001, RN7SL359P, RNU6-107P, RN7SL32P, SCARNA5, SCARNA6, UGT1A12P, UGT1A11P, UGT1A13P, RPL17P11, DNAJB3, UGT1A2P, MSL3P1, HSPE1P9, AGAP1-IT1, TMSB10P1, RNU7-127P, RN7SL204P, RNU1-31P, RPL3P5, RNU6-1051P, RNU6-1140P, RNU6-1333P, RNU6-234P, LINC01107, MIR4440, MIR4441, MIR4269, MIR2467, MIR4786, OR9S24P, OR5S1P, MIR149, CAPN10-AS1, MIR3133, BOK-AS1, RNA5SP122, MFF, CICP10, TM4SF20, AGFG1, C2orf83, SLC19A3, CCL20, DAW1, SPHKAP, PID1, DNER, TRIP12, FBXO36, SLC16A14, SP110, SP140, SP140L, SP100, CAB39, ITM2C, GPR55, SPATA3, C2orf72, PSMD1, HTR2B, ARMC9, B3GNT7, NCL, NMUR1, C2orf57, PTMA, PDE6D, COPS7B, NPPC, DIS3L2, ALPP, ALPPL2, ALPI, ECEL1, PRSS56, CHRND, CHRNG, TIGD1, EIF4E2, EFHD1, GIGYF2, KCNJ13, C2orf82, NGEF, NEU2, INPP5D, ATG16L1, SAG, DGKD, USP40, UGT1A8, UGT1A10, UGT1A9, UGT1A7, UGT1A6, UGT1A5, UGT1A4, UGT1A3, UGT1A1, MROH2A, HJURP, TRPM8, SPP2, ARL4C, SH3BP4, AGAP1, GBX2, ASB18, IQCA1, ACKR3, COPS8, COL6A3, MLPH, PRLH, RAB17, LRRFIP1, RBM44, RAMP1, UBE2F, UBE2F-SCLY, SCLY, ESPNL, KLHL30, FAM132B, ILKAP, HES6, PER2, TRAF3IP1, ASB1, TWIST2, HDAC4, NDUFA10, OR6B2, PRR21, OR6B3, MYEOV2, OTOS, GPC1, ANKMY1, DUSP28, RNPEPL1, CAPN10, GPR35, AQP12B, AQP12A, KIF1A, AGXT, C2orf54, SNED1, MTERFD2, PASK, PPP1R7, ANO7, HDLBP, SEPT2, FARP2, STK25, BOK, THAP4, ATG4B, DTYMK, ING5, D2HGDH, GAL3ST2, NEU4, PDCD1, CXXC11
chr20	60575179	60971463	M16	MIR4758, TAF4, LSM14B, PSMA7, SS18L1, MTG2, HRH3, OSBPL2, ADRM1, LAMA5, RPS21, CABLES2

chr21	30953803	35472860	M14	GRIK1-AS1, LINC00307, RPL8P2, KRTAP13-6P, MIR4327, KRTAP13-5P, KRTAP19-9P, KRTAP19-10P, KRTAP19-11P, KRTAP21-4P, KRTAP8-2P, KRTAP8-3P, KRTAP7-1, UBE3AP2, FBXW11P1, HMGN1P2, TPT1P1, HUNK-AS1, LINC00159, MIS18A-AS1, RN7SL109P, SNORA80, EXOSC3P1, RNA5SP490, LINC00846, OR7E23P, PAXBP1-AS1, LINC00945, IL10RB-AS1, USF1P1, RPS5P3, BTF3P6, LINC00649, RN7SL740P, BACH1, GRIK1, GRIK1-AS2, CLDN17, CLDN8, KRTAP24-1, KRTAP25-1, KRTAP26-1, KRTAP27-1, KRTAP23-1, KRTAP13-2, KRTAP13-1, KRTAP13-3, KRTAP13-4, KRTAP15-1, KRTAP19-1, KRTAP19-2, KRTAP19-3, KRTAP19-4, KRTAP19-5, KRTAP19-6, KRTAP19-7, KRTAP22-2, KRTAP6-3, KRTAP6-2, KRTAP22-1, KRTAP6-1, KRTAP20-1, KRTAP20-4, KRTAP20-2, KRTAP20-3, KRTAP21-3, KRTAP21-2, KRTAP21-1, KRTAP8-1, KRTAP11-1, KRTAP19-8, TIAM1, SOD1, SCAF4, HUNK, MIS18A, MRAP, URB1, C21orf119, EVA1C, TCP10L, C21orf59, SYNJ1, PAXBP1, C21orf49, C21orf62, OLIG2, OLIG1, C21orf54, IFNAR2, IL10RB, IFNAR1, IFNGR2, TMEM50B, DNAJC28, GART, SON, DONSON, CRYZL1, ITSN1, ATP5O, MRPS6, SLC5A3
chr22	41318336	42180424	M16	MIR1281, RNU6-375P, LRRC37A14P, RNU6-495P, RNU6-476P, HMGN2P10, RNU6ATAC22P, XPNPEP3, RBX1, EP300, L3MBTL2, CHADL, RANGAP1, ZC3H7B, TEF, TOB2, PHF5A, ACO2, POLR3H, CSDC2, PMM1, DESI1, XRCC6, NHP2L1, C22orf46, MEI1
chr22	50656186	50965085	M14	RN7SL500P, TUBGCP6, HDAC10, MAPK12, MAPK11, PLXNB2, DENND6B, PPP6R2, SBF1, ADM2, MIOX, LMF2, NCAPH2, SCO2, TYMP
chr3	238649	9729575	M11,M9	CHL1, CNTN6, CNTN4, IL5RA, TRNT1, CRBN, SUMF1, LRRN1, SETMAR, ITPR1, BHLHE40, ARL8B, EDEM1, GRM7, LMCD1, SSUH2, CAV3, OXTR, RAD18, SRGAP3, THUMPD3, SETD5, LHFPL4, MTMR14, CHL1-AS2, RNU6-1194P, RPS8P6, CHL1-AS1, RN7SL120P, RPL23AP39, RPL21P17, RN7SKP144, CNTN4-AS2, DNAJC19P4, CNTN4-AS1, PNPT1P1, MRPS10P2, ITPR1-AS1, EGOT, BHLHE40-AS1, RNF10P1, UBTFL8, MIR4790, RN7SL553P, GRM7-AS3, MRPS36P1, GRM7-AS2, GRM7-AS1, LMCD1-AS1, RNU4ATAC17P, LINC00312, OR7E122P, SRGAP3-AS1, SRGAP3-AS2, SRGAP3-AS3, SRGAP3-AS4, PGAM1P4, SETD5-AS1, DUSP5P2
chr3	9729575	47021402	M9	CCR8, SLC25A38, SLC6A11, RPSA, SLC6A1, RNU6-905P, RHBDF1P1, HRH1, FGD5-AS1, ATG7, MOBP, MYRIP, RPS3AP53, COL6A4P1, VGLL4, EIF1B, ENTPD3, SH3BP5-AS1, RNU6-454P, HMGN2P7, RNU6-1024P, EAF1-AS1, MIR4270, RN7SL110P, MIR3134, RN7SL4P, MIR563, LINC00690, MIR3714, RPL14, PLCL2-AS1, PDCL3P3, ZNF619, TAMM41, TIMP4, ZNF620, ZNF621, RAD23BP1, RNU6-138P, MIR4791, HSPA8P18, PPARG, CTNNB1, RNU4-85P, MIR3135A, SGOL1-AS1, RNY4P22, RNU6-822P, RNU6-815P, VENTXP7, ZNF385D-AS1, ZNF385D-AS2, HMGB1P5, RANP7, SALL4P5, RPL24P7, UBE2E2-AS1, TSEN2, ULK4, RNU6-922P, RNU6-788P, UBE2E1-AS1, LINC00691, NPM1P23, RPL31P20, THRB-IT1, THRB-AS1, MIR4792, EIF3KP2, TRAK1, RN7SL216P, RNA5SP125, CFL1P7, RNA5SP126, MIR4442, CRIP1P2, TAF9BP1, RPEP2, HMGB3P12, C3orf83, MKRN2, RAF1, VENTXP4, MINOS1P3, CCK, LYZL4, RNU6-342P, RN7SL859P, RPS20P15, VIPR1, RNU1-96P, FAM96AP1, LINC00693, TMEM40, MESTP4, RBMS3-AS3, RPS12P5, RBMS3-AS2, RBMS3-AS1, CNN2P6, SEC22C, RNA5SP127, THRAP3P1, OSBPL10-AS1, ZNF587P1, RPL21P40, NIFKP7, KRT18P15, IGBP1P3, RPL30P4, CNOT10-AS1, SUGT1P2, SEC13P1, SUMO2P10, RN7SL296P, CAND2, RNU7-110P, RNA5SP128, COX6CP10, SDAD1P3, FECHP1, KRT8P18, RNU6-243P, RPL32, ARPP21-AS1, MIR128-2, SS18L2, NKTR, IQSEC1, RFC3P1, NBPF21P, NUP210, RN7SKP227, HSPD1P6, ZBTB47, RNU6ATAC4P, PRADC1P1, RPL29P11, UBE2D3P2, KLHL40, CCDC13, RNU6-1301P, HDAC11, HHATL, UBE2FP1, TCEA1P2, RNA5SP129, RNU7-73P, HIGD1A, RPL21P135, NDUFAF4P3, MIR26A1, PPP2R2DP1, ACKR2, MTMR14, RNU6-235P, DLEC1P1, ACVR2B-AS1, DDTP1, RPL18AP7, CPNE9, KRBOX1, FBLN2, RNU6-1227P, DSTNP4, HNRNPA1P21, CYP8B1, ZNF662, BRPF1, EEF1A1P24, SNORA6, SNORA62, NFU1P1, RNU4-56P, RN7SL411P, ENTPD3-AS1, PGAM1P3, RNU5B-2P, RPL5P10, EEF1GP3, HMGN2P24, FAM198A, POMGNT2, OGG1, SNRK, WNT7A, RN7SKP58, ATP6V0E1P2, CAMK1, RPL36P20, TPMTP2, RNU4-78P, SALL4P6, ANO10, EIF4BP4, TADA3, ARPC4, VIPR1-AS1, RN7SL567P, HHATL-AS1, ARPC4-TTLL3, CCDC13-AS1, HNRNPA1P22, KRBOX1-AS1, TTLL3, RN7SL517P, SNRK-AS1, RPUSD3, ABHD5, CIDEC, JAGN1, TOPAZ1, RNU6-367P, MIR138-1, IL17RE, TCAIM, LINC00694, EI24P3, MPRIPP1, ZNF197-AS1, SOCS5P3, MIR564, RPL12P44, RNU5B-3P, RPS24P8, LARS2-AS1, LIMD1-AS1, RN7SL145P, NRBF2P2, FLT1P1, IL17RC, UQCRC2P1, ZNF445, PRSS44, ZNF852, ZKSCAN7, CRELD1, PRRT3, ZNF660, ZNF197, EMC3, FANCD2, ZNF35, ZNF502, ZNF501, TPRXL, CHCHD4, TMEM43, KIAA1143, KIF15, FANCD2OS, BRK1, VHL, IRAK2, XPC, TATDN2, LSM3, GHRL, SLC6A6, SEC13, TMEM42, TGM4, CCDC174, ATP2B2, ZDHHC3, C3orf20, FGD5, EXOSC7, NR2C2, CLEC3B, CDCP1, MRPS25, ZFYVE20, TMEM158, LARS2, CAPN7, SH3BP5, LIMD1, SACM1L, METTL6, EAF1, COLQ, SLC6A20, LZTFL1, HACL1, CCR9, BTD, FYCO1, ANKRD28, CXCR6, XCR1, GALNT15, CCR3, DPH3, OXNAD1, CCR1, CCR2, CCR5, CCRL2, LTF, RFTN1, DAZL, PLCL2, TBC1D5, RTP3, LRRC2, TDGF1, SATB1, KCNH8, ALS2CL, EFHB, RAB5A, PP2D1, KAT2B, TMIE, PRSS50, PRSS46, PRSS45, SGOL1, PRSS42, MYL3, PTH1R, ZNF385D, CCDC12, UBE2E2, NBEAL2, UBE2E1, NKIRAS1, RPL15, NR1D2, THRB, RARB, TOP2B, NGLY1, OXSM, LINC00692, LRRC3B, NEK10, SLC4A7, EOMES, CMC1, AZI2, ZCWPW2, RBMS3, TGFBR2, GADL1, STT3B, OSBPL10, ZNF860, GPD1L, CMTM8, CMTM7, CMTM6, DYNC1LI1, CNOT10, TRIM71, CCR4, GLB1, TMPPE, CRTAP, SUSD5, FBXL2, UBP1, CLASP2, PDCD6IP, ARPP21, STAC, DCLK3, TRANK1, EPM2AIP1, MLH1, LRRFIP2, GOLGA4, C3orf35, ITGA9, CTDSPL, VILL, PLCD1, DLEC1, ACAA1, MYD88, OXSR1, SLC22A13, SLC22A14, XYLB, ACVR2B, EXOG, SCN5A, SCN10A, SCN11A, WDR48, GORASP1, TTC21A, CSRNP1, XIRP1, CX3CR1, RNU6-882P, PRRT3-AS1, EMC3-AS1, CYCSP10, CIDECP, RNU6-670P, CYCSP11, RNU6-814P, LINC00852, GHRLOS, MIR378B, MIR885, ATP2B2-IT1, ATP2B2-IT2, LINC00606, SLC6A1-AS1, CHCHD4P4, FANCD2P2, CYCSP12, NUP210P2, MARK2P14, RN7SL147P, SYN2, ACTG1P12, GSTM5P1, RNU6-404P, RNA5SP123, RNU6-377P, CRIP1P1, KRT18P17, SNORA7A, HDAC11-AS1, LINC00620, VN1R20P, FGD5P1, VN1R21P, RNA5SP124, GRIP2
chr3	93591881	108819263	M9	RNU6-511P, HMGN1P7, RBBP4P2, RNU7-108P, ARMC10P1, WDR82P1, LINC00879, RPS18P6, MTHFD2P1, HNRNPKP4, RNU6-1094P, RPL18AP8, RCC2P5, CDV3P1, OR5BM1P, OR5AC4P, POU5F1P7, OR5H5P, OR5H3P, OR5H4P, OR5H7P, UBFD1P1, RPL38P4, WWP1P1, ST3GAL6-AS1, PDLIM1P4, RNU6-26P, RNU6-1263P, LINC00973, ACTG1P13, MIR3921, VTI1BP1, GMFBP1, RNU6-865P, ACTR3P3, ZNF90P1, FAM172BP, RNU6-1256P, ZBTB11-AS1, PDCL3P4, RNU6-461P, RNU1-43P, NDUFA4P2, MIR548AB, TUBBP11, LINC00882, FCF1P3, MTND4P16, MTND3P6, MTND5P16, MTND6P6, MTND1P16, MTND2P14, RNU6-1308P, LINC00883, LINC00635, LINC00636, HNRNPA1P17, PROS1, ARL13B, STX19, DHFRL1, NSUN3, MTRNR2L12, EPHA6, ARL6, CRYBG3, MINA, GABRR3, OR5AC1, OR5AC2, OR5H1, OR5H14, OR5H15, OR5H6, OR5H2, OR5H8P, OR5K4, OR5K3, OR5K1, OR5K2, CLDND1, CPOX, GPR15, ST3GAL6, DCBLD2, COL8A1, CMSS1, FILIP1L, TMEM30C, TBC1D23, NIT2, TOMM70A, LNP1, TMEM45A, GPR128, TFG, ABI3BP, IMPG2, SENP7, TRMT10C, PCNP, ZBTB11, RPL24, CEP97, NXPE3, NFKBIZ, ZPLD1, ALCAM, CBLB, CCDC54, BBX, CD47, IFT57, HHLA2, MYH15, KIAA1524, DZIP3, RETNLB, TRAT1, GUCA1C, MORC1
chr3	108819263	109214014	M2,M9	MORC1-AS1, LINC00488, RNU6-1236P, H3F3AP3, PPIAP15, MORC1, DPPA2, DPPA4
chr3	109214014	126751442	M9	ITGB5-AS1, ENO1P3, RNA5SP137, RNU6-230P, MIR5092, DUTP1, RNU6-232P, OR7E130P, OR7E29P, OR7E93P, OR7E53P, OR7E97P, MIR548I1, RPS3AP14, ENPP7P4, FAM86JP, ALDH1L1-AS1, ALDH1L1-AS2, WDR52, RNA5SP138, NUP210P1, RCC2P4, SPICE1, SIDT1, KIAA2018, NAA50, ATP6V1A, GRAMD1C, ZDHHC23, KIAA1407, QTRTD1, DRD3, ZNF80, TIGIT, ZBTB20, GAP43, LSAMP, IGSF11, C3orf30, UPK1B, MIR4445, B4GALT4, RNU6ATAC15P, PVRL3-AS1, ATP6V0CP2, PLCXD2-AS1, RFKP2, MIR567, OR7E100P, ARHGAP31, TMEM39A, WDR52-AS1, SIDT1-AS1, MIR4446, RN7SL767P, VPS26AP1, POGLUT1, MIR568, ZBTB20-AS1, ZBTB20-AS2, MIR4796, ZBTB20-AS3, YBX1P3, ZBTB20-AS4, RN7SL815P, LSAMP-AS1, LINC00903, RN7SL582P, BZW1P2, TIMMDC1, TUSC7, MIR4447, LINC00901, RNU5E-8P, PTMAP8, CD80, RNU6-1200P, ADPRH, PLA1A, IGSF11-AS1, B4GALT4-AS1, RPS26P21, ARHGAP31-AS1, RNU6-1127P, PHBP8, RN7SL762P, RN7SL397P, NAP1L1P3, MIR5682, RPL7AP11, RN7SL172P, RNU4-62P, POPDC2, HNRNPA1P23, EIF4BP8, MYLK-AS1, MYLK-AS2, MIR5002, RPL7P15, RNU6-143P, MIR544B, COX17, MAATS1, NR1I2, GSK3B, GPR156, LRRC58, FSTL1, NDUFB4, HGD, RABL3, GTF2E1, STXBP5L, POLQ, ARGFX, FBXO40, HCLS1, GOLGB1, IQCB1, EAF2, SLC15A2, ILDR1, CD86, CASR, CSTA, CCDC58, FAM162A, WDR5B, KPNA1, PARP9, DTX3L, PARP15, PARP14, HSPBAP1, DIRC2, SEMA5B, PDIA5, SEC22A, ADCY5, PTPLB, MYLK, CCDC14, ROPN1, KALRN, UMPS, ITGB5, MUC13, HEG1, SLC12A8, ZNF148, SNX4, OSBPL11, ALG1L, ROPN1B, SLC41A3, ALDH1L1, KLF15, CCDC37, ZXDC, UROC1, CHST13, C3orf22, TXNRD3, TXNRD3NB, CHCHD6, PLXNA1, PVRL3, CD96, ZBED2, PLCXD2, PHLDB2, ABHD10, TAGLN3, TMPRSS7, C3orf52, GCSAM, SLC9C1, CD200, BTLA, ATG3, SLC35A5, CCDC80, CD200R1L, CD200R1, GTPBP8, C3orf17, BOC
chr3	132036210	160968114	M9	GMPS, ANAPC13, ARL14, PPM1L, FOXL2, CEP63, C3orf72, PRR23A, B3GALNT1, MRPS22, KCNAB1, PRR23B, PRR23C, COPB2, EPHB1, NMD3, RBP2, RBP1, KY, NMNAT3, SSR3, TIPARP, LEKR1, PPP2R3A, CLSTN2, CCNL1, TRIM42, SLC25A36, ACPP, MSL2, SPSB4, ACPL2, PCCB, VEPH1, DNAJC13, NIP7P2, HSPA8P19, NPHP3-AS1, ZBTB38, PTX3, C3orf55, ACAD11, BFSP2-AS1, SHOX2, TFP1, RNU6-678P, RNA5SP140, HMGB3P14, RPL39P5, STAG1, HMGN1P9, HMGB3P13, MIR4788, RNU6-1174P, RNA5SP141, TDGF1P6, RNU6-1284P, RNY4P4, HMGN1P10, RNU7-198P, RNU6-789P, RSRC1, NPHP3, IL20RB-AS1, RNA5SP142, NPM1P17, HSPA8P9, KRT8P36, GAPDHP39, RPL23AP40, SLC35G2, RASA2, NCK1, MLF1, EEF1A1P25, ATP5G1P3, RPL7L1P7, BPESC1, RNU6-736P, ACTG1P1, RNF7, GRK7, IL20RB, ATP1B3, GFM1, SOX14, CLDN18, TFDP2, ACKR4, RN7SKP124, RN7SL724P, TRMT112P5, CLSTN2-AS1, UBA5, KRT18P35, RASA2-IT1, YWHAQP6, TPT1P3, DZIP1L, HMGN2P25, RNU6-509P, ATP1B3-AS1, RNU6-425P, RNU6-1294P, RNU1-100P, RPL6P9, RNA5SP143, PLS1-AS1, RN7SKP25, RNU7-47P, TMEM108, A4GNT, DBR1, BFSP2, PBX2P1, SLC9A9-AS1, SLC9A9-AS2, ARMC8, GAPDHP47, LXN, ST13P15, RARRES1, RNA5SP144, GM2AP1, RPL21P39, MFSD1, CDV3, TOPBP1, TF, RNU6-428P, PLSCR5-AS1, NME9, RNU6-505P, RPL21P71, ZIC4-AS1, NPM1P28, GK5, SRPRB, HNRNPA1P20, RPL38P1, HMGB1P30, UBQLN4P1, HLTF-AS1, MED28P2, CPHL1P, MRAS, IQCJ-SCHIP1, RAB6B, TM4SF1-AS1, RNU6-1098P, WWTR1-IT1, WWTR1-AS1, RNU6-507P, RNU6-720P, NPM1P29, HNRNPA1P24, IQCJ, XRN1, RPL32P9, C3orf36, SLCO2A1, SIAH2-AS1, CLRN1-AS1, ESYT3, SCHIP1, RYK, RNA5SP145, SETP11, CEP70, IL12A, LINC01100, C3orf80, IFT80, AMOTL2, ATR, FAIM, PIK3CB, SMC4, PLS1, MRPL42P6, MIR5186, MBNL1-AS1, ATP5LP5, HMGN2P13, RN7SL300P, RNU6-901P, RPL21P42, ARHGEF26-AS1, DDX50P2, RPL9P15, MME-AS1, PABPC1P10, TRIM59, PLCH1-AS1, PLCH1-AS2, RPL6P7, KPNA4, SETP14, VN2R1P, ALG1L15P, KCNAB1-AS2, KCNAB1-AS1, TIPARP-AS1, TRPC1, METTL15P1, LINC00886, PA2G4P4, KLF3P2, PCOLCE2, RN7SKP177, LINC00880, LINC00881, KRT18P34, RNA5SP146, RN7SKP46, MTAPP1, PAQR9, GPR79, MIR3919, IQCJ-SCHIP1-AS1, U2SURP, RNU2-31P, IL12A-AS1, BRD7P2, MIR15B, MIR16-2, RNU7-136P, SCARNA7, KRT8P12, RPL6P8, CHST2, SLC9A9, C3orf58, PLOD2, PLSCR4, PLSCR2, PLSCR1, PLSCR5, ZIC4, ZIC1, AGTR1, CPB1, CPA3, GYG1, HLTF, HPS3, CP, TM4SF18, TM4SF1, TM4SF4, WWTR1, COMMD2, ANKUB1, RNF13, PFN2, TSC22D2, SERP1, EIF2A, FAM194A, SIAH2, FAM188B2, CLRN1, MED12L, GPR171, P2RY14, GPR87, P2RY13, P2RY12, IGSF10, AADACL2, AADAC, SUCNR1, MBNL1, TMEM14E, P2RY1, RAP2B, C3orf79, ARHGEF26, DHX36, GPR149, MME, PLCH1, C3orf33, SLC33A1
chr4	86860	1824786	M14	ZNF595, ZNF732, ZNF141, ZNF721, PIGG, PDE6B, ATP5I, MYL5, MFSD7, PCGF3, CPLX1, GAK, TMEM175, DGKQ, SLC26A1, IDUA, FGFRL1, RNF212, SPON2, CTBP1, MAEA, UVSSA, CRIPAK, NKX1-1, FAM53A, SLBP, TMEM129, TACC3, FGFR3, LETM1, ZNF718, ZNF876P, MIR571, ABCA11P, RN7SL358P, TMED11P, CTBP1-AS2
chr4	69176105	69879871	M4	MT2P1, UGT2B29P, YTHDC1, TMPRSS11E, UGT2B17, UGT2B15, UGT2B10, UGT2A3

chr4	109097266	113739329	M14	LEF1-AS1, RPSAP34, EXOC7P1, RPL34-AS1, RNU6-431P, RCC2P8, RBMXP4, SEC24B-AS1, RN7SL55P, MIR576, SETP20, CDC42P4, HIGD1AP14, KRT19P3, RNU6-35P, RN7SL275P, HSBP1P2, RNU6-205P, ZBED1P1, RPL7L1P13, ZNF969P, MIR297, LYPLA1P2, RNU6-289P, CCDC34P1, TUBB8P3, RTEL1P1, TOX4P1, MIR302B, MIR367, MIR302D, MIR302A, MIR302C, OSTCP4, WRBP1, RPL7AP30, RPL34, OSTC, ETNPPL, COL25A1, SEC24B, CCDC109B, CASP6, PLA2G12A, CFI, GAR1, RRH, LRIT3, EGF, ELOVL6, ENPEP, PITX2, C4orf32, AP1AR, TIFA, ALPK1, NEUROG2, C4orf21, LARP7, ANK2
chr4	189020188	190948412	M4	RNU6-173P, NARG2P1, LINC01060, RNU7-192P, HSP90AA4P, RNU1-51P, MLLT10P2, TUBB7P, RNA5SP174, RNA5SP175, DUX4L9, TRIML2, TRIML1, FRG1, FRG2
chr5	14871460	31423051	M2	BASP1, CDH18, CDH12, PRDM9, C5orf17, CDH10, CDH9, CDH6, DROSHA, UQCRBP3, HNRNPKP5, SEPHS2P1, MARK2P5, MIR887, RNA5SP178, NACAP6, RNA5SP179, RNU6-660P, RNA5SP180, DCAF13P2, RNU6-1003P, RN7SKP133, FTH1P10, RPL36AP21, RN7SL58P, UBE2V1P12, RPL32P14, HSPD1P15, GUSBP1, HSPD1P1, PMCHL1, GCNT1P2, RN7SL572P, RNU6-374P, MSNP1, RNU4-43P, RNU6-738P, LINC01021, RNU6-909P, LSP1P3, SUCLG2P4, UBL5P1, RN7SKP207, RPL19P11, ANKH, FBXL7, MARCH11, ZNF622, FAM134B, MYO10
chr5	68463734	68805646	M16	CFL1P5, CHCHD2P2, RN7SL476P, RPS27P14, RN7SL616P, GUSBP3, CCNB1, CENPH, MRPS36, CDK7, CCDC125, TAF9, RAD17, MARVELD2, OCLN
chr5	133914186	134223872	M16	RNU6-1311P, RNU6-1164P, RNU6-757P, JADE2, SAR1B, SEC24A, CAMLG, DDX46, C5orf24, TXNDC15
chr5	140033522	140951607	M1	PCDHGC5, DIAPH1, VTRNA1-1, VTRNA1-2, VTRNA1-3, PCDHA14, PCDHB9, PCDHB18, PCDHB19P, PCDHGB8P, RN7SL68P, IK, WDR55, DND1, HARS, HARS2, ZMAT2, PCDHA1, PCDHA2, PCDHA3, PCDHA4, PCDHA5, PCDHA6, PCDHA7, PCDHA8, PCDHA9, PCDHA10, PCDHA11, PCDHA12, PCDHA13, PCDHAC1, PCDHAC2, PCDHB1, PCDHB2, PCDHB3, PCDHB4, PCDHB5, PCDHB6, PCDHB7, PCDHB8, PCDHB16, PCDHB10, PCDHB11, PCDHB12, PCDHB13, PCDHB14, PCDHB15, SLC25A2, TAF7, PCDHGA1, PCDHGA2, PCDHGA3, PCDHGB1, PCDHGA4, PCDHGB2, PCDHGA5, PCDHGB3, PCDHGA6, PCDHGA7, PCDHGB4, PCDHGA8, PCDHGA9, PCDHGB6, PCDHGA10, PCDHGB7, PCDHGA11, PCDHGA12, PCDHGC3, PCDHGC4
chr6	28478527	28499554	M4	GPX6, GPX5
chr6	28499554	28500097	M1,M4	GPX5
chr6	28500097	28547048	M1,M2,M4	GPX5, SCAND3
chr6	28547048	28870779	M1,M16,M2,M4,M8	LINC00533, RPSAP2, RPL13P, ZNF90P2, HCG14, SCAND3, TRIM27
chr6	28870779	31322941	M1,M16,M17,M2,M4,M8	RNU6-930P, KRT18P1, RN7SL471P, HCG15, HCG16, OR2AD1P, OR2P1P, OR2N1P, OR2J4P, OR2H4P, OR2G1P, OR2U1P, OR2U2P, OR2B4P, DDX6P1, UBDP1, MAS1LP1, RPS17P1, LINC01015, GPR53P, OR2I1P, OR2H5P, TMEM183AP1, SNORD32B, RPL13AP, SUMO2P1, ZDHHC20P1, HCG4P11, HLA-F-AS1, RPL23AP1, MICE, HCG9P5, IFITM4P, HLA-V, HCG4, HLA-P, RPL7AP7, MICG, HCG4P8, MICF, HCG4P7, HLA-H, HLA-T, DDX39BP1, MCCD1P1, HCG4B, HLA-K, HLA-U, HCG4P5, HLA-W, MICD, HCG9, DDX39BP2, MCCD1P2, ZNRD1-AS1, HCG4P3, HLA-J, ETF1P1, TRIM31-AS1, PAIP1P1, HCG17, TRIM26BP, HLA-L, HCG18, HLA-N, HCG19P, UBQLN1P1, MICC, TMPOP1, SUCLA2P1, RANP1, MIR877, PTMAP1, RPL7P4, MDC1-AS1, RN7SL353P, HCG20, LINC00243, RN7SKP186, DDR1-AS1, MIR4640, RN7SL175P, HCG21, HCG22, RNU6-1133P, POLR2LP, PSORS1C3, USP8P1, RPL3P2, WASF5P, TRIM27, C6orf100, ZNF311, OR2W1, OR2B3, OR2J1, OR2J3, OR2J2, OR14J1, OR5V1, OR12D3, OR12D2, OR12D1, OR11A1, OR10C1, OR2H1, MAS1L, UBD, GABBR1, OR2H2, MOG, ZFP57, HLA-F, HLA-G, HLA-A, ZNRD1, PPP1R11, RNF39, TRIM31, TRIM40, TRIM10, TRIM15, TRIM26, TRIM39, TRIM39-RPP21, RPP21, HLA-E, GNL1, PRR3, ABCF1, PPP1R10, MRPS18B, ATAT1, C6orf136, DHX16, PPP1R18, NRM, MDC1, TUBB, FLOT1, IER3, DDR1, GTF2H4, VARS2, SFTA2, DPCR1, MUC21, MUC22, C6orf15, PSORS1C1, CDSN, PSORS1C2, CCHCR1, TCF19, POU5F1, HCG27, HLA-C, HLA-B
chr6	31322941	31324925	M1,M16,M17,M2,M4,M6,M8	HLA-B
chr6	31324925	31918549	M1,M16,M17,M2,M4,M8	LY6G5B, LY6G5C, ABHD16A, DHFRP2, RNU6-283P, FGFR3P1, ZDHHC20P2, HLA-S, HCP5, LINC01149, PPIAP9, RPL15P4, SNORD117, SNORD84, DDX39B-AS1, UQCRHP1, SNORA38, C6orf47-AS1, MIR4646, RNU6-850P, SAPCD1-AS1, SNORD48, SNORD52, EHMT2-AS1, LY6G6F, LY6G6E, LY6G6D, C6orf25, LY6G6C, DDAH2, CLIC1, MSH5, MSH5-SAPCD1, SAPCD1, VWA7, VARS, LSM2, HSPA1L, HSPA1A, HSPA1B, C6orf48, NEU1, SLC44A4, EHMT2, C2, ZBTB12, CFB, HLA-B, MICA, MICB, MCCD1, ATP6V1G2-DDX39B, DDX39B, ATP6V1G2, NFKBIL1, LTA, TNF, LTB, LST1, NCR3, AIF1, PRRC2A, BAG6, APOM, C6orf47, GPANK1, CSNK2B
chr6	31918549	31919860	M1,M16,M17,M4,M8	CFB
chr6	31919860	31919864	M1,M17,M4,M8	CFB, NELFE
chr6	31919864	31922219	M1,M16,M17,M4,M8	NELFE
chr6	31922219	31922995	M1,M16,M4,M8	NELFE
chr6	31922995	32339656	M1,M16,M17,M4,M8	MIR1236, C4A-AS1, CYP21A1P, TNXA, STK19P, C4B-AS1, RNA5SP206, HNRNPA1P2, RNU6-603P, NELFE, SKIV2L, DXO, STK19, C4A, C4B, CYP21A2, TNXB, ATF6B, FKBPL, PRRT1, PPT2, PPT2-EGFL8, EGFL8, AGPAT1, RNF5, AGER, PBX2, GPSM3, NOTCH4, C6orf10
chr6	32339656	32412821	M16,M17,M4,M8	HCG23, C6orf10, BTNL2, HLA-DRA
chr6	32412821	32712935	M17,M4,M8	HLA-DRB9, RNU1-61P, HLA-DRB6, HLA-DQB1-AS1, MTCO3P1, HLA-DQB3, HLA-DRA, HLA-DRB5, HLA-DRB1, HLA-DQA1, HLA-DQB1, HLA-DQA2
chr6	32712935	33374263	M16,M17,M4,M8	HSD17B8, RING1, VPS52, MIR3135B, TAPSAR1, PPP1R2P1, HLA-Z, BRD2-IT1, RPL32P1, HLA-DPA2, COL11A2P1, HLA-DPB2, HLA-DPA3, HCG24, RNY4P10, MIR219-1, ZNF70P1, HCG25, MYL8P, LYPLA2P1, RPL35AP4, RPL12P1, RPS18, B3GALT4, WDR46, PFDN6, RGL2, TAPBP, ZBTB22, DAXX, KIFC1, HLA-DQA2, HLA-DQB2, HLA-DOB, TAP2, PSMB8, PSMB9, TAP1, HLA-DMB, HLA-DMA, BRD2, HLA-DOA, HLA-DPA1, HLA-DPB1, COL11A2, RXRB, SLC39A7
chr6	33374263	33409536	M16,M17,M4	MIR5004, KIFC1, PHF1, CUTA, SYNGAP1
chr6	33409536	33425318	M16,M4	SYNGAP1, ZBTB9
chr6	33425318	33541991	M16	RN7SL26P, ZBTB9, BAK1
chr6	73713630	73879522	M2	PGAM1P10, KCNQ5-AS1, KCNQ5
chr6	73879522	74520819	M14,M2	KHDC1P1, RPSAP41, EIF3EP1, SDCBP2P1, PAICSP3, OOEP-AS1, RPL39P3, RPS6P8, RNU6-975P, RPS27P15, SLC17A5, CD109, KCNQ5, KHDC1L, KHDC1, DPPA5, KHDC3L, OOEP, DDX43, MB21D1, MTO1, EEF1A1
chr6	74520819	75875222	M14	TXNP7, CD109, COL12A1
chr6	75875222	75953432	M14,M2	COL12A1, COX7A2
chr6	75953432	88776224	M14,M2,M9	HMGB1P39, MIR4463, RNU1-34P, RPL26P20, RNU6-1338P, RNU6-1016P, RN7SKP163, RNU6-155P, RNA5SP209, RNU6-248P, RNU6-261P, RPS6P7, HMGN3-AS1, DBIP1, GAPDHP63, RPL35AP18, AK4P5, RPL17P25, RNA5SP210, RNU6-130P, LAP3P1, SMARCE1P2, KRT18P64, DUTP5, TPT1P6, PKMP3, SNHG5, SMIM11P1, RNU4-72P, RNU4-12P, RPL7P27, NDUFA5P9, RN7SL643P, RPL7P29, MTHFD2P2, RN7SKP209, RCN1P1, HSPD1P10, ST13P16, RNU6-444P, RN7SL183P, COX7A2, TMEM30A, FILIP1, SENP6, MYO6, IMPG1, HTR1B, MEI4, IRAK1BP1, PHIP, HMGN3, LCA5, SH3BGRL2, ELOVL4, TTK, BCKDHB, FAM46A, IBTK, TPBG, UBE3D, DOPEY1, PGM3, RWDD2A, ME1, PRSS35, SNAP91, RIPPLY2, CYB5R4, MRAP2, KIAA1009, TBX18, NT5E, SNX14, SYNCRIP, HTR1E, CGA, ZNF292, GJB7, SMIM8, C6orf163, C6orf164, C6orf165, SLC35A1, RARS2, ORC3, AKIRIN2, SPACA1
chr6	88776224	111067328	M2,M9	RN7SL617P, RNU6-957P, PNISR, USP45, CCNC, ACTBP8, RNU2-61P, RN7SL336P, TUBB3P1, NACAP7, RN7SL11P, DNAJC19P6, CASP8AP2, RN7SKP110, MIR4464, MIR4643, CASC6, RN7SL415P, RPL5P19, ATF1P1, COPS5P1, CYCSP17, MANEA-AS1, KRT18P50, RN7SL797P, UFL1-AS1, RPS7P8, RNU4-70P, EEF1GP6, RN7SL509P, MIR2113, EIF4EBP2P3, MIR548AI, BDH2P1, TSTD3, MCHR2-AS1, NPM1P38, PRDX2P4, R3HDM2P2, NPM1P10, RNU6-897P, LINC00577, RNU6-1106P, BVES-AS1, RPL35P3, RN7SKP211, RN7SL47P, RNU6-344P, RNA5SP211, RNU6-527P, RPL21P65, RNU6-117P, MIR587, RNU6-1299P, RPS24P12, RNU6-437P, RPL23AP50, MTHFD2P3, RPL3P7, OSTM1-AS1, RNA5SP212, RNU6-1144P, RNU6-770P, SUMO2P8, LINC00222, ZNF259P1, ARMC2-AS1, RNU6-653P, PTCHD3P3, CCDC162P, RPL7P28, PRDM13, MCHR2, SIM1, ASCC3, GRIK2, HACE1, LIN28B, BVES, POPDC3, PREP, PRDM1, ATG5, AIM1, RTN4IP1, QRSL1, C6orf203, BEND3, PDSS2, SOBP, SCML4, SEC63, OSTM1, NR2E1, SNX3, LACE1, FOXO3, ARMC2, SESN1, CEP57L1, C6orf183, CD164, PPIL6, SMPD2, MICAL1, ZBTB24, AK9, FIG4, GPR6, WASF1, CDC40, METTL24, DDO, SLC22A16, CDK19, SPACA1, CNR1, RNGTT, PNRC1, SRSF12, PM20D2, GABRR1, GABRR2, UBE2J1, RRAGD, ANKRD6, LYRM2, MDN1, GJA10, BACH2, MAP3K7, EPHA7, MANEA, FUT9, UFL1, FHL5, GPR63, NDUFAF4, KLHL32, MMS22L, POU3F2, FBXL4, FAXC, COQ3
chr6	111067328	111672994	M16,M2,M9	RNU6-1115P, CNN2P9, RNU6-906P, GSTM2P1, RNU6-960P, CDK19, AMD1, GTF3C6, RPF2, SLC16A10, KIAA1919, REV3L

chr6	111672994	147707278	M2,M9	ALDH8A1, GRM1, IL22RA2, FCF1P5, REV3L-IT1, BRD7P4, TRAF3IP2-AS1, RNU6-1226P, KRT18P65, FEM1AP3, RNU6-1163P, FCF1P10, SOCS5P5, IFNGR1, NUDT19P3, RPSAP43, RNA5SP213, DNAJA1P4, RNU6-475P, TPI1P3, NIP7P3, RAB32, CBX3P9, KRT18P22, RPS29P13, RNA5SP214, RN7SKP18, RN7SKP51, RNU6-253P, NEPNP, BRD7P3, SSXP10, MIR548B, ADGB, RNU6-194P, MIR3144, RNU6-214P, RNA5SP215, COX6A1P3, RNU6-1286P, RNU4-35P, RNU4-76P, RNU2-8P, SLC25A5P7, HMGB3P18, RNU1-18P, RN7SL564P, ATP5LP2, NCOA7-AS1, RN7SKP56, RNA5SP216, PPP1R14BP5, MIR588, RNU6-200P, VIMP1, PRELID1P1, RPS4XP9, RNA5SP217, OLIG3, YWHAZP4, PDE7B, RPL5P18, TNFAIP3, EEF1DP5, MESTP1, BMPR1APS1, RNU6-861P, RPL5P21, RPL21P67, HBS1L, RNU4-18P, MIR548H5, RN7SKP245, MIR548AJ1, LINC01013, HIVEP2, EEF1A1P36, RPL21P66, TAAR7P, TAAR4P, TAAR3, HLFP1, CCNG1P1, SNORD101, SNORD100, SNORA33, HMGB1P13, MTFR2, RPL23AP46, LINC00326, MTCYBP4, HSPE1P21, FTH1P26, HMGA1P7, RN7SL408P, RNA5SP218, KRT8P42, CHCHD2P4, LINC01010, PERP, FAM8A6P, STXBP5, MYB-AS1, MIR548A2, LINC00271, KIAA1244, GAPDHP73, HMGB1P17, COX5BP2, NDUFS5P1, RNA5SP219, AIG1, RPL35AP3, BTF3L4P3, BCLAF1, RPSAP42, MARCKSL1P2, MIR3145, RNU6-427P, PBOV1, ATP5F1P6, HEBP2, RNA5SP220, MIR3668, MIR4465, RPS3AP24, RN7SKP106, RPS3AP23, TUBB8P2, RNA5SP221, VDAC1P8, MRPL42P3, TPT1P4, RNU1-33P, NHSL1, FUNDC2P3, RNA5SP222, RNU6-734P, KATNBL1P6, STXBP5-AS1, ADAT2, CCDC28A, PEX3, ECT2L, MAP7, REPS1, FUCA2, MAP3K5, PHACTR2, PEX7, ABRACL, HECA, TXLNB, SLC35D3, IL20RA, CITED2, NMBR, GJE1, LTV1, VTA1, ZC2HC1B, GPR126, PLAGL1, SF3B5, STX11, UTRN, MYB, EPM2A, FBXO30, SHPRH, AHI1, REV3L, TRAF3IP2, FYN, WISP3, TUBE1, FAM229B, LAMA4, RFPL4B, MARCKS, HDAC2, HS3ST5, FRK, NT5DC1, COL10A1, TSPYL4, DSE, TSPYL1, FAM26F, TRAPPC3L, FAM26E, FAM26D, RWDD1, RSPH4A, ZUFSP, KPNA5, FAM162B, GPRC6A, RFX6, VGLL2, ROS1, GOPC, DCBLD1, NUS1, SLC35F1, CEP85L, PLN, MCM9, ASF1A, FAM184A, MAN1A1, TBC1D32, GJA1, HSF2, SERINC1, PKIB, FABP7, SMPDL3A, CLVS2, TRDN, NKAIN2, RNF217, TPD52L1, HDDC2, HEY2, NCOA7, HINT3, TRMT11, CENPW, RSPO3, RNF146, ECHDC1, SOGA3, KIAA0408, C6orf58, THEMIS, PTPRK, LAMA2, ARHGAP18, TMEM244, L3MBTL3, SAMD3, TMEM200A, SMLR1, EPB41L2, AKAP7, ARG1, MED23, ENPP3, OR2A4, CTAGE9, ENPP1, CTGF, MOXD1, STX7, TAAR9, TAAR8, TAAR6, TAAR5, TAAR2, TAAR1, VNN1, VNN3, VNN2, SLC18B1, RPS12, EYA4, TCF21, TBPL1, SLC2A12, SGK1
chr6	147707278	160461673	M14,M2,M9	PCMT1, TIAM2, LRP11, RAET1E, RAET1G, TFB1M, ULBP2, ULBP1, CLDN20, NOX3, RAET1L, ULBP3, PPP1R14C, ARID1B, IYD, MYCT1, VIP, YAP1P1, FBXO5, RNU6-1222P, CYP51A1P3, TMEM242, SNRPEP6, PLEKHG1, RN7SL234P, FABP12P1, RNU7-3P, RPS18P9, SAMD5, CCT7P1, RAET1E-AS1, ZDHHC14, BTF3P10, RAET1K, MTRF1L, RAET1M, SASH1, PHBP1, RNU4-7P, SSR1P1, PDCL3P5, RNU6-302P, RNU6-300P, RNU6-1247P, RNY4P20, RN7SKP268, HSPA8P15, RNU6-813P, SYNE1-AS1, RNA5SP223, HSPD1P16, TUBB4BP7, RNA5SP224, RNA5SP225, MTND4P13, RNU6-896P, HMGB3P19, RPS4XP8, RNU6-824P, MIR1273C, RNU7-152P, MIR1202, MIR4466, LDHAL6FP, MIR3692, SNX9, HSPE1P26, RNU6-786P, SYNJ2-IT1, SRP72P2, RN7SL173P, CACYBPP3, TATDN2P2, AMZ2P2, MIR3918, EZR-AS1, RGS17, OSTCP1, MTHFD1L, RNU6-293P, FNDC1-IT1, HNRNPH1P1, RNU4ATAC18P, SNORA20, SNORA29, UST, AIRN, OPRM1, TAB2, SYNJ2, SUMO4, ZC3H12D, IPCEF1, PPIL4, CNKSR3, AKAP12, SERAC1, ZBTB2, RMND1, GINM1, SCAF8, GTF2H5, TULP4, C6orf211, KATNA1, TMEM181, CCDC170, DYNLT1, LATS1, SYTL3, ESR1, EZR, NUP43, C6orf99, RSPH3, TAGAP, FNDC1, SOD2, WTAP, ACAT2, TCP1, MRPL18, PNLDC1, MAS1, IGF2R, SYNE1
chr6	160461673	170893748	M2,M9	LPAL2, KRT8P44, CAHM, RN7SL366P, RNA5SP226, RNU6-730P, LINC00473, GAPDHP72, RNU6-153P, GNG5P1, HNRNPA1P49, RPS6KA2-IT1, MIR1913, FAM103A2P, RPS6KA2-AS1, MIR3939, MLLT4-AS1, KIF25-AS1, CTAGE13P, LINC00242, LINC00574, RPL12P23, MIR4644, IGF2R, SLC22A1, SLC22A2, SLC22A3, LPA, PLG, MAP3K4, AGPAT4, PARK2, PACRG, QKI, C6orf118, PDE10A, SDIM1, T, PRR18, SFT2D1, MPC1, RPS6KA2, RNASET2, FGFR1OP, CCR6, TCP10L2, GPR31, UNC93A, TTLL2, TCP10, C6orf123, MLLT4, KIF25, FRMD1, DACT2, SMOC2, THBS2, WDR27, C6orf120, PHF10, TCTE3, ERMARD, DLL1, FAM120B, PSMB1, TBP, PDCD2
chr7	4839801	6852668	M16	RADIL, PAPOLB, MMD2, RBAK-RBAKDN, RBAK, WIPI2, SLC29A4, TNRC18, FBXL18, ACTB, FSCN1, RNF216, OCM, CCZ1, RSPH10B, PMS2, AIMP2, EIF2AK1, ANKRD61, USP42, CYTH3, FAM220A, RAC1, DAGLB, KDELR2, GRID2IP, ZDHHC4, C7orf26, ZNF853, ZNF12, RSPH10B2, CCZ1B, RNF216P1, RBAKDN, OR10AH1P, ZNF890P, RNU6-215P, MIR589, RNF216-IT1, ZNF815P, RN7SL556P, RN7SL851P, RNU6-218P, ZNF316, PMS2CL
chr7	148581255	148975618	M16,M17	RN7SL569P, RNY5, RNY4, RNY3, RNY1, RNU6-650P, COX6B1P1, RN7SL521P, EZH2, PDIA4, ZNF786, ZNF425, ZNF398, ZNF282, ZNF212, ZNF783
chr7	148975618	148990933	M17	ZNF783
chr9	14725	14674184	M14	FOXD4, CBWD1, C9orf66, DOCK8, KANK1, DMRT1, DMRT3, DMRT2, SMARCA2, VLDLR, KCNV2, KIAA0020, RFX3, GLIS3, SLC1A1, SPATA6L, PPAPDC2, CDC37L1, AK3, RCL1, JAK2, INSL6, INSL4, RLN2, RLN1, PLGRKT, CD274, PDCD1LG2, KIAA1432, ERMP1, KIAA2026, MLANA, RANBP6, IL33, TPD52L3, UHRF2, C9orf38, GLDC, KDM4C, TMEM261, PTPRD, TYRP1, LURAP1L, MPDZ, NFIB, ZDHHC21, MIR1302-11, MIR1302-10, MIR1302-9, MIR1302-2, FAM138C, RN7SL412P, RNU6-1327P, RNU6-1073P, RPS27AP14, RNA5SP279, RNU2-25P, RN7SL592P, CARM1P1, GLIS3-AS1, RNU6-694P, MIR101-2, HNRNPA1P41, MTND6P5, MTND1P11, MTND4P14, MTND5P14, IGHEP2, HMGN2P31, AK4P4, MIR4665, RN7SL25P, RN7SL123P, RNF2P1, SNRPEP2, RPL4P5, RNU7-185P, RPS26P3, RN7SL5P, IMP3P1, RNU2-47P, RN7SL849P, TDPX2, LINC00583, PES1P2, RPL3P11
chr9	14674184	21141387	M14,M2	ZDHHC21, CER1, FREM1, TTC39B, SNAPC3, PSIP1, CCDC171, C9orf92, BNC2, CNTLN, SH3GL2, ADAMTSL1, FAM154A, RRAGA, HAUS6, PLIN2, DENND4C, RPS6, ACER2, SLC24A2, MLLT3, FOCAD, PTPLAD2, IFNB1, IFNW1, RNU6-1260P, LDHAP4, RNU6-559P, RN7SL157P, RPL7P33, RNU6-319P, RN7SL98P, FTH1P12, RNU6-246P, HMGN2P16, RNU6-14P, RN7SL720P, PABPC1P11, MIR3152, RN7SKP258, SCARNA8, RNU6-264P, RN7SL158P, NDUFA5P3, MIR4473, RNU4-26P, MIR4474, MIR491, IFNNP1, IFNWP4
chr9	21141387	21166147	M2	IFNW1, IFNA21
chr9	21166147	22008992	M14,M2	IFNA21, IFNA4, IFNA7, IFNA10, IFNA16, IFNA17, IFNA14, IFNA5, KLHL9, IFNA6, IFNA13, IFNA2, IFNA8, IFNA1, IFNE, MTAP, C9orf53, CDKN2A, CDKN2B, IFNWP15, IFNWP9, IFNWP18, IFNWP5, IFNA22P, IFNA20P, IFNA11P, IFNA12P, IFNWP2, IFNWP19, MIR31HG, MIR31, KHSRPP1, RN7SL151P, TUBB8P1, CDKN2B-AS1
chr9	22008992	22029512	M2	CDKN2B, CDKN2B-AS1, UBA52P6
chr9	22029512	36058860	M14,M2	SERPINH1P1, RN7SKP24, CNTFR-AS1, FAM205B, FAM205CP, GLULP4, YWHAZP6, SYF2P2, RN7SL338P, ATP8B5P, ZFAND6P1, RPL36AP33, MIR4667, RMRP, RN7SL22P, OR13E1P, NDUFA5P4, LINC00961, PGAM1P2, YBX1P10, OR13C6P, OR2S1P, OR2AM1P, DMRTA1, ELAVL2, IZUMO3, TUSC1, CAAP1, PLAA, IFT74, LRRC19, TEK, EQTN, MOB3B, IFNK, C9orf72, LINGO2, ACO1, DDX58, TOPORS, NDUFB6, GVQW1, TAF1L, TMEM215, APTX, DNAJA1, SMU1, B4GALT1, SPINK4, BAG1, CHMP5, NFX1, AQP7, AQP3, NOL6, ANKRD18B, TRBV20OR9-2, TRBV21OR9-2, TRBV23OR9-2, PRSS3, UBE2R2, UBAP2, DCAF12, UBAP1, KIF24, NUDT2, KIAA1161, C9orf24, FAM219A, DNAI1, ENHO, CNTFR, RPP25L, DCTN3, ARID3C, SIGMAR1, GALT, IL11RA, CCL27, CCL19, CCL21, FAM205A, KIAA1045, DNAJB5, C9orf131, VCP, FANCG, PIGO, STOML2, FAM214B, UNC13B, RUSC2, FAM166B, TESK1, CD72, SIT1, CCDC107, ARHGEF39, CA9, TPM2, TLN1, CREB3, GBA2, RGP1, MSMP, NPR2, SPAG8, HINT2, TMEM8B, FAM221B, OR13J1, HRCT1, OR2S2, OR13C7P, RECK, CDKN2B-AS1, SUMO2P2, RMRPP5, RN7SKP120, RN7SL100P, RNA5SP280, LINC00032, CTAGE12P, MIR876, MIR873, RBMXP2, KRT18P66, KRT18P36, RPS26P2, FTLP4, HSPA8P17, SLC25A6P2, HMGB3P23, RNA5SP281, SLC25A5P8, TOPORS-AS1, ASS1P12, TCEA1P4, RNU4ATAC15P, SNX18P7, CYP4F26P, PRSS3P4, ANXA2P2, TRBV22OR9-2, TRBV24OR9-2, TRBV25OR9-2, PTENP1, TRBV26OR9-2, TRBV29OR9-2, RNU4ATAC11P, SNORD121B, SNORD121A, OSTCP8, RNU7-36P, RN7SKP114, TUBB4BP2, RNA5SP282, RNU2-50P
chr9	36058860	45728282	M2	RNU4-53P, HMGB3P24, MIR4475, MIR4540, MIR4476, CHCHD4P3, RN7SL463P, RAB1C, RN7SKP171, PAICSP1, RNU7-124P, VN1R48P, FAM220BP, CYP4F33P, SNX18P3, FAM201A, RNU6-765P, VN2R3P, RN7SL640P, FAM74A5, ATP5A1P9, RN7SL462P, FAM74A1, VN2R4P, VN2R5P, RN7SL422P, FAM74A3, ATP5A1P1, FAM74A2, FAM74A6, ZNF658B, ATP5A1P8, RBPJP5, RN7SL763P, MEP1AP1, RNU6-1269P, SNX18P8, FAM95B1, CNN2P2, IGKV1OR9-2, MTND1P12, BMS1P14, AQP7P3, CYP4F59P, SNX18P4, RNU6-538P, MEP1AP3, ATP5A1P5, RN7SL343P, CNN2P4, CYP4F60P, SNX18P5, ANKRD20A7P, RNU6-599P, MEP1AP4, RBPJP6, ATP5A1P6, FAM27E4, CDK2AP2P1, MYO5BP1, RN7SL565P, RECK, GLIPR2, CCIN, CLTA, GNE, RNF38, MELK, PAX5, ZCCHC7, GRHPR, ZBTB5, POLR1E, FBXO10, TOMM5, FRMPD1, TRMT10B, EXOSC3, DCAF10, SLC25A51, SHB, ALDH1B1, IGFBPL1, ANKRD18A, CNTNAP3, SPATA31A1, SPATA31A2, SPATA31A3, ZNF658, SPATA31A4, SPATA31A5, ANKRD20A2, CBWD7, FOXD4L2, ANKRD20A3, SPATA31A6, CNTNAP3B, FAM27C, FAM27A
chr9	127074787	128230388	M11	NEK6, PSMB7, GPR144, NR5A1, NR6A1, OLFML2A, WDR38, RPL35, ARPC5L, GOLGA1, SCAI, PPP6C, RABEPK, HSPA5, GAPVD1, MAPKAP1, RN7SL302P, MIR181A2HG, MIR181A2, MIR181B2, RNU4-82P, PRPS1P2, RNU6-1020P
chr9	130119497	131046788	M11	GARNL3, SNORA65, MIR3911, MIR3960, RNA5SP296, MIR4672, SLC2A8, MIR199B, MIR3154, ZNF79, RPL12, LRSAM1, FAM129B, STXBP1, PTRH1, C9orf117, TTC16, TOR2A, SH2D3C, CDK9, FPGS, ENG, AK1, ST6GALNAC6, ST6GALNAC4, PIP5KL1, DPM2, FAM102A, NAIF1, SLC25A25, PTGES2, LCN2, C9orf16, CIZ1, DNM1, GOLGA2, SWI5
chr9	131046788	131749996	M11,M2	TMSB4XP4, MIR219-2, ODF2-AS1, RNU7-171P, VTI1BP4, HMGA1P4, RN7SL560P, SWI5, TRUB2, COQ4, SLC27A4, URM1, CERCAM, ODF2, GLE1, SPTAN1, WDR34, SET, PKN3, ZDHHC12, ZER1, TBC1D13, ENDOG, C9orf114, CCBL1, LRRC8A, PHYHD1, DOLK, NUP188
chr9	131749996	134077104	M11	C9orf106, RN7SL159P, LINC00963, UBE2V1P4, RN7SL665P, RNU6-881P, NUP188, SH3GLB2, FAM73B, DOLPP1, CRAT, PPP2R4, IER5L, NTMT1, C9orf50, ASB6, PRRX2, PTGES, TOR1B, TOR1A, C9orf78, USP20, FNBP1, GPR107, NCS1, HMCN2, ASS1, FUBP3, PRDM12, EXOSC2, ABL1, QRFP, FIBCD1, LAMC3, AIF1L, NUP214
chr9	139912424	140064491	M17	MIR3621, ABCA2, C9orf139, FUT7, NPDC1, ENTPD2, SAPCD2, UAP1L1, MAN1B1, DPP7, GRIN1, LRRC26
chrX	192990	5811707	M2	PLCXD1, GTPBP6, PPP2R3B, SHOX, CRLF2, CSF2RA, IL3RA, SLC25A6, ASMTL, P2RY8, AKAP17A, ASMT, DHRSX, ZBED1, CD99, XG, GYG2, ARSD, ARSE, ARSH, ARSF, CXorf28, MXRA5, PRKX, NLGN4X, LINC00685, FABP5P13, KRT18P53, RPL14P5, MIR3690, RNA5SP498, RN7SL355P, LINC00106, ASMTL-AS1, DHRSX-IT1, CD99P1, LINC00102, GYG2-AS1, ARSD-AS1, RN7SL578P, ASS1P4, RNU6-114P, RNU6-146P, PRKX-AS1, MTND6P12

chrX	15720904	24544391	M2	CA5B, ZRSR2, AP1S2, GRPR, MAGEB17, CTPS2, S100G, SYAP1, TXLNG, RBBP7, REPS2, NHS, SCML1, RAI2, BEND2, SCML2, CDKL5, RS1, PPEF1, PHKA2, GPR64, PDHA1, MAP3K15, SH3KBP1, CXorf23, MAP7D2, EIF1AX, RPS6KA3, CNKSR2, KLHL34, SMPX, MBTPS2, YY2, SMS, PHEX, ZNF645, DDX53, PTCHD1, PRDX4, ACOT9, SAT1, APOO, CXorf58, KLHL15, EIF2S3, ZFX, PDK3, CA5BP1, RNU5F-7P, SETP15, RPL6P30, RN7SL658P, MIR548AM, RNU7-56P, RNU4-6P, CBX1P2, CBX1P4, MIR4768, NHS-AS1, TMSB10P2, GJA6P, PPEF1-AS1, HAUS1P2, PHKA2-AS1, RN7SL48P, MIR23C, EIF1AX-AS1, RN7SKP183, RNU6-133P, RARRES2P3, RN7SL436P, PHEX-AS1, METTL15P3, RNU6-266P, FAM3C2, RPL9P7, ZFX-AS1, SUPT20HL2, SUPT20HL1, SNRPEP9
chrX	40489865	57937067	M2	SLC35A2, PIM2, MIR222, MED28P4, KRT18P68, PCNAP3, OTUD5, ACTBP1, GAPDHP65, RNU6-50P, ZNF674-AS1, MKI67P1, PGAM1P7, YBX1P8, RNU6-1189P, RNU12-2P, INE1, RN7SL785P, RNU6-149P, SNORA11C, NPM1P49, NUS1P1, MIR4769, UXT-AS1, CXXC1P1, KCND1, WASF4P, RPL7P57, ZNF630-AS1, SSX6, RNU6-707P, SSXP3, RNA5SP503, GRIPAP1, SSX9, SSXP9, MRPL32P1, VN1R110P, RNU6-1056P, RNU6-29P, RNU6-722P, RN7SL139P, RNU4-52P, RN7SL262P, SYP-AS1, HSPB1P2, TFE3, VDAC1P2, SALL1P1, USP27X-AS1, RNU6-421P, MIR532, MIR188, MIR500A, MIR362, MIR501, MIR500B, MIR660, MIR502, PYY3, DGKK, CCDC120, PRAF2, WDR45, RNU6-935P, H3F3AP5, HMGB1P15, CENPVP3, CENPVP2, CENPVP1, RNU6-504P, IPO7P1, TPMTP3, SNORA11E, SNORA11D, RBM22P6, RBM22P7, RBM22P10, RBM22P8, RBM22P9, RBM22P11, SSXP4, SSXP1, SSX8, RNA5SP504, SSXP5, GPKOW, MAGIX, EIF4A2P4, PLP2, PRICKLE3, LINC01155, RNU7-37P, ACTG1P10, KDM5C-IT1, VTRNA3-1P, MIR98, MIRLET7F2, SYP, RNA5SP505, RPL7AP71, RNU6-434P, PGAM4P1, SNORA11, MTND2P24, MTND1P30, RPL23AP83, UQCRBP1, PPP1R11P2, MTHFD1P1, CACNA1F, CCDC22, FOXP3, PPP1R3F, GAGE10, GAGE12J, GAGE13, GAGE2E, GAGE2D, GAGE12I, GAGE2C, GAGE2B, GAGE12B, GAGE12C, GAGE12D, GAGE12E, GAGE12F, GAGE12G, GAGE12H, GAGE2A, GAGE1, PAGE1, PAGE4, USP27X, CLCN5, AKAP4, CCNB3, SHROOM4, BMP15, NUDT10, CXorf67, NUDT11, GSPT2, MAGED1, MAGED4B, MAGED4, XAGE2B, XAGE1B, XAGE1A, XAGE2, XAGE1C, XAGE1D, XAGE1E, SSX7, SSX2, SSX2B, SPANXN5, XAGE5, XAGE3, FAM156B, FAM156A, GPR173, TSPYL2, KDM5C, IQSEC2, SMC1A, CXorf38, MED14, USP9X, DDX3X, RIBC1, NYX, CASK, HSD17B10, GPR34, GPR82, MAOA, MAOB, NDP, EFHC2, FUNDC1, DUSP21, KDM6A, HUWE1, CXorf36, KRBOX4, ZNF674, CHST7, SLC9A7, RP2, CXorf31, JADE3, RGN, NDUFB11, RBM10, UBA1, CDK16, USP11, ZNF157, ZNF41, CXorf24, ARAF, SYN1, TIMP1, CFP, ELK1, UXT, ZNF81, ZNF182, ZNF630, PHF8, SPACA5, SPACA5B, SSX5, SSX1, SSX3, SSX4, SSX4B, SLC38A5, FTSJ1, PORCN, EBP, TBC1D25, FAM120C, WNK3, RBM3, WDR13, TSR2, FGD1, WAS, GNL3L, ITIH6, MAGED2, SUV39H1, GLOD5, GATA1, HDAC6, TRO, ERAS, PCSK1N, TIMM17B, PFKFB1, PQBP1, APEX2, MED14-AS1, DPRXP6, ALAS2, MKRN4P, SDCBPP3, RPS2P55, RNA5SP502, RN7SL15P, CASK-AS1, RNU6-1321P, RN7SL406P, RN7SL144P, RNU6-202P, ATP5G2P4, RNU6-630P, RNU6-1124P, PPP1R2P9, IMPDH1P4, NANOGP10, NDP-AS1, TATDN2P1, RRM2P3, PAGE2B, FDPSP5, CHTF8P1, RPL19P20, PAGE2, RN7SL291P, RNU6-523P, KRT8P14, MIR221, FAM104B, MTRNR2L10, PAGE5, PAGE3, MAGEH1, USP51, FOXR2, RRAGB, KLF8, UBQLN2, SPIN3, SPIN2B, SPIN2A, FAAH2, ZXDB, ZXDA
chrX	62567107	70469875	M2	SPIN4-AS1, ARHGEF9-IT1, BTF3P8, MIR1468, HNRNPDP1, RN7SL799P, SHC1P1, YWHAZP7, KRT8P27, GRPEL2P2, CCT4P2, MORF4L1P5, TLE1P1, FRMD8P1, NANOGP9, ETF1P3, MIR223, EIF4BP9, PKMP2, RNU6 394P, RNU6-1225P, RNU6-245P, RPL31P63, COX6CP12, ACTR3P2, SERBP1P1, HMGN1P35, LINC00269, CYCSP43, CNOT7P1, MIR676, MTND4P31, IGBP1-AS2, IGBP1-AS1, RN7SL581P, RNA5SP507, RNY4P23, RNU4-81P, DLG3-AS1, RPS23P8, RNU1-56P, SOCS5P4, RN7SL746P, SPIN4, ARHGEF9, AMER1, ASB12, MTMR8, ZC4H2, ZC3H12B, LAS1L, MSN, VSIG4, HEPH, EDA2R, AR, OPHN1, YIPF6, STARD8, EFNB1, PJA1, FAM155B, EDA, AWAT2, OTUD6A, IGBP1, DGAT2L6, AWAT1, P2RY4, ARR3, RAB41, PDZD11, KIF4A, GDPD2, DLG3, TEX11, SLC7A3, SNX12, FOXO4, CXorf65, IL2RG, MED12, NLGN3, GJB1, ZMYM3
chrX	70469875	70712604	M17,M2	INGX, ZMYM3, NONO, ITGB1BP2, TAF1
chrX	70712604	138827948	M2	PIN4, ERCC6L, RPS4X, NKRF, CITED1, MIR548I4, SETP4, UBE2DNL, MIR361, NDUFA5P7, TPMTP4, EEF1A1P29, GEMIN8P3, MIR1277, RBBP8P1, FCF1P9, ARL5AP1, HNRNPA1P28, RPSAP15, AKAP17BP, MRPS17P9, RNU1-67P, RN7SL118P, SLC25A5-AS1, SEPT6, MIR766, SNORA69, SRIP2, USP12PX, RNF19BPX, UBE2V1P9, NKAPP1, RNU6-555P, PABPC5-AS1, SERBP1P4, RNU2-26P, VDAC1P3, EIF4A1P10, EEF1A1P30, KRT18P11, SNX3P1X, ST13P18, PA2G4P1, RPL7P55, HDAC8, RNU6-332P, PAICSP7, CCNB1IP1P3, MIR548M, CALM1P1, RNA5SP510, HSPA8P1, MIR3672, TPT1P13, RPL3P12, HNRNPDLP3, HNRNPDLP1, RPS7P13, RN7SL379P, RPS29P28, RN7SKP194, RN7SL74P, DIAPH2-AS1, MRRFP1, RPL6P29, EEF1A1P15, HMGB1P32, XRCC6P5, RPSAP8, HNRNPA1P26, TUBB4AP1, FERP1, RN7SL29P, HNRNPA1P27, MEMO1P4, RNU7-69P, CHCHD2P1, XIAP-AS1, RNU6-122P, ZIK1P1, HSPA8P20, TRMT2B-AS1, NPM1P34, MTND4LP1, MTND4P24, RNU6-934P, NANOGNBP3, RPL21P132, SOWAHD, RPL39, ARMCX3-AS1, RNU6-30P, RNU6-587P, RNU6-345P, RPL7AP72, MTND6P13, TCP11X3P, KRT18P44, RPL32P35, RPS26P56, RNA5SP513, UPF3B, FOXN3P1, SALL4P2, NXF4, RNU6-1130P, FAM45B, RN7SL191P, OR11Q1P, OR11N1P, OR5AW1P, OR5BH1P, OR1AA1P, RNU6-589P, LINC00630, MTND1P32, MTND2P2, OR13K1P, OR7L1P, OR2AF1P, HDGFP1, MTND4P32, MTND5P26, RNA5SP514, RAP2C-AS1, RNU6-98P, HS6ST2-AS1, RNU1-115P, RNU6-203P, RPSAP63, TIMM8BP2, MIR363, MIR92A2, MIR19B2, MIR20B, MIR18B, MIR106A, LARP1BP3, PHKA1, MIR450B, MIR450A1, MIR450A2, MIR542, MIR503HG, TCEAL3-AS1, MORF4L2-AS1, RNA5SP511, DPPA3P1, H2BFXP, PHBP10, RPL18AP14, KCTD9P2, RNU6-207P, MIR503, LINC00629, NAP1L4P2, RPL21P133, RNF113A, RPS7P12, RNU4-44P, RNU6-616P, NDUFA1, HMGB3P31, MIR548AN, EEF1A1P40, NCLP2, LINC00087, LINC00633, MYCLP1, AKAP14, DNAJA1P3, KRT18P49, DMRTC1B, FRMPD3-AS1, DMRTC1, RNU6-309P, LINC00086, RNA5SP515, CROCCP1, RPS5P7, DDX26B-AS1, MIR652, MIR3978, SNORD96B, AMMECR1-IT1, TDGF1P3, FCF1P4, GLUD1P9, RNU6-496P, HMGB1P12, LINC00890, PABPC1L2B, RN7SL661P, EIF4BP7, PABPC1L2A, RPL18AP15, RNA5SP512, ALG13-AS1, TRPC5OS, DPRXP7, HMGB3P30, NKAP, MIR4329, RN7SL266P, GAPDHP67, RNU6-1015P, MIR934, RNU6-320P, LINC00892, RNU6-972P, RPL7P56, RANP4, SNORD61, QTRT1P1, RNU1-57P, RN7SL93P, SSU72P1, RPL36AP53, SNORA35, MIR764, MIR1912, MIR1298, MIR1911, SRRM1P3, NAP1L6, NAP1L2, CDX4, CHIC1, RHOXF2B, HSPA8P7, RAC1P4, RHOXF1, MIR448, RHOXF2, ZCCHC13, RNU6-648P, RN7SL325P, ZFYVE9P1, YAP1P2, RNU6-985P, RN7SKP31, SLC16A2, ZBTB33, MAGEA13P, RPL36P18, RLIM, TMEM255A, ASS1P5, EEF1GP5, KIAA2022, RN7SL712P, AKR1B1P8, API5P1, RNU6-154P, KRT8P6, RNU6-1323P, SETP8, ABCB7, MIR504, FGF13-AS1, SNURFL, SRD5A1P1, TUBB4BP3, RPL12P43, UPRT, ATP1B4, ZDHHC15, INGX, LAMP2, CXorf49, CXorf49B, RNU6-1078P, RPS26P11, RN7SL388P, RN7SL264P, RNU2-68P, PHKA1-AS1, RNU1-112P, FAM226B, FAM226A, MAGEE2, PBDC1, LINC00684, MAGEE1, FGF16, ATRX, YWHAZP8, RNU6-1044P, MORF4L1P6, MAP2K4P1, RPL7P53, SEPHS1P4, TSIX, XIST, CUL4B, FXYD6P3, JPX, FTX, MCTS1, RPSAP14, UHRF2P1, DDX3YP1, C1GALT1C1, CT47B1, CT47A12, CT47A11, DDX3YP2, MKRN5P, USMG5P1, BMP2KL, RAB11FIP1P1, CT47A10, MIR421, MIR374B, RN7SL648P, MIR545, MIR374A, RN7SL790P, PRKRIRP1, RPS7P14, MAGT1, RN7SL641P, HNRNPA1P25, CT47A9, PABPC1P3, RNU6-330P, RNU6-562P, RPL21P134, CT47A8, PCNPP4, BRAFP1, CT47A7, TTC3P1, SAR1AP4, CT47A5, CT47A6, CT47A4, RNU6-867P, LDHBP2, RNA5SP508, MIR325, RNA5SP509, CT47A3, FABP5P15, RNU6-854P, RN7SL460P, FNDC3CP, HMGN1P34, UBE2V1P7, PPATP2, RPL7P54, KIF4CP, COX7B, CT47A1, CT47A2, ATP7A, CHMP1B2P, HNRNPH3P1, HK2P1, RNU6-493P, VDAC1P1, RNU6-995P, GLUD2, RNU6-974P, RPL22P22, GRIA3, ATG4AP1, TERF1P4, PGAM4, PGK1, THOC2, TAF9B, XIAP, CYSLTR1, ZCCHC5, LPAR4, P2RY10, GPR174, ITM2A, STAG2, TBX22, FAM46D, BRWD3, SH2D1A, TENM1, HMGN5, DCAF12L2, DCAF12L1, CXorf64, ACTRT1, SMARCA1, SH3BGRL, POU3F4, CYLC1, OCRL, RPS6KA6, HDX, APLN, XPNPEP2, SASH3, APOOL, SATL1, ZDHHC9, ZNF711, POF1B, UTP14A, CHM, DACH2, BCORL1, KLHL4, ELF4, CPXCR1, TGIF2LX, AIFM1, PABPC5, PCDH11X, NAP1L3, FAM133A, DIAPH2, RPA4, PCDH19, RAB33A, ZNF280C, TNMD, TSPAN6, SLC25A14, SRPX2, SYTL4, GPR119, RBMX2, CSTF2, NOX1, ENOX2, XKRX, ARL13A, ARHGAP36, TRMT2B, IGSF1, TMEM35, CENPI, DRP2, OR13H1, FRMD7, RAP2C, TAF7L, MBNL3, TIMM8A, BTK, RPL36A, HS6ST2, RPL36A-HNRNPH2, GLA, HNRNPH2, ARMCX4, ARMCX1, ARMCX6, ARMCX3, USP26, TFDP3, GPC4, GPC3, ARMCX2, CCDC160, PHF6, HPRT1, NXF5, PLAC1, FAM122B, FAM122C, ZMAT1, MOSPD1, TCEAL2, TCEAL6, BEX5, NXF2, SMIM10, FAM127C, FAM127A, FAM127B, CT55, ZNF75D, ZNF449, DDX26B, TCP11X1, NXF2B, CT45A1, CT45A2, CT45A3, TCP11X2, TMSB15A, ARMCX5, CT45A4, CT45A5, GPRASP1, GPRASP2, CT45A6, BHLHB9, SAGE1, RAB40AL, BEX1, NXF3, MMGT1, SLC9A6, BEX4, TCEAL8, FHL1, TCEAL5, BEX2, TCEAL7, WBP5, NGFRAP1, RAB40A, MAP7D3, TCEAL4, TCEAL3, TCEAL1, MORF4L2, GPR112, BRS3, HTATSF1, VGLL1, CD40LG, ARHGEF6, GLRA4, TMEM31, PLP1, RBMX, GPR101, ZIC3, RAB9B, TMSB15B, H2BFWT, H2BFM, SLC25A53, ZCCHC18, FAM199X, FGF13, ESX1, F9, IL1RAPL2, TEX13A, NRK, MCF2, SERPINA7, MUM1L1, CXorf57, RNF128, TBC1D8B, MORC4, ATP11C, RIPPLY1, CLDN2, RBM41, NUP62CL, PIH1D3, FRMPD3, PRPS1, TSC22D3, NCBP2L, MID2, TEX13B, VSIG1, PSMD10, ATG4A, COL4A6, COL4A5, IRS4, GUCY2F, NXT2, KCNE1L, ACSL4, TAF1, TMEM164, AMMECR1, OGT, GNG5P2, RGAG1, CHRDL1, ACRC, CXCR3, NHSL2, RGAG4, PAK3, CAPN6, DCX, ALG13, TRPC5, ZCCHC16, LHFPL1, AMOT, HTR2C, IL13RA2, LRCH2, RBMXL3, LUZP4, PLS3, AGTR2, SLC6A14, CT83, KLHL13, WDR44, DOCK11, IL13RA1, ZCCHC12, LONRF3, KIAA1210, PGRMC1, SLC25A43, SLC25A5, CXorf56, UBE2A
chrX	138827948	139796338	M14,M2	RNU6ATAC23P, MIR505, HNRNPA3P3, RN7SL727P, RPS17P17, ATP11C, CXorf66, SOX3, LINC00632
chrX	139796338	150911060	M2	ELL2P4, CXorf51B, CXorf51A, MIR513C, MIR513B, MIR513A1, MIR513A2, MIR506, MIR507, MIR508, MIR514B, MIR509-2, MIR509-3, MIR509-1, MIR510, MIR514A1, MIR514A2, MIR514A3, RNU6-382P, FMR1-AS1, FMR1-IT1, FTH1P8, RPL7L1P11, AFF2-IT1, RN7SKP267, RNU7-98P, IDSP1, LINC00893, TMEM185AP1, MAGEA7P, DUTP4, MAGEA8-AS1, LINC00894, MIR2114, XRCC6P2, RNU6-383P, RPL19P21, MIR4330, GPR50-AS1, MIR320D2, RNU6-3P, RBMX2P2, SPANXA2-OT1, RNA5SP516, RN7SKP81, RN7SKP149, PGBD4P6, MTND1P33, HNRNPH1P2, RRM2P4, HNRNPCP10, RN7SKP189, CYCSP44, MIR890, MIR888, MIR892A, MIR892B, MIR891B, MIR891A, RNA5SP517, LINC00632, CDR1, SPANXB2, SPANXB1, LDOC1, SPANXC, SPANXA1, SPANXA2, SPANXD, MAGEC3, MAGEC1, MAGEC2, SPANXN4, SPANXN3, SLITRK4, SPANXN2, UBE2NL, SPANXN1, SLITRK2, TMEM257, FMR1, FMR1NB, AFF2, IDS, CXorf40A, MAGEA9B, HSFX2, TMEM185A, MAGEA11, HSFX1, MAGEA9, MAGEA8, CXorf40B, MAMLD1, MTM1, MTMR1, CD99L2, HMGB3, GPR50, VMA21, PASD1, PRRG3, FATE1, CNGA2
chrX	150911060	151284195	M14,M2	MIR224, MIR452, MAGEA5, CNGA2, MAGEA4, GABRE
chrX	151284195	155240482	M2	MAGEA5, RNU6-764P, MIR105-1, MIR767, MIR105-2, KRT8P8, CSAG3, CSAG4, CSAG2, RN7SL190P, RN7SL667P, KRT18P48, NAA10, RPL18AP16, RN7SL687P, CYCSP45, HCFC1-AS1, MIR3202-2, MIR3202-1, MIR718, SNORA70, RN7SL697P, RN7SL742P, ATF4P2, ATF4P1, IKBKGP1, OR3B1P, SNORA36A, SNORA56, HMGN1P37, MIR1184-1, EEF1A1P31, RENBP, MIR1184-3, MIR1184-2, TMLHE-AS1, AMDP1, DPH3P2, TCEB1P24, TRPC6P, HCFC1, TMEM187, IRAK1, MECP2, OPN1LW, TEX28P2, OPN1MW, TEX28P1, OPN1MW2, TEX28, TKTL1, FLNA, EMD, RPL10, DNASE1L1, TAZ, ATP6AP1, GDI1, FAM50A, PLXNA3, LAGE3, UBL4A, SLC10A3, FAM3A, G6PD, IKBKG, CTAG1A, CTAG1B, CTAG2, GAB3, DKC1, MPP1, SMIM9, F8, H2AFB1, F8A1, FUNDC2, MTCP1, CMC4, BRCC3, VBP1, RAB39B, CLIC2, H2AFB2, F8A2, F8A3, H2AFB3, TMLHE, SPRY3, VAMP7, IL9R, MAGEA10, GABRA3, GABRQ, MAGEA6, MAGEA2B, MAGEA12, CSAG1, MAGEA2, MAGEA3, CETN2, NSDHL, ZNF185, PNMA5, PNMA3, PNMA6C, PNMA6A, PNMA6D, PNMA6B, MAGEA1, ZNF275, ZFP92, TREX2, HAUS7, BGN, ATP2B3, FAM58A, DUSP9, PNCK, SLC6A8, BCAP31, ABCD1, PLXNB3, SRPK3, IDH3G, SSR4, PDZD4, L1CAM, AVPR2, ARHGAP4

Supplementary Table S8

Mutations reported in Figures 1, 4 and 5 and found in TCGA and Cosmic databases

**Genes**	**Sample**	**Mutation**	**TCGA**	**Cosmic**
BRAF	M8,M4,M9,M11	V600E	0.37	ID=COSM476;OCCURENCE=3(adrenal_gland),3(urinary_tract),4(genital_tract),7(pancreas),16(liver),68(eye),2(prostate),4161(large_intestine),21(biliary_tract),11(breast),4377(skin),9(small_intestine),1(autonomic_ganglia),20(pituitary),242(ovary),32(soft_tissue),7(testis),459(haematopoietic_and_lymphoid_tissue),9534(thyroid),78(lung),1(meninges),12(upper_aerodigestive_tract),717(NS),15(bone),2(oesophagus),4(stomach),228(central_nervous_system)
BRAF	M14	D594N	0.002932551	ID=COSM27639;OCCURENCE=1(lung),1(haematopoietic_and_lymphoid_tissue),8(skin),1(small_intestine),7(large_intestine)
KRAS	M14	A146T		ID=COSM1165198,COSM19404;OCCURENCE=91(large_intestine),2(small_intestine),1(upper_aerodigestive_tract),11(haematopoietic_and_lymphoid_tissue)
ARAF	M8	V340L		
ARAF	M14	P194A/Q		
NF1	M16	Q1815X		ID=COSM3402744,COSM41804;OCCURENCE=1(central_nervous_system),1(soft_tissue)
NF1	M9	R1276X		ID=COSM1177899,COSM30762;OCCURENCE=1(haematopoietic_and_lymphoid_tissue),2(soft_tissue),1(endometrium)
PTEN	M16	E73V		ID=COSM5235;OCCURENCE=1(endometrium)
EGFR	M2	L654I		
FGFR4	M16	W655X		
FGFR4	M2	S375P		
ERBB3	M9	G1043V		
ERBB4	M16	G146E		
ERBB4	M14	K361M		
PPP6C	M11	P20L		
MET	M16	S233F		
KIT	M16	L576P	0.002932551	ID=COSM1290;OCCURENCE=1(haematopoietic_and_lymphoid_tissue),2(testis),2(NS),2(thymus),2(large_intestine),1(genital_tract),53(skin),2(breast),19(soft_tissue)
KIT	M2	D816Y		ID=COSM1310;OCCURENCE=1(large_intestine),1(genital_tract),4(ovary),40(haematopoietic_and_lymphoid_tissue),4(testis)
GRIA1	M16	G184E	0.005865103	
GRIA1	M8	splicing		
GRIA1	M9	G462E		
GRM3	M16	S255F		
GRM8	M16	R651Q		ID=COSM3698018,COSM203268;OCCURENCE=1(skin),2(large_intestine)
GRM8	M16	S129L	0.002932551	ID=COSM222142;OCCURENCE=1(skin)
GRIN3A	M4	E1105G		
GRIN3A	M9	R708Q	0.002932551	
GRIN3B	M14	A488G		
TP53	M1	R196X	0.008797654	ID=COSM1640847,COSM99667,COSM99666,COSM99668,COSM3378446,COSM99665,COSM10705;OCCURENCE=10(haematopoietic_and_lymphoid_tissue),9(lung),8(upper_aerodigestive_tract),17(oesophagus),13(central_nervous_system),11(stomach),1(liver),1(kidney),9(pancreas),6(urinary_tract),42(large_intestine),2(biliary_tract),10(skin),22(breast),11(ovary)
TP53BP2	M16	C49S		
TP53BP2	M11	S564R		
BRCA1	M1	splicing		
TSC2	M11	G113S/D		
TSC2	M14	R901C		ID=COSM1230660,COSM1230661;OCCURENCE=1(large_intestine),1(prostate)
CHD9	M11	R1988C		ID=COSM971432;OCCURENCE=1(endometrium)
CHD9	M14	A460D		
NALCN	M2	V1152I		
NALCN	M14	H1531Y	0.002932551	
NOTCH4	M11	A1063T		
KMT2C	M4	D348N		ID=COSM228110;OCCURENCE=1(skin)
KMT2C	M17	V919L		ID=COSM1131647;OCCURENCE=1(prostate),1(thyroid)
KMT2C	M11	R380L		ID=COSM225885;OCCURENCE=2(skin)
KMT2C	M11	C391X		ID=COSM230133;OCCURENCE=1(NS)
KMT2C	M2	R284Q		ID=COSM1179107;OCCURENCE=1(large_intestine),2(prostate),3(breast)
SMARCA2	M6	Q233P		
SMARCA2	M11	A1389S		
PREX1	M4	P1028L		
PREX1	M6	E462A		
SMG1	M17	G1875V		
SMG1	M16	P902S		

CNOT1	M17	Q1597H	
CNOT1	M16	P1027S	
SLIT2	M4	L224F	
SLIT3	M4	S268L	
SLIT3	M9	G575R	
ROBO2	M11	splicing	
ROBO4	M11	T404I	
EPHA8	M16	A451G	
SEMA3D	M11	E115K	
SEMA3F	M4	splicing	
SEMA4B	M16	S231F	
SEMA6B	M11	A307V	
SEMA6B	M11	P275L	
NOTCH2	M4	N922S	
NOTCH3	M4	R1669H	ID=COSM992073,COSM1589832;OCCURENCE=1(endometrium)
NCOR1	M11	splicing	
HEY2	M11	L253F	
LNX2	M9	L278F	
GLI1	M9	T1045A	
GLI2	M16	P1013S	
GLI2	M16	P1013L	
HHIP	M16	R128K	
VEGFA	M9	S256F	
MST1	M16	splicing	
MST1	M9	splicing	
MST1R	M16	P521L	
MST1R	M11	H79N	
NTRK1	M2	H291Y	
FLT1	M11	S1322C	
ROS1	M16	W1477X	
ROS1	M16	S615F	
ASXL3	M16	S1721Y	
ASXL3	M9	S1721F	
KMT2B	M9	F1225S	
CHD2	M16	S260N	
CHD6	M11	S2548F	
CHD7	M11	E2972X	
SMARCA1	M16	R716K	
SUV39H1	M17	V321L	
SETBP1	M11	P462H	
SETBP1	M11	P906S	
PRC1	M16	W26X	
SMYD1	M9	R449H	
PRDM10	M8	Q1037P	
REST	M1	P784L	
PRDM9	M16	E496K	
PRDM16	M16	S436F	
TET1	M16	I843K	
